# Integrative genomic analysis of blood pressure and related phenotypes in rats

**DOI:** 10.1242/dmm.048090

**Published:** 2021-05-19

**Authors:** Fumihiko Takeuchi, Yi-Qiang Liang, Masato Isono, Michiko Tajima, Zong Hu Cui, Yoko Iizuka, Takanari Gotoda, Toru Nabika, Norihiro Kato

**Affiliations:** 1 Department of Gene Diagnostics and Therapeutics, Research Institute, National Center for Global Health and Medicine, Tokyo 162-8655, Japan; 2 Department of Functional Pathology, Shimane University Faculty of Medicine, Izumo 693-0021, Japan; 3 Department of Diabetes and Metabolic Diseases, Graduate School of Medicine, University of Tokyo, Tokyo 113-0033, Japan; 4 Department of Metabolism and Biochemistry, Kyorin University Faculty of Medicine, Tokyo 181-8611, Japan

**Keywords:** Gene, Locus, Transcriptome, Blood pressure, Multifactorial disease, Genome-wide association studies

## Abstract

Despite remarkable progress made in human genome-wide association studies, there remains a substantial gap between statistical evidence for genetic associations and functional comprehension of the underlying mechanisms governing these associations. As a means of bridging this gap, we performed genomic analysis of blood pressure (BP) and related phenotypes in spontaneously hypertensive rats (SHR) and their substrain, stroke-prone SHR (SHRSP), both of which are unique genetic models of severe hypertension and cardiovascular complications. By integrating whole-genome sequencing, transcriptome profiling, genome-wide linkage scans (maximum *n*=1415), fine congenic mapping (maximum *n*=8704), pharmacological intervention and comparative analysis with transcriptome-wide association study (TWAS) datasets, we searched causal genes and causal pathways for the tested traits. The overall results validated the polygenic architecture of elevated BP compared with a non-hypertensive control strain, Wistar Kyoto rats (WKY); e.g. inter-strain BP differences between SHRSP and WKY could be largely explained by an aggregate of BP changes in seven SHRSP-derived consomic strains. We identified 26 potential target genes, including rat homologs of human TWAS loci, for the tested traits. In this study, we re-discovered 18 genes that had previously been determined to contribute to hypertension or cardiovascular phenotypes. Notably, five of these genes belong to the kallikrein–kinin/renin–angiotensin systems (KKS/RAS), in which the most prominent differential expression between hypertensive and non-hypertensive alleles could be detected in rat *Klk1* paralogs. In combination with a pharmacological intervention, we provide *in vivo* experimental evidence supporting the presence of key disease pathways, such as KKS/RAS, in a rat polygenic hypertension model.

## Abstract (precis)

**Summary:** Genome sequencing, transcriptome profiling and genetic mapping, with utilization of human transcriptome-wide association study data, identified 26 potential target genes that regulate blood pressure and related phenotypes in an animal model of hypertension.

## INTRODUCTION

Elevated blood pressure (BP) is prevalent in adults (>25%) (Kearney et al., 2005) and constitutes a major risk factor for cardiovascular diseases, such as stroke and coronary heart disease, accounting for at least 13% of annual deaths globally (Forouzanfar et al., 2017). BP is a complex trait, determined by genetic, environmental and demographic factors and their interaction, where the heritability is estimated to be between 20% and 50% (Salfati et al., 2015). To clarify the genetic basis of BP elevation, studies of molecular genetics have been performed by two tracks, one in humans and another in rodent models of hypertension, especially inbred hypertensive rats.

In humans, recent progress in large-scale genome-wide association studies (GWASs) has succeeded in the identification of over 1000 genetic loci influencing BP (Cabrera et al., 2019). Despite the large number of loci, the genetic variance explained by all loci in aggregate remains low (at most 5.7% of BP variation), indicating that individual common variants mostly exert weak effects on BP. Because complex traits such as BP are mainly associated with non-coding variants, it is suggested that GWAS causal variants often function through regulatory effects on target gene expression (Gallagher and Chen-Plotkin, 2018). Moreover, it has become known that GWAS loci are difficult to interpret due to their complex genetic architecture; i.e. multiple signals, multiple variants and/or multiple genes may be located even at a single locus (Cannon and Mohlke, 2018).

In rodents, several strains of genetically hypertensive rats, including spontaneously hypertensive rats (SHR), have been developed by selective inbreeding (Lerman et al., 2019) and used for quantitative trait locus (QTL) mapping to identify chromosomal regions harboring genes affecting BP (Nabika et al., 2012; Rapp, 2013; Pravenec et al., 2014). Earlier studies have discovered significant linkage signals on a number of chromosomes in experimental crosses produced between hypertensive and non-hypertensive rat strains (Cowley, 2006). It has been shown that although a single broad peak is observable spanning a large interval (usually 20–30 cM), refinement of a QTL map often leads to ghost peaks, which may derive from multiple closely linked loci in the corresponding region (Uleberg and Meuwissen, 2007). Consequently, as the exploration of decay of linkage disequilibrium (LD) is not helpful for fine mapping in rats, congenic mapping has been conducted in combination with gene expression profiling after genome-wide linkage scans (Petretto et al., 2006; Rapp, 2013). Thus, several target genes have been reported to impact hypertension and related phenotypes in rats (Cusi et al., 1997; Garrett and Rapp, 2003; Monti et al., 2008; Pravenec et al., 2008; McDermott-Roe et al., 2011), but the number of such genes is still limited.

Historically, two tracks, in humans and rats, have been pursued in parallel and interactively to clarify the genetic basis of essential hypertension (Padmanabhan and Joe, 2017). Following GWASs of BP traits in humans, although a series of bioinformatic approaches have been done, they cannot sufficiently bridge a gap between statistical evidence for numerous QTLs and a functional understanding of the key disease pathways and biological processes (Gallagher and Chen-Plotkin, 2018).

**Fig. 1.**
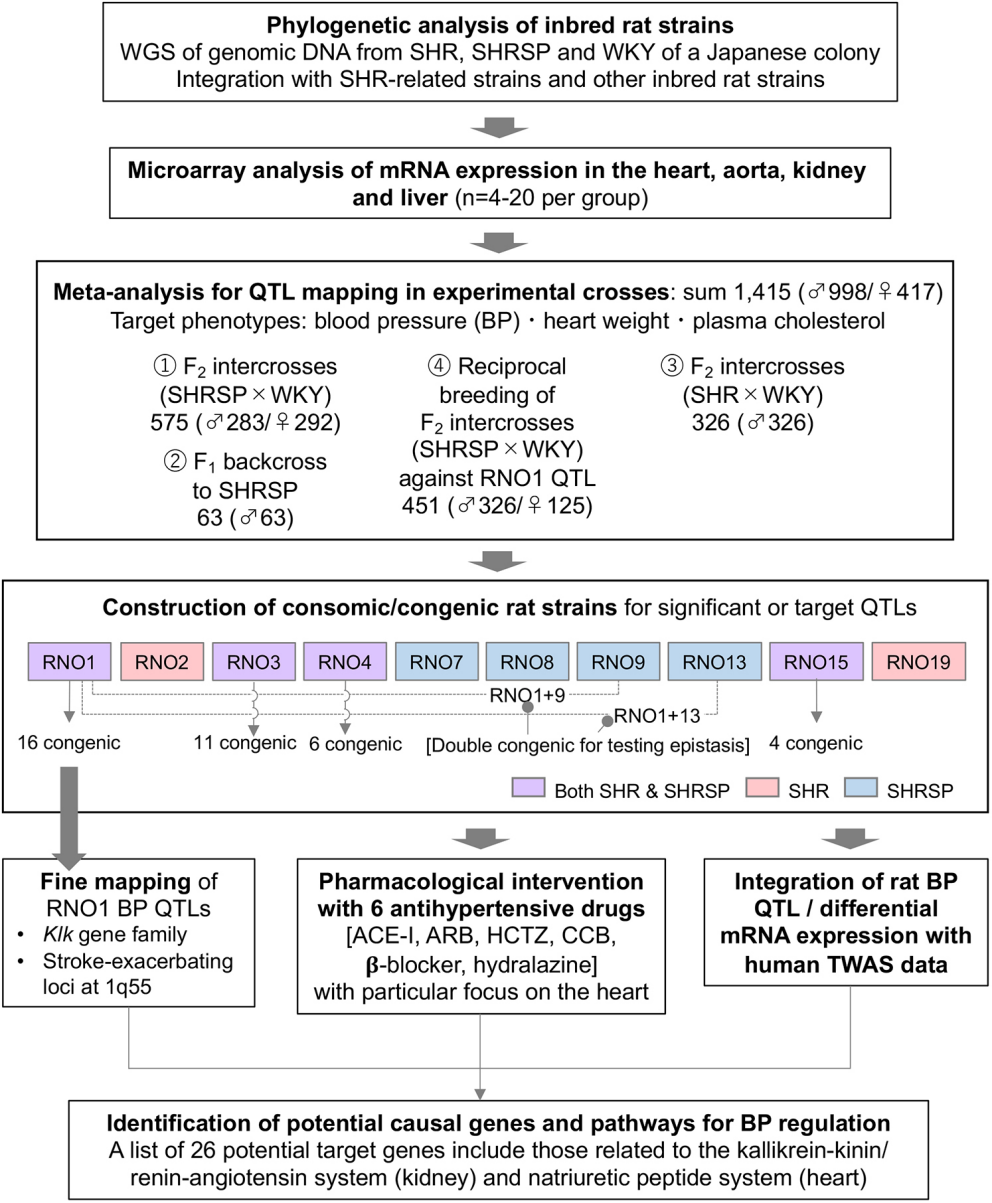
**Overview of study design.** Integrative genomic analysis involves whole-genome sequencing (WGS), gene expression analysis, meta-analysis of genome-wide linkage scans, fine congenic mapping, pharmacological intervention and comparative analysis with human transcriptome-wide association study (TWAS) datasets. ACE-I, angiotensin-converting enzyme inhibitor; ARB, angiotensin receptor blocker; BP, blood pressure; CCB, calcium-channel blocker; HCTZ, hydrochlorothiazide; QTL, quantitative trait locus; SHR, spontaneously hypertensive rats; SHRSP, stroke-prone SHR; WKY, Wistar Kyoto rats.

Accordingly, we perform integrative genomic analysis of BP and related phenotypes in SHR and their substrain, stroke-prone SHR (SHRSP), both of which are unique genetic models of severe hypertension and cardiovascular complications (Okamoto and Aoki, 1963; Nagaoka et al., 1976; Nabika et al., 2012). By integrating whole-genome sequencing (WGS), comprehensive gene expression analysis, meta-analysis of genome-wide linkage scans, fine congenic mapping, pharmacological intervention and comparative analysis with transcriptome-wide association study (TWAS) datasets (Gusev et al., 2016; Barbeira et al., 2018), we search causal genes and causal pathways underlying the tested traits (Fig. 1). We report potential target genes and biological networks related to hypertension in this study.

## RESULTS

### Phenotypic and phylogenetic characterization of SHR and SHRSP

**Fig. 2.**
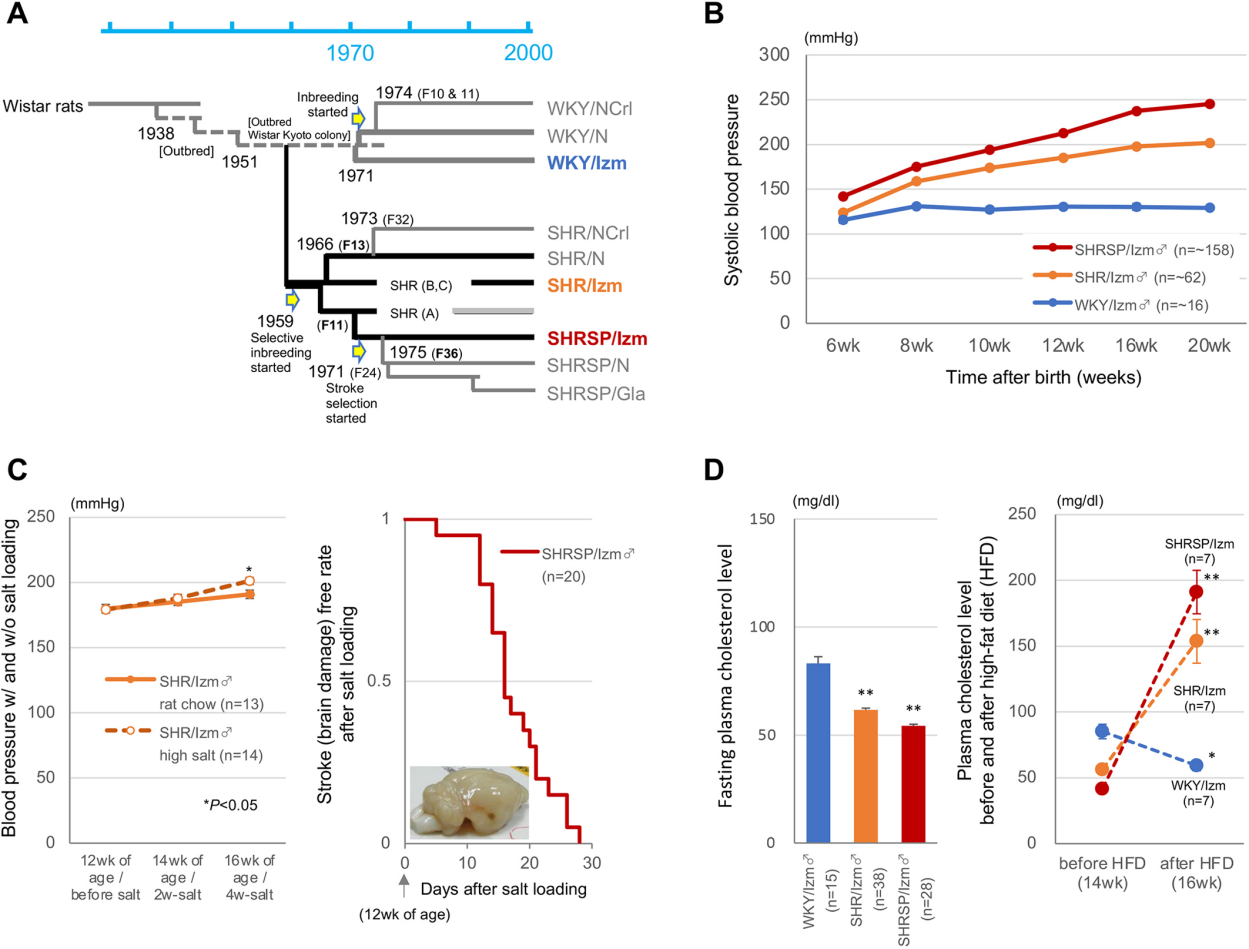
**Genealogy and phenotype characteristics of SHR, SHRSP and WKY.** (A) The genealogy of SHR, their substrains including SHRSP and WKY is schematically shown. (B–D) Inter-strain comparison of phenotypes is made for systolic blood pressure (BP) (B), responses to salt loading (C) and plasma cholesterol level with and without high-fat, high-cholesterol diet (HFD) (28.6% fat and 5% cholesterol) (D). A representative image of brain damage is shown in the inset of panel C. *P<0.05, **P<0.001 (unpaired Student's *t*-test, except for dietary comparison in panel D, for which a paired Student's *t*-test was used). For BP, *P*<0.01 after 8 weeks of age, versus WKY/Izm. Mean±s.e.m.

On standard rat chow, systolic BP was prominently elevated in both SHR and SHRSP compared with their non-hypertensive control strain, Wistar Kyoto rats (WKY) (Fig. 2A), as early as 6 weeks after birth (Fig. 2B; *P*<0.01 after 8 weeks of age). With salt loading, male SHRSP showed enhanced stroke proneness, developing stroke within 30 days; male SHR, although stroke resistant, showed significant BP increase (10 mm Hg, *P*<0.05) compared with the condition without salt loading (Fig. 2C). Although an original colony of SHR had been developed by selective inbreeding on elevated BP (Okamoto and Aoki, 1963), significant alterations in lipid metabolism were concomitantly found for SHR and SHRSP compared with WKY; plasma cholesterol level was significantly decreased on standard rat chow, whereas it was markedly elevated under high-fat, high-cholesterol diet (Fig. 2D).

We performed WGS of SHR, SHRSP and WKY of a Japanese colony (namely, SHR/Izm, SHRSP/Izm and WKY/Izm) and constructed a phylogenetic tree of inbred rat strains (Fig. S1). In this phylogenetic tree, a cluster of SHR substrains was separated from a cluster of WKY substrains, except for WKY/Gla (Atanur et al., 2013). Also, SHR/Izm was clearly differentiated from SHR/N and SHRSP/Izm, in accordance with the genealogy of SHR substrains (Louis and Howes, 1990; Nabika et al., 2012) (Fig. 2A). Looking at Wistar Kyoto colony-derived strains as a whole, we found them to be distant from other inbred hypertensive strains in the phylogenetic tree.

### Haplotype structure of Wistar Kyoto colony-derived strains

Based on WGS data for the present inbred strains, we could reproduce ancestral haplotype, which should have existed in the outbred Wistar Kyoto colony, and defined a strain distribution pattern (SDP) of bi-allelic variants. By computing overlap and non-overlap of SDPs between inbred strains, we detected 1942 segments of ancestral haplotype in the dataset for 13 tested strains. Because SHR had been originally developed from a pair of laboratory rats taken from the outbred Wistar Kyoto colony (Okamoto and Aoki, 1963), there should be one to four alleles at each locus unless contamination took place during the process of inbreeding. After selective inbreeding of SHR had been achieved in 1969, brother–sister inbreeding of WKY was separately initiated to provide non-hypertensive controls for SHR (Louis and Howes, 1990). Focusing on Wistar Kyoto colony-derived strains, we found that the rat genome consisted of 3863 regions (Table S1) with the following cumulative lengths for each number of ancestral haplotype classes: 1518, 254, 5 and 0.05 Mb for two, three, four and five haplotype classes, respectively (Fig. S1). Thus, for >90% of the rat genome, the number of ancestral haplotype classes appeared to be one or two among the Wistar Kyoto colony-derived strains.

**Fig. 3.**
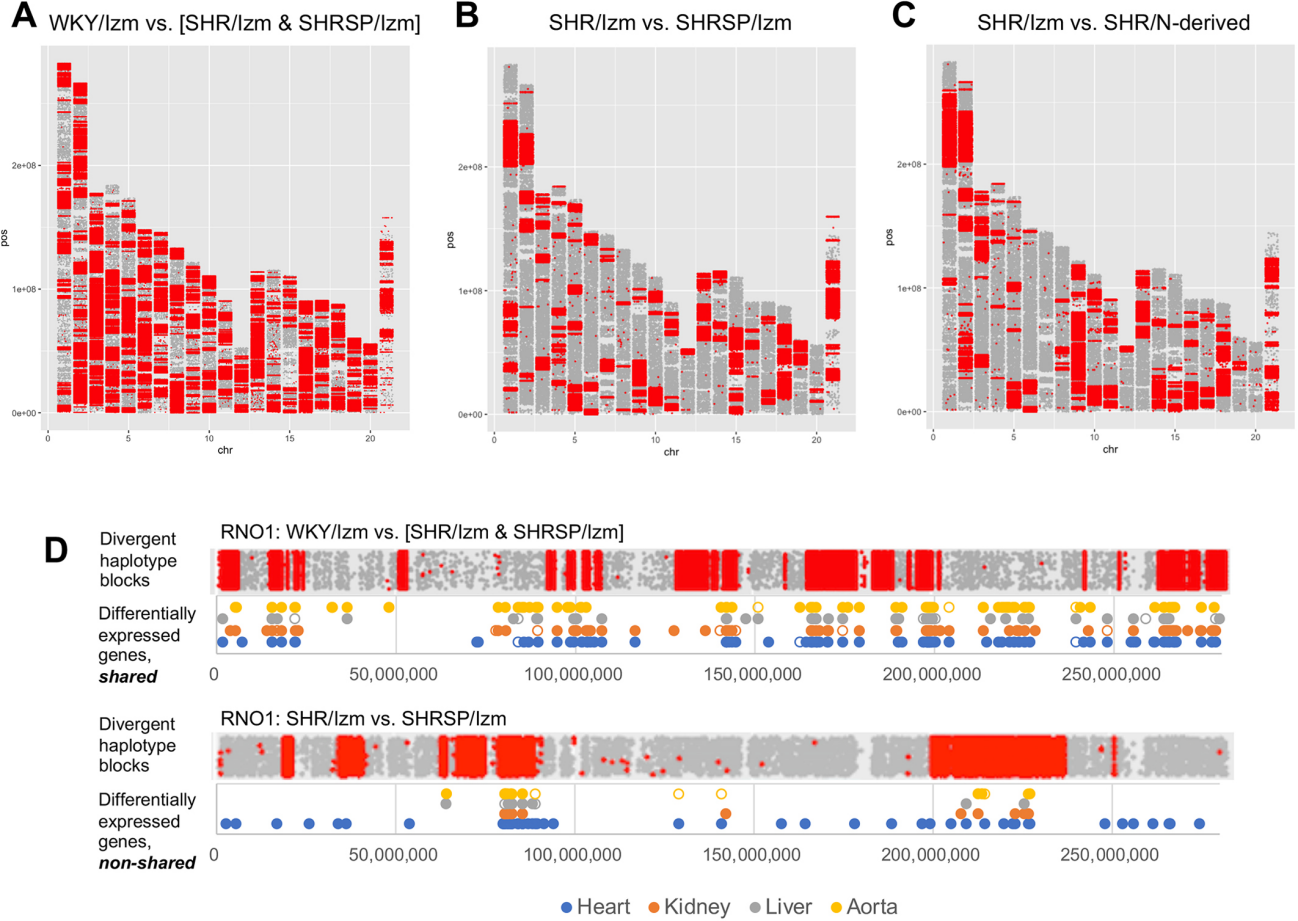
**Inter-strain genetic diversity and its relation to differential mRNA expression.** (A–C) A map of inter-strain genetic diversity between SHR of a Japanese colony (SHR/Izm and SHRSP/Izm) and WKY/Izm (A), between SHR/Izm and SHRSP/Izm (B), and between SHR/Izm and SHR/N-derived strains (C). Red dots indicate SNPs that segregate between the respective strains. (D) The location of significant transcripts is compared with chromosomal patterns of haplotype structure on RNO1. Shared transcripts, with a concordant direction of differential expression in two types of inter-progenitor-strain comparisons (i.e. SHRSP/Izm versus WKY/Izm and SHR/Izm versus WKY/Izm); non-shared transcripts, with differential expression detectable in either of the inter-progenitor-strain comparisons. For each transcript, significant and nominal significant levels of differential expression are depicted in closed and open circles, respectively. See Table S4 for details.

We also found substantial inter-strain genetic diversity between SHR and WKY (Fig. 3A), and between SHR substrains (Fig. 3B,C); e.g. 17% between SHR/Izm and SHRSP/Izm, 19% between SHR/Izm and SHR/N, and 14% between SHRSP/Izm and SHR/N, where the respective values were estimated by calculating the proportion of divergent haplotype blocks in the genome.

### Differential mRNA expression between progenitor strains

As a fundamental resource of molecular profiling, we investigated differentially expressed genes between hypertensive (SHR/Izm or SHRSP/Izm) and non-hypertensive (WKY/Izm) progenitor strains in target tissues using DNA microarray technology. The tissues included the heart, aorta, kidney (whole kidney and renal cortex) and liver. Except for the heart, four to eight samples per strain were analyzed simultaneously for each inter-strain comparison as biological replicates (Table S2). The number of samples was larger in the heart (*n*=16 each for the pair of SHRSP/Izm and WKY/Izm), because they were also used as controls in a pharmacological intervention study below.

Analyzing two datasets for inter-strain comparisons, i.e. SHRSP/Izm versus WKY/Izm and SHR/Izm versus WKY/Izm, we detected significant transcripts (those showing *P*<10^−4^ reproducibly and consistently) for shared differential expression: 846 (heart), 764 (aorta), 413 (whole kidney), 630 (renal cortex) and 328 (liver) transcripts (Table S3). Also, we detected significant transcripts (those showing *P*<10^−8^ in one inter-strain comparison but not in the other) for non-shared differential expression: 568 (heart), 32 (aorta), 53 (whole kidney), 30 (renal cortex) and 12 (liver) transcripts (Table S3).

To discriminate between *cis*- and *trans*-acting influences on gene expression, we compared the location of significant transcripts with haplotype structure. On RNO1, for instance, 90 out of 177 transcripts (51%) for shared differential expression and 28 out of 61 transcripts (46%) for non-shared differential expression in any of the tissues were respectively located in the diverse ancestral haplotype segments, showing approximately half of their distribution patterns concordant with strain differences in question (Fig. 3D; Table S4). Moreover, we listed genes that demonstrated shared differential expression in three or more tissues and were likely to be under *cis*-acting influences (Table S5).

### Linkage analysis in experimental rat crosses

For physical mapping of QTL, we performed meta-analysis of BP, plasma cholesterol and heart weight in experimental rat crosses (*n*=1415 in total), produced by mating SHR/Izm or SHRSP/Izm with WKY/Izm (Fig. 1). Using the combined datasets, we identified genome-wide significant (|Z-score|>3.89) linkage to BP in seven chromosomal regions, of which RNO19 QTL was new in SHR. The most significant and consistent linkage to BP was detected on RNO1, as reported previously (Kato et al., 2003a, 2003b). We also identified significant linkage to plasma cholesterol in seven chromosomal regions, of which two QTLs on RNO14 and RNO19 were new. Linkage to BP and cholesterol phenotypes overlapped on RNO1, RNO15 and RNP19. Further, we identified significant linkage to BP-adjusted heart weight in two chromosomal regions, each on RNO3 and RNO13 (Fig. S2). When linkage analysis was performed separately by sex, there were no significant sex-specific QTLs.

In reciprocal *F*_2_ inter-crosses between SHRSP/Izm and WKY/Izm, we found a potential epistatic interaction of BP QTLs between RNO1 and RNO9, and between RNO1 and RNO13 (nominal *P*_het_<0.05) (Fig. S3), and therefore constructed double congenic strains thereof (1×9pW and 1×13pW) to harbor both chromosomal regions.

### Analysis of consomic and congenic rat strains

Based on the results of current linkage analysis in a Japanese colony and previous congenic mapping in other colonies involving SHR, SHRSP and WKY (Frantz et al., 1998; Hübner et al., 1999; Alemayehu et al., 2002; McBride et al., 2003; Monti et al., 2003; Koh-Tan et al., 2017), we constructed consomic strains for a total of nine chromosomes by using simple sequence length polymorphism markers (Tables S6 and S7): i.e. RNO1, RNO3, RNO4 and RNO15 for both SHR/Izm and SHRSP/Izm; RNO2 and RNO19 for SHR/Izm; and RNO7, RNO8, RNO9 and RNO13 for SHRSP/Izm to be used as a recipient strain (Fig. 1). Compared with the progenitor strains, BP, plasma cholesterol and heart weight significantly changed (i.e. increased or decreased) in the individual consomic and double congenic strains (Table S8), mostly validating the results of current linkage analysis. Although a linkage peak had not reached significant level, BP was significantly reduced in the RNO2 consomic strain, indicating the lack of statistical power in the genome-wide scan.

**Fig. 4.**
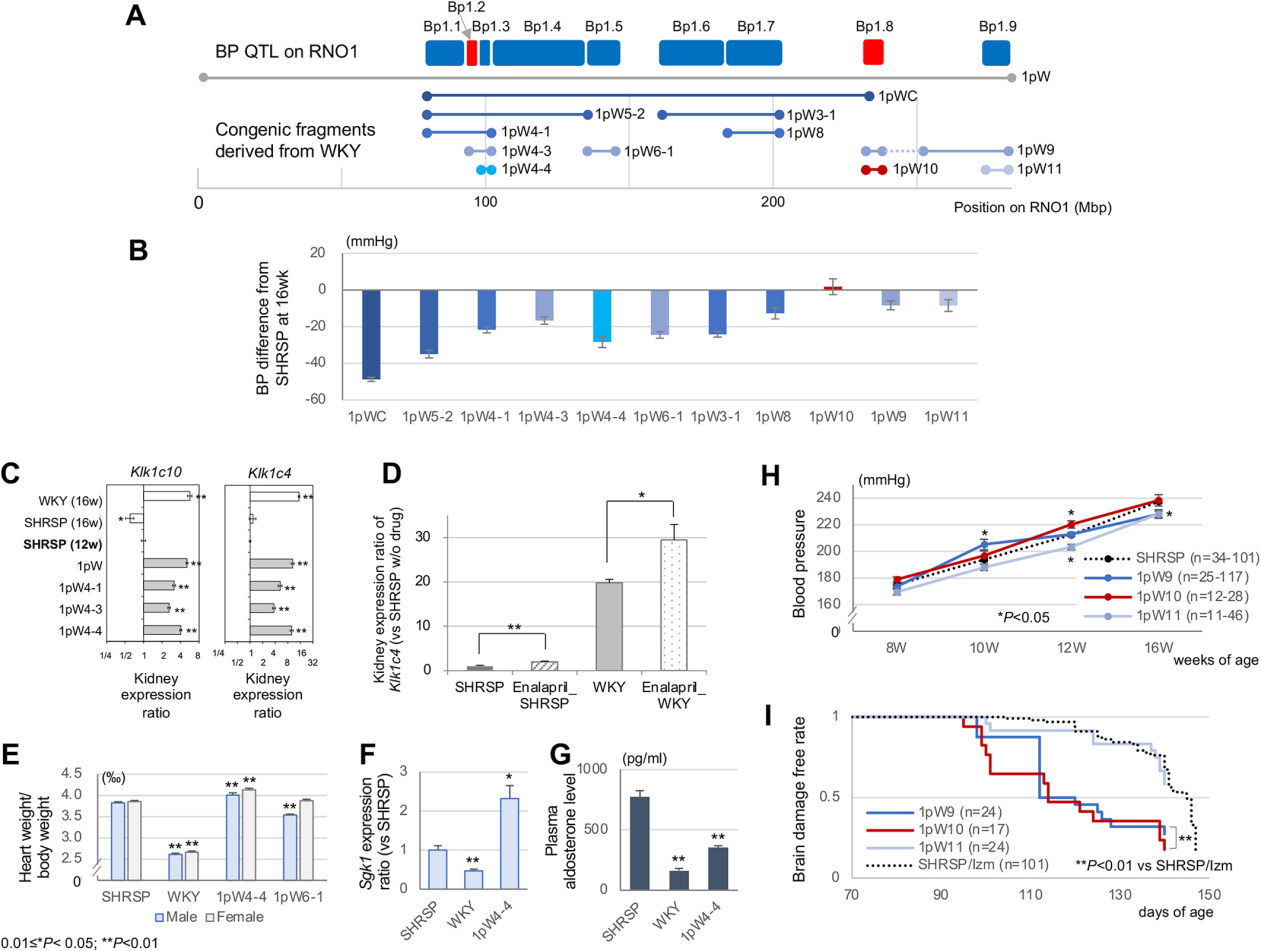
**Fine mapping of BP QTLs on RNO1 and phenotype examination.** (A) Dissection of nine BP QTLs on RNO1 by constructing consomic (1pW) and congenic strains (1pW*; the asterisk indicates a further description of congenic strains) from SHRSP/Izm and WKY/Izm (also refer to Fig. S4). The direction of BP changes at each QTL is indicated in blue (decrease) or red (increase), compared with the recipient progenitor strain (i.e. SHRSP/Izm in this case). (B) BP difference at 16 weeks of age in individual RNO1 congenic strains (refer to Table S9A). (C) Comparison of differential gene expression of *Klk1c10* and *Klk1c4* in the kidney between the relevant RNO1 consomic/congenic strains at 12 weeks of age and the progenitor strains (*n*=5 per group). (D) *Klk1c4* mRNA expression changes in the kidney of progenitor strains (*n*=5 per group) with and without a 4-week administration of enalapril, where the *Klk1c4* mRNA expression level in SHRSP/Izm without drug administration is set to be 1. (E–G) Inter-strain comparison of noticeable phenotypes related to Bp1.3; heart weight divided by body weight (refer to Table S9A) (E), *Sgk1* gene expression in the kidney (F) and plasma aldosterone level (G), between 1pW4-4 and progenitor strains (*n*=5 per group for *Sgk1* and aldosterone). (H,I) Inter-strain comparison of noticeable phenotypes related to Bp1.8; BP at earlier ages (H) and the incidence of brain damage on standard rat chow (I), between 1pW9, 1pW10, 1pW11 and SHRSP/Izm. *P<0.05, **P<0.01 (unpaired Student's *t*-test). Mean±s.e.m.

We further constructed panels of congenic strains from part of the consomic strains, i.e. 1pW and 1SW (RNO1), 3pW and 3SW (RNO3), 4SW (RNO4), and 15pW and 15SW (RNO15) strains (Tables S9 and S10). By using common-segment and sequential methods (Shao et al., 2010) in combination, we successfully identified multiple BP QTLs on RNO1 (Bp1.1 to Bp1.9), RNO3 (Bp3.1 to Bp3.5), RNO4 (Bp4.1 to Bp4.3) and RNO15 (Bp15.1 and Bp15.2) (Fig. 4A,B; Figs S4 and S5, and Table S11). When two or more separate BP QTLs were co-introgressed into a single congenic strain, their composite effects were varied in the manner from additive to contracted; moreover, inter-strain BP differences could be largely explained by an aggregate of BP changes in seven SHRSP-derived consomic strains (Fig. S3).

**Fig. 5.**
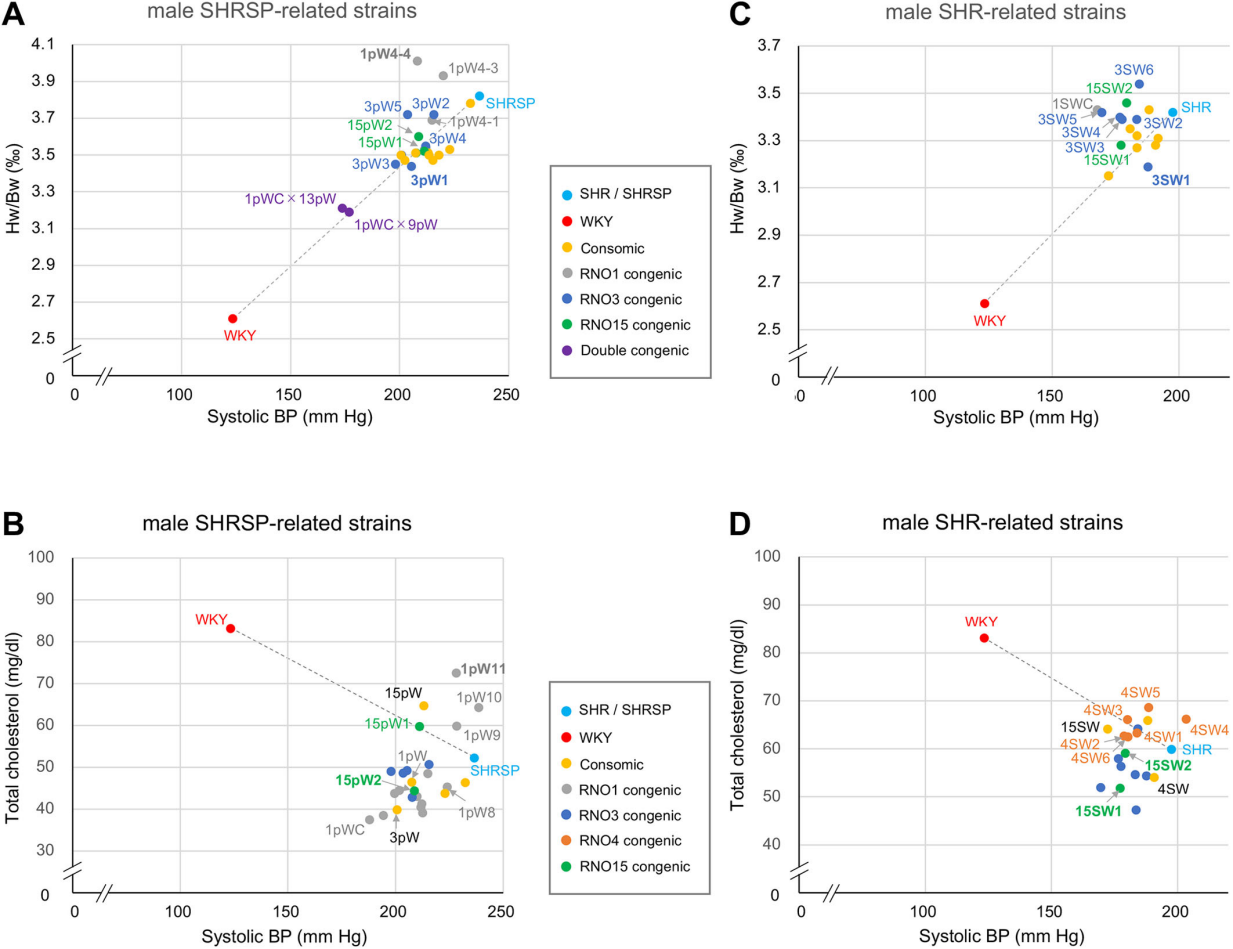
**Correlation of phenotypes measured in panels of consomic and congenic strains.** (A–D) For BP and related phenotypes [i.e. plasma cholesterol and heart weight divided by body weight (Hw/Bw)], the mean values are plotted for each of the consomic (yellow circles), RNO1 congenic (grey circles), RNO3 congenic (dark-blue circles), RNO15 congenic (green circles), RNO4 congenic (orange circles) and double congenic (purple circles) strains as well as the progenitor strains, SHR/Izm and SHRSP/Izm (light-blue circles) and WKY/Izm (red circles). The inter-phenotype correlations for male SHRSP-related strains are shown between systolic BP and Hw/Bw (A) and between systolic BP and plasma cholesterol (B); and similarly for male SHR-related strains between systolic BP and Hw/Bw (C) and between systolic BP and plasma cholesterol (D), where the names of some consomic/congenic strains are provided in the plots for readability and ease of inter-strain comparison. See Tables S8–S10 to further collate a dot in the plot with the corresponding consomic or congenic strain.

Panels of congenic strains allowed us to dissect QTLs not only for a particular trait but also for more than one trait concomitantly in the chromosomal region of interest. For instance, on RNO3, a prominent decrease in both heart weight and BP was similarly detected in 3pW1 and 3SW1, where a reported QTL for cardiac hypertrophy on 3p12 (Inomata et al., 2005; McDermott-Roe et al., 2011) appeared to be successfully isolated (Fig. 5A,B). On RNO15, plasma cholesterol QTL was prominent in 15SW1 but not in 15pW1, whereas BP QTL was significant in both congenic strains (Fig. 5C**,**D), indicating the possible non-pleiotropic regulation at the corresponding loci.

### Dissection of BP QTLs on RNO1

We dissected nine BP QTLs on RNO1 by constructing 15 congenic strains from SHRSP/Izm and WKY/Izm (Fig. S4). For one of the BP QTLs, Bp1.3, we narrowed down the congenic fragment as small as 3.7 Mb in a congenic strain, 1pW4-4, which showed significant BP decrease (−28 mm Hg, *P*=3×10^−13^ in male) compared with SHRSP/Izm (Fig. 4B). By microarray analysis, we first identified highly significant differential expression at *Klk1c3* and *Klk1c9* in the kidney between progenitor strains and the relevant RNO1 congenic strains (1pW4-1, 1pW4-3 and 1pW4-4) (Fig. S6). There was no significant transcript (assumed to be *cis*-eQTL) other than *Klk1c10*, *Klk1c9* and *LOC102556967* in the congenic fragment, by DNA microarray (Table S4). We then validated the differential expression of rat *Klk1* paralogs in the kallikrein gene family (Fig. 4C; Table S12), of which the most prominent differential expression was detected at *Klk1c4* (9.9-fold increase in 1pW4-4, *P*=5×10^−4^). mRNA expression of the *Klk1* paralogs was significantly increased by the administration of enalapril and candesartan in SHRSP/Izm and/or WKY/Izm (Fig. 4D; Fig. S7). Moreover, in 1pW4-4, heart weight (divided by body weight) was significantly increased (*P*=0.003 in males and *P*=4×10^−6^ in females) despite prominent BP reduction, compared with SHRSP/Izm (Figs. 4E and 5A), which accorded with the previous findings in the kidney of rats transgenic for bradykinin (Barros et al., 2018). Regarding this, we found serum and glucocorticoid regulated kinase 1 (*Sgk1*) as a potential mediator (Martín-Fernández et al., 2014); i.e. the *Sgk1* transcript significantly increased in the kidney whereas aldosterone level significantly decreased in the plasma of 1pW4-4 compared with SHRSP/Izm (Fig. 4F,G).

As presumed by the linkage analysis results (Fig. S2), our fine congenic mapping confirmed another BP QTL on 1q55, in which WKY-derived alleles exerted significant BP-increasing effects (Fig. 4A,B). We introgressed WKY-derived hypertensive alleles (at Bp1.8) into 1pW9 and 1pW10, which resulted in significant (*P*<0.05) BP elevation at earlier ages (Fig. 4H) and also the accelerated occurrence of brain damage compared with SHRSP/Izm (Fig. 4I).

### Pharmacological intervention and related physiological pathways

**Fig. 6.**
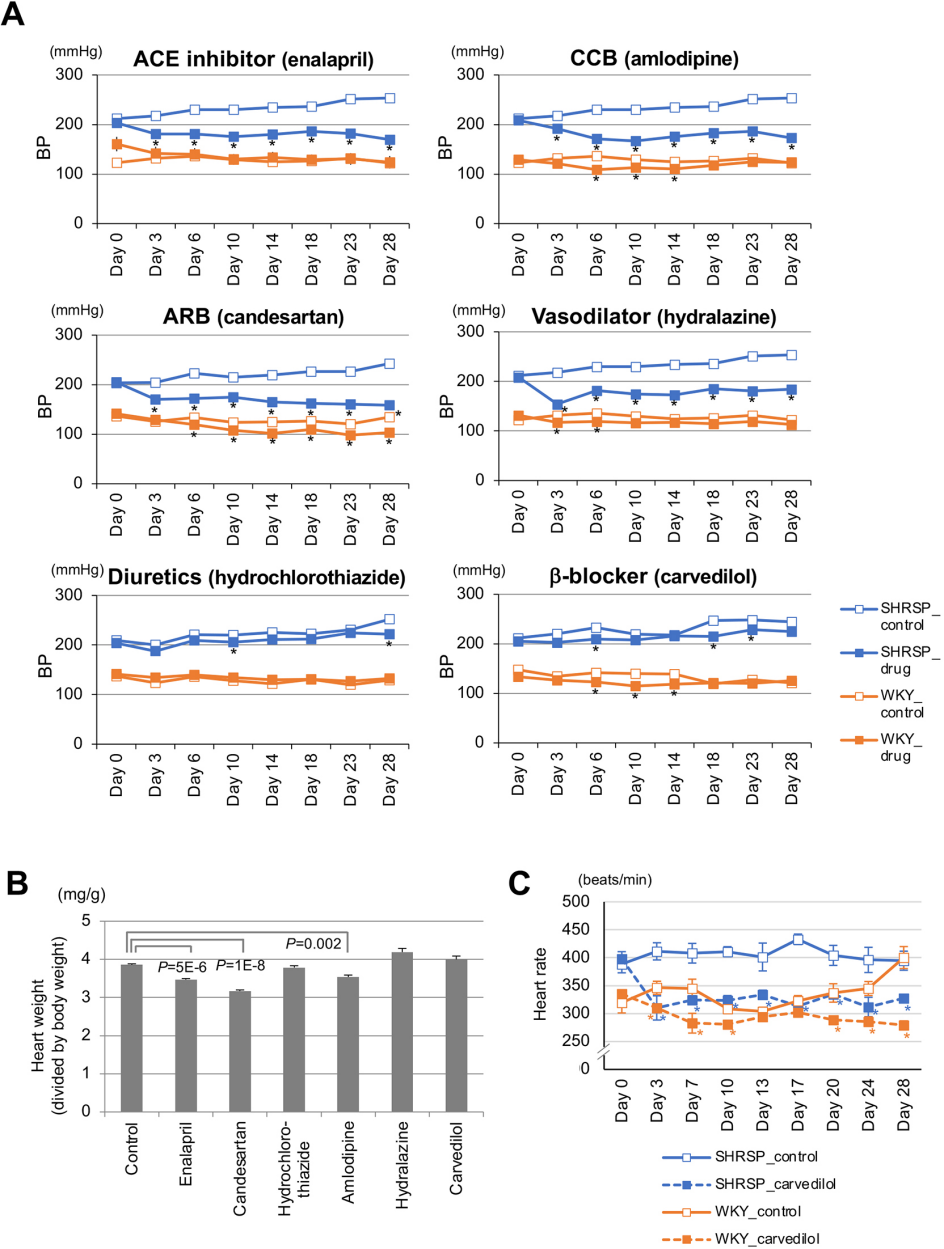
**Hemodynamic changes induced by administration of antihypertensive drugs.** (A) Longitudinal BP changes induced by six classes of antihypertensive drugs (*n*=5-6 per group): enalapril (20 mg/kg/day) from ACE inhibitors, candesartan (5 mg/kg/day) from ARBs, hydrochlorothiazide (20 mg/kg/day) from diuretics, amlodipine (20 mg/kg/day) from calcium-channel blockers (CCBs), hydralazine (10 mg/kg/day) and carvedilol (50 mg/kg/day) from vasodilating β-blockers. (B) Pharmacological impacts on heart weight after a 4-week administration of antihypertensive drugs (*n*=4 per antihypertensive drug group and *n*=34 for control group). (C) Heart rate changes induced by administration of carvedilol (*n*=5-6 per group). *P<0.05 (unpaired Student's *t*-test). Mean ±s.e.m.

In the pharmacological intervention study, we found four antihypertensive drugs, including two inhibitors of the renin-angiotensin system (RAS), enalapril and candesartan, to be effective for markedly lowering BP in SHRSP/Izm (Fig. 6A); three of the four drugs (other than hydralazine) also showed significant amelioration of cardiac hypertrophy (Fig. 6B). Carvedilol, however, did not decrease BP to an appreciable extent, even though it sufficiently suppressed heart rate in SHRSP/Izm and WKY/Izm (Fig. 6C).

To evaluate hemodynamic impacts on transcript in the heart, we meta-analyzed mRNA expression differences between a group of rats undergoing antihypertensive treatment (with any of the four BP-decreasing drugs) and their control group. There was an overall fair correlation of expression changes between antihypertensive treatment and the inter-strain diversity; for some genes (e.g. *Nppb*), we found the presence of drug specificity regarding expression changes (Fig. S8). This appeared to result from the involvement of the relevant genes in the corresponding drug-related pathways and provide potential mechanistic insight (Fig. S7).

In particular, considering the relation between the kallikrein–kinin system (KKS) and RAS, we looked at their respective component genes and detected significant expression changes in some of them (Fig. S7 and Table S13). Besides the *Klk1* paralogs, the *Kng1* and *Prep* genes showed significant shared differential expression, whereas the *Agt*, *Ace* and *Agtrap* genes showed significant non-shared differential expression (Fig. S7). Of the significant genes, the direction of differential expression at *Agt* and *Ace* appeared to be reversed from what is expected, i.e. SHR/Izm-derived and SHRSP/Izm-derived alleles showed BP-decreasing effects compared with WKY/Izm-derived alleles at *Agt* on RNO19 and *Ace* on RNO10, respectively (Table 1). All of the tested target genes were located in the ancestral haplotype blocks that differed between the progenitor strains, and presumed to be under *cis*-acting influences.

**Table 1.**
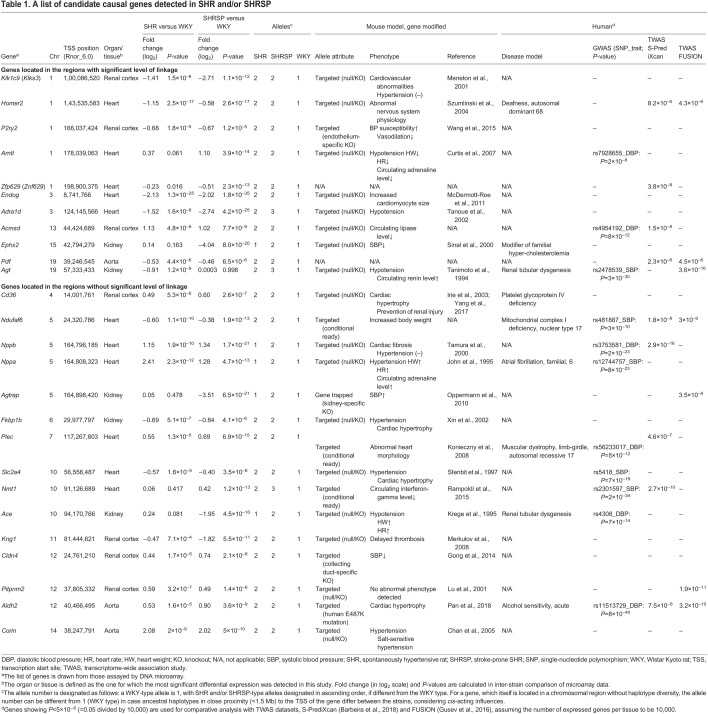
A list of candidate causal genes detected in SHR and/or SHRSP

### Exploration of causal genes for QTLs

We discovered a few examples for the failure to identify significant linkage signals in the BP QTL plot, even though BP QTL should have actually existed. On RNO4, for instance, we detected by fine congenic mapping three separate BP QTLs in a region (3–49 Mb), at a distance from the linkage peak (81–87 Mb) (Fig. S5). In the corresponding region, Bp4.1 was hidden by its adjacent QTL (Bp4.2) with mutually reversed genetic impacts; i.e. BP changes were canceled when both QTLs were concomitantly introgressed into a congenic strain, 4SW4. Here, *Cd36* could be a potential causal gene for Bp4.1; significant inter-strain differential expression was found for *Cd36* in the kidney and liver, although its expression pattern appeared to be different from that previously reported for SHR/N (Gotoda et al., 1999; Pravenec et al., 2008). Also, on RNO5, despite the absence of prominent linkage signals to BP, we found significant differential expression at several genes clustered on 5q36 (Fig. S5), where WKY-derived alleles exerted BP-decreasing effects at *Agtrap* but SHRSP-derived alleles did so at *Nppb* and *Nppa* (Table 1). From the pharmacological intervention and time-course study of inter-strain differential expression (Figs S5 and S8), BP changes did not principally influence the *Nppb* expression level in the heart of SHRSP/Izm.

Furthermore, we inspected a series of genes showing significant differential expression in the target tissues, to see whether they coincided with genes inferable from the study of gene modification in mice and rats and/or TWAS in humans. As a consequence, we could make a list of 26 genes for causal gene prioritization (Table 1).

Of the 26 genes, nine genes (35%) were identified principally by comparative analysis with human TWAS datasets; eight genes, including *P2ry2* (Wang et al., 2015) and *Arntl* (Curtis et al., 2007; Woon et al., 2007), were selected based on phenotypes of gene-deleted mice or rats; two genes [*Endog* (McDermott-Roe et al., 2011) and *Cd36* (Pravenec et al., 2008)] were reported previously; and the remaining seven genes [*Nppb* (Holditch et al., 2015), *Nppa* (John et al., 1995; Flister et al., 2013), *Corin* (Chan et al., 2005), *Ace* (Krege et al., 1995; Esther et al., 1996), *Agt* (Tanimoto et al., 1994), *Adra1d* (Tanoue et al., 2002) and *Ephx2* (Sinal et al., 2000)] were also previously reported but showed the inverted direction of potential allelic impacts on BP between the progenitor strains studied.

### Evaluation of known candidate pathways other than KKS and RAS

Natriuretic peptides (NPs) are synthesized by the heart, brain and other organs, serving as a counter-regulatory system for the RAS. In the present study, gene expression level of *Nppa* and *Nppb* was significantly reduced in a BP-dependent and BP-independent manner, respectively, by administration of antihypertensive drugs in the heart of SHRSP/Izm (Fig. S8); also, differential gene expression was more pronounced for *Nppb* compared with *Nppa* at 6 weeks of age, pre-hypertensive period, in the whole heart between SHRSP/Izm and WKY/Izm (Fig. S5)*.* Thus, the elevated *Nppb* gene expression in the heart of SHRSP/Izm appeared to be determined to some extent by genetic factors. For *Nppa* and *Nppb*, WKY-derived alleles exerted BP-increasing effects in the heart, working in the opposite direction to what is expected (Steinhelper et al., 1990; Holditch et al., 2015). Likewise, *Corin*, another component in the NP system, showed significant inter-strain differential expression in the aorta both between SHRSP/Izm and WKY/Izm (4.07-fold, *P*=5×10^−10^) and between SHR/Izm and WKY/Izm (4.22-fold, *P*=2×10^−9^), in the opposite direction to what is expected (Chan et al., 2005) (Table S13).

Sympathetic nervous system (SNS) effects are mediated by catecholamines that act on G protein-coupled adrenergic receptors (ARs). Vascular contraction is primarily controlled by α1-ARs, of which there are three subtypes, *Adra1a*, *Adra1b* and *Adra1d*. Differential gene expression of *Adra1d* in the heart was highly significant between SHRSP/Izm and WKY/Izm (0.15-fold, *P*=4×10^−25^) and between SHR/Izm and WKY/Izm (0.35-fold, *P*=2×10^−8^), in the opposite direction to what is expected (Tanoue et al., 2002). Similarly, for *Adra1b* and *Adra1a*, there was modest to significant differential gene expression in the heart, in the inverted direction (Table S13). These results appeared to be in accordance with the observation that carvedilol treatment could induce less prominent BP-lowering effects in SHRSP/Izm than other antihypertensive drugs (Fig. 6).

## DISCUSSION

Our integrative genomic analysis identified 26 potential target genes (Table 1) affecting BP and related phenotypes (i.e. plasma cholesterol and heart weight) in SHR and/or SHRSP; only four (of 26) genes – *Endog*, *Cd36*, *Ephx2* and *Arntl* (Woon et al., 2007; Monti et al., 2008; Pravenec et al., 2008; McDermott-Roe et al., 2011) – had previously been reported to show pathophysiological relevance to cardiovascular traits in SHR/SHRSP colonies. By extensive analyses including genome-wide linkage scans (maximum *n*=1415) and fine congenic mapping (maximum *n*=8704), we could provide the polygenic architecture of divergent BP between hypertensive and non-hypertensive strains. To our knowledge, this is the most comprehensive genetic study of hypertension conducted to date in SHR. It has turned out that when pairing the progenitor strains derived from the same outbred colony, the total number of BP loci is smaller (presumably *N*<100) and the allelic effect size is generally larger (change in BP=10–20 mm Hg) in rats than in humans.

It is of particular note that our multi-faceted approach provides substantial evidence for the *Klk1* paralogs as a plausible target gene on 1q22, near which the strongest linkage signal was consistently identified by genome-wide scans in our experimental crosses (Fig. S2). By fine congenic mapping and gene expression profiling, we confirm that the *Klk1* paralogs are *cis*-eQTL and that three *Klk1* paralogs show prominent (>4-fold) differential gene expression in the kidney (Table S12 and Fig. S6).

The human kallikrein locus comprises the *KLK1* gene, which encodes tissue kallikrein (TK), and 14 kallikrein-related genes, *KLK2* to *KLK15*; these homologous genes are clustered on a single locus (Waeckel et al., 2013). During evolution, in primates, the *KLK1* sublocus has repeatedly been duplicated and there are ten functional genes in rats, comprising rat *Klk1* and its nine paralogs (Lundwall, 2013). TK was originally discovered through its BP-lowering effect, later ascribed to enzymatic activity to generate the potent vasodilators kinins from kininogens. TK is synthesized in several organs including the kidney and exocrine glands (Waeckel et al., 2013), and constitutes the main components of the renal KKS, together with angiotensin-converting enzyme (ACE) (Vio et al., 1992). Here, ACE is identical to kininase II rapidly degrading kinins, and both ACE inhibitor and angiotensin receptor blocker can increase the bioavailability of kinins (Siragy et al., 2001). Our results of gene expression changes by a pharmacological intervention (Fig. 4D; Fig. S7) support the biological processes involving the *Klk1* paralogs. Previous studies in TK-deficient mice indicate that TK has antihypertensive effect in the pathological situations of mineralocorticoid excess and salt retention but not in non-hypertensive animals (Meneton et al., 2001; Potier et al., 2013), while another study on gene therapy shows a prolonged reduction in systemic BP by adenovirus-mediated human TK gene delivery into SHR (Wang et al., 2004); all of these findings are in accordance with our results.

The KKS/RAS is found to be one of the key pathways defining BP differences between WKY and SHR or SHRSP; this is further supported by the drug-treatment outcomes (Fig. 6). Besides the *Klk1* paralogs, multiple genes, including *Kng1*, *Prep* (Macconi et al., 2012) and *Agtrap* (Oppermann et al., 2010), appear to contribute to the inter-strain BP differences, whereas two well-known RAS components, *Agt* and *Ace*, have the opposite genetic impacts, according to gene expression analysis in the kidney (Fig. S7). Thus, the pathogenetic roles of individual components in a key pathway should be interpreted with consideration of the direction of an allelic effect on target traits. Also, even if specific genes are not translated to humans, key pathways are likely to be conserved across the species. In the NP system, known to serve as a counter-regulatory system for the RAS, *Nppb* gene expression is significantly elevated in the heart of SHRSP, which is BP independent and genetically defined, at least in part (Figs S5 and S8), working in the opposite direction, i.e. BP reduction, to what is expected (Holditch et al., 2015). Similarly, some components of the SNS, such as *Adra1d* (Tanoue et al., 2002), appear to work in the direction to ameliorate hypertension (Table S13).

Other than the KKS/RAS, there must exist multiple key pathways pathogenetically regulating BP, although the details remain unknown. In exploring such causal pathways, the study of SHR and SHRSP provides definite advantages (Nabika et al., 2012). Above all, because the hypertension alleles were almost fixed by selective inbreeding to *F*_3_ generation in the development of SHR (Okamoto and Aoki, 1963), a relatively small number of causal genes are estimated to control the hypertension trait in SHR (Tanase et al., 1970). For this issue, we could identify significant BP linkage in seven chromosomal regions, where segments of ancestral haplotype, with each containing one or more BP QTL, are inherited from the outbred Wistar colony and assumed to harbor a set of core disease-related genes (Boyle et al., 2017). In addition to the rapid fixation of the hypertensive trait, the availability of substrains (i.e. SHR/Izm, SHRSP/Izm and SHR/N) helps detect and fine map BP QTLs in SHR, in a sense equivalent to *trans*-ethnic GWASs in humans (Morris, 2011). However, there are limitations to the differentiation of multiple QTLs in a chromosomal region, even if we use a series of analytical methods in rodents. As a complementary approach, we compare rat hypertension-related transcriptome data with human TWAS datasets in the identical target tissues, allowing for the nomination of 11 candidate genes (Table 1). Among the genes, *Aldh2* is noteworthy, because it is differentially expressed in the aorta and considered a plausible causal gene on human 12q24 (Kato et al., 2011). Also, *Homer2* is a promising candidate gene located at the BP QTL, Bp1.5, given its physiological role in calcium signaling (Salanova et al., 2013) and potential interaction with the KKS/RAS, as shown by the pharmacological intervention in the heart (Fig. S8).

There are several points that need to be considered in interpreting the current findings. First, the higher statistical power is required to detect QTLs with a complex genetic architecture (e.g. BP loci near *Cd36* on RNO4 and *Agtrap* on RNO5) and less prominent effect (e.g. BP loci on RNO2 and RNO10) by linkage analysis (Fig. S5). Second, multiple layers of evidence are indispensable to draw conclusions about causality, given that inter-strain differential gene expression can be either the cause or the consequence of BP elevation in target tissues (Figs S7 and S8). Third, inter-species comparative analysis using TWAS datasets does not achieve the sufficient coverage of BP loci, because eQTLs have been detected for part of the GWAS loci [<20% in arterial tissue (Evangelou et al., 2018)] in humans. Fourth, *cis*-regulation of genes is primarily estimated in this study by differential gene expression between the progenitor strains, with subsequent confirmation by congenic mapping in a particular region; this differs from experimental approaches generally used for genetical genomics (Breitling et al., 2008). Fifth, QTLs for non-BP traits, such as plasma cholesterol, appear to have been concomitantly fixed with BP QTLs in the development of inbred hypertensive rats (Louis and Howes, 1990), but causal relation between the traits may differ in individual loci. Sixth, it is possible that the biological impacts of some causal genes or pathways are not comparable between the species and can be explored principally in rats, as previously performed for *Endog* (McDermott-Roe et al., 2011). Seventh, substitution mapping with consomic/congenic strains replaces not only alleles with *cis*-acting effect on a trait of interest, but also alleles impacting genes located elsewhere in the genome through epistatic interaction. Eighth, the possible presence of sex-specific QTLs needs to be further explored for BP and related phenotypes in rats, considering sex differences in heritability reported in humans (Ge et al., 2017).

In conclusion, we identify 26 potential target genes that regulate BP and related phenotypes in the animal model of polygenic hypertension. Of the 26 potential targets, five and three genes belong to the KKS/RAS and NP system, respectively. By integrative genomic analysis, we provide *in vivo* experimental evidence supporting the presence of key disease pathways such as KKS/RAS and core disease-related gene loci (e.g. rat *Klk1* paralogs for the KKS/RAS), at least in the inbred hypertensive rats, for the BP trait assumed to be highly polygenic in GWAS. The research resources constructed in the present study will be the foundation to pursue the genetic architecture of complex diseases/traits.

## MATERIALS AND METHODS

### Animal procedures

All animal experiments conformed to the Guidelines for Animal Experiments of National Center for Global Health and Medicine (NCGM), and were approved by the Animal Research Committee of NCGM (permission number 19014).

The name SHR is used for SHRSP and rats with a low incidence of spontaneous stroke, i.e. stroke-resistant SHR (SHRSR), as a whole; it is also used to designate SHRSR for short, in contrast to SHRSP. SHR was originally developed from a pair of rats taken from the outbred Wistar colony – a male rat with BP of 150–170 mm Hg and a female rat with BP a little above average (130–140 mm Hg) for the colony – in Kyoto, Japan by selective inbreeding on elevated BP (Okamoto and Aoki, 1963). The genealogy of SHR has been described elsewhere (Louis and Howes, 1990; Nabika et al., 1991; 2012). Briefly, after selective inbreeding was initiated in 1959, two sublines were separated as early as *F*_12_ generation; SHRSP was further separated from one subline, whereas currently distributed SHR (or SHRSR) was developed from the other subline. Then, in 1966 (at *F*_13_ generation), breeder SHRs were sent to the National Institutes of Health (NIH), USA and established to be an inbred strain of the NIH colony (or SHR/N), independent of a Japanese colony (SHR/Izm). SHR and SHRSP are designated with a suffix (e.g. N, Gla and Izm) to distinguish the colony or stock, from which the rats are derived. After inbreeding of SHR had been achieved in 1969, brother–sister inbreeding of WKY was initiated in Kyoto and at the NIH to provide non-hypertensive controls for SHR. Because non-inbred WKYs were sent from Kyoto to NIH in 1971 and breeding stocks of WKY were distributed to commercial suppliers as early as *F*_10_ generation in 1974 (Fig. 2A), substantial genetic heterogeneity has occurred among different stocks of WKY (Kurtz et al., 1989). All SHRs and SHRSPs of a Japanese colony that we used in the present study had originated from a single colony of Shimane University Faculty of Medicine, Izumo.

Experimental rat crosses were produced by mating SHR or SHRSP with WKY rats – *F*_2_(SHRSP×WKY) (283 male and 292 female), *F*_1_(SHRSP×WKY)×SHRSP backcross (63 male) and *F*_2_(SHR×WKY) (326 male) – as previously described (Kato et al., 2003a, 2003b; Watanabe et al., 2005). In addition, reciprocal *F*_2_ inter-crosses were produced to evaluate a potential epistatic interaction with RNO1 BP QTL (located in a ∼130 Mb fragment), which showed the strongest linkage to BP in *F*_2_ crosses involving SHRSP and WKY of a Japanese colony (Kato et al., 2003b). First, two reciprocal congenic strains were respectively constructed by introgressing a WKY-derived fragment for RNO1 BP QTL into SHRSP [SHRSP.WKY-(*D1Wox18*–*D1Rat116*)/Izm, named as 1pWC for short] and vice versa [WKY.SHRSP-(*D1Wox18*–*D1Rat116*)/Izm, named as 1WpC], as previously described (Wang et al., 2008). Then, two reciprocal *F*_2_ inter-crosses were respectively produced between 1pWC and WKY (136 male and 125 female; all *F*_2_ rats possess WKY alleles for RNO1 BP QTL) and between SHRSP and 1WpC (190 male; all *F*_2_ rats possess SHRSP alleles for RNO1 BP QT) (Fig. S3).

Rats were weaned at 4 weeks after birth and placed on normal rat chow (SP diet, Funabashi Farm, Japan) unless otherwise indicated. Some rats were fed a high-fat, high-cholesterol diet (HFD) (28.6% fat and 5% cholesterol, Funabashi Farm,) after 14 weeks of age. All rats were laboratory animals and treated in compliance with institutional regulations. Systolic BP was measured by the tail-cuff methods, as previously described (Kato et al., 2003a), in which three consecutive BP readings were taken and averaged for each session. A total cholesterol level in plasma was measured with blood samples drawn from the tail vein of the rats in an overnight (16-h) fasting state. The timing of brain damage was evaluated based upon a series of procedures, which included *T*_2_ weighted magnetic resonance imaging, physiological parameters (i.e. BP and body weight changes) and development of neurological/behavioral dysfunction.

The rats were killed under pentobarbital anesthesia (200 mg/kg via intraperitoneal infusion), and the organs (heart, aorta, kidney and liver) were excised and immediately frozen at −70°C for subsequent RNA extraction.

### Construction of consomic and congenic strains

Based on the linkage analysis results, we chose to construct consomic strains for seven chromosomes – RNO1, RNO3, RNO4, RNO9, RNO13, RNO15 and RNO19 – in which significant BP linkage was detected. Although we could not find significant BP linkage in our dataset, we added RNO2 to the list of consomic strains, considering solid evidence for the presence of BP QTL on RNO2 from SHR/SHRSP of other colonies (Alemayehu et al., 2002; McBride et al., 2003). Assuming that QTLs for BP and cholesterol, located in close proximity, happened to have been inherited together through selective inbreeding on elevated BP, we also chose RNO7 and RNO8 (in which cholesterol QTLs were detected) for the construction of consomic strains (Fig. 1).

A speed congenic strategy was used to transfer a single entire chromosome (for chromosome substitution or consomic strains) or a segment of a particular chromosome (for congenic strains) from WKY onto the genetic background of SHR or SHRSP as previously described (Kato et al., 2003b). A total of 242–252 simple sequence length polymorphism (SSLP) markers (Table S4) were selected to cover the rat genome and used for genotyping to construct consomic and congenic strains according to a marker-assisted congenic breeding design. To isolate and dissect QTLs in panels of congenic strains, which were derived from part of the consomic strains first constructed – i.e. 1pW (for RNO1), 3pW and 3SW (for RNO3), 4SW (for RNO4), and 15pW and 15SW (for RNO15) strains – common-segment and sequential methods were used in combination (Shao et al., 2010). Moreover, double congenic strains were constructed between SHRSP and WKY to harbor both RNO1 and RNO9 QTLs (1×9pW strain) and both RNO1 and RNO13 QTLs (1×13pW strain) by mating RNO1 congenic (1pWC) with RNO9 and RNO13 consomic (9pW and 13pW) strains, because a potential epistatic interaction between these chromosomes was indicated by linkage analysis in the reciprocal *F*_2_ inter-crosses mentioned above.

The boundaries of the target regions of the congenic strains are shown in Table S11.

Rat consomic/congenic strains generated in this study have been deposited to the National BioResource Project (NBRP) for the Rat in Japan.

### Linkage analysis

Meta-analysis was performed to combine multiple datasets (*n*=1415 in total), part of which had been reported in the study of individual experimental crosses (Kato et al., 2000; 2003a; 2003b; Inomata et al., 2005; Watanabe et al., 2005; Mashimo et al., 2007). Genotyping was done mostly with SSLP markers as previously described (Kato et al., 2003a), and phenotype values were quantile normalized. Heart weight was adjusted for BP. Linkage scans were performed for each dataset of individual experimental crosses separately by sex, using the qtl package (version 1.45-2) (Broman and Sen, 2009) of the R software. To estimate the underlying genotype at each locus on the genome, the probabilities were calculated every 0.5 cM with the hidden Markov model, assuming an error rate of 0.01 for the observed genotypes of marker loci. At each locus, the genotype–phenotype association was examined under the additive model by using a score test of the SNPTEST software (version 2.5.2) (Marchini and Howie, 2010), which accounts for uncertainties of the true genotype. First, the allelic effect size of one progenitor strain in comparison with the other and its standard error were estimated in individual experimental crosses. Then, the estimates were combined across the experimental crosses by the fixed effect method, with the rmeta package (version 3.0) of the R software. Association was regarded as genome-wide significant when the *P*-value at the locus was <1×10^−4^ (which corresponds to a |Z-score|>3.89), according to the guidelines proposed by Lander and Kruglyak (1995). Here, the Z-score is the number of standard deviations to reflect the amount of variability within a tested dataset. We used the SSLP marker information from the Rat Genome Database (https://rgd.mcw.edu/rgdweb/search/markers.html?100) and the sex-averaged genetic map for rat genome assembly (rn6) (Littrell et al., 2018).

It has been reported that large samples are not always required to identify a biologically meaningful QTL (Wang and Xu, 2019); e.g. *n*≥500 can detect a QTL explaining 5% of the phenotypic variance with an 80% statistical power in the situation in which the polygenic effect size is 1, the LD parameter is 0.5 and the nominal type 1 error is 0.05. Although sample size in the individual experimental crosses was limited principally due to the resource for rat breeding and phenotyping burden, meta-analysis (*n*=1415 in total) allowed for substantial statistical power.

### WGS

WGS was performed in the present study for four inbred strains: three progenitor strains (SHRSP/Izm, SHR/Izm and WKY/Izm) plus a RNO1 congenic strain, 1pW9. DNA libraries were prepared using a TruSeq DNA PCR-free kit (Illumina) and 459–482 million 150-bp paired-end reads were sequenced on a HiSeq×Sequencer (Illumina) with an average read depth of 42–44×. We used the BWA program (version 0.7.15) for mapping the reads to the rn6 reference genome and the GATK program (version 3.8) for variant discovery. For inter-strain comparison and phylogenetic analyses, we used publicly available genome sequences of rat strains (Hermsen et al., 2015) other than four sequenced strains by combining their VCF files, for which we retained genotype calls with a read depth of ≥4 and a genotype quality score of ≥10.

The phylogenetic tree was constructed by the maximum-likelihood method and 100 times of bootstrapping. We used RAxML software (version 8.2.12) (Stamatakis, 2014) and applied the GTR+Gamma model of DNA substitution to the SNPs in the nuclear genome.

### Reconstruction of ancestral haplotype map

Through the process of inbreeding, chromosomes of breeder SHRs and WKYs have experienced recombination. We attempted to reproduce ancestral haplotype, which had originally existed in the outbred Wistar Kyoto colony, based on WGS data for the present inbred strains. Because the ancestral laboratory rats were taken from genetically diverse wild animals, any chromosomal segment could have differed at many variants between ancestral chromosomes that once existed in the colony. A tract of ancestral haplotype that has been transmitted *en masse* to the descendants is detectable as a segment shared between the present inbred strains. Accordingly, in any segment, there should exist one or several distinct classes of haplotype between the inbred rat strains derived from the Wistar Kyoto colony (Louis and Howes, 1990). A group of variants located on such a haplotype segment are in complete LD with each other and allow us to define a SDP of bi-allelic variants throughout the genome. Using this property, we reconstructed a genome map of ancestral haplotype by enumerating all haplotype classes and then computing their overlap between the inbred strains.

Specifically, we used a dataset for 13 Wistar Kyoto colony-derived strains (including SHR/Izm, SHRSP/Izm and WKY/Izm). There were 2,825,721 variants in the dataset for 13 strains, from which we retained variants that could be called homozygous in all strains. Looking at these variants, we defined a segment (or block) of ancestral haplotype as the one in which ≥20 adjacent variants in the SDP were clustered; here, a term, adjacent, was defined such that two consecutive variants in the SDP were separated by <100 non-SDP variants (that do not constitute the SDP in question). This threshold should help to reduce false positives resulting from technical errors of next-generation sequencing, whereas it could fail to detect some, although not many, short segments of ancestral haplotype or could split a long segment of ancestral haplotype into two or more shorter ones. According to this threshold, we detected 1942 segments of ancestral haplotype in the dataset for 13 strains. When a given chromosomal region in the genome does not overlap with any segment of ancestral haplotype, the alleles in that region are considered identical between the inbred strains tested. However, when the region overlaps with one (or two) segment(s) of ancestral haplotype, there must be two (or three) distinct haplotype classes of SDP variant in the corresponding region. By computing such overlap and non-overlap with the custom Perl and R scripts, we estimated the genomic distribution of ancestral haplotype among the inbred strains.

As an approach to evaluating inter-strain genetic diversity, we calculated the proportion of ancestral haplotype segments that were different between the two strains in question.

### Gene expression analysis

The RNA was extracted, the quality of RNA was assessed, and microarray analysis was performed as previously described (Liang et al., 2020) using the Whole Rat Genome Microarray 4×44K (Agilent Technologies). In each array, low-quality probes were filtered out using the Agi4×44PreProcess package of the R software. For a set of arrays used for each specific analysis, the probes with <50% call rates were omitted, and the signals were quantile normalized across the arrays.

For quantitative PCR of mRNA, PCR primer sequences for the target genes were designed originally or taken from literature (Tables S12 and S14).

### Pharmacological evaluation

We chose to use six classes of antihypertensive drugs: enalapril from ACE inhibitors, candesartan from angiotensin receptor blockers (ARBs), hydrochlorothiazide from diuretics, amlodipine from calcium-channel blockers, carvedilol from vasodilating β-blockers and hydralazine. Enalapril and hydrochlorothiazide were provided by Towa Pharmaceutical (Osaka, Japan), candesartan was provided by Takeda Pharmaceutical (Osaka, Japan), amlodipine was provided by Pfizer (New York, NY, USA), carvedilol was provided by Daiichi-Sankyo (Tokyo, Japan), and hydralazine was provided by Sanwa Kagaku Kenkyusho (Nagoya, Japan).

We first conducted a pilot study, in which we tested a range of drug doses previously reported: 10–30 mg/kg/day for enalapril, 1–10 mg/kg/day for candesartan, 20–40 mg/kg/day for hydrochlorothiazide, 10–30 mg/kg/day for amlodipine, 50–100 mg/kg/day for carvedilol and 1–10 mg/kg/day for hydralazine. Based on the results in the pilot study, we determined an appropriate dose for each drug as in the previous study (Ochiai et al., 2008), where we showed part of the pharmacological intervention results (three drugs administered on SHRSP). Systolic BP was measured every 3–4 days for 4 weeks (between 12 and 16 weeks of age, *n*=5-6 per group). After 4 weeks of pharmacological intervention (at 16 weeks of age), the rats were killed under pentobarbital anesthesia (200 mg/kg via intraperitoneal infusion), and the organs (heart and kidney) were excised for RNA analysis.

### Test of candidate genes for BP regulation

We chose a total of 201 genes as part of the candidate genes for BP regulation, by searching for mouse models with the terms ‘blood pressure AND hypertension’ and/or ‘blood pressure AND hypotension’ in the Phenotypes, Alleles & Diseases Query within the Mouse Genome Informatics Database (http://www.informatics.jax.org), and for rat models with gene modification of the corresponding genes. We then compared this list of candidate genes with genes showing shared and non-shared differential expression detected in the present study.

### Comparison with TWAS datasets

TWAS integrates GWAS and gene expression datasets in humans to prioritize the likely causal genes at GWAS loci, which may be related to BP differences between hypertensive and control rat strains as well. Therefore, not for fine mapping of GWAS-identified intervals but for causal gene prioritization, we compared TWAS datasets with differential gene expression in target tissues of the rat. That is, when there are multiple TWAS hits (or genes) in an LD block showing a significant GWAS signal, it is not evident which gene is causal. To filter out a list of potential genes, we selected a TWAS hit that could be concomitantly supported by rat experiments. We downloaded the TWAS results analyzed by the S-PrediXcan (Barbeira et al., 2018) and FUSION (Gusev et al., 2016) software, where we used the GWAS results for diastolic BP and dyslipidemia in the UK Biobank, and those for LDL cholesterol reported by Teslovich et al. (2010), and gene expression datasets for the heart left ventricle, aorta and liver from the Genotype–Tissue Expression project (GTEx) (GTEx Consortium, 2017), and kidney from The Cancer Genome Atlas (TCGA) (Kandoth et al., 2013).

### Microarray analysis

Differential expression for each transcript was tested by multiple regression with log2-transformed intensity and the type of experimental condition (such as strain category and administration of antihypertensive drug) used as the dependent and independent variables, respectively. The variance of expression level was estimated by the empirical Bayesian procedure using the limma package (Ritchie et al., 2015) of the R software on multiple microarray datasets for each target tissue. We made an adjustment for multiple testing of transcriptome by the Benjamini–Hochberg procedure, and regarded a false discovery rate (q-value) of <0.05 as statistically significant. Alternatively, when we examined two sets of data for inter-progenitor-strain comparisons, i.e. SHRSP/Izm versus WKY/Izm and SHR/Izm versus WKY/Izm, we regarded genes that showed *P*<10^−4^ reproducibly in two types of comparison with a concordant direction of differential expression as significant for ‘shared’ differential expression. Also, for differential expression detectable in either of the inter-progenitor-strain comparisons, we regarded genes that showed *P*<10^−8^ in one comparison but not in the other (*P*≥0.05 with a concordant direction or all *P*-values in the opposite direction) as significant for ‘non-shared’ differential expression.

### Statistical analysis

The results are expressed as the means±s.e.m., and differences were analyzed using unpaired Student's *t*-test (two-tailed) when comparing two group means unless otherwise indicated. *P*<0.05 was considered to be nominal significant.

## Supplementary Material

Supplementary information
