# Supplementary material for: Integrative genomic analysis of blood pressure and related phenotypes in rats
Source: Dis Model Mech. 2021 May 19;14(5):dmm048090. doi: 10.1242/dmm.048090 (PMC8188887; doi:10.1242/dmm.048090)
Supplement: Supplementary information [file dmm-14-048090-s1.pdf]

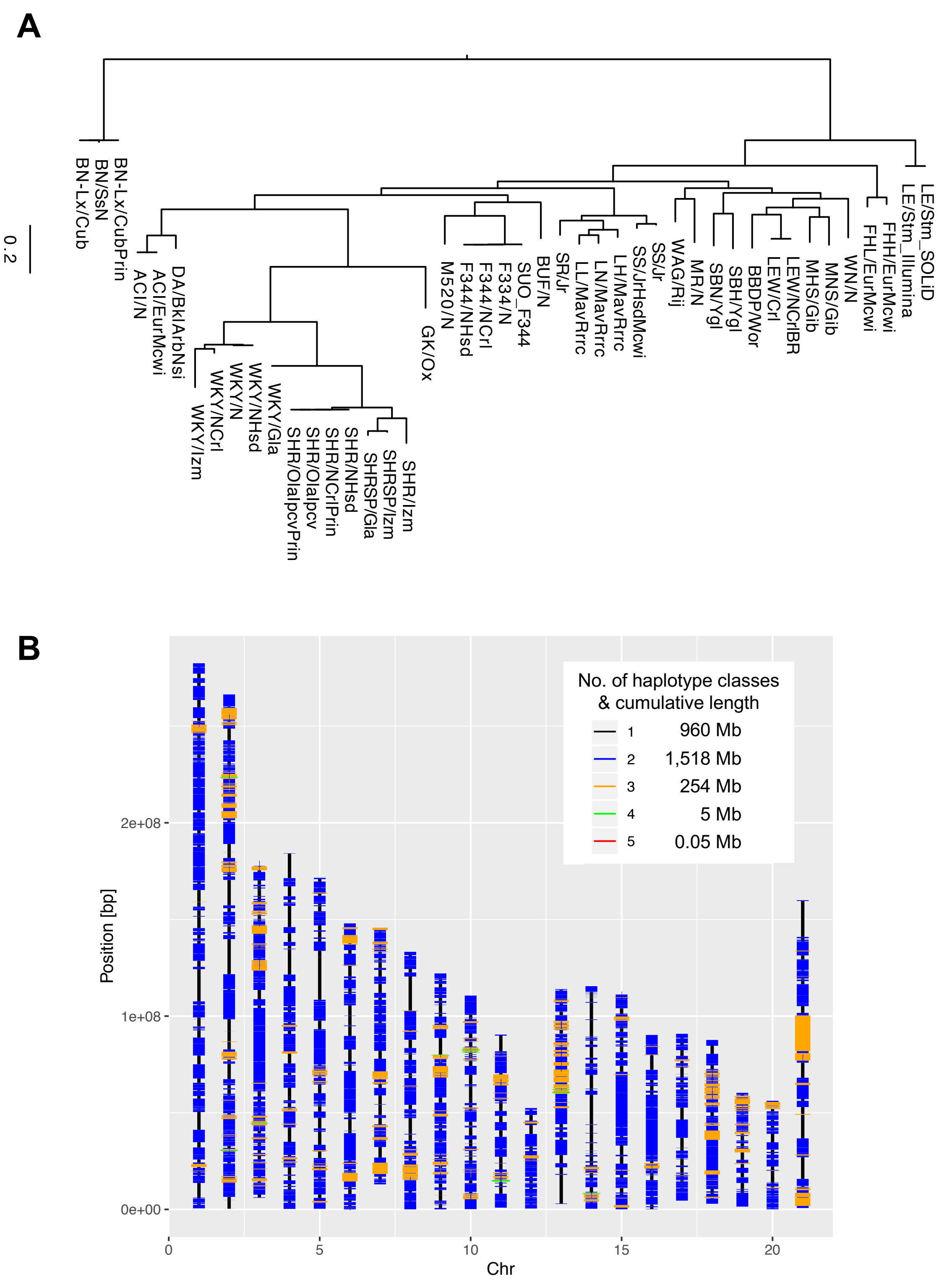

Fig. S1: Phylogenetic tree and ancestral haplotype map. (A) Whole genome sequencing (WGS) data for 12 Wistar Kyoto colony-derived strains and 33 other inbred rat strains are used for the construction of phylogenetic tree. To avoid bias in maximum likelihood estimation, the congenic strain 1pW9 is excluded from phylogenetic inference. **(B)** A map of ancestral haplotype is reconstructed for the Wistar Kyoto colony, based on WGS data for the present inbred strains. A cumulative length of chromosomal regions is shown for each number of ancestral haplotype classes (1 to 5) in the inset.

Disease Models & Mechanisms • Supplementary information

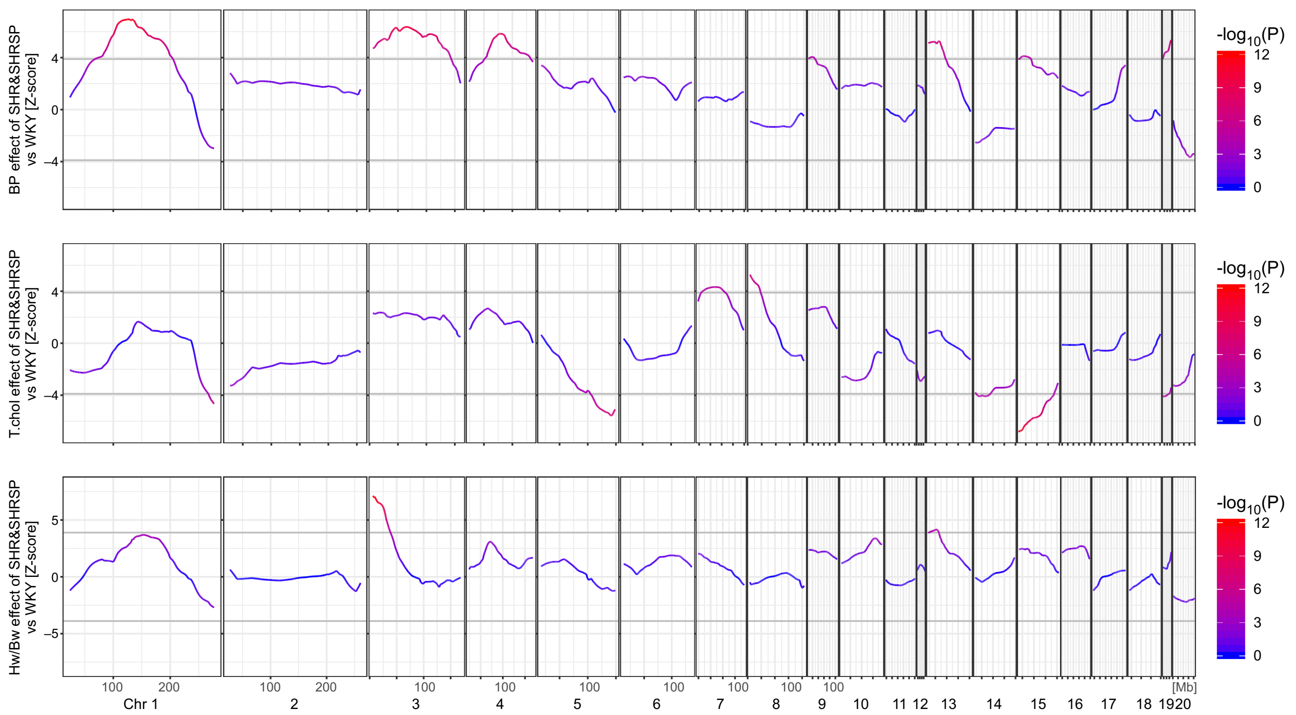

Fig. S2: Meta-analysis of genome-wide linkage scans. Genome-wide quantitative trait locus (QTL) mapping is performed on systolic blood pressure (BP) (in the top panel), plasma total cholesterol (in the middle panel) and heart weight divided by body weight (Hw/Bw) (in the bottom panel) in experimental crosses between WKY/Izm and SHR/Izm and between WKY/Izm and SHRSP/Izm. The underlying genotype is estimated at each locus on the genome, the genotype-phenotype association is examined under the additive model, and the strength of linkage is expressed in Z-score, where |Z-score| >3.89, corresponding to $P<1\times10^{-4}$, is defined to be genome-wide significant.

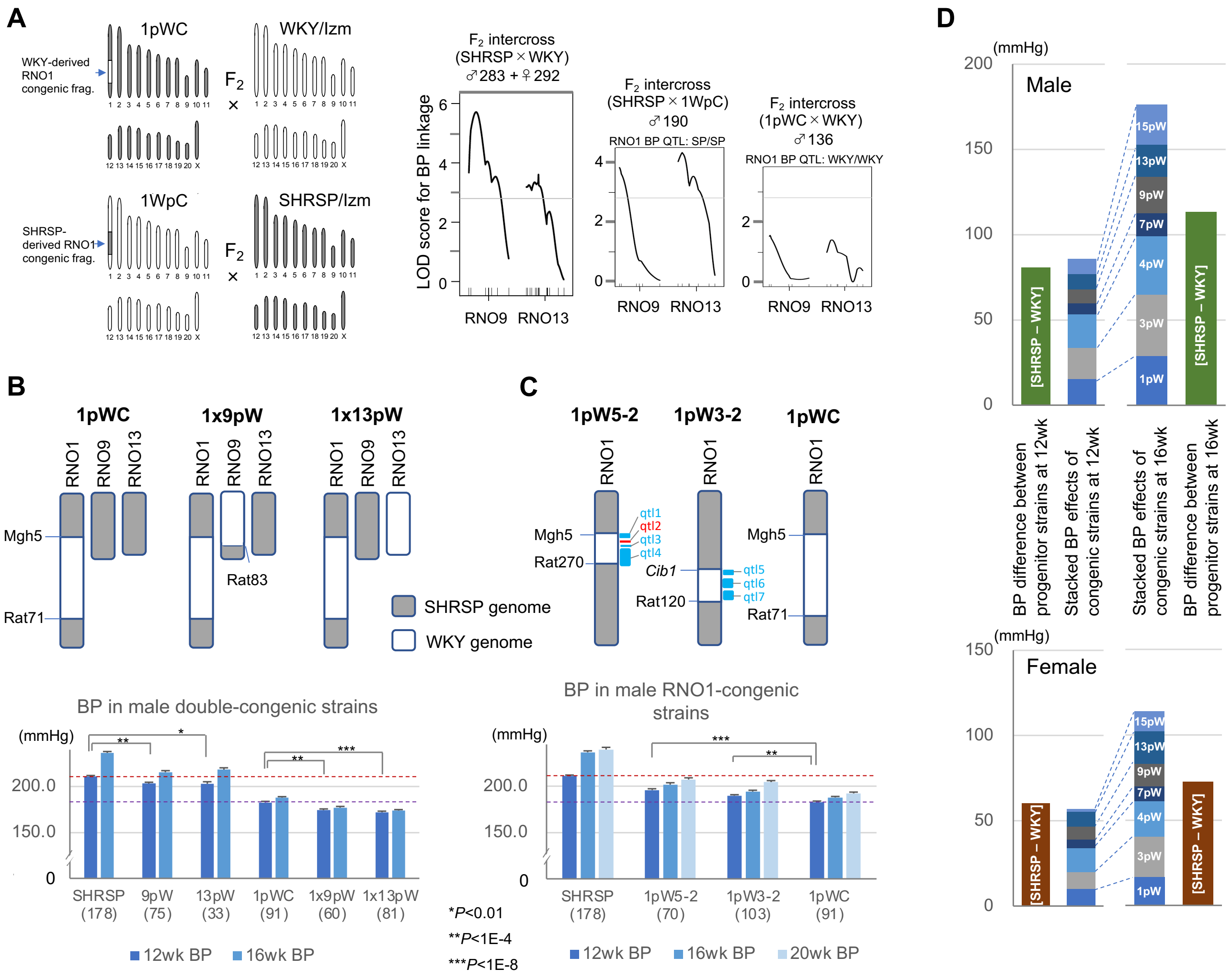

Fig. S3: Evaluation of potential epistatic interactions of BP QTLs. (A) Schematic explanation for the development of reciprocal F_2 intercrosses, which are produced between 1pWC and WKY and between SHRSP and 1WpC (see Methods). LOD score plots show potential epistatic interactions of BP QTLs between RNO1 and RNO9 and between RNO1 and RNO13. Comparison of BP measurements in two double congenic strains (1×9pW and 1×13pW) (**B**) and three RNO1-congenic strains (1pW5-2, 1pW3-2 and 1pWC) (**C**) with those in SHRSP/Izm indicates a diversity of composite effects of BP QTLs. See **Fig. S4A** about a panel of RNO1-congenic strains. **(D)** An aggregate of BP changes in 7 SHRSP-derived consomic strains is shown for males (in the upper panel) and females (in the lower panel).

Disease Models & Mechanisms • Supplementary information

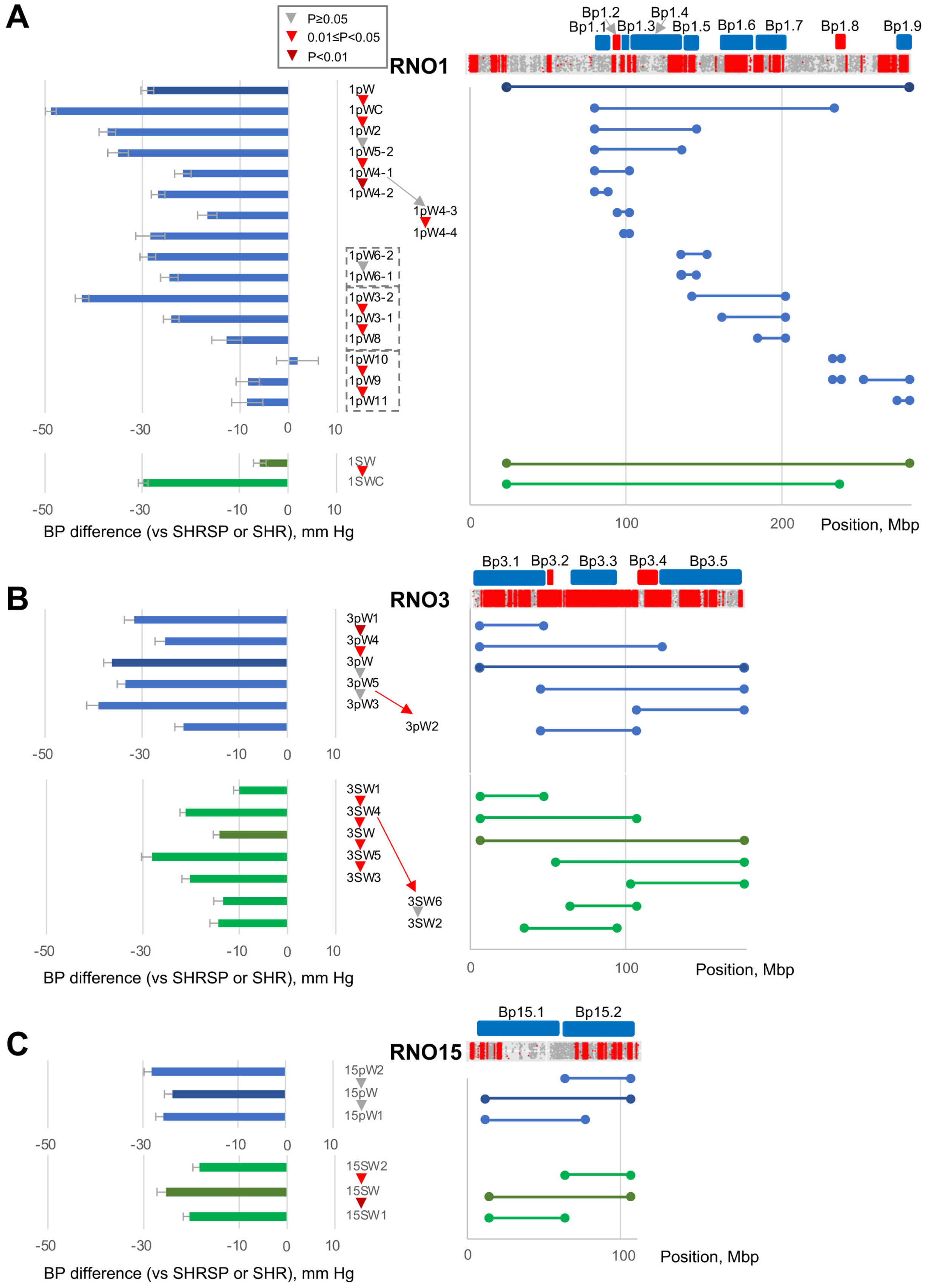

Fig. S4: Panels of congenic strains developed for three rat chromosomes. (A) For RNO1, a consomic (1pW) and 15 congenic strains (1pW*; the asterisk indicates a further description of congenic strains) are

Disease Models & Mechanisms • Supplementary information

constructed from SHRSP/Izm and WKY/Izm, while consomic (1SW) and congenic (1SWC) strains are constructed from SHR/Izm and WKY/Izm. BP differences from the recipient (ie, hypertensive in this case) progenitor strains are shown in the left part; the location of congenic fragments and the likely interval of BP QTLs are shown together with a map of inter-strain genetic diversity (refer to **Fig. 3A**) in the right part. Here, the direction of BP changes at each QTL is colored in blue (decrease) or red (increase), compared with the recipient progenitor strains. **(B)** For RNO3, a consomic (3pW) and 5 congenic (3pW*) strains are constructed from SHRSP/Izm and WKY/Izm, while a consomic (3SW) and 6 congenic (3SW*) strains are constructed from SHR/Izm and WKY/Izm. **(C)** For RNO15, a consomic (15pW) and 2 congenic (15pW1 and 15pW2) strains are constructed from SHRSP/Izm and WKY/Izm, while a consomic (15SW) and 6 congenic (15SW1 and 15SW2) strains are constructed from SHR/Izm and WKY/Izm.

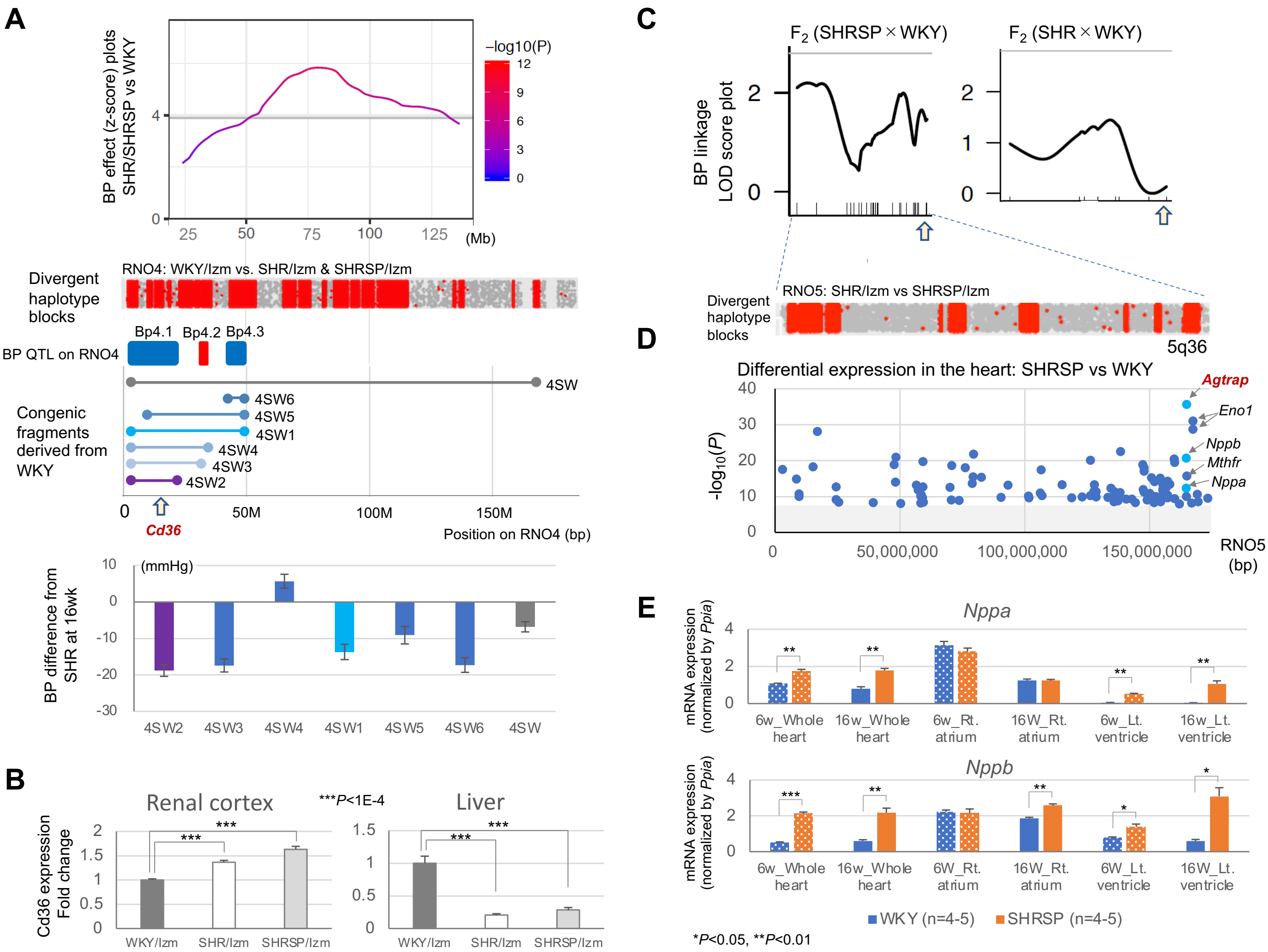

Fig. S5: BP QTLs with a complex genetic architecture on RNO4 and RNO5. (A) BP QTLs are detected in a region (3–49 Mb), at a distance from the linkage peak (81–87 Mb), in the upper panel: the location of congenic fragments and the likely interval of BP QTLs (Bp4.1 to 4.3) are shown together with a map of inter-strain genetic diversity (refer to **Fig. 3A**) in the middle panel; and BP differences from the recipient progenitor strain, SHR/Izm, are shown in the lower part. **(B)** Inter-strain differential mRNA expression of *Cd36*, located at Bp4.1, is significant (P<1×10^{-4}) in the kidney and liver (n=4 per strain), shown by fold change (SHR/Izm versus WKY/Izm). **(C)** A tendency of BP linkage is detectable in F_2 (SHRSP/Izm×WKY/Izm) but not in F_2 (SHR/Izm×WKY/Izm) by linkage plots, where the position of 5q36 is indicated by arrows. **(D)** Regional plots of differential gene expression on RNO5 in the heart between SHRSP/Izm and WKY/Izm. The dots indicate significant (P<1×10^{-8}) transcripts, with the names provided for some highly significant genes on 5q36. **(E)** Inter-strain differential gene expression of *Nppa* and *Nppb*, with each evaluated by qPCR for the whole heart, right atrium and left ventricle, longitudinally at 6 weeks and 16 weeks of age. *P<0.05, **P<0.01 by unpaired t test.

Disease Models & Mechanisms • Supplementary information

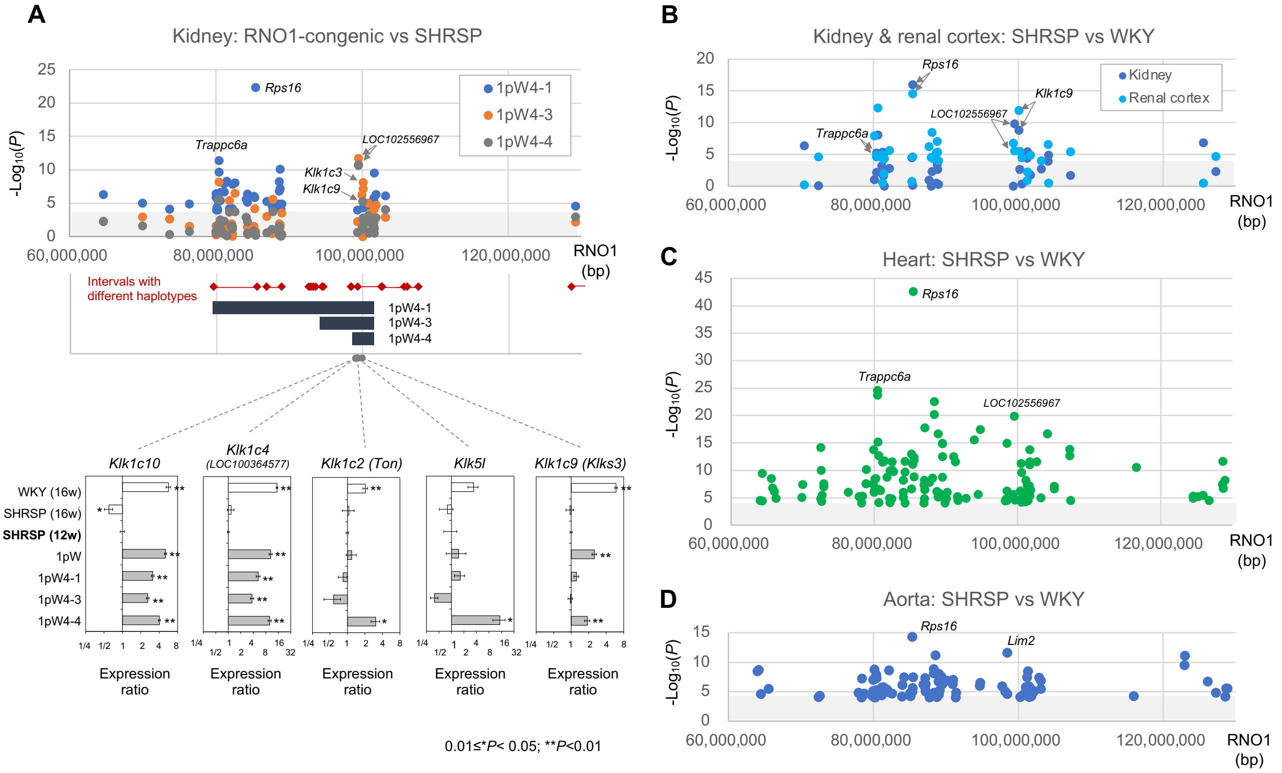

Fig. S6: Fine congenic mapping and gene expression profiling in the target region on RNO1. (A) Regional plots of differential gene expression in the kidney between SHRSP/Izm and each of three RNO1-congenic strains (1pW4-1, 1pW4-3 and 1pW4-4), in combination with fine congenic mapping and mRNA expression analysis of the *Klk1* paralogues. **P*<0.05, ***P*<0.01. Regional plots of differential gene expression between SHRSP/Izm and WKY/Izm are also shown for the kidney and renal cortex (**B**), heart (**C**) and aorta (**D**). The dots indicate modestly significant ($P<1\times10^{-4}$) transcripts, with the names provided for some highly significant genes. In panels, **A** and **B**, when a given transcript attains $P<1\times10^{-4}$ in comparison with any of 3 congenic strains (in **A**) or either of 2 tissues (in **B**), $-\log_{10}(P)$ values of that transcript are displayed for all strains or tissues (which may show $P\geq1\times10^{-4}$).

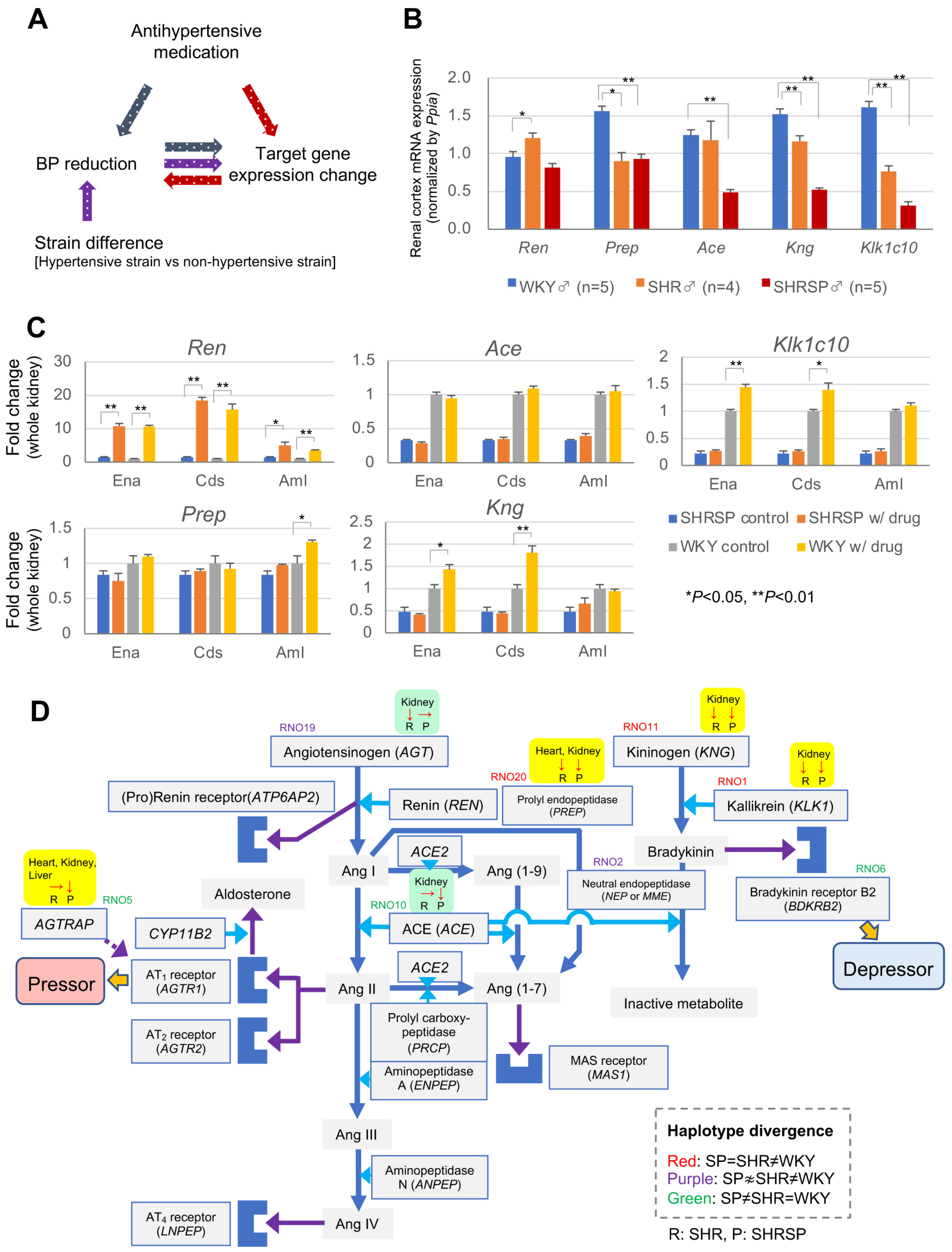

Fig. S7: Gene expression changes in target tissues of progenitor strains, induced by pharmacological intervention. (A) Cause and effect relationship of BP reduction. BP reduction can result from strain differences and antihypertensive medication, either of which will lead to gene expression changes in a BP-

Disease Models & Mechanisms • Supplementary information

dependent manner. On the other hand, some antihypertensive drugs may influence the expression of particular genes without changing systemic BP, ie, in a BP-independent manner via pharmacological activity, then causing BP reduction. **(B)** Inter-strain differential expression of 5 genes, which are components of the kallikrein-kinin and renin-angiotensin systems (KKS/RAS), in the renal cortex, evaluated by qPCR. **(C)** mRNA expression changes evaluated by qPCR for 5 KKS/RAS genes in the kidney of progenitor strains with and without a 4-week administration of enalapril (Ena), candesartan (Cds) and amlodipine (Aml), where mRNA expression level in WKY/Izm without drug administration is set to be 1. *P<0.05, **P<0.01. **(D)** A diagram of the KKS/RAS and the relationship of KKS/RAS gene expression changes identified in SHR and SHRSP; in the diagram, enalapril prevents an enzyme, angiotensin converting enzyme (ACE), from producing angiotensin II (Ang II) and inactivating bradykinin, while candesartan blocks the action of Ang II by binding to and inhibiting the AT_1 receptor.

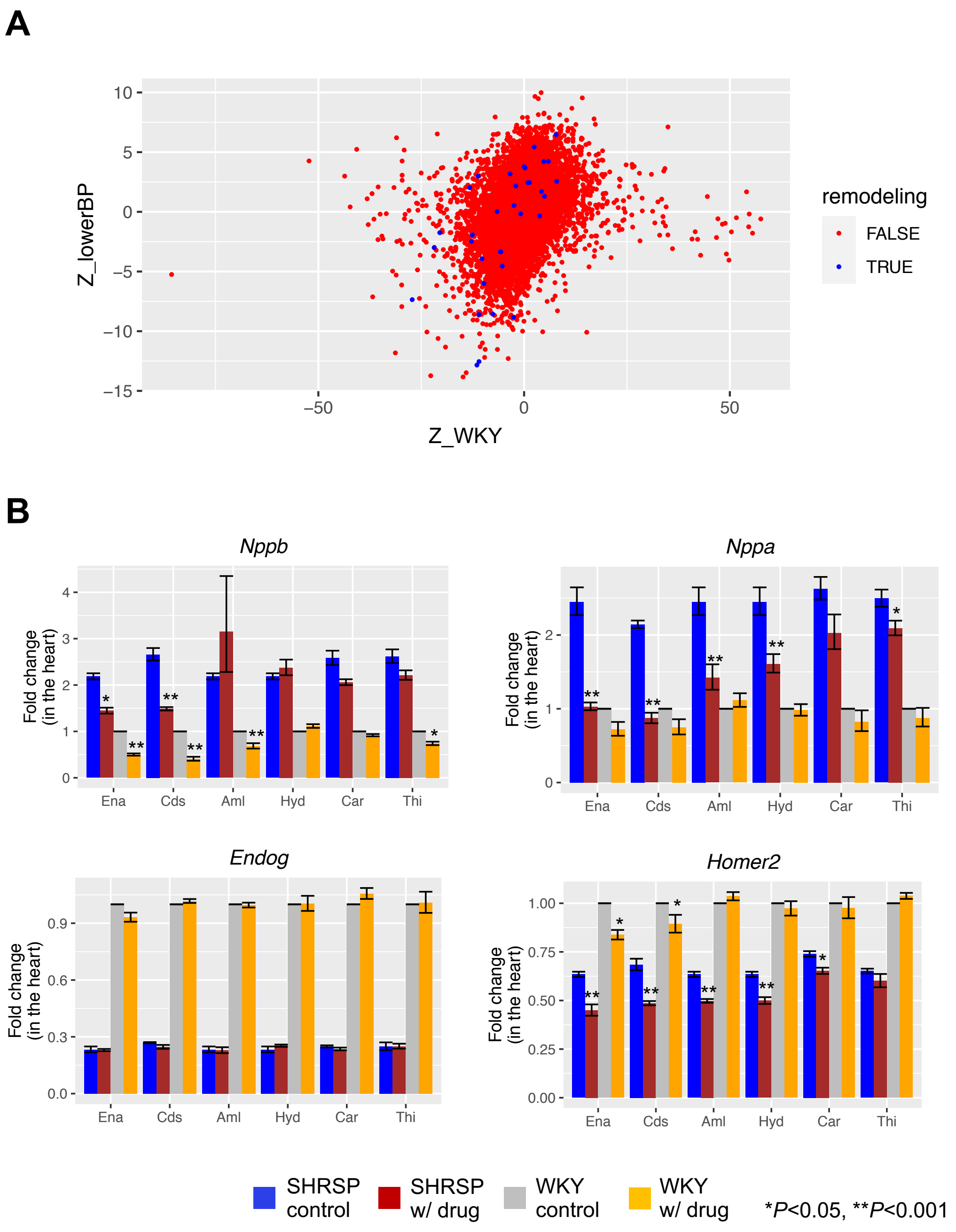

Fig. S8: Differential gene expression in the heart and drug specificity. (A) An overall fair correlation (*r*=0.325, *P*<2.2×10^{-16}) of differential gene expression between strain differences (non-hypertensive WKY/Izm versus hypertensive SHRSP/Izm, Z-score in *x*-axis) and antihypertensive medication (a group of rats treated with any of the four BP-decreasing drugs versus their control group, Z-score in *y*-axis) (refer to **Fig. 6**). WKY allele-associated effects tend to be positively correlated with BP lowering effects on mRNA expression differences; the correlation is pronounced for a group of genes (blue dots; the gene list is drawn from a study by Saucerman *et al.*, 2019, PMID: 30683889) that are involved in cardiac remodeling in response to alteration of mechanical cues (*r*=0.590). **(B)** mRNA expression changes of 4 candidate genes in the heart of progenitor strains with and without a 4-week administration of 6 antihypertensive drugs, where mRNA expression level in WKY/Izm without drug administration is set to be 1.

Disease Models & Mechanisms • Supplementary information

Table S1. Genome-wide haplotype structure of Wistar Kyoto colony-derived strains

No. of region	Chr	Start (Rnor_6.0)	End (Rnor_6.0)	No. of haplotype classes	LD clusters count	Ancestral haplotype segments [haplotype number for individual strain]	Wistar Kyoto colony-derived strains: SHRSP/Gla [2]	1pW9 (SHRSP-derived congenic) [10]	SHRSP/Izm [12]	SHR/OlaIpcv [1]	SHR/NCrlPrin [3]	SHR/NHsd [4]	SHR/OlaIpcvPrin [5]	SHR/Izm [11]	WKY/Gla [7]	WKY/Izm [13]	WKY/N [6]	WKY/NCrl [8]	WKY/NHsd [9]
1	1	558,853	2,371,874	2	1	0_0_0_0_0_0_0_0_0_0_0_0_1.793	1	1	1	1	1	1	1	1	1	2	1	1	1
2	1	2,371,875	2,782,592	1	0														
3	1	2,782,593	3,385,165	2	1	0_0_0_0_0_0_0_0_0_0_0_0_1.701	1	1	1	1	1	1	1	1	1	2	1	1	1
4	1	3,385,166	3,537,217	1	0														
5	1	3,537,218	5,492,362	2	1	0_0_0_0_0_1_0_0_1_0_0_0_1.212	1	1	1	1	1	1	1	1	1	2	2	1	2
6	1	5,492,363	5,737,252	1	0														
7	1	5,737,253	6,088,449	2	1	0_0_0_0_0_1_0_0_1_0_0_0_1.500	1	1	1	1	1	1	1	1	1	2	2	1	2
8	1	6,088,450	13,700,488	1	0														
9	1	13,700,489	15,158,018	2	1	1_1_1_1_1_1_1_0_1_1_1_1_1.150	2	2	2	2	2	2	2	2	2	2	2	1	2
10	1	15,158,019	15,160,758	1	0														
11	1	15,160,759	17,422,542	2	1	1_1_1_1_1_0_1_0_0_1_1_1_0.334	2	2	2	2	2	2	2	2	2	1	1	1	1
12	1	17,422,543	17,423,064	1	0														
13	1	17,423,065	17,473,223	2	1	1_1_1_1_1_1_1_0_1_1_1_1_1.86	2	2	2	2	2	2	2	2	2	2	2	1	2
14	1	17,473,224	17,794,944	1	0														
15	1	17,794,945	19,128,873	2	1	0_0_0_0_0_0_0_1_0_0_0_0_1.631	1	1	1	1	1	1	1	1	1	2	1	2	1
16	1	19,128,874	19,332,898	1	0														
17	1	19,332,899	19,779,021	2	1	0_1_0_0_0_1_0_0_1_1_0_1_0.530	2	2	2	1	1	1	1	1	1	1	2	1	2
18	1	19,779,022	19,779,141	3	2	0_1_0_0_0_1_0_0_1_1_0_1_0.530	3	3	3	2	2	2	2	2	2	1	3	1	3
19	1	19,779,142	20,001,852	3	3	0_0_0_0_0_0_0_1_0_0_0_0_1.94 (	2	2	2	1	1	1	1	1	1	3	2	3	2
20	1	20,001,853	20,008,191	4	4	0_0_0_0_0_0_0_1_0_0_0_0_1.94 (	2	2	2	1	1	1	1	1	1	4	2	3	2
21	1	20,008,192	20,013,655	4	3	0_1_0_0_0_1_0_0_1_1_0_1_0.530	4	4	4	3	3	3	3	3	3	2	4	1	4
22	1	20,013,656	20,020,072	3	2	1_0_1_1_1_0_1_0_0_0_1_0_0.224	2	2	2	3	3	3	3	3	3	2	2	1	2
23	1	20,020,073	20,041,244	2	1	1_1_1_1_1_1_1_0_1_1_1_1_1.177	2	2	2	2	2	2	2	2	2	2	2	1	2
24	1	20,041,245	20,043,042	1	0														
25	1	20,043,043	21,529,918	2	1	0_1_0_0_0_1_0_0_1_1_0_1_0.131	2	2	2	1	1	1	1	1	1	1	2	1	2
26	1	21,529,919	21,917,575	1	0														
27	1	21,917,576	21,921,040	2	1	0_0_0_0_0_0_0_1_0_0_0_0_1.512	1	1	1	1	1	1	1	1	1	2	1	2	1
28	1	21,921,041	23,567,341	3	2	0_0_0_0_0_0_0_1_0_0_0_0_1.512	2	2	2	2	2	2	2	2	2	3	1	3	1
29	1	23,567,342	24,270,697	2	1	0_0_0_0_0_0_0_1_0_0_0_0_1.512	1	1	1	1	1	1	1	1	1	2	1	2	1
30	1	24,270,698	25,813,594	1	0														
31	1	25,813,595	27,430,847	2	1	1_1_1_1_1_0_1_1_0_1_1_1_1.419	2	2	2	2	2	2	2	2	2	2	1	2	1
32	1	27,430,848	27,432,118	1	0														
33	1	27,432,119	28,365,567	2	1	1_1_1_1_1_0_1_0_0_1_1_1_1.904	2	2	2	2	2	2	2	2	2	2	1	1	1
34	1	28,365,568	28,370,790	1	0														
35	1	28,370,791	32,065,780	2	1	1_1_1_1_1_0_1_1_0_1_1_1_1.275	2	2	2	2	2	2	2	2	2	2	1	2	1
36	1	32,065,781	34,752,986	1	0														
37	1	34,752,987	41,194,064	2	1	0_1_0_0_0_0_0_0_0_1_0_1_0.282	2	2	2	1	1	1	1	1	1	1	1	1	1
38	1	41,194,065	50,364,378	1	0														
39	1	50,364,379	51,093,314	2	1	1_0_1_1_1_1_1_0_1_1_1_1_1.104	1	2	2	2	2	2	2	2	2	2	2	1	2
40	1	51,093,315	51,096,766	1	0														
41	1	51,096,767	51,856,782	2	1	1_0_1_1_1_1_1_0_1_1_1_1_0.803	1	2	2	2	2	2	2	2	2	1	2	1	2
42	1	51,856,783	52,028,646	1	0														
43	1	52,028,647	52,889,397	2	1	1_0_1_1_1_1_1_0_1_1_1_1_0.115	1	2	2	2	2	2	2	2	2	1	2	1	2
44	1	52,889,398	52,919,964	1	0														
45	1	52,919,965	53,268,709	2	1	1_0_1_1_1_1_1_1_1_1_1_1_0.244	1	2	2	2	2	2	2	2	2	1	2	2	2
46	1	53,268,710	53,282,158	1	0														
47	1	53,282,159	57,362,320	2	1	1_0_1_1_1_1_1_1_1_1_1_1_1.111	1	2	2	2	2	2	2	2	2	2	2	2	2
48	1	57,362,321	58,653,132	1	0														
49	1	58,653,133	61,220,168	2	1	1_0_1_1_1_1_1_1_1_1_1_1_1.976	1	2	2	2	2	2	2	2	2	2	2	2	2
50	1	61,220,169	61,344,766	1	0														
51	1	61,344,767	61,465,598	2	1	1_0_1_1_1_1_1_1_1_1_1_1_1.36	1	2	2	2	2	2	2	2	2	2	2	2	2
52	1	61,465,599	61,670,453	1	0														
53	1	61,670,454	63,132,940	2	1	1_0_1_1_1_1_1_1_1_1_1_1_1.960	1	2	2	2	2	2	2	2	2	2	2	2	2
54	1	63,132,941	63,166,736	1	0														
55	1	63,166,737	64,648,925	2	1	0_0_0_0_0_1_1_1_1_1_0_1_1.784	1	2	2	1	1	1	1	1	2	2	2	2	2
56	1	64,648,926	64,705,694	1	0														
57	1	64,705,695	65,498,384	2	1	1_0_1_1_1_1_1_1_1_1_1_1_1.85	1	2	2	2	2	2	2	2	2	2	2	2	2
58	1	65,498,385	67,709,543	1	0														
59	1	67,709,544	75,061,343	2	1	0_0_0_0_0_1_1_1_1_1_0_1_1.314	1	2	2	1	1	1	1	1	2	2	2	2	2
60	1	75,061,344	79,608,513	1	0														
61	1	79,608,514	85,545,574	2	1	0_1_0_0_0_0_0_0_0_1_0_1_0.437	2	2	2	1	1	1	1	1	1	1	1	1	1
62	1	85,545,575	85,588,908	1	0														
63	1	85,588,909	85,976,721	2	1	1_1_1_1_1_0_1_1_0_1_1_1_1.695	2	2	2	2	2	2	2	2	2	2	1	2	1
64	1	85,976,722	85,984,892	1	0														
65	1	85,984,893	86,825,798	2	1	1_0_1_1_1_1_1_1_1_1_1_1_1.505	1	2	2	2	2	2	2	2	2	2	2	2	2
66	1	86,825,799	86,835,763	1	0														
67	1	86,835,764	88,914,994	2	1	0_1_0_0_0_0_0_0_0_1_0_1_0.104	2	2	2	1	1	1	1	1	1	1	1	1	1
68	1	88,914,995	92,615,962	1	0														
69	1	92,615,963	92,917,237	2	1	0_0_0_0_0_0_0_0_0_0_0_0_1.318	1	1	1	1	1	1	1	1	1	2	1	1	1
70	1	92,917,238	93,229,460	1	0														
71	1	93,229,461	93,573,019	2	1	0_0_0_0_0_0_0_0_0_0_0_0_1.290	1	1	1	1	1	1	1	1	1	2	1	1	1
72	1	93,573,020	93,856,420	1	0														
73	1	93,856,421	94,462,831	2	1	1_1_1_1_1_0_0_0_0_1_1_1_1.452	2	2	2	2	2	2	2	2	1	2	1	1	1
74	1	94,462,832	94,508,155	3	2	1_1_1_1_1_0_0_0_0_1_1_1_0.171	3	3	3	3	3	3	3	3	1	2	1	1	1
75	1	94,508,156	94,636,774	2	1	1_1_1_1_1_0_0_0_0_1_1_1_0.171	2	2	2	2	2	2	2	2	1	1	1	1	1
76	1	94,636,775	98,360,380	1	0														
77	1	98,360,381	98,426,698	2	1	1_1_1_1_1_0_1_0_0_1_1_1_0.87	2	2	2	2	2	2	2	2	2	1	1	1	1
78	1	98,426,699	99,315,979	1	0														

79	1	99,315,980	102,566,923	2	1	1_1_1_1_1_0_1_0_0_1_1_1_0.512	2	2	2	2	2	2	2	2	2	1	1	1	1
80	1	102,566,924	102,690,159	1	0														
81	1	102,690,160	105,670,912	2	1	0_1_0_0_0_0_0_0_0_1_1_1_0.221	2	2	2	1	1	1	1	2	1	1	1	1	1
82	1	105,670,913	106,087,660	1	0														
83	1	106,087,661	107,612,574	2	1	0_1_0_0_0_0_0_0_0_1_1_1_0.138	2	2	2	1	1	1	1	2	1	1	1	1	1
84	1	107,612,575	124,287,025	1	0														
85	1	124,287,026	125,499,215	2	1	1_1_1_1_1_0_1_0_0_1_1_1_1.648	2	2	2	2	2	2	2	2	2	2	1	1	1
86	1	125,499,216	128,601,412	1	0														
87	1	128,601,413	132,276,744	2	1	1_1_1_1_1_0_1_0_0_1_1_1_0.307	2	2	2	2	2	2	2	2	2	1	1	1	1
88	1	132,276,745	132,369,454	1	0														
89	1	132,369,455	137,606,926	2	1	1_1_1_1_1_0_1_0_0_1_1_1_0.498	2	2	2	2	2	2	2	2	2	1	1	1	1
90	1	137,606,927	138,765,018	1	0														
91	1	138,765,019	139,067,793	2	1	1_1_1_1_1_0_1_0_0_1_1_1_0.382	2	2	2	2	2	2	2	2	2	1	1	1	1
92	1	139,067,794	139,262,851	1	0														
93	1	139,262,852	140,123,614	2	1	1_1_1_1_1_0_1_0_0_1_1_1_0.911	2	2	2	2	2	2	2	2	2	1	1	1	1
94	1	140,123,615	141,966,315	1	0														
95	1	141,966,316	143,583,679	2	1	1_1_1_1_1_0_1_0_0_1_1_1_0.127	2	2	2	2	2	2	2	2	2	1	1	1	1
96	1	143,583,680	143,950,813	1	0														
97	1	143,950,814	145,513,870	2	1	1_1_1_1_1_0_1_0_0_1_1_1_0.103	2	2	2	2	2	2	2	2	2	1	1	1	1
98	1	145,513,871	151,472,952	1	0														
99	1	151,472,953	151,779,500	2	1	1_0_1_1_1_0_1_0_0_1_1_1_1.205	1	2	2	2	2	2	2	2	2	2	1	1	1
100	1	151,779,501	154,682,506	1	0														
101	1	154,682,507	155,738,749	2	1	1_0_1_1_1_0_1_0_0_1_1_1_1.738	1	2	2	2	2	2	2	2	2	2	1	1	1
102	1	155,738,750	155,763,512	3	2	1_0_1_1_1_0_1_0_0_1_1_1_1.738	2	3	3	3	3	3	3	3	3	3	1	1	1
103	1	155,763,513	156,443,285	2	1	1_0_1_1_1_0_1_0_0_1_1_1_1.738	1	2	2	2	2	2	2	2	2	2	1	1	1
104	1	156,443,286	156,509,733	1	0														
105	1	156,509,734	156,638,901	2	1	1_1_1_1_1_0_1_0_0_1_1_1_1.74	2	2	2	2	2	2	2	2	2	2	1	1	1
106	1	156,638,902	159,028,968	1	0														
107	1	159,028,969	159,029,403	2	1	1_0_1_1_1_0_0_0_0_0_0_0_1.23	1	1	1	2	2	2	2	1	1	2	1	1	1
108	1	159,029,404	159,157,006	3	2	0_0_0_0_0_0_0_0_0_0_0_0_1.27	1	1	1	2	2	2	2	1	1	3	1	1	1
109	1	159,157,007	159,157,695	2	1	1_0_1_1_1_0_0_0_0_0_0_0_1.23	1	1	1	2	2	2	2	1	1	2	1	1	1
110	1	159,157,696	165,214,654	1	0														
111	1	165,214,655	168,469,070	2	1	1_1_1_1_1_0_1_0_0_1_1_1_0.301	2	2	2	2	2	2	2	2	2	1	1	1	1
112	1	168,469,071	169,078,850	1	0														
113	1	169,078,851	170,041,068	2	1	1_1_1_1_1_0_1_0_0_1_1_1_0.797	2	2	2	2	2	2	2	2	2	1	1	1	1
114	1	170,041,069	170,194,497	1	0														
115	1	170,194,498	176,044,710	2	1	1_1_1_1_1_0_1_0_0_1_1_1_0.394	2	2	2	2	2	2	2	2	2	1	1	1	1
116	1	176,044,711	176,063,475	1	0														
117	1	176,063,476	177,038,679	2	1	1_1_1_1_1_0_1_0_0_1_1_1_0.107	2	2	2	2	2	2	2	2	2	1	1	1	1
118	1	177,038,680	177,105,368	1	0														
119	1	177,105,369	177,395,264	2	1	1_1_1_1_1_0_1_0_0_1_1_1_0.299	2	2	2	2	2	2	2	2	2	1	1	1	1
120	1	177,395,265	177,511,936	1	0														
121	1	177,511,937	184,474,684	2	1	1_1_1_1_1_0_1_0_0_1_1_1_0.193	2	2	2	2	2	2	2	2	2	1	1	1	1
122	1	184,474,685	184,475,528	1	0														
123	1	184,475,529	184,993,034	2	1	1_1_1_1_1_0_1_1_0_1_1_1_1.404	2	2	2	2	2	2	2	2	2	2	1	2	1
124	1	184,993,035	184,993,652	1	0														
125	1	184,993,653	187,584,314	2	1	0_0_0_0_0_0_0_1_0_0_0_0_1.267	1	1	1	1	1	1	1	1	1	2	1	2	1
126	1	187,584,315	188,176,103	1	0														
127	1	188,176,104	189,081,346	2	1	0_0_0_0_0_0_0_1_0_0_0_0_1.738	1	1	1	1	1	1	1	1	1	2	1	2	1
128	1	189,081,347	189,082,713	1	0														
129	1	189,082,714	189,296,104	2	1	0_0_0_0_0_0_0_0_0_0_0_0_1.360	1	1	1	1	1	1	1	1	1	2	1	1	1
130	1	189,296,105	189,297,690	1	0														
131	1	189,297,691	189,388,700	2	1	1_1_1_1_1_0_1_0_0_1_1_1_1.25	2	2	2	2	2	2	2	2	2	2	1	1	1
132	1	189,388,701	189,445,639	1	0														
133	1	189,445,640	193,635,180	2	1	1_1_1_1_1_0_1_0_0_1_1_1_1.209	2	2	2	2	2	2	2	2	2	2	1	1	1
134	1	193,635,181	193,699,572	1	0														
135	1	193,699,573	194,075,168	2	1	1_1_1_1_1_0_1_0_0_1_1_1_0.449	2	2	2	2	2	2	2	2	2	1	1	1	1
136	1	194,075,169	194,076,882	1	0														
137	1	194,076,883	194,580,835	2	1	1_1_1_1_1_0_1_0_0_1_1_1_1.362	2	2	2	2	2	2	2	2	2	2	1	1	1
138	1	194,580,836	195,970,719	1	0														
139	1	195,970,720	197,993,994	2	1	1_1_1_1_1_0_1_0_0_1_1_1_0.224	2	2	2	2	2	2	2	2	2	1	1	1	1
140	1	197,993,995	197,999,055	1	0														
141	1	197,999,056	200,356,425	2	1	0_1_0_0_0_0_0_0_0_1_1_1_0.148	2	2	2	1	1	1	1	2	1	1	1	1	1
142	1	200,356,426	200,377,883	1	0														
143	1	200,377,884	201,929,971	2	1	0_0_0_0_0_0_0_0_0_0_1_0_0.102	1	1	1	1	1	1	1	2	1	1	1	1	1
144	1	201,929,972	202,033,374	1	0														
145	1	202,033,375	202,455,616	2	1	0_1_0_0_0_0_0_0_0_1_1_1_0.233	2	2	2	1	1	1	1	2	1	1	1	1	1
146	1	202,455,617	202,508,332	1	0														
147	1	202,508,333	205,418,671	2	1	0_0_0_0_0_0_0_0_0_0_1_0_0.130	1	1	1	1	1	1	1	2	1	1	1	1	1
148	1	205,418,672	206,474,727	1	0														
149	1	206,474,728	214,640,486	2	1	0_0_0_0_0_0_0_0_0_0_1_0_0.704	1	1	1	1	1	1	1	2	1	1	1	1	1
150	1	214,640,487	214,712,569	1	0														
151	1	214,712,570	218,155,530	2	1	0_0_0_0_0_0_0_0_0_0_1_0_0.289	1	1	1	1	1	1	1	2	1	1	1	1	1
152	1	218,155,531	218,390,296	1	0														
153	1	218,390,297	223,016,428	2	1	0_0_0_0_0_0_0_0_0_0_1_0_0.330	1	1	1	1	1	1	1	2	1	1	1	1	1
154	1	223,016,429	223,488,703	1	0														
155	1	223,488,704	226,821,144	2	1	0_0_0_0_0_0_0_0_0_0_1_0_0.196	1	1	1	1	1	1	1	2	1	1	1	1	1
156	1	226,821,145	226,822,470	1	0														
157	1	226,822,471	231,420,049	2	1	1_1_1_1_1_0_1_0_0_1_0_1_0.369	2	2	2	2	2	2	2	1	2	1	1	1	1
158	1	231,420,050	231,671,705	1	0														
159	1	231,671,706	232,127,607	2	1	0_0_0_0_0_1_0_1_1_1_1_0_1.198	1	2	1	1	1	1	1	2	1	2	2	2	2
160	1	232,127,608	232,299,761	1	0														
161	1	232,299,762	233,668,478	2	1	0_0_0_0_0_1_0_1_1_1_1_0_1.105	1	2	1	1	1	1	1	2	1	2	2	2	2
162	1	233,668,479	234,383,542	1	0														
163	1	234,383,543	234,623,867	2	1	0_1_0_0_0_1_0_1_1_1_1_1_1.121	2	2	2	1	1	1	1	2	1	2	2	2	2

164	1	234,623,868	235,166,011	1	0														
165	1	235,166,012	237,064,171	2	1	0_0_0_0_0_1_0_1_1_1_1_0_1.787	1	2	1	1	1	1	1	2	1	2	2	2	2
166	1	237,064,172	237,131,451	1	0														
167	1	237,131,452	237,249,016	2	1	0_1_0_0_0_1_1_1_1_1_1_1_1.75	2	2	2	1	1	1	1	2	2	2	2	2	2
168	1	237,249,017	237,329,473	1	0														
169	1	237,329,474	239,554,071	2	1	0_1_0_0_0_1_1_1_1_1_1_1_1.191	2	2	2	1	1	1	1	2	2	2	2	2	2
170	1	239,554,072	239,991,195	1	0														
171	1	239,991,196	242,560,458	2	1	0_1_0_0_0_1_1_1_1_1_1_1_1.172	2	2	2	1	1	1	1	2	2	2	2	2	2
172	1	242,560,459	242,801,115	1	0														
173	1	242,801,116	243,167,325	2	1	1_1_1_1_1_0_0_0_0_1_1_1_0.483	2	2	2	2	2	2	2	2	1	1	1	1	1
174	1	243,167,326	243,184,021	1	0														
175	1	243,184,022	243,760,638	2	1	1_1_1_1_1_0_0_0_0_1_1_1_1.506	2	2	2	2	2	2	2	2	1	2	1	1	1
176	1	243,760,639	244,163,036	1	0														
177	1	244,163,037	244,457,512	2	1	0_1_0_0_0_0_0_0_0_1_1_1_1.96	2	2	2	1	1	1	1	2	1	2	1	1	1
178	1	244,457,513	245,029,329	1	0														
179	1	245,029,330	246,315,485	2	1	1_1_1_1_1_0_0_0_0_1_1_1_1.629	2	2	2	2	2	2	2	2	1	2	1	1	1
180	1	246,315,486	246,473,972	3	2	0_1_0_0_0_1_1_1_1_1_1_1_1.88	3	3	3	1	1	1	1	3	2	3	2	2	2
181	1	246,473,973	246,561,491	3	3	0_1_0_0_0_0_0_0_0_1_1_1_1.149	3	3	3	1	1	1	1	3	2	3	2	2	2
182	1	246,561,492	246,685,787	3	2	0_1_0_0_0_0_0_0_0_1_1_1_1.149	3	3	3	1	1	1	1	3	2	3	2	2	2
183	1	246,685,788	246,718,115	3	3	0_1_0_0_0_0_0_0_0_1_1_1_1.149	3	3	3	1	1	1	1	3	2	3	2	2	2
184	1	246,718,116	246,766,282	3	2	0_1_0_0_0_0_0_0_0_1_1_1_1.149	3	3	3	2	2	2	2	3	1	3	1	1	1
185	1	246,766,283	247,046,613	2	1	0_1_0_0_0_0_0_0_0_1_1_1_1.149	2	2	2	1	1	1	1	2	1	2	1	1	1
186	1	247,046,614	247,201,246	3	2	0_1_0_0_0_0_0_0_0_1_1_1_1.149	3	3	3	1	1	1	1	3	2	3	2	2	2
187	1	247,201,247	247,682,381	3	3	0_1_0_0_0_0_0_0_0_1_1_1_1.149	3	3	3	1	1	1	1	3	2	3	2	2	2
188	1	247,682,382	247,860,558	3	2	0_1_0_0_0_0_0_0_0_1_1_1_1.149	3	3	3	2	2	2	2	3	1	3	1	1	1
189	1	247,860,559	248,492,647	3	3	0_1_0_0_0_0_0_0_0_1_1_1_1.149	3	3	3	1	1	1	1	3	2	3	2	2	2
190	1	248,492,648	248,578,702	3	2	0_1_0_0_0_0_0_0_0_1_1_1_1.149	3	3	3	2	2	2	2	3	1	3	1	1	1
191	1	248,578,703	249,010,881	3	3	0_1_0_0_0_0_0_0_0_1_1_1_1.149	3	3	3	1	1	1	1	3	2	3	2	2	2
192	1	249,010,882	249,103,697	3	2	0_1_0_0_0_0_0_0_0_1_1_1_1.149	3	3	3	1	1	1	1	3	2	3	2	2	2
193	1	249,103,698	249,242,672	2	1	0_1_0_0_0_0_0_0_0_1_1_1_1.149	2	2	2	1	1	1	1	2	1	2	1	1	1
194	1	249,242,673	249,312,001	3	2	0_1_0_0_0_0_0_0_0_1_1_1_1.149	3	3	3	2	2	2	2	3	1	3	1	1	1
195	1	249,312,002	249,424,008	3	3	0_1_0_0_0_0_0_0_0_1_1_1_1.149	3	3	3	1	1	1	1	3	2	3	2	2	2
196	1	249,424,009	249,481,504	3	2	0_1_0_0_0_0_0_0_0_1_1_1_1.149	3	3	3	1	1	1	1	3	2	3	2	2	2
197	1	249,481,505	249,497,854	2	1	0_1_0_0_0_0_0_0_0_1_1_1_1.149	2	2	2	1	1	1	1	2	1	2	1	1	1
198	1	249,497,855	249,756,555	3	2	0_1_0_0_0_0_0_0_0_1_1_1_1.149	3	3	3	2	2	2	2	3	1	3	1	1	1
199	1	249,756,556	250,557,116	3	3	0_1_0_0_0_0_0_0_0_1_1_1_1.149	3	3	3	1	1	1	1	3	2	3	2	2	2
200	1	250,557,117	250,799,822	3	2	0_1_0_0_0_0_0_0_0_1_1_1_1.149	3	3	3	1	1	1	1	3	2	3	2	2	2
201	1	250,799,823	251,841,366	2	1	0_1_0_0_0_1_1_1_1_1_1_1_1.962	2	2	2	1	1	1	1	2	2	2	2	2	2
202	1	251,841,367	252,003,333	1	0														
203	1	252,003,334	252,548,679	2	1	0_1_0_0_0_1_1_1_1_1_1_1_1.186	2	2	2	1	1	1	1	2	2	2	2	2	2
204	1	252,548,680	252,553,355	1	0														
205	1	252,553,356	252,574,886	2	1	1_0_1_1_1_1_1_1_1_1_0_0_1.133	1	2	1	2	2	2	2	1	2	2	2	2	2
206	1	252,574,887	252,742,621	3	2	1_0_1_1_1_1_1_1_1_1_0_0_1.133	1	2	1	3	3	3	3	1	2	2	2	2	2
207	1	252,742,622	252,743,026	2	1	1_1_1_1_1_0_0_0_0_0_1_1_0.69	2	1	2	2	2	2	2	2	1	1	1	1	1
208	1	252,743,027	254,158,290	1	0														
209	1	254,158,291	259,772,552	2	1	0_1_0_0_0_1_1_1_1_1_1_1_1.643	2	2	2	1	1	1	1	2	2	2	2	2	2
210	1	259,772,553	263,506,843	1	0														
211	1	263,506,844	264,567,350	2	1	1_1_1_1_1_0_0_0_0_0_1_1_0.941	2	1	2	2	2	2	2	2	1	1	1	1	1
212	1	264,567,351	264,628,646	1	0														
213	1	264,628,647	264,746,331	2	1	1_1_1_1_1_0_0_0_0_1_1_1_1.110	2	2	2	2	2	2	2	2	1	2	1	1	1
214	1	264,746,332	264,747,483	1	0														
215	1	264,747,484	270,770,675	2	1	1_1_1_1_1_0_0_0_0_0_1_1_0.574	2	1	2	2	2	2	2	2	1	1	1	1	1
216	1	270,770,676	272,358,544	1	0														
217	1	272,358,545	274,840,613	2	1	1_1_1_1_1_0_0_0_0_0_1_1_0.333	2	1	2	2	2	2	2	2	1	1	1	1	1
218	1	274,840,614	277,152,454	1	0														
219	1	277,152,455	280,384,730	2	1	1_1_1_1_1_0_0_0_0_0_1_1_0.377	2	1	2	2	2	2	2	2	1	1	1	1	1
220	1	280,384,731	280,527,679	1	0														
221	1	280,527,680	282,528,505	2	1	1_1_1_1_1_0_0_0_0_0_1_1_0.122	2	1	2	2	2	2	2	2	1	1	1	1	1
222	2	358,368	385,376	2	1	1_1_1_1_1_0_0_0_0_1_1_1_0.79	2	2	2	2	2	2	2	2	1	1	1	1	1
223	2	385,377	4,799,428	1	0														
224	2	4,799,429	4,858,314	2	1	1_1_1_1_1_0_1_0_0_1_1_1_0.39	2	2	2	2	2	2	2	2	2	1	1	1	1
225	2	4,858,315	6,478,470	1	0														
226	2	6,478,471	9,110,037	2	1	1_1_1_1_1_0_0_0_0_1_1_1_0.143	2	2	2	2	2	2	2	2	1	1	1	1	1
227	2	9,110,038	9,155,060	1	0														
228	2	9,155,061	11,935,146	2	1	1_1_1_1_1_0_0_0_0_1_1_1_0.244	2	2	2	2	2	2	2	2	1	1	1	1	1
229	2	11,935,147	11,937,736	1	0														
230	2	11,937,737	13,716,187	2	1	1_1_1_1_1_0_0_1_0_1_1_1_0.224	2	2	2	2	2	2	2	2	1	1	1	2	1
231	2	13,716,188	13,739,278	3	2	1_1_1_1_1_0_0_1_0_1_1_1_0.224	3	3	3	3	3	3	3	3	1	1	1	2	1
232	2	13,739,279	13,853,036	3	3	1_1_1_1_1_0_0_0_0_1_1_1_0.81	3	3	3	3	3	3	3	3	1	1	1	2	1
233	2	13,853,037	14,000,652	3	2	1_1_1_1_1_0_0_0_0_1_1_1_0.81	3	3	3	3	3	3	3	3	1	1	1	2	1
234	2	14,000,653	14,150,213	2	1	1_1_1_1_1_0_0_1_0_1_1_1_0.224	2	2	2	2	2	2	2	2	1	1	1	2	1
235	2	14,150,214	14,717,801	3	2	1_1_1_1_1_0_0_0_0_1_1_1_0.143	3	3	3	3	3	3	3	3	1	1	1	2	1
236	2	14,717,802	14,742,242	2	1	1_1_1_1_1_0_0_0_0_1_1_1_0.143	2	2	2	2	2	2	2	2	1	1	1	1	1
237	2	14,742,243	15,657,997	3	2	1_1_1_1_1_0_0_0_0_1_1_1_0.143	3	3	3	3	3	3	3	3	1	1	1	2	1
238	2	15,657,998	15,871,627	2	1	1_1_1_1_1_0_0_1_0_1_1_1_0.132	2	2	2	2	2	2	2	2	1	1	1	2	1
239	2	15,871,628	16,320,890	3	2	1_1_1_1_1_0_0_0_0_1_1_1_0.209	3	3	3	3	3	3	3	3	1	1	1	2	1
240	2	16,320,891	16,773,477	2	1	1_1_1_1_1_0_0_0_0_1_1_1_0.209	2	2	2	2	2	2	2	2	1	1	1	1	1
241	2	16,773,478	17,306,244	3	2	1_1_1_1_1_0_0_0_0_1_1_1_0.209	3	3	3	3	3	3	3	3	1	1	1	2	1
242	2	17,306,245	18,062,317	2	1	1_1_1_1_1_0_0_0_0_1_1_1_0.209	2	2	2	2	2	2	2	2	1	1	1	1	1
243	2	18,062,318	18,177,527	1	0														
244	2	18,177,528	19,291,055	2	1	1_1_1_1_1_0_0_0_0_1_1_1_0.137	2	2	2	2	2	2	2	2	1	1	1	1	1
245	2	19,291,056	19,368,601	1	0														
246	2	19,368,602	23,533,759	2	1	1_1_1_1_1_0_0_0_0_1_1_1_0.412	2	2	2	2	2	2	2	2	1	1	1	1	1
247	2	23,533,760	23,622,931	1	0														
248	2	23,622,932	23,850,269	2	1	1_1_1_1_1_0_0_0_0_1_1_1_0.186	2	2	2	2	2	2	2	2	1	1	1	1	1

249	2	23,850,270	24,292,620	1	0													
250	2	24,292,621	24,306,487	2	1	1_1_1_1_1_0_0_0_0_1_1_1_0.43	2	2	2	2	2	2	2	2	1	1	1	1	1
251	2	24,306,488	24,529,146	1	0													
252	2	24,529,147	27,036,589	2	1	1_1_1_1_1_0_0_0_0_1_1_1_0.372	2	2	2	2	2	2	2	2	1	1	1	1	1
253	2	27,036,590	27,044,862	1	0													
254	2	27,044,863	27,236,305	2	1	0_0_0_0_0_0_0_0_0_0_1_0_0.116	1	1	1	1	1	1	1	2	1	1	1	1	1
255	2	27,236,306	27,245,699	1	0													
256	2	27,245,700	28,346,260	2	1	1_1_1_1_1_0_0_0_0_1_1_1_0.111	2	2	2	2	2	2	2	2	1	1	1	1	1
257	2	28,346,261	28,523,162	1	0													
258	2	28,523,163	29,249,740	2	1	0_0_0_0_0_0_0_0_0_0_0_0_1.629	1	1	1	1	1	1	1	1	1	2	1	1	1
259	2	29,249,741	29,250,857	1	0													
260	2	29,250,858	29,933,415	2	1	1_1_1_1_1_0_0_0_0_1_1_1_0.149	2	2	2	2	2	2	2	2	1	1	1	1	1
261	2	29,933,416	29,933,418	3	2	1_1_1_1_1_0_0_0_0_1_1_1_0.149	3	3	3	3	3	3	3	3	1	2	1	1	1
262	2	29,933,419	30,144,009	3	3	0_0_0_0_0_0_0_0_0_0_0_0_1.171	2	2	2	2	2	2	2	2	1	3	1	1	1
263	2	30,144,010	30,189,032	3	2	0_0_0_0_0_0_0_0_0_0_0_0_1.171	2	2	2	2	2	2	2	2	1	3	1	1	1
264	2	30,189,033	30,335,463	2	1	1_1_1_1_1_0_0_0_0_1_1_1_1.395	2	2	2	2	2	2	2	2	1	2	1	1	1
265	2	30,335,464	30,344,818	3	2	1_1_1_1_1_0_0_0_0_1_1_1_0.21	3	3	3	3	3	3	3	3	1	2	1	1	1
266	2	30,344,819	30,360,978	3	3	0_0_0_0_0_0_0_0_0_0_0_0_1.357	2	2	2	2	2	2	2	2	1	3	1	1	1
267	2	30,360,979	30,367,076	4	4	0_0_0_0_0_0_0_0_0_0_0_0_1.357	2	2	2	3	3	3	3	3	1	4	1	1	1
268	2	30,367,077	30,368,811	4	5	0_0_0_0_0_0_0_0_0_0_0_0_1.357	3	3	3	2	2	2	2	2	1	4	1	1	1
269	2	30,368,812	30,389,385	4	6	0_0_0_0_0_0_0_0_0_0_0_0_1.357	3	3	3	1	1	1	1	1	2	4	2	2	2
270	2	30,389,386	30,473,511	4	7	0_0_0_0_0_0_0_0_0_0_0_0_1.357	3	3	3	1	1	1	1	1	2	4	2	2	2
271	2	30,473,512	30,538,564	4	6	0_0_0_0_0_0_0_0_0_0_0_0_1.357	3	3	3	1	1	1	1	1	2	4	2	2	2
272	2	30,538,565	30,545,068	4	5	0_0_0_0_0_0_0_0_0_0_0_0_1.357	3	3	3	1	1	1	1	1	2	4	2	2	2
273	2	30,545,069	30,569,290	4	6	0_0_0_0_0_0_0_0_0_0_0_0_1.357	3	3	3	1	1	1	1	1	2	4	2	2	2
274	2	30,569,291	30,604,866	4	5	0_0_0_0_0_0_0_0_0_0_0_0_1.357	3	3	3	1	1	1	1	1	2	4	2	2	2
275	2	30,604,867	30,621,186	4	4	0_0_0_0_0_0_0_0_0_0_0_0_1.357	3	3	3	2	2	2	2	2	1	4	1	1	1
276	2	30,621,187	30,635,878	4	3	0_0_0_0_0_0_0_0_0_0_0_0_1.357	1	1	1	3	3	3	3	3	2	4	2	2	2
277	2	30,635,879	30,663,865	3	2	0_0_0_0_0_0_0_0_0_0_0_0_1.357	1	1	1	2	2	2	2	2	2	3	2	2	2
278	2	30,663,866	30,677,186	3	3	0_0_0_0_0_0_0_0_0_0_0_0_1.357	2	2	2	1	1	1	1	1	1	3	1	1	1
279	2	30,677,187	30,762,505	4	4	0_0_0_0_0_0_0_0_0_0_0_0_1.357	3	3	3	2	2	2	2	2	1	4	1	1	1
280	2	30,762,506	30,766,747	4	3	0_0_0_0_0_0_0_0_0_0_0_0_1.357	3	3	3	2	2	2	2	2	1	4	1	1	1
281	2	30,766,748	30,783,140	3	2	0_0_0_0_0_0_0_0_0_0_0_0_1.357	2	2	2	1	1	1	1	1	1	3	1	1	1
282	2	30,783,141	30,798,531	4	3	0_0_0_0_0_0_0_0_0_0_0_0_1.357	3	3	3	2	2	2	2	2	1	4	1	1	1
283	2	30,798,532	30,851,536	4	4	0_0_0_0_0_0_0_0_0_0_0_0_1.357	3	3	3	1	1	1	1	1	2	4	2	2	2
284	2	30,851,537	30,851,723	4	5	0_0_0_0_0_0_0_0_0_0_0_0_1.357	3	3	3	1	1	1	1	1	2	4	2	2	2
285	2	30,851,724	30,861,754	4	4	0_0_0_0_0_0_0_0_0_0_0_0_1.357	3	3	3	2	2	2	2	2	1	4	1	1	1
286	2	30,861,755	30,992,248	3	3	0_0_0_0_0_0_0_0_0_0_0_0_1.357	2	2	2	1	1	1	1	1	1	3	1	1	1
287	2	30,992,249	30,992,582	3	2	0_0_0_0_0_0_0_0_0_0_0_0_1.357	2	2	2	1	1	1	1	1	1	3	1	1	1
288	2	30,992,583	30,996,213	2	1	0_1_0_0_0_0_0_0_0_1_0_1_0.520	2	2	2	1	1	1	1	1	1	1	1	1	1
289	2	30,996,214	31,225,405	3	2	0_1_0_0_0_0_0_0_0_1_0_1_0.520	3	3	3	1	1	1	1	1	2	1	2	2	2
290	2	31,225,406	31,255,859	3	3	0_1_0_0_0_0_0_0_0_1_0_1_0.520	3	3	3	1	1	1	1	1	2	1	2	2	2
291	2	31,255,860	31,256,257	3	2	0_1_0_0_0_0_0_0_0_1_0_1_0.520	3	3	3	2	2	2	2	2	1	2	1	1	1
292	2	31,256,258	31,835,608	2	1	1_1_1_1_1_0_0_0_0_1_1_1_1.256	2	2	2	2	2	2	2	2	1	2	1	1	1
293	2	31,835,609	32,657,529	1	0													
294	2	32,657,530	32,789,421	2	1	0_0_0_0_0_0_0_0_0_0_1_0_0.137	1	1	1	1	1	1	1	2	1	1	1	1	1
295	2	32,789,422	32,793,327	1	0													
296	2	32,793,328	32,834,828	2	1	0_1_0_0_0_0_0_0_0_1_1_1_0.84	2	2	2	1	1	1	1	2	1	1	1	1	1
297	2	32,834,829	32,940,663	1	0													
298	2	32,940,664	34,976,063	2	1	1_1_1_1_1_0_0_0_0_1_1_1_0.174	2	2	2	2	2	2	2	2	1	1	1	1	1
299	2	34,976,064	35,072,018	1	0													
300	2	35,072,019	36,316,721	2	1	0_1_0_0_0_1_1_1_1_1_1_1_1.235	2	2	2	1	1	1	1	2	2	2	2	2	2
301	2	36,316,722	36,441,062	1	0													
302	2	36,441,063	37,639,832	2	1	0_1_0_0_0_1_1_1_1_1_1_1_1.587	2	2	2	1	1	1	1	2	2	2	2	2	2
303	2	37,639,833	37,640,910	3	2	0_1_0_0_0_1_1_1_1_1_1_1_1.587	3	3	3	1	1	1	1	3	2	2	2	2	2
304	2	37,640,911	37,686,112	3	3	0_1_0_0_0_0_0_0_0_1_1_1_0.616	3	3	3	1	1	1	1	3	2	2	2	2	2
305	2	37,686,113	37,730,481	3	2	0_1_0_0_0_0_0_0_0_1_1_1_0.616	3	3	3	2	2	2	2	3	1	1	1	1	1
306	2	37,730,482	37,816,175	3	3	0_1_0_0_0_0_0_0_0_1_1_1_0.616	3	3	3	1	1	1	1	3	2	2	2	2	2
307	2	37,816,176	37,816,532	3	2	0_1_0_0_0_0_0_0_0_1_1_1_0.616	3	3	3	1	1	1	1	3	2	2	2	2	2
308	2	37,816,533	38,328,512	2	1	0_1_0_0_0_0_0_0_0_1_1_1_0.616	2	2	2	1	1	1	1	2	1	1	1	1	1
309	2	38,328,513	38,401,098	3	2	0_1_0_0_0_0_0_0_0_1_1_1_0.616	3	3	3	2	2	2	2	3	1	1	1	1	1
310	2	38,401,099	38,531,337	2	1	1_1_1_1_1_0_0_0_0_1_1_1_0.177	2	2	2	2	2	2	2	2	1	1	1	1	1
311	2	38,531,338	38,670,604	3	2	0_1_0_0_0_0_0_0_0_1_1_1_0.229	3	3	3	2	2	2	2	3	1	1	1	1	1
312	2	38,670,605	38,692,786	3	3	0_1_0_0_0_0_0_0_0_1_1_1_0.229	3	3	3	1	1	1	1	3	2	2	2	2	2
313	2	38,692,787	38,718,241	3	2	0_1_0_0_0_0_0_0_0_1_1_1_0.229	3	3	3	1	1	1	1	3	2	2	2	2	2
314	2	38,718,242	38,914,483	2	1	0_1_0_0_0_0_0_0_0_1_1_1_0.229	2	2	2	1	1	1	1	2	1	1	1	1	1
315	2	38,914,484	38,966,600	3	2	0_1_0_0_0_0_0_0_0_1_1_1_0.229	3	3	3	2	2	2	2	3	1	1	1	1	1
316	2	38,966,601	40,068,878	2	1	1_1_1_1_1_0_0_0_0_1_1_1_0.962	2	2	2	2	2	2	2	2	1	1	1	1	1
317	2	40,068,879	40,183,563	1	0													
318	2	40,183,564	40,937,666	2	1	1_1_1_1_1_0_0_0_0_1_1_1_0.515	2	2	2	2	2	2	2	2	1	1	1	1	1
319	2	40,937,667	40,960,157	1	0													
320	2	40,960,158	41,146,134	2	1	0_1_0_0_0_0_0_0_0_1_1_1_0.180	2	2	2	1	1	1	1	2	1	1	1	1	1
321	2	41,146,135	41,153,759	1	0													
322	2	41,153,760	43,615,871	2	1	0_1_0_0_0_0_0_0_0_1_1_1_1.186	2	2	2	1	1	1	1	2	1	2	1	1	1
323	2	43,615,872	43,620,827	1	0													
324	2	43,620,828	45,801,262	2	1	0_0_0_0_0_0_0_0_0_0_0_0_1.197	1	1	1	1	1	1	1	1	1	2	1	1	1
325	2	45,801,263	45,802,491	1	0													
326	2	45,802,492	46,775,132	2	1	0_1_0_0_0_0_0_0_0_1_1_1_0.117	2	2	2	1	1	1	1	2	1	1	1	1	1
327	2	46,775,133	46,783,482	3	2	0_0_0_0_0_0_0_0_0_0_0_0_1.668	2	2	2	1	1	1	1	2	1	3	1	1	1
328	2	46,783,483	46,977,626	3	3	0_0_0_0_0_0_0_0_0_0_0_0_1.668	2	2	2	1	1	1	1	2	1	3	1	1	1
329	2	46,977,627	47,034,380	3	2	0_0_0_0_0_0_0_0_0_0_0_0_1.668	2	2	2	1	1	1	1	2	1	3	1	1	1
330	2	47,034,381	47,407,769	3	3	0_0_0_0_0_0_0_0_0_0_0_0_1.668	2	2	2	1	1	1	1	2	1	3	1	1	1
331	2	47,407,770	47,487,395	3	2	0_0_0_0_0_0_0_0_0_0_0_0_1.668	2	2	2	1	1	1	1	2	1	3	1	1	1
332	2	47,487,396	47,612,291	2	1	0_0_0_0_0_0_0_0_0_0_0_0_1.668	1	1	1	1	1	1	1	1	1	2	1	1	1
333	2	47,612,292	47,672,103	3	2	0_0_0_0_0_0_0_0_0_0_0_0_1.668	2	2	2	1	1	1	1	2	1	3	1	1	1

334	2	47,672,104	47,817,707	3	3	0_0_0_0_0_0_0_0_0_0_0_0_1.668	2	2	2	1	1	1	1	2	1	3	1	1	1
335	2	47,817,708	47,978,637	3	2	0_0_0_0_0_0_0_0_0_0_0_0_1.668	2	2	2	1	1	1	1	2	1	3	1	1	1
336	2	47,978,638	48,130,492	3	3	0_0_0_0_0_0_0_0_0_0_0_0_1.668	2	2	2	1	1	1	1	2	1	3	1	1	1
337	2	48,130,493	48,154,067	3	2	0_0_0_0_0_0_0_0_0_0_0_0_1.668	2	2	2	1	1	1	1	2	1	3	1	1	1
338	2	48,154,068	48,238,241	2	1	0_1_0_0_0_0_0_0_0_1_1_1_0.323	2	2	2	1	1	1	1	2	1	1	1	1	1
339	2	48,238,242	48,244,381	1	0														
340	2	48,244,382	48,335,245	2	1	0_1_0_0_0_0_0_0_0_1_1_1_1.36	2	2	2	1	1	1	1	2	1	2	1	1	1
341	2	48,335,246	48,358,796	3	2	0_0_0_0_0_0_0_0_0_0_0_0_1.201	2	2	2	1	1	1	1	2	1	3	1	1	1
342	2	48,358,797	48,361,555	3	3	0_0_0_0_0_0_0_0_0_0_0_0_1.201	2	2	2	1	1	1	1	2	1	3	1	1	1
343	2	48,361,556	48,452,990	3	2	0_0_0_0_0_0_0_0_0_0_0_0_1.201	2	2	2	1	1	1	1	2	1	3	1	1	1
344	2	48,452,991	48,729,727	3	3	0_0_0_0_0_0_0_0_0_0_0_0_1.201	2	2	2	1	1	1	1	2	1	3	1	1	1
345	2	48,729,728	48,733,863	3	2	0_1_0_0_0_0_0_0_0_1_1_1_0.297	3	3	3	1	1	1	1	3	1	2	1	1	1
346	2	48,733,864	48,774,380	2	1	0_1_0_0_0_0_0_0_0_1_1_1_0.297	2	2	2	1	1	1	1	2	1	1	1	1	1
347	2	48,774,381	48,857,186	3	2	0_0_0_0_0_0_0_0_0_0_0_0_1.138	2	2	2	1	1	1	1	2	1	3	1	1	1
348	2	48,857,187	48,916,939	2	1	0_0_0_0_0_0_0_0_0_0_0_0_1.138	1	1	1	1	1	1	1	1	1	2	1	1	1
349	2	48,916,940	48,920,743	1	0														
350	2	48,920,744	48,995,700	2	1	0_1_0_0_0_0_0_0_0_1_1_1_1.55	2	2	2	1	1	1	1	2	1	2	1	1	1
351	2	48,995,701	49,199,150	1	0														
352	2	49,199,151	49,292,440	2	1	0_0_0_0_0_0_0_0_0_0_0_0_1.68	1	1	1	1	1	1	1	1	1	2	1	1	1
353	2	49,292,441	49,745,005	1	0														
354	2	49,745,006	51,189,595	2	1	1_1_1_1_1_0_0_0_0_1_1_1_0.200	2	2	2	2	2	2	2	2	1	1	1	1	1
355	2	51,189,596	51,207,263	1	0														
356	2	51,207,264	51,404,203	2	1	0_1_0_0_0_0_0_0_0_1_1_1_1.297	2	2	2	1	1	1	1	2	1	2	1	1	1
357	2	51,404,204	51,431,364	3	2	0_0_0_0_0_0_0_0_0_0_0_0_1.25	2	2	2	1	1	1	1	2	1	3	1	1	1
358	2	51,431,365	51,475,880	2	1	0_0_0_0_0_0_0_0_0_0_0_0_1.25	1	1	1	1	1	1	1	1	1	2	1	1	1
359	2	51,475,881	51,477,532	1	0														
360	2	51,477,533	51,906,568	2	1	1_1_1_1_1_0_0_0_0_1_1_1_0.245	2	2	2	2	2	2	2	2	1	1	1	1	1
361	2	51,906,569	51,907,004	1	0														
362	2	51,907,005	55,428,909	2	1	0_0_0_0_0_0_0_0_0_0_0_0_1.320	1	1	1	1	1	1	1	1	1	2	1	1	1
363	2	55,428,910	55,449,060	1	0														
364	2	55,449,061	56,306,966	2	1	0_1_0_0_0_0_0_0_0_1_1_1_0.104	2	2	2	1	1	1	1	2	1	1	1	1	1
365	2	56,306,967	56,605,148	3	2	0_0_0_0_0_0_0_0_0_0_0_0_1.112	2	2	2	1	1	1	1	2	1	3	1	1	1
366	2	56,605,149	56,619,874	2	1	0_0_0_0_0_0_0_0_0_0_0_0_1.112	1	1	1	1	1	1	1	1	1	2	1	1	1
367	2	56,619,875	56,668,979	1	0														
368	2	56,668,980	59,000,469	2	1	0_1_0_0_0_0_0_0_0_1_1_1_1.107	2	2	2	1	1	1	1	2	1	2	1	1	1
369	2	59,000,470	59,279,219	1	0														
370	2	59,279,220	59,832,367	2	1	1_1_1_1_1_0_0_0_0_1_1_1_1.421	2	2	2	2	2	2	2	2	1	2	1	1	1
371	2	59,832,368	62,066,583	1	0														
372	2	62,066,584	62,640,195	2	1	0_1_0_0_0_0_0_0_0_1_1_1_0.538	2	2	2	1	1	1	1	2	1	1	1	1	1
373	2	62,640,196	64,250,447	1	0														
374	2	64,250,448	64,995,424	2	1	1_1_1_1_1_0_0_0_0_1_1_1_1.554	2	2	2	2	2	2	2	2	1	2	1	1	1
375	2	64,995,425	65,185,645	1	0														
376	2	65,185,646	65,763,290	2	1	1_1_1_1_1_0_0_0_0_1_1_1_1.284	2	2	2	2	2	2	2	2	1	2	1	1	1
377	2	65,763,291	65,766,258	1	0														
378	2	65,766,259	67,362,645	2	1	1_1_1_1_1_0_0_0_0_1_1_1_0.212	2	2	2	2	2	2	2	2	1	1	1	1	1
379	2	67,362,646	75,386,345	1	0														
380	2	75,386,346	75,865,286	2	1	1_1_1_1_1_0_0_0_0_1_1_1_0.577	2	2	2	2	2	2	2	2	1	1	1	1	1
381	2	75,865,287	75,873,680	1	0														
382	2	75,873,681	76,541,283	2	1	1_1_1_1_1_0_0_0_0_1_1_1_1.389	2	2	2	2	2	2	2	2	1	2	1	1	1
383	2	76,541,284	77,452,956	1	0														
384	2	77,452,957	77,599,606	2	1	1_1_1_1_1_0_0_0_0_1_1_1_1.62	2	2	2	2	2	2	2	2	1	2	1	1	1
385	2	77,599,607	77,609,426	3	2	1_0_1_1_1_0_0_0_0_0_0_0_1.577	2	2	2	3	3	3	3	2	1	3	1	1	1
386	2	77,609,427	77,728,340	3	3	0_1_0_0_0_0_0_0_0_1_1_1_0.142	3	3	3	2	2	2	2	3	1	2	1	1	1
387	2	77,728,341	77,857,162	3	2	0_1_0_0_0_0_0_0_0_1_1_1_0.142	3	3	3	2	2	2	2	3	1	2	1	1	1
388	2	77,857,163	78,214,871	3	3	0_1_0_0_0_0_0_0_0_1_1_1_0.142	3	3	3	2	2	2	2	3	1	2	1	1	1
389	2	78,214,872	78,543,690	3	2	0_1_0_0_0_0_0_0_0_1_1_1_0.142	3	3	3	2	2	2	2	3	1	2	1	1	1
390	2	78,543,691	78,795,875	2	1	0_1_0_0_0_0_0_0_0_1_1_1_0.142	2	2	2	1	1	1	1	2	1	1	1	1	1
391	2	78,795,876	79,049,202	3	2	0_1_0_0_0_0_0_0_0_1_1_1_0.142	3	3	3	2	2	2	2	3	1	2	1	1	1
392	2	79,049,203	79,153,148	2	1	0_1_0_0_0_0_0_0_0_1_1_1_0.142	2	2	2	1	1	1	1	2	1	1	1	1	1
393	2	79,153,149	79,217,625	3	2	0_1_0_0_0_0_0_0_0_1_1_1_0.142	3	3	3	2	2	2	2	3	1	2	1	1	1
394	2	79,217,626	79,577,349	3	3	0_1_0_0_0_0_0_0_0_1_1_1_0.142	3	3	3	2	2	2	2	3	1	2	1	1	1
395	2	79,577,350	79,578,358	3	2	0_1_0_0_0_0_0_0_0_1_1_1_0.142	3	3	3	2	2	2	2	3	1	2	1	1	1
396	2	79,578,359	79,583,114	2	1	1_1_1_1_1_0_0_0_0_1_1_1_1.162	2	2	2	2	2	2	2	2	1	2	1	1	1
397	2	79,583,115	79,583,681	3	2	0_0_0_0_0_0_0_0_0_0_0_0_1.936	2	2	2	2	2	2	2	2	1	3	1	1	1
398	2	79,583,682	80,105,526	3	3	0_0_0_0_0_0_0_0_0_0_0_0_1.936	2	2	2	2	2	2	2	2	1	3	1	1	1
399	2	80,105,527	80,254,453	3	2	0_0_0_0_0_0_0_0_0_0_0_0_1.936	2	2	2	2	2	2	2	2	1	3	1	1	1
400	2	80,254,454	80,456,494	3	3	0_0_0_0_0_0_0_0_0_0_0_0_1.936	2	2	2	2	2	2	2	2	1	3	1	1	1
401	2	80,456,495	80,572,883	3	2	0_0_0_0_0_0_0_0_0_0_0_0_1.936	2	2	2	2	2	2	2	2	1	3	1	1	1
402	2	80,572,884	80,692,243	3	3	0_0_0_0_0_0_0_0_0_0_0_0_1.936	2	2	2	2	2	2	2	2	1	3	1	1	1
403	2	80,692,244	81,272,165	3	2	0_0_0_0_0_0_0_0_0_0_0_0_1.936	2	2	2	2	2	2	2	2	1	3	1	1	1
404	2	81,272,166	81,321,333	2	1	1_1_1_1_1_0_0_0_0_1_1_1_0.109	2	2	2	2	2	2	2	2	1	1	1	1	1
405	2	81,321,334	81,327,062	3	2	0_0_0_0_0_0_0_0_0_0_0_0_1.145	2	2	2	2	2	2	2	2	1	3	1	1	1
406	2	81,327,063	81,495,659	3	3	0_0_0_0_0_0_0_0_0_0_0_0_1.145	2	2	2	2	2	2	2	2	1	3	1	1	1
407	2	81,495,660	81,636,055	3	2	0_0_0_0_0_0_0_0_0_0_0_0_1.145	2	2	2	2	2	2	2	2	1	3	1	1	1
408	2	81,636,056	82,460,875	2	1	0_0_0_0_0_0_0_0_0_0_0_0_1.145	1	1	1	1	1	1	1	1	1	2	1	1	1
409	2	82,460,876	82,463,009	1	0														
410	2	82,463,010	82,773,743	2	1	1_1_1_1_1_0_0_1_0_1_1_1_0.393	2	2	2	2	2	2	2	2	1	1	1	2	1
411	2	82,773,744	83,395,176	1	0														
412	2	83,395,177	83,415,601	2	1	1_1_1_1_1_0_0_1_0_1_1_1_0.57	2	2	2	2	2	2	2	2	1	1	1	2	1
413	2	83,415,602	86,622,793	1	0														
414	2	86,622,794	86,626,848	2	1	1_1_1_1_1_0_0_1_0_1_0_1_0.217	2	2	2	2	2	2	2	1	1	1	1	2	1
415	2	86,626,849	86,651,193	3	2	0_0_0_0_0_0_0_0_0_0_1_0_0.42	2	2	2	2	2	2	2	3	1	1	1	2	1
416	2	86,651,194	86,823,486	3	3	0_0_0_0_0_0_0_0_0_0_1_0_0.42	2	2	2	2	2	2	2	3	1	1	1	2	1
417	2	86,823,487	86,857,342	3	2	1_1_1_1_1_0_0_1_0_1_0_1_0.217	3	3	3	3	3	3	3	2	1	1	1	3	1
418	2	86,857,343	86,859,622	2	1	1_1_1_1_1_0_0_1_0_1_0_1_0.217	2	2	2	2	2	2	2	1	1	1	1	2	1

419	2	86,859,623	92,063,759	1	0														
420	2	92,063,760	92,090,065	2	1	1_1_1_1_1_0_1_0_0_1_1_1_0.48	2	2	2	2	2	2	2	2	2	1	1	1	1
421	2	92,090,066	98,324,625	1	0														
422	2	98,324,626	98,648,545	2	1	1_1_1_1_1_0_0_0_0_1_1_1_0.230	2	2	2	2	2	2	2	2	1	1	1	1	1
423	2	98,648,546	98,649,064	1	0														
424	2	98,649,065	112,267,754	2	1	1_1_1_1_1_0_0_0_0_1_1_1_1.757	2	2	2	2	2	2	2	2	1	2	1	1	1
425	2	112,267,755	112,272,063	1	0														
426	2	112,272,064	113,583,747	2	1	1_1_1_1_1_0_0_0_0_1_1_1_0.114	2	2	2	2	2	2	2	2	1	1	1	1	1
427	2	113,583,748	113,941,995	1	0														
428	2	113,941,996	121,680,758	2	1	1_1_1_1_1_0_0_0_0_1_1_1_0.696	2	2	2	2	2	2	2	2	1	1	1	1	1
429	2	121,680,759	139,840,216	1	0														
430	2	139,840,217	141,915,621	2	1	1_1_1_1_1_0_0_0_0_1_1_1_0.169	2	2	2	2	2	2	2	2	1	1	1	1	1
431	2	141,915,622	142,693,341	1	0														
432	2	142,693,342	143,444,853	2	1	1_1_1_1_1_0_0_0_0_1_1_1_0.806	2	2	2	2	2	2	2	2	1	1	1	1	1
433	2	143,444,854	146,393,220	1	0														
434	2	146,393,221	146,595,004	2	1	1_0_1_1_1_0_0_0_0_0_0_0_1.297	1	1	1	2	2	2	2	1	1	2	1	1	1
435	2	146,595,005	146,832,240	1	0														
436	2	146,832,241	147,044,873	2	1	1_0_1_1_1_0_0_0_0_0_0_0_1.202	1	1	1	2	2	2	2	1	1	2	1	1	1
437	2	147,044,874	147,585,386	1	0														
438	2	147,585,387	149,029,068	2	1	0_0_0_0_0_0_0_0_0_0_1_0_0.927	1	1	1	1	1	1	1	2	1	1	1	1	1
439	2	149,029,069	149,098,044	1	0														
440	2	149,098,045	151,705,374	2	1	0_0_0_0_0_0_0_0_0_0_1_0_0.207	1	1	1	1	1	1	1	2	1	1	1	1	1
441	2	151,705,375	152,017,286	1	0														
442	2	152,017,287	153,934,956	2	1	0_0_0_0_0_0_0_0_0_0_1_0_0.141	1	1	1	1	1	1	1	2	1	1	1	1	1
443	2	153,934,957	155,799,693	1	0														
444	2	155,799,694	157,279,704	2	1	0_0_0_0_0_0_0_0_0_0_1_0_0.286	1	1	1	1	1	1	1	2	1	1	1	1	1
445	2	157,279,705	170,468,520	1	0														
446	2	170,468,521	172,248,464	2	1	0_0_0_0_0_0_0_0_0_0_1_0_0.175	1	1	1	1	1	1	1	2	1	1	1	1	1
447	2	172,248,465	173,121,019	1	0														
448	2	173,121,020	173,756,133	2	1	0_0_0_0_0_0_0_0_0_0_1_0_0.865	1	1	1	1	1	1	1	2	1	1	1	1	1
449	2	173,756,134	174,114,306	1	0														
450	2	174,114,307	174,483,677	2	1	1_1_1_1_1_0_0_1_0_1_1_1_0.255	2	2	2	2	2	2	2	2	1	1	1	2	1
451	2	174,483,678	174,484,298	3	2	0_0_0_0_0_0_0_0_0_0_1_0_0.728	2	2	2	2	2	2	2	3	1	1	1	2	1
452	2	174,484,299	175,865,890	3	3	0_0_0_0_0_0_0_0_0_0_1_0_0.728	2	2	2	2	2	2	2	3	1	1	1	2	1
453	2	175,865,891	175,928,739	3	2	1_1_1_1_1_0_0_1_0_1_0_1_0.160	3	3	3	3	3	3	3	2	1	1	1	3	1
454	2	175,928,740	176,032,772	3	3	0_0_0_0_0_0_0_0_0_0_1_0_0.39	2	2	2	2	2	2	2	3	1	1	1	2	1
455	2	176,032,773	176,108,748	3	2	1_1_1_1_1_0_0_1_0_1_0_1_0.160	3	3	3	3	3	3	3	2	1	1	1	3	1
456	2	176,108,749	176,768,838	3	3	0_0_0_0_0_0_0_0_0_0_1_0_0.443	2	2	2	2	2	2	2	3	1	1	1	2	1
457	2	176,768,839	176,770,149	3	2	1_1_1_1_1_0_0_1_0_1_0_1_0.160	3	3	3	3	3	3	3	2	1	1	1	3	1
458	2	176,770,150	176,996,467	2	1	1_1_1_1_1_0_0_1_0_1_1_1_0.255	2	2	2	2	2	2	2	2	1	1	1	2	1
459	2	176,996,468	177,050,653	3	2	1_1_1_1_1_0_0_1_0_1_0_1_0.193	3	3	3	3	3	3	3	2	1	1	1	3	1
460	2	177,050,654	177,358,454	3	3	0_0_0_0_0_0_0_0_0_0_1_0_0.150	2	2	2	2	2	2	2	3	1	1	1	2	1
461	2	177,358,455	177,358,964	3	2	0_0_0_0_0_0_0_0_0_0_1_0_0.150	2	2	2	2	2	2	2	3	1	1	1	2	1
462	2	177,358,965	177,359,580	2	1	0_0_0_0_0_0_0_0_0_0_1_0_0.150	1	1	1	1	1	1	1	2	1	1	1	1	1
463	2	177,359,581	177,360,080	3	2	0_0_0_0_0_0_0_0_0_0_1_0_0.150	2	2	2	2	2	2	2	3	1	1	1	1	1
464	2	177,360,081	177,417,832	3	3	0_0_0_0_0_0_0_0_0_0_1_0_0.150	2	2	2	2	2	2	2	3	1	1	1	1	1
465	2	177,417,833	177,486,640	3	2	1_1_1_1_1_0_0_0_0_1_0_1_0.539	3	3	3	3	3	3	3	2	1	1	1	1	1
466	2	177,486,641	177,509,354	2	1	1_1_1_1_1_0_0_0_0_1_0_1_0.539	2	2	2	2	2	2	2	1	1	1	1	1	1
467	2	177,509,355	177,566,007	3	2	0_0_0_0_0_0_0_0_0_0_1_0_0.96	2	2	2	2	2	2	2	3	1	1	1	1	1
468	2	177,566,008	177,783,171	3	3	0_0_0_0_0_0_0_0_0_0_1_0_0.96	2	2	2	2	2	2	2	3	1	1	1	1	1
469	2	177,783,172	177,826,357	3	2	1_1_1_1_1_0_0_0_0_1_0_1_0.539	3	3	3	3	3	3	3	2	1	1	1	1	1
470	2	177,826,358	177,978,889	3	3	0_0_0_0_0_0_0_0_0_0_1_0_0.76	2	2	2	2	2	2	2	3	1	1	1	1	1
471	2	177,978,890	178,236,866	3	2	1_1_1_1_1_0_0_0_0_1_0_1_0.539	3	3	3	3	3	3	3	2	1	1	1	1	1
472	2	178,236,867	178,599,212	2	1	1_1_1_1_1_0_0_0_0_1_1_1_0.406	2	2	2	2	2	2	2	2	1	1	1	1	1
473	2	178,599,213	178,668,688	3	2	0_0_0_0_0_0_0_0_0_0_1_0_0.247	2	2	2	2	2	2	2	3	1	1	1	1	1
474	2	178,668,689	178,776,050	2	1	0_0_0_0_0_0_0_0_0_0_1_0_0.247	1	1	1	1	1	1	1	2	1	1	1	1	1
475	2	178,776,051	178,776,988	3	2	0_0_0_0_0_0_0_0_0_0_1_0_0.247	2	2	2	2	2	2	2	3	1	1	1	1	1
476	2	178,776,989	178,864,187	3	3	0_0_0_0_0_0_0_0_0_0_1_0_0.247	2	2	2	2	2	2	2	3	1	1	1	1	1
477	2	178,864,188	178,891,817	3	2	0_0_0_0_0_0_0_0_0_0_1_0_0.247	2	2	2	2	2	2	2	3	1	1	1	1	1
478	2	178,891,818	179,135,210	2	1	1_1_1_1_1_0_0_0_0_1_1_1_0.507	2	2	2	2	2	2	2	2	1	1	1	1	1
479	2	179,135,211	179,219,959	1	0														
480	2	179,219,960	179,239,544	2	1	0_0_0_0_0_0_0_0_0_0_1_0_0.276	1	1	1	1	1	1	1	2	1	1	1	1	1
481	2	179,239,545	179,323,663	3	2	0_0_0_0_0_0_0_0_0_0_1_0_0.276	2	2	2	2	2	2	2	3	1	1	1	1	1
482	2	179,323,664	179,520,750	3	3	0_0_0_0_0_0_0_0_0_0_1_0_0.276	2	2	2	2	2	2	2	3	1	1	1	1	1
483	2	179,520,751	179,543,777	3	2	0_0_0_0_0_0_0_0_0_0_1_0_0.276	2	2	2	2	2	2	2	3	1	1	1	1	1
484	2	179,543,778	179,926,377	3	3	0_0_0_0_0_0_0_0_0_0_1_0_0.276	2	2	2	2	2	2	2	3	1	1	1	1	1
485	2	179,926,378	179,935,530	3	2	1_1_1_1_1_0_0_0_0_1_0_1_0.278	3	3	3	3	3	3	3	2	1	1	1	1	1
486	2	179,935,531	179,937,402	2	1	1_1_1_1_1_0_0_0_0_1_1_1_0.321	2	2	2	2	2	2	2	2	1	1	1	1	1
487	2	179,937,403	179,941,347	1	0														
488	2	179,941,348	180,125,726	2	1	0_1_0_0_0_0_0_0_0_1_0_1_0.245	2	2	2	1	1	1	1	1	1	1	1	1	1
489	2	180,125,727	180,157,743	1	0														
490	2	180,157,744	181,778,643	2	1	1_1_1_1_1_0_0_0_0_1_1_1_0.143	2	2	2	2	2	2	2	2	1	1	1	1	1
491	2	181,778,644	184,725,944	1	0														
492	2	184,725,945	184,781,830	2	1	1_1_1_1_1_0_1_0_0_1_1_1_0.31	2	2	2	2	2	2	2	2	2	1	1	1	1
493	2	184,781,831	189,030,308	1	0														
494	2	189,030,309	199,739,640	2	1	1_1_1_1_1_0_0_0_0_1_1_1_0.640	2	2	2	2	2	2	2	2	1	1	1	1	1
495	2	199,739,641	199,801,408	1	0														
496	2	199,801,409	200,477,390	2	1	1_1_1_1_1_0_0_0_0_1_1_1_0.585	2	2	2	2	2	2	2	2	1	1	1	1	1
497	2	200,477,391	201,304,914	1	0														
498	2	201,304,915	202,303,709	2	1	1_1_1_1_1_0_0_0_0_1_1_1_0.176	2	2	2	2	2	2	2	2	1	1	1	1	1
499	2	202,303,710	202,304,613	3	2	0_0_0_0_0_0_0_0_0_0_1_0_0.225	2	2	2	2	2	2	2	3	1	1	1	1	1
500	2	202,304,614	203,048,898	3	3	0_0_0_0_0_0_0_0_0_0_1_0_0.225	2	2	2	2	2	2	2	3	1	1	1	1	1
501	2	203,048,899	203,106,642	3	2	0_0_0_0_0_0_0_0_0_0_1_0_0.225	2	2	2	2	2	2	2	3	1	1	1	1	1
502	2	203,106,643	203,188,179	2	1	0_0_0_0_0_0_0_0_0_0_1_0_0.225	1	1	1	1	1	1	1	2	1	1	1	1	1
503	2	203,188,180	203,212,046	3	2	0_0_0_0_0_0_0_0_0_0_1_0_0.225	2	2	2	2	2	2	2	3	1	1	1	1	1

504	2	203,212,047	203,688,491	3	3	0_0_0_0_0_0_0_0_0_1_0_0.225	2	2	2	2	2	2	2	3	1	1	1	1	1
505	2	203,688,492	203,725,561	3	2	0_0_0_0_0_0_0_0_0_1_0_0.225	2	2	2	2	2	2	2	3	1	1	1	1	1
506	2	203,725,562	206,020,275	3	3	0_0_0_0_0_0_0_0_0_1_0_0.225	2	2	2	2	2	2	2	3	1	1	1	1	1
507	2	206,020,276	206,025,814	3	2	0_0_0_0_0_0_0_0_0_1_0_0.225	2	2	2	2	2	2	2	3	1	1	1	1	1
508	2	206,025,815	207,253,169	2	1	1_1_1_1_1_0_0_0_0_1_1_1_0.192	2	2	2	2	2	2	2	2	1	1	1	1	1
509	2	207,253,170	207,298,915	3	2	1_1_1_1_1_0_0_0_0_1_0_1_0.118	3	3	3	3	3	3	3	2	1	1	1	1	1
510	2	207,298,916	207,713,827	2	1	1_1_1_1_1_0_0_0_0_1_0_1_0.118	2	2	2	2	2	2	2	1	1	1	1	1	1
511	2	207,713,828	208,867,193	3	2	0_0_0_0_0_0_0_0_0_1_0_1.578	2	2	2	2	2	2	2	3	1	3	1	1	1
512	2	208,867,194	208,913,444	2	1	1_1_1_1_1_0_0_0_0_1_0_1_0.118	2	2	2	2	2	2	2	1	1	1	1	1	1
513	2	208,913,445	209,565,206	3	2	0_0_0_0_0_0_0_0_0_1_0_1.727	2	2	2	2	2	2	2	3	1	3	1	1	1
514	2	209,565,207	209,794,898	3	3	0_0_0_0_0_0_0_0_0_1_0_1.727	2	2	2	2	2	2	2	3	1	3	1	1	1
515	2	209,794,899	209,922,119	3	2	0_0_0_0_0_0_0_0_0_1_0_1.727	2	2	2	2	2	2	2	3	1	3	1	1	1
516	2	209,922,120	210,014,948	2	1	0_0_0_0_0_0_0_0_0_1_0_1.727	1	1	1	1	1	1	1	2	1	2	1	1	1
517	2	210,014,949	210,017,247	1	0														
518	2	210,017,248	210,217,725	2	1	1_1_1_1_1_0_0_0_0_1_0_1_0.235	2	2	2	2	2	2	2	1	1	1	1	1	1
519	2	210,217,726	210,448,939	3	2	0_0_0_0_0_0_0_0_0_1_0_1.119	2	2	2	2	2	2	2	3	1	3	1	1	1
520	2	210,448,940	210,478,159	2	1	0_0_0_0_0_0_0_0_0_1_0_1.119	1	1	1	1	1	1	1	2	1	2	1	1	1
521	2	210,478,160	210,526,684	1	0														
522	2	210,526,685	211,034,632	2	1	1_1_1_1_1_0_0_0_0_1_1_1_1.334	2	2	2	2	2	2	2	2	1	2	1	1	1
523	2	211,034,633	211,040,401	1	0														
524	2	211,040,402	211,327,186	2	1	1_1_1_1_1_0_0_0_0_1_1_1_0.217	2	2	2	2	2	2	2	2	1	1	1	1	1
525	2	211,327,187	211,340,169	1	0														
526	2	211,340,170	212,232,015	2	1	0_0_0_0_0_0_0_0_0_1_0_0.702	1	1	1	1	1	1	1	2	1	1	1	1	1
527	2	212,232,016	212,330,382	1	0														
528	2	212,330,383	213,506,787	2	1	0_0_0_0_0_0_0_0_0_1_0_0.512	1	1	1	1	1	1	1	2	1	1	1	1	1
529	2	213,506,788	213,507,391	3	2	0_0_0_0_0_0_0_0_0_1_0_0.512	2	2	2	2	2	2	2	3	1	1	1	1	1
530	2	213,507,392	213,578,514	3	3	0_0_0_0_0_0_0_0_0_1_0_0.512	2	2	2	2	2	2	2	3	1	1	1	1	1
531	2	213,578,515	213,580,315	3	2	0_0_0_0_0_0_0_0_0_1_0_0.512	2	2	2	2	2	2	2	3	1	1	1	1	1
532	2	213,580,316	213,580,978	2	1	1_1_1_1_1_0_0_0_0_1_0_1_0.616	2	2	2	2	2	2	2	1	1	1	1	1	1
533	2	213,580,979	213,584,490	3	2	0_0_0_0_0_0_0_0_0_0_0_1.195	2	2	2	2	2	2	2	1	1	3	1	1	1
534	2	213,584,491	214,434,355	3	3	0_0_0_0_0_0_0_0_0_0_0_1.195	1	1	1	1	1	1	1	2	2	3	2	2	2
535	2	214,434,356	214,473,749	3	2	0_0_0_0_0_0_0_0_0_0_0_1.195	1	1	1	1	1	1	1	2	2	3	2	2	2
536	2	214,473,750	215,019,087	3	3	0_0_0_0_0_0_0_0_0_0_0_1.195	1	1	1	1	1	1	1	2	2	3	2	2	2
537	2	215,019,088	215,020,148	3	2	0_0_0_0_0_0_0_0_0_0_0_1.195	1	1	1	1	1	1	1	2	2	3	2	2	2
538	2	215,020,149	215,569,266	2	1	0_0_0_0_0_0_0_0_0_0_0_1.195	1	1	1	1	1	1	1	1	1	2	1	1	1
539	2	215,569,267	215,572,707	3	2	0_0_0_0_0_0_0_0_0_0_0_1.195	2	2	2	2	2	2	2	1	1	3	1	1	1
540	2	215,572,708	215,709,549	3	3	0_0_0_0_0_0_0_0_0_0_0_1.195	1	1	1	1	1	1	1	2	2	3	2	2	2
541	2	215,709,550	215,709,926	3	2	0_0_0_0_0_0_0_0_0_0_0_1.195	2	2	2	2	2	2	2	1	1	3	1	1	1
542	2	215,709,927	216,277,720	2	1	0_0_0_0_0_0_0_0_0_0_0_1.195	1	1	1	1	1	1	1	1	1	2	1	1	1
543	2	216,277,721	216,310,891	1	0														
544	2	216,310,892	217,141,195	2	1	0_0_0_0_0_0_0_0_0_0_0_1.268	1	1	1	1	1	1	1	1	1	2	1	1	1
545	2	217,141,196	217,143,761	3	2	0_0_0_0_0_0_0_0_0_0_0_1.268	1	1	1	1	1	1	1	2	2	3	2	2	2
546	2	217,143,762	217,522,235	3	3	0_0_0_0_0_0_0_0_0_0_0_1.268	1	1	1	1	1	1	1	2	2	3	2	2	2
547	2	217,522,236	217,538,310	3	2	0_0_0_0_0_0_0_0_0_0_0_1.268	1	1	1	1	1	1	1	2	2	3	2	2	2
548	2	217,538,311	218,255,268	2	1	0_0_0_0_0_0_0_0_0_0_0_1.268	1	1	1	1	1	1	1	1	1	2	1	1	1
549	2	218,255,269	218,255,396	3	2	0_0_0_0_0_0_0_0_0_0_0_1.268	1	1	1	1	1	1	1	2	2	3	2	2	2
550	2	218,255,397	219,341,826	3	3	0_0_0_0_0_0_0_0_0_0_0_1.268	1	1	1	1	1	1	1	2	2	3	2	2	2
551	2	219,341,827	219,341,965	3	2	0_0_0_0_0_1_1_1_1_0_1_0_0.636	2	2	2	2	2	2	2	3	3	1	3	3	3
552	2	219,341,966	219,869,904	2	1	1_1_1_1_1_0_0_0_0_1_0_1_0.154	2	2	2	2	2	2	2	1	1	1	1	1	1
553	2	219,869,905	219,874,659	3	2	0_0_0_0_0_0_0_0_0_0_0_1.223	2	2	2	2	2	2	2	1	1	3	1	1	1
554	2	219,874,660	219,946,542	2	1	0_0_0_0_0_0_0_0_0_0_0_1.223	1	1	1	1	1	1	1	1	1	2	1	1	1
555	2	219,946,543	219,946,823	3	2	0_0_0_0_0_0_0_0_0_0_0_1.223	2	2	2	2	2	2	2	1	1	3	1	1	1
556	2	219,946,824	220,292,851	3	3	0_0_0_0_0_0_0_0_0_0_0_1.223	1	1	1	1	1	1	1	2	2	3	2	2	2
557	2	220,292,852	220,294,975	3	2	0_0_0_0_0_0_0_0_0_0_0_1.223	2	2	2	2	2	2	2	1	1	3	1	1	1
558	2	220,294,976	220,902,465	2	1	1_1_1_1_1_0_0_0_0_1_0_1_0.131	2	2	2	2	2	2	2	1	1	1	1	1	1
559	2	220,902,466	220,911,834	1	0														
560	2	220,911,835	222,701,103	2	1	0_0_0_0_0_0_0_0_0_0_0_1.146	1	1	1	1	1	1	1	1	1	2	1	1	1
561	2	222,701,104	222,704,195	3	2	0_0_0_0_0_0_0_0_0_0_0_1.146	1	1	1	1	1	1	1	2	2	3	2	2	2
562	2	222,704,196	222,802,228	3	3	0_0_0_0_0_0_0_0_0_0_0_1.146	1	1	1	1	1	1	1	2	2	3	2	2	2
563	2	222,802,229	222,822,152	3	2	0_0_0_0_0_1_1_1_1_0_1_0_0.44	2	2	2	2	2	2	2	3	3	1	3	3	3
564	2	222,822,153	222,830,803	2	1	1_1_1_1_1_0_0_0_0_1_0_1_0.163	2	2	2	2	2	2	2	1	1	1	1	1	1
565	2	222,830,804	222,836,089	3	2	1_1_1_1_1_0_0_0_0_1_0_1_0.163	3	3	3	3	3	3	3	2	1	1	1	1	1
566	2	222,836,090	222,895,570	3	3	0_0_0_0_0_0_0_0_0_1_0_0.79	2	2	2	2	2	2	2	3	1	1	1	1	1
567	2	222,895,571	223,047,160	3	2	0_0_0_0_0_0_0_0_0_1_0_0.79	2	2	2	2	2	2	2	3	1	1	1	1	1
568	2	223,047,161	223,130,500	4	3	0_0_0_0_0_0_0_0_0_1_0_0.79	3	3	3	3	3	3	3	4	2	1	1	1	1
569	2	223,130,501	223,160,800	3	2	0_0_0_0_0_0_0_0_0_1_0_0.79	2	2	2	2	2	2	2	3	1	1	1	1	1
570	2	223,160,801	223,346,186	3	3	0_0_0_0_0_0_0_0_0_1_0_0.79	2	2	2	2	2	2	2	3	1	1	1	1	1
571	2	223,346,187	223,346,500	3	2	1_1_1_1_1_0_0_0_0_1_0_1_0.62	3	3	3	3	3	3	3	2	1	1	1	1	1
572	2	223,346,501	223,349,533	2	1	1_1_1_1_1_0_0_0_0_1_1_1_0.251	2	2	2	2	2	2	2	2	1	1	1	1	1
573	2	223,349,534	223,400,739	3	2	0_0_0_0_0_0_0_0_0_0_0_1.121	2	2	2	2	2	2	2	2	1	3	1	1	1
574	2	223,400,740	223,401,144	4	3	0_0_0_0_0_0_0_0_0_0_0_1.121	2	2	2	2	2	2	2	3	1	4	1	1	1
575	2	223,401,145	223,414,645	4	4	0_0_0_0_0_0_0_0_0_0_0_1.121	2	2	2	2	2	2	2	3	1	4	1	1	1
576	2	223,414,646	223,669,096	4	5	0_0_0_0_0_0_0_0_0_0_0_1.121	2	2	2	2	2	2	2	3	1	4	1	1	1
577	2	223,669,097	223,669,312	4	4	0_0_0_0_0_0_0_0_0_0_0_1.121	2	2	2	2	2	2	2	3	1	4	1	1	1
578	2	223,669,313	223,669,360	4	3	0_0_0_0_0_0_0_0_0_0_0_1.121	3	3	3	3	3	3	3	2	1	4	1	1	1
579	2	223,669,361	223,761,011	4	4	0_0_0_0_0_0_0_0_0_0_0_1.121	3	3	3	3	3	3	3	2	1	4	1	1	1
580	2	223,761,012	223,761,025	4	3	0_0_0_0_0_0_0_0_0_0_0_1.121	3	3	3	3	3	3	3	2	1	4	1	1	1
581	2	223,761,026	223,761,693	3	2	1_1_1_1_1_0_0_0_0_1_0_1_0.199	3	3	3	3	3	3	3	2	1	2	1	1	1
582	2	223,761,694	223,788,123	2	1	1_1_1_1_1_0_0_0_0_1_0_1_0.199	2	2	2	2	2	2	2	1	1	1	1	1	1
583	2	223,788,124	223,788,790	1	0														
584	2	223,788,791	223,806,962	2	1	0_0_0_0_0_0_0_0_0_1_0_0.528	1	1	1	1	1	1	1	2	1	1	1	1	1
585	2	223,806,963	223,869,088	3	2	0_0_0_0_0_0_0_0_0_1_0_0.528	1	1	1	1	1	1	1	3	2	1	2	2	2
586	2	223,869,089	224,948,367	3	3	0_0_0_0_0_0_0_0_0_1_0_0.528	1	1	1	1	1	1	1	3	2	1	2	2	2
587	2	224,948,368	224,948,782	4	4	0_0_0_0_0_0_0_0_0_1_0_0.528	1	1	1	2	2	2	2	4	3	2	3	3	3
588	2	224,948,783	224,957,140	4	5	0_0_0_0_0_0_0_0_0_1_0_0.528	2	2	2	1	1	1	1	4	3	1	3	3	3

589	2	224,957,141	224,958,244	4	4	0_0_0_0_0_1_1_1_1_0_1_0_0.485	2	2	2	1	1	1	1	4	3	1	3	3	3
590	2	224,958,245	225,179,385	3	3	0_1_0_0_0_0_0_0_0_1_1_1_0.325	3	3	3	2	2	2	2	3	1	2	1	1	1
591	2	225,179,386	225,216,559	3	2	0_1_0_0_0_0_0_0_0_1_1_1_0.325	3	3	3	2	2	2	2	3	1	2	1	1	1
592	2	225,216,560	225,274,124	3	3	0_1_0_0_0_0_0_0_0_1_1_1_0.325	3	3	3	2	2	2	2	3	1	2	1	1	1
593	2	225,274,125	225,296,671	3	2	0_1_0_0_0_0_0_0_0_1_1_1_0.325	3	3	3	2	2	2	2	3	1	2	1	1	1
594	2	225,296,672	225,944,207	2	1	1_0_1_1_1_0_0_0_0_0_0_0_1.102	1	1	1	2	2	2	2	1	1	2	1	1	1
595	2	225,944,208	225,961,606	3	2	1_0_1_1_1_0_0_0_0_0_0_0_1.102	2	2	2	3	3	3	3	2	1	3	1	1	1
596	2	225,961,607	225,997,371	2	1	1_0_1_1_1_0_0_0_0_0_0_0_1.102	1	1	1	2	2	2	2	1	1	2	1	1	1
597	2	225,997,372	226,006,629	1	0														
598	2	226,006,630	226,007,077	2	1	0_0_0_0_0_1_1_1_1_0_1_0_0.230	1	1	1	1	1	1	1	2	2	1	2	2	2
599	2	226,007,078	226,010,214	3	2	0_0_0_0_0_0_0_0_0_0_0_0_1.482	1	1	1	1	1	1	1	2	2	3	2	2	2
600	2	226,010,215	226,311,641	3	3	0_0_0_0_0_0_0_0_0_0_0_0_1.482	1	1	1	1	1	1	1	2	2	3	2	2	2
601	2	226,311,642	226,313,974	3	2	0_0_0_0_0_0_0_0_0_0_0_0_1.482	1	1	1	1	1	1	1	2	2	3	2	2	2
602	2	226,313,975	226,486,938	2	1	0_0_0_0_0_0_0_0_0_0_0_0_1.482	1	1	1	1	1	1	1	1	1	2	1	1	1
603	2	226,486,939	226,555,064	1	0														
604	2	226,555,065	226,887,930	2	1	0_1_0_0_0_0_0_0_0_1_1_1_0.270	2	2	2	1	1	1	1	2	1	1	1	1	1
605	2	226,887,931	227,012,740	1	0														
606	2	227,012,741	227,049,765	2	1	0_1_0_0_0_0_0_0_0_1_1_1_0.105	2	2	2	1	1	1	1	2	1	1	1	1	1
607	2	227,049,766	227,097,283	3	2	0_1_0_0_0_0_0_0_0_1_1_1_0.105	3	3	3	2	2	2	2	3	1	2	1	1	1
608	2	227,097,284	228,673,321	2	1	1_0_1_1_1_0_0_0_0_0_0_0_1.228	1	1	1	2	2	2	2	1	1	2	1	1	1
609	2	228,673,322	228,783,689	1	0														
610	2	228,783,690	229,119,007	2	1	1_0_1_1_1_0_0_0_0_0_0_0_1.575	1	1	1	2	2	2	2	1	1	2	1	1	1
611	2	229,119,008	229,225,533	1	0														
612	2	229,225,534	229,852,405	2	1	1_0_1_1_1_0_0_0_0_0_0_0_1.658	1	1	1	2	2	2	2	1	1	2	1	1	1
613	2	229,852,406	230,216,797	1	0														
614	2	230,216,798	230,575,831	2	1	0_1_0_0_0_1_1_1_1_1_1_1_1.175	2	2	2	1	1	1	1	2	2	2	2	2	2
615	2	230,575,832	231,566,217	1	0														
616	2	231,566,218	233,163,150	2	1	1_0_1_1_1_0_0_0_0_0_0_0_1.181	1	1	1	2	2	2	2	1	1	2	1	1	1
617	2	233,163,151	233,171,773	1	0														
618	2	233,171,774	234,733,315	2	1	0_1_0_0_0_1_1_1_1_1_1_1_1.339	2	2	2	1	1	1	1	2	2	2	2	2	2
619	2	234,733,316	234,737,454	1	0														
620	2	234,737,455	235,045,015	2	1	0_1_0_0_0_0_0_0_0_1_1_1_0.503	2	2	2	1	1	1	1	2	1	1	1	1	1
621	2	235,045,016	235,231,496	1	0														
622	2	235,231,497	235,326,642	2	1	0_1_0_0_0_1_1_1_1_1_1_1_1.44	2	2	2	1	1	1	1	2	2	2	2	2	2
623	2	235,326,643	235,361,512	1	0														
624	2	235,361,513	235,409,830	2	1	0_1_0_0_0_0_0_0_0_1_1_1_0.125	2	2	2	1	1	1	1	2	1	1	1	1	1
625	2	235,409,831	235,410,843	1	0														
626	2	235,410,844	235,499,806	2	1	0_0_0_0_0_0_0_0_0_0_0_0_1.85	1	1	1	1	1	1	1	1	1	2	1	1	1
627	2	235,499,807	235,501,919	1	0														
628	2	235,501,920	235,709,086	2	1	0_1_0_0_0_1_1_1_1_1_1_1_1.211	2	2	2	1	1	1	1	2	2	2	2	2	2
629	2	235,709,087	235,743,464	1	0														
630	2	235,743,465	237,417,686	2	1	0_1_0_0_0_1_1_1_1_1_1_1_1.774	2	2	2	1	1	1	1	2	2	2	2	2	2
631	2	237,417,687	237,641,914	1	0														
632	2	237,641,915	238,626,634	2	1	0_1_0_0_0_1_1_1_1_1_1_1_1.476	2	2	2	1	1	1	1	2	2	2	2	2	2
633	2	238,626,635	238,891,497	1	0														
634	2	238,891,498	239,283,015	2	1	0_1_0_0_0_1_1_1_1_1_1_1_1.819	2	2	2	1	1	1	1	2	2	2	2	2	2
635	2	239,283,016	239,288,862	3	2	0_0_0_0_0_0_0_1_0_0_0_0_1.28	2	2	2	1	1	1	1	2	2	3	2	3	2
636	2	239,288,863	239,333,479	3	3	0_0_0_0_0_0_0_1_0_0_0_0_1.28	2	2	2	1	1	1	1	2	2	3	2	3	2
637	2	239,333,480	239,348,439	3	2	0_0_0_0_0_0_0_1_0_0_0_0_1.28	2	2	2	1	1	1	1	2	2	3	2	3	2
638	2	239,348,440	240,146,084	2	1	0_1_0_0_0_1_1_1_1_1_1_1_1.819	2	2	2	1	1	1	1	2	2	2	2	2	2
639	2	240,146,085	240,399,468	1	0														
640	2	240,399,469	241,316,188	2	1	0_1_0_0_0_1_1_1_1_1_1_1_1.319	2	2	2	1	1	1	1	2	2	2	2	2	2
641	2	241,316,189	241,352,978	1	0														
642	2	241,352,979	242,649,989	2	1	0_1_0_0_0_1_1_1_1_1_1_1_1.639	2	2	2	1	1	1	1	2	2	2	2	2	2
643	2	242,649,990	248,724,579	1	0														
644	2	248,724,580	249,886,592	2	1	1_1_1_1_1_1_1_0_1_1_1_1_1.538	2	2	2	2	2	2	2	2	2	2	2	1	2
645	2	249,886,593	249,888,246	1	0														
646	2	249,888,247	250,461,967	2	1	0_0_0_0_0_0_0_1_0_0_0_0_1.960	1	1	1	1	1	1	1	1	1	2	1	2	1
647	2	250,461,968	250,616,475	3	2	0_0_0_0_0_0_0_1_0_0_0_0_1.960	1	2	2	2	2	2	2	2	2	3	2	3	2
648	2	250,616,476	250,836,179	3	3	0_0_0_0_0_0_0_1_0_0_0_0_1.960	1	2	2	2	2	2	2	2	2	3	2	3	2
649	2	250,836,180	250,842,608	3	2	0_0_0_0_0_0_0_1_0_0_0_0_1.960	1	2	2	2	2	2	2	2	2	3	2	3	2
650	2	250,842,609	250,997,220	2	1	1_0_1_1_1_1_1_0_1_1_1_1_0.165	1	2	2	2	2	2	2	2	2	1	2	1	2
651	2	250,997,221	250,998,626	3	2	1_0_1_1_1_1_1_0_1_1_1_1_0.165	1	3	3	3	3	3	3	3	3	2	3	2	3
652	2	250,998,627	251,095,653	3	3	0_0_0_0_0_0_0_1_0_0_0_0_1.357	1	2	2	2	2	2	2	2	2	3	2	3	2
653	2	251,095,654	251,218,213	3	2	0_0_0_0_0_0_0_1_0_0_0_0_1.357	1	2	2	2	2	2	2	2	2	3	2	3	2
654	2	251,218,214	251,312,259	3	3	0_0_0_0_0_0_0_1_0_0_0_0_1.357	1	2	2	2	2	2	2	2	2	3	2	3	2
655	2	251,312,260	251,369,523	3	2	0_0_0_0_0_0_0_1_0_0_0_0_1.357	1	2	2	2	2	2	2	2	2	3	2	3	2
656	2	251,369,524	251,555,759	2	1	1_0_1_1_1_1_1_0_1_1_1_1_0.165	1	2	2	2	2	2	2	2	2	1	2	1	2
657	2	251,555,760	251,556,110	3	2	0_0_0_0_0_0_0_1_0_0_0_0_1.476	1	2	2	2	2	2	2	2	2	3	2	3	2
658	2	251,556,111	251,846,317	3	3	0_0_0_0_0_0_0_1_0_0_0_0_1.476	1	2	2	2	2	2	2	2	2	3	2	3	2
659	2	251,846,318	251,908,789	3	2	0_0_0_0_0_0_0_1_0_0_0_0_1.476	1	2	2	2	2	2	2	2	2	3	2	3	2
660	2	251,908,790	252,061,553	3	3	0_0_0_0_0_0_0_1_0_0_0_0_1.476	1	2	2	2	2	2	2	2	2	3	2	3	2
661	2	252,061,554	252,107,912	3	2	0_0_0_0_0_0_0_1_0_0_0_0_1.476	1	2	2	2	2	2	2	2	2	3	2	3	2
662	2	252,107,913	252,198,716	2	1	0_0_0_0_0_0_0_1_0_0_0_0_1.476	1	1	1	1	1	1	1	1	1	2	1	2	1
663	2	252,198,717	252,361,103	1	0														
664	2	252,361,104	252,485,611	2	1	1_0_1_1_1_1_1_0_1_1_1_1_0.147	1	2	2	2	2	2	2	2	2	1	2	1	2
665	2	252,485,612	252,728,797	1	0														
666	2	252,728,798	253,156,867	2	1	1_0_1_1_1_0_0_0_0_1_1_1_0.717	1	2	2	2	2	2	2	2	1	1	1	1	1
667	2	253,156,868	253,157,500	1	0														
668	2	253,157,501	253,420,738	2	1	1_0_1_1_1_0_1_0_0_1_1_1_0.362	1	2	2	2	2	2	2	2	2	1	1	1	1
669	2	253,420,739	253,423,363	1	0														
670	2	253,423,364	253,547,497	2	1	1_0_1_1_1_0_1_0_0_1_1_1_1.162	1	2	2	2	2	2	2	2	2	2	1	1	1
671	2	253,547,498	253,558,630	3	2	1_0_1_1_1_0_1_0_0_1_1_1_1.162	2	3	3	3	3	3	3	3	3	3	1	1	1
672	2	253,558,631	253,757,215	3	3	1_0_1_1_1_0_1_0_0_1_1_1_1.162	1	3	3	3	3	3	3	3	3	3	2	2	2
673	2	253,757,216	253,817,891	3	2	1_0_1_1_1_0_1_0_0_1_1_1_1.162	2	3	3	3	3	3	3	3	3	3	1	1	1

674	2	253,817,892	254,146,375	3	3	1_0_1_1_1_0_1_0_0_1_1_1_1.162	1	3	3	3	3	3	3	3	3	3	2	2	2
675	2	254,146,376	254,296,029	3	2	1_0_1_1_1_0_1_0_0_1_1_1_1.162	2	3	3	3	3	3	3	3	3	3	1	1	1
676	2	254,296,030	254,796,388	3	3	1_0_1_1_1_0_1_0_0_1_1_1_1.162	1	3	3	3	3	3	3	3	3	3	2	2	2
677	2	254,796,389	255,020,831	3	2	1_0_1_1_1_0_1_0_0_1_1_1_1.162	1	3	3	3	3	3	3	3	3	3	2	2	2
678	2	255,020,832	255,126,937	3	3	1_0_1_1_1_0_1_0_0_1_1_1_1.162	1	3	3	3	3	3	3	3	3	3	2	2	2
679	2	255,126,938	255,308,084	3	2	1_0_1_1_1_0_1_0_0_1_1_1_1.162	1	3	3	3	3	3	3	3	3	3	2	2	2
680	2	255,308,085	255,361,715	2	1	1_0_1_1_1_0_1_0_0_1_1_1_1.162	1	2	2	2	2	2	2	2	2	2	1	1	1
681	2	255,361,716	255,950,553	3	2	1_0_1_1_1_0_1_0_0_1_1_1_1.162	1	3	3	3	3	3	3	3	3	3	2	2	2
682	2	255,950,554	255,959,217	2	1	1_0_1_1_1_0_1_0_0_1_1_1_1.162	1	2	2	2	2	2	2	2	2	2	1	1	1
683	2	255,959,218	256,032,914	3	2	1_0_1_1_1_0_1_0_0_1_1_1_1.162	2	3	3	3	3	3	3	3	3	3	1	1	1
684	2	256,032,915	256,216,192	3	3	1_0_1_1_1_0_1_0_0_1_1_1_1.162	1	3	3	3	3	3	3	3	3	3	2	2	2
685	2	256,216,193	256,321,660	3	2	1_0_1_1_1_0_1_0_0_1_1_1_1.162	2	3	3	3	3	3	3	3	3	3	1	1	1
686	2	256,321,661	256,322,025	2	1	1_1_1_1_1_0_1_0_0_1_1_1_1.376	2	2	2	2	2	2	2	2	2	2	1	1	1
687	2	256,322,026	256,322,328	3	2	1_1_1_1_1_0_1_0_0_1_1_1_0.946	3	3	3	3	3	3	3	3	3	2	1	1	1
688	2	256,322,329	256,707,366	3	3	0_0_0_0_0_0_0_0_0_0_0_0_1.102	2	2	2	2	2	2	2	2	2	3	1	1	1
689	2	256,707,367	256,825,724	3	2	0_0_0_0_0_0_0_0_0_0_0_0_1.102	2	2	2	2	2	2	2	2	2	3	1	1	1
690	2	256,825,725	257,155,421	3	3	0_0_0_0_0_0_0_0_0_0_0_0_1.102	2	2	2	2	2	2	2	2	2	3	1	1	1
691	2	257,155,422	257,240,747	3	2	0_0_0_0_0_0_0_0_0_0_0_0_1.102	2	2	2	2	2	2	2	2	2	3	1	1	1
692	2	257,240,748	258,056,456	3	3	0_0_0_0_0_0_0_0_0_0_0_0_1.102	2	2	2	2	2	2	2	2	2	3	1	1	1
693	2	258,056,457	258,110,907	3	2	0_0_0_0_0_0_0_0_0_0_0_0_1.102	2	2	2	2	2	2	2	2	2	3	1	1	1
694	2	258,110,908	258,137,582	2	1	1_1_1_1_1_0_1_0_0_1_1_1_1.337	2	2	2	2	2	2	2	2	2	2	1	1	1
695	2	258,137,583	258,145,920	3	2	1_0_1_1_1_0_1_0_0_1_1_1_1.411	2	3	3	3	3	3	3	3	3	3	1	1	1
696	2	258,145,921	258,440,753	3	3	1_0_1_1_1_0_1_0_0_1_1_1_1.411	1	3	3	3	3	3	3	3	3	3	2	2	2
697	2	258,440,754	258,575,378	3	2	1_0_1_1_1_0_1_0_0_1_1_1_1.411	1	3	3	3	3	3	3	3	3	3	2	2	2
698	2	258,575,379	258,684,640	3	3	1_0_1_1_1_0_1_0_0_1_1_1_1.411	1	3	3	3	3	3	3	3	3	3	2	2	2
699	2	258,684,641	258,802,142	3	2	1_0_1_1_1_0_1_0_0_1_1_1_1.411	1	3	3	3	3	3	3	3	3	3	2	2	2
700	2	258,802,143	259,004,859	3	3	1_0_1_1_1_0_1_0_0_1_1_1_1.411	1	3	3	3	3	3	3	3	3	3	2	2	2
701	2	259,004,860	259,012,715	3	2	1_0_1_1_1_0_1_0_0_1_1_1_1.411	2	3	3	3	3	3	3	3	3	3	1	1	1
702	2	259,012,716	259,014,279	2	1	1_1_1_1_1_0_1_0_0_1_1_1_1.85	2	2	2	2	2	2	2	2	2	2	1	1	1
703	2	259,014,280	259,018,686	3	2	1_1_1_1_1_0_1_0_0_1_1_1_0.102	3	3	3	3	3	3	3	3	3	2	1	1	1
704	2	259,018,687	259,137,851	3	3	0_0_0_0_0_0_0_0_0_0_0_0_1.253	2	2	2	2	2	2	2	2	2	3	1	1	1
705	2	259,137,852	259,182,650	3	2	0_0_0_0_0_0_0_0_0_0_0_0_1.253	2	2	2	2	2	2	2	2	2	3	1	1	1
706	2	259,182,651	259,424,738	3	3	0_0_0_0_0_0_0_0_0_0_0_0_1.253	2	2	2	2	2	2	2	2	2	3	1	1	1
707	2	259,424,739	259,431,273	3	2	1_1_1_1_1_0_1_0_0_1_1_1_0.102	3	3	3	3	3	3	3	3	3	2	1	1	1
708	2	259,431,274	259,915,117	2	1	1_1_1_1_1_0_1_0_0_1_1_1_0.102	2	2	2	2	2	2	2	2	2	1	1	1	1
709	2	259,915,118	260,173,433	1	0														
710	2	260,173,434	260,358,233	2	1	1_1_1_1_1_0_1_0_0_1_1_1_0.619	2	2	2	2	2	2	2	2	2	1	1	1	1
711	2	260,358,234	260,378,959	3	2	0_0_0_0_0_0_0_0_0_0_1_0_0.33	2	2	2	2	2	2	2	3	2	1	1	1	1
712	2	260,378,960	266,338,454	2	1	1_1_1_1_1_0_1_0_0_1_1_1_0.619	2	2	2	2	2	2	2	2	2	1	1	1	1
713	2	266,338,455	266,348,315	3	2	0_1_0_0_0_0_0_0_0_1_1_1_0.77	3	3	3	2	2	2	2	3	2	1	1	1	1
714	2	266,348,316	266,432,690	2	1	0_1_0_0_0_0_0_0_0_1_1_1_0.77	2	2	2	1	1	1	1	2	1	1	1	1	1
715	3	5,981,195	6,246,807	2	1	1_1_1_1_1_0_0_0_0_1_1_1_0.202	2	2	2	2	2	2	2	2	1	1	1	1	1
716	3	6,246,808	7,627,936	1	0														
717	3	7,627,937	10,334,219	2	1	1_1_1_1_1_0_0_0_0_1_1_1_0.183	2	2	2	2	2	2	2	2	1	1	1	1	1
718	3	10,334,220	10,412,897	3	2	0_0_0_0_0_0_0_0_0_0_0_0_1.452	2	2	2	2	2	2	2	2	1	3	1	1	1
719	3	10,412,898	10,695,900	2	1	0_0_0_0_0_0_0_0_0_0_0_0_1.452	1	1	1	1	1	1	1	1	1	2	1	1	1
720	3	10,695,901	10,703,926	1	0														
721	3	10,703,927	11,935,468	2	1	1_1_1_1_1_0_0_0_0_1_1_1_0.121	2	2	2	2	2	2	2	2	1	1	1	1	1
722	3	11,935,469	12,018,429	3	2	0_0_0_0_0_0_0_0_0_0_0_0_1.940	2	2	2	2	2	2	2	2	1	3	1	1	1
723	3	12,018,430	12,073,955	3	3	0_0_0_0_0_0_0_0_0_0_0_0_1.940	2	2	2	2	2	2	2	2	1	3	1	1	1
724	3	12,073,956	12,142,955	3	2	0_0_0_0_0_0_0_0_0_0_0_0_1.940	2	2	2	2	2	2	2	2	1	3	1	1	1
725	3	12,142,956	12,257,483	2	1	0_0_0_0_0_0_0_0_0_0_0_0_1.940	1	1	1	1	1	1	1	1	1	2	1	1	1
726	3	12,257,484	12,443,971	3	2	0_0_0_0_0_0_0_0_0_0_0_0_1.940	2	2	2	2	2	2	2	2	1	3	1	1	1
727	3	12,443,972	13,122,402	2	1	0_0_0_0_0_0_0_0_0_0_0_0_1.940	1	1	1	1	1	1	1	1	1	2	1	1	1
728	3	13,122,403	13,228,578	3	2	0_0_0_0_0_0_0_0_0_0_0_0_1.940	2	2	2	2	2	2	2	2	1	3	1	1	1
729	3	13,228,579	13,858,232	2	1	1_1_1_1_1_0_0_0_0_1_1_1_0.303	2	2	2	2	2	2	2	2	1	1	1	1	1
730	3	13,858,233	14,490,262	1	0														
731	3	14,490,263	14,582,601	2	1	0_0_0_0_0_0_0_0_0_0_0_0_1.288	1	1	1	1	1	1	1	1	1	2	1	1	1
732	3	14,582,602	14,616,398	3	2	0_0_0_0_0_0_0_0_0_0_0_0_1.288	2	2	2	2	2	2	2	2	1	3	1	1	1
733	3	14,616,399	14,923,247	3	3	0_0_0_0_0_0_0_0_0_0_0_0_1.288	2	2	2	2	2	2	2	2	1	3	1	1	1
734	3	14,923,248	14,954,094	3	2	1_1_1_1_1_0_0_0_0_1_1_1_0.640	3	3	3	3	3	3	3	3	1	2	1	1	1
735	3	14,954,095	15,054,436	2	1	1_1_1_1_1_0_0_0_0_1_1_1_0.640	2	2	2	2	2	2	2	2	1	1	1	1	1
736	3	15,054,437	15,102,893	3	2	0_0_0_0_0_0_0_0_0_0_0_0_1.911	2	2	2	2	2	2	2	2	1	3	1	1	1
737	3	15,102,894	15,188,394	3	3	0_0_0_0_0_0_0_0_0_0_0_0_1.911	2	2	2	2	2	2	2	2	1	3	1	1	1
738	3	15,188,395	15,235,196	3	2	0_0_0_0_0_0_0_0_0_0_0_0_1.911	2	2	2	2	2	2	2	2	1	3	1	1	1
739	3	15,235,197	15,357,074	3	3	0_0_0_0_0_0_0_0_0_0_0_0_1.911	2	2	2	2	2	2	2	2	1	3	1	1	1
740	3	15,357,075	16,140,024	3	2	0_0_0_0_0_0_0_0_0_0_0_0_1.911	2	2	2	2	2	2	2	2	1	3	1	1	1
741	3	16,140,025	16,321,667	2	1	0_0_0_0_0_0_0_0_0_0_0_0_1.911	1	1	1	1	1	1	1	1	1	2	1	1	1
742	3	16,321,668	16,348,192	1	0														
743	3	16,348,193	16,727,346	2	1	1_1_1_1_1_0_1_0_0_1_1_1_0.708	2	2	2	2	2	2	2	2	2	1	1	1	1
744	3	16,727,347	17,097,174	1	0														
745	3	17,097,175	20,763,345	2	1	1_1_1_1_1_0_1_0_0_1_1_1_0.371	2	2	2	2	2	2	2	2	2	1	1	1	1
746	3	20,763,346	20,838,048	1	0														
747	3	20,838,049	20,839,432	2	1	0_0_0_0_0_0_0_0_0_0_0_0_1.206	1	1	1	1	1	1	1	1	1	2	1	1	1
748	3	20,839,433	21,088,071	3	2	0_0_0_0_0_0_0_0_0_0_0_0_1.206	2	2	2	2	2	2	2	2	1	3	1	1	1
749	3	21,088,072	21,108,103	3	3	0_0_0_0_0_0_0_0_0_0_0_0_1.206	2	2	2	2	2	2	2	2	1	3	1	1	1
750	3	21,108,104	21,110,959	3	2	1_1_1_1_1_0_0_0_0_1_1_1_0.156	3	3	3	3	3	3	3	3	1	2	1	1	1
751	3	21,110,960	23,867,181	2	1	1_1_1_1_1_0_0_0_0_1_1_1_1.119	2	2	2	2	2	2	2	2	1	2	1	1	1
752	3	23,867,182	23,869,512	1	0														
753	3	23,869,513	23,878,258	2	1	1_1_1_1_1_0_0_0_0_1_1_1_0.320	2	2	2	2	2	2	2	2	1	1	1	1	1
754	3	23,878,259	24,046,724	3	2	0_0_0_0_0_0_0_0_0_0_0_0_1.442	2	2	2	2	2	2	2	2	1	3	1	1	1
755	3	24,046,725	24,133,120	3	3	0_0_0_0_0_0_0_0_0_0_0_0_1.442	2	2	2	2	2	2	2	2	1	3	1	1	1
756	3	24,133,121	24,271,101	3	2	0_0_0_0_0_0_0_0_0_0_0_0_1.442	2	2	2	2	2	2	2	2	1	3	1	1	1
757	3	24,271,102	24,413,643	2	1	0_0_0_0_0_0_0_0_0_0_0_0_1.442	1	1	1	1	1	1	1	1	1	2	1	1	1
758	3	24,413,644	24,474,600	3	2	0_0_0_0_0_0_0_0_0_0_0_0_1.442	2	2	2	2	2	2	2	2	1	3	1	1	1

759	3	24,474,601	24,742,179	2	1	1_1_1_1_1_0_0_0_0_1_1_1_0.318	2	2	2	2	2	2	2	2	1	1	1	1	1
760	3	24,742,180	24,902,177	1	0														
761	3	24,902,178	25,913,973	2	1	1_1_1_1_1_0_0_0_0_1_1_1_0.131	2	2	2	2	2	2	2	2	1	1	1	1	1
762	3	25,913,974	27,567,189	1	0														
763	3	27,567,190	28,130,277	2	1	1_1_1_1_1_0_0_0_0_1_1_1_0.934	2	2	2	2	2	2	2	2	1	1	1	1	1
764	3	28,130,278	28,576,687	3	2	0_0_0_0_0_0_0_0_0_0_0_0_1.541	2	2	2	2	2	2	2	2	1	3	1	1	1
765	3	28,576,688	28,629,879	2	1	0_0_0_0_0_0_0_0_0_0_0_0_1.541	1	1	1	1	1	1	1	1	1	2	1	1	1
766	3	28,629,880	28,707,164	3	2	0_0_0_0_0_0_0_0_0_0_0_0_1.541	2	2	2	2	2	2	2	2	1	3	1	1	1
767	3	28,707,165	29,208,503	2	1	1_1_1_1_1_0_0_0_0_1_1_1_0.526	2	2	2	2	2	2	2	2	1	1	1	1	1
768	3	29,208,504	30,630,945	1	0														
769	3	30,630,946	32,878,546	2	1	1_1_1_1_1_0_0_0_0_1_1_1_0.198	2	2	2	2	2	2	2	2	1	1	1	1	1
770	3	32,878,547	33,563,875	1	0														
771	3	33,563,876	33,790,248	2	1	1_1_1_1_1_0_0_0_0_1_1_1_0.130	2	2	2	2	2	2	2	2	1	1	1	1	1
772	3	33,790,249	34,395,985	1	0														
773	3	34,395,986	34,996,202	2	1	0_0_0_0_0_0_0_1_0_0_0_0_1.767	1	1	1	1	1	1	1	1	1	2	1	2	1
774	3	34,996,203	35,030,702	3	2	0_0_0_0_0_0_0_1_0_0_0_0_1.767	2	2	2	2	2	2	2	2	1	3	1	3	1
775	3	35,030,703	35,109,888	2	1	0_0_0_0_0_0_0_1_0_0_0_0_1.767	1	1	1	1	1	1	1	1	1	2	1	2	1
776	3	35,109,889	35,111,575	1	0														
777	3	35,111,576	36,275,004	2	1	1_1_1_1_1_0_0_0_0_1_1_1_0.280	2	2	2	2	2	2	2	2	1	1	1	1	1
778	3	36,275,005	36,275,810	3	2	0_0_0_0_0_0_0_0_0_0_1_0_0.257	2	2	2	2	2	2	2	3	1	1	1	1	1
779	3	36,275,811	36,839,848	3	3	0_0_0_0_0_0_0_0_0_0_1_0_0.257	2	2	2	2	2	2	2	3	1	1	1	1	1
780	3	36,839,849	36,865,664	3	2	1_1_1_1_1_0_0_0_0_1_0_1_0.392	3	3	3	3	3	3	3	2	1	1	1	1	1
781	3	36,865,665	36,916,385	2	1	1_1_1_1_1_0_0_0_0_1_1_1_0.280	2	2	2	2	2	2	2	2	1	1	1	1	1
782	3	36,916,386	36,930,982	3	2	0_0_0_0_0_0_0_0_0_0_1_0_0.80	2	2	2	2	2	2	2	3	1	1	1	1	1
783	3	36,930,983	37,208,623	3	3	0_0_0_0_0_0_0_0_0_0_1_0_0.80	2	2	2	2	2	2	2	3	1	1	1	1	1
784	3	37,208,624	37,253,308	3	2	1_1_1_1_1_0_0_0_0_1_0_1_0.137	3	3	3	3	3	3	3	2	1	1	1	1	1
785	3	37,253,309	37,288,189	3	3	0_0_0_0_0_0_0_0_0_0_1_0_0.407	2	2	2	2	2	2	2	3	1	1	1	1	1
786	3	37,288,190	37,293,299	3	2	0_0_0_0_0_0_0_0_0_0_1_0_0.407	2	2	2	2	2	2	2	3	1	1	1	1	1
787	3	37,293,300	37,691,544	2	1	0_0_0_0_0_0_0_0_0_0_1_0_0.407	1	1	1	1	1	1	1	2	1	1	1	1	1
788	3	37,691,545	37,712,848	1	0														
789	3	37,712,849	38,055,385	2	1	1_1_1_1_1_0_0_0_0_1_1_1_0.424	2	2	2	2	2	2	2	2	1	1	1	1	1
790	3	38,055,386	38,057,020	1	0														
791	3	38,057,021	39,056,799	2	1	0_0_0_0_0_0_0_0_0_0_1_0_0.908	1	1	1	1	1	1	1	2	1	1	1	1	1
792	3	39,056,800	39,204,796	1	0														
793	3	39,204,797	39,427,586	2	1	0_0_0_0_0_0_0_0_0_0_1_0_0.150	1	1	1	1	1	1	1	2	1	1	1	1	1
794	3	39,427,587	40,439,565	1	0														
795	3	40,439,566	42,545,008	2	1	0_0_0_0_0_0_0_0_0_0_1_0_0.136	1	1	1	1	1	1	1	2	1	1	1	1	1
796	3	42,545,009	42,619,021	1	0														
797	3	42,619,022	43,076,543	2	1	0_0_0_0_0_0_0_0_0_0_1_0_0.76	1	1	1	1	1	1	1	2	1	1	1	1	1
798	3	43,076,544	43,077,390	3	2	0_0_0_0_0_0_0_0_0_0_1_0_0.76	1	1	1	1	1	1	1	3	1	2	1	2	1
799	3	43,077,391	43,114,298	3	3	0_0_0_0_0_0_0_0_0_0_1_0_0.76	1	1	1	1	1	1	1	3	1	2	1	2	1
800	3	43,114,299	43,148,661	3	2	0_0_0_0_0_0_0_1_0_0_0_0_1.143	2	2	2	2	2	2	2	1	2	3	2	3	2
801	3	43,148,662	43,279,208	2	1	0_0_0_0_0_0_0_1_0_0_0_0_1.143	1	1	1	1	1	1	1	1	1	2	1	2	1
802	3	43,279,209	43,279,292	3	2	0_0_0_0_0_0_0_1_0_0_0_0_1.143	2	2	2	2	2	2	2	1	2	3	2	3	2
803	3	43,279,293	43,448,322	2	1	1_1_1_1_1_1_1_0_1_1_0_1_0.564	2	2	2	2	2	2	2	1	2	1	2	1	2
804	3	43,448,323	43,521,171	3	2	0_0_0_0_0_0_0_1_0_0_0_0_1.41	2	2	2	2	2	2	2	1	2	3	2	3	2
805	3	43,521,172	43,622,639	2	1	1_1_1_1_1_1_1_0_1_1_0_1_0.564	2	2	2	2	2	2	2	1	2	1	2	1	2
806	3	43,622,640	43,622,691	3	2	0_0_0_0_0_0_0_0_0_0_1_0_0.359	2	2	2	2	2	2	2	3	2	1	2	1	2
807	3	43,622,692	44,412,565	3	3	0_0_0_0_0_0_0_0_0_0_1_0_0.359	1	1	1	1	1	1	1	3	1	2	1	2	1
808	3	44,412,566	44,412,845	4	4	0_0_0_0_0_0_0_0_0_0_1_0_0.359	2	2	2	2	2	2	2	4	1	3	1	3	1
809	3	44,412,846	44,413,424	4	5	0_0_0_0_0_0_0_0_0_0_1_0_0.359	1	1	1	1	1	1	1	4	2	3	2	3	2
810	3	44,413,425	44,463,818	4	6	0_0_0_0_0_0_0_0_0_0_1_0_0.359	1	1	1	1	1	1	1	4	2	3	2	3	2
811	3	44,463,819	44,515,888	4	5	0_0_0_0_0_0_0_0_0_0_1_0_0.359	2	2	2	2	2	2	2	4	1	3	1	3	1
812	3	44,515,889	44,738,614	4	4	0_0_0_0_0_0_0_1_0_0_0_0_1.417	3	3	3	3	3	3	3	2	1	4	1	4	1
813	3	44,738,615	44,738,718	4	3	0_0_0_0_0_0_0_1_0_0_0_0_1.417	3	3	3	3	3	3	3	2	1	4	1	4	1
814	3	44,738,719	44,739,804	3	2	1_1_1_1_1_0_0_0_0_1_0_1_0.64	3	3	3	3	3	3	3	2	1	1	1	1	1
815	3	44,739,805	44,806,205	2	1	1_1_1_1_1_0_0_0_0_1_1_1_0.210	2	2	2	2	2	2	2	2	1	1	1	1	1
816	3	44,806,206	44,957,606	3	2	1_1_1_1_1_0_0_0_0_1_0_1_0.48	3	3	3	3	3	3	3	2	1	1	1	1	1
817	3	44,957,607	45,065,204	2	1	1_1_1_1_1_0_0_0_0_1_1_1_0.210	2	2	2	2	2	2	2	2	1	1	1	1	1
818	3	45,065,205	45,065,600	3	2	1_1_1_1_1_0_0_0_0_1_0_1_0.422	3	3	3	3	3	3	3	2	1	1	1	1	1
819	3	45,065,601	45,733,372	3	3	0_0_0_0_0_0_0_0_0_0_1_0_0.244	2	2	2	2	2	2	2	3	1	1	1	1	1
820	3	45,733,373	45,734,102	3	2	0_0_0_0_0_0_0_0_0_0_1_0_0.244	2	2	2	2	2	2	2	3	1	1	1	1	1
821	3	45,734,103	46,215,377	2	1	1_1_1_1_1_0_0_0_0_1_1_1_0.210	2	2	2	2	2	2	2	2	1	1	1	1	1
822	3	46,215,378	46,440,513	3	2	0_0_0_0_0_0_0_1_0_0_0_0_1.60	2	2	2	2	2	2	2	2	1	3	1	3	1
823	3	46,440,514	47,288,932	2	1	1_1_1_1_1_0_0_0_0_1_1_1_0.210	2	2	2	2	2	2	2	2	1	1	1	1	1
824	3	47,288,933	47,413,601	3	2	0_0_0_0_0_0_0_1_0_0_0_0_1.420	2	2	2	2	2	2	2	2	1	3	1	3	1
825	3	47,413,602	47,554,126	2	1	0_0_0_0_0_0_0_1_0_0_0_0_1.420	1	1	1	1	1	1	1	1	1	2	1	2	1
826	3	47,554,127	48,324,863	3	2	0_0_0_0_0_0_0_1_0_0_0_0_1.420	2	2	2	2	2	2	2	2	1	3	1	3	1
827	3	48,324,864	48,484,065	3	3	0_0_0_0_0_0_0_1_0_0_0_0_1.420	2	2	2	2	2	2	2	2	1	3	1	3	1
828	3	48,484,066	48,503,080	3	2	0_0_0_0_0_0_0_1_0_0_0_0_1.420	2	2	2	2	2	2	2	2	1	3	1	3	1
829	3	48,503,081	49,321,629	2	1	1_1_1_1_1_0_0_0_0_1_1_1_0.144	2	2	2	2	2	2	2	2	1	1	1	1	1
830	3	49,321,630	49,535,372	1	0														
831	3	49,535,373	50,208,980	2	1	1_1_1_1_1_0_0_0_0_1_1_1_0.557	2	2	2	2	2	2	2	2	1	1	1	1	1
832	3	50,208,981	50,305,760	1	0														
833	3	50,305,761	51,895,694	2	1	1_1_1_1_1_0_0_0_0_1_1_1_0.171	2	2	2	2	2	2	2	2	1	1	1	1	1
834	3	51,895,695	51,967,342	1	0														
835	3	51,967,343	52,942,507	2	1	1_1_1_1_1_0_0_0_0_1_1_1_0.149	2	2	2	2	2	2	2	2	1	1	1	1	1
836	3	52,942,508	53,225,284	1	0														
837	3	53,225,285	55,843,126	2	1	1_1_1_1_1_0_0_0_0_1_1_1_0.270	2	2	2	2	2	2	2	2	1	1	1	1	1
838	3	55,843,127	56,664,409	1	0														
839	3	56,664,410	56,795,723	2	1	1_1_1_1_1_0_1_0_0_1_1_1_0.175	2	2	2	2	2	2	2	2	2	1	1	1	1
840	3	56,795,724	56,876,806	1	0														
841	3	56,876,807	58,388,847	2	1	1_1_1_1_1_0_1_0_0_1_1_1_0.654	2	2	2	2	2	2	2	2	2	1	1	1	1
842	3	58,388,848	60,268,819	1	0														
843	3	60,268,820	60,385,073	2	1	0_0_0_0_0_0_0_0_0_0_0_0_1.102	1	1	1	1	1	1	1	1	1	2	1	1	1

Disease Models & Mechanisms • Supplementary information

844	3	60,385,074	61,035,043	1	0														
845	3	61,035,044	61,744,390	2	1	1_1_1_1_1_0_1_0_0_1_1_1_1.348	2	2	2	2	2	2	2	2	2	2	1	1	1
846	3	61,744,391	61,750,147	1	0														
847	3	61,750,148	61,751,300	2	1	1_1_1_1_1_0_1_0_0_1_1_1_0.22	2	2	2	2	2	2	2	2	2	1	1	1	1
848	3	61,751,301	61,839,643	3	2	0_0_0_0_0_0_0_0_0_0_0_0_1.336	2	2	2	2	2	2	2	2	2	3	1	1	1
849	3	61,839,644	65,064,567	2	1	0_0_0_0_0_0_0_0_0_0_0_0_1.336	1	1	1	1	1	1	1	1	1	2	1	1	1
850	3	65,064,568	65,143,694	3	2	0_0_0_0_0_0_0_0_0_0_0_0_1.336	2	2	2	2	2	2	2	2	2	3	1	1	1
851	3	65,143,695	65,235,551	2	1	1_1_1_1_1_0_1_0_0_1_1_1_0.128	2	2	2	2	2	2	2	2	2	1	1	1	1
852	3	65,235,552	65,563,098	3	2	0_0_0_0_0_0_0_0_0_0_0_0_1.165	2	2	2	2	2	2	2	2	2	3	1	1	1
853	3	65,563,099	66,411,747	2	1	1_1_1_1_1_0_1_0_0_1_1_1_0.128	2	2	2	2	2	2	2	2	2	1	1	1	1
854	3	66,411,748	66,469,850	1	0														
855	3	66,469,851	76,466,778	2	1	1_1_1_1_1_0_1_0_0_1_1_1_0.103	2	2	2	2	2	2	2	2	2	1	1	1	1
856	3	76,466,779	76,562,767	1	0														
857	3	76,562,768	92,039,714	2	1	1_1_1_1_1_0_1_0_0_1_1_1_0.276	2	2	2	2	2	2	2	2	2	1	1	1	1
858	3	92,039,715	92,130,071	3	2	1_1_1_1_1_0_0_0_0_1_1_1_0.48	3	3	3	3	3	3	3	3	2	1	1	1	1
859	3	92,130,072	95,067,536	2	1	1_1_1_1_1_0_1_0_0_1_1_1_0.276	2	2	2	2	2	2	2	2	2	1	1	1	1
860	3	95,067,537	95,154,349	3	2	0_0_0_0_0_0_0_0_0_0_0_0_1.40	2	2	2	2	2	2	2	2	2	3	1	1	1
861	3	95,154,350	104,248,354	2	1	1_1_1_1_1_0_1_0_0_1_1_1_0.276	2	2	2	2	2	2	2	2	2	1	1	1	1
862	3	104,248,355	105,420,419	1	0														
863	3	105,420,420	108,724,002	2	1	1_1_1_1_1_0_1_0_0_1_1_1_0.252	2	2	2	2	2	2	2	2	2	1	1	1	1
864	3	108,724,003	108,726,051	1	0														
865	3	108,726,052	108,770,946	2	1	1_1_1_1_1_0_1_0_0_1_0_1_0.70	2	2	2	2	2	2	2	1	2	1	1	1	1
866	3	108,770,947	113,535,147	1	0														
867	3	113,535,148	116,538,474	2	1	0_0_0_0_0_0_0_0_0_0_0_0_1.239	1	1	1	1	1	1	1	1	1	2	1	1	1
868	3	116,538,475	116,539,221	1	0														
869	3	116,539,222	117,295,623	2	1	1_1_1_1_1_0_1_0_0_1_1_1_0.849	2	2	2	2	2	2	2	2	2	1	1	1	1
870	3	117,295,624	117,470,361	1	0														
871	3	117,470,362	118,664,981	2	1	1_1_1_1_1_0_1_0_0_1_1_1_0.716	2	2	2	2	2	2	2	2	2	1	1	1	1
872	3	118,664,982	118,668,992	1	0														
873	3	118,668,993	118,832,838	2	1	0_0_0_0_0_0_0_0_0_0_0_0_1.206	1	1	1	1	1	1	1	1	1	2	1	1	1
874	3	118,832,839	118,850,948	1	0														
875	3	118,850,949	120,119,276	2	1	1_1_1_1_1_0_1_0_0_1_1_1_0.117	2	2	2	2	2	2	2	2	2	1	1	1	1
876	3	120,119,277	120,232,787	1	0														
877	3	120,232,788	122,877,899	2	1	1_1_1_1_1_0_1_0_0_1_1_1_0.272	2	2	2	2	2	2	2	2	2	1	1	1	1
878	3	122,877,900	122,880,625	1	0														
879	3	122,880,626	123,592,972	2	1	0_0_0_0_0_0_0_0_0_0_1_0_0.146	1	1	1	1	1	1	1	2	1	1	1	1	1
880	3	123,592,973	123,593,376	3	2	0_0_0_0_0_0_0_0_0_0_1_0_0.146	2	2	2	2	2	2	2	3	2	1	1	1	1
881	3	123,593,377	124,349,897	3	3	0_0_0_0_0_0_0_0_0_0_1_0_0.146	2	2	2	2	2	2	2	3	2	1	1	1	1
882	3	124,349,898	124,408,497	3	2	0_0_0_0_0_0_0_0_0_0_1_0_0.146	2	2	2	2	2	2	2	3	2	1	1	1	1
883	3	124,408,498	125,480,088	3	3	0_0_0_0_0_0_0_0_0_0_1_0_0.146	2	2	2	2	2	2	2	3	2	1	1	1	1
884	3	125,480,089	125,513,760	3	2	0_0_0_0_0_0_0_0_0_0_1_0_0.146	2	2	2	2	2	2	2	3	2	1	1	1	1
885	3	125,513,761	125,856,450	3	3	0_0_0_0_0_0_0_0_0_0_1_0_0.146	2	2	2	2	2	2	2	3	2	1	1	1	1
886	3	125,856,451	125,856,578	3	2	1_1_1_1_1_0_1_0_0_1_0_1_0.171	3	3	3	3	3	3	3	2	3	1	1	1	1
887	3	125,856,579	125,899,040	2	1	1_1_1_1_1_0_1_0_0_1_0_1_0.171	2	2	2	2	2	2	2	1	2	1	1	1	1
888	3	125,899,041	125,902,476	3	2	0_0_0_0_0_0_0_1_0_0_1_0_0.650	2	2	2	2	2	2	2	3	2	1	1	3	1
889	3	125,902,477	126,519,413	3	3	0_0_0_0_0_0_0_1_0_0_1_0_0.650	1	1	1	1	1	1	1	3	1	2	2	3	2
890	3	126,519,414	126,523,065	3	2	0_0_0_0_0_0_0_1_0_0_1_0_0.650	1	1	1	1	1	1	1	3	1	2	2	3	2
891	3	126,523,066	126,552,616	2	1	0_0_0_0_0_0_0_1_0_0_1_0_0.650	1	1	1	1	1	1	1	2	1	1	1	2	1
892	3	126,552,617	126,635,509	3	2	0_0_0_0_0_0_0_1_0_0_1_0_0.650	2	2	2	2	2	2	2	3	2	1	1	3	1
893	3	126,635,510	126,936,992	3	3	0_0_0_0_0_0_0_1_0_0_1_0_0.650	1	1	1	1	1	1	1	3	1	2	2	3	2
894	3	126,936,993	127,039,820	3	2	0_0_0_0_0_0_0_1_0_0_1_0_0.650	2	2	2	2	2	2	2	3	2	1	1	3	1
895	3	127,039,821	127,045,981	2	1	0_0_0_0_0_0_0_1_0_0_1_0_0.650	1	1	1	1	1	1	1	2	1	1	1	2	1
896	3	127,045,982	127,047,325	1	0														
897	3	127,047,326	127,119,171	2	1	1_1_1_1_1_0_0_0_0_1_1_1_1.108	2	2	2	2	2	2	2	2	1	2	1	1	1
898	3	127,119,172	127,121,530	1	0														
899	3	127,121,531	127,126,521	2	1	1_1_1_1_1_0_1_0_0_1_0_1_0.368	2	2	2	2	2	2	2	1	2	1	1	1	1
900	3	127,126,522	127,127,199	3	2	0_0_0_0_0_0_0_1_0_0_1_0_0.416	2	2	2	2	2	2	2	3	2	1	1	3	1
901	3	127,127,200	127,754,595	3	3	0_0_0_0_0_0_0_1_0_0_1_0_0.416	1	1	1	1	1	1	1	3	1	2	2	3	2
902	3	127,754,596	127,757,862	3	2	0_0_0_0_0_0_0_1_0_0_1_0_0.416	1	1	1	1	1	1	1	3	1	2	2	3	2
903	3	127,757,863	127,794,925	2	1	0_0_0_0_0_1_0_0_1_0_0_0_1.116	1	1	1	1	1	1	1	1	1	2	2	1	2
904	3	127,794,926	127,801,351	3	2	0_0_0_0_0_1_0_0_1_0_0_0_1.116	2	2	2	2	2	2	2	1	2	3	3	1	3
905	3	127,801,352	128,074,300	3	3	0_0_0_0_0_0_0_1_0_0_1_0_0.129	1	1	1	1	1	1	1	3	1	2	2	3	2
906	3	128,074,301	128,074,379	3	2	0_0_0_0_0_1_0_0_1_0_0_0_1.116	2	2	2	2	2	2	2	1	2	3	3	1	3
907	3	128,074,380	128,187,840	2	1	0_0_0_0_0_1_0_0_1_0_0_0_1.116	1	1	1	1	1	1	1	1	1	2	2	1	2
908	3	128,187,841	128,188,192	3	2	0_0_0_0_0_1_0_0_1_0_0_0_1.116	2	2	2	2	2	2	2	1	2	3	3	1	3
909	3	128,188,193	128,929,510	3	3	0_0_0_0_0_0_0_1_0_0_1_0_0.416	1	1	1	1	1	1	1	3	1	2	2	3	2
910	3	128,929,511	128,931,242	3	2	0_0_0_0_0_0_0_1_0_0_1_0_0.416	1	1	1	1	1	1	1	3	1	2	2	3	2
911	3	128,931,243	128,935,511	2	1	0_0_0_0_0_0_0_1_0_0_1_0_0.416	1	1	1	1	1	1	1	2	1	1	1	2	1
912	3	128,935,512	129,029,617	1	0														
913	3	129,029,618	129,677,630	2	1	0_1_0_0_0_0_0_1_0_1_1_1_0.106	2	2	2	1	1	1	1	2	1	1	1	2	1
914	3	129,677,631	129,682,942	1	0														
915	3	129,682,943	129,893,415	2	1	0_1_0_0_0_0_0_0_0_1_1_1_0.75	2	2	2	1	1	1	1	2	1	1	1	1	1
916	3	129,893,416	130,045,827	1	0														
917	3	130,045,828	130,467,178	2	1	0_1_0_0_0_0_0_0_0_1_1_1_0.258	2	2	2	1	1	1	1	2	1	1	1	1	1
918	3	130,467,179	130,468,265	1	0														
919	3	130,468,266	134,599,119	2	1	0_1_0_0_0_0_0_0_0_1_1_1_1.167	2	2	2	1	1	1	1	2	1	2	1	1	1
920	3	134,599,120	134,830,553	3	2	0_1_0_0_0_0_0_0_0_1_1_1_1.167	3	3	3	2	2	2	2	3	2	3	1	1	1
921	3	134,830,554	135,759,020	2	1	1_1_1_1_1_0_1_0_0_1_1_1_1.643	2	2	2	2	2	2	2	2	2	2	1	1	1
922	3	135,759,021	135,948,024	1	0														
923	3	135,948,025	136,431,485	2	1	1_1_1_1_1_0_1_0_0_1_1_1_1.237	2	2	2	2	2	2	2	2	2	2	1	1	1
924	3	136,431,486	136,464,923	3	2	0_1_0_0_0_0_0_0_0_1_1_1_1.44	3	3	3	2	2	2	2	3	2	3	1	1	1
925	3	136,464,924	136,546,911	3	3	0_1_0_0_0_0_0_0_0_1_1_1_1.44	3	3	3	1	1	1	1	3	1	3	2	2	2
926	3	136,546,912	136,580,191	3	2	0_1_0_0_0_0_0_0_0_1_1_1_1.44	3	3	3	2	2	2	2	3	2	3	1	1	1
927	3	136,580,192	136,580,299	2	1	1_1_1_1_1_0_1_0_0_1_1_1_1.237	2	2	2	2	2	2	2	2	2	2	1	1	1
928	3	136,580,300	136,583,320	1	0														

Disease Models & Mechanisms • Supplementary information

929	3	136,583,321	137,009,967	2	1	0_1_0_0_0_0_0_0_0_1_1_1_0.737	2	2	2	1	1	1	1	2	1	1	1	1	1
930	3	137,009,968	137,275,877	3	2	0_1_0_0_0_0_0_0_0_1_1_1_0.737	3	3	3	2	2	2	2	3	2	1	1	1	1
931	3	137,275,878	137,570,685	3	3	0_1_0_0_0_0_0_0_0_1_1_1_0.737	3	3	3	1	1	1	1	3	1	2	2	2	2
932	3	137,570,686	137,573,863	3	2	0_1_0_0_0_0_0_0_0_1_1_1_0.737	3	3	3	2	2	2	2	3	2	1	1	1	1
933	3	137,573,864	141,785,247	2	1	1_1_1_1_1_0_1_0_0_1_1_1_0.407	2	2	2	2	2	2	2	2	2	1	1	1	1
934	3	141,785,248	141,786,668	1	0														
935	3	141,786,669	141,959,345	2	1	1_1_1_1_1_0_1_0_0_1_1_1_1.155	2	2	2	2	2	2	2	2	2	2	1	1	1
936	3	141,959,346	141,961,575	1	0														
937	3	141,961,576	142,561,780	2	1	1_1_1_1_1_0_1_0_0_1_1_1_0.365	2	2	2	2	2	2	2	2	2	1	1	1	1
938	3	142,561,781	142,632,943	3	2	0_1_0_0_0_1_0_1_1_1_1_1_1.77	3	3	3	1	1	1	1	3	1	2	2	2	2
939	3	142,632,944	142,647,474	2	1	0_1_0_0_0_1_0_1_1_1_1_1_1.77	2	2	2	1	1	1	1	2	1	2	2	2	2
940	3	142,647,475	142,668,563	3	2	0_1_0_0_0_1_0_1_1_1_1_1_1.77	2	2	2	1	1	1	1	2	1	3	2	2	2
941	3	142,668,564	142,815,795	3	3	0_0_0_0_0_0_0_0_0_0_0_0_1.122	2	2	2	1	1	1	1	2	1	3	2	2	2
942	3	142,815,796	145,175,910	3	2	0_0_0_0_0_0_0_0_0_0_0_0_1.122	1	1	1	2	2	2	2	1	2	3	1	1	1
943	3	145,175,911	145,808,093	3	3	0_0_0_0_0_0_0_0_0_0_0_0_1.122	2	2	2	1	1	1	1	2	1	3	2	2	2
944	3	145,808,094	146,550,287	3	2	0_0_0_0_0_0_0_0_0_0_0_0_1.122	1	1	1	2	2	2	2	1	2	3	1	1	1
945	3	146,550,288	146,819,312	3	3	0_0_0_0_0_0_0_0_0_0_0_0_1.122	2	2	2	1	1	1	1	2	1	3	2	2	2
946	3	146,819,313	146,827,766	3	2	0_1_0_0_0_1_0_1_1_1_1_1_1.111	2	2	2	1	1	1	1	2	1	3	2	2	2
947	3	146,827,767	147,105,059	2	1	0_1_0_0_0_1_0_1_1_1_1_1_1.111	2	2	2	1	1	1	1	2	1	2	2	2	2
948	3	147,105,060	147,107,477	1	0														
949	3	147,107,478	147,122,184	2	1	0_0_0_0_0_0_0_0_0_0_0_0_1.330	1	1	1	1	1	1	1	1	1	2	1	1	1
950	3	147,122,185	147,282,746	3	2	0_0_0_0_0_0_0_0_0_0_0_0_1.330	1	1	1	2	2	2	2	1	2	3	1	1	1
951	3	147,282,747	147,329,740	2	1	0_0_0_0_0_0_0_0_0_0_0_0_1.330	1	1	1	1	1	1	1	1	1	2	1	1	1
952	3	147,329,741	147,371,495	1	0														
953	3	147,371,496	149,070,334	2	1	1_0_1_1_1_0_1_0_0_0_0_0_1.173	1	1	1	2	2	2	2	1	2	2	1	1	1
954	3	149,070,335	149,338,591	1	0														
955	3	149,338,592	149,645,487	2	1	1_0_1_1_1_0_1_0_0_0_0_0_1.108	1	1	1	2	2	2	2	1	2	2	1	1	1
956	3	149,645,488	149,952,453	1	0														
957	3	149,952,454	150,085,084	2	1	0_1_0_0_0_0_0_0_0_1_0_1_0.101	2	2	2	1	1	1	1	1	1	1	1	1	1
958	3	150,085,085	150,099,254	3	2	0_1_0_0_0_0_0_0_0_1_0_1_0.101	3	3	3	2	2	2	2	1	2	2	1	1	1
959	3	150,099,255	150,245,625	2	1	0_1_0_0_0_0_0_0_0_1_0_1_0.101	2	2	2	1	1	1	1	1	1	1	1	1	1
960	3	150,245,626	151,747,625	1	0														
961	3	151,747,626	151,793,010	2	1	0_1_0_0_0_0_0_1_0_1_0_1_0.54	2	2	2	1	1	1	1	1	1	1	1	2	1
962	3	151,793,011	151,878,556	1	0														
963	3	151,878,557	152,117,912	2	1	0_1_0_0_0_0_0_1_0_1_0_1_0.288	2	2	2	1	1	1	1	1	1	1	1	2	1
964	3	152,117,913	152,523,617	1	0														
965	3	152,523,618	152,524,576	2	1	1_1_1_1_1_0_1_0_0_1_0_1_1.626	2	2	2	2	2	2	2	1	2	2	1	1	1
966	3	152,524,577	152,525,008	3	2	0_1_0_0_0_0_0_0_0_1_0_1_0.621	3	3	3	2	2	2	2	1	2	2	1	1	1
967	3	152,525,009	153,593,056	3	3	0_1_0_0_0_0_0_0_0_1_0_1_0.621	3	3	3	2	2	2	2	1	2	2	1	1	1
968	3	153,593,057	153,595,234	3	2	0_1_0_0_0_0_0_0_0_1_0_1_0.621	3	3	3	2	2	2	2	1	2	2	1	1	1
969	3	153,595,235	153,657,944	2	1	1_0_1_1_1_0_1_0_0_0_0_0_1.513	1	1	1	2	2	2	2	1	2	2	1	1	1
970	3	153,657,945	153,670,596	1	0														
971	3	153,670,597	153,710,897	2	1	0_1_0_0_0_0_0_1_0_1_0_1_0.63	2	2	2	1	1	1	1	1	1	1	1	2	1
972	3	153,710,898	153,807,061	1	0														
973	3	153,807,062	153,829,972	2	1	0_1_0_0_0_0_0_1_0_1_0_1_0.26	2	2	2	1	1	1	1	1	1	1	1	2	1
974	3	153,829,973	153,966,135	1	0														
975	3	153,966,136	153,975,901	2	1	1_0_1_1_1_0_1_0_0_0_0_0_1.165	1	1	1	2	2	2	2	1	2	2	1	1	1
976	3	153,975,902	154,015,384	3	2	0_1_0_0_0_0_0_0_0_1_0_1_0.123	3	3	3	2	2	2	2	1	2	2	1	1	1
977	3	154,015,385	154,430,003	3	3	0_1_0_0_0_0_0_0_0_1_0_1_0.123	3	3	3	2	2	2	2	1	2	2	1	1	1
978	3	154,430,004	154,430,554	3	2	0_1_0_0_0_0_0_0_0_1_0_1_0.123	3	3	3	2	2	2	2	1	2	2	1	1	1
979	3	154,430,555	154,437,548	2	1	0_1_0_0_0_0_0_0_0_1_0_1_0.123	2	2	2	1	1	1	1	1	1	1	1	1	1
980	3	154,437,549	154,579,965	3	2	0_1_0_0_0_0_0_0_0_1_0_1_0.123	3	3	3	2	2	2	2	1	2	1	1	1	1
981	3	154,579,966	155,401,477	2	1	0_1_0_0_0_0_0_0_0_1_0_1_0.123	2	2	2	1	1	1	1	1	1	1	1	1	1
982	3	155,401,478	155,439,796	3	2	0_1_0_0_0_0_0_0_0_1_0_1_0.123	3	3	3	1	1	1	1	1	1	1	1	2	1
983	3	155,439,797	155,987,891	2	1	0_1_0_0_0_0_0_1_0_1_0_1_0.593	2	2	2	1	1	1	1	1	1	1	1	2	1
984	3	155,987,892	155,988,535	1	0														
985	3	155,988,536	156,410,572	2	1	0_1_0_0_0_0_0_1_0_1_1_1_0.463	2	2	2	1	1	1	1	2	1	1	1	2	1
986	3	156,410,573	156,410,605	3	2	0_1_0_0_0_0_0_0_0_1_1_1_0.647	3	3	3	1	1	1	1	3	1	1	1	2	1
987	3	156,410,606	156,585,005	3	3	0_1_0_0_0_0_0_0_0_1_1_1_0.647	3	3	3	1	1	1	1	3	1	1	1	2	1
988	3	156,585,006	156,599,252	3	2	0_1_0_0_0_0_0_0_0_1_1_1_0.647	3	3	3	2	2	2	2	3	2	2	2	1	2
989	3	156,599,253	157,106,213	2	1	0_1_0_0_0_0_0_0_0_1_1_1_0.647	2	2	2	1	1	1	1	2	1	1	1	1	1
990	3	157,106,214	157,106,696	1	0														
991	3	157,106,697	157,601,574	2	1	1_1_1_1_1_0_1_0_0_1_1_1_0.519	2	2	2	2	2	2	2	2	2	1	1	1	1
992	3	157,601,575	157,714,396	1	0														
993	3	157,714,397	157,858,920	2	1	0_0_0_0_0_1_0_0_1_0_0_0_1.820	1	1	1	1	1	1	1	1	1	2	2	1	2
994	3	157,858,921	157,875,076	3	2	0_0_0_0_0_1_0_0_1_0_0_0_1.820	2	2	2	2	2	2	2	2	2	3	3	1	3
995	3	157,875,077	158,096,518	3	3	0_0_0_0_0_1_0_0_1_0_0_0_1.820	2	2	2	2	2	2	2	2	2	3	3	1	3
996	3	158,096,519	158,167,421	3	2	0_0_0_0_0_1_0_0_1_0_0_0_1.820	2	2	2	2	2	2	2	2	2	3	3	1	3
997	3	158,167,422	158,584,235	3	3	0_0_0_0_0_1_0_0_1_0_0_0_1.820	2	2	2	2	2	2	2	2	2	3	3	1	3
998	3	158,584,236	158,587,250	3	2	1_1_1_1_1_0_1_0_0_1_1_1_0.382	3	3	3	3	3	3	3	3	3	2	2	1	2
999	3	158,587,251	158,630,358	2	1	1_1_1_1_1_1_1_0_1_1_1_1_1.353	2	2	2	2	2	2	2	2	2	2	2	1	2
1000	3	158,630,359	158,645,358	3	2	0_0_1_0_0_1_0_1_1_0_0_0_1.119	1	1	1	1	3	1	1	1	1	3	3	2	3
1001	3	158,645,359	158,736,380	2	1	0_0_1_0_0_1_0_1_1_0_0_0_1.119	1	1	1	1	2	1	1	1	1	2	2	2	2
1002	3	158,736,381	159,072,235	3	2	0_0_1_0_0_1_0_1_1_0_0_0_1.119	1	1	1	1	2	1	1	1	1	2	2	3	2
1003	3	159,072,236	159,509,382	2	1	1_1_0_1_1_0_1_1_0_1_1_1_0.613	2	2	2	2	1	2	2	2	2	1	1	2	1
1004	3	159,509,383	159,967,102	1	0														
1005	3	159,967,103	160,634,219	2	1	0_0_1_0_0_1_0_1_1_0_0_0_1.755	1	1	1	1	2	1	1	1	1	2	2	2	2
1006	3	160,634,220	161,762,646	1	0														
1007	3	161,762,647	162,093,353	2	1	0_0_1_0_0_1_0_1_1_0_1_0_1.246	1	1	1	1	2	1	1	2	1	2	2	2	2
1008	3	162,093,354	164,887,040	1	0														
1009	3	164,887,041	164,897,549	2	1	1_1_1_1_1_0_1_0_0_1_1_1_0.30	2	2	2	2	2	2	2	2	2	1	1	1	1
1010	3	164,897,550	166,058,398	1	0														
1011	3	166,058,399	166,466,449	2	1	1_1_0_1_1_1_1_0_1_1_1_1_1.202	2	2	2	2	1	2	2	2	2	2	2	1	2
1012	3	166,466,450	167,175,170	1	0														
1013	3	167,175,171	167,863,740	2	1	0_0_0_0_0_0_0_0_0_0_1_0_1.81	1	1	1	1	1	1	1	2	1	2	1	1	1

1014	3	167,863,741	167,909,227	1	0													
1015	3	167,909,228	167,998,980	2	1	1_1_1_1_1_1_1_0_1_1_1_1_1.43	2	2	2	2	2	2	2	2	2	2	1	2
1016	3	167,998,981	170,143,872	1	0													
1017	3	170,143,873	171,176,709	2	1	1_1_1_1_1_0_1_0_0_1_1_1_1.720	2	2	2	2	2	2	2	2	2	2	1	1	1
1018	3	171,176,710	171,177,758	1	0													
1019	3	171,177,759	174,582,925	2	1	0_0_0_0_0_0_0_0_0_0_1_0_1.213	1	1	1	1	1	1	1	2	1	2	1	1	1
1020	3	174,582,926	175,403,344	1	0													
1021	3	175,403,345	175,476,470	2	1	1_1_1_1_1_0_1_0_0_1_1_1_0.751	2	2	2	2	2	2	2	2	2	1	1	1	1
1022	3	175,476,471	175,482,726	3	2	0_0_0_0_0_0_0_0_0_0_0_0_1.507	2	2	2	2	2	2	2	2	2	3	1	1	1
1023	3	175,482,727	177,387,531	3	3	0_0_0_0_0_0_0_0_0_0_0_0_1.507	2	2	2	2	2	2	2	2	2	3	1	1	1
1024	3	177,387,532	177,388,236	3	2	0_0_0_0_0_0_0_0_0_0_0_0_1.507	2	2	2	2	2	2	2	2	2	3	1	1	1
1025	3	177,388,237	177,388,667	2	1	0_0_0_0_0_0_0_0_0_0_0_0_1.507	1	1	1	1	1	1	1	1	1	2	1	1	1
1026	3	177,388,668	177,531,383	1	0													
1027	3	177,531,384	177,698,927	2	1	0_0_0_0_0_0_0_0_0_0_1_0_1.82	1	1	1	1	1	1	1	2	1	2	1	1	1
1028	4	254,424	708,852	2	1	1_0_1_1_1_0_0_0_0_1_1_1_0.51	1	2	2	2	2	2	2	2	1	1	1	1	1
1029	4	708,853	731,557	1	0													
1030	4	731,558	3,454,336	2	1	1_0_1_1_1_0_1_0_0_1_1_1_0.772	1	2	2	2	2	2	2	2	2	1	1	1	1
1031	4	3,454,337	4,065,957	1	0													
1032	4	4,065,958	4,482,421	2	1	1_0_1_1_1_0_0_0_0_1_1_1_0.593	1	2	2	2	2	2	2	2	1	1	1	1	1
1033	4	4,482,422	4,597,522	1	0													
1034	4	4,597,523	5,265,936	2	1	1_0_1_1_1_1_1_1_1_1_1_1_1.582	1	2	2	2	2	2	2	2	2	2	2	2	2
1035	4	5,265,937	5,270,410	1	0													
1036	4	5,270,411	7,433,495	2	1	1_0_1_1_1_0_0_0_0_1_1_1_1.118	1	2	2	2	2	2	2	2	1	2	1	1	1
1037	4	7,433,496	7,518,220	1	0													
1038	4	7,518,221	8,719,215	2	1	1_1_1_1_1_0_0_0_0_1_1_1_1.567	2	2	2	2	2	2	2	2	1	2	1	1	1
1039	4	8,719,216	8,720,150	1	0													
1040	4	8,720,151	9,023,148	2	1	1_1_1_1_1_0_0_0_0_1_1_1_0.308	2	2	2	2	2	2	2	2	1	1	1	1	1
1041	4	9,023,149	9,023,691	1	0													
1042	4	9,023,692	9,690,928	2	1	0_0_0_0_0_0_0_0_0_0_0_0_1.788	1	1	1	1	1	1	1	1	1	2	1	1	1
1043	4	9,690,929	9,739,436	1	0													
1044	4	9,739,437	10,463,563	2	1	1_1_1_1_1_0_0_0_0_1_1_1_0.777	2	2	2	2	2	2	2	2	1	1	1	1	1
1045	4	10,463,564	11,562,348	1	0													
1046	4	11,562,349	11,666,309	2	1	1_1_1_1_1_0_0_0_0_1_1_1_1.75	2	2	2	2	2	2	2	2	1	2	1	1	1
1047	4	11,666,310	11,675,386	1	0													
1048	4	11,675,387	12,142,742	2	1	1_1_1_1_1_0_0_0_0_1_1_1_0.449	2	2	2	2	2	2	2	2	1	1	1	1	1
1049	4	12,142,743	13,087,459	1	0													
1050	4	13,087,460	15,031,106	2	1	1_1_1_1_1_0_0_0_0_1_1_1_0.224	2	2	2	2	2	2	2	2	1	1	1	1	1
1051	4	15,031,107	17,405,147	1	0													
1052	4	17,405,148	17,682,184	2	1	1_1_1_1_1_0_0_0_0_1_1_1_0.120	2	2	2	2	2	2	2	2	1	1	1	1	1
1053	4	17,682,185	17,731,782	1	0													
1054	4	17,731,783	18,008,403	2	1	1_1_1_1_1_0_0_0_0_1_1_1_0.180	2	2	2	2	2	2	2	2	1	1	1	1	1
1055	4	18,008,404	21,754,950	1	0													
1056	4	21,754,951	21,988,312	2	1	1_1_1_1_1_0_0_0_0_1_1_1_0.702	2	2	2	2	2	2	2	2	1	1	1	1	1
1057	4	21,988,313	22,030,750	3	2	1_1_1_1_1_0_0_0_0_1_1_1_0.702	3	3	3	3	3	3	3	3	2	1	1	1	1
1058	4	22,030,751	22,482,857	2	1	1_1_1_1_1_0_0_0_0_1_1_1_0.702	2	2	2	2	2	2	2	2	1	1	1	1	1
1059	4	22,482,858	22,643,533	1	0													
1060	4	22,643,534	23,776,641	2	1	1_1_1_1_1_0_0_0_0_1_1_1_0.123	2	2	2	2	2	2	2	2	1	1	1	1	1
1061	4	23,776,642	23,804,172	1	0													
1062	4	23,804,173	24,089,639	2	1	0_1_0_0_0_0_0_0_0_1_0_1_0.192	2	2	2	1	1	1	1	1	1	1	1	1	1
1063	4	24,089,640	24,227,923	3	2	0_1_0_0_0_0_0_0_0_1_0_1_0.192	3	3	3	2	2	2	2	2	1	1	1	1	1
1064	4	24,227,924	24,391,749	2	1	1_0_1_1_1_0_0_0_0_0_1_0_0.356	1	1	1	2	2	2	2	2	1	1	1	1	1
1065	4	24,391,750	24,398,460	3	2	1_0_1_1_1_0_0_0_0_0_1_0_0.356	2	2	2	3	3	3	3	3	1	1	1	1	1
1066	4	24,398,461	24,821,396	3	3	0_1_0_0_0_0_0_0_0_1_0_1_0.210	3	3	3	2	2	2	2	2	1	1	1	1	1
1067	4	24,821,397	24,822,359	3	2	1_0_1_1_1_0_0_0_0_0_1_0_0.356	2	2	2	3	3	3	3	3	1	1	1	1	1
1068	4	24,822,360	25,518,843	2	1	1_1_1_1_1_0_0_0_0_1_1_1_0.108	2	2	2	2	2	2	2	2	1	1	1	1	1
1069	4	25,518,844	25,534,766	3	2	1_0_1_1_1_0_0_0_0_0_1_0_0.127	2	2	2	3	3	3	3	3	1	1	1	1	1
1070	4	25,534,767	25,927,648	2	1	1_0_1_1_1_0_0_0_0_0_1_0_0.127	1	1	1	2	2	2	2	2	1	1	1	1	1
1071	4	25,927,649	26,015,030	3	2	1_0_1_1_1_0_0_0_0_0_1_0_0.127	2	2	2	3	3	3	3	3	1	1	1	1	1
1072	4	26,015,031	26,891,257	3	3	0_1_0_0_0_0_0_0_0_1_0_1_0.673	3	3	3	2	2	2	2	2	1	1	1	1	1
1073	4	26,891,258	26,899,896	3	2	0_1_0_0_0_0_0_0_0_1_0_1_0.673	3	3	3	2	2	2	2	2	1	1	1	1	1
1074	4	26,899,897	27,014,996	2	1	1_1_1_1_1_0_0_0_0_1_1_1_0.914	2	2	2	2	2	2	2	2	1	1	1	1	1
1075	4	27,014,997	27,086,047	3	2	0_0_0_0_0_0_0_0_0_0_1_0_0.25	2	2	2	2	2	2	2	3	1	1	1	1	1
1076	4	27,086,048	27,577,293	2	1	1_1_1_1_1_0_0_0_0_1_1_1_0.914	2	2	2	2	2	2	2	2	1	1	1	1	1
1077	4	27,577,294	28,157,309	1	0													
1078	4	28,157,310	31,949,211	2	1	0_0_0_0_0_0_0_0_0_0_0_0_1.252	1	1	1	1	1	1	1	1	1	2	1	1	1
1079	4	31,949,212	31,950,379	1	0													
1080	4	31,950,380	32,656,593	2	1	1_1_1_1_1_0_0_0_0_1_1_1_0.668	2	2	2	2	2	2	2	2	1	1	1	1	1
1081	4	32,656,594	32,665,137	1	0													
1082	4	32,665,138	34,974,973	2	1	1_1_1_1_1_0_1_0_0_1_1_1_0.144	2	2	2	2	2	2	2	2	2	1	1	1	1
1083	4	34,974,974	34,975,582	1	0													
1084	4	34,975,583	34,995,596	2	1	1_1_1_1_1_0_1_0_0_1_1_1_1.22	2	2	2	2	2	2	2	2	2	2	1	1	1
1085	4	34,995,597	41,637,054	1	0													
1086	4	41,637,055	41,665,166	2	1	0_1_0_0_0_0_0_0_0_1_0_1_0.32	2	2	2	1	1	1	1	1	1	1	1	1	1
1087	4	41,665,167	42,425,131	1	0													
1088	4	42,425,132	43,814,493	2	1	0_0_0_0_0_0_0_0_0_0_0_0_1.197	1	1	1	1	1	1	1	1	1	2	1	1	1
1089	4	43,814,494	43,815,337	3	2	0_0_0_0_0_0_0_0_0_0_0_0_1.197	2	2	2	1	1	1	1	1	1	3	1	1	1
1090	4	43,815,338	44,330,170	3	3	0_0_0_0_0_0_0_0_0_0_0_0_1.197	2	2	2	1	1	1	1	1	1	3	1	1	1
1091	4	44,330,171	44,333,154	3	2	0_0_0_0_0_0_0_0_0_0_0_0_1.197	2	2	2	1	1	1	1	1	1	3	1	1	1
1092	4	44,333,155	44,661,213	2	1	0_0_0_0_0_0_0_0_0_0_0_0_1.197	1	1	1	1	1	1	1	1	1	2	1	1	1
1093	4	44,661,214	44,714,727	1	0													
1094	4	44,714,728	47,524,371	2	1	0_0_0_0_0_0_0_0_0_0_0_0_1.262	1	1	1	1	1	1	1	1	1	2	1	1	1
1095	4	47,524,372	47,526,861	1	0													
1096	4	47,526,862	50,705,426	2	1	1_1_1_1_1_0_1_0_0_1_1_1_0.392	2	2	2	2	2	2	2	2	2	1	1	1	1
1097	4	50,705,427	50,725,499	1	0													
1098	4	50,725,500	50,759,262	2	1	0_1_0_0_0_0_0_0_0_1_0_1_0.633	2	2	2	1	1	1	1	1	1	1	1	1	1

1099	4	50,759,263	50,762,581	3	2	0_1_0_0_0_0_0_0_0_1_0_1_0.633	3	3	3	2	2	2	2	2	2	1	1	1	1
1100	4	50,762,582	51,779,946	3	3	0_1_0_0_0_0_0_0_0_1_0_1_0.633	3	3	3	2	2	2	2	2	2	1	1	1	1
1101	4	51,779,947	51,785,781	3	2	1_0_1_1_1_0_1_0_0_0_1_0_0.522	2	2	2	3	3	3	3	3	3	1	1	1	1
1102	4	51,785,782	51,865,791	2	1	1_1_1_1_1_0_1_0_0_1_1_1_0.498	2	2	2	2	2	2	2	2	2	1	1	1	1
1103	4	51,865,792	52,085,551	1	0														
1104	4	52,085,552	52,093,397	2	1	1_1_1_1_1_0_1_0_0_1_1_1_0.134	2	2	2	2	2	2	2	2	2	1	1	1	1
1105	4	52,093,398	52,098,192	3	2	1_0_1_1_1_0_1_0_0_0_1_0_0.112	2	2	2	3	3	3	3	3	3	1	1	1	1
1106	4	52,098,193	52,442,825	3	3	0_1_0_0_0_0_0_0_0_1_0_1_0.186	3	3	3	2	2	2	2	2	2	1	1	1	1
1107	4	52,442,826	52,445,369	3	2	0_1_0_0_0_0_0_0_0_1_0_1_0.186	3	3	3	2	2	2	2	2	2	1	1	1	1
1108	4	52,445,370	53,022,501	2	1	1_1_1_1_1_0_1_0_0_1_1_1_0.134	2	2	2	2	2	2	2	2	2	1	1	1	1
1109	4	53,022,502	54,946,109	1	0														
1110	4	54,946,110	55,637,844	2	1	0_1_0_0_0_0_0_0_0_1_0_1_0.683	2	2	2	1	1	1	1	1	1	1	1	1	1
1111	4	55,637,845	55,748,765	1	0														
1112	4	55,748,766	56,467,377	2	1	0_1_0_0_0_0_0_0_0_1_0_1_0.905	2	2	2	1	1	1	1	1	1	1	1	1	1
1113	4	56,467,378	62,937,591	1	0														
1114	4	62,937,592	64,326,619	2	1	0_1_0_0_0_0_0_0_0_1_0_1_0.127	2	2	2	1	1	1	1	1	1	1	1	1	1
1115	4	64,326,620	64,332,160	1	0														
1116	4	64,332,161	65,504,627	2	1	1_1_1_1_1_0_1_0_0_1_1_1_0.126	2	2	2	2	2	2	2	2	2	1	1	1	1
1117	4	65,504,628	65,726,450	1	0														
1118	4	65,726,451	68,137,276	2	1	1_1_1_1_1_0_1_0_0_1_1_1_0.253	2	2	2	2	2	2	2	2	2	1	1	1	1
1119	4	68,137,277	68,180,376	3	2	1_0_1_1_1_1_1_1_1_1_1_1_1.32	1	3	3	3	3	3	3	3	3	2	2	2	2
1120	4	68,180,377	69,285,926	2	1	1_1_1_1_1_0_1_0_0_1_1_1_0.253	2	2	2	2	2	2	2	2	2	1	1	1	1
1121	4	69,285,927	69,297,627	1	0														
1122	4	69,297,628	69,551,849	2	1	0_1_0_0_0_0_0_0_0_1_0_1_0.413	2	2	2	1	1	1	1	1	1	1	1	1	1
1123	4	69,551,850	70,571,186	1	0														
1124	4	70,571,187	70,838,422	2	1	0_1_0_0_0_0_0_0_0_1_0_1_0.931	2	2	2	1	1	1	1	1	1	1	1	1	1
1125	4	70,838,423	70,850,057	1	0														
1126	4	70,850,058	73,345,682	2	1	1_1_1_1_1_0_1_0_0_1_1_1_0.266	2	2	2	2	2	2	2	2	2	1	1	1	1
1127	4	73,345,683	73,425,394	1	0														
1128	4	73,425,395	75,500,593	2	1	1_1_1_1_1_0_1_0_0_1_1_1_0.304	2	2	2	2	2	2	2	2	2	1	1	1	1
1129	4	75,500,594	80,568,958	1	0														
1130	4	80,568,959	80,606,849	2	1	0_1_0_0_0_0_0_0_0_1_0_1_0.243	2	2	2	1	1	1	1	1	1	1	1	1	1
1131	4	80,606,850	80,607,095	3	2	0_1_0_0_0_0_0_0_0_1_0_1_0.243	3	3	3	2	2	2	2	2	2	1	1	1	1
1132	4	80,607,096	81,764,113	3	3	0_1_0_0_0_0_0_0_0_1_0_1_0.243	3	3	3	2	2	2	2	2	2	1	1	1	1
1133	4	81,764,114	81,767,106	3	2	0_1_0_0_0_0_0_0_0_1_0_1_0.243	3	3	3	2	2	2	2	2	2	1	1	1	1
1134	4	81,767,107	84,167,803	2	1	0_1_0_0_0_0_0_0_0_1_0_1_0.243	2	2	2	1	1	1	1	1	1	1	1	1	1
1135	4	84,167,804	84,167,886	1	0														
1136	4	84,167,887	85,193,823	2	1	1_1_1_1_1_0_1_0_0_1_0_1_0.182	2	2	2	2	2	2	2	1	2	1	1	1	1
1137	4	85,193,824	85,196,480	1	0														
1138	4	85,196,481	89,433,199	2	1	1_1_1_1_1_0_1_0_0_1_1_1_0.357	2	2	2	2	2	2	2	2	2	1	1	1	1
1139	4	89,433,200	89,482,912	1	0														
1140	4	89,482,913	90,813,983	2	1	1_1_1_1_1_0_1_0_0_1_1_1_0.156	2	2	2	2	2	2	2	2	2	1	1	1	1
1141	4	90,813,984	92,228,682	1	0														
1142	4	92,228,683	92,961,246	2	1	1_1_1_1_1_0_1_0_0_1_1_1_0.198	2	2	2	2	2	2	2	2	2	1	1	1	1
1143	4	92,961,247	93,010,719	3	2	0_0_0_0_0_0_0_0_0_0_0_0_1.43	2	2	2	2	2	2	2	2	2	3	1	1	1
1144	4	93,010,720	94,235,256	2	1	1_1_1_1_1_0_1_0_0_1_1_1_0.198	2	2	2	2	2	2	2	2	2	1	1	1	1
1145	4	94,235,257	94,317,465	3	2	0_0_0_0_0_0_0_0_0_0_0_0_1.603	2	2	2	2	2	2	2	2	2	3	1	1	1
1146	4	94,317,466	94,560,562	2	1	0_0_0_0_0_0_0_0_0_0_0_0_1.603	1	1	1	1	1	1	1	1	1	2	1	1	1
1147	4	94,560,563	94,632,048	3	2	0_0_0_0_0_0_0_0_0_0_0_0_1.603	2	2	2	2	2	2	2	2	2	3	1	1	1
1148	4	94,632,049	95,293,452	3	3	0_0_0_0_0_0_0_0_0_0_0_0_1.603	2	2	2	2	2	2	2	2	2	3	1	1	1
1149	4	95,293,453	95,414,559	3	2	0_0_0_0_0_0_0_0_0_0_0_0_1.603	2	2	2	2	2	2	2	2	2	3	1	1	1
1150	4	95,414,560	95,464,274	3	3	0_0_0_0_0_0_0_0_0_0_0_0_1.603	2	2	2	2	2	2	2	2	2	3	1	1	1
1151	4	95,464,275	95,469,006	3	2	0_0_0_0_0_0_0_0_0_0_0_0_1.603	2	2	2	2	2	2	2	2	2	3	1	1	1
1152	4	95,469,007	96,338,396	2	1	1_1_1_1_1_0_1_0_0_1_1_1_1.930	2	2	2	2	2	2	2	2	2	2	1	1	1
1153	4	96,338,397	96,816,545	1	0														
1154	4	96,816,546	97,334,061	2	1	1_1_1_1_1_0_1_0_0_1_1_1_0.112	2	2	2	2	2	2	2	2	2	1	1	1	1
1155	4	97,334,062	97,349,382	3	2	1_1_1_1_1_0_0_0_0_1_1_1_0.45	3	3	3	3	3	3	3	3	2	1	1	1	1
1156	4	97,349,383	97,909,531	2	1	1_1_1_1_1_0_1_0_0_1_1_1_0.112	2	2	2	2	2	2	2	2	2	1	1	1	1
1157	4	97,909,532	97,960,158	1	0														
1158	4	97,960,159	102,117,018	2	1	1_1_1_1_1_0_1_0_0_1_1_1_0.329	2	2	2	2	2	2	2	2	2	1	1	1	1
1159	4	102,117,019	102,918,343	1	0														
1160	4	102,918,344	104,386,454	2	1	1_1_1_1_1_0_1_0_0_1_1_1_0.786	2	2	2	2	2	2	2	2	2	1	1	1	1
1161	4	104,386,455	104,420,288	1	0														
1162	4	104,420,289	104,629,175	2	1	1_1_1_1_1_0_1_0_0_1_1_1_1.270	2	2	2	2	2	2	2	2	2	2	1	1	1
1163	4	104,629,176	105,117,063	1	0														
1164	4	105,117,064	106,324,947	2	1	1_1_1_1_1_0_1_0_0_1_1_1_0.135	2	2	2	2	2	2	2	2	2	1	1	1	1
1165	4	106,324,948	106,379,560	1	0														
1166	4	106,379,561	115,469,162	2	1	1_1_1_1_1_0_1_0_0_1_1_1_0.875	2	2	2	2	2	2	2	2	2	1	1	1	1
1167	4	115,469,163	118,076,114	1	0														
1168	4	118,076,115	121,470,020	2	1	1_1_1_1_1_0_1_0_0_1_1_1_1.288	2	2	2	2	2	2	2	2	2	2	1	1	1
1169	4	121,470,021	134,150,628	1	0														
1170	4	134,150,629	134,196,876	2	1	1_1_1_1_1_0_1_0_0_1_1_1_0.32	2	2	2	2	2	2	2	2	2	1	1	1	1
1171	4	134,196,877	134,648,203	1	0														
1172	4	134,648,204	134,887,742	2	1	1_1_1_1_1_0_1_0_0_1_1_1_0.210	2	2	2	2	2	2	2	2	2	1	1	1	1
1173	4	134,887,743	136,334,349	1	0														
1174	4	136,334,350	138,339,137	2	1	0_1_0_0_0_0_0_0_0_1_1_1_0.162	2	2	2	1	1	1	1	2	1	1	1	1	1
1175	4	138,339,138	142,994,088	1	0														
1176	4	142,994,089	146,029,160	2	1	1_1_1_1_1_0_1_0_0_1_0_1_0.250	2	2	2	2	2	2	2	1	2	1	1	1	1
1177	4	146,029,161	157,056,867	1	0														
1178	4	157,056,868	158,076,010	2	1	1_1_1_1_1_0_1_0_0_1_1_1_1.477	2	2	2	2	2	2	2	2	2	2	1	1	1
1179	4	158,076,011	158,151,146	1	0														
1180	4	158,151,147	158,458,807	2	1	1_1_1_1_1_0_1_0_0_1_1_1_1.290	2	2	2	2	2	2	2	2	2	2	1	1	1
1181	4	158,458,808	158,574,043	1	0														
1182	4	158,574,044	158,918,945	2	1	1_1_1_1_1_0_1_0_0_1_1_1_0.369	2	2	2	2	2	2	2	2	2	1	1	1	1
1183	4	158,918,946	160,178,795	1	0														

1184	4	160,178,796	161,984,387	2	1	1_1_1_1_1_0_0_0_0_1_1_1_1.543	2	2	2	2	2	2	2	2	1	2	1	1	1
1185	4	161,984,388	167,157,365	1	0														
1186	4	167,157,366	168,058,096	2	1	0_0_0_0_0_0_0_0_0_0_0_0_1.327	1	1	1	1	1	1	1	1	1	2	1	1	1
1187	4	168,058,097	168,064,834	1	0														
1188	4	168,064,835	169,645,649	2	1	1_1_1_1_1_0_0_0_0_1_1_1_0.690	2	2	2	2	2	2	2	2	1	1	1	1	1
1189	4	169,645,650	169,775,788	1	0														
1190	4	169,775,789	169,914,201	2	1	1_1_1_1_1_0_0_0_0_1_0_1_0.153	2	2	2	2	2	2	2	1	1	1	1	1	1
1191	4	169,914,202	169,916,177	1	0														
1192	4	169,916,178	171,376,827	2	1	1_0_0_1_1_1_1_1_1_0_1_0_1.153	1	1	1	2	1	2	2	2	2	2	2	2	2
1193	4	171,376,828	184,052,228	1	0														
1194	4	184,052,229	184,195,686	2	1	1_1_1_1_1_0_0_0_0_1_0_1_0.32	2	2	2	2	2	2	2	1	1	1	1	1	1
1195	5	340,858	3,094,249	2	1	1_1_1_1_1_0_1_0_0_1_1_1_0.149	2	2	2	2	2	2	2	2	2	1	1	1	1
1196	5	3,094,250	3,115,335	1	0														
1197	5	3,115,336	3,265,262	2	1	0_0_0_0_0_0_0_0_0_0_1_0_0.57	1	1	1	1	1	1	1	2	1	1	1	1	1
1198	5	3,265,263	3,270,697	1	0														
1199	5	3,270,698	3,583,467	2	1	1_1_1_1_1_0_1_0_0_1_0_1_0.204	2	2	2	2	2	2	2	1	2	1	1	1	1
1200	5	3,583,468	3,687,028	3	2	1_1_1_1_1_0_1_0_0_1_0_1_0.204	3	3	3	3	3	3	3	2	3	1	1	1	1
1201	5	3,687,029	3,773,229	3	3	0_0_0_0_0_0_0_0_0_0_1_0_0.58	2	2	2	2	2	2	2	3	2	1	1	1	1
1202	5	3,773,230	3,825,454	3	2	1_1_1_1_1_0_1_0_0_1_0_1_0.204	3	3	3	3	3	3	3	2	3	1	1	1	1
1203	5	3,825,455	4,153,741	3	3	0_0_0_0_0_0_0_0_0_0_1_0_0.214	2	2	2	2	2	2	2	3	2	1	1	1	1
1204	5	4,153,742	4,156,893	3	2	1_1_1_1_1_0_1_0_0_1_0_1_0.204	3	3	3	3	3	3	3	2	3	1	1	1	1
1205	5	4,156,894	4,161,347	2	1	1_1_1_1_1_0_1_0_0_1_0_1_0.204	2	2	2	2	2	2	2	1	2	1	1	1	1
1206	5	4,161,348	4,373,529	3	2	1_1_1_1_1_0_1_0_0_1_0_1_0.204	3	3	3	3	3	3	3	1	3	1	2	1	2
1207	5	4,373,530	5,957,931	2	1	1_1_1_1_1_0_1_0_0_1_0_1_0.204	2	2	2	2	2	2	2	1	2	1	1	1	1
1208	5	5,957,932	7,736,692	1	0														
1209	5	7,736,693	11,173,361	2	1	1_1_1_1_1_0_1_0_0_1_0_1_0.390	2	2	2	2	2	2	2	1	2	1	1	1	1
1210	5	11,173,362	11,355,678	1	0														
1211	5	11,355,679	14,487,825	2	1	1_1_1_1_1_0_1_0_0_1_0_1_0.316	2	2	2	2	2	2	2	1	2	1	1	1	1
1212	5	14,487,826	14,675,453	1	0														
1213	5	14,675,454	15,801,328	2	1	1_1_1_1_1_0_1_0_0_1_0_1_0.134	2	2	2	2	2	2	2	1	2	1	1	1	1
1214	5	15,801,329	16,094,108	1	0														
1215	5	16,094,109	16,225,866	2	1	1_1_1_1_1_0_1_0_0_1_0_1_0.97	2	2	2	2	2	2	2	1	2	1	1	1	1
1216	5	16,225,867	16,509,351	1	0														
1217	5	16,509,352	16,839,802	2	1	1_1_1_1_1_1_1_0_1_1_1_1_1.220	2	2	2	2	2	2	2	2	2	2	2	1	2
1218	5	16,839,803	16,844,269	1	0														
1219	5	16,844,270	18,928,103	2	1	0_0_0_0_0_0_0_1_0_0_0_0_1.190	1	1	1	1	1	1	1	1	1	2	1	2	1
1220	5	18,928,104	18,960,844	1	0														
1221	5	18,960,845	20,258,819	2	1	0_0_0_0_0_1_0_0_1_0_1_0_0.296	1	1	1	1	1	1	1	2	1	1	2	1	2
1222	5	20,258,820	21,283,865	3	2	0_0_0_0_0_0_0_0_0_0_1_0_0.495	1	1	1	1	1	1	1	3	1	1	2	1	2
1223	5	21,283,866	21,995,335	2	1	0_0_0_0_0_1_0_0_1_0_1_0_0.296	1	1	1	1	1	1	1	2	1	1	2	1	2
1224	5	21,995,336	22,644,364	3	2	0_0_0_0_0_0_0_0_0_0_1_0_0.507	1	1	1	1	1	1	1	3	1	1	2	1	2
1225	5	22,644,365	22,755,971	2	1	0_0_0_0_0_1_0_0_1_0_1_0_0.296	1	1	1	1	1	1	1	2	1	1	2	1	2
1226	5	22,755,972	22,809,551	3	2	0_0_0_0_0_0_0_0_0_0_1_0_0.574	1	1	1	1	1	1	1	3	1	1	2	1	2
1227	5	22,809,552	23,456,219	2	1	0_0_0_0_0_0_0_0_0_0_1_0_0.574	1	1	1	1	1	1	1	2	1	1	1	1	1
1228	5	23,456,220	23,457,455	1	0														
1229	5	23,457,456	23,458,722	2	1	1_1_1_1_1_0_1_1_0_1_1_1_1.37	2	2	2	2	2	2	2	2	2	2	1	2	1
1230	5	23,458,723	23,459,219	3	2	1_1_1_1_1_0_1_0_0_1_1_1_0.229	3	3	3	3	3	3	3	3	3	2	1	2	1
1231	5	23,459,220	23,559,663	3	3	0_0_0_0_0_0_0_1_0_0_0_0_1.235	2	2	2	2	2	2	2	2	2	3	1	3	1
1232	5	23,559,664	23,638,845	3	2	0_0_0_0_0_0_0_1_0_0_0_0_1.235	2	2	2	2	2	2	2	2	2	3	1	3	1
1233	5	23,638,846	23,713,020	3	3	0_0_0_0_0_0_0_1_0_0_0_0_1.235	2	2	2	2	2	2	2	2	2	3	1	3	1
1234	5	23,713,021	23,734,537	3	2	0_0_0_0_0_0_0_1_0_0_0_0_1.235	2	2	2	2	2	2	2	2	2	3	1	3	1
1235	5	23,734,538	25,741,996	2	1	1_1_1_1_1_0_1_0_0_1_1_1_0.229	2	2	2	2	2	2	2	2	2	1	1	1	1
1236	5	25,741,997	25,807,406	1	0														
1237	5	25,807,407	26,597,482	2	1	1_1_1_1_1_0_1_0_0_1_1_1_0.103	2	2	2	2	2	2	2	2	2	1	1	1	1
1238	5	26,597,483	29,792,661	1	0														
1239	5	29,792,662	29,828,188	2	1	1_1_1_1_1_0_1_0_0_1_1_1_0.37	2	2	2	2	2	2	2	2	2	1	1	1	1
1240	5	29,828,189	29,981,906	1	0														
1241	5	29,981,907	30,122,104	2	1	1_1_1_1_1_0_1_0_0_1_1_1_0.407	2	2	2	2	2	2	2	2	2	1	1	1	1
1242	5	30,122,105	30,129,935	3	2	1_1_1_1_1_0_1_0_0_1_1_1_0.407	3	3	3	3	3	3	3	3	3	2	1	1	1
1243	5	30,129,936	30,208,668	3	3	0_0_0_0_0_0_0_0_0_0_0_0_1.357	2	2	2	2	2	2	2	2	2	3	1	1	1
1244	5	30,208,669	30,642,771	3	2	0_0_0_0_0_0_0_0_0_0_0_0_1.357	2	2	2	2	2	2	2	2	2	3	1	1	1
1245	5	30,642,772	33,432,964	2	1	0_0_0_0_0_0_0_0_0_0_0_0_1.357	1	1	1	1	1	1	1	1	1	2	1	1	1
1246	5	33,432,965	40,172,198	1	0														
1247	5	40,172,199	40,626,395	2	1	1_1_1_1_1_0_1_0_0_1_1_1_0.718	2	2	2	2	2	2	2	2	2	1	1	1	1
1248	5	40,626,396	40,901,080	1	0														
1249	5	40,901,081	41,889,702	2	1	1_1_1_1_1_0_1_0_0_1_1_1_0.122	2	2	2	2	2	2	2	2	2	1	1	1	1
1250	5	41,889,703	47,322,931	1	0														
1251	5	47,322,932	47,560,338	2	1	1_1_1_1_1_0_1_0_0_1_1_1_0.386	2	2	2	2	2	2	2	2	2	1	1	1	1
1252	5	47,560,339	47,642,066	1	0														
1253	5	47,642,067	50,643,491	2	1	1_1_1_1_1_0_1_0_0_1_1_1_0.287	2	2	2	2	2	2	2	2	2	1	1	1	1
1254	5	50,643,492	53,367,892	1	0														
1255	5	53,367,893	53,584,040	2	1	1_1_1_1_1_0_1_0_0_1_1_1_0.305	2	2	2	2	2	2	2	2	2	1	1	1	1
1256	5	53,584,041	53,862,979	1	0														
1257	5	53,862,980	54,238,072	2	1	1_1_1_1_1_0_1_0_0_1_1_1_0.354	2	2	2	2	2	2	2	2	2	1	1	1	1
1258	5	54,238,073	54,554,804	1	0														
1259	5	54,554,805	55,852,679	2	1	1_1_1_1_1_0_1_0_0_1_1_1_0.164	2	2	2	2	2	2	2	2	2	1	1	1	1
1260	5	55,852,680	56,134,436	1	0														
1261	5	56,134,437	60,103,859	2	1	1_1_1_1_1_0_1_0_0_1_1_1_0.417	2	2	2	2	2	2	2	2	2	1	1	1	1
1262	5	60,103,860	64,613,769	1	0														
1263	5	64,613,770	65,114,505	2	1	0_0_0_0_0_0_0_0_0_0_0_0_1.332	1	1	1	1	1	1	1	1	1	2	1	1	1
1264	5	65,114,506	65,161,060	3	2	0_0_0_0_0_0_0_0_0_0_0_0_1.332	2	2	2	1	1	1	1	1	1	3	1	1	1
1265	5	65,161,061	65,221,418	3	3	0_0_0_0_0_0_0_0_0_0_0_0_1.332	2	2	2	1	1	1	1	1	1	3	1	1	1
1266	5	65,221,419	65,221,542	3	2	0_0_0_0_0_0_0_0_0_0_0_0_1.332	1	1	1	2	2	2	2	2	2	3	2	2	2
1267	5	65,221,543	65,224,589	2	1	0_0_0_0_0_0_0_0_0_0_0_0_1.332	1	1	1	1	1	1	1	1	1	2	1	1	1
1268	5	65,224,590	65,250,308	3	2	0_0_0_0_0_0_0_0_0_0_0_0_1.332	2	2	2	1	1	1	1	2	1	3	1	1	1

Disease Models & Mechanisms • Supplementary information

1269	5	65,250,309	65,467,153	3	3	0_0_0_0_0_0_0_0_0_0_0_0_1.332	2	2	2	1	1	1	1	2	1	3	1	1	1
1270	5	65,467,154	65,473,642	3	2	0_0_0_0_0_0_0_0_0_0_0_0_1.332	2	2	2	1	1	1	1	2	1	3	1	1	1
1271	5	65,473,643	65,915,687	2	1	0_0_0_0_0_0_0_0_0_0_0_0_1.332	1	1	1	1	1	1	1	1	1	2	1	1	1
1272	5	65,915,688	66,088,418	3	2	0_0_0_0_0_0_0_0_0_0_0_0_1.332	2	2	2	1	1	1	1	2	1	3	1	1	1
1273	5	66,088,419	66,274,936	3	3	0_0_0_0_0_0_0_0_0_0_0_0_1.332	2	2	2	1	1	1	1	2	1	3	1	1	1
1274	5	66,274,937	66,447,945	3	2	0_0_0_0_0_0_0_0_0_0_0_0_1.332	2	2	2	1	1	1	1	2	1	3	1	1	1
1275	5	66,447,946	68,062,508	2	1	0_0_0_0_0_0_0_0_0_0_0_0_1.332	1	1	1	1	1	1	1	1	1	2	1	1	1
1276	5	68,062,509	68,702,185	1	0														
1277	5	68,702,186	68,873,366	2	1	0_0_0_0_0_0_0_0_0_0_0_0_1.384	1	1	1	1	1	1	1	1	1	2	1	1	1
1278	5	68,873,367	68,907,233	3	2	0_0_0_0_0_0_0_0_0_0_0_0_1.384	1	1	1	2	2	2	2	2	2	3	2	2	2
1279	5	68,907,234	68,945,307	2	1	1_0_1_1_1_1_1_1_1_0_1_0_0.266	1	1	1	2	2	2	2	2	2	1	2	2	2
1280	5	68,945,308	69,039,570	3	2	0_1_0_0_0_0_0_0_0_1_0_1_0.99	3	3	3	2	2	2	2	2	2	1	2	2	2
1281	5	69,039,571	69,151,468	3	3	0_0_0_0_0_0_0_0_0_0_0_0_1.183	2	2	2	1	1	1	1	1	1	3	1	1	1
1282	5	69,151,469	69,154,844	3	2	0_0_0_0_0_0_0_0_0_0_0_0_1.183	1	1	1	2	2	2	2	2	2	3	2	2	2
1283	5	69,154,845	69,576,519	2	1	0_0_0_0_0_0_0_0_0_0_0_0_1.183	1	1	1	1	1	1	1	1	1	2	1	1	1
1284	5	69,576,520	69,585,217	3	2	0_0_0_0_0_0_0_0_0_0_0_0_1.183	1	1	1	2	2	2	2	2	2	3	2	2	2
1285	5	69,585,218	69,786,360	3	3	0_0_0_0_0_0_0_0_0_0_0_0_1.183	2	2	2	1	1	1	1	1	1	3	1	1	1
1286	5	69,786,361	69,890,748	3	2	0_1_0_0_0_0_0_0_0_1_0_1_0.824	3	3	3	2	2	2	2	2	2	1	2	2	2
1287	5	69,890,749	70,189,472	3	3	0_0_0_0_0_0_0_0_0_0_0_0_1.348	2	2	2	1	1	1	1	1	1	3	1	1	1
1288	5	70,189,473	70,232,478	3	2	0_0_0_0_0_0_0_0_0_0_0_0_1.348	2	2	2	1	1	1	1	1	1	3	1	1	1
1289	5	70,232,479	70,581,125	3	3	0_0_0_0_0_0_0_0_0_0_0_0_1.348	2	2	2	1	1	1	1	1	1	3	1	1	1
1290	5	70,581,126	70,582,928	3	2	0_0_0_0_0_0_0_0_0_0_0_0_1.348	2	2	2	1	1	1	1	1	1	3	1	1	1
1291	5	70,582,929	70,727,476	2	1	0_1_0_0_0_0_0_0_0_1_0_1_0.824	2	2	2	1	1	1	1	1	1	1	1	1	1
1292	5	70,727,477	70,745,287	3	2	0_1_0_0_0_0_0_0_0_1_0_1_0.824	3	3	3	2	2	2	2	2	2	1	2	2	2
1293	5	70,745,288	70,810,762	3	3	0_0_0_0_0_0_0_0_0_0_0_0_1.554	2	2	2	1	1	1	1	1	1	3	1	1	1
1294	5	70,810,763	70,811,027	3	2	0_0_0_0_0_0_0_0_0_0_0_0_1.554	1	1	1	2	2	2	2	2	2	3	2	2	2
1295	5	70,811,028	70,814,173	2	1	0_0_0_0_0_0_0_0_0_0_0_0_1.554	1	1	1	1	1	1	1	1	1	2	1	1	1
1296	5	70,814,174	70,815,161	3	2	0_0_0_0_0_0_0_0_0_0_0_0_1.554	1	1	1	2	2	2	2	2	2	3	1	1	1
1297	5	70,815,162	71,951,998	3	3	0_0_0_0_0_0_0_0_0_0_0_0_1.554	2	2	2	1	1	1	1	1	1	3	2	2	2
1298	5	71,951,999	71,957,448	3	2	0_0_0_0_0_0_0_0_0_0_0_0_1.554	2	2	2	1	1	1	1	1	1	3	2	2	2
1299	5	71,957,449	72,392,360	2	1	0_1_0_0_0_1_0_1_1_1_0_1_0.887	2	2	2	1	1	1	1	1	1	1	2	2	2
1300	5	72,392,361	72,403,310	3	2	0_1_0_0_0_1_0_1_1_1_0_1_0.887	3	3	3	2	2	2	2	2	2	1	3	3	3
1301	5	72,403,311	72,480,643	3	3	0_0_0_0_0_0_0_0_0_0_0_0_1.346	2	2	2	1	1	1	1	1	1	3	2	2	2
1302	5	72,480,644	72,507,349	3	2	0_0_0_0_0_0_0_0_0_0_0_0_1.346	2	2	2	1	1	1	1	1	1	3	2	2	2
1303	5	72,507,350	72,611,067	2	1	0_0_0_0_0_0_0_0_0_0_0_0_1.346	1	1	1	1	1	1	1	1	1	2	1	1	1
1304	5	72,611,068	72,614,696	3	2	0_0_0_0_0_0_0_0_0_0_0_0_1.346	2	2	2	1	1	1	1	1	1	3	2	2	2
1305	5	72,614,697	72,750,780	3	3	0_0_0_0_0_0_0_0_0_0_0_0_1.346	2	2	2	1	1	1	1	1	1	3	2	2	2
1306	5	72,750,781	72,776,184	3	2	0_0_0_0_0_0_0_0_0_0_0_0_1.346	2	2	2	1	1	1	1	1	1	3	2	2	2
1307	5	72,776,185	72,778,681	3	3	0_0_0_0_0_0_0_0_0_0_0_0_1.346	2	2	2	1	1	1	1	1	1	3	2	2	2
1308	5	72,778,682	72,780,058	3	2	0_0_0_0_0_0_0_0_0_0_0_0_1.346	1	1	1	2	2	2	2	2	2	3	1	1	1
1309	5	72,780,059	73,687,145	2	1	1_0_1_1_1_0_1_0_0_0_1_0_0.162	1	1	1	2	2	2	2	2	2	1	1	1	1
1310	5	73,687,146	73,691,079	3	2	0_1_0_0_0_1_0_1_1_1_0_1_0.152	3	3	3	2	2	2	2	2	2	1	3	3	3
1311	5	73,691,080	73,894,412	3	3	0_0_0_0_0_0_0_0_0_0_0_0_1.83	2	2	2	1	1	1	1	1	1	3	2	2	2
1312	5	73,894,413	73,895,227	3	2	0_1_0_0_0_1_0_1_1_1_0_1_0.152	3	3	3	2	2	2	2	2	2	1	3	3	3
1313	5	73,895,228	74,535,500	2	1	1_0_1_1_1_0_1_0_0_0_1_0_0.162	1	1	1	2	2	2	2	2	2	1	1	1	1
1314	5	74,535,501	74,987,687	1	0														
1315	5	74,987,688	83,063,830	2	1	0_0_0_0_0_0_0_0_0_0_0_0_1.121	1	1	1	1	1	1	1	1	1	2	1	1	1
1316	5	83,063,831	83,078,568	3	2	0_0_0_0_0_0_0_0_0_0_0_0_1.121	2	2	2	1	1	1	1	2	1	3	2	2	2
1317	5	83,078,569	92,487,164	2	1	0_0_0_0_0_0_0_0_0_0_0_0_1.121	1	1	1	1	1	1	1	1	1	2	1	1	1
1318	5	92,487,165	92,578,409	1	0														
1319	5	92,578,410	93,227,652	2	1	0_0_0_0_0_0_0_0_0_0_0_0_1.186	1	1	1	1	1	1	1	1	1	2	1	1	1
1320	5	93,227,653	97,613,675	1	0														
1321	5	97,613,676	98,940,957	2	1	1_1_1_1_1_0_1_0_0_1_0_1_0.773	2	2	2	2	2	2	2	1	2	1	1	1	1
1322	5	98,940,958	98,946,761	1	0														
1323	5	98,946,762	99,190,248	2	1	0_0_0_0_0_0_0_0_0_0_0_0_1.249	1	1	1	1	1	1	1	1	1	2	1	1	1
1324	5	99,190,249	99,195,527	1	0														
1325	5	99,195,528	104,148,680	2	1	1_1_1_1_1_0_1_0_0_1_0_1_0.502	2	2	2	2	2	2	2	1	2	1	1	1	1
1326	5	104,148,681	104,149,629	1	0														
1327	5	104,149,630	105,347,240	2	1	1_1_1_1_1_0_1_0_0_1_1_1_0.785	2	2	2	2	2	2	2	2	2	1	1	1	1
1328	5	105,347,241	105,406,380	1	0														
1329	5	105,406,381	107,449,399	2	1	1_1_1_1_1_0_1_0_0_1_1_1_0.167	2	2	2	2	2	2	2	2	2	1	1	1	1
1330	5	107,449,400	107,586,725	1	0														
1331	5	107,586,726	108,446,988	2	1	0_0_0_0_0_1_0_0_1_0_0_0_1.684	1	1	1	1	1	1	1	1	1	2	2	1	2
1332	5	108,446,989	130,552,150	1	0														
1333	5	130,552,151	130,563,804	2	1	1_1_1_1_1_0_0_0_0_1_1_1_0.36	2	2	2	2	2	2	2	2	1	1	1	1	1
1334	5	130,563,805	133,744,359	1	0														
1335	5	133,744,360	138,592,456	2	1	0_0_0_0_0_0_0_0_0_0_0_0_1.335	1	1	1	1	1	1	1	1	1	2	1	1	1
1336	5	138,592,457	139,991,621	1	0														
1337	5	139,991,622	140,660,922	2	1	1_1_1_1_1_0_1_0_0_1_0_1_0.523	2	2	2	2	2	2	2	1	2	1	1	1	1
1338	5	140,660,923	143,674,940	1	0														
1339	5	143,674,941	144,357,339	2	1	1_1_1_1_1_0_0_0_0_1_1_1_1.498	2	2	2	2	2	2	2	2	1	2	1	1	1
1340	5	144,357,340	146,908,416	1	0														
1341	5	146,908,417	148,638,312	2	1	1_1_1_1_1_0_0_0_0_1_1_1_0.171	2	2	2	2	2	2	2	2	1	1	1	1	1
1342	5	148,638,313	148,687,101	3	2	1_1_1_1_1_0_0_0_0_1_1_1_0.171	3	3	3	3	3	3	3	3	2	1	1	1	1
1343	5	148,687,102	149,284,940	2	1	1_1_1_1_1_0_1_0_0_1_1_1_0.764	2	2	2	2	2	2	2	2	2	1	1	1	1
1344	5	149,284,941	149,286,743	1	0														
1345	5	149,286,744	149,403,906	2	1	1_1_1_1_1_0_1_0_0_1_1_1_1.92	2	2	2	2	2	2	2	2	2	2	1	1	1
1346	5	149,403,907	149,407,576	1	0														
1347	5	149,407,577	151,571,883	2	1	1_1_1_1_1_0_1_0_0_1_1_1_0.281	2	2	2	2	2	2	2	2	2	1	1	1	1
1348	5	151,571,884	151,574,570	1	0														
1349	5	151,574,571	152,421,646	2	1	0_0_0_0_0_0_0_0_0_0_0_0_1.577	1	1	1	1	1	1	1	1	1	2	1	1	1
1350	5	152,421,647	152,423,261	1	0														
1351	5	152,423,262	153,552,974	2	1	1_0_1_1_1_1_1_1_1_0_1_0_0.117	1	1	1	2	2	2	2	2	2	1	2	2	2
1352	5	153,552,975	153,560,134	1	0														
1353	5	153,560,135	154,235,860	2	1	0_1_0_0_0_0_0_0_0_1_0_1_0.490	2	2	2	1	1	1	1	1	1	1	1	1	1

Disease Models & Mechanisms • Supplementary information

1354	5	154,235,861	156,860,193	1	0														
1355	5	156,860,194	157,301,109	2	1	1_1_1_1_1_0_0_0_0_1_1_1_1.377	2	2	2	2	2	2	2	2	1	2	1	1	1
1356	5	157,301,110	158,046,374	1	0														
1357	5	158,046,375	158,999,027	2	1	0_0_0_0_0_0_0_0_0_0_0_0_1.936	1	1	1	1	1	1	1	1	1	2	1	1	1
1358	5	158,999,028	159,109,463	1	0														
1359	5	159,109,464	162,244,891	2	1	0_0_0_0_0_0_0_0_0_0_0_0_1.359	1	1	1	1	1	1	1	1	1	2	1	1	1
1360	5	162,244,892	163,246,622	1	0														
1361	5	163,246,623	163,477,309	2	1	0_0_0_0_0_0_0_0_0_0_0_0_1.175	1	1	1	1	1	1	1	1	1	2	1	1	1
1362	5	163,477,310	163,954,722	3	2	0_0_0_0_0_0_0_0_0_0_0_0_1.175	2	2	2	1	1	1	1	1	2	3	2	2	2
1363	5	163,954,723	166,283,682	2	1	0_1_0_0_0_1_1_1_1_1_0_1_0.143	2	2	2	1	1	1	1	1	2	1	2	2	2
1364	5	166,283,683	166,397,512	1	0														
1365	5	166,397,513	166,873,972	2	1	0_1_0_0_0_1_1_1_1_1_0_1_0.508	2	2	2	1	1	1	1	1	2	1	2	2	2
1366	5	166,873,973	166,875,180	1	0														
1367	5	166,875,181	169,299,882	2	1	0_1_0_0_0_1_0_1_1_1_0_1_0.244	2	2	2	1	1	1	1	1	1	1	2	2	2
1368	5	169,299,883	169,302,326	1	0														
1369	5	169,302,327	169,614,779	2	1	0_0_0_0_0_0_0_0_0_0_1_0_1.256	1	1	1	1	1	1	1	2	1	2	1	1	1
1370	5	169,614,780	170,861,915	1	0														
1371	5	170,861,916	171,528,213	2	1	0_0_0_0_0_0_0_0_0_0_0_0_1.326	1	1	1	1	1	1	1	1	1	2	1	1	1
1372	6	7,586	349,966	2	1	0_0_0_0_0_0_0_0_0_0_1_0_0.487	1	1	1	1	1	1	1	2	1	1	1	1	1
1373	6	349,967	351,284	1	0														
1374	6	351,285	827,605	2	1	0_0_0_0_0_1_1_1_1_0_1_0_0.547	1	1	1	1	1	1	1	2	2	1	2	2	2
1375	6	827,606	847,605	3	2	0_0_0_0_0_0_0_0_0_0_1_0_0.113	1	1	1	1	1	1	1	3	2	1	2	2	2
1376	6	847,606	1,811,286	2	1	0_0_0_0_0_0_0_0_0_0_1_0_0.113	1	1	1	1	1	1	1	2	1	1	1	1	1
1377	6	1,811,287	2,237,739	1	0														
1378	6	2,237,740	3,804,605	2	1	0_0_0_0_0_0_0_0_0_0_1_0_0.139	1	1	1	1	1	1	1	2	1	1	1	1	1
1379	6	3,804,606	3,844,546	3	2	0_0_0_0_0_0_0_0_0_0_0_0_1.33	1	1	1	1	1	1	1	2	1	3	1	1	1
1380	6	3,844,547	3,886,098	2	1	0_0_0_0_0_0_0_0_0_0_1_0_0.139	1	1	1	1	1	1	1	2	1	1	1	1	1
1381	6	3,886,099	4,498,634	1	0														
1382	6	4,498,635	5,839,263	2	1	0_0_0_0_0_0_0_0_0_0_0_0_1.155	1	1	1	1	1	1	1	1	1	2	1	1	1
1383	6	5,839,264	5,854,039	1	0														
1384	6	5,854,040	5,924,530	2	1	1_1_1_1_1_0_1_0_0_1_1_1_0.89	2	2	2	2	2	2	2	2	2	1	1	1	1
1385	6	5,924,531	5,935,038	1	0														
1386	6	5,935,039	7,978,441	2	1	0_0_0_0_0_0_0_0_0_0_0_0_1.202	1	1	1	1	1	1	1	1	1	2	1	1	1
1387	6	7,978,442	10,408,269	1	0														
1388	6	10,408,270	11,046,089	2	1	0_0_0_0_0_0_0_0_0_0_0_0_1.270	1	1	1	1	1	1	1	1	1	2	1	1	1
1389	6	11,046,090	11,559,635	1	0														
1390	6	11,559,636	14,504,026	2	1	0_0_0_0_0_0_0_0_0_0_0_0_1.276	1	1	1	1	1	1	1	1	1	2	1	1	1
1391	6	14,504,027	18,890,516	3	2	0_0_0_0_0_0_0_0_0_0_0_0_1.276	1	1	1	2	2	2	2	1	1	3	1	1	1
1392	6	18,890,517	19,072,587	2	1	1_0_1_1_1_0_0_0_0_0_0_0_1.651	1	1	1	2	2	2	2	1	1	2	1	1	1
1393	6	19,072,588	19,099,103	1	0														
1394	6	19,099,104	19,266,237	2	1	0_0_0_0_0_0_0_0_0_0_1_0_0.118	1	1	1	1	1	1	1	2	1	1	1	1	1
1395	6	19,266,238	19,416,718	1	0														
1396	6	19,416,719	19,730,097	2	1	0_0_0_0_0_0_0_0_0_0_1_0_0.238	1	1	1	1	1	1	1	2	1	1	1	1	1
1397	6	19,730,098	19,802,542	1	0														
1398	6	19,802,543	20,249,068	2	1	0_0_0_0_0_0_0_0_0_0_1_0_0.542	1	1	1	1	1	1	1	2	1	1	1	1	1
1399	6	20,249,069	20,786,984	1	0														
1400	6	20,786,985	22,575,282	2	1	0_0_0_0_0_0_0_0_0_0_1_0_0.205	1	1	1	1	1	1	1	2	1	1	1	1	1
1401	6	22,575,283	22,674,832	1	0														
1402	6	22,674,833	23,545,077	2	1	1_1_1_1_1_0_1_0_0_1_0_1_0.188	2	2	2	2	2	2	2	1	2	1	1	1	1
1403	6	23,545,078	23,781,477	3	2	1_1_1_1_1_0_1_0_0_1_0_1_0.188	3	3	3	3	3	3	3	2	3	1	1	1	1
1404	6	23,781,478	24,089,598	3	3	0_0_0_0_0_0_0_0_0_0_1_0_0.118	2	2	2	2	2	2	2	3	2	1	1	1	1
1405	6	24,089,599	24,099,666	3	2	0_0_0_0_0_0_0_0_0_0_1_0_0.118	2	2	2	2	2	2	2	3	2	1	1	1	1
1406	6	24,099,667	24,867,442	2	1	1_1_1_1_1_0_1_0_0_1_0_1_0.188	2	2	2	2	2	2	2	1	2	1	1	1	1
1407	6	24,867,443	25,191,742	1	0														
1408	6	25,191,743	25,299,245	2	1	0_0_0_0_0_0_0_0_0_0_1_0_0.97	1	1	1	1	1	1	1	2	1	1	1	1	1
1409	6	25,299,246	25,346,145	1	0														
1410	6	25,346,146	39,449,045	2	1	1_1_1_1_1_0_1_0_0_1_1_1_0.836	2	2	2	2	2	2	2	2	2	1	1	1	1
1411	6	39,449,046	40,565,496	1	0														
1412	6	40,565,497	40,689,038	2	1	1_1_1_1_1_0_1_0_0_1_1_1_0.134	2	2	2	2	2	2	2	2	2	1	1	1	1
1413	6	40,689,039	46,709,023	1	0														
1414	6	46,709,024	47,555,856	2	1	1_1_1_1_1_0_0_0_0_1_1_1_0.104	2	2	2	2	2	2	2	2	1	1	1	1	1
1415	6	47,555,857	48,447,001	1	0														
1416	6	48,447,002	48,552,210	2	1	1_1_1_1_1_0_0_0_0_1_1_1_0.95	2	2	2	2	2	2	2	2	1	1	1	1	1
1417	6	48,552,211	49,761,887	1	0														
1418	6	49,761,888	49,967,442	2	1	1_1_1_1_1_0_0_0_0_1_1_1_0.201	2	2	2	2	2	2	2	2	1	1	1	1	1
1419	6	49,967,443	50,056,326	1	0														
1420	6	50,056,327	50,218,748	2	1	1_1_1_1_1_0_0_0_0_1_1_1_0.101	2	2	2	2	2	2	2	2	1	1	1	1	1
1421	6	50,218,749	50,356,695	1	0														
1422	6	50,356,696	57,021,305	2	1	1_1_1_1_1_0_0_0_0_1_1_1_0.676	2	2	2	2	2	2	2	2	1	1	1	1	1
1423	6	57,021,306	57,054,712	1	0														
1424	6	57,054,713	62,314,166	2	1	1_1_1_1_1_0_0_0_0_1_1_1_0.666	2	2	2	2	2	2	2	2	1	1	1	1	1
1425	6	62,314,167	62,413,284	1	0														
1426	6	62,413,285	71,672,066	2	1	1_1_1_1_1_0_0_0_0_1_1_1_0.958	2	2	2	2	2	2	2	2	1	1	1	1	1
1427	6	71,672,067	74,509,950	1	0														
1428	6	74,509,951	74,748,835	2	1	1_1_1_1_1_0_0_0_0_1_1_1_0.189	2	2	2	2	2	2	2	2	1	1	1	1	1
1429	6	74,748,836	75,284,286	1	0														
1430	6	75,284,287	76,363,118	2	1	1_1_1_1_1_0_0_0_0_1_1_1_0.641	2	2	2	2	2	2	2	2	1	1	1	1	1
1431	6	76,363,119	82,708,299	1	0														
1432	6	82,708,300	87,477,284	2	1	1_1_1_1_1_0_0_0_0_1_1_1_0.502	2	2	2	2	2	2	2	2	1	1	1	1	1
1433	6	87,477,285	87,578,687	1	0														
1434	6	87,578,688	89,742,280	2	1	1_1_1_1_1_0_0_0_0_1_1_1_0.227	2	2	2	2	2	2	2	2	1	1	1	1	1
1435	6	89,742,281	94,798,387	1	0														
1436	6	94,798,388	95,149,349	2	1	1_1_1_1_1_0_0_0_0_1_1_1_0.639	2	2	2	2	2	2	2	2	1	1	1	1	1
1437	6	95,149,350	100,044,038	1	0														
1438	6	100,044,039	100,183,675	2	1	1_1_1_1_1_0_0_0_0_1_1_1_0.169	2	2	2	2	2	2	2	2	1	1	1	1	1

Disease Models & Mechanisms • Supplementary information

1439	6	100,183,676	105,559,175	1	0														
1440	6	105,559,176	106,416,732	2	1	1_1_1_1_1_0_0_0_0_1_1_1_0.615	2	2	2	2	2	2	2	2	1	1	1	1	1
1441	6	106,416,733	106,444,297	1	0														
1442	6	106,444,298	106,758,525	2	1	0_0_0_0_0_0_0_0_0_0_0_0_1.168	1	1	1	1	1	1	1	1	1	2	1	1	1
1443	6	106,758,526	106,843,286	1	0														
1444	6	106,843,287	107,689,423	2	1	0_0_0_0_0_0_0_0_0_0_0_0_1.793	1	1	1	1	1	1	1	1	1	2	1	1	1
1445	6	107,689,424	107,707,136	1	0														
1446	6	107,707,137	107,772,037	2	1	1_1_1_1_1_0_0_0_0_1_1_1_0.160	2	2	2	2	2	2	2	2	1	1	1	1	1
1447	6	107,772,038	107,772,698	1	0														
1448	6	107,772,699	111,439,253	2	1	0_0_0_0_0_0_0_0_0_0_0_0_1.298	1	1	1	1	1	1	1	1	1	2	1	1	1
1449	6	111,439,254	112,020,347	1	0														
1450	6	112,020,348	112,556,968	2	1	0_0_0_0_0_0_0_0_0_0_0_0_1.682	1	1	1	1	1	1	1	1	1	2	1	1	1
1451	6	112,556,969	113,517,992	1	0														
1452	6	113,517,993	113,733,434	2	1	0_0_0_0_0_0_0_0_0_0_0_0_1.350	1	1	1	1	1	1	1	1	1	2	1	1	1
1453	6	113,733,435	114,328,450	1	0														
1454	6	114,328,451	118,899,635	2	1	0_0_0_0_0_0_0_0_0_0_0_0_1.487	1	1	1	1	1	1	1	1	1	2	1	1	1
1455	6	118,899,636	119,611,341	1	0														
1456	6	119,611,342	120,497,075	2	1	1_1_1_1_1_1_1_0_1_1_1_1_1.442	2	2	2	2	2	2	2	2	2	2	2	1	2
1457	6	120,497,076	125,946,893	1	0														
1458	6	125,946,894	126,001,602	2	1	0_0_0_0_0_1_0_0_1_0_0_0_1.54	1	1	1	1	1	1	1	1	1	2	2	1	2
1459	6	126,001,603	126,013,985	1	0														
1460	6	126,013,986	126,108,388	2	1	0_0_0_0_0_0_0_0_0_0_0_0_1.149	1	1	1	1	1	1	1	1	1	2	1	1	1
1461	6	126,108,389	126,109,835	3	2	0_0_0_0_0_0_0_0_0_0_0_0_1.149	2	2	2	2	2	2	2	2	2	3	1	1	1
1462	6	126,109,836	126,116,456	3	3	0_0_0_0_0_0_0_0_0_0_0_0_1.149	2	2	2	2	2	2	2	2	2	3	1	1	1
1463	6	126,116,457	126,127,576	3	2	1_1_1_1_1_0_1_0_0_1_1_1_0.688	3	3	3	3	3	3	3	3	3	2	1	1	1
1464	6	126,127,577	126,193,286	2	1	1_1_1_1_1_0_1_0_0_1_1_1_0.688	2	2	2	2	2	2	2	2	2	1	1	1	1
1465	6	126,193,287	126,237,931	3	2	0_0_0_0_0_0_0_0_0_0_0_0_1.22	2	2	2	2	2	2	2	2	2	3	1	1	1
1466	6	126,237,932	126,279,728	3	3	0_0_0_0_0_0_0_0_0_0_0_0_1.22	2	2	2	2	2	2	2	2	2	3	1	1	1
1467	6	126,279,729	126,313,127	3	2	1_1_1_1_1_0_1_0_0_1_1_1_0.688	3	3	3	3	3	3	3	3	3	2	1	1	1
1468	6	126,313,128	126,344,877	3	3	0_0_0_0_0_0_0_0_0_0_0_0_1.20	2	2	2	2	2	2	2	2	2	3	1	1	1
1469	6	126,344,878	126,456,911	3	2	1_1_1_1_1_0_1_0_0_1_1_1_0.688	3	3	3	3	3	3	3	3	3	2	1	1	1
1470	6	126,456,912	132,273,972	2	1	1_1_1_1_1_0_1_0_0_1_1_1_0.688	2	2	2	2	2	2	2	2	2	1	1	1	1
1471	6	132,273,973	134,120,384	1	0														
1472	6	134,120,385	137,024,053	2	1	1_1_1_1_1_0_1_0_0_1_1_1_0.279	2	2	2	2	2	2	2	2	2	1	1	1	1
1473	6	137,024,054	137,026,180	3	2	0_1_0_0_0_0_0_0_0_1_0_1_0.177	3	3	3	2	2	2	2	2	2	1	1	1	1
1474	6	137,026,181	137,410,098	3	3	0_1_0_0_0_0_0_0_0_1_0_1_0.177	3	3	3	2	2	2	2	2	2	1	1	1	1
1475	6	137,410,099	137,415,012	3	2	0_1_0_0_0_0_0_0_0_1_0_1_0.177	3	3	3	2	2	2	2	2	2	1	1	1	1
1476	6	137,415,013	137,422,029	2	1	1_0_1_1_1_0_1_0_0_0_1_0_0.180	1	1	1	2	2	2	2	2	2	1	1	1	1
1477	6	137,422,030	137,648,992	1	0														
1478	6	137,648,993	137,650,131	2	1	1_0_1_1_1_0_1_0_0_0_1_0_0.108	1	1	1	2	2	2	2	2	2	1	1	1	1
1479	6	137,650,132	137,661,456	3	2	0_1_0_0_0_0_0_0_0_1_0_1_0.107	3	3	3	2	2	2	2	2	2	1	1	1	1
1480	6	137,661,457	140,932,316	3	3	0_1_0_0_0_0_0_0_0_1_0_1_0.107	3	3	3	2	2	2	2	2	2	1	1	1	1
1481	6	140,932,317	140,932,760	3	2	0_1_0_0_0_0_0_0_0_1_0_1_0.107	3	3	3	2	2	2	2	2	2	1	1	1	1
1482	6	140,932,761	140,999,214	2	1	1_1_1_1_1_0_1_0_0_1_1_1_0.180	2	2	2	2	2	2	2	2	2	1	1	1	1
1483	6	140,999,215	141,011,608	3	2	0_1_0_0_0_0_0_0_0_1_0_1_0.321	3	3	3	2	2	2	2	2	2	1	1	1	1
1484	6	141,011,609	141,916,162	3	3	0_1_0_0_0_0_0_0_0_1_0_1_0.321	3	3	3	2	2	2	2	2	2	1	1	1	1
1485	6	141,916,163	141,921,333	3	2	0_1_0_0_0_0_0_0_0_1_0_1_0.321	3	3	3	2	2	2	2	2	2	1	1	1	1
1486	6	141,921,334	144,954,399	2	1	0_1_0_0_0_0_0_0_0_1_0_1_0.321	2	2	2	1	1	1	1	1	1	1	1	1	1
1487	6	144,954,400	145,076,526	3	2	0_1_0_0_0_0_0_0_0_1_0_1_0.321	3	3	3	2	2	2	2	2	2	1	1	1	1
1488	6	145,076,527	145,292,639	3	3	0_1_0_0_0_0_0_0_0_1_0_1_0.321	3	3	3	2	2	2	2	2	2	1	1	1	1
1489	6	145,292,640	145,293,003	3	2	1_0_1_1_1_0_1_0_0_0_1_0_0.202	2	2	2	3	3	3	3	3	3	1	1	1	1
1490	6	145,293,004	145,315,958	2	1	1_1_1_1_1_0_1_0_0_1_1_1_0.417	2	2	2	2	2	2	2	2	2	1	1	1	1
1491	6	145,315,959	145,416,068	3	2	0_1_0_0_0_1_0_1_1_1_1_1_1.201	3	3	3	1	1	1	1	3	1	2	2	2	2
1492	6	145,416,069	145,547,435	3	3	0_1_0_0_0_0_0_0_0_1_1_1_0.413	3	3	3	1	1	1	1	3	1	2	2	2	2
1493	6	145,547,436	145,571,952	3	2	0_1_0_0_0_0_0_0_0_1_1_1_0.413	3	3	3	2	2	2	2	3	2	1	1	1	1
1494	6	145,571,953	145,680,920	2	1	0_1_0_0_0_0_0_0_0_1_1_1_0.413	2	2	2	1	1	1	1	2	1	1	1	1	1
1495	6	145,680,921	145,805,223	3	2	0_1_0_0_0_0_0_0_0_1_1_1_0.413	3	3	3	2	2	2	2	3	2	1	1	1	1
1496	6	145,805,224	145,925,706	3	3	0_1_0_0_0_0_0_0_0_1_1_1_0.413	3	3	3	1	1	1	1	3	1	2	2	2	2
1497	6	145,925,707	146,027,216	3	2	0_1_0_0_0_0_0_0_0_1_1_1_0.413	3	3	3	2	2	2	2	3	2	1	1	1	1
1498	6	146,027,217	147,965,308	2	1	1_1_1_1_1_0_1_0_0_1_1_1_0.118	2	2	2	2	2	2	2	2	2	1	1	1	1
1499	7	12,955,570	13,746,982	2	1	1_1_1_1_1_0_0_0_0_1_1_1_0.957	2	2	2	2	2	2	2	2	1	1	1	1	1
1500	7	13,746,983	13,784,141	1	0														
1501	7	13,784,142	13,874,187	2	1	1_0_1_1_1_0_1_0_0_0_1_0_0.90	1	1	1	2	2	2	2	2	2	1	1	1	1
1502	7	13,874,188	14,046,107	1	0														
1503	7	14,046,108	15,250,837	2	1	1_1_1_1_1_0_0_0_0_1_1_1_0.825	2	2	2	2	2	2	2	2	1	1	1	1	1
1504	7	15,250,838	15,426,707	1	0														
1505	7	15,426,708	15,921,413	2	1	1_1_1_1_1_0_0_0_0_1_1_1_0.283	2	2	2	2	2	2	2	2	1	1	1	1	1
1506	7	15,921,414	16,886,806	1	0														
1507	7	16,886,807	18,341,453	2	1	1_1_1_1_1_0_0_0_0_1_1_1_0.189	2	2	2	2	2	2	2	2	1	1	1	1	1
1508	7	18,341,454	18,344,208	3	2	1_1_1_1_1_0_0_0_0_1_1_1_0.189	3	3	3	3	3	3	3	3	1	2	1	1	1
1509	7	18,344,209	18,371,309	3	3	0_0_0_0_0_0_0_0_0_0_0_0_1.409	2	2	2	2	2	2	2	2	1	3	1	1	1
1510	7	18,371,310	23,467,157	3	2	0_0_0_0_0_0_0_0_0_0_0_0_1.409	2	2	2	2	2	2	2	2	1	3	1	1	1
1511	7	23,467,158	23,711,297	3	3	0_0_0_0_0_0_0_0_0_0_0_0_1.409	2	2	2	2	2	2	2	2	1	3	1	1	1
1512	7	23,711,298	23,817,670	3	2	0_0_0_0_0_0_0_0_0_0_0_0_1.409	2	2	2	2	2	2	2	2	1	3	1	1	1
1513	7	23,817,671	24,090,674	3	3	0_0_0_0_0_0_0_0_0_0_0_0_1.409	2	2	2	2	2	2	2	2	1	3	1	1	1
1514	7	24,090,675	24,178,060	3	2	0_0_0_0_0_0_0_0_0_0_0_0_1.409	2	2	2	2	2	2	2	2	1	3	1	1	1
1515	7	24,178,061	26,825,555	2	1	0_0_0_0_0_0_0_0_0_0_0_0_1.409	1	1	1	1	1	1	1	1	1	2	1	1	1
1516	7	26,825,556	32,283,829	1	0														
1517	7	32,283,830	35,302,576	2	1	0_0_0_0_0_0_0_0_0_0_0_0_1.355	1	1	1	1	1	1	1	1	1	2	1	1	1
1518	7	35,302,577	35,308,327	3	2	0_0_0_0_0_0_0_0_0_0_0_0_1.355	2	2	2	1	1	1	1	1	1	3	1	1	1
1519	7	35,308,328	35,343,841	3	3	0_0_0_0_0_0_0_0_0_0_0_0_1.355	2	2	2	1	1	1	1	1	1	3	1	1	1
1520	7	35,343,842	35,345,695	3	2	0_1_0_0_0_0_0_0_0_1_0_1_0.103	3	3	3	2	2	2	2	2	2	1	2	2	2
1521	7	35,345,696	35,434,652	2	1	1_0_1_1_1_1_1_1_1_0_1_0_0.125	1	1	1	2	2	2	2	2	2	1	2	2	2
1522	7	35,434,653	35,434,890	1	0														
1523	7	35,434,891	35,935,004	2	1	1_0_1_1_1_0_1_0_0_0_1_0_0.398	1	1	1	2	2	2	2	2	2	1	1	1	1

1524	7	35,935,005	35,941,998	3	2	0_0_0_0_0_0_0_0_0_0_0_0_1.271	1	1	1	2	2	2	2	2	2	3	1	1	1
1525	7	35,941,999	36,156,634	3	3	0_0_0_0_0_0_0_0_0_0_0_0_1.271	2	2	2	1	1	1	1	1	1	3	2	2	2
1526	7	36,156,635	36,189,803	3	2	0_0_0_0_0_0_0_0_0_0_0_0_1.271	1	1	1	2	2	2	2	2	2	3	1	1	1
1527	7	36,189,804	36,196,742	3	3	0_0_0_0_0_0_0_0_0_0_0_0_1.271	2	2	2	1	1	1	1	1	1	3	2	2	2
1528	7	36,196,743	36,197,128	3	2	0_0_0_0_0_0_0_0_0_0_0_0_1.271	1	1	1	2	2	2	2	2	2	3	1	1	1
1529	7	36,197,129	36,237,863	2	1	0_0_0_0_0_0_0_0_0_0_0_0_1.271	1	1	1	1	1	1	1	1	1	2	1	1	1
1530	7	36,237,864	36,239,432	1	0														
1531	7	36,239,433	36,272,753	2	1	1_0_1_1_1_1_1_1_1_0_1_0_0.823	1	1	1	2	2	2	2	2	2	1	2	2	2
1532	7	36,272,754	36,298,857	3	2	0_1_0_0_0_0_0_0_0_1_0_1_0.280	3	3	3	2	2	2	2	2	2	1	2	2	2
1533	7	36,298,858	36,831,326	3	3	0_0_0_0_0_0_0_0_0_0_0_0_1.212	2	2	2	1	1	1	1	1	1	3	1	1	1
1534	7	36,831,327	36,949,742	3	2	0_1_0_0_0_0_0_0_0_1_0_1_0.280	3	3	3	2	2	2	2	2	2	1	2	2	2
1535	7	36,949,743	37,108,025	3	3	0_0_0_0_0_0_0_0_0_0_0_0_1.111	2	2	2	1	1	1	1	1	1	3	1	1	1
1536	7	37,108,026	37,202,940	3	2	0_1_0_0_0_0_0_0_0_1_0_1_0.280	3	3	3	2	2	2	2	2	2	1	2	2	2
1537	7	37,202,941	37,436,770	3	3	0_0_0_0_0_0_0_0_0_0_0_0_1.114	2	2	2	1	1	1	1	1	1	3	1	1	1
1538	7	37,436,771	37,437,408	3	2	0_1_0_0_0_0_0_0_0_1_0_1_0.280	3	3	3	2	2	2	2	2	2	1	2	2	2
1539	7	37,437,409	38,963,708	2	1	0_1_0_0_0_0_0_0_0_1_0_1_0.280	2	2	2	1	1	1	1	1	1	1	1	1	1
1540	7	38,963,709	39,085,732	1	0														
1541	7	39,085,733	39,971,779	2	1	1_1_1_1_1_0_1_0_0_1_1_1_1.483	2	2	2	2	2	2	2	2	2	2	1	1	1
1542	7	39,971,780	40,022,246	1	0														
1543	7	40,022,247	40,628,823	2	1	1_0_1_1_1_1_1_1_1_0_1_0_0.801	1	1	1	2	2	2	2	2	2	1	2	2	2
1544	7	40,628,824	40,631,724	1	0														
1545	7	40,631,725	40,647,623	2	1	0_1_0_0_0_0_0_0_0_1_0_1_0.132	2	2	2	1	1	1	1	1	1	1	1	1	1
1546	7	40,647,624	40,723,009	3	2	0_1_0_0_0_0_0_0_0_1_0_1_0.132	3	3	3	2	2	2	2	2	2	1	1	1	1
1547	7	40,723,010	40,836,816	3	3	0_1_0_0_0_0_0_0_0_1_0_1_0.132	3	3	3	2	2	2	2	2	2	1	1	1	1
1548	7	40,836,817	40,836,844	3	2	0_1_0_0_0_0_0_0_0_1_0_1_0.132	3	3	3	2	2	2	2	2	2	1	1	1	1
1549	7	40,836,845	40,874,578	2	1	0_1_0_0_0_0_0_0_0_1_0_1_0.132	2	2	2	1	1	1	1	1	1	1	1	1	1
1550	7	40,874,579	40,895,923	1	0														
1551	7	40,895,924	42,347,233	2	1	1_1_1_1_1_0_1_0_0_1_1_1_1.892	2	2	2	2	2	2	2	2	2	2	1	1	1
1552	7	42,347,234	42,348,306	1	0														
1553	7	42,348,307	42,503,029	2	1	1_0_1_1_1_1_1_1_1_0_1_0_0.231	1	1	1	2	2	2	2	2	2	1	2	2	2
1554	7	42,503,030	42,507,846	1	0														
1555	7	42,507,847	42,508,139	2	1	0_1_0_0_0_0_0_0_0_1_0_1_0.121	2	2	2	1	1	1	1	1	1	1	1	1	1
1556	7	42,508,140	42,529,841	3	2	0_1_0_0_0_0_0_0_0_1_0_1_0.121	3	3	3	2	2	2	2	2	2	1	1	1	1
1557	7	42,529,842	42,904,131	3	3	0_1_0_0_0_0_0_0_0_1_0_1_0.121	3	3	3	2	2	2	2	2	2	1	1	1	1
1558	7	42,904,132	42,932,142	3	2	0_1_0_0_0_0_0_0_0_1_0_1_0.121	3	3	3	2	2	2	2	2	2	1	1	1	1
1559	7	42,932,143	43,478,907	3	3	0_1_0_0_0_0_0_0_0_1_0_1_0.121	3	3	3	2	2	2	2	2	2	1	1	1	1
1560	7	43,478,908	43,479,252	3	2	0_1_0_0_0_0_0_0_0_1_0_1_0.121	3	3	3	2	2	2	2	2	2	1	1	1	1
1561	7	43,479,253	43,523,102	2	1	1_0_1_1_1_0_1_0_0_0_1_0_0.430	1	1	1	2	2	2	2	2	2	1	1	1	1
1562	7	43,523,103	43,523,893	3	2	0_1_0_0_0_0_0_0_0_1_0_1_0.734	3	3	3	2	2	2	2	2	2	1	1	1	1
1563	7	43,523,894	43,667,726	3	3	0_1_0_0_0_0_0_0_0_1_0_1_0.734	3	3	3	2	2	2	2	2	2	1	1	1	1
1564	7	43,667,727	43,667,789	3	2	0_1_0_0_0_0_0_0_0_1_0_1_0.734	3	3	3	2	2	2	2	2	2	1	1	1	1
1565	7	43,667,790	44,000,877	2	1	0_1_0_0_0_0_0_0_0_1_0_1_0.734	2	2	2	1	1	1	1	1	1	1	1	1	1
1566	7	44,000,878	47,300,158	1	0														
1567	7	47,300,159	47,702,490	2	1	1_0_1_1_1_1_1_1_1_1_1_1_1.107	1	2	2	2	2	2	2	2	2	2	2	2	2
1568	7	47,702,491	47,703,641	1	0														
1569	7	47,703,642	48,161,983	2	1	1_1_1_1_1_0_1_0_0_1_1_1_0.347	2	2	2	2	2	2	2	2	2	1	1	1	1
1570	7	48,161,984	48,196,370	1	0														
1571	7	48,196,371	48,612,724	2	1	1_0_1_1_1_0_1_0_0_1_1_1_0.565	1	2	2	2	2	2	2	2	2	1	1	1	1
1572	7	48,612,725	48,627,545	1	0														
1573	7	48,627,546	48,778,038	2	1	1_0_1_1_1_1_1_1_1_1_1_1_1.25	1	2	2	2	2	2	2	2	2	2	2	2	2
1574	7	48,778,039	54,200,096	1	0														
1575	7	54,200,097	54,415,788	2	1	0_0_0_0_0_0_0_0_0_0_0_0_1.220	1	1	1	1	1	1	1	1	1	2	1	1	1
1576	7	54,415,789	59,009,309	1	0														
1577	7	59,009,310	62,838,579	2	1	0_0_0_0_0_0_0_0_0_0_0_0_1.228	1	1	1	1	1	1	1	1	1	2	1	1	1
1578	7	62,838,580	64,663,241	1	0														
1579	7	64,663,242	64,958,606	2	1	0_0_0_0_0_0_0_0_0_0_0_0_1.603	1	1	1	1	1	1	1	1	1	2	1	1	1
1580	7	64,958,607	65,928,396	3	2	0_0_0_0_0_0_0_0_0_0_0_0_1.603	2	2	2	2	2	2	2	2	2	3	1	1	1
1581	7	65,928,397	67,234,927	2	1	1_1_1_1_1_0_1_0_0_1_1_1_0.107	2	2	2	2	2	2	2	2	2	1	1	1	1
1582	7	67,234,928	67,379,433	1	0														
1583	7	67,379,434	67,380,307	2	1	1_1_1_1_1_0_1_0_0_1_1_1_0.483	2	2	2	2	2	2	2	2	2	1	1	1	1
1584	7	67,380,308	67,508,916	3	2	0_0_0_0_0_0_0_0_0_0_0_0_1.306	2	2	2	2	2	2	2	2	2	3	1	1	1
1585	7	67,508,917	67,641,122	3	3	0_0_0_0_0_0_0_0_0_0_0_0_1.306	2	2	2	2	2	2	2	2	2	3	1	1	1
1586	7	67,641,123	68,018,324	3	2	0_0_0_0_0_0_0_0_0_0_0_0_1.306	2	2	2	2	2	2	2	2	2	3	1	1	1
1587	7	68,018,325	68,021,369	2	1	1_1_1_1_1_0_1_0_0_1_1_1_0.483	2	2	2	2	2	2	2	2	2	1	1	1	1
1588	7	68,021,370	68,305,336	3	2	0_0_0_0_0_0_0_1_0_0_0_0_1.171	2	2	2	2	2	2	2	2	2	3	1	3	1
1589	7	68,305,337	68,444,159	3	3	0_0_0_0_0_0_0_1_0_0_0_0_1.171	2	2	2	2	2	2	2	2	2	3	1	3	1
1590	7	68,444,160	68,447,998	3	2	0_0_0_0_0_0_0_1_0_0_0_0_1.171	2	2	2	2	2	2	2	2	2	3	1	3	1
1591	7	68,447,999	68,497,677	2	1	0_0_0_0_0_0_0_1_0_0_0_0_1.171	1	1	1	1	1	1	1	1	1	2	1	2	1
1592	7	68,497,678	71,417,927	3	2	0_0_0_0_0_0_0_1_0_0_0_0_1.171	2	2	2	2	2	2	2	2	2	3	1	3	1
1593	7	71,417,928	71,440,391	2	1	0_0_0_0_0_0_0_1_0_0_0_0_1.171	1	1	1	1	1	1	1	1	1	2	1	2	1
1594	7	71,440,392	71,449,197	1	0														
1595	7	71,449,198	77,932,233	2	1	1_1_1_1_1_0_1_1_0_1_1_1_1.105	2	2	2	2	2	2	2	2	2	2	1	2	1
1596	7	77,932,234	82,015,968	1	0														
1597	7	82,015,969	86,669,663	2	1	1_1_1_1_1_0_1_0_0_1_1_1_0.464	2	2	2	2	2	2	2	2	2	1	1	1	1
1598	7	86,669,664	86,848,283	1	0														
1599	7	86,848,284	90,303,141	2	1	1_1_1_1_1_0_1_0_0_1_1_1_0.318	2	2	2	2	2	2	2	2	2	1	1	1	1
1600	7	90,303,142	90,703,861	1	0														
1601	7	90,703,862	91,206,569	2	1	1_1_1_1_1_0_1_0_0_1_1_1_0.362	2	2	2	2	2	2	2	2	2	1	1	1	1
1602	7	91,206,570	91,216,056	1	0														
1603	7	91,216,057	91,355,286	2	1	1_1_1_1_1_0_0_0_0_1_1_1_0.151	2	2	2	2	2	2	2	2	1	1	1	1	1
1604	7	91,355,287	91,373,653	1	0														
1605	7	91,373,654	93,885,682	2	1	1_1_1_1_1_0_1_0_0_1_1_1_0.103	2	2	2	2	2	2	2	2	2	1	1	1	1
1606	7	93,885,683	93,888,190	1	0														
1607	7	93,888,191	95,254,876	2	1	0_0_0_0_0_1_0_0_1_0_0_0_1.150	1	1	1	1	1	1	1	1	1	2	2	1	2
1608	7	95,254,877	95,262,491	1	0														

Disease Models & Mechanisms • Supplementary information

1609	7	95,262,492	95,894,192	2	1	1_1_1_1_1_1_1_0_1_1_1_1_1.686	2	2	2	2	2	2	2	2	2	2	2	1	2
1610	7	95,894,193	95,993,003	1	0														
1611	7	95,993,004	96,495,336	2	1	1_1_1_1_1_0_1_0_0_1_1_1_0.288	2	2	2	2	2	2	2	2	2	1	1	1	1
1612	7	96,495,337	97,180,348	1	0														
1613	7	97,180,349	100,953,954	2	1	1_1_1_1_1_0_1_0_0_1_1_1_0.332	2	2	2	2	2	2	2	2	2	1	1	1	1
1614	7	100,953,955	101,422,283	1	0														
1615	7	101,422,284	103,129,349	2	1	1_1_1_1_1_1_1_0_1_1_1_1_1.610	2	2	2	2	2	2	2	2	2	2	2	1	2
1616	7	103,129,350	103,130,040	1	0														
1617	7	103,130,041	104,350,446	2	1	0_0_0_0_0_0_0_1_0_0_0_0_1.100	1	1	1	1	1	1	1	1	1	2	1	2	1
1618	7	104,350,447	104,350,952	1	0														
1619	7	104,350,953	105,359,511	2	1	1_1_1_1_1_0_0_0_0_1_1_1_0.675	2	2	2	2	2	2	2	2	1	1	1	1	1
1620	7	105,359,512	105,722,378	1	0														
1621	7	105,722,379	105,865,877	2	1	1_1_1_1_1_0_0_0_0_1_1_1_0.223	2	2	2	2	2	2	2	2	1	1	1	1	1
1622	7	105,865,878	105,867,543	1	0														
1623	7	105,867,544	108,417,019	2	1	1_1_1_1_1_0_0_0_0_1_1_1_1.160	2	2	2	2	2	2	2	2	1	2	1	1	1
1624	7	108,417,020	109,472,857	1	0														
1625	7	109,472,858	111,999,557	2	1	0_0_0_0_0_0_0_0_0_0_0_0_1.297	1	1	1	1	1	1	1	1	1	2	1	1	1
1626	7	111,999,558	116,729,268	1	0														
1627	7	116,729,269	117,888,121	2	1	1_1_1_1_1_0_0_0_0_1_1_1_0.606	2	2	2	2	2	2	2	2	1	1	1	1	1
1628	7	117,888,122	118,094,646	1	0														
1629	7	118,094,647	119,434,705	2	1	1_1_1_1_1_0_0_0_0_1_1_1_0.708	2	2	2	2	2	2	2	2	1	1	1	1	1
1630	7	119,434,706	119,435,063	1	0														
1631	7	119,435,064	120,420,069	2	1	1_1_1_1_1_0_1_0_0_1_1_1_0.908	2	2	2	2	2	2	2	2	2	1	1	1	1
1632	7	120,420,070	123,889,362	1	0														
1633	7	123,889,363	124,920,911	2	1	1_0_1_1_1_0_1_0_0_0_1_0_0.938	1	1	1	2	2	2	2	2	2	1	1	1	1
1634	7	124,920,912	127,750,887	1	0														
1635	7	127,750,888	128,096,402	2	1	1_0_1_1_1_0_1_0_0_0_1_0_0.617	1	1	1	2	2	2	2	2	2	1	1	1	1
1636	7	128,096,403	128,097,655	1	0														
1637	7	128,097,656	128,376,397	2	1	1_1_1_1_1_0_1_0_0_1_1_1_0.358	2	2	2	2	2	2	2	2	2	1	1	1	1
1638	7	128,376,398	128,685,800	1	0														
1639	7	128,685,801	129,147,552	2	1	1_0_1_1_1_0_1_0_0_1_1_1_0.669	1	2	2	2	2	2	2	2	2	1	1	1	1
1640	7	129,147,553	129,945,484	1	0														
1641	7	129,945,485	132,244,882	2	1	1_0_1_1_1_0_1_0_0_1_1_1_0.302	1	2	2	2	2	2	2	2	2	1	1	1	1
1642	7	132,244,883	132,626,808	1	0														
1643	7	132,626,809	132,761,909	2	1	1_0_1_1_1_0_1_0_0_1_1_1_0.132	1	2	2	2	2	2	2	2	2	1	1	1	1
1644	7	132,761,910	133,213,600	1	0														
1645	7	133,213,601	133,233,176	2	1	1_1_1_1_1_0_1_0_0_1_1_1_1.70	2	2	2	2	2	2	2	2	2	2	1	1	1
1646	7	133,233,177	133,258,459	3	2	1_0_1_1_1_1_1_1_1_0_1_0_0.45	1	1	1	3	3	3	3	3	3	1	2	2	2
1647	7	133,258,460	133,310,041	2	1	1_1_1_1_1_0_1_0_0_1_1_1_1.70	2	2	2	2	2	2	2	2	2	2	1	1	1
1648	7	133,310,042	133,314,301	3	2	1_0_1_1_1_1_1_1_1_0_1_0_0.236	1	1	1	3	3	3	3	3	3	1	2	2	2
1649	7	133,314,302	133,378,322	3	3	1_0_1_1_1_0_1_0_0_0_1_0_0.184	1	1	1	3	3	3	3	3	3	1	2	2	2
1650	7	133,378,323	133,522,605	3	2	1_0_1_1_1_0_1_0_0_0_1_0_0.184	1	1	1	3	3	3	3	3	3	1	2	2	2
1651	7	133,522,606	133,540,594	3	3	1_0_1_1_1_0_1_0_0_0_1_0_0.184	1	1	1	3	3	3	3	3	3	1	2	2	2
1652	7	133,540,595	133,542,244	3	2	1_0_1_1_1_1_1_1_1_0_1_0_0.236	1	1	1	3	3	3	3	3	3	1	2	2	2
1653	7	133,542,245	134,387,986	2	1	1_1_1_1_1_0_1_0_0_1_1_1_1.725	2	2	2	2	2	2	2	2	2	2	1	1	1
1654	7	134,387,987	134,389,095	1	0														
1655	7	134,389,096	134,399,093	2	1	0_0_0_0_0_0_0_0_0_0_0_0_1.86	1	1	1	1	1	1	1	1	1	2	1	1	1
1656	7	134,399,094	134,405,988	3	2	0_0_0_0_0_0_0_0_0_0_0_0_1.86	2	2	2	2	2	2	2	2	1	3	1	1	1
1657	7	134,405,989	134,713,132	3	3	0_0_0_0_0_0_0_0_0_0_0_0_1.86	2	2	2	2	2	2	2	2	1	3	1	1	1
1658	7	134,713,133	134,752,354	3	2	1_1_1_1_1_0_0_0_0_1_1_1_0.343	3	3	3	3	3	3	3	3	1	2	1	1	1
1659	7	134,752,355	134,865,057	2	1	1_1_1_1_1_0_0_0_0_1_1_1_0.343	2	2	2	2	2	2	2	2	1	1	1	1	1
1660	7	134,865,058	134,872,630	3	2	0_0_0_0_0_0_0_0_0_0_0_0_1.301	2	2	2	2	2	2	2	2	1	3	1	1	1
1661	7	134,872,631	134,905,835	3	3	0_0_0_0_0_0_0_0_0_0_0_0_1.301	2	2	2	2	2	2	2	2	1	3	1	1	1
1662	7	134,905,836	134,957,685	3	2	0_0_0_0_0_0_0_0_0_0_0_0_1.301	2	2	2	2	2	2	2	2	1	3	1	1	1
1663	7	134,957,686	135,334,350	2	1	0_0_0_0_0_0_0_0_0_0_0_0_1.301	1	1	1	1	1	1	1	1	1	2	1	1	1
1664	7	135,334,351	135,428,720	1	0														
1665	7	135,428,721	135,571,000	2	1	0_0_0_0_0_0_0_0_0_0_0_0_1.478	1	1	1	1	1	1	1	1	1	2	1	1	1
1666	7	135,571,001	135,731,922	3	2	0_0_0_0_0_0_0_0_0_0_0_0_1.478	2	2	2	2	2	2	2	2	1	3	1	1	1
1667	7	135,731,923	135,732,660	2	1	0_0_0_0_0_0_0_0_0_0_0_0_1.478	1	1	1	1	1	1	1	1	1	2	1	1	1
1668	7	135,732,661	135,734,774	1	0														
1669	7	135,734,775	136,472,092	2	1	1_1_1_1_1_0_0_0_0_1_1_1_1.591	2	2	2	2	2	2	2	2	1	2	1	1	1
1670	7	136,472,093	136,723,970	1	0														
1671	7	136,723,971	137,110,377	2	1	0_0_0_0_0_0_0_0_0_0_0_0_1.103	1	1	1	1	1	1	1	1	1	2	1	1	1
1672	7	137,110,378	137,110,544	3	2	0_0_0_0_0_0_0_0_0_0_0_0_1.103	2	2	2	2	2	2	2	2	1	3	1	1	1
1673	7	137,110,545	137,304,896	3	3	0_0_0_0_0_0_0_0_0_0_0_0_1.103	2	2	2	2	2	2	2	2	1	3	1	1	1
1674	7	137,304,897	137,336,583	3	2	0_0_0_0_0_0_0_0_0_0_0_0_1.103	2	2	2	2	2	2	2	2	1	3	1	1	1
1675	7	137,336,584	137,684,595	3	3	0_0_0_0_0_0_0_0_0_0_0_0_1.103	2	2	2	2	2	2	2	2	1	3	1	1	1
1676	7	137,684,596	137,750,938	3	2	0_0_0_0_0_0_0_0_0_0_0_0_1.103	2	2	2	2	2	2	2	2	1	3	1	1	1
1677	7	137,750,939	137,905,271	3	3	0_0_0_0_0_0_0_0_0_0_0_0_1.103	2	2	2	2	2	2	2	2	1	3	1	1	1
1678	7	137,905,272	137,908,864	3	2	0_0_0_0_0_0_0_0_0_0_0_0_1.103	2	2	2	2	2	2	2	2	1	3	1	1	1
1679	7	137,908,865	137,979,666	2	1	1_1_1_1_1_0_0_0_0_1_1_1_0.104	2	2	2	2	2	2	2	2	1	1	1	1	1
1680	7	137,979,667	138,043,256	3	2	0_0_0_0_0_0_0_0_0_0_0_0_1.39	2	2	2	2	2	2	2	2	1	3	1	1	1
1681	7	138,043,257	138,237,453	3	3	0_0_0_0_0_0_0_0_0_0_0_0_1.39	2	2	2	2	2	2	2	2	1	3	1	1	1
1682	7	138,237,454	138,240,972	3	2	1_1_1_1_1_0_0_0_0_1_1_1_0.104	3	3	3	3	3	3	3	3	1	2	1	1	1
1683	7	138,240,973	138,397,739	2	1	1_1_1_1_1_0_0_0_0_1_1_1_0.104	2	2	2	2	2	2	2	2	1	1	1	1	1
1684	7	138,397,740	138,400,169	3	2	0_0_0_0_0_0_0_0_0_0_0_0_1.154	2	2	2	2	2	2	2	2	1	3	1	1	1
1685	7	138,400,170	138,453,576	3	3	0_0_0_0_0_0_0_0_0_0_0_0_1.154	2	2	2	2	2	2	2	2	1	3	1	1	1
1686	7	138,453,577	138,490,587	3	2	0_0_0_0_0_0_0_0_0_0_0_0_1.154	2	2	2	2	2	2	2	2	1	3	1	1	1
1687	7	138,490,588	138,576,569	3	3	0_0_0_0_0_0_0_0_0_0_0_0_1.154	2	2	2	2	2	2	2	2	1	3	1	1	1
1688	7	138,576,570	138,633,869	3	2	1_1_1_1_1_0_0_0_0_1_1_1_0.104	3	3	3	3	3	3	3	3	1	2	1	1	1
1689	7	138,633,870	139,988,200	2	1	1_1_1_1_1_0_0_0_0_1_1_1_1.981	2	2	2	2	2	2	2	2	1	2	1	1	1
1690	7	139,988,201	140,102,568	1	0														
1691	7	140,102,569	143,418,643	2	1	1_1_1_1_1_0_0_0_0_1_1_1_1.166	2	2	2	2	2	2	2	2	1	2	1	1	1
1692	7	143,418,644	143,420,666	1	0														
1693	7	143,420,667	143,534,159	2	1	0_0_0_0_0_0_0_0_0_0_0_0_1.155	1	1	1	1	1	1	1	1	1	2	1	1	1

1694	7	143,534,160	143,672,273	1	0														
1695	7	143,672,274	143,708,411	2	1	0_0_0_0_0_0_0_0_0_0_0_0_1.45	1	1	1	1	1	1	1	1	1	2	1	1	1
1696	7	143,708,412	143,708,859	1	0														
1697	7	143,708,860	144,523,314	2	1	1_1_1_1_1_0_0_0_0_1_1_1_0.487	2	2	2	2	2	2	2	2	1	1	1	1	1
1698	7	144,523,315	144,686,177	3	2	1_1_1_1_1_0_0_0_0_1_1_1_0.487	3	3	3	3	3	3	3	3	1	2	1	1	1
1699	7	144,686,178	145,092,349	3	3	0_0_0_0_0_0_0_0_0_0_0_0_1.57	2	2	2	2	2	2	2	2	1	3	1	1	1
1700	7	145,092,350	145,268,031	3	2	1_1_1_1_1_0_0_0_0_1_1_1_0.487	3	3	3	3	3	3	3	3	1	2	1	1	1
1701	7	145,268,032	145,647,795	3	3	0_0_0_0_0_0_0_0_0_0_0_0_1.28	2	2	2	2	2	2	2	2	1	3	1	1	1
1702	7	145,647,796	145,716,083	3	2	0_0_0_0_0_0_0_0_0_0_0_0_1.28	2	2	2	2	2	2	2	2	1	3	1	1	1
1703	7	145,716,084	145,720,861	2	1	0_0_0_0_0_0_0_0_0_0_0_0_1.28	1	1	1	1	1	1	1	1	1	2	1	1	1
1704	8	19,614	3,521,260	2	1	1_1_1_1_1_0_1_0_0_1_1_1_0.251	2	2	2	2	2	2	2	2	2	1	1	1	1
1705	8	3,521,261	3,527,343	1	0														
1706	8	3,527,344	3,936,668	2	1	0_0_0_0_0_1_1_1_1_1_0_1_1.475	1	2	2	1	1	1	1	1	2	2	2	2	2
1707	8	3,936,669	3,937,619	1	0														
1708	8	3,937,620	4,283,847	2	1	1_0_1_1_1_1_1_1_1_1_1_1_1.493	1	2	2	2	2	2	2	2	2	2	2	2	2
1709	8	4,283,848	4,430,855	1	0														
1710	8	4,430,856	8,763,804	2	1	1_1_1_1_1_0_1_0_0_1_1_1_0.476	2	2	2	2	2	2	2	2	2	1	1	1	1
1711	8	8,763,805	9,394,974	1	0														
1712	8	9,394,975	13,677,352	2	1	1_1_1_1_1_0_1_0_0_1_1_1_0.442	2	2	2	2	2	2	2	2	2	1	1	1	1
1713	8	13,677,353	13,678,952	1	0														
1714	8	13,678,953	14,727,372	2	1	1_1_1_1_1_0_0_0_0_1_1_1_0.145	2	2	2	2	2	2	2	2	1	1	1	1	1
1715	8	14,727,373	14,734,280	1	0														
1716	8	14,734,281	15,072,111	2	1	0_0_0_0_0_0_0_0_0_0_0_0_1.464	1	1	1	1	1	1	1	1	1	2	1	1	1
1717	8	15,072,112	15,159,228	3	2	0_0_0_0_0_0_0_0_0_0_0_0_1.464	2	2	2	2	2	2	2	2	1	3	1	1	1
1718	8	15,159,229	15,240,304	2	1	1_1_1_1_1_0_0_0_0_1_1_1_0.28	2	2	2	2	2	2	2	2	1	1	1	1	1
1719	8	15,240,305	15,254,453	3	2	1_1_1_1_1_0_0_0_0_1_1_1_0.28	3	3	3	3	3	3	3	3	1	2	1	1	1
1720	8	15,254,454	15,357,355	2	1	1_1_1_1_1_0_0_0_0_1_1_1_1.280	2	2	2	2	2	2	2	2	1	2	1	1	1
1721	8	15,357,356	15,364,935	3	2	1_1_1_1_1_0_0_0_0_1_1_1_0.53	3	3	3	3	3	3	3	3	1	2	1	1	1
1722	8	15,364,936	15,459,547	3	3	0_0_0_0_0_0_0_0_0_0_0_0_1.23	2	2	2	2	2	2	2	2	1	3	1	1	1
1723	8	15,459,548	16,019,802	3	2	1_1_1_1_1_0_0_0_0_1_1_1_0.53	3	3	3	3	3	3	3	3	1	2	1	1	1
1724	8	16,019,803	16,066,068	2	1	1_1_1_1_1_0_0_0_0_1_1_1_1.280	2	2	2	2	2	2	2	2	1	2	1	1	1
1725	8	16,066,069	16,077,357	3	2	1_1_1_1_1_0_0_0_0_1_1_1_0.803	3	3	3	3	3	3	3	3	1	2	1	1	1
1726	8	16,077,358	16,080,133	3	3	0_0_0_0_0_0_0_0_0_0_0_0_1.75	2	2	2	2	2	2	2	2	1	3	1	1	1
1727	8	16,080,134	16,355,201	3	2	0_0_0_0_0_0_0_0_0_0_0_0_1.75	2	2	2	2	2	2	2	2	1	3	1	1	1
1728	8	16,355,202	16,429,740	2	1	1_1_1_1_1_0_0_0_0_1_1_1_0.803	2	2	2	2	2	2	2	2	1	1	1	1	1
1729	8	16,429,741	16,437,893	3	2	0_0_0_0_0_0_0_0_0_0_0_0_1.33	2	2	2	2	2	2	2	2	1	3	1	1	1
1730	8	16,437,894	16,635,345	3	3	0_0_0_0_0_0_0_0_0_0_0_0_1.33	2	2	2	2	2	2	2	2	1	3	1	1	1
1731	8	16,635,346	16,936,972	3	2	1_1_1_1_1_0_0_0_0_1_1_1_0.803	3	3	3	3	3	3	3	3	1	2	1	1	1
1732	8	16,936,973	16,938,612	2	1	1_1_1_1_1_0_0_0_0_1_1_1_0.803	2	2	2	2	2	2	2	2	1	1	1	1	1
1733	8	16,938,613	17,192,957	3	2	0_0_0_0_0_0_0_0_0_0_0_0_1.200	2	2	2	2	2	2	2	2	1	3	1	1	1
1734	8	17,192,958	17,203,227	2	1	1_1_1_1_1_0_0_0_0_1_1_1_0.803	2	2	2	2	2	2	2	2	1	1	1	1	1
1735	8	17,203,228	17,260,147	3	2	1_1_1_1_1_0_0_0_0_1_1_1_0.803	3	3	3	3	3	3	3	3	1	2	1	1	1
1736	8	17,260,148	18,082,662	3	3	0_0_0_0_0_0_0_0_0_0_0_0_1.201	2	2	2	2	2	2	2	2	1	3	1	1	1
1737	8	18,082,663	18,202,860	3	2	0_0_0_0_0_0_0_0_0_0_0_0_1.201	2	2	2	2	2	2	2	2	1	3	1	1	1
1738	8	18,202,861	18,506,418	3	3	0_0_0_0_0_0_0_0_0_0_0_0_1.201	2	2	2	2	2	2	2	2	1	3	1	1	1
1739	8	18,506,419	18,632,056	3	2	0_0_0_0_0_0_0_0_0_0_0_0_1.201	2	2	2	2	2	2	2	2	1	3	1	1	1
1740	8	18,632,057	18,842,296	2	1	1_1_1_1_1_0_0_0_0_1_1_1_1.722	2	2	2	2	2	2	2	2	1	2	1	1	1
1741	8	18,842,297	18,885,684	3	2	1_1_1_1_1_0_0_0_0_1_1_1_0.172	3	3	3	3	3	3	3	3	1	2	1	1	1
1742	8	18,885,685	19,288,354	3	3	0_0_0_0_0_0_0_0_0_0_0_0_1.216	2	2	2	2	2	2	2	2	1	3	1	1	1
1743	8	19,288,355	19,506,876	3	2	0_0_0_0_0_0_0_0_0_0_0_0_1.216	2	2	2	2	2	2	2	2	1	3	1	1	1
1744	8	19,506,877	19,567,723	3	3	0_0_0_0_0_0_0_0_0_0_0_0_1.216	2	2	2	2	2	2	2	2	1	3	1	1	1
1745	8	19,567,724	20,576,786	3	2	0_0_0_0_0_0_0_0_0_0_0_0_1.216	2	2	2	2	2	2	2	2	1	3	1	1	1
1746	8	20,576,787	20,653,755	3	3	0_0_0_0_0_0_0_0_0_0_0_0_1.216	2	2	2	2	2	2	2	2	1	3	1	1	1
1747	8	20,653,756	20,679,271	3	2	0_0_0_0_0_0_0_0_0_0_0_0_1.216	2	2	2	2	2	2	2	2	1	3	1	1	1
1748	8	20,679,272	20,716,202	3	3	0_0_0_0_0_0_0_0_0_0_0_0_1.216	2	2	2	2	2	2	2	2	1	3	1	1	1
1749	8	20,716,203	20,727,501	3	2	1_1_1_1_1_0_0_0_0_1_1_1_0.172	3	3	3	3	3	3	3	3	1	2	1	1	1
1750	8	20,727,502	21,165,530	3	3	0_0_0_0_0_0_0_0_0_0_0_0_1.40	2	2	2	2	2	2	2	2	1	3	1	1	1
1751	8	21,165,531	21,203,249	3	2	1_1_1_1_1_0_0_0_0_1_1_1_0.172	3	3	3	3	3	3	3	3	1	2	1	1	1
1752	8	21,203,250	21,228,026	3	3	0_0_0_0_0_0_0_0_0_0_0_0_1.292	2	2	2	2	2	2	2	2	1	3	1	1	1
1753	8	21,228,027	21,243,082	3	2	0_0_0_0_0_0_0_0_0_0_0_0_1.292	2	2	2	2	2	2	2	2	1	3	1	1	1
1754	8	21,243,083	22,041,485	3	3	0_0_0_0_0_0_0_0_0_0_0_0_1.292	2	2	2	2	2	2	2	2	1	3	1	1	1
1755	8	22,041,486	22,043,245	3	2	0_0_0_0_0_0_0_0_0_0_0_0_1.292	2	2	2	2	2	2	2	2	1	3	1	1	1
1756	8	22,043,246	22,165,969	2	1	0_0_0_0_0_0_0_0_0_0_0_0_1.292	1	1	1	1	1	1	1	1	1	2	1	1	1
1757	8	22,165,970	22,208,442	3	2	0_0_0_0_0_0_0_0_0_0_0_0_1.292	2	2	2	2	2	2	2	2	1	3	1	1	1
1758	8	22,208,443	23,153,950	3	3	0_0_0_0_0_0_0_0_0_0_0_0_1.292	2	2	2	2	2	2	2	2	1	3	1	1	1
1759	8	23,153,951	23,206,482	3	2	0_0_0_0_0_0_0_0_0_0_0_0_1.292	2	2	2	2	2	2	2	2	1	3	1	1	1
1760	8	23,206,483	24,339,806	2	1	0_0_0_0_0_0_0_0_0_0_0_0_1.292	1	1	1	1	1	1	1	1	1	2	1	1	1
1761	8	24,339,807	24,354,302	3	2	0_0_0_0_0_0_0_0_0_0_0_0_1.292	2	2	2	2	2	2	2	2	1	3	1	1	1
1762	8	24,354,303	24,738,018	2	1	1_1_1_1_1_0_0_0_0_1_1_1_1.489	2	2	2	2	2	2	2	2	1	2	1	1	1
1763	8	24,738,019	24,739,573	1	0														
1764	8	24,739,574	25,319,049	2	1	0_0_0_0_0_0_0_0_0_0_0_0_1.754	1	1	1	1	1	1	1	1	1	2	1	1	1
1765	8	25,319,050	25,989,437	1	0														
1766	8	25,989,438	26,613,875	2	1	1_1_1_1_1_0_0_0_0_1_1_1_1.479	2	2	2	2	2	2	2	2	1	2	1	1	1
1767	8	26,613,876	26,778,295	1	0														
1768	8	26,778,296	27,759,634	2	1	1_1_1_1_1_0_0_0_0_1_1_1_0.136	2	2	2	2	2	2	2	2	1	1	1	1	1
1769	8	27,759,635	28,943,906	3	2	0_0_0_0_0_0_0_0_0_0_0_0_1.618	2	2	2	2	2	2	2	2	1	3	1	1	1
1770	8	28,943,907	29,063,775	2	1	0_0_0_0_0_0_0_0_0_0_0_0_1.618	1	1	1	1	1	1	1	1	1	2	1	1	1
1771	8	29,063,776	29,309,481	3	2	0_0_0_0_0_0_0_0_0_0_0_0_1.618	2	2	2	2	2	2	2	2	1	3	1	1	1
1772	8	29,309,482	29,360,148	2	1	0_0_0_0_0_0_0_0_0_0_0_0_1.618	1	1	1	1	1	1	1	1	1	2	1	1	1
1773	8	29,360,149	29,386,165	3	2	0_0_0_0_0_0_0_0_0_0_0_0_1.618	2	2	2	2	2	2	2	2	1	3	1	1	1
1774	8	29,386,166	30,186,467	2	1	1_1_1_1_1_0_0_0_0_1_1_1_0.109	2	2	2	2	2	2	2	2	1	1	1	1	1
1775	8	30,186,468	30,190,610	3	2	0_0_0_0_0_0_0_0_0_0_0_0_1.633	2	2	2	2	2	2	2	2	1	3	1	1	1
1776	8	30,190,611	30,235,642	3	3	0_0_0_0_0_0_0_0_0_0_0_0_1.633	2	2	2	2	2	2	2	2	1	3	1	1	1
1777	8	30,235,643	30,253,972	3	2	0_0_0_0_0_0_0_0_0_0_0_0_1.633	2	2	2	2	2	2	2	2	1	3	1	1	1
1778	8	30,253,973	30,631,002	2	1	0_0_0_0_0_0_0_0_0_0_0_0_1.633	1	1	1	1	1	1	1	1	1	2	1	1	1

Disease Models & Mechanisms • Supplementary information

1779	8	30,631,003	30,650,686	3	2	0_0_0_0_0_0_0_0_0_0_0_0_1.633	2	2	2	2	2	2	2	2	1	3	1	1	1
1780	8	30,650,687	30,754,785	3	3	0_0_0_0_0_0_0_0_0_0_0_0_1.633	2	2	2	2	2	2	2	2	1	3	1	1	1
1781	8	30,754,786	30,764,947	3	2	0_0_0_0_0_0_0_0_0_0_0_0_1.633	2	2	2	2	2	2	2	2	1	3	1	1	1
1782	8	30,764,948	30,864,639	2	1	0_0_0_0_0_0_0_0_0_0_0_0_1.633	1	1	1	1	1	1	1	1	1	2	1	1	1
1783	8	30,864,640	30,904,624	3	2	0_0_0_0_0_0_0_0_0_0_0_0_1.633	2	2	2	2	2	2	2	2	1	3	1	1	1
1784	8	30,904,625	30,951,300	3	3	0_0_0_0_0_0_0_0_0_0_0_0_1.633	2	2	2	2	2	2	2	2	1	3	1	1	1
1785	8	30,951,301	30,951,958	3	2	0_0_0_0_0_0_0_0_0_0_0_0_1.633	2	2	2	2	2	2	2	2	1	3	1	1	1
1786	8	30,951,959	30,987,811	2	1	1_1_1_1_1_0_0_0_0_1_1_1_1.176	2	2	2	2	2	2	2	2	1	2	1	1	1
1787	8	30,987,812	31,088,220	3	2	1_1_1_1_1_0_0_0_0_1_1_1_0.121	3	3	3	3	3	3	3	3	1	2	1	1	1
1788	8	31,088,221	31,221,195	3	3	0_0_0_0_0_0_0_0_0_0_0_0_1.63	2	2	2	2	2	2	2	2	1	3	1	1	1
1789	8	31,221,196	31,223,507	3	2	0_0_0_0_0_0_0_0_0_0_0_0_1.63	2	2	2	2	2	2	2	2	1	3	1	1	1
1790	8	31,223,508	32,796,368	2	1	1_1_1_1_1_0_0_0_0_1_1_1_1.176	2	2	2	2	2	2	2	2	1	2	1	1	1
1791	8	32,796,369	33,620,326	1	0														
1792	8	33,620,327	33,735,516	2	1	0_1_0_0_0_1_1_1_1_1_1_1_1.20	2	2	2	1	1	1	1	2	2	2	2	2	2
1793	8	33,735,517	33,874,709	1	0														
1794	8	33,874,710	35,527,805	2	1	0_1_0_0_0_1_1_1_1_1_1_1_1.923	2	2	2	1	1	1	1	2	2	2	2	2	2
1795	8	35,527,806	38,750,468	1	0														
1796	8	38,750,469	39,044,816	2	1	0_0_0_0_0_0_0_0_0_0_1_0_0.271	1	1	1	1	1	1	1	2	1	1	1	1	1
1797	8	39,044,817	47,196,166	1	0														
1798	8	47,196,167	47,871,973	2	1	0_1_0_0_0_1_1_1_1_1_1_1_1.271	2	2	2	1	1	1	1	2	2	2	2	2	2
1799	8	47,871,974	48,012,312	1	0														
1800	8	48,012,313	49,201,764	2	1	0_1_0_0_0_1_1_1_1_1_1_1_1.457	2	2	2	1	1	1	1	2	2	2	2	2	2
1801	8	49,201,765	49,627,601	1	0														
1802	8	49,627,602	51,283,588	2	1	0_1_0_0_0_1_1_1_1_1_1_1_1.954	2	2	2	1	1	1	1	2	2	2	2	2	2
1803	8	51,283,589	51,303,470	3	2	0_1_0_0_0_0_0_0_0_1_1_1_1.229	3	3	3	1	1	1	1	3	2	3	2	2	2
1804	8	51,303,471	51,821,976	2	1	0_1_0_0_0_0_0_0_0_1_1_1_1.229	2	2	2	1	1	1	1	2	1	2	1	1	1
1805	8	51,821,977	54,122,109	1	0														
1806	8	54,122,110	61,273,712	2	1	1_1_1_1_1_0_0_0_0_1_1_1_1.337	2	2	2	2	2	2	2	2	1	2	1	1	1
1807	8	61,273,713	61,361,210	1	0														
1808	8	61,361,211	63,080,412	2	1	1_1_1_1_1_0_0_0_0_1_1_1_1.703	2	2	2	2	2	2	2	2	1	2	1	1	1
1809	8	63,080,413	63,160,504	1	0														
1810	8	63,160,505	63,612,844	2	1	1_1_1_1_1_0_0_0_0_1_1_1_1.291	2	2	2	2	2	2	2	2	1	2	1	1	1
1811	8	63,612,845	63,869,111	3	2	1_1_1_1_1_0_0_0_0_1_1_1_0.247	3	3	3	3	3	3	3	3	1	2	1	1	1
1812	8	63,869,112	64,046,156	3	3	0_0_0_0_0_0_0_0_0_0_0_0_1.102	2	2	2	2	2	2	2	2	1	3	1	1	1
1813	8	64,046,157	64,047,652	3	2	1_1_1_1_1_0_0_0_0_1_1_1_0.247	3	3	3	3	3	3	3	3	1	2	1	1	1
1814	8	64,047,653	67,956,473	2	1	1_1_1_1_1_0_0_0_0_1_1_1_1.291	2	2	2	2	2	2	2	2	1	2	1	1	1
1815	8	67,956,474	69,192,050	1	0														
1816	8	69,192,051	69,318,260	2	1	1_1_1_1_1_0_1_0_0_1_1_1_0.259	2	2	2	2	2	2	2	2	2	1	1	1	1
1817	8	69,318,261	70,425,382	1	0														
1818	8	70,425,383	74,490,215	2	1	1_1_1_1_1_0_0_0_0_1_1_1_1.237	2	2	2	2	2	2	2	2	1	2	1	1	1
1819	8	74,490,216	74,665,052	1	0														
1820	8	74,665,053	77,345,956	2	1	1_1_1_1_1_0_0_0_0_1_1_1_0.117	2	2	2	2	2	2	2	2	1	1	1	1	1
1821	8	77,345,957	80,261,582	1	0														
1822	8	80,261,583	80,484,596	2	1	0_0_0_0_0_0_0_0_0_0_0_0_1.180	1	1	1	1	1	1	1	1	1	2	1	1	1
1823	8	80,484,597	81,012,895	1	0														
1824	8	81,012,896	83,461,535	2	1	0_0_0_0_0_0_0_0_0_0_0_0_1.174	1	1	1	1	1	1	1	1	1	2	1	1	1
1825	8	83,461,536	86,592,086	1	0														
1826	8	86,592,087	86,623,757	2	1	1_1_1_1_1_0_0_0_0_1_1_1_0.26	2	2	2	2	2	2	2	2	1	1	1	1	1
1827	8	86,623,758	87,757,304	1	0														
1828	8	87,757,305	88,783,655	2	1	1_1_1_1_1_0_0_0_0_1_1_1_0.102	2	2	2	2	2	2	2	2	1	1	1	1	1
1829	8	88,783,656	88,787,289	1	0														
1830	8	88,787,290	90,660,699	2	1	1_1_1_1_1_0_0_0_0_1_0_1_0.234	2	2	2	2	2	2	2	1	1	1	1	1	1
1831	8	90,660,700	90,661,100	1	0														
1832	8	90,661,101	91,873,434	2	1	1_1_1_1_1_0_0_0_0_1_1_1_0.221	2	2	2	2	2	2	2	2	1	1	1	1	1
1833	8	91,873,435	92,185,994	3	2	0_0_0_0_0_0_0_0_0_0_0_0_1.305	2	2	2	2	2	2	2	2	1	3	1	1	1
1834	8	92,185,995	92,386,823	3	3	0_0_0_0_0_0_0_0_0_0_0_0_1.305	2	2	2	2	2	2	2	2	1	3	1	1	1
1835	8	92,386,824	92,393,054	3	2	0_0_0_0_0_0_0_0_0_0_0_0_1.305	2	2	2	2	2	2	2	2	1	3	1	1	1
1836	8	92,393,055	92,539,855	2	1	1_1_1_1_1_0_0_0_0_1_1_1_1.330	2	2	2	2	2	2	2	2	1	2	1	1	1
1837	8	92,539,856	92,676,213	1	0														
1838	8	92,676,214	93,100,784	2	1	1_1_1_1_1_0_0_0_0_1_1_1_1.483	2	2	2	2	2	2	2	2	1	2	1	1	1
1839	8	93,100,785	93,102,612	1	0														
1840	8	93,102,613	94,848,898	2	1	1_1_1_1_1_0_0_0_0_1_1_1_0.172	2	2	2	2	2	2	2	2	1	1	1	1	1
1841	8	94,848,899	94,899,210	1	0														
1842	8	94,899,211	99,981,386	2	1	0_0_0_0_0_0_0_0_0_0_0_0_1.442	1	1	1	1	1	1	1	1	1	2	1	1	1
1843	8	99,981,387	99,982,214	1	0														
1844	8	99,982,215	102,620,943	2	1	0_0_0_0_0_0_0_1_0_0_0_0_1.410	1	1	1	1	1	1	1	1	1	2	1	2	1
1845	8	102,620,944	102,625,375	3	2	0_0_0_0_0_0_0_1_0_0_0_0_1.410	2	2	2	2	2	2	2	2	1	3	1	3	1
1846	8	102,625,376	102,792,047	3	3	0_0_0_0_0_0_0_1_0_0_0_0_1.410	2	2	2	2	2	2	2	2	1	3	1	3	1
1847	8	102,792,048	102,856,736	3	2	0_0_0_0_0_0_0_1_0_0_0_0_1.410	2	2	2	2	2	2	2	2	1	3	1	3	1
1848	8	102,856,737	102,913,891	3	3	0_0_0_0_0_0_0_1_0_0_0_0_1.410	2	2	2	2	2	2	2	2	1	3	1	3	1
1849	8	102,913,892	102,919,961	3	2	0_0_0_0_0_0_0_1_0_0_0_0_1.410	2	2	2	2	2	2	2	2	1	3	1	3	1
1850	8	102,919,962	102,926,170	2	1	0_0_0_0_0_0_0_1_0_0_0_0_1.410	1	1	1	1	1	1	1	1	1	2	1	2	1
1851	8	102,926,171	120,468,042	1	0														
1852	8	120,468,043	122,564,693	2	1	1_1_1_1_1_0_0_0_0_1_1_1_1.116	2	2	2	2	2	2	2	2	1	2	1	1	1
1853	8	122,564,694	124,332,773	1	0														
1854	8	124,332,774	126,052,611	2	1	1_1_1_1_1_0_0_0_0_1_1_1_0.240	2	2	2	2	2	2	2	2	1	1	1	1	1
1855	8	126,052,612	126,134,545	1	0														
1856	8	126,134,546	129,427,280	2	1	1_1_1_1_1_0_0_0_0_1_1_1_0.300	2	2	2	2	2	2	2	2	1	1	1	1	1
1857	8	129,427,281	129,504,141	1	0														
1858	8	129,504,142	131,077,154	2	1	1_1_1_1_1_0_0_0_0_1_1_1_0.117	2	2	2	2	2	2	2	2	1	1	1	1	1
1859	8	131,077,155	131,532,695	1	0														
1860	8	131,532,696	133,264,660	2	1	1_1_1_1_1_0_0_0_0_1_1_1_0.948	2	2	2	2	2	2	2	2	1	1	1	1	1
1861	9	6,170	756,598	2	1	1_1_1_1_1_0_1_0_0_1_1_1_0.141	2	2	2	2	2	2	2	2	2	1	1	1	1
1862	9	756,599	2,048,672	1	0														
1863	9	2,048,673	2,173,663	2	1	0_0_0_0_0_0_0_0_0_0_1_0_0.101	1	1	1	1	1	1	1	2	1	1	1	1	1

Disease Models & Mechanisms • Supplementary information

1864	9	2,173,664	2,685,276	1	0														
1865	9	2,685,277	3,095,279	2	1	0_0_0_0_0_0_0_0_0_0_1_0_0.564	1	1	1	1	1	1	1	2	1	1	1	1	1
1866	9	3,095,280	3,101,158	1	0														
1867	9	3,101,159	3,166,971	2	1	1_1_1_1_1_0_1_0_0_1_1_1_0.175	2	2	2	2	2	2	2	2	2	1	1	1	1
1868	9	3,166,972	3,275,043	3	2	0_1_0_0_0_0_0_0_0_1_1_1_0.23	3	3	3	2	2	2	2	3	2	1	1	1	1
1869	9	3,275,044	3,278,568	2	1	0_1_0_0_0_0_0_0_0_1_1_1_0.23	2	2	2	1	1	1	1	2	1	1	1	1	1
1870	9	3,278,569	3,279,038	1	0														
1871	9	3,279,039	3,873,439	2	1	0_0_0_0_0_0_0_0_0_0_1_0_0.498	1	1	1	1	1	1	1	2	1	1	1	1	1
1872	9	3,873,440	3,991,143	1	0														
1873	9	3,991,144	4,560,315	2	1	1_1_1_1_1_0_1_0_0_1_1_1_0.173	2	2	2	2	2	2	2	2	2	1	1	1	1
1874	9	4,560,316	7,185,432	1	0														
1875	9	7,185,433	7,227,430	2	1	1_1_1_1_1_0_0_0_0_1_1_1_0.26	2	2	2	2	2	2	2	2	1	1	1	1	1
1876	9	7,227,431	10,098,654	1	0														
1877	9	10,098,655	11,403,300	2	1	1_1_1_1_1_0_1_0_0_1_0_1_0.343	2	2	2	2	2	2	2	1	2	1	1	1	1
1878	9	11,403,301	13,388,768	1	0														
1879	9	13,388,769	14,894,472	2	1	1_1_1_1_1_0_1_0_0_1_1_1_0.208	2	2	2	2	2	2	2	2	2	1	1	1	1
1880	9	14,894,473	15,091,783	1	0														
1881	9	15,091,784	15,478,096	2	1	1_1_1_1_1_1_1_0_1_1_0_1_0.384	2	2	2	2	2	2	2	1	2	1	2	1	2
1882	9	15,478,097	15,985,758	1	0														
1883	9	15,985,759	17,523,948	2	1	1_1_1_1_1_1_1_0_1_1_0_1_0.814	2	2	2	2	2	2	2	1	2	1	2	1	2
1884	9	17,523,949	17,525,732	1	0														
1885	9	17,525,733	18,145,596	2	1	0_1_0_0_0_0_0_0_0_0_1_0_1.136	2	1	1	1	1	1	1	2	1	2	1	1	1
1886	9	18,145,597	18,221,172	3	2	0_1_0_0_0_0_0_0_0_0_1_0_1.136	3	2	2	2	2	2	2	3	2	3	1	1	1
1887	9	18,221,173	18,312,208	3	3	0_1_0_0_0_0_0_0_0_0_1_0_1.136	3	2	2	2	2	2	2	3	2	3	1	1	1
1888	9	18,312,209	18,419,216	3	2	0_1_0_0_0_0_0_0_0_0_1_0_1.136	3	2	2	2	2	2	2	3	2	3	1	1	1
1889	9	18,419,217	18,708,946	3	3	0_1_0_0_0_0_0_0_0_0_1_0_1.136	3	2	2	2	2	2	2	3	2	3	1	1	1
1890	9	18,708,947	18,733,406	3	2	1_0_1_1_1_0_1_0_0_1_0_1_0.206	2	3	3	3	3	3	3	2	3	2	1	1	1
1891	9	18,733,407	18,733,426	4	3	1_0_1_1_1_0_1_0_0_1_0_1_0.206	3	4	4	4	4	4	4	2	4	2	1	1	1
1892	9	18,733,427	18,812,713	4	4	0_0_0_0_0_0_0_0_0_0_1_0_1.93	2	3	3	3	3	3	3	4	3	4	1	1	1
1893	9	18,812,714	18,993,611	3	3	0_0_0_0_0_0_0_0_0_0_1_0_1.93	2	2	2	2	2	2	2	3	2	3	1	1	1
1894	9	18,993,612	19,018,877	3	2	1_1_1_1_1_0_1_0_0_1_0_1_0.65	3	3	3	3	3	3	3	2	3	2	1	1	1
1895	9	19,018,878	19,021,050	2	1	1_1_1_1_1_0_1_0_0_1_1_1_1.88	2	2	2	2	2	2	2	2	2	2	1	1	1
1896	9	19,021,051	19,021,972	1	0														
1897	9	19,021,973	19,070,756	2	1	1_0_1_1_1_0_1_0_0_1_1_1_1.20	1	2	2	2	2	2	2	2	2	2	1	1	1
1898	9	19,070,757	19,075,871	1	0														
1899	9	19,075,872	19,079,518	2	1	0_1_0_0_0_0_0_0_0_0_1_0_1.87	2	1	1	1	1	1	1	2	1	2	1	1	1
1900	9	19,079,519	19,149,715	3	2	0_1_0_0_0_0_0_0_0_0_1_0_1.87	3	2	2	2	2	2	2	3	2	3	1	1	1
1901	9	19,149,716	19,152,546	2	1	1_0_1_1_1_0_1_0_0_1_0_1_0.28	1	2	2	2	2	2	2	1	2	1	1	1	1
1902	9	19,152,547	19,152,866	1	0														
1903	9	19,152,867	19,158,961	2	1	1_0_1_1_1_0_1_0_0_1_1_1_0.112	1	2	2	2	2	2	2	2	2	1	1	1	1
1904	9	19,158,962	19,330,090	3	2	1_0_1_1_1_0_1_0_0_1_1_1_0.112	1	3	3	3	3	3	3	3	3	1	2	2	2
1905	9	19,330,091	19,428,608	3	3	1_0_1_1_1_0_1_0_0_1_1_1_0.112	1	3	3	3	3	3	3	3	3	1	2	2	2
1906	9	19,428,609	19,428,757	3	2	1_0_1_1_1_1_1_1_1_1_1_1_0.134	1	3	3	3	3	3	3	3	3	1	2	2	2
1907	9	19,428,758	20,002,015	2	1	1_1_1_1_1_0_1_0_0_1_1_1_1.329	2	2	2	2	2	2	2	2	2	2	1	1	1
1908	9	20,002,016	20,025,354	1	0														
1909	9	20,025,355	20,459,839	2	1	1_1_1_1_1_0_1_0_0_1_1_1_1.238	2	2	2	2	2	2	2	2	2	2	1	1	1
1910	9	20,459,840	20,513,602	1	0														
1911	9	20,513,603	20,728,273	2	1	0_0_0_0_0_0_0_0_0_0_0_0_1.260	1	1	1	1	1	1	1	1	1	2	1	1	1
1912	9	20,728,274	20,729,085	1	0														
1913	9	20,729,086	20,962,949	2	1	1_1_1_1_1_0_1_0_0_1_1_1_0.157	2	2	2	2	2	2	2	2	2	1	1	1	1
1914	9	20,962,950	20,980,559	1	0														
1915	9	20,980,560	21,431,771	2	1	0_1_0_0_0_1_0_1_1_1_1_1_1.413	2	2	2	1	1	1	1	2	1	2	2	2	2
1916	9	21,431,772	22,125,954	1	0														
1917	9	22,125,955	22,593,791	2	1	0_1_0_0_0_1_0_1_1_1_1_1_1.143	2	2	2	1	1	1	1	2	1	2	2	2	2
1918	9	22,593,792	22,618,067	1	0														
1919	9	22,618,068	22,849,661	2	1	0_1_0_0_0_1_0_1_1_1_1_1_1.117	2	2	2	1	1	1	1	2	1	2	2	2	2
1920	9	22,849,662	22,854,220	1	0														
1921	9	22,854,221	22,868,546	2	1	1_1_1_1_1_0_1_0_0_1_1_1_1.42	2	2	2	2	2	2	2	2	2	2	1	1	1
1922	9	22,868,547	22,874,260	3	2	1_1_1_1_1_0_1_0_0_1_1_1_0.24	3	3	3	3	3	3	3	3	3	2	1	1	1
1923	9	22,874,261	22,897,756	4	3	0_1_0_0_0_0_0_0_0_1_1_1_1.24	4	4	4	2	2	2	2	4	2	3	1	1	1
1924	9	22,897,757	22,918,584	3	2	1_1_1_1_1_0_1_0_0_1_1_1_0.24	3	3	3	3	3	3	3	3	3	2	1	1	1
1925	9	22,918,585	22,925,984	3	3	0_0_0_0_0_0_0_0_0_0_0_0_1.91	2	2	2	2	2	2	2	2	2	3	1	1	1
1926	9	22,925,985	22,930,187	3	2	0_0_0_0_0_0_0_0_0_0_0_0_1.91	2	2	2	2	2	2	2	2	2	3	1	1	1
1927	9	22,930,188	22,948,271	2	1	0_0_0_0_0_0_0_0_0_0_0_0_1.91	1	1	1	1	1	1	1	1	1	2	1	1	1
1928	9	22,948,272	23,128,064	3	2	0_0_0_0_0_0_0_0_0_0_0_0_1.91	1	1	1	2	2	2	2	1	2	3	1	1	1
1929	9	23,128,065	23,158,421	2	1	1_0_1_1_1_0_1_0_0_0_0_0_1.171	1	1	1	2	2	2	2	1	2	2	1	1	1
1930	9	23,158,422	23,493,802	3	2	0_0_0_0_0_0_0_0_0_0_0_0_1.305	1	1	1	2	2	2	2	1	2	3	1	1	1
1931	9	23,493,803	23,504,268	2	1	0_0_0_0_0_0_0_0_0_0_0_0_1.305	1	1	1	1	1	1	1	1	1	2	1	1	1
1932	9	23,504,269	23,507,955	3	2	0_0_0_0_0_0_0_0_0_0_0_0_1.305	1	1	1	2	2	2	2	2	2	3	1	1	1
1933	9	23,507,956	24,237,970	3	3	0_0_0_0_0_0_0_0_0_0_0_0_1.305	2	2	2	1	1	1	1	1	1	3	2	2	2
1934	9	24,237,971	24,308,817	3	2	0_1_0_0_0_1_0_1_1_1_0_1_0.440	3	3	3	2	2	2	2	2	2	1	3	3	3
1935	9	24,308,818	24,739,293	3	3	0_0_0_0_0_0_0_0_0_0_0_0_1.201	2	2	2	1	1	1	1	1	1	3	2	2	2
1936	9	24,739,294	24,749,279	3	2	0_1_0_0_0_1_0_1_1_1_0_1_0.440	3	3	3	2	2	2	2	2	2	1	3	3	3
1937	9	24,749,280	24,768,840	2	1	0_1_0_0_0_1_0_1_1_1_0_1_0.440	2	2	2	1	1	1	1	1	1	1	2	2	2
1938	9	24,768,841	24,769,992	1	0														
1939	9	24,769,993	25,803,234	2	1	0_1_0_0_0_0_0_0_0_1_0_1_0.176	2	2	2	1	1	1	1	1	1	1	1	1	1
1940	9	25,803,235	25,989,941	3	2	0_1_0_0_0_0_0_0_0_1_0_1_0.176	3	3	3	2	2	2	2	1	2	1	1	1	1
1941	9	25,989,942	26,009,471	3	3	0_1_0_0_0_0_0_0_0_1_0_1_0.176	3	3	3	1	1	1	1	2	1	2	2	2	2
1942	9	26,009,472	26,054,874	3	2	0_1_0_0_0_1_0_1_1_1_1_1_1.35	3	3	3	1	1	1	1	2	1	2	2	2	2
1943	9	26,054,875	26,630,511	2	1	1_1_1_1_1_0_1_0_0_1_0_1_0.630	2	2	2	2	2	2	2	1	2	1	1	1	1
1944	9	26,630,512	27,224,274	1	0														
1945	9	27,224,275	28,086,983	2	1	0_0_0_0_0_0_0_0_0_0_0_0_1.763	1	1	1	1	1	1	1	1	1	2	1	1	1
1946	9	28,086,984	28,127,209	1	0														
1947	9	28,127,210	28,144,984	2	1	0_0_0_0_0_0_0_0_0_0_1_0_0.35	1	1	1	1	1	1	1	2	1	1	1	1	1
1948	9	28,144,985	28,158,032	1	0														

Disease Models & Mechanisms • Supplementary information

1949	9	28,158,033	28,246,425	2	1	1_1_1_1_1_0_1_0_0_1_0_1_0.160	2	2	2	2	2	2	2	1	2	1	1	1	1
1950	9	28,246,426	28,258,522	3	2	0_0_0_0_0_0_0_0_0_0_1_0_0.131	2	2	2	2	2	2	2	3	2	1	1	1	1
1951	9	28,258,523	28,345,465	2	1	0_0_0_0_0_0_0_0_0_0_1_0_0.131	1	1	1	1	1	1	1	2	1	1	1	1	1
1952	9	28,345,466	28,349,729	3	2	0_0_0_0_0_0_0_0_0_0_1_0_0.131	2	2	2	2	2	2	2	3	2	1	1	1	1
1953	9	28,349,730	28,392,994	3	3	0_0_0_0_0_0_0_0_0_0_1_0_0.131	2	2	2	2	2	2	2	3	2	1	1	1	1
1954	9	28,392,995	28,480,693	3	2	1_1_1_1_1_0_1_0_0_1_0_1_0.157	3	3	3	3	3	3	3	2	3	1	1	1	1
1955	9	28,480,694	28,532,413	3	3	0_0_0_0_0_0_0_0_0_0_1_0_0.221	2	2	2	2	2	2	2	3	2	1	1	1	1
1956	9	28,532,414	28,567,562	3	2	1_1_1_1_1_0_1_0_0_1_0_1_0.157	3	3	3	3	3	3	3	2	3	1	1	1	1
1957	9	28,567,563	28,789,161	2	1	1_1_1_1_1_0_1_0_0_1_0_1_0.157	2	2	2	2	2	2	2	1	2	1	1	1	1
1958	9	28,789,162	28,792,050	1	0														
1959	9	28,792,051	29,066,548	2	1	1_1_1_1_1_0_1_0_0_1_1_1_0.282	2	2	2	2	2	2	2	2	2	1	1	1	1
1960	9	29,066,549	29,068,108	1	0														
1961	9	29,068,109	32,315,178	2	1	1_1_1_1_1_0_1_0_0_1_0_1_0.471	2	2	2	2	2	2	2	1	2	1	1	1	1
1962	9	32,315,179	32,334,705	1	0														
1963	9	32,334,706	34,298,856	2	1	1_1_1_1_1_0_1_0_0_1_0_1_0.198	2	2	2	2	2	2	2	1	2	1	1	1	1
1964	9	34,298,857	34,314,461	1	0														
1965	9	34,314,462	34,533,662	2	1	1_1_1_1_1_0_1_0_0_1_1_1_0.364	2	2	2	2	2	2	2	2	2	1	1	1	1
1966	9	34,533,663	34,534,592	3	2	0_0_0_0_0_0_0_0_0_0_1_0_0.458	2	2	2	2	2	2	2	3	2	1	1	1	1
1967	9	34,534,593	34,602,580	3	3	0_0_0_0_0_0_0_0_0_0_1_0_0.458	2	2	2	2	2	2	2	3	2	1	1	1	1
1968	9	34,602,581	34,603,614	3	2	0_0_0_0_0_0_0_0_0_0_1_0_0.458	2	2	2	2	2	2	2	3	2	1	1	1	1
1969	9	34,603,615	34,872,423	2	1	0_0_0_0_0_0_0_0_0_0_1_0_0.458	1	1	1	1	1	1	1	2	1	1	1	1	1
1970	9	34,872,424	35,345,485	1	0														
1971	9	35,345,486	35,878,962	2	1	1_1_1_1_1_0_1_0_0_1_0_1_0.417	2	2	2	2	2	2	2	1	2	1	1	1	1
1972	9	35,878,963	35,954,353	1	0														
1973	9	35,954,354	36,646,671	2	1	1_1_1_1_1_0_1_0_0_1_1_1_0.422	2	2	2	2	2	2	2	2	2	1	1	1	1
1974	9	36,646,672	36,690,608	1	0														
1975	9	36,690,609	40,838,817	2	1	0_0_0_0_0_0_0_0_0_0_1_0_0.276	1	1	1	1	1	1	1	2	1	1	1	1	1
1976	9	40,838,818	41,837,870	1	0														
1977	9	41,837,871	42,255,159	2	1	0_0_0_0_0_0_0_0_0_0_1_0_1.184	1	1	1	1	1	1	1	2	1	2	1	1	1
1978	9	42,255,160	42,280,947	3	2	0_0_0_0_0_0_0_0_0_0_1_0_1.184	2	2	2	2	2	2	2	3	2	3	1	1	1
1979	9	42,280,948	43,611,454	2	1	0_0_0_0_0_0_0_0_0_0_1_0_1.184	1	1	1	1	1	1	1	2	1	2	1	1	1
1980	9	43,611,455	44,252,322	1	0														
1981	9	44,252,323	45,522,115	2	1	0_1_0_0_0_1_0_1_1_1_1_1_1.590	2	2	2	1	1	1	1	2	1	2	2	2	2
1982	9	45,522,116	45,605,118	1	0														
1983	9	45,605,119	48,067,530	2	1	0_1_0_0_0_1_0_1_1_1_1_1_1.133	2	2	2	1	1	1	1	2	1	2	2	2	2
1984	9	48,067,531	48,072,451	1	0														
1985	9	48,072,452	48,078,189	2	1	0_1_0_0_0_0_0_0_0_1_1_1_0.570	2	2	2	1	1	1	1	2	1	1	1	1	1
1986	9	48,078,190	49,369,003	3	2	0_1_0_0_0_0_0_0_0_1_1_1_0.570	3	3	3	2	2	2	2	3	2	1	1	1	1
1987	9	49,369,004	49,433,445	2	1	1_1_1_1_1_0_1_0_0_1_1_1_0.642	2	2	2	2	2	2	2	2	2	1	1	1	1
1988	9	49,433,446	49,544,610	3	2	0_1_0_0_0_1_0_1_1_1_1_1_1.343	3	3	3	1	1	1	1	3	1	2	2	2	2
1989	9	49,544,611	50,875,503	2	1	0_1_0_0_0_1_0_1_1_1_1_1_1.343	2	2	2	1	1	1	1	2	1	2	2	2	2
1990	9	50,875,504	50,884,572	3	2	0_1_0_0_0_0_0_0_0_1_1_1_0.131	3	3	3	1	1	1	1	3	1	2	2	2	2
1991	9	50,884,573	51,155,060	3	3	0_1_0_0_0_0_0_0_0_1_1_1_0.131	3	3	3	1	1	1	1	3	1	2	2	2	2
1992	9	51,155,061	51,180,238	3	2	0_1_0_0_0_0_0_0_0_1_1_1_0.131	3	3	3	1	1	1	1	3	1	2	2	2	2
1993	9	51,180,239	55,255,502	2	1	0_1_0_0_0_1_0_1_1_1_1_1_1.343	2	2	2	1	1	1	1	2	1	2	2	2	2
1994	9	55,255,503	57,881,321	1	0														
1995	9	57,881,322	60,557,241	2	1	0_1_0_0_0_1_0_1_1_1_1_1_1.149	2	2	2	1	1	1	1	2	1	2	2	2	2
1996	9	60,557,242	60,560,522	1	0														
1997	9	60,560,523	62,998,469	2	1	0_1_0_0_0_0_0_0_0_1_1_1_0.237	2	2	2	1	1	1	1	2	1	1	1	1	1
1998	9	62,998,470	63,008,890	1	0														
1999	9	63,008,891	68,259,066	2	1	0_1_0_0_0_1_0_1_1_1_1_1_1.189	2	2	2	1	1	1	1	2	1	2	2	2	2
2000	9	68,259,067	68,285,647	1	0														
2001	9	68,285,648	68,287,470	2	1	0_0_0_0_0_0_0_0_0_1_1_1_0.122	1	2	2	1	1	1	1	2	1	1	1	1	1
2002	9	68,287,471	68,565,344	3	2	0_0_0_0_0_0_0_0_0_1_1_1_0.122	2	3	3	2	2	2	2	3	2	1	1	1	1
2003	9	68,565,345	69,003,039	3	3	0_0_0_0_0_0_0_0_0_1_1_1_0.122	1	3	3	1	1	1	1	3	1	2	2	2	2
2004	9	69,003,040	69,059,663	3	2	0_0_0_0_0_0_0_0_0_1_1_1_0.122	2	3	3	2	2	2	2	3	2	1	1	1	1
2005	9	69,059,664	69,334,854	3	3	0_0_0_0_0_0_0_0_0_1_1_1_0.122	1	3	3	1	1	1	1	3	1	2	2	2	2
2006	9	69,334,855	69,345,518	3	2	0_0_0_0_0_0_0_0_0_1_1_1_0.122	2	3	3	2	2	2	2	3	2	1	1	1	1
2007	9	69,345,519	69,508,507	2	1	0_0_0_0_0_0_0_0_0_1_1_1_0.122	1	2	2	1	1	1	1	2	1	1	1	1	1
2008	9	69,508,508	70,614,078	3	2	0_0_0_0_0_0_0_0_0_1_1_1_0.122	2	3	3	2	2	2	2	3	2	1	1	1	1
2009	9	70,614,079	70,614,308	2	1	1_1_1_1_1_0_1_0_0_1_1_1_0.507	2	2	2	2	2	2	2	2	2	1	1	1	1
2010	9	70,614,309	70,649,729	3	2	0_0_0_0_0_1_0_1_1_1_1_1_1.111	1	3	3	1	1	1	1	3	1	2	2	2	2
2011	9	70,649,730	70,788,172	2	1	0_0_0_0_0_1_0_1_1_1_1_1_1.111	1	2	2	1	1	1	1	2	1	2	2	2	2
2012	9	70,788,173	70,802,286	3	2	0_0_0_0_0_0_0_0_0_1_1_1_0.318	1	3	3	1	1	1	1	3	1	2	2	2	2
2013	9	70,802,287	71,024,244	3	3	0_0_0_0_0_0_0_0_0_1_1_1_0.318	1	3	3	1	1	1	1	3	1	2	2	2	2
2014	9	71,024,245	71,122,987	3	2	0_0_0_0_0_0_0_0_0_1_1_1_0.318	2	3	3	2	2	2	2	3	2	1	1	1	1
2015	9	71,122,988	71,228,314	3	3	0_0_0_0_0_0_0_0_0_1_1_1_0.318	1	3	3	1	1	1	1	3	1	2	2	2	2
2016	9	71,228,315	71,659,958	3	2	0_0_0_0_0_0_0_0_0_1_1_1_0.318	2	3	3	2	2	2	2	3	2	1	1	1	1
2017	9	71,659,959	72,181,387	3	3	0_0_0_0_0_0_0_0_0_1_1_1_0.318	1	3	3	1	1	1	1	3	1	2	2	2	2
2018	9	72,181,388	72,394,507	3	2	0_0_0_0_0_0_0_0_0_1_1_1_0.318	2	3	3	2	2	2	2	3	2	1	1	1	1
2019	9	72,394,508	72,940,483	3	3	0_0_0_0_0_0_0_0_0_1_1_1_0.318	1	3	3	1	1	1	1	3	1	2	2	2	2
2020	9	72,940,484	74,185,072	3	2	0_0_0_0_0_0_0_0_0_1_1_1_0.318	2	3	3	2	2	2	2	3	2	1	1	1	1
2021	9	74,185,073	75,753,861	2	1	0_0_0_0_0_0_0_0_0_1_1_1_0.318	1	2	2	1	1	1	1	2	1	1	1	1	1
2022	9	75,753,862	76,021,207	1	0														
2023	9	76,021,208	77,825,090	2	1	0_0_0_0_0_0_0_0_0_1_1_1_0.204	1	2	2	1	1	1	1	2	1	1	1	1	1
2024	9	77,825,091	77,836,607	3	2	0_0_0_0_0_0_0_0_0_1_1_1_0.204	2	3	3	2	2	2	2	3	2	1	1	1	1
2025	9	77,836,608	77,895,028	3	3	0_0_0_0_0_0_0_0_0_1_1_1_0.204	1	3	3	1	1	1	1	3	1	2	2	2	2
2026	9	77,895,029	78,196,861	3	2	0_0_0_0_0_0_0_0_0_1_1_1_0.204	2	3	3	2	2	2	2	3	2	1	1	1	1
2027	9	78,196,862	78,210,003	2	1	0_0_0_0_0_0_0_0_0_1_1_1_0.204	1	2	2	1	1	1	1	2	1	1	1	1	1
2028	9	78,210,004	78,218,152	3	2	0_0_0_0_0_0_0_0_0_1_1_1_0.204	1	3	3	1	1	1	1	3	1	2	2	2	2
2029	9	78,218,153	78,227,152	2	1	0_0_0_0_0_1_0_1_1_1_1_1_1.38	1	2	2	1	1	1	1	2	1	2	2	2	2
2030	9	78,227,153	78,260,309	3	2	0_0_0_0_0_0_0_0_0_1_1_1_1.65 [illegible]	1	3	3	1	1	1	1	3	1	3	2	2	2
2031	9	78,260,310	78,389,991	3	3	0_0_0_0_0_0_0_0_0_1_1_1_1.65 [illegible]	1	3	3	1	1	1	1	3	1	3	2	2	2
2032	9	78,389,992	78,435,141	3	2	0_0_0_0_0_0_0_0_0_1_1_1_1.65 [illegible]	2	3	3	2	2	2	2	3	2	3	1	1	1
2033	9	78,435,142	78,453,178	2	1	1_1_1_1_1_0_1_0_0_1_1_1_1.294	2	2	2	2	2	2	2	2	2	2	1	1	1

2034	9	78,453,179	78,491,753	3	2	0_0_0_0_0_0_0_0_0_1_1_1_0.753	2	3	3	2	2	2	2	3	2	2	1	1	1
2035	9	78,491,754	79,008,868	3	3	0_0_0_0_0_0_0_0_0_1_1_1_0.753	2	3	3	2	2	2	2	3	2	2	1	1	1
2036	9	79,008,869	79,155,074	3	2	0_0_0_0_0_0_0_0_0_1_1_1_0.753	2	3	3	2	2	2	2	3	2	2	1	1	1
2037	9	79,155,075	79,159,310	2	1	0_0_0_0_0_0_0_0_0_1_1_1_0.753	1	2	2	1	1	1	1	2	1	1	1	1	1
2038	9	79,159,311	79,260,385	3	2	0_0_0_0_0_0_0_0_0_1_1_1_0.753	2	3	3	2	2	2	2	3	2	1	1	1	1
2039	9	79,260,386	79,432,989	3	3	0_0_0_0_0_0_0_0_0_1_1_1_0.753	1	3	3	1	1	1	1	3	1	2	2	2	2
2040	9	79,432,990	79,469,298	3	2	0_0_0_0_0_0_0_0_0_1_1_1_0.753	2	3	3	2	2	2	2	3	2	1	1	1	1
2041	9	79,469,299	79,492,163	3	3	0_0_0_0_0_0_0_0_0_1_1_1_0.753	1	3	3	1	1	1	1	3	1	2	2	2	2
2042	9	79,492,164	79,564,552	3	2	0_0_0_0_0_0_0_0_0_1_1_1_0.753	2	3	3	2	2	2	2	3	2	1	1	1	1
2043	9	79,564,553	79,612,097	4	3	0_0_0_0_0_0_0_0_0_1_1_1_0.753	3	4	4	3	3	3	3	4	3	2	1	1	1
2044	9	79,612,098	79,658,237	4	4	0_0_0_0_0_0_0_0_0_0_0_0_1.158	2	3	3	2	2	2	2	3	2	4	1	1	1
2045	9	79,658,238	79,784,982	4	3	0_0_0_0_0_0_0_0_0_0_0_0_1.158	2	3	3	2	2	2	2	3	2	4	1	1	1
2046	9	79,784,983	79,791,174	3	2	0_0_0_0_0_0_0_0_0_0_0_0_1.158	2	1	1	2	2	2	2	1	2	3	1	1	1
2047	9	79,791,175	79,798,216	2	1	0_0_0_0_0_0_0_0_0_0_0_0_1.158	1	1	1	1	1	1	1	1	1	2	1	1	1
2048	9	79,798,217	79,799,660	1	0														
2049	9	79,799,661	79,821,978	2	1	0_0_0_0_0_0_0_0_0_1_1_1_1.127	1	2	2	1	1	1	1	2	1	2	1	1	1
2050	9	79,821,979	79,966,169	3	2	0_0_0_0_0_0_0_0_0_1_1_1_1.127	1	3	3	1	1	1	1	3	1	3	2	2	2
2051	9	79,966,170	80,111,679	3	3	0_0_0_0_0_0_0_0_0_1_1_1_1.127	1	3	3	1	1	1	1	3	1	3	2	2	2
2052	9	80,111,680	80,113,928	3	2	0_0_0_0_0_0_0_0_0_1_1_1_1.127	1	3	3	1	1	1	1	3	1	3	2	2	2
2053	9	80,113,929	80,122,027	2	1	0_0_0_0_0_0_0_0_0_1_1_1_1.127	1	2	2	1	1	1	1	2	1	2	1	1	1
2054	9	80,122,028	88,736,282	1	0														
2055	9	88,736,283	93,275,332	2	1	1_1_1_1_1_0_1_0_0_1_1_1_0.318	2	2	2	2	2	2	2	2	2	1	1	1	1
2056	9	93,275,333	93,276,740	3	2	1_1_1_1_1_0_1_0_0_1_0_1_0.60	3	3	3	3	3	3	3	2	3	1	1	1	1
2057	9	93,276,741	93,402,484	3	3	0_0_0_0_0_0_0_0_0_0_1_0_0.21	2	2	2	2	2	2	2	3	2	1	1	1	1
2058	9	93,402,485	93,403,154	3	2	0_0_0_0_0_0_0_0_0_0_1_0_0.21	2	2	2	2	2	2	2	3	2	1	1	1	1
2059	9	93,403,155	93,406,281	2	1	1_1_1_1_1_0_1_0_0_1_1_1_0.318	2	2	2	2	2	2	2	2	2	1	1	1	1
2060	9	93,406,282	93,542,437	3	2	0_1_0_0_0_0_0_0_0_1_1_1_0.101	3	3	3	2	2	2	2	3	2	1	1	1	1
2061	9	93,542,438	93,542,504	2	1	1_1_1_1_1_0_1_0_0_1_1_1_0.318	2	2	2	2	2	2	2	2	2	1	1	1	1
2062	9	93,542,505	93,549,739	3	2	1_0_1_1_1_0_1_0_0_0_1_0_0.263	2	2	2	3	3	3	3	3	3	1	1	1	1
2063	9	93,549,740	94,300,665	3	3	0_1_0_0_0_0_0_0_0_1_0_1_0.408	3	3	3	2	2	2	2	2	2	1	1	1	1
2064	9	94,300,666	94,382,454	3	2	0_1_0_0_0_0_0_0_0_1_0_1_0.408	3	3	3	2	2	2	2	2	2	1	1	1	1
2065	9	94,382,455	94,384,751	2	1	1_1_1_1_1_0_1_0_0_1_1_1_0.318	2	2	2	2	2	2	2	2	2	1	1	1	1
2066	9	94,384,752	95,435,060	3	2	0_1_0_0_0_0_0_0_0_1_1_1_0.118	3	3	3	2	2	2	2	3	2	1	1	1	1
2067	9	95,435,061	96,214,953	2	1	0_1_0_0_0_0_0_0_0_1_1_1_0.118	2	2	2	1	1	1	1	2	1	1	1	1	1
2068	9	96,214,954	96,597,297	1	0														
2069	9	96,597,298	99,738,788	2	1	1_1_1_1_1_0_1_0_0_1_1_1_0.203	2	2	2	2	2	2	2	2	2	1	1	1	1
2070	9	99,738,789	101,142,588	1	0														
2071	9	101,142,589	101,239,381	2	1	0_0_0_0_0_1_1_1_1_1_0_1_1.147	1	2	2	1	1	1	1	1	2	2	2	2	2
2072	9	101,239,382	101,250,240	1	0														
2073	9	101,250,241	101,595,318	2	1	1_1_1_1_1_0_1_0_0_1_1_1_0.175	2	2	2	2	2	2	2	2	2	1	1	1	1
2074	9	101,595,319	105,711,537	1	0														
2075	9	105,711,538	105,891,026	2	1	0_0_0_0_0_0_0_1_0_0_0_0_1.275	1	1	1	1	1	1	1	1	1	2	1	2	1
2076	9	105,891,027	106,773,276	1	0														
2077	9	106,773,277	107,042,353	2	1	0_0_0_0_0_0_0_1_0_0_0_0_1.236	1	1	1	1	1	1	1	1	1	2	1	2	1
2078	9	107,042,354	107,051,055	1	0														
2079	9	107,051,056	107,793,347	2	1	0_1_0_0_0_0_0_0_0_1_1_1_0.938	2	2	2	1	1	1	1	2	1	1	1	1	1
2080	9	107,793,348	109,422,592	1	0														
2081	9	109,422,593	111,007,431	2	1	0_1_0_0_0_0_0_0_0_1_1_1_0.134	2	2	2	1	1	1	1	2	1	1	1	1	1
2082	9	111,007,432	111,374,746	1	0														
2083	9	111,374,747	112,798,205	2	1	0_1_0_0_0_0_0_0_0_1_1_1_0.150	2	2	2	1	1	1	1	2	1	1	1	1	1
2084	9	112,798,206	112,877,858	1	0														
2085	9	112,877,859	114,910,210	2	1	0_1_0_0_0_0_0_0_0_1_1_1_0.162	2	2	2	1	1	1	1	2	1	1	1	1	1
2086	9	114,910,211	116,667,583	1	0														
2087	9	116,667,584	117,930,050	2	1	0_1_0_0_0_0_0_0_0_1_1_1_0.128	2	2	2	1	1	1	1	2	1	1	1	1	1
2088	9	117,930,051	117,930,804	1	0														
2089	9	117,930,805	118,304,821	2	1	1_0_1_1_1_0_1_1_0_0_0_0_1.595	1	1	1	2	2	2	2	1	2	2	1	2	1
2090	9	118,304,822	118,798,164	1	0														
2091	9	118,798,165	119,354,733	2	1	1_1_1_1_1_1_1_0_1_1_1_1_1.267	2	2	2	2	2	2	2	2	2	2	2	1	2
2092	9	119,354,734	119,526,606	1	0														
2093	9	119,526,607	122,082,980	2	1	1_1_1_1_1_1_1_0_1_1_1_1_1.121	2	2	2	2	2	2	2	2	2	2	2	1	2
2094	10	143,014	5,287,831	2	1	1_1_1_1_1_0_0_0_0_1_1_1_0.365	2	2	2	2	2	2	2	2	1	1	1	1	1
2095	10	5,287,832	5,626,363	3	2	0_1_0_0_0_0_0_0_0_1_1_1_0.538	3	3	3	2	2	2	2	3	1	1	1	1	1
2096	10	5,626,364	5,857,499	3	3	0_1_0_0_0_0_0_0_0_1_1_1_0.538	3	3	3	1	1	1	1	3	2	2	2	2	2
2097	10	5,857,500	6,198,888	3	2	0_1_0_0_0_0_0_0_0_1_1_1_0.538	3	3	3	2	2	2	2	3	1	1	1	1	1
2098	10	6,198,889	6,295,817	2	1	1_1_1_1_1_0_0_0_0_1_1_1_0.365	2	2	2	2	2	2	2	2	1	1	1	1	1
2099	10	6,295,818	6,296,881	3	2	0_1_0_0_0_1_1_1_1_1_1_1_1.30	3	3	3	1	1	1	1	3	2	2	2	2	2
2100	10	6,296,882	6,472,816	3	3	0_1_0_0_0_0_0_0_0_1_1_1_0.500	3	3	3	1	1	1	1	3	2	2	2	2	2
2101	10	6,472,817	6,516,600	3	2	0_1_0_0_0_0_0_0_0_1_1_1_0.500	3	3	3	2	2	2	2	3	1	1	1	1	1
2102	10	6,516,601	6,558,099	3	3	0_1_0_0_0_0_0_0_0_1_1_1_0.500	3	3	3	1	1	1	1	3	2	2	2	2	2
2103	10	6,558,100	6,612,843	3	2	0_1_0_0_0_0_0_0_0_1_1_1_0.500	3	3	3	1	1	1	1	3	2	2	2	2	2
2104	10	6,612,844	6,626,313	2	1	0_1_0_0_0_0_0_0_0_1_1_1_0.500	2	2	2	1	1	1	1	2	1	1	1	1	1
2105	10	6,626,314	6,760,052	3	2	0_1_0_0_0_0_0_0_0_1_1_1_0.500	3	3	3	2	2	2	2	3	1	1	1	1	1
2106	10	6,760,053	6,895,514	3	3	0_1_0_0_0_0_0_0_0_1_1_1_0.500	3	3	3	1	1	1	1	3	2	2	2	2	2
2107	10	6,895,515	7,140,098	3	2	0_1_0_0_0_0_0_0_0_1_1_1_0.500	3	3	3	2	2	2	2	3	1	1	1	1	1
2108	10	7,140,099	7,234,972	2	1	1_1_1_1_1_0_0_0_0_1_1_1_0.547	2	2	2	2	2	2	2	2	1	1	1	1	1
2109	10	7,234,973	7,423,438	3	2	0_1_0_0_0_0_0_0_0_1_1_1_0.53	3	3	3	2	2	2	2	3	1	1	1	1	1
2110	10	7,423,439	7,424,857	2	1	1_1_1_1_1_0_0_0_0_1_1_1_0.547	2	2	2	2	2	2	2	2	1	1	1	1	1
2111	10	7,424,858	7,427,069	1	0														
2112	10	7,427,070	7,465,406	2	1	0_0_0_0_0_0_0_0_0_0_1_0_0.133	1	1	1	1	1	1	1	2	1	1	1	1	1
2113	10	7,465,407	7,537,136	3	2	0_0_0_0_0_0_0_0_0_0_1_0_0.133	1	1	1	2	2	2	2	3	1	1	1	1	1
2114	10	7,537,137	7,561,966	2	1	0_0_0_0_0_0_0_0_0_0_1_0_0.133	1	1	1	1	1	1	1	2	1	1	1	1	1
2115	10	7,561,967	7,613,506	1	0														
2116	10	7,613,507	7,666,279	2	1	1_1_1_1_1_0_0_0_0_1_1_1_0.69	2	2	2	2	2	2	2	2	1	1	1	1	1
2117	10	7,666,280	7,711,879	3	2	0_1_0_0_0_0_0_0_0_1_1_1_0.24	3	3	3	2	2	2	2	3	1	1	1	1	1
2118	10	7,711,880	7,714,278	2	1	0_1_0_0_0_0_0_0_0_1_1_1_0.24	2	2	2	1	1	1	1	2	1	1	1	1	1

2119	10	7,714,279	7,718,616	1	0														
2120	10	7,718,617	7,774,828	2	1	0_1_0_0_0_1_1_1_1_1_1_1_1.172	2	2	2	1	1	1	1	2	2	2	2	2	2
2121	10	7,774,829	7,777,895	3	2	0_1_0_0_0_1_1_1_1_1_1_1_1.172	2	2	2	1	1	1	1	3	2	2	2	2	2
2122	10	7,777,896	8,069,917	3	3	0_0_0_0_0_0_0_0_0_0_1_0_0.225	2	2	2	1	1	1	1	3	2	2	2	2	2
2123	10	8,069,918	8,073,086	3	2	0_0_0_0_0_0_0_0_0_0_1_0_0.225	2	2	2	1	1	1	1	3	2	2	2	2	2
2124	10	8,073,087	8,075,473	2	1	0_1_0_0_0_1_1_1_1_1_1_1_1.172	2	2	2	1	1	1	1	2	2	2	2	2	2
2125	10	8,075,474	8,076,911	3	2	0_1_0_0_0_0_0_0_0_1_0_1_0.305	3	3	3	1	1	1	1	2	2	2	2	2	2
2126	10	8,076,912	8,226,144	3	3	0_1_0_0_0_0_0_0_0_1_0_1_0.305	3	3	3	1	1	1	1	2	2	2	2	2	2
2127	10	8,226,145	8,227,759	3	2	0_1_0_0_0_0_0_0_0_1_0_1_0.305	3	3	3	1	1	1	1	2	2	2	2	2	2
2128	10	8,227,760	8,467,176	2	1	0_1_0_0_0_0_0_0_0_1_0_1_0.305	2	2	2	1	1	1	1	1	1	1	1	1	1
2129	10	8,467,177	9,061,017	1	0														
2130	10	9,061,018	9,359,271	2	1	0_1_0_0_0_0_0_0_0_1_0_1_0.276	2	2	2	1	1	1	1	1	1	1	1	1	1
2131	10	9,359,272	9,360,936	1	0														
2132	10	9,360,937	13,233,442	2	1	1_1_1_1_1_0_0_0_0_1_0_1_0.345	2	2	2	2	2	2	2	1	1	1	1	1	1
2133	10	13,233,443	13,271,704	3	2	1_1_1_1_1_0_0_0_0_1_0_1_0.345	3	3	3	3	3	3	3	2	2	1	1	1	1
2134	10	13,271,705	13,617,000	2	1	1_1_1_1_1_0_0_0_0_1_0_1_0.345	2	2	2	2	2	2	2	1	1	1	1	1	1
2135	10	13,617,001	14,892,034	1	0														
2136	10	14,892,035	18,986,116	2	1	1_1_1_1_1_0_0_0_0_1_0_1_0.497	2	2	2	2	2	2	2	1	1	1	1	1	1
2137	10	18,986,117	18,988,943	3	2	1_1_1_1_1_0_0_0_0_1_0_1_0.497	3	3	3	3	3	3	3	2	1	1	1	1	1
2138	10	18,988,944	19,022,796	3	3	0_0_0_0_0_0_0_0_0_0_1_0_0.154	2	2	2	2	2	2	2	3	1	1	1	1	1
2139	10	19,022,797	19,024,298	3	2	0_0_0_0_0_0_0_0_0_0_1_0_0.154	2	2	2	2	2	2	2	3	1	1	1	1	1
2140	10	19,024,299	19,073,724	2	1	0_0_0_0_0_0_0_0_0_0_1_0_0.154	1	1	1	1	1	1	1	2	1	1	1	1	1
2141	10	19,073,725	19,106,629	3	2	0_0_0_0_0_0_0_0_0_0_1_0_0.154	2	2	2	2	2	2	2	3	1	1	1	1	1
2142	10	19,106,630	19,107,991	3	3	0_0_0_0_0_0_0_0_0_0_1_0_0.154	2	2	2	2	2	2	2	3	1	1	1	1	1
2143	10	19,107,992	19,114,372	3	2	1_1_1_1_1_0_0_0_0_1_0_1_0.35	3	3	3	3	3	3	3	2	1	1	1	1	1
2144	10	19,114,373	19,127,270	2	1	1_1_1_1_1_0_0_0_0_1_1_1_0.167	2	2	2	2	2	2	2	2	1	1	1	1	1
2145	10	19,127,271	19,131,778	3	2	0_0_0_0_0_0_0_0_0_0_1_0_0.45	2	2	2	2	2	2	2	3	1	1	1	1	1
2146	10	19,131,779	19,165,555	3	3	0_0_0_0_0_0_0_0_0_0_1_0_0.45	2	2	2	2	2	2	2	3	1	1	1	1	1
2147	10	19,165,556	19,165,919	3	2	1_1_1_1_1_0_0_0_0_1_0_1_0.22	3	3	3	3	3	3	3	2	1	1	1	1	1
2148	10	19,165,920	19,350,141	2	1	1_1_1_1_1_0_0_0_0_1_1_1_0.167	2	2	2	2	2	2	2	2	1	1	1	1	1
2149	10	19,350,142	19,350,503	3	2	1_1_1_1_1_0_0_0_0_1_0_1_0.67	3	3	3	3	3	3	3	2	1	1	1	1	1
2150	10	19,350,504	19,455,857	3	3	0_0_0_0_0_0_0_0_0_0_1_0_0.65	2	2	2	2	2	2	2	3	1	1	1	1	1
2151	10	19,455,858	19,456,017	3	2	1_1_1_1_1_0_0_0_0_1_0_1_0.67	3	3	3	3	3	3	3	2	1	1	1	1	1
2152	10	19,456,018	20,343,341	2	1	1_1_1_1_1_0_0_0_0_1_1_1_0.167	2	2	2	2	2	2	2	2	1	1	1	1	1
2153	10	20,343,342	20,344,301	3	2	1_1_1_1_1_0_0_0_0_1_0_1_0.187	3	3	3	3	3	3	3	2	1	1	1	1	1
2154	10	20,344,302	20,429,414	3	3	0_0_0_0_0_0_0_0_0_0_1_0_0.80	2	2	2	2	2	2	2	3	1	1	1	1	1
2155	10	20,429,415	20,445,322	4	4	0_0_0_0_0_0_0_0_0_0_0_0_1.69	2	2	2	2	2	2	2	3	1	4	1	1	1
2156	10	20,445,323	20,494,331	4	3	0_0_0_0_0_0_0_0_0_0_0_0_1.69	2	2	2	2	2	2	2	3	1	4	1	1	1
2157	10	20,494,332	20,496,026	3	2	0_0_0_0_0_0_0_0_0_0_0_0_1.69	1	1	1	1	1	1	1	2	1	3	1	1	1
2158	10	20,496,027	20,499,295	2	1	0_0_0_0_0_0_0_0_0_0_0_0_1.69	1	1	1	1	1	1	1	1	1	2	1	1	1
2159	10	20,499,296	20,502,646	3	2	0_0_0_0_0_0_0_0_0_0_0_0_1.69	1	1	1	2	2	1	2	2	1	3	1	1	1
2160	10	20,502,647	20,642,315	3	3	0_0_0_0_0_0_0_0_0_0_0_0_1.69	2	2	2	1	1	2	1	1	2	3	2	2	2
2161	10	20,642,316	20,647,125	3	2	0_0_0_0_0_0_0_0_0_0_0_0_1.69	2	2	2	1	1	2	1	1	2	3	2	2	2
2162	10	20,647,126	21,184,857	2	1	0_1_0_1_0_1_1_1_1_1_0_1_0.777	2	2	2	1	1	2	1	1	2	1	2	2	2
2163	10	21,184,858	21,188,806	1	0														
2164	10	21,188,807	21,382,151	2	1	1_1_1_1_1_0_0_0_0_1_1_1_1.96	2	2	2	2	2	2	2	2	1	2	1	1	1
2165	10	21,382,152	21,390,541	1	0														
2166	10	21,390,542	23,751,086	2	1	1_1_1_1_1_0_0_0_0_1_1_1_0.267	2	2	2	2	2	2	2	2	1	1	1	1	1
2167	10	23,751,087	24,130,091	1	0														
2168	10	24,130,092	25,222,121	2	1	1_1_1_1_1_0_0_0_0_1_1_1_0.122	2	2	2	2	2	2	2	2	1	1	1	1	1
2169	10	25,222,122	27,424,781	1	0														
2170	10	27,424,782	27,746,186	2	1	1_1_1_1_1_0_0_0_0_1_1_1_0.264	2	2	2	2	2	2	2	2	1	1	1	1	1
2171	10	27,746,187	28,944,848	1	0														
2172	10	28,944,849	29,075,385	2	1	1_1_1_1_1_0_0_0_0_1_1_1_0.190	2	2	2	2	2	2	2	2	1	1	1	1	1
2173	10	29,075,386	30,239,763	1	0														
2174	10	30,239,764	30,728,796	2	1	1_1_1_1_1_0_0_0_0_1_1_1_0.819	2	2	2	2	2	2	2	2	1	1	1	1	1
2175	10	30,728,797	30,756,813	3	2	0_0_0_0_0_0_0_0_0_0_0_0_1.454	2	2	2	2	2	2	2	2	1	3	1	1	1
2176	10	30,756,814	30,851,889	3	3	0_0_0_0_0_0_0_0_0_0_0_0_1.454	2	2	2	2	2	2	2	2	1	3	1	1	1
2177	10	30,851,890	30,852,768	3	2	0_0_0_0_0_0_0_0_0_0_0_0_1.454	2	2	2	2	2	2	2	2	1	3	1	1	1
2178	10	30,852,769	30,853,439	2	1	0_0_0_0_0_0_0_0_0_0_0_0_1.454	1	1	1	1	1	1	1	1	1	2	1	1	1
2179	10	30,853,440	31,344,159	3	2	0_0_0_0_0_0_0_0_0_0_0_0_1.454	2	2	2	2	2	2	2	2	2	3	1	1	1
2180	10	31,344,160	32,641,857	2	1	1_1_1_1_1_0_1_0_0_1_1_1_0.230	2	2	2	2	2	2	2	2	2	1	1	1	1
2181	10	32,641,858	33,960,785	1	0														
2182	10	33,960,786	34,063,447	2	1	1_1_1_1_1_0_0_0_0_1_1_1_0.83	2	2	2	2	2	2	2	2	1	1	1	1	1
2183	10	34,063,448	36,217,661	1	0														
2184	10	36,217,662	38,258,778	2	1	1_1_1_1_1_0_1_0_0_1_1_1_1.101	2	2	2	2	2	2	2	2	2	2	1	1	1
2185	10	38,258,779	42,419,590	1	0														
2186	10	42,419,591	43,058,109	2	1	1_1_1_1_1_1_1_0_1_1_1_1_1.292	2	2	2	2	2	2	2	2	2	2	2	1	2
2187	10	43,058,110	43,119,174	1	0														
2188	10	43,119,175	43,344,085	2	1	1_1_1_1_1_1_1_0_1_1_1_1_1.87	2	2	2	2	2	2	2	2	2	2	2	1	2
2189	10	43,344,086	45,341,268	1	0														
2190	10	45,341,269	45,980,144	2	1	1_1_1_1_1_1_1_0_1_1_1_1_1.355	2	2	2	2	2	2	2	2	2	2	2	1	2
2191	10	45,980,145	45,981,246	1	0														
2192	10	45,981,247	50,570,884	2	1	1_1_1_1_1_0_1_0_0_1_1_1_1.243	2	2	2	2	2	2	2	2	2	2	1	1	1
2193	10	50,570,885	50,578,205	3	2	1_1_1_1_1_0_1_0_0_1_1_1_0.304	3	3	3	3	3	3	3	3	3	2	1	1	1
2194	10	50,578,206	50,723,796	3	3	0_0_0_0_0_0_0_0_0_0_0_0_1.55	2	2	2	2	2	2	2	2	2	3	1	1	1
2195	10	50,723,797	50,727,372	3	2	1_1_1_1_1_0_1_0_0_1_1_1_0.304	3	3	3	3	3	3	3	3	3	2	1	1	1
2196	10	50,727,373	50,808,844	2	1	1_1_1_1_1_0_1_0_0_1_1_1_0.304	2	2	2	2	2	2	2	2	2	1	1	1	1
2197	10	50,808,845	50,831,345	3	2	1_1_1_1_1_0_1_0_0_1_1_1_0.304	3	3	3	3	3	3	3	3	3	2	1	1	1
2198	10	50,831,346	51,323,680	2	1	1_1_1_1_1_0_1_0_0_1_1_1_1.711	2	2	2	2	2	2	2	2	2	2	1	1	1
2199	10	51,323,681	51,756,481	1	0														
2200	10	51,756,482	52,097,197	2	1	0_0_0_0_0_0_0_0_0_0_0_0_1.393	1	1	1	1	1	1	1	1	1	2	1	1	1
2201	10	52,097,198	52,101,045	1	0														
2202	10	52,101,046	52,101,901	2	1	1_1_1_1_1_0_1_0_0_1_1_1_1.242	2	2	2	2	2	2	2	2	2	2	1	1	1
2203	10	52,101,902	52,331,828	3	2	1_1_1_1_1_0_1_0_0_1_1_1_0.226	3	3	3	3	3	3	3	3	3	2	1	1	1

Disease Models & Mechanisms • Supplementary information

2204	10	52,331,829	52,357,717	3	3	0_0_0_0_0_0_0_0_0_0_0_0_1.336	2	2	2	2	2	2	2	2	2	3	1	1	1
2205	10	52,357,718	52,372,143	3	2	0_0_0_0_0_0_0_0_0_0_0_0_1.336	2	2	2	2	2	2	2	2	2	3	1	1	1
2206	10	52,372,144	52,585,720	3	3	0_0_0_0_0_0_0_0_0_0_0_0_1.336	2	2	2	2	2	2	2	2	2	3	1	1	1
2207	10	52,585,721	52,699,462	3	2	0_0_0_0_0_0_0_0_0_0_0_0_1.336	2	2	2	2	2	2	2	2	2	3	1	1	1
2208	10	52,699,463	52,703,136	2	1	1_1_1_1_1_0_1_0_0_1_1_1_0.226	2	2	2	2	2	2	2	2	2	1	1	1	1
2209	10	52,703,137	52,707,876	1	0														
2210	10	52,707,877	52,829,250	2	1	1_1_1_1_1_0_1_0_0_1_1_1_1.113	2	2	2	2	2	2	2	2	2	2	1	1	1
2211	10	52,829,251	53,921,112	1	0														
2212	10	53,921,113	55,026,591	2	1	1_1_1_1_1_0_1_0_0_1_1_1_1.579	2	2	2	2	2	2	2	2	2	2	1	1	1
2213	10	55,026,592	57,142,276	1	0														
2214	10	57,142,277	57,144,469	2	1	0_1_0_0_0_0_0_0_0_1_1_1_0.23	2	2	2	1	1	1	1	2	1	1	1	1	1
2215	10	57,144,470	63,467,004	1	0														
2216	10	63,467,005	63,612,792	2	1	1_1_1_1_1_0_1_0_0_1_1_1_0.125	2	2	2	2	2	2	2	2	2	1	1	1	1
2217	10	63,612,793	64,022,354	1	0														
2218	10	64,022,355	64,701,500	2	1	1_1_1_1_1_0_1_0_0_1_1_1_0.521	2	2	2	2	2	2	2	2	2	1	1	1	1
2219	10	64,701,501	69,637,613	1	0														
2220	10	69,637,614	70,303,225	2	1	1_1_1_1_1_0_1_0_0_1_1_1_0.419	2	2	2	2	2	2	2	2	2	1	1	1	1
2221	10	70,303,226	70,304,599	1	0														
2222	10	70,304,600	74,247,543	2	1	0_0_0_0_0_0_0_0_0_0_0_0_1.292	1	1	1	1	1	1	1	1	1	2	1	1	1
2223	10	74,247,544	76,428,533	1	0														
2224	10	76,428,534	76,540,378	2	1	0_0_0_0_0_0_0_0_0_0_0_0_1.24	1	1	1	1	1	1	1	1	1	2	1	1	1
2225	10	76,540,379	76,927,766	1	0														
2226	10	76,927,767	77,159,367	2	1	1_1_1_1_1_0_1_0_0_1_1_1_0.412	2	2	2	2	2	2	2	2	2	1	1	1	1
2227	10	77,159,368	77,175,759	3	2	0_0_0_0_0_0_0_0_0_0_0_0_1.227	2	2	2	2	2	2	2	2	2	3	1	1	1
2228	10	77,175,760	77,368,588	3	3	0_0_0_0_0_0_0_0_0_0_0_0_1.227	2	2	2	2	2	2	2	2	2	3	1	1	1
2229	10	77,368,589	77,517,022	3	2	0_0_0_0_0_0_0_0_0_0_0_0_1.227	2	2	2	2	2	2	2	2	2	3	1	1	1
2230	10	77,517,023	77,542,385	3	3	0_0_0_0_0_0_0_0_0_0_0_0_1.227	2	2	2	2	2	2	2	2	2	3	1	1	1
2231	10	77,542,386	77,546,176	3	2	0_0_0_0_0_0_0_0_0_0_0_0_1.227	2	2	2	2	2	2	2	2	2	3	1	1	1
2232	10	77,546,177	77,752,812	2	1	1_1_1_1_1_0_1_0_0_1_1_1_1.247	2	2	2	2	2	2	2	2	2	2	1	1	1
2233	10	77,752,813	77,758,582	1	0														
2234	10	77,758,583	77,761,022	2	1	1_1_1_1_1_0_1_0_0_1_1_1_0.486	2	2	2	2	2	2	2	2	2	1	1	1	1
2235	10	77,761,023	77,801,541	3	2	0_0_0_0_0_0_0_0_0_0_0_0_1.156	2	2	2	2	2	2	2	2	2	3	1	1	1
2236	10	77,801,542	77,925,701	3	3	0_0_0_0_0_0_0_0_0_0_0_0_1.156	2	2	2	2	2	2	2	2	2	3	1	1	1
2237	10	77,925,702	78,000,752	3	2	0_0_0_0_0_0_0_0_0_0_0_0_1.156	2	2	2	2	2	2	2	2	2	3	1	1	1
2238	10	78,000,753	78,336,319	2	1	1_1_1_1_1_0_1_0_0_1_1_1_0.486	2	2	2	2	2	2	2	2	2	1	1	1	1
2239	10	78,336,320	78,832,129	1	0														
2240	10	78,832,130	79,237,216	2	1	1_1_1_1_1_0_1_0_0_1_1_1_0.477	2	2	2	2	2	2	2	2	2	1	1	1	1
2241	10	79,237,217	79,930,283	1	0														
2242	10	79,930,284	80,170,797	2	1	1_1_1_1_1_0_1_0_0_1_1_1_0.205	2	2	2	2	2	2	2	2	2	1	1	1	1
2243	10	80,170,798	80,972,944	1	0														
2244	10	80,972,945	81,133,475	2	1	1_0_1_1_1_0_1_0_0_0_1_0_0.237	1	1	1	2	2	2	2	2	2	1	1	1	1
2245	10	81,133,476	81,166,489	1	0														
2246	10	81,166,490	81,166,521	2	1	0_1_0_0_0_1_0_1_1_1_1_1_1.55	2	2	2	1	1	1	1	2	1	2	2	2	2
2247	10	81,166,522	81,167,649	3	2	0_1_0_0_0_0_0_0_0_1_1_1_1.279	3	3	3	1	1	1	1	3	1	3	2	2	2
2248	10	81,167,650	81,251,653	4	3	0_1_0_0_0_0_0_0_0_1_1_1_0.131	4	4	4	1	1	1	1	4	1	3	2	2	2
2249	10	81,251,654	81,252,890	3	2	0_1_0_0_0_0_0_0_0_1_1_1_0.131	3	3	3	1	1	1	1	3	1	2	1	1	1
2250	10	81,252,891	81,353,560	4	3	0_1_0_0_0_0_0_0_0_1_1_1_0.131	4	4	4	2	2	2	2	4	2	3	1	1	1
2251	10	81,353,561	81,458,737	4	4	0_1_0_0_0_0_0_0_0_1_1_1_0.131	4	4	4	1	1	1	1	4	1	3	2	2	2
2252	10	81,458,738	81,459,977	4	3	0_1_0_0_0_0_0_0_0_1_1_1_1.279	3	3	3	1	1	1	1	3	1	4	2	2	2
2253	10	81,459,978	81,485,187	3	2	0_1_0_0_0_0_0_0_0_1_1_1_1.279	3	3	3	1	1	1	1	3	1	3	2	2	2
2254	10	81,485,188	81,491,983	2	1	0_1_0_0_0_0_0_0_0_1_1_1_1.279	2	2	2	1	1	1	1	2	1	2	1	1	1
2255	10	81,491,984	81,733,080	3	2	0_1_0_0_0_0_0_0_0_1_1_1_1.279	3	3	3	2	2	2	2	3	2	3	1	1	1
2256	10	81,733,081	81,941,023	3	3	0_1_0_0_0_0_0_0_0_1_1_1_1.279	3	3	3	1	1	1	1	3	1	3	2	2	2
2257	10	81,941,024	81,979,617	3	2	0_1_0_0_0_0_0_0_0_1_1_1_1.279	3	3	3	1	1	1	1	3	1	3	2	2	2
2258	10	81,979,618	82,032,284	2	1	0_1_0_0_0_1_0_1_1_1_1_1_1.88	2	2	2	1	1	1	1	2	1	2	2	2	2
2259	10	82,032,285	82,036,050	1	0														
2260	10	82,036,051	82,087,041	2	1	0_1_0_0_0_0_0_0_0_1_1_1_0.45	2	2	2	1	1	1	1	2	1	1	1	1	1
2261	10	82,087,042	82,132,596	3	2	0_0_0_0_0_0_0_0_0_0_0_0_1.155	2	2	2	1	1	1	1	2	1	3	1	1	1
2262	10	82,132,597	82,225,577	4	3	0_0_0_0_0_0_0_0_0_0_0_0_1.155	3	3	3	2	2	2	2	3	2	4	1	1	1
2263	10	82,225,578	82,259,028	4	4	0_0_0_0_0_0_0_0_0_0_0_0_1.155	3	3	3	2	2	2	2	3	2	4	1	1	1
2264	10	82,259,029	82,265,123	4	3	0_0_0_0_0_0_0_0_0_0_0_0_1.155	3	3	3	2	2	2	2	3	2	4	1	1	1
2265	10	82,265,124	82,357,621	4	4	0_0_0_0_0_0_0_0_0_0_0_0_1.155	3	3	3	2	2	2	2	3	2	4	1	1	1
2266	10	82,357,622	82,439,740	3	3	0_0_0_0_0_0_0_0_0_0_0_0_1.155	2	2	2	2	2	2	2	2	2	3	1	1	1
2267	10	82,439,741	82,495,604	3	2	0_0_0_0_0_0_0_0_0_0_0_0_1.155	2	2	2	2	2	2	2	2	2	3	1	1	1
2268	10	82,495,605	82,711,368	4	3	0_0_0_0_0_0_0_0_0_0_0_0_1.155	3	3	3	2	2	2	2	3	2	4	1	1	1
2269	10	82,711,369	82,721,376	3	2	0_0_0_0_0_0_0_0_0_0_0_0_1.155	2	2	2	2	2	2	2	2	2	3	1	1	1
2270	10	82,721,377	82,722,957	4	3	0_0_0_0_0_0_0_0_0_0_0_0_1.155	2	2	2	3	3	3	3	3	3	4	1	1	1
2271	10	82,722,958	82,826,739	4	4	0_0_0_0_0_0_0_0_0_0_0_0_1.155	3	3	3	2	2	2	2	2	2	4	1	1	1
2272	10	82,826,740	82,832,195	4	3	0_0_0_0_0_0_0_0_0_0_0_0_1.155	2	2	2	3	3	3	3	3	3	4	1	1	1
2273	10	82,832,196	82,845,744	3	2	0_0_0_0_0_0_0_0_0_0_0_0_1.155	2	2	2	2	2	2	2	2	2	3	1	1	1
2274	10	82,845,745	82,963,527	2	1	1_1_1_1_1_0_1_0_0_1_1_1_0.929	2	2	2	2	2	2	2	2	2	1	1	1	1
2275	10	82,963,528	83,397,713	3	2	0_0_0_0_0_0_0_0_0_0_0_0_1.283	2	2	2	2	2	2	2	2	2	3	1	1	1
2276	10	83,397,714	83,441,559	3	3	0_0_0_0_0_0_0_0_0_0_0_0_1.283	2	2	2	2	2	2	2	2	2	3	1	1	1
2277	10	83,441,560	83,842,071	3	2	0_0_0_0_0_0_0_0_0_0_0_0_1.283	2	2	2	2	2	2	2	2	2	3	1	1	1
2278	10	83,842,072	83,925,935	2	1	1_1_1_1_1_0_1_0_0_1_1_1_0.929	2	2	2	2	2	2	2	2	2	1	1	1	1
2279	10	83,925,936	83,925,987	1	0														
2280	10	83,925,988	84,280,022	2	1	1_1_1_1_1_0_1_0_0_1_1_1_1.306	2	2	2	2	2	2	2	2	2	2	1	1	1
2281	10	84,280,023	86,523,135	1	0														
2282	10	86,523,136	86,568,651	2	1	0_1_0_0_0_1_0_1_1_1_1_1_1.41	2	2	2	1	1	1	1	2	1	2	2	2	2
2283	10	86,568,652	86,807,700	1	0														
2284	10	86,807,701	86,887,014	2	1	1_1_1_1_1_0_1_0_0_1_1_1_0.234	2	2	2	2	2	2	2	2	2	1	1	1	1
2285	10	86,887,015	87,140,569	3	2	0_1_0_0_0_0_0_0_0_1_1_1_0.106	3	3	3	2	2	2	2	3	2	1	1	1	1
2286	10	87,140,570	87,142,131	2	1	0_1_0_0_0_0_0_0_0_1_1_1_0.106	2	2	2	1	1	1	1	2	1	1	1	1	1
2287	10	87,142,132	87,149,039	3	2	0_1_0_0_0_0_0_0_0_1_1_1_0.106	3	3	3	1	1	1	1	3	1	2	2	2	2
2288	10	87,149,040	88,113,398	2	1	0_1_0_0_0_1_0_1_1_1_1_1_1.699	2	2	2	1	1	1	1	2	1	2	2	2	2

2289	10	88,113,399	88,175,415	3	2	0_1_0_0_0_0_0_0_0_1_1_1_0.261	3	3	3	1	1	1	1	3	1	2	2	2	2
2290	10	88,175,416	88,200,279	3	3	0_1_0_0_0_0_0_0_0_1_1_1_0.261	3	3	3	1	1	1	1	3	1	2	2	2	2
2291	10	88,200,280	88,561,651	3	2	0_1_0_0_0_0_0_0_0_1_1_1_0.261	3	3	3	2	2	2	2	3	2	1	1	1	1
2292	10	88,561,652	88,796,770	2	1	1_1_1_1_1_0_1_0_0_1_1_1_0.444	2	2	2	2	2	2	2	2	2	1	1	1	1
2293	10	88,796,771	90,926,536	1	0														
2294	10	90,926,537	91,788,350	2	1	0_0_0_0_0_0_0_0_0_0_1_0_0.795	1	1	1	1	1	1	1	2	1	1	1	1	1
2295	10	91,788,351	91,850,312	1	0														
2296	10	91,850,313	92,781,306	2	1	0_0_0_0_0_1_0_0_1_0_1_0_1.738	1	1	1	1	1	1	1	2	1	2	2	1	2
2297	10	92,781,307	92,853,672	1	0														
2298	10	92,853,673	94,909,906	2	1	0_0_0_0_0_1_0_0_1_0_1_0_1.183	1	1	1	1	1	1	1	2	1	2	2	1	2
2299	10	94,909,907	94,917,880	1	0														
2300	10	94,917,881	95,236,326	2	1	0_0_0_0_0_1_0_0_1_0_0_0_1.253	1	1	1	1	1	1	1	1	1	2	2	1	2
2301	10	95,236,327	95,246,089	1	0														
2302	10	95,246,090	95,635,485	2	1	0_0_0_0_0_0_0_1_0_0_1_0_0.309	1	1	1	1	1	1	1	2	1	1	1	2	1
2303	10	95,635,486	95,647,311	1	0														
2304	10	95,647,312	95,957,603	2	1	0_0_0_0_0_1_0_0_1_0_1_0_1.261	1	1	1	1	1	1	1	2	1	2	2	1	2
2305	10	95,957,604	95,959,562	1	0														
2306	10	95,959,563	96,458,756	2	1	0_0_0_0_0_1_0_0_1_0_0_0_1.102	1	1	1	1	1	1	1	1	1	2	2	1	2
2307	10	96,458,757	96,461,126	3	2	0_0_0_0_0_1_0_0_1_0_0_0_1.102	2	2	2	2	2	2	2	1	2	3	3	1	3
2308	10	96,461,127	97,373,864	3	3	0_0_0_0_0_0_0_1_0_0_1_0_0.439	1	1	1	1	1	1	1	3	1	2	2	3	2
2309	10	97,373,865	97,382,876	3	2	0_0_0_0_0_0_0_1_0_0_1_0_0.439	2	2	2	2	2	2	2	3	2	1	1	3	1
2310	10	97,382,877	98,423,826	2	1	1_1_1_1_1_0_1_0_0_1_0_1_0.149	2	2	2	2	2	2	2	1	2	1	1	1	1
2311	10	98,423,827	98,916,700	1	0														
2312	10	98,916,701	98,978,286	2	1	1_1_1_1_1_0_1_0_0_1_1_1_1.60	2	2	2	2	2	2	2	2	2	2	1	1	1
2313	10	98,978,287	99,044,054	1	0														
2314	10	99,044,055	99,174,931	2	1	0_1_0_0_0_1_0_1_1_1_0_1_0.52	2	2	2	1	1	1	1	1	1	1	2	2	2
2315	10	99,174,932	100,639,210	1	0														
2316	10	100,639,211	101,323,368	2	1	1_1_1_1_1_0_1_0_0_1_1_1_0.787	2	2	2	2	2	2	2	2	2	1	1	1	1
2317	10	101,323,369	101,377,188	1	0														
2318	10	101,377,189	107,491,702	2	1	1_1_1_1_1_0_1_0_0_1_1_1_0.745	2	2	2	2	2	2	2	2	2	1	1	1	1
2319	10	107,491,703	107,493,274	1	0														
2320	10	107,493,275	110,616,985	2	1	0_0_0_0_0_0_0_0_0_0_0_0_1.238	1	1	1	1	1	1	1	1	1	2	1	1	1
2321	11	897,929	4,338,346	2	1	1_0_1_1_1_0_0_0_0_1_0_1_1.93	1	2	2	2	2	2	2	1	1	2	1	1	1
2322	11	4,338,347	4,438,080	1	0														
2323	11	4,438,081	8,123,206	2	1	1_0_1_1_1_0_1_0_0_1_0_1_0.490	1	2	2	2	2	2	2	1	2	1	1	1	1
2324	11	8,123,207	11,609,689	1	0														
2325	11	11,609,690	12,107,880	2	1	0_1_0_0_0_0_0_0_0_1_1_1_0.589	2	2	2	1	1	1	1	2	1	1	1	1	1
2326	11	12,107,881	13,458,450	1	0														
2327	11	13,458,451	13,789,933	2	1	1_0_1_1_1_0_1_0_0_1_0_1_0.442	1	2	2	2	2	2	2	1	2	1	1	1	1
2328	11	13,789,934	14,228,495	1	0														
2329	11	14,228,496	14,228,509	2	1	1_1_1_1_1_1_1_0_1_1_1_1_1.40	2	2	2	2	2	2	2	2	2	2	2	1	2
2330	11	14,228,510	14,328,200	3	2	1_1_1_1_1_1_1_0_1_0_0_0_1.82	3	2	2	3	3	3	3	2	3	3	3	1	3
2331	11	14,328,201	14,330,915	2	1	1_1_1_1_1_1_1_0_1_0_0_0_1.82	2	1	1	2	2	2	2	1	2	2	2	1	2
2332	11	14,330,916	14,435,571	3	2	0_0_0_0_0_0_0_0_0_1_1_1_0.464	2	3	3	2	2	2	2	3	2	2	2	1	2
2333	11	14,435,572	14,578,561	4	3	0_0_0_0_0_0_0_0_0_1_1_1_0.464	2	4	4	2	2	2	2	4	2	3	3	1	3
2334	11	14,578,562	14,699,973	4	4	0_0_0_0_0_0_0_0_0_1_1_1_0.464	2	4	4	2	2	2	2	4	2	3	3	1	3
2335	11	14,699,974	14,785,870	4	5	0_0_0_0_0_0_0_0_0_1_1_1_0.464	1	4	4	1	1	1	1	4	1	3	3	2	3
2336	11	14,785,871	14,816,276	4	4	0_0_0_0_0_0_0_0_0_1_1_1_0.464	2	4	4	2	2	2	2	4	2	3	3	1	3
2337	11	14,816,277	14,946,677	4	3	0_0_0_0_0_0_0_0_0_1_1_1_0.464	2	4	4	2	2	2	2	4	2	3	3	1	3
2338	11	14,946,678	15,051,010	4	4	0_0_0_0_0_0_0_0_0_1_1_1_0.464	2	4	4	2	2	2	2	4	2	3	3	1	3
2339	11	15,051,011	15,204,854	4	3	0_0_0_0_0_0_0_0_0_1_1_1_0.464	2	4	4	2	2	2	2	4	2	3	3	1	3
2340	11	15,204,855	15,220,788	3	2	0_0_0_0_0_0_0_0_0_1_1_1_0.464	1	3	3	1	1	1	1	3	1	2	2	1	2
2341	11	15,220,789	15,225,651	2	1	0_0_0_0_0_1_0_0_1_0_0_0_1.277	1	1	1	1	1	1	1	1	1	2	2	1	2
2342	11	15,225,652	15,254,874	3	2	0_0_0_0_0_1_0_0_1_0_0_0_1.277	2	1	1	2	2	2	2	1	2	3	3	1	3
2343	11	15,254,875	15,254,930	2	1	0_0_0_0_0_1_0_0_1_0_0_0_1.277	1	1	1	1	1	1	1	1	1	2	2	1	2
2344	11	15,254,931	15,255,978	1	0														
2345	11	15,255,979	15,257,200	2	1	0_0_0_0_0_1_0_1_1_1_1_1_1.41	1	2	2	1	1	1	1	2	1	2	2	2	2
2346	11	15,257,201	15,306,641	3	2	0_0_0_0_0_1_0_1_1_1_1_1_1.41	1	3	3	1	1	1	1	3	1	3	2	3	2
2347	11	15,306,642	15,315,146	2	1	1_1_1_1_1_0_1_1_0_1_1_1_1.20	2	2	2	2	2	2	2	2	2	2	1	2	1
2348	11	15,315,147	15,518,172	1	0														
2349	11	15,518,173	15,725,176	2	1	0_0_0_0_0_0_0_1_0_1_1_1_1.308	1	2	2	1	1	1	1	2	1	2	1	2	1
2350	11	15,725,177	15,758,773	3	2	0_0_0_0_0_0_0_1_0_1_1_1_1.308	2	3	3	2	2	2	2	3	2	3	1	3	1
2351	11	15,758,774	15,762,342	3	3	0_0_0_0_0_0_0_1_0_1_1_1_1.308	1	3	3	1	1	1	1	3	1	3	2	3	2
2352	11	15,762,343	15,878,875	3	2	0_0_0_0_0_0_0_1_0_1_1_1_1.308	1	3	3	1	1	1	1	3	1	3	2	3	2
2353	11	15,878,876	16,136,887	2	1	0_0_0_0_0_0_0_1_0_1_1_1_1.308	1	2	2	1	1	1	1	2	1	2	1	2	1
2354	11	16,136,888	16,265,705	1	0														
2355	11	16,265,706	16,288,443	2	1	0_0_0_0_0_1_0_1_1_1_1_1_1.38	1	2	2	1	1	1	1	2	1	2	2	2	2
2356	11	16,288,444	16,307,971	3	2	0_0_0_0_0_1_0_0_1_1_1_1_0.147	1	3	3	1	1	1	1	3	1	2	3	2	3
2357	11	16,307,972	16,400,958	3	3	0_0_0_0_0_0_0_1_0_0_0_0_1.53	1	2	2	1	1	1	1	2	1	3	2	3	2
2358	11	16,400,959	16,532,521	3	2	0_0_0_0_0_0_0_1_0_0_0_0_1.53	1	2	2	1	1	1	1	2	1	3	2	3	2
2359	11	16,532,522	16,654,894	3	3	0_0_0_0_0_0_0_1_0_0_0_0_1.53	1	2	2	1	1	1	1	2	1	3	2	3	2
2360	11	16,654,895	16,655,216	3	2	0_0_0_0_0_1_0_0_1_1_1_1_0.147	1	3	3	1	1	1	1	3	1	2	3	2	3
2361	11	16,655,217	16,733,636	2	1	0_0_0_0_0_1_0_1_1_1_1_1_1.110	1	2	2	1	1	1	1	2	1	2	2	2	2
2362	11	16,733,637	16,734,840	3	2	0_0_0_0_0_1_0_0_1_1_1_1_0.245	1	3	3	1	1	1	1	3	1	2	3	2	3
2363	11	16,734,841	16,740,753	3	3	0_0_0_0_0_0_0_1_0_0_0_0_1.345	1	2	2	1	1	1	1	2	1	3	2	3	2
2364	11	16,740,754	17,045,631	3	2	0_0_0_0_0_0_0_1_0_0_0_0_1.345	1	2	2	1	1	1	1	2	1	3	2	3	2
2365	11	17,045,632	17,160,705	3	3	0_0_0_0_0_0_0_1_0_0_0_0_1.345	1	2	2	1	1	1	1	2	1	3	2	3	2
2366	11	17,160,706	17,267,253	3	2	0_0_0_0_0_0_0_1_0_0_0_0_1.345	1	2	2	1	1	1	1	2	1	3	2	3	2
2367	11	17,267,254	17,387,737	2	1	0_0_0_0_0_0_0_1_0_0_0_0_1.345	1	1	1	1	1	1	1	1	1	2	1	2	1
2368	11	17,387,738	17,387,887	1	0														
2369	11	17,387,888	17,838,401	2	1	0_0_0_0_0_1_1_1_1_1_1_1_1.577	1	2	2	1	1	1	1	2	2	2	2	2	2
2370	11	17,838,402	17,838,719	3	2	0_0_0_0_0_0_0_1_0_0_0_0_1.135	1	2	2	1	1	1	1	2	2	3	2	3	2
2371	11	17,838,720	18,079,151	3	3	0_0_0_0_0_0_0_1_0_0_0_0_1.135	1	2	2	1	1	1	1	2	2	3	2	3	2
2372	11	18,079,152	18,150,721	3	2	0_0_0_0_0_0_0_1_0_0_0_0_1.135	2	1	1	2	2	2	2	1	1	3	1	3	1
2373	11	18,150,722	18,283,406	2	1	1_1_1_1_1_0_0_1_0_0_0_0_1.548	2	1	1	2	2	2	2	1	1	2	1	2	1

2374	11	18,283,407	18,338,506	3	2	0_0_0_0_0_0_0_1_0_0_0_0_1.23	2	1	1	2	2	2	2	1	1	3	1	3	1
2375	11	18,338,507	18,433,286	2	1	1_1_1_1_1_0_0_1_0_0_0_0_1.548	2	1	1	2	2	2	2	1	1	2	1	2	1
2376	11	18,433,287	18,439,246	3	2	0_0_0_0_0_0_0_1_0_0_0_0_1.83	2	1	1	2	2	2	2	1	1	3	1	3	1
2377	11	18,439,247	18,470,862	3	3	0_0_0_0_0_0_0_1_0_0_0_0_1.83	1	2	2	1	1	1	1	2	2	3	2	3	2
2378	11	18,470,863	18,473,707	3	2	0_0_0_0_0_0_0_1_0_0_0_0_1.83	1	2	2	1	1	1	1	2	2	3	2	3	2
2379	11	18,473,708	18,706,651	2	1	0_0_0_0_0_1_1_1_1_1_1_1_1.105	1	2	2	1	1	1	1	2	2	2	2	2	2
2380	11	18,706,652	18,892,067	3	2	0_0_0_0_0_0_0_1_0_0_0_0_1.133	1	2	2	1	1	1	1	2	2	3	2	3	2
2381	11	18,892,068	18,901,120	3	3	0_0_0_0_0_0_0_1_0_0_0_0_1.133	1	2	2	1	1	1	1	2	2	3	2	3	2
2382	11	18,901,121	19,121,070	3	2	0_0_0_0_0_0_0_1_0_0_0_0_1.133	2	1	1	2	2	2	2	1	1	3	1	3	1
2383	11	19,121,071	19,127,836	2	1	0_0_0_0_0_0_0_1_0_0_0_0_1.133	1	1	1	1	1	1	1	1	1	2	1	2	1
2384	11	19,127,837	19,359,431	1	0														
2385	11	19,359,432	19,413,599	2	1	1_1_1_1_1_0_0_1_0_1_1_1_1.23	2	2	2	2	2	2	2	2	1	2	1	2	1
2386	11	19,413,600	19,414,200	1	0														
2387	11	19,414,201	20,640,854	2	1	0_0_0_0_0_1_1_1_1_1_1_1_1.789	1	2	2	1	1	1	1	2	2	2	2	2	2
2388	11	20,640,855	23,834,575	1	0														
2389	11	23,834,576	23,996,002	2	1	1_1_1_1_1_1_1_0_1_1_1_1_1.79	2	2	2	2	2	2	2	2	2	2	2	1	2
2390	11	23,996,003	27,225,176	1	0														
2391	11	27,225,177	31,225,226	2	1	1_1_1_1_1_0_0_0_0_1_1_1_0.457	2	2	2	2	2	2	2	2	1	1	1	1	1
2392	11	31,225,227	31,501,031	1	0														
2393	11	31,501,032	33,028,060	2	1	1_1_1_1_1_0_0_0_0_1_1_1_0.159	2	2	2	2	2	2	2	2	1	1	1	1	1
2394	11	33,028,061	33,028,339	1	0														
2395	11	33,028,340	37,538,621	2	1	1_1_1_1_1_0_1_0_0_1_1_1_0.441	2	2	2	2	2	2	2	2	2	1	1	1	1
2396	11	37,538,622	40,915,368	1	0														
2397	11	40,915,369	42,834,856	2	1	1_1_1_1_1_0_1_0_0_1_1_1_0.258	2	2	2	2	2	2	2	2	2	1	1	1	1
2398	11	42,834,857	42,837,031	1	0														
2399	11	42,837,032	45,179,746	2	1	1_1_1_1_1_0_1_0_0_1_1_1_1.203	2	2	2	2	2	2	2	2	2	2	1	1	1
2400	11	45,179,747	48,393,533	1	0														
2401	11	48,393,534	50,895,226	2	1	1_1_1_1_1_0_1_0_0_1_1_1_1.272	2	2	2	2	2	2	2	2	2	2	1	1	1
2402	11	50,895,227	54,915,491	1	0														
2403	11	54,915,492	55,265,536	2	1	0_0_0_0_0_0_0_0_0_0_0_0_1.375	1	1	1	1	1	1	1	1	1	2	1	1	1
2404	11	55,265,537	55,977,073	1	0														
2405	11	55,977,074	57,021,353	2	1	0_0_0_0_0_0_0_1_0_0_0_0_1.140	1	1	1	1	1	1	1	1	1	2	1	2	1
2406	11	57,021,354	57,029,281	3	2	0_0_0_0_0_0_0_1_0_0_0_0_1.140	2	2	2	2	2	2	2	2	2	3	1	3	1
2407	11	57,029,282	57,208,141	3	3	0_0_0_0_0_0_0_1_0_0_0_0_1.140	2	2	2	2	2	2	2	2	2	3	1	3	1
2408	11	57,208,142	57,212,655	3	2	0_0_0_0_0_0_0_1_0_0_0_0_1.140	2	2	2	2	2	2	2	2	2	3	1	3	1
2409	11	57,212,656	57,220,054	2	1	1_1_1_1_1_0_1_0_0_1_1_1_0.279	2	2	2	2	2	2	2	2	2	1	1	1	1
2410	11	57,220,055	57,220,501	3	2	1_1_1_1_1_0_1_0_0_1_1_1_0.279	3	3	3	3	3	3	3	3	3	2	1	1	1
2411	11	57,220,502	57,274,983	3	3	0_0_0_0_0_0_0_0_0_0_0_0_1.92	2	2	2	2	2	2	2	2	2	3	1	1	1
2412	11	57,274,984	57,472,962	3	2	0_0_0_0_0_0_0_0_0_0_0_0_1.92	2	2	2	2	2	2	2	2	2	3	1	1	1
2413	11	57,472,963	57,751,707	2	1	1_1_1_1_1_0_1_0_0_1_1_1_0.279	2	2	2	2	2	2	2	2	2	1	1	1	1
2414	11	57,751,708	57,757,062	3	2	1_1_1_1_1_0_1_0_0_1_1_1_0.279	3	3	3	3	3	3	3	3	3	2	1	2	1
2415	11	57,757,063	57,860,579	3	3	0_0_0_0_0_0_0_1_0_0_0_0_1.57	2	2	2	2	2	2	2	2	2	3	1	3	1
2416	11	57,860,580	57,906,976	3	2	1_1_1_1_1_0_1_0_0_1_1_1_0.279	3	3	3	3	3	3	3	3	3	2	1	2	1
2417	11	57,906,977	60,113,427	2	1	1_1_1_1_1_0_1_0_0_1_1_1_0.279	2	2	2	2	2	2	2	2	2	1	1	1	1
2418	11	60,113,428	60,220,782	3	2	1_1_1_1_1_0_1_0_0_1_1_1_0.279	3	3	3	3	3	3	3	3	3	2	1	1	1
2419	11	60,220,783	60,251,690	2	1	1_1_1_1_1_0_1_0_0_1_1_1_1.304	2	2	2	2	2	2	2	2	2	2	1	1	1
2420	11	60,251,691	60,394,515	3	2	1_1_1_1_1_0_1_0_0_1_1_1_0.273	3	3	3	3	3	3	3	3	3	2	1	1	1
2421	11	60,394,516	64,077,319	2	1	1_1_1_1_1_0_1_0_0_1_1_1_0.273	2	2	2	2	2	2	2	2	2	1	1	1	1
2422	11	64,077,320	64,079,897	1	0														
2423	11	64,079,898	64,094,093	2	1	0_0_0_0_0_1_0_0_1_0_0_0_1.31	1	1	1	1	1	1	1	1	1	2	2	1	2
2424	11	64,094,094	64,095,269	1	0														
2425	11	64,095,270	64,155,640	2	1	0_0_0_0_0_1_0_0_1_0_1_0_1.499	1	1	1	1	1	1	1	2	1	2	2	1	2
2426	11	64,155,641	64,977,054	3	2	0_0_0_0_0_1_0_0_1_0_1_0_1.499	2	2	2	2	2	2	2	3	2	3	3	1	3
2427	11	64,977,055	65,040,067	4	3	0_0_0_0_0_0_0_0_0_0_1_0_1.53	2	2	2	2	2	2	2	4	2	4	3	1	3
2428	11	65,040,068	65,306,799	3	2	0_0_0_0_0_0_0_0_0_0_1_0_1.53	1	1	1	1	1	1	1	3	1	3	2	1	2
2429	11	65,306,800	65,322,968	2	1	0_0_0_0_0_1_0_0_1_0_1_0_1.499	1	1	1	1	1	1	1	2	1	2	2	1	2
2430	11	65,322,969	65,362,507	1	0														
2431	11	65,362,508	65,631,023	2	1	1_1_1_1_1_0_1_0_0_1_1_1_0.138	2	2	2	2	2	2	2	2	2	1	1	1	1
2432	11	65,631,024	65,694,715	1	0														
2433	11	65,694,716	65,695,008	2	1	0_0_0_0_0_1_0_0_1_0_1_0_1.818	1	1	1	1	1	1	1	2	1	2	2	1	2
2434	11	65,695,009	67,135,183	3	2	0_0_0_0_0_0_0_0_0_0_1_0_1.943	1	1	1	1	1	1	1	3	1	3	2	1	2
2435	11	67,135,184	67,167,307	5	3	0_0_0_0_0_0_0_0_0_0_1_0_1.943	2	2	2	2	2	2	2	5	2	4	3	1	3
2436	11	67,167,308	67,220,491	3	2	0_0_0_0_0_0_0_0_0_0_1_0_1.943	1	1	1	1	1	1	1	3	1	3	2	1	2
2437	11	67,220,492	67,224,035	2	1	0_0_0_0_0_1_0_0_1_0_1_0_1.818	1	1	1	1	1	1	1	2	1	2	2	1	2
2438	11	67,224,036	67,224,103	1	0														
2439	11	67,224,104	67,236,737	2	1	1_1_1_1_1_0_1_1_0_1_1_1_1.30	2	2	2	2	2	2	2	2	2	2	1	2	1
2440	11	67,236,738	67,334,442	3	2	1_1_1_1_1_0_1_0_0_1_1_1_1.120	3	3	3	3	3	3	3	3	3	3	1	2	1
2441	11	67,334,443	67,375,988	2	1	1_1_1_1_1_0_1_0_0_1_1_1_1.120	2	2	2	2	2	2	2	2	2	2	1	1	1
2442	11	67,375,989	67,447,363	3	2	1_1_1_1_1_0_1_0_0_1_1_1_1.120	3	3	3	3	3	3	3	3	3	3	1	2	1
2443	11	67,447,364	67,472,812	2	1	1_1_1_1_1_0_1_0_0_1_1_1_1.120	2	2	2	2	2	2	2	2	2	2	1	1	1
2444	11	67,472,813	67,719,989	3	2	1_1_1_1_1_0_1_0_0_1_1_1_1.120	3	3	3	3	3	3	3	3	3	3	2	1	2
2445	11	67,719,990	68,250,685	3	3	1_1_1_1_1_0_1_0_0_1_1_1_1.120	3	3	3	3	3	3	3	3	3	3	1	2	1
2446	11	68,250,686	68,351,934	3	2	1_1_1_1_1_0_1_0_0_1_1_1_1.120	3	3	3	3	3	3	3	3	3	3	1	2	1
2447	11	68,351,935	68,597,933	3	3	1_1_1_1_1_0_1_0_0_1_1_1_1.120	3	3	3	3	3	3	3	3	3	3	1	2	1
2448	11	68,597,934	68,702,395	3	2	1_1_1_1_1_0_1_0_0_1_1_1_1.120	3	3	3	3	3	3	3	3	3	3	1	2	1
2449	11	68,702,396	68,914,876	3	3	1_1_1_1_1_0_1_0_0_1_1_1_1.120	3	3	3	3	3	3	3	3	3	3	1	2	1
2450	11	68,914,877	69,080,219	3	2	1_1_1_1_1_0_1_0_0_1_1_1_1.120	3	3	3	3	3	3	3	3	3	3	1	2	1
2451	11	69,080,220	69,205,945	3	3	1_1_1_1_1_0_1_0_0_1_1_1_1.120	3	3	3	3	3	3	3	3	3	3	1	2	1
2452	11	69,205,946	69,294,765	3	2	1_1_1_1_1_0_1_0_0_1_1_1_1.120	3	3	3	3	3	3	3	3	3	3	2	1	2
2453	11	69,294,766	69,295,619	3	3	1_1_1_1_1_0_1_0_0_1_1_1_1.120	3	3	3	3	3	3	3	3	3	3	1	2	1
2454	11	69,295,620	69,387,733	3	2	1_1_1_1_1_0_1_0_0_1_1_1_1.120	3	3	3	3	3	3	3	3	3	3	1	2	1
2455	11	69,387,734	69,559,401	3	3	1_1_1_1_1_0_1_0_0_1_1_1_1.120	3	3	3	3	3	3	3	3	3	3	1	2	1
2456	11	69,559,402	69,710,363	3	2	1_1_1_1_1_0_1_0_0_1_1_1_1.120	3	3	3	3	3	3	3	3	3	3	2	1	2
2457	11	69,710,364	69,809,566	3	3	1_1_1_1_1_0_1_0_0_1_1_1_1.120	3	3	3	3	3	3	3	3	3	3	1	2	1
2458	11	69,809,567	69,812,439	3	2	1_1_1_1_1_0_1_0_0_1_1_1_1.120	3	3	3	3	3	3	3	3	3	3	1	2	1

Disease Models & Mechanisms • Supplementary information

2459	11	69,812,440	69,841,397	2	1	1_1_1_1_1_0_1_1_0_1_1_1_1.99	2	2	2	2	2	2	2	2	2	2	1	2	1
2460	11	69,841,398	70,499,976	1	0														
2461	11	70,499,977	74,637,607	2	1	1_1_1_1_1_0_1_0_0_1_0_1_0.377	2	2	2	2	2	2	2	1	2	1	1	1	1
2462	11	74,637,608	74,639,869	1	0														
2463	11	74,639,870	76,427,756	2	1	1_1_1_1_1_0_1_0_0_1_1_1_0.275	2	2	2	2	2	2	2	2	2	1	1	1	1
2464	11	76,427,757	76,468,319	3	2	0_1_0_0_0_0_0_0_0_1_0_1_0.48	3	3	3	2	2	2	2	2	2	1	1	1	1
2465	11	76,468,320	76,564,838	2	1	1_1_1_1_1_0_1_0_0_1_1_1_0.275	2	2	2	2	2	2	2	2	2	1	1	1	1
2466	11	76,564,839	76,616,284	1	0														
2467	11	76,616,285	79,116,888	2	1	1_1_1_1_1_0_1_0_0_1_1_1_0.395	2	2	2	2	2	2	2	2	2	1	1	1	1
2468	11	79,116,889	79,120,312	3	2	0_1_0_0_0_0_0_0_0_1_1_1_0.47	3	3	3	2	2	2	2	3	2	1	1	1	1
2469	11	79,120,313	79,288,206	3	3	0_1_0_0_0_0_0_0_0_1_1_1_0.47	3	3	3	1	1	1	1	3	1	2	2	2	2
2470	11	79,288,207	79,298,181	3	2	0_1_0_0_0_1_0_1_1_1_1_1_1.195	3	3	3	1	1	1	1	3	1	2	2	2	2
2471	11	79,298,182	79,600,684	2	1	0_1_0_0_0_1_0_1_1_1_1_1_1.195	2	2	2	1	1	1	1	2	1	2	2	2	2
2472	11	79,600,685	79,721,971	1	0														
2473	11	79,721,972	80,670,619	2	1	0_0_0_0_0_0_0_0_0_0_0_0_1.113	1	1	1	1	1	1	1	1	1	2	1	1	1
2474	11	80,670,620	80,673,413	3	2	0_0_0_0_0_0_0_0_0_0_0_0_1.113	2	2	2	1	1	1	1	1	1	3	1	1	1
2475	11	80,673,414	80,811,570	3	3	0_0_0_0_0_0_0_0_0_0_0_0_1.113	2	2	2	1	1	1	1	1	1	3	1	1	1
2476	11	80,811,571	80,813,534	3	2	0_0_0_0_0_0_0_0_0_0_0_0_1.113	2	2	2	1	1	1	1	1	1	3	1	1	1
2477	11	80,813,535	81,025,513	2	1	0_1_0_0_0_0_0_0_0_1_0_1_0.512	2	2	2	1	1	1	1	1	1	1	1	1	1
2478	11	81,025,514	81,027,453	1	0														
2479	11	81,027,454	81,269,581	2	1	1_1_1_1_1_0_1_0_0_1_1_1_0.378	2	2	2	2	2	2	2	2	2	1	1	1	1
2480	11	81,269,582	81,274,362	1	0														
2481	11	81,274,363	81,802,603	2	1	0_1_0_0_0_0_0_0_0_1_0_1_0.493	2	2	2	1	1	1	1	1	1	1	1	1	1
2482	11	81,802,604	89,878,598	1	0														
2483	11	89,878,599	90,438,543	2	1	0_1_0_0_0_0_0_0_0_1_1_1_0.151	2	2	2	1	1	1	1	2	1	1	1	1	1
2484	12	458,548	1,947,251	2	1	1_1_1_1_1_0_1_0_0_1_1_1_0.676	2	2	2	2	2	2	2	2	2	1	1	1	1
2485	12	1,947,252	1,989,037	1	0														
2486	12	1,989,038	2,682,550	2	1	1_1_1_1_1_0_1_0_0_1_1_1_0.302	2	2	2	2	2	2	2	2	2	1	1	1	1
2487	12	2,682,551	2,828,466	1	0														
2488	12	2,828,467	4,126,734	2	1	1_1_1_1_1_0_1_0_0_1_1_1_0.88	2	2	2	2	2	2	2	2	2	1	1	1	1
2489	12	4,126,735	4,211,576	1	0														
2490	12	4,211,577	5,824,522	2	1	1_1_1_1_1_0_1_0_0_1_1_1_0.743	2	2	2	2	2	2	2	2	2	1	1	1	1
2491	12	5,824,523	5,939,727	1	0														
2492	12	5,939,728	6,251,455	2	1	1_1_1_1_1_0_1_0_0_1_1_1_0.451	2	2	2	2	2	2	2	2	2	1	1	1	1
2493	12	6,251,456	6,260,311	1	0														
2494	12	6,260,312	6,273,649	2	1	1_1_1_1_1_0_1_1_0_1_1_1_1.37	2	2	2	2	2	2	2	2	2	2	1	2	1
2495	12	6,273,650	6,274,095	1	0														
2496	12	6,274,096	6,385,313	2	1	1_1_1_1_1_0_1_0_0_1_1_1_1.89	2	2	2	2	2	2	2	2	2	2	1	1	1
2497	12	6,385,314	6,394,848	1	0														
2498	12	6,394,849	6,540,659	2	1	1_1_1_1_1_0_1_0_0_1_1_1_0.75	2	2	2	2	2	2	2	2	2	1	1	1	1
2499	12	6,540,660	6,572,832	1	0														
2500	12	6,572,833	6,594,429	2	1	1_1_1_1_1_0_1_1_0_1_1_1_1.23	2	2	2	2	2	2	2	2	2	2	1	2	1
2501	12	6,594,430	6,908,112	1	0														
2502	12	6,908,113	7,730,634	2	1	1_1_1_1_1_0_1_1_0_1_1_1_1.145	2	2	2	2	2	2	2	2	2	2	1	2	1
2503	12	7,730,635	7,739,812	1	0														
2504	12	7,739,813	8,408,484	2	1	1_1_1_1_1_0_0_1_0_1_1_1_1.510	2	2	2	2	2	2	2	2	1	2	1	2	1
2505	12	8,408,485	8,494,057	1	0														
2506	12	8,494,058	10,658,500	2	1	1_1_1_1_1_0_0_1_0_1_1_1_1.106	2	2	2	2	2	2	2	2	1	2	1	2	1
2507	12	10,658,501	11,322,440	1	0														
2508	12	11,322,441	13,355,993	2	1	1_1_1_1_1_1_1_0_1_1_1_1_1.920	2	2	2	2	2	2	2	2	2	2	2	1	2
2509	12	13,355,994	13,502,117	1	0														
2510	12	13,502,118	13,803,027	2	1	1_1_1_1_1_1_1_0_1_1_1_1_1.43	2	2	2	2	2	2	2	2	2	2	2	1	2
2511	12	13,803,028	14,614,237	1	0														
2512	12	14,614,238	16,250,464	2	1	1_1_1_1_1_0_0_1_0_1_1_1_1.119	2	2	2	2	2	2	2	2	1	2	1	2	1
2513	12	16,250,465	16,795,016	1	0														
2514	12	16,795,017	16,818,446	2	1	0_0_0_0_0_0_0_1_0_0_0_0_1.31	1	1	1	1	1	1	1	1	1	2	1	2	1
2515	12	16,818,447	16,878,306	1	0														
2516	12	16,878,307	19,344,899	2	1	0_0_0_0_0_0_0_0_0_0_0_0_1.142	1	1	1	1	1	1	1	1	1	2	1	1	1
2517	12	19,344,900	19,451,239	1	0														
2518	12	19,451,240	21,822,323	2	1	0_0_0_0_0_0_0_0_0_0_0_0_1.887	1	1	1	1	1	1	1	1	1	2	1	1	1
2519	12	21,822,324	21,849,995	3	2	0_0_0_0_0_0_0_0_0_0_0_0_1.887	2	2	2	2	2	2	2	2	2	3	1	1	1
2520	12	21,849,996	22,683,586	2	1	0_0_0_0_0_0_0_0_0_0_0_0_1.887	1	1	1	1	1	1	1	1	1	2	1	1	1
2521	12	22,683,587	22,684,289	1	0														
2522	12	22,684,290	25,038,167	2	1	1_1_1_1_1_0_1_0_0_1_1_1_1.187	2	2	2	2	2	2	2	2	2	2	1	1	1
2523	12	25,038,168	25,042,759	3	2	1_1_1_1_1_0_1_0_0_1_1_1_0.441	3	3	3	3	3	3	3	3	3	2	1	1	1
2524	12	25,042,760	25,257,890	3	3	0_0_0_0_0_0_0_0_0_0_0_0_1.132	2	2	2	2	2	2	2	2	2	3	1	1	1
2525	12	25,257,891	25,272,741	3	2	0_0_0_0_0_0_0_0_0_0_0_0_1.132	2	2	2	2	2	2	2	2	2	3	1	1	1
2526	12	25,272,742	25,669,131	2	1	1_1_1_1_1_0_1_0_0_1_1_1_0.441	2	2	2	2	2	2	2	2	2	1	1	1	1
2527	12	25,669,132	25,713,300	3	2	0_0_0_0_0_0_0_0_0_0_0_0_1.53	2	2	2	2	2	2	2	2	2	3	1	1	1
2528	12	25,713,301	25,714,525	2	1	0_0_0_0_0_0_0_0_0_0_0_0_1.53	1	1	1	1	1	1	1	1	1	2	1	1	1
2529	12	25,714,526	25,779,737	1	0														
2530	12	25,779,738	26,324,318	2	1	1_1_1_1_1_0_0_0_0_1_1_1_1.235	2	2	2	2	2	2	2	2	1	2	1	1	1
2531	12	26,324,319	26,353,117	3	2	1_1_1_1_1_0_0_0_0_1_1_1_0.91	3	3	3	3	3	3	3	3	1	2	1	1	1
2532	12	26,353,118	26,494,926	3	3	0_0_0_0_0_0_0_0_0_0_0_0_1.58	2	2	2	2	2	2	2	2	1	3	1	1	1
2533	12	26,494,927	26,496,256	3	2	1_1_1_1_1_0_0_0_0_1_1_1_0.91	3	3	3	3	3	3	3	3	1	2	1	1	1
2534	12	26,496,257	26,512,686	2	1	1_1_1_1_1_0_0_0_0_1_1_1_1.235	2	2	2	2	2	2	2	2	1	2	1	1	1
2535	12	26,512,687	26,524,545	3	2	1_1_1_1_1_0_0_0_0_1_1_1_0.438	3	3	3	3	3	3	3	3	1	2	1	1	1
2536	12	26,524,546	26,993,369	3	3	0_0_0_0_0_0_0_0_0_0_0_0_1.313	2	2	2	2	2	2	2	2	1	3	1	1	1
2537	12	26,993,370	27,046,165	3	2	1_1_1_1_1_0_0_0_0_1_1_1_0.438	3	3	3	3	3	3	3	3	1	2	1	1	1
2538	12	27,046,166	27,399,693	3	3	0_0_0_0_0_0_0_0_0_0_0_0_1.132	2	2	2	2	2	2	2	2	1	3	1	1	1
2539	12	27,399,694	27,434,605	3	2	0_0_0_0_0_0_0_0_0_0_0_0_1.132	2	2	2	2	2	2	2	2	1	3	1	1	1
2540	12	27,434,606	28,037,257	3	3	0_0_0_0_0_0_0_0_0_0_0_0_1.132	2	2	2	2	2	2	2	2	1	3	1	1	1
2541	12	28,037,258	28,038,287	3	2	0_0_0_0_0_0_0_0_0_0_0_0_1.132	2	2	2	2	2	2	2	2	1	3	1	1	1
2542	12	28,038,288	28,713,325	2	1	0_0_0_0_0_0_0_0_0_0_0_0_1.132	1	1	1	1	1	1	1	1	1	2	1	1	1
2543	12	28,713,326	28,716,110	1	0														

2544	12	28,716,111	28,725,112	2	1	1_1_1_1_1_0_0_0_0_1_1_1_1.20	2	2	2	2	2	2	2	2	1	2	1	1	1
2545	12	28,725,113	28,725,974	1	0														
2546	12	28,725,975	30,199,962	2	1	1_1_1_1_1_0_0_0_0_1_1_1_0.142	2	2	2	2	2	2	2	2	1	1	1	1	1
2547	12	30,199,963	30,989,808	1	0														
2548	12	30,989,809	31,802,204	2	1	0_0_0_0_0_0_0_0_0_0_0_0_1.884	1	1	1	1	1	1	1	1	1	2	1	1	1
2549	12	31,802,205	32,493,092	1	0														
2550	12	32,493,093	32,582,262	2	1	0_0_0_0_0_0_0_0_0_0_0_0_1.136	1	1	1	1	1	1	1	1	1	2	1	1	1
2551	12	32,582,263	32,637,971	1	0														
2552	12	32,637,972	32,823,627	2	1	0_0_0_0_0_0_0_0_0_0_0_0_1.321	1	1	1	1	1	1	1	1	1	2	1	1	1
2553	12	32,823,628	32,824,016	1	0														
2554	12	32,824,017	39,003,977	2	1	1_1_1_1_1_0_0_0_0_1_1_1_0.662	2	2	2	2	2	2	2	2	1	1	1	1	1
2555	12	39,003,978	43,636,993	1	0														
2556	12	43,636,994	44,475,395	2	1	1_1_1_1_1_0_1_0_0_1_1_1_0.357	2	2	2	2	2	2	2	2	2	1	1	1	1
2557	12	44,475,396	45,459,004	3	2	0_0_0_0_0_0_0_0_0_0_0_0_1.909	2	2	2	2	2	2	2	2	2	3	1	1	1
2558	12	45,459,005	45,897,077	2	1	0_0_0_0_0_0_0_0_0_0_0_0_1.909	1	1	1	1	1	1	1	1	1	2	1	1	1
2559	12	45,897,078	48,884,594	1	0														
2560	12	48,884,595	51,118,142	2	1	0_0_0_0_0_0_0_0_0_0_1_0_0.277	1	1	1	1	1	1	1	2	1	1	1	1	1
2561	12	51,118,143	51,548,633	1	0														
2562	12	51,548,634	52,448,394	2	1	0_0_0_0_0_0_0_0_0_0_1_0_0.609	1	1	1	1	1	1	1	2	1	1	1	1	1
2563	13	2,828,333	3,069,523	2	1	1_1_1_1_1_0_1_0_0_1_1_1_0.326	2	2	2	2	2	2	2	2	2	1	1	1	1
2564	13	3,069,524	17,918,825	1	0														
2565	13	17,918,826	18,333,325	2	1	0_0_0_0_0_0_0_1_0_0_0_0_1.518	1	1	1	1	1	1	1	1	1	2	1	2	1
2566	13	18,333,326	21,760,657	1	0														
2567	13	21,760,658	23,915,736	2	1	1_1_1_1_1_0_0_0_0_1_1_1_0.356	2	2	2	2	2	2	2	2	1	1	1	1	1
2568	13	23,915,737	28,134,940	1	0														
2569	13	28,134,941	36,034,022	2	1	1_1_1_1_1_0_0_0_0_1_1_1_0.636	2	2	2	2	2	2	2	2	1	1	1	1	1
2570	13	36,034,023	37,190,323	1	0														
2571	13	37,190,324	37,299,004	2	1	1_1_1_1_1_0_0_0_0_1_1_1_0.39	2	2	2	2	2	2	2	2	1	1	1	1	1
2572	13	37,299,005	37,412,788	1	0														
2573	13	37,412,789	40,088,452	2	1	1_1_1_1_1_0_0_0_0_1_1_1_0.328	2	2	2	2	2	2	2	2	1	1	1	1	1
2574	13	40,088,453	41,235,976	1	0														
2575	13	41,235,977	42,467,129	2	1	1_1_1_1_1_0_0_0_0_1_1_1_0.110	2	2	2	2	2	2	2	2	1	1	1	1	1
2576	13	42,467,130	44,123,508	1	0														
2577	13	44,123,509	52,371,821	2	1	1_1_1_1_1_0_1_0_0_1_1_1_0.971	2	2	2	2	2	2	2	2	2	1	1	1	1
2578	13	52,371,822	53,357,935	3	2	0_0_0_0_0_0_0_0_0_0_0_0_1.261	2	2	2	2	2	2	2	2	2	3	1	1	1
2579	13	53,357,936	55,182,841	2	1	0_0_0_0_0_0_0_0_0_0_0_0_1.261	1	1	1	1	1	1	1	1	1	2	1	1	1
2580	13	55,182,842	55,197,412	3	2	0_0_0_0_0_0_0_0_0_0_0_0_1.261	2	2	2	2	2	2	2	2	2	3	1	1	1
2581	13	55,197,413	55,273,045	3	3	0_0_0_0_0_0_0_0_0_0_0_0_1.261	2	2	2	2	2	2	2	2	2	3	1	1	1
2582	13	55,273,046	55,323,517	3	2	0_0_0_0_0_0_0_0_0_0_0_0_1.261	2	2	2	2	2	2	2	2	2	3	1	1	1
2583	13	55,323,518	55,377,710	3	3	0_0_0_0_0_0_0_0_0_0_0_0_1.261	2	2	2	2	2	2	2	2	2	3	1	1	1
2584	13	55,377,711	55,423,336	3	2	0_0_0_0_0_0_0_0_0_0_0_0_1.261	2	2	2	2	2	2	2	2	2	3	1	1	1
2585	13	55,423,337	55,437,848	3	3	0_0_0_0_0_0_0_0_0_0_0_0_1.261	2	2	2	2	2	2	2	2	2	3	1	1	1
2586	13	55,437,849	55,481,396	3	2	0_0_0_0_0_0_0_0_0_0_0_0_1.261	2	2	2	2	2	2	2	2	2	3	1	1	1
2587	13	55,481,397	57,415,458	2	1	1_1_1_1_1_0_1_0_0_1_1_1_0.289	2	2	2	2	2	2	2	2	2	1	1	1	1
2588	13	57,415,459	57,478,186	1	0														
2589	13	57,478,187	58,472,058	2	1	1_1_1_1_1_0_1_0_0_1_1_1_0.980	2	2	2	2	2	2	2	2	2	1	1	1	1
2590	13	58,472,059	58,472,212	1	0														
2591	13	58,472,213	59,806,499	2	1	0_0_0_0_0_0_0_1_0_0_0_0_1.185	1	1	1	1	1	1	1	1	1	2	1	2	1
2592	13	59,806,500	59,810,976	3	2	0_0_0_0_0_0_0_1_0_0_0_0_1.185	2	2	2	2	2	2	2	1	2	3	2	3	2
2593	13	59,810,977	60,326,775	3	3	0_0_0_0_0_0_0_0_0_0_1_0_0.606	1	1	1	1	1	1	1	3	1	2	1	2	1
2594	13	60,326,776	60,328,249	3	2	0_0_0_0_0_0_0_0_0_0_1_0_0.606	2	2	2	2	2	2	2	3	2	1	2	1	2
2595	13	60,328,250	60,328,341	4	3	0_0_0_0_0_0_0_0_0_0_1_0_0.606	3	3	3	3	3	3	3	4	3	1	2	1	2
2596	13	60,328,342	60,405,096	4	4	0_0_0_0_0_0_0_0_0_0_1_0_0.606	2	2	2	2	2	2	2	4	2	1	3	1	3
2597	13	60,405,097	60,407,760	4	3	0_0_0_0_0_0_0_0_0_0_1_0_0.606	2	2	2	2	2	2	2	4	2	1	3	1	3
2598	13	60,407,761	60,606,833	4	4	0_0_0_0_0_0_0_0_0_0_1_0_0.606	1	1	1	1	1	1	1	4	1	3	2	3	2
2599	13	60,606,834	60,610,515	4	3	0_0_0_0_0_0_0_0_0_0_1_0_0.606	2	2	2	2	2	2	2	4	2	3	1	3	1
2600	13	60,610,516	60,732,983	4	4	0_0_0_0_0_0_0_0_0_0_1_0_0.606	2	2	2	2	2	2	2	4	2	3	1	3	1
2601	13	60,732,984	60,842,057	4	5	0_0_0_0_0_0_0_0_0_0_1_0_0.606	2	2	2	2	2	2	2	4	2	3	1	3	1
2602	13	60,842,058	60,845,383	4	4	0_0_0_0_0_0_0_1_0_0_0_0_1.234	3	3	3	3	3	3	3	2	3	4	1	4	1
2603	13	60,845,384	60,928,872	3	3	1_1_1_1_1_0_1_0_0_1_0_1_0.410	3	3	3	3	3	3	3	2	3	2	1	2	1
2604	13	60,928,873	60,985,196	3	2	1_1_1_1_1_0_1_0_0_1_0_1_0.410	3	3	3	3	3	3	3	1	3	1	2	1	2
2605	13	60,985,197	61,258,121	3	3	1_1_1_1_1_0_1_0_0_1_0_1_0.410	3	3	3	3	3	3	3	2	3	2	1	2	1
2606	13	61,258,122	61,403,107	3	2	1_1_1_1_1_0_1_0_0_1_0_1_0.410	3	3	3	3	3	3	3	1	3	1	2	1	2
2607	13	61,403,108	61,480,490	2	1	1_1_1_1_1_1_1_0_1_1_0_1_0.697	2	2	2	2	2	2	2	1	2	1	2	1	2
2608	13	61,480,491	61,516,256	3	2	1_1_1_1_1_0_1_0_0_1_0_1_0.321	3	3	3	3	3	3	3	1	3	1	2	1	2
2609	13	61,516,257	61,815,530	3	3	1_1_1_1_1_0_1_0_0_1_0_1_0.321	3	3	3	3	3	3	3	2	3	2	1	2	1
2610	13	61,815,531	61,909,662	3	2	1_1_1_1_1_0_1_0_0_1_0_1_0.321	3	3	3	3	3	3	3	1	3	1	2	1	2
2611	13	61,909,663	61,910,852	4	3	0_0_0_0_0_0_0_0_0_0_1_0_0.288	3	3	3	3	3	3	3	4	3	1	2	1	2
2612	13	61,910,853	62,008,953	4	4	0_0_0_0_0_0_0_0_0_0_1_0_0.288	2	2	2	2	2	2	2	4	2	3	1	3	1
2613	13	62,008,954	62,012,428	4	3	0_0_0_0_0_0_0_0_0_0_1_0_0.288	2	2	2	2	2	2	2	4	2	3	1	3	1
2614	13	62,012,429	62,029,985	4	4	0_0_0_0_0_0_0_0_0_0_1_0_0.288	1	1	1	1	1	1	1	4	1	3	2	3	2
2615	13	62,029,986	62,046,930	4	5	0_0_0_0_0_0_0_0_0_0_1_0_0.288	1	1	1	1	1	1	1	4	1	3	2	3	2
2616	13	62,046,931	62,058,943	4	6	0_0_0_0_0_0_0_0_0_0_1_0_0.288	1	1	1	1	1	1	1	4	1	3	2	3	2
2617	13	62,058,944	62,113,011	4	7	0_0_0_0_0_0_0_0_0_0_1_0_0.288	1	1	1	1	1	1	1	4	1	3	2	3	2
2618	13	62,113,012	62,135,407	4	6	0_0_0_0_0_0_0_0_0_0_1_0_0.288	2	2	2	2	2	2	2	4	2	1	3	1	3
2619	13	62,135,408	62,156,146	4	5	0_0_0_0_0_0_0_0_0_0_1_0_0.288	2	2	2	2	2	2	2	4	2	1	3	1	3
2620	13	62,156,147	62,167,499	4	4	0_0_0_0_0_0_0_0_0_0_1_0_0.288	2	2	2	2	2	2	2	4	2	1	3	1	3
2621	13	62,167,500	62,217,558	3	3	0_0_0_0_0_0_0_0_0_0_1_0_0.288	1	1	1	1	1	1	1	3	1	1	2	1	2
2622	13	62,217,559	62,356,784	3	2	0_0_0_0_0_0_0_0_0_0_1_0_0.288	1	1	1	1	1	1	1	3	1	1	2	1	2
2623	13	62,356,785	62,493,417	2	1	0_0_0_0_0_0_0_0_0_0_1_0_0.288	1	1	1	1	1	1	1	2	1	1	1	1	1
2624	13	62,493,418	62,493,944	1	0														
2625	13	62,493,945	62,561,264	2	1	1_1_1_1_1_1_1_0_1_1_0_1_0.89	2	2	2	2	2	2	2	1	2	1	2	1	2
2626	13	62,561,265	62,765,449	3	2	1_1_1_1_1_0_1_0_0_1_0_1_0.105	3	3	3	3	3	3	3	1	3	1	2	1	2
2627	13	62,765,450	63,157,857	3	3	1_1_1_1_1_0_1_0_0_1_0_1_0.105	3	3	3	3	3	3	3	2	3	2	1	2	1
2628	13	63,157,858	63,206,515	4	4	0_0_0_0_0_0_0_0_0_0_1_0_0.664	3	3	3	3	3	3	3	4	3	2	1	2	1

2629	13	63,206,516	63,225,346	4	5	0_0_0_0_0_0_0_0_0_0_1_0_0.664	2	2	2	2	2	2	2	4	2	3	1	3	1
2630	13	63,225,347	63,253,279	4	4	0_0_0_0_0_0_0_0_0_0_1_0_0.664	2	2	2	2	2	2	2	4	2	3	1	3	1
2631	13	63,253,280	63,273,975	4	3	0_0_0_0_0_0_0_0_0_0_1_0_0.664	2	2	2	2	2	2	2	4	2	3	1	3	1
2632	13	63,273,976	63,276,758	3	2	0_0_0_0_0_0_0_0_0_0_1_0_0.664	2	2	2	2	2	2	2	3	2	1	1	1	1
2633	13	63,276,759	63,898,784	3	3	0_0_0_0_0_0_0_0_0_0_1_0_0.664	2	2	2	2	2	2	2	3	2	1	1	1	1
2634	13	63,898,785	63,912,711	3	2	0_0_0_0_0_0_0_0_0_0_1_0_0.664	2	2	2	2	2	2	2	3	2	1	1	1	1
2635	13	63,912,712	63,989,222	2	1	1_1_1_1_1_0_1_0_0_1_0_1_0.105	2	2	2	2	2	2	2	1	2	1	1	1	1
2636	13	63,989,223	63,996,936	3	2	1_1_1_1_1_0_1_0_0_1_0_1_0.105	3	3	3	3	3	3	3	2	3	1	1	1	1
2637	13	63,996,937	64,660,842	3	3	0_0_0_0_0_0_0_0_0_0_1_0_0.488	2	2	2	2	2	2	2	3	2	1	1	1	1
2638	13	64,660,843	64,738,340	3	2	0_0_0_0_0_0_0_0_0_0_1_0_0.488	2	2	2	2	2	2	2	3	2	1	1	1	1
2639	13	64,738,341	64,784,342	3	3	0_0_0_0_0_0_0_0_0_0_1_0_0.488	2	2	2	2	2	2	2	3	2	1	1	1	1
2640	13	64,784,343	64,784,511	3	2	0_0_0_0_0_0_0_0_0_0_1_0_0.488	2	2	2	2	2	2	2	3	2	1	1	1	1
2641	13	64,784,512	65,697,015	2	1	1_1_1_1_1_0_1_0_0_1_0_1_0.143	2	2	2	2	2	2	2	1	2	1	1	1	1
2642	13	65,697,016	65,697,039	3	2	1_1_1_1_1_0_1_0_0_1_0_1_0.143	3	3	3	3	3	3	3	2	3	1	1	1	1
2643	13	65,697,040	65,754,448	3	3	0_0_0_0_0_0_0_0_0_0_1_0_0.179	2	2	2	2	2	2	2	3	2	1	1	1	1
2644	13	65,754,449	65,804,787	3	2	0_0_0_0_0_0_0_0_0_0_1_0_0.179	2	2	2	2	2	2	2	3	2	1	1	1	1
2645	13	65,804,788	68,289,184	3	3	0_0_0_0_0_0_0_0_0_0_1_0_0.179	2	2	2	2	2	2	2	3	2	1	1	1	1
2646	13	68,289,185	68,347,380	3	2	0_0_0_0_0_0_0_0_0_0_1_0_0.179	2	2	2	2	2	2	2	3	2	1	1	1	1
2647	13	68,347,381	68,647,027	3	3	0_0_0_0_0_0_0_0_0_0_1_0_0.179	2	2	2	2	2	2	2	3	2	1	1	1	1
2648	13	68,647,028	68,649,523	3	2	1_1_1_1_1_0_1_0_0_1_0_1_0.284	3	3	3	3	3	3	3	2	3	1	1	1	1
2649	13	68,649,524	68,861,371	2	1	1_1_1_1_1_0_1_0_0_1_1_1_0.443	2	2	2	2	2	2	2	2	2	1	1	1	1
2650	13	68,861,372	68,862,530	3	2	1_0_1_1_1_0_1_0_0_0_1_0_0.154	2	2	2	3	3	3	3	3	3	1	1	1	1
2651	13	68,862,531	70,856,865	3	3	0_1_0_0_0_0_0_0_0_1_0_1_0.188	3	3	3	2	2	2	2	2	2	1	1	1	1
2652	13	70,856,866	70,870,437	3	2	0_1_0_0_0_0_0_0_0_1_0_1_0.188	3	3	3	2	2	2	2	2	2	1	1	1	1
2653	13	70,870,438	70,879,670	2	1	1_1_1_1_1_0_1_0_0_1_1_1_0.443	2	2	2	2	2	2	2	2	2	1	1	1	1
2654	13	70,879,671	71,857,927	3	2	1_0_1_1_1_0_1_0_0_1_1_1_0.107	2	3	3	3	3	3	3	3	3	1	1	1	1
2655	13	71,857,928	71,956,442	3	3	1_0_1_1_1_0_1_0_0_1_1_1_0.107	1	3	3	3	3	3	3	3	3	2	2	2	2
2656	13	71,956,443	72,358,061	3	2	1_0_1_1_1_0_1_0_0_1_1_1_0.107	2	3	3	3	3	3	3	3	3	1	1	1	1
2657	13	72,358,062	72,510,232	2	1	1_1_1_1_1_0_1_0_0_1_1_1_0.443	2	2	2	2	2	2	2	2	2	1	1	1	1
2658	13	72,510,233	72,511,512	1	0														
2659	13	72,511,513	73,126,298	2	1	1_0_1_1_1_1_1_1_1_1_1_1_1.290	1	2	2	2	2	2	2	2	2	2	2	2	2
2660	13	73,126,299	73,127,041	1	0														
2661	13	73,127,042	74,307,928	2	1	0_0_0_0_0_1_0_1_1_1_1_1_1.483	1	2	2	1	1	1	1	2	1	2	2	2	2
2662	13	74,307,929	74,308,802	1	0														
2663	13	74,308,803	74,309,635	2	1	0_0_0_0_0_0_0_0_0_0_1_1_1_0.141	1	2	2	1	1	1	1	2	1	1	1	1	1
2664	13	74,309,636	76,171,678	3	2	0_0_0_0_0_0_0_0_0_0_1_1_1_0.141	2	3	3	2	2	2	2	3	2	1	1	1	1
2665	13	76,171,679	76,172,959	2	1	1_1_1_1_1_0_1_0_0_1_1_1_0.801	2	2	2	2	2	2	2	2	2	1	1	1	1
2666	13	76,172,960	76,178,512	3	2	1_1_1_1_1_0_1_0_0_1_0_1_0.20	3	3	3	3	3	3	3	2	3	1	1	1	1
2667	13	76,178,513	76,192,197	3	3	0_0_0_0_0_0_0_0_0_0_1_0_0.103	2	2	2	2	2	2	2	3	2	1	1	1	1
2668	13	76,192,198	76,197,031	3	2	0_0_0_0_0_0_0_0_0_0_1_0_0.103	2	2	2	2	2	2	2	3	2	1	1	1	1
2669	13	76,197,032	77,211,702	2	1	0_0_0_0_0_0_0_0_0_0_1_0_0.103	1	1	1	1	1	1	1	2	1	1	1	1	1
2670	13	77,211,703	77,212,150	1	0														
2671	13	77,212,151	79,095,643	2	1	1_1_1_1_1_0_1_0_0_1_0_1_0.155	2	2	2	2	2	2	2	1	2	1	1	1	1
2672	13	79,095,644	79,154,771	3	2	0_0_0_0_0_0_0_0_0_0_1_0_0.196	2	2	2	2	2	2	2	3	2	1	1	1	1
2673	13	79,154,772	79,175,114	2	1	0_0_0_0_0_0_0_0_0_0_1_0_0.196	1	1	1	1	1	1	1	2	1	1	1	1	1
2674	13	79,175,115	79,596,768	3	2	0_0_0_0_0_0_0_0_0_0_1_0_0.196	2	2	2	2	2	2	2	3	2	2	1	1	1
2675	13	79,596,769	79,665,597	2	1	1_1_1_1_1_0_1_0_0_1_0_1_1.852	2	2	2	2	2	2	2	1	2	2	1	1	1
2676	13	79,665,598	80,150,576	3	2	0_0_0_0_0_0_0_0_0_0_1_0_0.459	2	2	2	2	2	2	2	3	2	2	1	1	1
2677	13	80,150,577	80,719,211	3	3	0_0_0_0_0_0_0_0_0_0_1_0_0.459	2	2	2	2	2	2	2	3	2	2	1	1	1
2678	13	80,719,212	80,726,184	3	2	0_0_0_0_0_0_0_0_0_0_1_0_0.459	2	2	2	2	2	2	2	3	2	2	1	1	1
2679	13	80,726,185	80,728,857	2	1	1_1_1_1_1_0_1_0_0_1_1_1_1.133	2	2	2	2	2	2	2	2	2	2	1	1	1
2680	13	80,728,858	80,731,953	1	0														
2681	13	80,731,954	81,235,031	2	1	1_1_1_1_1_0_1_0_0_1_1_1_0.815	2	2	2	2	2	2	2	2	2	1	1	1	1
2682	13	81,235,032	81,239,199	1	0														
2683	13	81,239,200	81,239,304	2	1	1_1_1_1_1_0_0_0_0_1_1_1_0.428	2	2	2	2	2	2	2	2	1	1	1	1	1
2684	13	81,239,305	81,278,573	3	2	0_0_0_0_0_0_0_1_0_0_0_0_1.28	2	2	2	2	2	2	2	2	1	3	1	3	1
2685	13	81,278,574	81,541,800	2	1	1_1_1_1_1_0_0_0_0_1_1_1_0.428	2	2	2	2	2	2	2	2	1	1	1	1	1
2686	13	81,541,801	81,547,553	1	0														
2687	13	81,547,554	81,548,177	2	1	0_0_0_0_0_0_0_0_0_0_1_0_0.320	1	1	1	1	1	1	1	2	1	1	1	1	1
2688	13	81,548,178	81,553,644	3	2	0_0_0_0_0_0_0_0_0_0_1_0_0.320	2	2	2	2	2	2	2	3	2	1	1	1	1
2689	13	81,553,645	82,498,745	3	3	0_0_0_0_0_0_0_0_0_0_1_0_0.320	2	2	2	2	2	2	2	3	2	1	1	1	1
2690	13	82,498,746	82,505,896	3	2	1_1_1_1_1_0_1_0_0_1_0_1_0.610	3	3	3	3	3	3	3	2	3	1	1	1	1
2691	13	82,505,897	83,995,222	2	1	1_1_1_1_1_0_1_0_0_1_1_1_0.311	2	2	2	2	2	2	2	2	2	1	1	1	1
2692	13	83,995,223	83,995,847	3	2	0_0_0_0_0_0_0_0_0_0_1_0_0.200	2	2	2	2	2	2	2	3	2	1	1	1	1
2693	13	83,995,848	84,359,593	3	3	0_0_0_0_0_0_0_0_0_0_1_0_0.200	2	2	2	2	2	2	2	3	2	1	1	1	1
2694	13	84,359,594	84,361,389	3	2	0_0_0_0_0_0_0_0_0_0_1_0_0.200	2	2	2	2	2	2	2	3	2	1	1	1	1
2695	13	84,361,390	84,363,217	2	1	0_0_0_0_0_0_0_0_0_0_1_0_0.200	1	1	1	1	1	1	1	2	1	1	1	1	1
2696	13	84,363,218	84,490,742	1	0														
2697	13	84,490,743	84,507,471	2	1	1_1_1_1_1_0_1_0_0_1_0_1_0.32	2	2	2	2	2	2	2	1	2	1	1	1	1
2698	13	84,507,472	84,546,207	3	2	1_1_1_1_1_0_1_0_0_1_0_1_0.32	3	3	3	3	3	3	3	2	3	1	1	1	1
2699	13	84,546,208	84,686,128	2	1	1_1_1_1_1_0_1_0_0_1_1_1_0.187	2	2	2	2	2	2	2	2	2	1	1	1	1
2700	13	84,686,129	84,796,821	1	0														
2701	13	84,796,822	84,823,653	2	1	0_0_0_0_0_0_0_0_0_0_1_0_0.116	1	1	1	1	1	1	1	2	1	1	1	1	1
2702	13	84,823,654	84,827,457	3	2	0_0_0_0_0_0_0_0_0_0_1_0_0.116	2	2	2	2	2	2	2	3	2	1	1	1	1
2703	13	84,827,458	84,970,017	3	3	0_0_0_0_0_0_0_0_0_0_1_0_0.116	2	2	2	2	2	2	2	3	2	1	1	1	1
2704	13	84,970,018	84,970,798	3	2	1_1_1_1_1_0_1_0_0_1_0_1_0.321	3	3	3	3	3	3	3	2	3	1	1	1	1
2705	13	84,970,799	85,037,556	2	1	1_1_1_1_1_0_1_0_0_1_0_1_0.321	2	2	2	2	2	2	2	1	2	1	1	1	1
2706	13	85,037,557	85,043,328	3	2	0_0_0_0_0_0_0_0_0_0_1_0_0.365	2	2	2	2	2	2	2	3	2	1	1	1	1
2707	13	85,043,329	85,108,474	2	1	0_0_0_0_0_0_0_0_0_0_1_0_0.365	1	1	1	1	1	1	1	2	1	1	1	1	1
2708	13	85,108,475	85,112,270	3	2	0_0_0_0_0_0_0_0_0_0_1_0_0.365	2	2	2	2	2	2	2	3	2	1	1	1	1
2709	13	85,112,271	85,929,952	3	3	0_0_0_0_0_0_0_0_0_0_1_0_0.365	2	2	2	2	2	2	2	3	2	1	1	1	1
2710	13	85,929,953	86,162,574	3	2	1_1_1_1_1_0_1_0_0_1_0_1_0.946	3	3	3	3	3	3	3	2	3	1	1	1	1
2711	13	86,162,575	86,379,672	3	3	0_0_0_0_0_0_0_0_0_0_1_0_0.24	2	2	2	2	2	2	2	3	2	1	1	1	1
2712	13	86,379,673	86,412,529	3	2	1_1_1_1_1_0_1_0_0_1_0_1_0.946	3	3	3	3	3	3	3	2	3	1	1	1	1
2713	13	86,412,530	86,582,752	2	1	1_1_1_1_1_0_1_0_0_1_0_1_0.946	2	2	2	2	2	2	2	1	2	1	1	1	1

2714	13	86,582,753	86,587,030	1	0														
2715	13	86,587,031	87,420,926	2	1	0_0_0_0_0_0_0_0_0_0_1_0_0.429	1	1	1	1	1	1	1	2	1	1	1	1	1
2716	13	87,420,927	87,571,644	3	2	0_0_0_0_0_0_0_0_0_0_1_0_0.429	2	2	2	2	2	2	2	3	2	1	1	1	1
2717	13	87,571,645	88,663,027	2	1	1_1_1_1_1_0_1_0_0_1_0_1_0.100	2	2	2	2	2	2	2	1	2	1	1	1	1
2718	13	88,663,028	89,012,695	1	0														
2719	13	89,012,696	90,696,004	2	1	1_1_1_1_1_0_1_0_0_1_1_1_0.161	2	2	2	2	2	2	2	2	2	1	1	1	1
2720	13	90,696,005	90,803,845	3	2	0_0_0_0_0_0_0_0_0_0_0_0_1.120	2	2	2	2	2	2	2	2	2	3	1	1	1
2721	13	90,803,846	90,805,542	4	3	0_0_0_0_0_0_0_0_0_0_0_0_1.120	2	2	2	2	2	2	2	2	2	4	3	1	3
2722	13	90,805,543	90,844,431	4	4	0_0_0_0_0_0_0_0_0_0_0_0_1.120	2	2	2	2	2	2	2	2	2	4	3	1	3
2723	13	90,844,432	90,917,937	3	3	0_0_0_0_0_0_0_0_0_0_0_0_1.120	1	1	1	1	1	1	1	1	1	3	2	1	2
2724	13	90,917,938	90,918,329	3	2	0_0_0_0_0_1_0_0_1_0_0_0_1.32	1	1	1	1	1	1	1	1	1	3	2	1	2
2725	13	90,918,330	90,974,115	2	1	1_1_1_1_1_0_1_1_0_1_1_1_1.53	2	2	2	2	2	2	2	2	2	2	1	2	1
2726	13	90,974,116	92,736,878	1	0														
2727	13	92,736,879	92,973,201	2	1	1_1_1_1_1_0_1_1_0_1_1_1_1.93	2	2	2	2	2	2	2	2	2	2	1	2	1
2728	13	92,973,202	92,975,652	1	0														
2729	13	92,975,653	92,975,931	2	1	1_1_1_1_1_0_1_0_0_1_1_1_0.484	2	2	2	2	2	2	2	2	2	1	1	1	1
2730	13	92,975,932	93,260,155	3	2	0_0_0_0_0_0_0_1_0_0_0_0_1.572	2	2	2	2	2	2	2	2	2	3	1	3	1
2731	13	93,260,156	93,309,387	3	3	0_0_0_0_0_0_0_1_0_0_0_0_1.572	2	2	2	2	2	2	2	2	2	3	1	3	1
2732	13	93,309,388	93,669,032	3	2	0_0_0_0_0_0_0_1_0_0_0_0_1.572	2	2	2	2	2	2	2	2	2	3	1	3	1
2733	13	93,669,033	93,858,151	2	1	1_1_1_1_1_0_1_0_0_1_1_1_0.484	2	2	2	2	2	2	2	2	2	1	1	1	1
2734	13	93,858,152	94,029,215	3	2	0_0_0_0_0_0_0_1_0_0_0_0_1.316	2	2	2	2	2	2	2	2	2	3	1	3	1
2735	13	94,029,216	94,148,408	2	1	0_0_0_0_0_0_0_1_0_0_0_0_1.316	1	1	1	1	1	1	1	1	1	2	1	2	1
2736	13	94,148,409	94,152,173	1	0														
2737	13	94,152,174	94,227,017	2	1	0_0_0_0_0_0_0_0_0_0_0_0_1.321	1	1	1	1	1	1	1	1	1	2	1	1	1
2738	13	94,227,018	94,415,425	3	2	0_0_0_0_0_0_0_0_0_0_0_0_1.321	2	2	2	2	2	2	2	2	2	3	1	1	1
2739	13	94,415,426	94,420,335	2	1	1_1_1_1_1_0_1_0_0_1_1_1_0.784	2	2	2	2	2	2	2	2	2	1	1	1	1
2740	13	94,420,336	95,712,384	3	2	0_0_0_0_0_1_0_0_1_0_0_0_1.474	2	2	2	2	2	2	2	2	2	3	3	1	3
2741	13	95,712,385	95,718,059	2	1	0_0_0_0_0_1_0_0_1_0_0_0_1.474	1	1	1	1	1	1	1	1	1	2	2	1	2
2742	13	95,718,060	95,719,434	1	0														
2743	13	95,719,435	95,721,489	2	1	1_1_1_1_1_0_0_1_0_1_1_1_0.23	2	2	2	2	2	2	2	2	1	1	1	2	1
2744	13	95,721,490	95,747,553	3	2	1_1_1_1_1_0_0_0_0_1_1_1_0.337	3	3	3	3	3	3	3	3	1	1	1	2	1
2745	13	95,747,554	96,111,025	2	1	1_1_1_1_1_0_0_0_0_1_1_1_0.337	2	2	2	2	2	2	2	2	1	1	1	1	1
2746	13	96,111,026	96,418,902	3	2	1_1_1_1_1_0_0_0_0_1_1_1_0.337	3	3	3	3	3	3	3	3	1	1	1	2	1
2747	13	96,418,903	96,422,523	2	1	1_1_1_1_1_0_0_1_0_1_1_1_0.164	2	2	2	2	2	2	2	2	1	1	1	2	1
2748	13	96,422,524	96,480,527	1	0														
2749	13	96,480,528	96,503,014	2	1	0_0_0_0_0_0_0_0_0_0_0_0_1.567	1	1	1	1	1	1	1	1	1	2	1	1	1
2750	13	96,503,015	96,981,435	3	2	0_0_0_0_0_0_0_0_0_0_0_0_1.567	2	2	2	2	2	2	2	2	2	3	1	1	1
2751	13	96,981,436	96,982,551	2	1	1_1_1_1_1_0_1_0_0_1_1_1_0.274	2	2	2	2	2	2	2	2	2	1	1	1	1
2752	13	96,982,552	97,116,474	3	2	0_0_0_0_0_1_0_0_1_0_0_0_1.154	2	2	2	2	2	2	2	2	2	3	3	1	3
2753	13	97,116,475	97,123,565	2	1	0_0_0_0_0_1_0_0_1_0_0_0_1.154	1	1	1	1	1	1	1	1	1	2	2	1	2
2754	13	97,123,566	97,129,449	1	0														
2755	13	97,129,450	97,135,695	2	1	1_1_1_1_1_0_0_1_0_1_1_1_0.94	2	2	2	2	2	2	2	2	1	1	1	2	1
2756	13	97,135,696	97,200,235	3	2	1_1_1_1_1_0_0_0_0_1_1_1_0.21	3	3	3	3	3	3	3	3	1	1	1	2	1
2757	13	97,200,236	97,202,033	2	1	1_1_1_1_1_0_0_1_0_1_1_1_0.94	2	2	2	2	2	2	2	2	1	1	1	2	1
2758	13	97,202,034	97,317,677	1	0														
2759	13	97,317,678	97,317,996	2	1	0_0_0_0_0_0_0_0_0_0_0_0_1.943	1	1	1	1	1	1	1	1	1	2	1	1	1
2760	13	97,317,997	97,567,852	3	2	0_0_0_0_0_0_0_0_0_0_0_0_1.943	2	2	2	2	2	2	2	2	2	3	1	1	1
2761	13	97,567,853	100,078,415	2	1	0_0_0_0_0_0_0_0_0_0_0_0_1.943	1	1	1	1	1	1	1	1	1	2	1	1	1
2762	13	100,078,416	100,181,412	3	2	0_0_0_0_0_0_0_0_0_0_0_0_1.943	2	2	2	2	2	2	2	2	2	3	1	1	1
2763	13	100,181,413	100,554,686	2	1	0_0_0_0_0_0_0_0_0_0_0_0_1.943	1	1	1	1	1	1	1	1	1	2	1	1	1
2764	13	100,554,687	101,992,275	1	0														
2765	13	101,992,276	102,077,064	2	1	0_0_0_0_0_0_0_0_0_0_1_0_0.162	1	1	1	1	1	1	1	2	1	1	1	1	1
2766	13	102,077,065	102,376,344	1	0														
2767	13	102,376,345	104,703,058	2	1	0_0_0_0_0_0_0_0_0_0_1_0_1.208	1	1	1	1	1	1	1	2	1	2	1	1	1
2768	13	104,703,059	104,704,931	1	0														
2769	13	104,704,932	105,927,116	2	1	1_1_1_1_1_0_0_0_0_1_0_1_0.118	2	2	2	2	2	2	2	1	1	1	1	1	1
2770	13	105,927,117	106,466,673	1	0														
2771	13	106,466,674	107,224,672	2	1	1_1_1_1_1_0_0_0_0_1_1_1_0.118	2	2	2	2	2	2	2	2	1	1	1	1	1
2772	13	107,224,673	107,225,117	3	2	1_1_1_1_1_0_0_0_0_1_0_1_0.561	3	3	3	3	3	3	3	2	1	1	1	1	1
2773	13	107,225,118	107,369,788	3	3	0_0_0_0_0_0_0_0_0_0_1_0_0.61	2	2	2	2	2	2	2	3	1	1	1	1	1
2774	13	107,369,789	107,546,832	3	2	1_1_1_1_1_0_0_0_0_1_0_1_0.561	3	3	3	3	3	3	3	2	1	1	1	1	1
2775	13	107,546,833	107,762,230	3	3	0_0_0_0_0_0_0_0_0_0_1_0_0.73	2	2	2	2	2	2	2	3	1	1	1	1	1
2776	13	107,762,231	107,763,634	3	2	1_1_1_1_1_0_0_0_0_1_0_1_0.561	3	3	3	3	3	3	3	2	1	1	1	1	1
2777	13	107,763,635	107,764,739	2	1	1_1_1_1_1_0_0_0_0_1_0_1_0.561	2	2	2	2	2	2	2	1	1	1	1	1	1
2778	13	107,764,740	107,770,544	3	2	0_0_0_0_0_1_1_1_1_0_1_0_0.161	2	2	2	2	2	2	2	3	3	1	3	3	3
2779	13	107,770,545	108,197,726	3	3	0_0_0_0_0_0_0_0_0_0_0_0_1.216	1	1	1	1	1	1	1	2	2	3	2	2	2
2780	13	108,197,727	108,202,627	3	2	0_0_0_0_0_0_0_0_0_0_0_0_1.216	1	1	1	1	1	1	1	2	2	3	2	2	2
2781	13	108,202,628	108,746,923	2	1	0_0_0_0_0_0_0_0_0_0_0_0_1.216	1	1	1	1	1	1	1	1	1	2	1	1	1
2782	13	108,746,924	108,747,254	3	2	0_0_0_0_0_0_0_0_0_0_0_0_1.216	2	2	2	2	2	2	2	1	1	3	1	1	1
2783	13	108,747,255	108,855,438	3	3	0_0_0_0_0_0_0_0_0_0_0_0_1.216	1	1	1	1	1	1	1	2	2	3	2	2	2
2784	13	108,855,439	108,920,387	3	2	0_0_0_0_0_0_0_0_0_0_0_0_1.216	2	2	2	2	2	2	2	1	1	3	1	1	1
2785	13	108,920,388	110,925,631	2	1	0_0_0_0_0_0_0_0_0_0_0_0_1.216	1	1	1	1	1	1	1	1	1	2	1	1	1
2786	13	110,925,632	110,980,578	1	0														
2787	13	110,980,579	113,120,618	2	1	1_1_1_1_1_0_0_0_0_1_0_1_0.465	2	2	2	2	2	2	2	1	1	1	1	1	1
2788	13	113,120,619	113,396,836	1	0														
2789	13	113,396,837	113,533,743	2	1	0_0_0_0_0_0_0_0_0_0_1_0_0.107	1	1	1	1	1	1	1	2	1	1	1	1	1
2790	13	113,533,744	113,678,214	1	0														
2791	13	113,678,215	113,982,205	2	1	1_1_1_1_1_0_0_0_0_1_1_1_0.110	2	2	2	2	2	2	2	2	1	1	1	1	1
2792	14	341,465	3,046,076	2	1	0_0_0_0_0_0_0_0_0_0_0_0_1.173	1	1	1	1	1	1	1	1	1	2	1	1	1
2793	14	3,046,077	3,048,864	1	0														
2794	14	3,048,865	3,529,977	2	1	0_1_0_0_0_0_0_0_0_1_1_1_1.225	2	2	2	1	1	1	1	2	1	2	1	1	1
2795	14	3,529,978	3,659,551	1	0														
2796	14	3,659,552	3,741,787	2	1	0_0_0_0_0_0_0_0_0_0_0_0_1.96	1	1	1	1	1	1	1	1	1	2	1	1	1
2797	14	3,741,788	3,790,198	3	2	0_0_0_0_0_0_0_0_0_0_0_0_1.96	2	2	2	1	1	1	1	2	1	3	1	1	1
2798	14	3,790,199	4,099,292	2	1	0_1_0_0_0_0_0_0_0_1_1_1_0.152	2	2	2	1	1	1	1	2	1	1	1	1	1

Disease Models & Mechanisms • Supplementary information

2799	14	4,099,293	4,225,720	3	2	0_0_0_0_0_0_0_0_0_0_0_0_1.39	2	2	2	1	1	1	1	2	1	3	1	1	1
2800	14	4,225,721	4,307,626	2	1	0_1_0_0_0_0_0_0_0_1_1_1_0.152	2	2	2	1	1	1	1	2	1	1	1	1	1
2801	14	4,307,627	4,503,779	3	2	0_0_0_0_0_0_0_0_0_0_0_0_1.235	2	2	2	1	1	1	1	2	1	3	1	1	1
2802	14	4,503,780	4,527,556	2	1	0_1_0_0_0_0_0_0_0_1_1_1_0.152	2	2	2	1	1	1	1	2	1	1	1	1	1
2803	14	4,527,557	4,567,334	3	2	0_0_0_0_0_0_0_0_0_0_0_0_1.26	2	2	2	1	1	1	1	2	1	3	1	1	1
2804	14	4,567,335	4,598,676	3	3	0_0_0_0_0_0_0_0_0_0_0_0_1.26	2	2	2	1	1	1	1	2	1	3	1	1	1
2805	14	4,598,677	4,750,874	3	2	0_1_0_0_0_0_0_0_0_1_1_1_0.152	3	3	3	1	1	1	1	3	1	2	1	1	1
2806	14	4,750,875	4,757,472	3	3	0_0_0_0_0_0_0_0_0_0_0_0_1.103	2	2	2	1	1	1	1	2	1	3	1	1	1
2807	14	4,757,473	4,988,342	3	2	0_0_0_0_0_0_0_0_0_0_0_0_1.103	2	2	2	1	1	1	1	2	1	3	1	1	1
2808	14	4,988,343	5,109,548	2	1	0_1_0_0_0_0_0_0_0_1_1_1_0.152	2	2	2	1	1	1	1	2	1	1	1	1	1
2809	14	5,109,549	5,251,175	3	2	0_0_0_0_0_0_0_0_0_0_0_0_1.112	2	2	2	1	1	1	1	2	1	3	1	1	1
2810	14	5,251,176	5,290,480	3	3	0_0_0_0_0_0_0_0_0_0_0_0_1.112	2	2	2	1	1	1	1	2	1	3	1	1	1
2811	14	5,290,481	5,358,479	3	2	0_0_0_0_0_0_0_0_0_0_0_0_1.112	2	2	2	1	1	1	1	2	1	3	1	1	1
2812	14	5,358,480	5,780,102	2	1	0_1_0_0_0_0_0_0_0_1_1_1_0.152	2	2	2	1	1	1	1	2	1	1	1	1	1
2813	14	5,780,103	5,964,448	3	2	0_1_0_0_0_0_0_0_0_1_1_1_0.152	3	3	3	1	1	1	1	3	1	2	1	1	1
2814	14	5,964,449	6,123,152	2	1	0_1_0_0_0_0_0_0_0_1_1_1_1.156	2	2	2	1	1	1	1	2	1	2	1	1	1
2815	14	6,123,153	6,165,250	3	2	0_1_0_0_0_0_0_0_0_1_1_1_0.134	3	3	3	1	1	1	1	3	1	2	1	1	1
2816	14	6,165,251	6,497,026	3	3	0_0_0_0_0_0_0_0_0_0_0_0_1.37	2	2	2	1	1	1	1	2	1	3	1	1	1
2817	14	6,497,027	6,497,058	3	2	0_0_0_0_0_0_0_0_0_0_0_0_1.37	2	2	2	1	1	1	1	2	1	3	1	1	1
2818	14	6,497,059	6,523,710	2	1	0_1_0_0_0_0_0_0_0_1_1_1_0.134	2	2	2	1	1	1	1	2	1	1	1	1	1
2819	14	6,523,711	6,523,790	3	2	0_1_0_0_0_0_0_0_0_1_1_1_0.134	3	3	3	1	1	1	1	3	1	2	1	1	1
2820	14	6,523,791	6,902,430	3	3	0_0_0_0_0_0_0_0_0_0_0_0_1.331	2	2	2	1	1	1	1	2	1	3	1	1	1
2821	14	6,902,431	6,913,104	3	2	0_1_0_0_0_0_0_0_0_1_1_1_0.134	3	3	3	1	1	1	1	3	1	2	1	1	1
2822	14	6,913,105	6,980,349	2	1	0_1_0_0_0_0_0_0_0_1_1_1_0.134	2	2	2	1	1	1	1	2	1	1	1	1	1
2823	14	6,980,350	7,036,000	3	2	0_0_0_0_0_0_0_0_0_0_0_0_1.23	2	2	2	1	1	1	1	2	1	3	1	1	1
2824	14	7,036,001	7,238,384	2	1	0_1_0_0_0_0_0_0_0_1_1_1_0.134	2	2	2	1	1	1	1	2	1	1	1	1	1
2825	14	7,238,385	7,254,237	3	2	0_1_0_0_0_0_0_0_0_1_1_1_0.134	3	3	3	1	1	1	1	3	1	2	1	1	1
2826	14	7,254,238	7,587,509	3	3	0_0_0_0_0_0_0_0_0_0_0_0_1.217	2	2	2	1	1	1	1	2	1	3	1	1	1
2827	14	7,587,510	7,611,358	4	4	0_0_0_0_0_0_0_0_0_0_0_0_1.217	3	3	3	1	1	1	1	3	1	4	2	2	2
2828	14	7,611,359	7,768,060	3	3	0_0_0_0_0_0_0_0_0_0_0_0_1.217	2	2	2	1	1	1	1	2	1	3	1	1	1
2829	14	7,768,061	7,799,505	3	2	0_0_0_0_0_0_0_0_0_0_0_0_1.217	2	2	2	1	1	1	1	2	1	3	1	1	1
2830	14	7,799,506	7,842,641	2	1	0_1_0_0_0_0_0_0_0_1_1_1_0.134	2	2	2	1	1	1	1	2	1	1	1	1	1
2831	14	7,842,642	7,881,429	3	2	0_0_0_0_0_0_0_0_0_0_0_0_1.40	2	2	2	1	1	1	1	2	1	3	1	1	1
2832	14	7,881,430	7,922,054	2	1	0_1_0_0_0_0_0_0_0_1_1_1_0.134	2	2	2	1	1	1	1	2	1	1	1	1	1
2833	14	7,922,055	8,124,138	3	2	0_0_0_0_0_0_0_0_0_0_0_0_1.171	2	2	2	1	1	1	1	2	1	3	1	1	1
2834	14	8,124,139	8,124,412	4	3	0_0_0_0_0_0_0_0_0_0_0_0_1.171	3	3	3	1	1	1	1	3	1	4	2	2	2
2835	14	8,124,413	8,129,959	4	4	0_0_0_0_0_0_0_0_0_0_0_0_1.171	3	3	3	1	1	1	1	3	1	4	2	2	2
2836	14	8,129,960	8,136,230	4	5	0_0_0_0_0_0_0_0_0_0_0_0_1.171	3	3	3	1	1	1	1	3	1	4	2	2	2
2837	14	8,136,231	8,168,744	4	6	0_0_0_0_0_0_0_0_0_0_0_0_1.171	3	3	3	1	1	1	1	3	1	4	2	2	2
2838	14	8,168,745	8,182,872	4	5	0_0_0_0_0_0_0_0_0_0_0_0_1.171	3	3	3	1	1	1	1	3	1	4	2	2	2
2839	14	8,182,873	8,197,093	4	4	0_0_0_0_0_0_0_0_0_0_0_0_1.171	3	3	3	1	1	1	1	3	1	4	2	2	2
2840	14	8,197,094	8,211,262	4	5	0_0_0_0_0_0_0_0_0_0_0_0_1.171	3	3	3	1	1	1	1	3	1	4	2	2	2
2841	14	8,211,263	8,282,337	4	4	0_0_0_0_0_0_0_0_0_0_0_0_1.171	3	3	3	2	2	2	2	3	2	4	1	1	1
2842	14	8,282,338	8,308,362	3	3	0_1_0_0_0_0_0_0_0_1_1_1_0.134	3	3	3	2	2	2	2	3	2	2	1	1	1
2843	14	8,308,363	8,310,378	4	4	0_1_0_0_0_0_0_0_0_1_1_1_0.134	4	4	4	2	2	2	2	4	2	3	1	1	1
2844	14	8,310,379	8,328,829	4	5	0_1_0_0_0_0_0_0_0_1_1_1_0.134	4	4	4	1	1	1	1	4	1	3	2	2	2
2845	14	8,328,830	8,330,492	4	4	0_1_0_0_0_0_0_0_0_1_1_1_1.29	3	3	3	1	1	1	1	3	1	4	2	2	2
2846	14	8,330,493	8,331,848	4	4	0_1_0_0_0_1_0_1_1_1_1_1_1.107	3	3	3	1	1	1	1	3	1	4	2	2	2
2847	14	8,331,849	8,332,513	3	2	0_1_0_0_0_1_0_1_1_1_1_1_1.107	2	2	2	1	1	1	1	2	1	3	2	2	2
2848	14	8,332,514	8,380,186	3	3	0_0_0_0_0_0_0_0_0_0_0_0_1.141	2	2	2	1	1	1	1	2	1	3	2	2	2
2849	14	8,380,187	8,407,705	4	4	0_0_0_0_0_0_0_0_0_0_0_0_1.141	3	3	3	1	1	1	1	3	1	4	2	2	2
2850	14	8,407,706	8,422,033	4	3	0_0_0_0_0_0_0_0_0_0_0_0_1.141	3	3	3	1	1	1	1	3	1	4	2	2	2
2851	14	8,422,034	8,483,619	4	4	0_0_0_0_0_0_0_0_0_0_0_0_1.141	3	3	3	1	1	1	1	3	1	4	2	2	2
2852	14	8,483,620	8,488,730	4	5	0_0_0_0_0_0_0_0_0_0_0_0_1.141	3	3	3	1	1	1	1	3	1	4	2	2	2
2853	14	8,488,731	8,488,984	4	4	0_0_0_0_0_0_0_0_0_0_0_0_1.141	3	3	3	1	1	1	1	3	1	4	2	2	2
2854	14	8,488,985	8,492,172	3	3	0_0_0_0_0_0_0_0_0_0_0_0_1.141	2	2	2	1	1	1	1	2	1	3	1	1	1
2855	14	8,492,173	8,492,537	3	2	0_1_0_0_0_0_0_0_0_1_1_1_0.153	3	3	3	1	1	1	1	3	1	2	1	1	1
2856	14	8,492,538	8,507,216	2	1	0_1_0_0_0_0_0_0_0_1_1_1_1.81	2	2	2	1	1	1	1	2	1	2	1	1	1
2857	14	8,507,217	8,507,269	3	2	0_1_0_0_0_0_0_0_0_1_1_1_1.81	3	3	3	2	2	2	2	3	2	3	1	1	1
2858	14	8,507,270	8,508,248	2	1	1_1_1_1_1_0_1_0_0_1_1_1_1.41	2	2	2	2	2	2	2	2	2	2	1	1	1
2859	14	8,508,249	8,520,174	3	2	1_1_1_1_1_0_1_0_0_1_1_1_0.146	3	3	3	3	3	3	3	3	3	2	1	1	1
2860	14	8,520,175	8,587,949	3	3	0_0_0_0_0_0_0_0_0_0_0_0_1.187	2	2	2	2	2	2	2	2	2	3	1	1	1
2861	14	8,587,950	8,593,630	3	2	0_0_0_0_0_0_0_0_0_0_0_0_1.187	2	2	2	2	2	2	2	2	2	3	1	1	1
2862	14	8,593,631	8,690,841	2	1	0_0_0_0_0_0_0_0_0_0_0_0_1.187	1	1	1	1	1	1	1	1	1	2	1	1	1
2863	14	8,690,842	8,847,729	1	0														
2864	14	8,847,730	8,902,941	2	1	1_0_1_1_1_0_1_0_0_0_0_0_1.173	1	1	1	2	2	2	2	1	2	2	1	1	1
2865	14	8,902,942	8,903,129	1	0														
2866	14	8,903,130	9,130,542	2	1	1_1_1_1_1_0_1_0_0_1_1_1_1.594	2	2	2	2	2	2	2	2	2	2	1	1	1
2867	14	9,130,543	11,497,886	1	0														
2868	14	11,497,887	14,001,114	2	1	0_1_0_0_0_0_0_0_0_1_1_1_0.428	2	2	2	1	1	1	1	2	1	1	1	1	1
2869	14	14,001,115	14,040,801	1	0														
2870	14	14,040,802	14,501,561	2	1	1_1_1_1_1_0_1_0_0_1_1_1_0.571	2	2	2	2	2	2	2	2	2	1	1	1	1
2871	14	14,501,562	14,886,851	1	0														
2872	14	14,886,852	16,450,710	2	1	0_1_0_0_0_1_0_1_1_1_1_1_1.430	2	2	2	1	1	1	1	2	1	2	2	2	2
2873	14	16,450,711	17,770,773	1	0														
2874	14	17,770,774	18,080,937	2	1	0_1_0_0_0_1_0_1_1_1_1_1_1.218	2	2	2	1	1	1	1	2	1	2	2	2	2
2875	14	18,080,938	18,701,720	1	0														
2876	14	18,701,721	18,843,992	2	1	1_1_1_1_1_0_1_0_0_1_1_1_0.150	2	2	2	2	2	2	2	2	2	1	1	1	1
2877	14	18,843,993	18,864,279	3	2	0_1_0_0_0_0_0_0_0_1_1_1_0.340	3	3	3	2	2	2	2	3	2	1	1	1	1
2878	14	18,864,280	18,903,459	3	3	0_1_0_0_0_0_0_0_0_1_1_1_0.340	3	3	3	1	1	1	1	3	1	2	2	2	2
2879	14	18,903,460	18,915,589	3	2	0_1_0_0_0_0_0_0_0_1_1_1_0.340	3	3	3	2	2	2	2	3	2	1	1	1	1
2880	14	18,915,590	18,947,113	2	1	0_1_0_0_0_0_0_0_0_1_1_1_0.340	2	2	2	1	1	1	1	2	1	1	1	1	1
2881	14	18,947,114	18,965,175	3	2	0_1_0_0_0_0_0_0_0_1_1_1_0.340	3	3	3	2	2	2	2	3	2	1	1	1	1
2882	14	18,965,176	19,144,657	3	3	0_1_0_0_0_0_0_0_0_1_1_1_0.340	3	3	3	1	1	1	1	3	1	2	2	2	2
2883	14	19,144,658	19,147,968	3	2	0_1_0_0_0_0_0_0_0_1_1_1_0.340	3	3	3	2	2	2	2	3	2	1	1	1	1

2884	14	19,147,969	20,062,159	2	1	0_1_0_0_0_0_0_0_0_1_1_1_0.340	2	2	2	1	1	1	1	2	1	1	1	1	1
2885	14	20,062,160	20,095,335	3	2	0_0_0_0_0_1_0_0_1_0_0_0_1.145	2	2	2	1	1	1	1	2	1	3	3	1	3
2886	14	20,095,336	20,110,080	3	3	0_0_0_0_0_1_0_0_1_0_0_0_1.145	2	2	2	1	1	1	1	2	1	3	3	1	3
2887	14	20,110,081	20,252,665	3	2	0_0_0_0_0_1_0_0_1_0_0_0_1.145	2	2	2	1	1	1	1	2	1	3	3	1	3
2888	14	20,252,666	20,784,403	2	1	0_1_0_0_0_0_0_0_0_1_1_1_0.340	2	2	2	1	1	1	1	2	1	1	1	1	1
2889	14	20,784,404	21,377,185	3	2	0_1_0_0_0_0_0_0_0_1_1_1_0.340	3	3	3	2	2	2	2	3	2	1	1	1	1
2890	14	21,377,186	21,605,155	3	3	0_1_0_0_0_0_0_0_0_1_1_1_0.340	3	3	3	1	1	1	1	3	1	2	2	2	2
2891	14	21,605,156	21,742,885	3	2	0_1_0_0_0_0_0_0_0_1_1_1_0.340	3	3	3	2	2	2	2	3	2	1	1	1	1
2892	14	21,742,886	21,931,469	3	3	0_1_0_0_0_0_0_0_0_1_1_1_0.340	3	3	3	1	1	1	1	3	1	2	2	2	2
2893	14	21,931,470	22,006,496	3	2	0_1_0_0_0_0_0_0_0_1_1_1_0.340	3	3	3	2	2	2	2	3	2	1	1	1	1
2894	14	22,006,497	22,292,410	2	1	1_1_1_1_1_0_1_0_0_1_1_1_0.606	2	2	2	2	2	2	2	2	2	1	1	1	1
2895	14	22,292,411	22,343,962	1	0														
2896	14	22,343,963	22,925,142	2	1	0_1_0_0_0_1_0_1_1_1_1_1_1.142	2	2	2	1	1	1	1	2	1	2	2	2	2
2897	14	22,925,143	24,216,772	1	0														
2898	14	24,216,773	24,919,726	2	1	0_1_0_0_0_1_0_1_1_1_1_1_1.495	2	2	2	1	1	1	1	2	1	2	2	2	2
2899	14	24,919,727	27,379,651	1	0														
2900	14	27,379,652	28,088,103	2	1	0_1_0_0_0_1_0_1_1_1_1_1_1.489	2	2	2	1	1	1	1	2	1	2	2	2	2
2901	14	28,088,104	28,866,127	1	0														
2902	14	28,866,128	28,973,128	2	1	0_1_0_0_0_1_0_1_1_1_1_1_1.50	2	2	2	1	1	1	1	2	1	2	2	2	2
2903	14	28,973,129	30,308,150	1	0														
2904	14	30,308,151	30,391,978	2	1	1_1_1_1_1_0_0_0_0_1_1_1_0.82	2	2	2	2	2	2	2	2	1	1	1	1	1
2905	14	30,391,979	34,233,847	1	0														
2906	14	34,233,848	34,279,243	2	1	0_1_0_0_0_1_0_1_1_1_1_1_1.23	2	2	2	1	1	1	1	2	1	2	2	2	2
2907	14	34,279,244	34,360,474	1	0														
2908	14	34,360,475	34,680,455	2	1	0_1_0_0_0_1_0_1_1_1_1_1_1.23	2	2	2	1	1	1	1	2	1	2	2	2	2
2909	14	34,680,456	39,317,550	1	0														
2910	14	39,317,551	39,538,204	2	1	1_1_1_1_1_0_1_0_0_1_1_1_0.152	2	2	2	2	2	2	2	2	2	1	1	1	1
2911	14	39,538,205	43,726,203	1	0														
2912	14	43,726,204	43,964,268	2	1	1_1_1_1_1_0_1_0_0_1_1_1_0.207	2	2	2	2	2	2	2	2	2	1	1	1	1
2913	14	43,964,269	59,324,529	1	0														
2914	14	59,324,530	61,391,395	2	1	1_0_1_1_1_0_1_0_0_0_1_0_0.162	1	1	1	2	2	2	2	2	2	1	1	1	1
2915	14	61,391,396	62,247,649	1	0														
2916	14	62,247,650	62,470,642	2	1	0_0_0_0_0_1_1_1_1_1_0_1_1.328	1	2	2	1	1	1	1	1	2	2	2	2	2
2917	14	62,470,643	69,635,769	1	0														
2918	14	69,635,770	72,467,716	2	1	1_1_1_1_1_0_1_0_0_1_0_1_0.191	2	2	2	2	2	2	2	1	2	1	1	1	1
2919	14	72,467,717	72,791,315	1	0														
2920	14	72,791,316	73,158,778	2	1	0_0_0_0_0_0_0_0_0_0_1_0_0.186	1	1	1	1	1	1	1	2	1	1	1	1	1
2921	14	73,158,779	73,672,740	1	0														
2922	14	73,672,741	74,259,360	2	1	0_0_0_0_0_1_0_0_1_0_1_0_1.288	1	1	1	1	1	1	1	2	1	2	2	1	2
2923	14	74,259,361	74,297,250	3	2	0_0_0_0_0_1_0_0_1_0_0_0_1.876	1	1	1	1	1	1	1	2	1	3	3	1	3
2924	14	74,297,251	75,504,016	2	1	0_0_0_0_0_1_0_0_1_0_0_0_1.876	1	1	1	1	1	1	1	1	1	2	2	1	2
2925	14	75,504,017	77,518,852	1	0														
2926	14	77,518,853	78,439,490	2	1	0_0_0_0_0_0_0_0_0_0_0_0_1.146	1	1	1	1	1	1	1	1	1	2	1	1	1
2927	14	78,439,491	84,043,276	1	0														
2928	14	84,043,277	85,201,816	2	1	0_0_0_0_0_0_0_0_0_0_0_0_1.357	1	1	1	1	1	1	1	1	1	2	1	1	1
2929	14	85,201,817	87,334,416	1	0														
2930	14	87,334,417	87,736,061	2	1	0_0_0_0_0_0_0_0_0_0_0_0_1.336	1	1	1	1	1	1	1	1	1	2	1	1	1
2931	14	87,736,062	87,952,258	1	0														
2932	14	87,952,259	88,371,815	2	1	0_0_0_0_0_0_0_0_0_0_0_0_1.333	1	1	1	1	1	1	1	1	1	2	1	1	1
2933	14	88,371,816	88,466,745	1	0														
2934	14	88,466,746	88,883,462	2	1	0_0_0_0_0_0_0_0_0_0_0_0_1.463	1	1	1	1	1	1	1	1	1	2	1	1	1
2935	14	88,883,463	89,331,379	1	0														
2936	14	89,331,380	89,473,579	2	1	0_0_0_0_0_0_0_0_0_0_0_0_1.89	1	1	1	1	1	1	1	1	1	2	1	1	1
2937	14	89,473,580	98,041,999	1	0														
2938	14	98,042,000	98,324,960	2	1	0_0_0_0_0_0_0_1_0_0_0_0_1.424	1	1	1	1	1	1	1	1	1	2	1	2	1
2939	14	98,324,961	106,912,883	1	0														
2940	14	106,912,884	107,293,273	2	1	1_0_1_1_1_0_0_0_0_0_1_0_0.431	1	1	1	2	2	2	2	2	1	1	1	1	1
2941	14	107,293,274	109,508,055	1	0														
2942	14	109,508,056	109,566,026	2	1	0_0_0_0_0_0_0_0_0_0_0_0_1.70	1	1	1	1	1	1	1	1	1	2	1	1	1
2943	14	109,566,027	110,371,714	1	0														
2944	14	110,371,715	110,445,758	2	1	0_1_0_0_0_0_0_0_0_1_0_1_0.89	2	2	2	1	1	1	1	1	1	1	1	1	1
2945	14	110,445,759	111,344,471	1	0														
2946	14	111,344,472	111,511,017	2	1	0_1_0_0_0_0_0_0_0_1_0_1_0.284	2	2	2	1	1	1	1	1	1	1	1	1	1
2947	14	111,511,018	112,307,838	1	0														
2948	14	112,307,839	112,522,577	2	1	1_0_1_1_1_0_1_0_0_0_1_0_0.80	1	1	1	2	2	2	2	2	2	1	1	1	1
2949	14	112,522,578	112,690,666	1	0														
2950	14	112,690,667	115,478,732	2	1	1_0_1_1_1_0_1_0_0_0_1_0_0.197	1	1	1	2	2	2	2	2	2	1	1	1	1
2951	15	62,679	968,510	2	1	0_0_0_0_0_0_0_0_0_0_0_0_1.828	1	1	1	1	1	1	1	1	1	2	1	1	1
2952	15	968,511	1,261,274	3	2	0_0_0_0_0_0_0_0_0_0_0_0_1.828	1	1	1	1	1	1	1	2	1	3	1	1	1
2953	15	1,261,275	1,271,196	2	1	0_0_0_0_0_0_0_0_0_0_1_0_1.202	1	1	1	1	1	1	1	2	1	2	1	1	1
2954	15	1,271,197	1,448,234	3	2	0_0_0_0_0_0_0_0_0_0_1_0_0.174	1	1	1	1	1	1	1	3	1	2	1	1	1
2955	15	1,448,235	2,259,082	3	3	0_0_0_0_0_0_0_0_0_0_0_0_1.248	1	1	1	1	1	1	1	2	1	3	1	1	1
2956	15	2,259,083	2,301,559	3	2	0_0_0_0_0_0_0_0_0_0_1_0_0.174	1	1	1	1	1	1	1	3	1	2	1	1	1
2957	15	2,301,560	5,766,961	2	1	0_0_0_0_0_0_0_0_0_0_1_0_0.174	1	1	1	1	1	1	1	2	1	1	1	1	1
2958	15	5,766,962	6,007,288	3	2	0_0_0_0_0_0_0_0_0_0_0_0_1.542	1	1	1	1	1	1	1	2	1	3	1	1	1
2959	15	6,007,289	7,659,944	2	1	0_0_0_0_0_0_0_0_0_0_0_0_1.542	1	1	1	1	1	1	1	1	1	2	1	1	1
2960	15	7,659,945	8,208,716	1	0														
2961	15	8,208,717	8,726,614	2	1	0_0_0_0_0_0_0_0_0_0_0_0_1.796	1	1	1	1	1	1	1	1	1	2	1	1	1
2962	15	8,726,615	11,021,953	1	0														
2963	15	11,021,954	11,175,116	2	1	0_0_0_0_0_0_0_0_0_0_0_0_1.208	1	1	1	1	1	1	1	1	1	2	1	1	1
2964	15	11,175,117	11,236,475	3	2	0_0_0_0_0_0_0_0_0_0_0_0_1.208	2	2	2	2	2	2	2	1	1	3	1	1	1
2965	15	11,236,476	12,182,017	2	1	1_1_1_1_1_0_0_0_0_1_0_1_0.146	2	2	2	2	2	2	2	1	1	1	1	1	1
2966	15	12,182,018	12,246,723	1	0														
2967	15	12,246,724	12,739,211	2	1	1_1_1_1_1_0_0_0_0_1_0_1_0.703	2	2	2	2	2	2	2	1	1	1	1	1	1
2968	15	12,739,212	12,744,853	1	0														

Disease Models & Mechanisms • Supplementary information

2969	15	12,744,854	14,419,185	2	1	0_0_0_0_0_0_0_0_0_0_1_0_0.125	1	1	1	1	1	1	1	2	1	1	1	1	1
2970	15	14,419,186	14,426,573	1	0														
2971	15	14,426,574	15,075,988	2	1	1_1_1_1_1_0_0_0_0_1_0_1_0.100	2	2	2	2	2	2	2	1	1	1	1	1	1
2972	15	15,075,989	15,078,837	1	0														
2973	15	15,078,838	15,800,975	2	1	0_0_0_0_0_0_0_0_0_0_1_0_0.154	1	1	1	1	1	1	1	2	1	1	1	1	1
2974	15	15,800,976	15,820,558	3	2	0_0_0_0_0_0_0_0_0_0_1_0_0.154	2	2	2	2	2	2	2	3	1	1	1	1	1
2975	15	15,820,559	15,853,328	3	3	0_0_0_0_0_0_0_0_0_0_1_0_0.154	2	2	2	2	2	2	2	3	1	1	1	1	1
2976	15	15,853,329	15,853,998	3	2	0_0_0_0_0_0_0_0_0_0_1_0_0.154	2	2	2	2	2	2	2	3	1	1	1	1	1
2977	15	15,853,999	16,578,004	2	1	0_0_0_0_0_0_0_0_0_0_1_0_0.154	1	1	1	1	1	1	1	2	1	1	1	1	1
2978	15	16,578,005	17,831,056	1	0														
2979	15	17,831,057	20,055,666	2	1	0_0_0_0_0_0_0_0_0_0_0_0_1.140	1	1	1	1	1	1	1	1	1	2	1	1	1
2980	15	20,055,667	20,056,771	3	2	0_0_0_0_0_0_0_0_0_0_0_0_1.140	2	2	2	2	2	2	2	2	2	3	1	1	1
2981	15	20,056,772	20,163,490	3	3	0_0_0_0_0_0_0_0_0_0_0_0_1.140	2	2	2	2	2	2	2	2	2	3	1	1	1
2982	15	20,163,491	20,165,414	3	2	1_1_1_1_1_0_1_0_0_1_1_1_0.89	3	3	3	3	3	3	3	3	3	2	1	1	1
2983	15	20,165,415	20,166,839	2	1	1_1_1_1_1_0_1_0_0_1_1_1_1.266	2	2	2	2	2	2	2	2	2	2	1	1	1
2984	15	20,166,840	20,169,692	3	2	1_1_1_1_1_0_1_0_0_1_0_1_0.343	3	3	3	3	3	3	3	2	3	2	1	1	1
2985	15	20,169,693	20,477,754	3	3	0_0_0_0_0_0_0_0_0_0_1_0_1.410	2	2	2	2	2	2	2	3	2	3	1	1	1
2986	15	20,477,755	20,496,645	3	2	0_0_0_0_0_0_0_0_0_0_1_0_1.410	2	2	2	2	2	2	2	3	2	3	1	1	1
2987	15	20,496,646	20,670,340	2	1	0_0_0_0_0_0_0_0_0_0_1_0_1.410	1	1	1	1	1	1	1	2	1	2	1	1	1
2988	15	20,670,341	20,671,751	1	0														
2989	15	20,671,752	23,675,496	2	1	0_1_0_0_0_0_0_0_0_1_1_1_1.555	2	2	2	1	1	1	1	2	1	2	1	1	1
2990	15	23,675,497	27,523,814	1	0														
2991	15	27,523,815	28,268,950	2	1	1_1_1_1_1_0_0_0_0_1_1_1_1.606	2	2	2	2	2	2	2	2	1	2	1	1	1
2992	15	28,268,951	33,031,879	1	0														
2993	15	33,031,880	33,783,295	2	1	0_1_0_0_0_0_0_0_0_1_0_1_0.662	2	2	2	1	1	1	1	1	1	1	1	1	1
2994	15	33,783,296	33,825,635	1	0														
2995	15	33,825,636	35,702,187	2	1	1_1_1_1_1_0_0_0_0_1_0_1_0.174	2	2	2	2	2	2	2	1	1	1	1	1	1
2996	15	35,702,188	35,720,952	3	2	0_0_0_0_0_1_0_0_1_0_1_0_0.33	2	2	2	2	2	2	2	3	1	1	3	1	3
2997	15	35,720,953	37,432,981	2	1	1_1_1_1_1_0_0_0_0_1_0_1_0.174	2	2	2	2	2	2	2	1	1	1	1	1	1
2998	15	37,432,982	37,958,534	1	0														
2999	15	37,958,535	42,328,568	2	1	0_1_0_0_0_0_0_0_0_1_0_1_0.525	2	2	2	1	1	1	1	1	1	1	1	1	1
3000	15	42,328,569	42,485,945	1	0														
3001	15	42,485,946	59,932,290	2	1	0_1_0_0_0_1_1_1_1_1_0_1_0.104	2	2	2	1	1	1	1	1	2	1	2	2	2
3002	15	59,932,291	59,934,144	1	0														
3003	15	59,934,145	60,184,577	2	1	0_1_0_0_0_1_0_1_1_1_0_1_0.284	2	2	2	1	1	1	1	1	1	1	2	2	2
3004	15	60,184,578	60,184,841	1	0														
3005	15	60,184,842	67,058,896	2	1	1_0_1_1_1_0_1_0_0_0_1_0_0.699	1	1	1	2	2	2	2	2	2	1	1	1	1
3006	15	67,058,897	67,060,335	1	0														
3007	15	67,060,336	68,697,344	2	1	0_1_0_0_0_0_0_0_0_1_0_1_0.197	2	2	2	1	1	1	1	1	1	1	1	1	1
3008	15	68,697,345	68,697,754	1	0														
3009	15	68,697,755	69,497,847	2	1	1_1_1_1_1_0_1_0_0_1_0_1_0.327	2	2	2	2	2	2	2	1	2	1	1	1	1
3010	15	69,497,848	69,507,131	1	0														
3011	15	69,507,132	69,554,453	2	1	1_1_1_1_1_0_1_0_0_1_1_1_0.900	2	2	2	2	2	2	2	2	2	1	1	1	1
3012	15	69,554,454	70,018,200	3	2	0_1_0_0_0_0_0_0_0_1_1_1_0.224	3	3	3	2	2	2	2	3	2	1	1	1	1
3013	15	70,018,201	71,165,907	2	1	1_1_1_1_1_0_1_0_0_1_1_1_0.900	2	2	2	2	2	2	2	2	2	1	1	1	1
3014	15	71,165,908	74,333,415	1	0														
3015	15	74,333,416	77,224,381	2	1	1_1_1_1_1_0_1_0_0_1_1_1_0.371	2	2	2	2	2	2	2	2	2	1	1	1	1
3016	15	77,224,382	78,237,092	1	0														
3017	15	78,237,093	81,321,093	2	1	1_1_1_1_1_0_1_0_0_1_1_1_1.430	2	2	2	2	2	2	2	2	2	2	1	1	1
3018	15	81,321,094	84,266,259	1	0														
3019	15	84,266,260	84,587,514	2	1	1_1_1_1_1_0_0_0_0_1_1_1_0.300	2	2	2	2	2	2	2	2	1	1	1	1	1
3020	15	84,587,515	84,841,589	1	0														
3021	15	84,841,590	85,323,430	2	1	1_1_1_1_1_0_0_0_0_1_1_1_0.639	2	2	2	2	2	2	2	2	1	1	1	1	1
3022	15	85,323,431	86,215,453	1	0														
3023	15	86,215,454	87,397,201	2	1	1_1_1_1_1_0_0_0_0_1_1_1_0.130	2	2	2	2	2	2	2	2	1	1	1	1	1
3024	15	87,397,202	88,767,570	1	0														
3025	15	88,767,571	88,956,841	2	1	1_1_1_1_1_0_0_0_0_1_1_1_1.357	2	2	2	2	2	2	2	2	1	2	1	1	1
3026	15	88,956,842	88,985,861	3	2	1_1_1_1_1_0_0_0_0_1_1_1_0.33	3	3	3	3	3	3	3	3	1	2	1	1	1
3027	15	88,985,862	89,342,891	2	1	1_1_1_1_1_0_0_0_0_1_1_1_1.357	2	2	2	2	2	2	2	2	1	2	1	1	1
3028	15	89,342,892	89,366,374	1	0														
3029	15	89,366,375	89,460,546	2	1	1_1_1_1_1_0_0_0_0_1_1_1_0.151	2	2	2	2	2	2	2	2	1	1	1	1	1
3030	15	89,460,547	89,464,014	1	0														
3031	15	89,464,015	89,538,315	2	1	1_1_1_1_1_0_0_1_0_1_1_1_0.101	2	2	2	2	2	2	2	2	1	1	1	2	1
3032	15	89,538,316	89,541,561	1	0														
3033	15	89,541,562	90,790,816	2	1	1_1_1_1_1_0_0_1_0_1_1_1_1.728	2	2	2	2	2	2	2	2	1	2	1	2	1
3034	15	90,790,817	90,793,063	1	0														
3035	15	90,793,064	91,779,941	2	1	1_1_1_1_1_0_0_0_0_1_1_1_1.599	2	2	2	2	2	2	2	2	1	2	1	1	1
3036	15	91,779,942	91,973,071	1	0														
3037	15	91,973,072	92,001,954	2	1	1_1_1_1_1_0_0_0_0_1_1_1_1.46	2	2	2	2	2	2	2	2	1	2	1	1	1
3038	15	92,001,955	93,385,456	1	0														
3039	15	93,385,457	93,516,882	2	1	1_1_1_1_1_0_0_0_0_1_1_1_1.72	2	2	2	2	2	2	2	2	1	2	1	1	1
3040	15	93,516,883	93,558,871	1	0														
3041	15	93,558,872	93,581,125	2	1	1_1_1_1_1_0_0_0_0_1_1_1_0.22	2	2	2	2	2	2	2	2	1	1	1	1	1
3042	15	93,581,126	93,875,201	1	0														
3043	15	93,875,202	94,563,211	2	1	1_1_1_1_1_0_0_0_0_1_1_1_1.454	2	2	2	2	2	2	2	2	1	2	1	1	1
3044	15	94,563,212	94,598,788	1	0														
3045	15	94,598,789	94,637,879	2	1	1_1_1_1_1_0_0_0_0_1_1_1_1.21	2	2	2	2	2	2	2	2	1	2	1	1	1
3046	15	94,637,880	94,638,534	1	0														
3047	15	94,638,535	94,659,841	2	1	1_1_1_1_1_0_0_0_0_1_1_1_0.46	2	2	2	2	2	2	2	2	1	1	1	1	1
3048	15	94,659,842	94,661,277	1	0														
3049	15	94,661,278	94,964,750	2	1	1_0_1_1_1_1_1_1_1_1_1_1_1.210	1	2	2	2	2	2	2	2	2	2	2	2	2
3050	15	94,964,751	94,965,611	1	0														
3051	15	94,965,612	96,051,825	2	1	1_1_1_1_1_0_0_0_0_1_1_1_0.137	2	2	2	2	2	2	2	2	1	1	1	1	1
3052	15	96,051,826	96,063,669	1	0														
3053	15	96,063,670	96,772,817	2	1	1_1_1_1_1_0_0_0_0_1_1_1_1.414	2	2	2	2	2	2	2	2	1	2	1	1	1

3054	15	96,772,818	96,855,867	1	0														
3055	15	96,855,868	97,556,389	2	1	1_1_1_1_1_0_0_0_0_1_1_1_1.630	2	2	2	2	2	2	2	2	1	2	1	1	1
3056	15	97,556,390	97,559,886	1	0														
3057	15	97,559,887	97,639,538	2	1	0_0_0_0_0_0_0_0_0_0_0_0_1.144	1	1	1	1	1	1	1	1	1	2	1	1	1
3058	15	97,639,539	97,660,602	3	2	0_0_0_0_0_0_0_0_0_0_0_0_1.144	2	2	2	2	2	2	2	2	1	3	1	1	1
3059	15	97,660,603	98,083,143	3	3	0_0_0_0_0_0_0_0_0_0_0_0_1.144	2	2	2	2	2	2	2	2	1	3	1	1	1
3060	15	98,083,144	98,164,729	3	2	0_0_0_0_0_0_0_0_0_0_0_0_1.144	2	2	2	2	2	2	2	2	1	3	1	1	1
3061	15	98,164,730	98,251,239	3	3	0_0_0_0_0_0_0_0_0_0_0_0_1.144	2	2	2	2	2	2	2	2	1	3	1	1	1
3062	15	98,251,240	98,284,184	3	2	0_0_0_0_0_0_0_0_0_0_0_0_1.144	2	2	2	2	2	2	2	2	1	3	1	1	1
3063	15	98,284,185	98,465,304	2	1	0_0_0_0_0_0_0_0_0_0_0_0_1.144	1	1	1	1	1	1	1	1	1	2	1	1	1
3064	15	98,465,305	98,643,740	3	2	0_0_0_0_0_0_0_0_0_0_0_0_1.144	2	2	2	2	2	2	2	2	1	3	1	1	1
3065	15	98,643,741	98,921,807	3	3	0_0_0_0_0_0_0_0_0_0_0_0_1.144	2	2	2	2	2	2	2	2	1	3	1	1	1
3066	15	98,921,808	99,072,496	3	2	0_0_0_0_0_0_0_0_0_0_0_0_1.144	2	2	2	2	2	2	2	2	1	3	1	1	1
3067	15	99,072,497	99,136,313	3	3	0_0_0_0_0_0_0_0_0_0_0_0_1.144	2	2	2	2	2	2	2	2	1	3	1	1	1
3068	15	99,136,314	99,183,536	3	2	0_0_0_0_0_0_0_0_0_0_0_0_1.144	2	2	2	2	2	2	2	2	1	3	1	1	1
3069	15	99,183,537	99,281,857	3	3	0_0_0_0_0_0_0_0_0_0_0_0_1.144	2	2	2	2	2	2	2	2	1	3	1	1	1
3070	15	99,281,858	99,443,841	3	2	0_0_0_0_0_0_0_0_0_0_0_0_1.144	2	2	2	2	2	2	2	2	1	3	1	1	1
3071	15	99,443,842	99,555,779	3	3	0_0_0_0_0_0_0_0_0_0_0_0_1.144	2	2	2	2	2	2	2	2	1	3	1	1	1
3072	15	99,555,780	99,738,051	3	2	0_0_0_0_0_0_0_0_0_0_0_0_1.144	2	2	2	2	2	2	2	2	1	3	1	1	1
3073	15	99,738,052	99,772,767	3	3	0_0_0_0_0_0_0_0_0_0_0_0_1.144	2	2	2	2	2	2	2	2	1	3	1	1	1
3074	15	99,772,768	99,777,462	3	2	0_0_0_0_0_0_0_0_0_0_0_0_1.144	2	2	2	2	2	2	2	2	1	3	1	1	1
3075	15	99,777,463	100,694,492	2	1	1_1_1_1_1_0_0_0_0_1_1_1_1.524	2	2	2	2	2	2	2	2	1	2	1	1	1
3076	15	100,694,493	100,857,542	1	0														
3077	15	100,857,543	101,290,608	2	1	1_1_1_1_1_0_0_0_0_1_1_1_1.287	2	2	2	2	2	2	2	2	1	2	1	1	1
3078	15	101,290,609	104,001,881	1	0														
3079	15	104,001,882	104,064,233	2	1	1_1_1_1_1_0_0_0_0_1_1_1_0.139	2	2	2	2	2	2	2	2	1	1	1	1	1
3080	15	104,064,234	104,101,488	3	2	1_0_1_1_1_0_0_0_0_1_1_1_0.171	2	3	3	3	3	3	3	3	1	1	1	1	1
3081	15	104,101,489	105,579,659	2	1	1_0_1_1_1_0_0_0_0_1_1_1_0.171	1	2	2	2	2	2	2	2	1	1	1	1	1
3082	15	105,579,660	105,589,899	1	0														
3083	15	105,589,900	106,558,598	2	1	1_1_1_1_1_0_0_0_0_1_1_1_0.141	2	2	2	2	2	2	2	2	1	1	1	1	1
3084	15	106,558,599	106,559,207	1	0														
3085	15	106,559,208	106,578,913	2	1	1_0_1_1_1_0_0_0_0_1_1_1_0.35	1	2	2	2	2	2	2	2	1	1	1	1	1
3086	15	106,578,914	106,580,541	1	0														
3087	15	106,580,542	108,580,236	2	1	1_0_1_1_1_1_1_1_1_1_1_1_1.463	1	2	2	2	2	2	2	2	2	2	2	2	2
3088	15	108,580,237	108,603,244	1	0														
3089	15	108,603,245	109,433,463	2	1	1_0_1_1_1_0_0_1_0_1_1_1_1.258	1	2	2	2	2	2	2	2	1	2	1	2	1
3090	15	109,433,464	109,438,274	3	2	0_0_0_0_0_0_0_1_0_0_0_0_1.160	1	2	2	2	2	2	2	2	1	3	1	3	1
3091	15	109,438,275	109,511,215	3	3	0_0_0_0_0_0_0_1_0_0_0_0_1.160	1	2	2	2	2	2	2	2	1	3	1	3	1
3092	15	109,511,216	109,693,038	3	2	0_0_0_0_0_0_0_1_0_0_0_0_1.160	1	2	2	2	2	2	2	2	1	3	1	3	1
3093	15	109,693,039	109,693,039	2	1	1_0_1_1_1_0_0_0_0_1_1_1_0.92	1	2	2	2	2	2	2	2	1	1	1	1	1
3094	15	109,693,040	109,695,282	1	0														
3095	15	109,695,283	111,161,452	2	1	1_0_1_1_1_0_0_1_0_1_1_1_1.742	1	2	2	2	2	2	2	2	1	2	1	2	1
3096	16	85,710	274,443	2	1	1_1_1_1_1_0_1_0_0_1_1_1_0.29	2	2	2	2	2	2	2	2	2	1	1	1	1
3097	16	274,444	1,140,495	1	0														
3098	16	1,140,496	1,614,763	2	1	0_0_0_0_0_1_0_0_1_0_0_0_1.404	1	1	1	1	1	1	1	1	1	2	2	1	2
3099	16	1,614,764	2,530,260	1	0														
3100	16	2,530,261	6,435,926	2	1	0_1_0_0_0_0_0_0_0_1_1_1_0.439	2	2	2	1	1	1	1	2	1	1	1	1	1
3101	16	6,435,927	6,904,004	1	0														
3102	16	6,904,005	9,596,520	2	1	0_1_0_0_0_0_0_0_0_1_1_1_0.296	2	2	2	1	1	1	1	2	1	1	1	1	1
3103	16	9,596,521	9,740,043	1	0														
3104	16	9,740,044	9,897,597	2	1	1_0_1_1_1_0_0_0_0_0_0_0_1.101	1	1	1	2	2	2	2	1	1	2	1	1	1
3105	16	9,897,598	9,968,979	1	0														
3106	16	9,968,980	11,631,028	2	1	1_1_1_1_1_0_0_0_0_1_1_1_1.116	2	2	2	2	2	2	2	2	1	2	1	1	1
3107	16	11,631,029	11,634,682	1	0														
3108	16	11,634,683	14,355,655	2	1	0_1_0_0_0_0_0_0_0_1_1_1_0.224	2	2	2	1	1	1	1	2	1	1	1	1	1
3109	16	14,355,656	14,405,132	1	0														
3110	16	14,405,133	15,145,246	2	1	0_1_0_0_0_0_0_0_0_1_1_1_0.950	2	2	2	1	1	1	1	2	1	1	1	1	1
3111	16	15,145,247	17,469,568	1	0														
3112	16	17,469,569	17,493,039	2	1	0_1_0_0_0_0_0_0_0_1_1_1_0.48	2	2	2	1	1	1	1	2	1	1	1	1	1
3113	16	17,493,040	17,798,400	1	0														
3114	16	17,798,401	18,032,256	2	1	0_1_0_0_0_0_0_0_0_1_1_1_0.285	2	2	2	1	1	1	1	2	1	1	1	1	1
3115	16	18,032,257	18,531,033	1	0														
3116	16	18,531,034	20,207,289	2	1	0_1_0_0_0_0_0_0_0_1_1_1_0.139	2	2	2	1	1	1	1	2	1	1	1	1	1
3117	16	20,207,290	20,458,400	1	0														
3118	16	20,458,401	21,119,208	2	1	0_1_0_0_0_0_0_0_0_1_1_1_0.143	2	2	2	1	1	1	1	2	1	1	1	1	1
3119	16	21,119,209	21,612,814	3	2	0_1_0_0_0_0_0_0_0_1_1_1_0.143	3	3	3	2	2	2	2	3	1	2	1	1	1
3120	16	21,612,815	22,487,122	3	3	0_1_0_0_0_0_0_0_0_1_1_1_0.143	3	3	3	2	2	2	2	3	1	2	1	1	1
3121	16	22,487,123	22,518,814	3	2	0_1_0_0_0_0_0_0_0_1_1_1_0.143	3	3	3	2	2	2	2	3	1	2	1	1	1
3122	16	22,518,815	22,769,067	3	3	0_1_0_0_0_0_0_0_0_1_1_1_0.143	3	3	3	2	2	2	2	3	1	2	1	1	1
3123	16	22,769,068	22,772,127	3	2	0_1_0_0_0_0_0_0_0_1_1_1_0.143	3	3	3	2	2	2	2	3	1	2	1	1	1
3124	16	22,772,128	23,042,096	2	1	0_1_0_0_0_0_0_0_0_1_1_1_0.143	2	2	2	1	1	1	1	2	1	1	1	1	1
3125	16	23,042,097	23,045,491	3	2	0_1_0_0_0_0_0_0_0_1_1_1_0.143	3	3	3	2	2	2	2	3	1	2	1	1	1
3126	16	23,045,492	23,160,140	3	3	0_1_0_0_0_0_0_0_0_1_1_1_0.143	3	3	3	2	2	2	2	3	1	2	1	1	1
3127	16	23,160,141	23,200,242	3	2	0_1_0_0_0_0_0_0_0_1_1_1_0.143	3	3	3	2	2	2	2	3	1	2	1	1	1
3128	16	23,200,243	23,282,296	2	1	0_1_0_0_0_0_0_0_0_1_1_1_0.143	2	2	2	1	1	1	1	2	1	1	1	1	1
3129	16	23,282,297	23,291,263	3	2	0_1_0_0_0_0_0_0_0_1_1_1_0.143	3	3	3	2	2	2	2	3	1	2	1	1	1
3130	16	23,291,264	23,440,734	2	1	1_0_1_1_1_0_0_0_0_0_0_0_1.171	1	1	1	2	2	2	2	1	1	2	1	1	1
3131	16	23,440,735	23,479,007	3	2	0_1_0_0_0_0_0_0_0_1_1_1_0.60	3	3	3	2	2	2	2	3	1	2	1	1	1
3132	16	23,479,008	23,479,294	4	3	0_1_0_0_0_0_0_0_0_1_1_1_0.60	4	4	4	3	3	3	3	4	1	2	1	1	1
3133	16	23,479,295	23,480,526	3	2	0_1_0_0_0_0_0_0_0_1_1_1_0.60	3	3	3	2	2	2	2	3	1	1	1	1	1
3134	16	23,480,527	23,480,715	2	1	1_1_1_1_1_0_0_0_0_1_1_1_0.240	2	2	2	2	2	2	2	2	1	1	1	1	1
3135	16	23,480,716	23,484,690	3	2	0_0_0_0_0_0_0_0_0_0_0_0_1.338	2	2	2	2	2	2	2	2	1	3	1	1	1
3136	16	23,484,691	23,861,331	3	3	0_0_0_0_0_0_0_0_0_0_0_0_1.338	2	2	2	2	2	2	2	2	1	3	1	1	1
3137	16	23,861,332	23,990,638	3	2	0_0_0_0_0_0_0_0_0_0_0_0_1.338	2	2	2	2	2	2	2	2	1	3	1	1	1
3138	16	23,990,639	24,013,734	2	1	1_1_1_1_1_0_0_0_0_1_1_1_0.240	2	2	2	2	2	2	2	2	1	1	1	1	1

Disease Models & Mechanisms • Supplementary information

3139	16	24,013,735	24,848,135	1	0													
3140	16	24,848,136	25,358,221	2	1	1_1_1_1_1_0_0_0_0_1_1_1_0.490	2	2	2	2	2	2	2	2	1	1	1	1	1
3141	16	25,358,222	25,446,382	1	0													
3142	16	25,446,383	25,694,240	2	1	1_1_1_1_1_0_0_0_0_1_1_1_0.146	2	2	2	2	2	2	2	2	1	1	1	1	1
3143	16	25,694,241	25,781,642	1	0													
3144	16	25,781,643	27,668,322	2	1	1_1_1_1_1_0_0_0_0_1_1_1_0.120	2	2	2	2	2	2	2	2	1	1	1	1	1
3145	16	27,668,323	27,839,879	1	0													
3146	16	27,839,880	46,518,881	2	1	1_1_1_1_1_0_1_0_0_1_1_1_0.207	2	2	2	2	2	2	2	2	2	1	1	1	1
3147	16	46,518,882	46,561,249	3	2	1_1_1_1_1_0_1_0_0_1_1_1_0.207	3	3	3	3	3	3	3	3	3	2	1	1	1
3148	16	46,561,250	52,851,800	2	1	1_1_1_1_1_0_1_0_0_1_1_1_0.207	2	2	2	2	2	2	2	2	2	1	1	1	1
3149	16	52,851,801	53,027,693	1	0													
3150	16	53,027,694	57,080,482	2	1	1_1_1_1_1_0_1_0_0_1_0_1_0.300	2	2	2	2	2	2	2	1	2	1	1	1	1
3151	16	57,080,483	57,084,290	1	0													
3152	16	57,084,291	59,334,739	2	1	0_1_0_0_0_0_0_0_0_1_0_1_0.247	2	2	2	1	1	1	1	1	1	1	1	1	1
3153	16	59,334,740	60,342,602	1	0													
3154	16	60,342,603	61,386,960	2	1	0_0_0_0_0_0_0_0_0_0_0_0_1.130	1	1	1	1	1	1	1	1	1	2	1	1	1
3155	16	61,386,961	63,846,552	1	0													
3156	16	63,846,553	64,522,697	2	1	1_1_1_1_1_0_0_0_0_1_1_1_0.977	2	2	2	2	2	2	2	2	1	1	1	1	1
3157	16	64,522,698	64,717,657	1	0													
3158	16	64,717,658	65,581,559	2	1	1_1_1_1_1_0_0_0_0_1_1_1_0.805	2	2	2	2	2	2	2	2	1	1	1	1	1
3159	16	65,581,560	66,906,749	1	0													
3160	16	66,906,750	68,344,879	2	1	0_1_0_0_0_0_0_0_0_1_0_1_0.118	2	2	2	1	1	1	1	1	1	1	1	1	1
3161	16	68,344,880	68,346,239	1	0													
3162	16	68,346,240	69,505,207	2	1	0_1_0_0_0_0_0_0_0_1_1_1_0.147	2	2	2	1	1	1	1	2	1	1	1	1	1
3163	16	69,505,208	69,604,950	1	0													
3164	16	69,604,951	72,200,518	2	1	0_1_0_0_0_0_0_0_0_1_1_1_0.170	2	2	2	1	1	1	1	2	1	1	1	1	1
3165	16	72,200,519	79,805,404	1	0													
3166	16	79,805,405	79,807,775	2	1	1_1_1_1_1_0_1_0_0_1_1_1_0.146	2	2	2	2	2	2	2	2	2	1	1	1	1
3167	16	79,807,776	80,001,914	3	2	0_1_0_0_0_0_0_0_0_1_1_1_0.215	3	3	3	2	2	2	2	3	2	1	1	1	1
3168	16	80,001,915	80,037,650	3	3	0_1_0_0_0_0_0_0_0_1_1_1_0.215	3	3	3	1	1	1	1	3	1	2	2	2	2
3169	16	80,037,651	80,039,365	3	2	0_1_0_0_0_1_0_1_1_1_1_1_1.232	3	3	3	1	1	1	1	3	1	2	2	2	2
3170	16	80,039,366	80,253,229	2	1	0_1_0_0_0_1_0_1_1_1_1_1_1.232	2	2	2	1	1	1	1	2	1	2	2	2	2
3171	16	80,253,230	80,253,454	1	0													
3172	16	80,253,455	84,073,956	2	1	1_1_1_1_1_0_1_0_0_1_1_1_0.438	2	2	2	2	2	2	2	2	2	1	1	1	1
3173	16	84,073,957	84,202,847	3	2	0_0_0_0_0_0_0_0_0_0_0_0_1.215	2	2	2	2	2	2	2	2	2	3	1	1	1
3174	16	84,202,848	84,276,478	2	1	1_1_1_1_1_0_1_0_0_1_1_1_0.438	2	2	2	2	2	2	2	2	2	1	1	1	1
3175	16	84,276,479	84,458,629	3	2	0_0_0_0_0_0_0_0_0_0_0_0_1.87	2	2	2	2	2	2	2	2	2	3	1	1	1
3176	16	84,458,630	85,037,766	2	1	1_1_1_1_1_0_1_0_0_1_1_1_0.438	2	2	2	2	2	2	2	2	2	1	1	1	1
3177	16	85,037,767	85,164,276	1	0													
3178	16	85,164,277	90,243,818	2	1	1_1_1_1_1_0_1_0_0_1_1_1_0.431	2	2	2	2	2	2	2	2	2	1	1	1	1
3179	17	4,536,037	8,641,652	2	1	0_1_0_0_0_0_0_0_0_1_1_1_0.538	2	2	2	1	1	1	1	2	1	1	1	1	1
3180	17	8,641,653	8,651,240	1	0													
3181	17	8,651,241	8,915,290	2	1	0_1_0_0_0_0_0_0_0_1_0_1_0.346	2	2	2	1	1	1	1	1	1	1	1	1	1
3182	17	8,915,291	8,987,645	1	0													
3183	17	8,987,646	9,130,950	2	1	0_1_0_0_0_0_0_0_0_1_1_1_0.340	2	2	2	1	1	1	1	2	1	1	1	1	1
3184	17	9,130,951	9,144,300	1	0													
3185	17	9,144,301	10,966,734	2	1	0_1_0_0_0_0_0_0_0_1_0_1_0.168	2	2	2	1	1	1	1	1	1	1	1	1	1
3186	17	10,966,735	11,205,887	1	0													
3187	17	11,205,888	12,554,931	2	1	0_1_0_0_0_0_0_1_0_1_0_1_0.113	2	2	2	1	1	1	1	1	1	1	1	2	1
3188	17	12,554,932	12,556,913	1	0													
3189	17	12,556,914	13,444,612	2	1	0_1_0_0_0_0_0_1_0_1_1_1_0.136	2	2	2	1	1	1	1	2	1	1	1	2	1
3190	17	13,444,613	13,448,027	1	0													
3191	17	13,448,028	14,420,162	2	1	0_1_0_0_0_0_0_0_0_1_1_1_0.103	2	2	2	1	1	1	1	2	1	1	1	1	1
3192	17	14,420,163	14,727,191	1	0													
3193	17	14,727,192	14,874,918	2	1	0_0_0_0_0_0_0_0_0_0_1_0_0.185	1	1	1	1	1	1	1	2	1	1	1	1	1
3194	17	14,874,919	15,274,808	1	0													
3195	17	15,274,809	16,743,315	2	1	0_0_0_0_0_0_0_0_0_0_1_0_0.138	1	1	1	1	1	1	1	2	1	1	1	1	1
3196	17	16,743,316	16,915,928	1	0													
3197	17	16,915,929	17,396,320	2	1	0_0_0_0_0_0_0_0_0_0_1_0_0.103	1	1	1	1	1	1	1	2	1	1	1	1	1
3198	17	17,396,321	17,402,241	3	2	0_0_0_0_0_0_0_0_0_0_1_0_0.103	1	1	1	1	1	1	1	3	1	1	1	2	1
3199	17	17,402,242	17,525,719	3	3	0_0_0_0_0_0_0_0_0_0_1_0_0.103	1	1	1	1	1	1	1	3	1	1	1	2	1
3200	17	17,525,720	17,613,705	3	2	0_0_0_0_0_0_0_0_0_0_1_0_0.103	1	1	1	1	1	1	1	3	1	1	1	2	1
3201	17	17,613,706	17,810,749	2	1	0_0_0_0_0_0_0_0_0_0_1_0_0.103	1	1	1	1	1	1	1	2	1	1	1	1	1
3202	17	17,810,750	18,272,036	1	0													
3203	17	18,272,037	18,341,804	2	1	1_1_1_1_1_0_1_1_0_1_1_1_1.59	2	2	2	2	2	2	2	2	2	2	1	2	1
3204	17	18,341,805	18,342,993	1	0													
3205	17	18,342,994	18,655,155	2	1	0_0_0_0_0_0_0_0_0_0_1_0_0.269	1	1	1	1	1	1	1	2	1	1	1	1	1
3206	17	18,655,156	18,731,658	1	0													
3207	17	18,731,659	18,773,650	2	1	0_0_0_0_0_0_0_0_0_0_1_0_0.22	1	1	1	1	1	1	1	2	1	1	1	1	1
3208	17	18,773,651	18,951,382	1	0													
3209	17	18,951,383	19,127,975	2	1	0_0_0_0_0_0_0_0_0_0_1_0_0.208	1	1	1	1	1	1	1	2	1	1	1	1	1
3210	17	19,127,976	21,916,214	1	0													
3211	17	21,916,215	22,698,441	2	1	0_0_0_0_0_0_0_1_0_0_0_0_1.710	1	1	1	1	1	1	1	1	1	2	1	2	1
3212	17	22,698,442	25,958,462	1	0													
3213	17	25,958,463	26,190,629	2	1	1_1_1_1_1_0_1_0_0_1_1_1_0.392	2	2	2	2	2	2	2	2	2	1	1	1	1
3214	17	26,190,630	26,585,305	1	0													
3215	17	26,585,306	28,491,385	2	1	1_1_1_1_1_0_1_0_0_1_1_1_0.209	2	2	2	2	2	2	2	2	2	1	1	1	1
3216	17	28,491,386	28,626,539	1	0													
3217	17	28,626,540	28,788,651	2	1	1_1_1_1_1_0_1_0_0_1_1_1_0.294	2	2	2	2	2	2	2	2	2	1	1	1	1
3218	17	28,788,652	28,859,526	1	0													
3219	17	28,859,527	30,399,937	2	1	1_1_1_1_1_0_1_0_0_1_1_1_0.158	2	2	2	2	2	2	2	2	2	1	1	1	1
3220	17	30,399,938	31,742,941	1	0													
3221	17	31,742,942	31,863,523	2	1	1_1_1_1_1_0_1_0_0_1_1_1_0.37	2	2	2	2	2	2	2	2	2	1	1	1	1
3222	17	31,863,524	37,031,938	1	0													
3223	17	37,031,939	46,511,490	2	1	1_1_1_1_1_0_1_0_0_1_1_1_0.607	2	2	2	2	2	2	2	2	2	1	1	1	1

3224	17	46,511,491	52,663,163	1	0														
3225	17	52,663,164	55,309,838	2	1	1_1_1_1_1_0_1_0_0_1_1_1_0.212	2	2	2	2	2	2	2	2	2	1	1	1	1
3226	17	55,309,839	55,311,871	1	0														
3227	17	55,311,872	55,739,908	2	1	1_1_1_1_1_0_0_0_0_1_1_1_0.723	2	2	2	2	2	2	2	2	1	1	1	1	1
3228	17	55,739,909	55,777,533	1	0														
3229	17	55,777,534	55,936,180	2	1	1_1_1_1_1_0_1_0_0_1_1_1_0.224	2	2	2	2	2	2	2	2	2	1	1	1	1
3230	17	55,936,181	55,947,835	1	0														
3231	17	55,947,836	57,423,506	2	1	1_1_1_1_1_0_0_0_0_1_1_1_0.150	2	2	2	2	2	2	2	2	1	1	1	1	1
3232	17	57,423,507	57,596,600	1	0														
3233	17	57,596,601	58,262,183	2	1	0_0_0_0_0_0_0_1_0_0_0_0_1.253	1	1	1	1	1	1	1	1	1	2	1	2	1
3234	17	58,262,184	58,414,822	1	0														
3235	17	58,414,823	58,555,580	2	1	1_1_1_1_1_1_1_0_1_1_1_1_1.61	2	2	2	2	2	2	2	2	2	2	2	1	2
3236	17	58,555,581	58,645,036	1	0														
3237	17	58,645,037	59,629,248	2	1	1_1_1_1_1_0_0_1_0_1_1_1_0.994	2	2	2	2	2	2	2	2	1	1	1	2	1
3238	17	59,629,249	59,778,848	1	0														
3239	17	59,778,849	62,021,599	2	1	1_1_1_1_1_0_0_0_0_1_1_1_0.238	2	2	2	2	2	2	2	2	1	1	1	1	1
3240	17	62,021,600	63,071,137	1	0														
3241	17	63,071,138	64,251,912	2	1	0_0_0_0_0_0_0_1_0_0_0_0_1.619	1	1	1	1	1	1	1	1	1	2	1	2	1
3242	17	64,251,913	64,297,093	1	0														
3243	17	64,297,094	64,583,805	2	1	0_0_0_0_0_0_0_1_0_0_0_0_1.259	1	1	1	1	1	1	1	1	1	2	1	2	1
3244	17	64,583,806	64,605,049	1	0														
3245	17	64,605,050	64,732,112	2	1	1_1_1_1_1_0_0_1_0_1_1_1_1.55	2	2	2	2	2	2	2	2	1	2	1	2	1
3246	17	64,732,113	64,741,311	1	0														
3247	17	64,741,312	65,637,168	2	1	1_1_1_1_1_0_0_0_0_1_1_1_1.710	2	2	2	2	2	2	2	2	1	2	1	1	1
3248	17	65,637,169	65,638,591	1	0														
3249	17	65,638,592	65,721,705	2	1	0_0_0_0_0_0_0_0_0_0_0_0_1.103	1	1	1	1	1	1	1	1	1	2	1	1	1
3250	17	65,721,706	66,823,872	1	0														
3251	17	66,823,873	68,112,643	2	1	1_1_1_1_1_0_0_1_0_1_1_1_0.140	2	2	2	2	2	2	2	2	1	1	1	2	1
3252	17	68,112,644	68,376,360	1	0														
3253	17	68,376,361	68,616,683	2	1	1_1_1_1_1_0_0_1_0_1_1_1_0.383	2	2	2	2	2	2	2	2	1	1	1	2	1
3254	17	68,616,684	70,053,709	1	0														
3255	17	70,053,710	70,129,795	2	1	1_1_1_1_1_1_1_0_1_1_1_1_1.36	2	2	2	2	2	2	2	2	2	2	2	1	2
3256	17	70,129,796	73,354,018	1	0														
3257	17	73,354,019	73,566,697	2	1	1_1_1_1_1_1_1_0_1_1_1_1_1.74	2	2	2	2	2	2	2	2	2	2	2	1	2
3258	17	73,566,698	73,567,926	1	0														
3259	17	73,567,927	73,777,132	2	1	1_1_1_1_1_0_0_0_0_1_0_1_0.493	2	2	2	2	2	2	2	1	1	1	1	1	1
3260	17	73,777,133	73,938,688	3	2	1_1_1_1_1_0_0_0_0_1_0_1_0.493	3	3	3	3	3	3	3	1	1	1	1	2	1
3261	17	73,938,689	73,998,945	3	3	1_1_1_1_1_0_0_0_0_1_0_1_0.493	3	3	3	3	3	3	3	1	1	1	1	2	1
3262	17	73,998,946	74,040,718	3	2	1_1_1_1_1_0_0_0_0_1_0_1_0.493	3	3	3	3	3	3	3	1	1	1	1	2	1
3263	17	74,040,719	74,155,395	2	1	1_1_1_1_1_0_0_0_0_1_0_1_0.493	2	2	2	2	2	2	2	1	1	1	1	1	1
3264	17	74,155,396	74,191,616	3	2	1_1_1_1_1_0_0_0_0_1_0_1_0.493	3	3	3	3	3	3	3	1	1	1	1	2	1
3265	17	74,191,617	74,326,318	2	1	1_1_1_1_1_0_0_1_0_1_0_1_0.244	2	2	2	2	2	2	2	1	1	1	1	2	1
3266	17	74,326,319	74,328,525	1	0														
3267	17	74,328,526	74,656,626	2	1	1_1_1_1_1_1_1_0_1_1_1_1_1.168	2	2	2	2	2	2	2	2	2	2	2	1	2
3268	17	74,656,627	74,715,232	1	0														
3269	17	74,715,233	75,653,017	2	1	1_1_1_1_1_1_1_0_1_1_1_1_1.347	2	2	2	2	2	2	2	2	2	2	2	1	2
3270	17	75,653,018	76,667,418	1	0														
3271	17	76,667,419	76,791,129	2	1	0_0_0_0_0_0_0_0_0_0_1_0_0.396	1	1	1	1	1	1	1	2	1	1	1	1	1
3272	17	76,791,130	76,801,070	3	2	0_0_0_0_0_0_0_0_0_0_0_0_1.44 (	1	1	1	1	1	1	1	2	1	3	1	1	1
3273	17	76,801,071	76,835,449	3	3	0_0_0_0_0_0_0_0_0_0_0_0_1.44 (	1	1	1	1	1	1	1	2	1	3	1	1	1
3274	17	76,835,450	76,838,326	3	2	0_0_0_0_0_0_0_0_0_0_1_0_0.396	1	1	1	1	1	1	1	3	1	2	1	1	1
3275	17	76,838,327	76,839,172	2	1	0_0_0_0_0_0_0_0_0_0_1_0_0.396	1	1	1	1	1	1	1	2	1	1	1	1	1
3276	17	76,839,173	76,858,653	3	2	0_0_0_0_0_0_0_0_0_0_1_0_0.396	2	2	2	2	2	2	2	3	1	1	1	1	1
3277	17	76,858,654	77,123,555	3	3	0_0_0_0_0_0_0_0_0_0_1_0_0.396	2	2	2	2	2	2	2	3	1	1	1	1	1
3278	17	77,123,556	77,124,567	3	2	0_0_0_0_0_0_0_0_0_0_1_0_0.396	2	2	2	2	2	2	2	3	1	1	1	1	1
3279	17	77,124,568	77,124,849	2	1	0_0_0_0_0_0_0_0_0_0_1_0_0.396	1	1	1	1	1	1	1	2	1	1	1	1	1
3280	17	77,124,850	77,161,201	3	2	0_0_0_0_0_0_0_0_0_0_1_0_0.396	1	1	1	1	1	1	1	3	1	2	1	1	1
3281	17	77,161,202	77,243,308	3	3	0_0_0_0_0_0_0_0_0_0_0_0_1.126	1	1	1	1	1	1	1	2	1	3	1	1	1
3282	17	77,243,309	77,251,582	3	2	0_0_0_0_0_0_0_0_0_0_0_0_1.126	1	1	1	1	1	1	1	2	1	3	1	1	1
3283	17	77,251,583	77,283,560	2	1	0_0_0_0_0_0_0_0_0_0_0_0_1.126	1	1	1	1	1	1	1	1	1	2	1	1	1
3284	17	77,283,561	77,285,807	3	2	0_0_0_0_0_0_0_0_0_0_0_0_1.126	1	1	1	1	1	1	1	2	1	3	1	1	1
3285	17	77,285,808	77,395,259	3	3	0_0_0_0_0_0_0_0_0_0_0_0_1.126	1	1	1	1	1	1	1	2	1	3	1	1	1
3286	17	77,395,260	77,430,856	3	2	0_0_0_0_0_0_0_0_0_0_1_0_0.179	1	1	1	1	1	1	1	3	1	2	1	1	1
3287	17	77,430,857	77,581,209	3	3	0_0_0_0_0_0_0_0_0_0_0_0_1.166	1	1	1	1	1	1	1	2	1	3	1	1	1
3288	17	77,581,210	77,667,722	3	2	0_0_0_0_0_0_0_0_0_0_0_0_1.166	1	1	1	1	1	1	1	2	1	3	1	1	1
3289	17	77,667,723	78,822,819	2	1	0_0_0_0_0_0_0_0_0_0_1_0_1.156	1	1	1	1	1	1	1	2	1	2	1	1	1
3290	17	78,822,820	78,823,331	1	0														
3291	17	78,823,332	79,411,941	2	1	1_1_1_1_1_0_0_0_0_1_1_1_1.653	2	2	2	2	2	2	2	2	1	2	1	1	1
3292	17	79,411,942	81,307,425	1	0														
3293	17	81,307,426	81,352,293	2	1	1_1_1_1_1_0_0_0_0_1_1_1_1.20	2	2	2	2	2	2	2	2	1	2	1	1	1
3294	17	81,352,294	82,353,945	1	0														
3295	17	82,353,946	84,209,252	2	1	1_1_1_1_1_0_0_0_0_1_1_1_0.298	2	2	2	2	2	2	2	2	1	1	1	1	1
3296	17	84,209,253	84,332,230	1	0														
3297	17	84,332,231	85,313,808	2	1	1_1_1_1_1_0_0_0_0_1_1_1_0.749	2	2	2	2	2	2	2	2	1	1	1	1	1
3298	17	85,313,809	85,948,678	1	0														
3299	17	85,948,679	88,488,184	2	1	0_0_0_0_0_0_0_0_0_0_0_0_1.294	1	1	1	1	1	1	1	1	1	2	1	1	1
3300	17	88,488,185	88,772,272	1	0														
3301	17	88,772,273	90,843,096	2	1	0_0_0_0_0_0_0_0_0_0_0_0_1.186	1	1	1	1	1	1	1	1	1	2	1	1	1
3302	18	2,922,863	5,577,714	2	1	0_0_0_0_0_0_0_0_0_0_0_0_1.262	1	1	1	1	1	1	1	1	1	2	1	1	1
3303	18	5,577,715	5,799,067	1	0														
3304	18	5,799,068	5,976,444	2	1	0_0_0_0_0_0_0_0_0_0_0_0_1.213	1	1	1	1	1	1	1	1	1	2	1	1	1
3305	18	5,976,445	5,977,660	3	2	0_0_0_0_0_0_0_0_0_0_0_0_1.213	2	2	2	2	2	2	2	2	2	3	1	1	1
3306	18	5,977,661	6,113,328	3	3	0_0_0_0_0_0_0_0_0_0_0_0_1.213	2	2	2	2	2	2	2	2	2	3	1	1	1
3307	18	6,113,329	6,582,022	3	2	0_0_0_0_0_0_0_0_0_0_0_0_1.213	2	2	2	2	2	2	2	2	2	3	1	1	1
3308	18	6,582,023	8,107,272	2	1	0_0_0_0_0_0_0_0_0_0_0_0_1.213	1	1	1	1	1	1	1	1	1	2	1	1	1

Disease Models & Mechanisms • Supplementary information

3309	18	8,107,273	8,651,195	1	0														
3310	18	8,651,196	9,361,323	2	1	0_0_0_0_0_0_0_0_0_0_0_0_1.700	1	1	1	1	1	1	1	1	1	2	1	1	1
3311	18	9,361,324	10,503,652	1	0														
3312	18	10,503,653	12,170,096	2	1	0_0_0_0_0_0_0_0_0_0_0_0_1.212	1	1	1	1	1	1	1	1	1	2	1	1	1
3313	18	12,170,097	12,189,151	3	2	0_0_0_0_0_0_0_0_0_0_0_0_1.212	2	2	2	2	2	2	2	2	2	3	1	1	1
3314	18	12,189,152	12,304,992	3	3	0_0_0_0_0_0_0_0_0_0_0_0_1.212	2	2	2	2	2	2	2	2	2	3	1	1	1
3315	18	12,304,993	12,321,415	3	2	1_1_1_1_1_0_1_0_0_1_1_1_0.153	3	3	3	3	3	3	3	3	3	2	1	1	1
3316	18	12,321,416	13,293,324	2	1	1_1_1_1_1_0_1_0_0_1_1_1_0.153	2	2	2	2	2	2	2	2	2	1	1	1	1
3317	18	13,293,325	13,293,969	1	0														
3318	18	13,293,970	14,909,473	2	1	1_1_1_1_1_0_1_0_0_1_0_1_0.187	2	2	2	2	2	2	2	1	2	1	1	1	1
3319	18	14,909,474	14,977,218	1	0														
3320	18	14,977,219	14,981,025	2	1	1_1_1_1_1_0_1_0_0_1_1_1_0.120	2	2	2	2	2	2	2	2	2	1	1	1	1
3321	18	14,981,026	15,116,589	3	2	0_0_0_0_0_0_0_0_0_0_0_0_1.819	2	2	2	2	2	2	2	2	2	3	1	1	1
3322	18	15,116,590	15,723,032	2	1	0_0_0_0_0_0_0_0_0_0_0_0_1.819	1	1	1	1	1	1	1	1	1	2	1	1	1
3323	18	15,723,033	15,765,921	1	0														
3324	18	15,765,922	17,866,923	2	1	1_1_1_1_1_0_1_0_0_1_0_1_0.224	2	2	2	2	2	2	2	1	2	1	1	1	1
3325	18	17,866,924	17,880,824	1	0														
3326	18	17,880,825	18,061,638	2	1	0_0_0_0_0_0_0_0_0_0_0_0_1.839	1	1	1	1	1	1	1	1	1	2	1	1	1
3327	18	18,061,639	18,625,034	3	2	0_0_0_0_0_0_0_0_0_0_0_0_1.839	2	2	2	2	2	2	2	1	2	3	1	1	1
3328	18	18,625,035	19,525,709	3	3	0_0_0_0_0_0_0_0_0_0_0_0_1.839	2	2	2	2	2	2	2	1	2	3	1	1	1
3329	18	19,525,710	19,574,654	3	2	1_1_1_1_1_0_1_0_0_1_0_1_0.874	3	3	3	3	3	3	3	1	3	2	1	1	1
3330	18	19,574,655	19,599,354	2	1	1_1_1_1_1_0_1_0_0_1_0_1_0.874	2	2	2	2	2	2	2	1	2	1	1	1	1
3331	18	19,599,355	19,600,939	3	2	0_0_0_0_0_0_0_0_0_0_1_0_0.273	2	2	2	2	2	2	2	3	2	1	1	1	1
3332	18	19,600,940	20,075,949	3	3	0_0_0_0_0_0_0_0_0_0_1_0_0.273	2	2	2	2	2	2	2	3	2	1	1	1	1
3333	18	20,075,950	20,076,073	3	2	1_1_1_1_1_0_1_0_0_1_0_1_0.874	3	3	3	3	3	3	3	2	3	1	1	1	1
3334	18	20,076,074	20,165,327	2	1	1_1_1_1_1_0_1_0_0_1_1_1_0.392	2	2	2	2	2	2	2	2	2	1	1	1	1
3335	18	20,165,328	20,165,878	3	2	1_1_1_1_1_0_1_0_0_1_0_1_0.826	3	3	3	3	3	3	3	2	3	1	1	1	1
3336	18	20,165,879	20,228,029	3	3	0_0_0_0_0_0_0_0_0_0_1_0_0.43	2	2	2	2	2	2	2	3	2	1	1	1	1
3337	18	20,228,030	20,228,236	3	2	1_1_1_1_1_0_1_0_0_1_0_1_0.826	3	3	3	3	3	3	3	2	3	1	1	1	1
3338	18	20,228,237	27,801,378	2	1	1_1_1_1_1_0_1_0_0_1_0_1_0.826	2	2	2	2	2	2	2	1	2	1	1	1	1
3339	18	27,801,379	27,805,189	1	0														
3340	18	27,805,190	31,720,079	2	1	1_1_1_1_1_0_1_0_0_1_0_1_1.157	2	2	2	2	2	2	2	1	2	2	1	1	1
3341	18	31,720,080	31,722,049	1	0														
3342	18	31,722,050	32,425,421	2	1	1_1_1_1_1_0_1_0_0_1_0_1_0.626	2	2	2	2	2	2	2	1	2	1	1	1	1
3343	18	32,425,422	32,426,390	1	0														
3344	18	32,426,391	32,632,981	2	1	1_1_1_1_1_0_1_0_0_1_0_1_1.247	2	2	2	2	2	2	2	1	2	2	1	1	1
3345	18	32,632,982	32,634,484	1	0														
3346	18	32,634,485	36,010,751	2	1	1_1_1_1_1_0_1_0_0_1_0_1_0.157	2	2	2	2	2	2	2	1	2	1	1	1	1
3347	18	36,010,752	40,784,397	3	2	1_1_1_1_1_0_1_0_0_1_0_1_0.157	3	3	3	3	3	3	3	1	3	2	1	1	1
3348	18	40,784,398	43,248,732	2	1	1_1_1_1_1_0_1_0_0_1_0_1_1.204	2	2	2	2	2	2	2	1	2	2	1	1	1
3349	18	43,248,733	43,254,277	3	2	0_0_0_0_0_0_0_0_0_0_0_0_1.221	2	2	2	2	2	2	2	1	2	3	1	1	1
3350	18	43,254,278	43,660,054	3	3	0_0_0_0_0_0_0_0_0_0_0_0_1.221	2	2	2	2	2	2	2	1	2	3	1	1	1
3351	18	43,660,055	43,670,806	3	2	0_0_0_0_0_0_0_0_0_0_0_0_1.221	2	2	2	2	2	2	2	1	2	3	1	1	1
3352	18	43,670,807	43,798,850	2	1	1_1_1_1_1_0_1_0_0_1_0_1_1.204	2	2	2	2	2	2	2	1	2	2	1	1	1
3353	18	43,798,851	43,877,267	3	2	0_0_0_0_0_0_0_0_0_0_0_0_1.150	2	2	2	2	2	2	2	1	2	3	1	1	1
3354	18	43,877,268	44,010,346	3	3	0_0_0_0_0_0_0_0_0_0_0_0_1.150	2	2	2	2	2	2	2	1	2	3	1	1	1
3355	18	44,010,347	44,011,041	3	2	0_0_0_0_0_0_0_0_0_0_0_0_1.150	2	2	2	2	2	2	2	1	2	3	1	1	1
3356	18	44,011,042	44,017,868	2	1	0_0_0_0_0_0_0_0_0_0_0_0_1.150	1	1	1	1	1	1	1	1	1	2	1	1	1
3357	18	44,017,869	44,202,312	1	0														
3358	18	44,202,313	44,449,062	2	1	1_1_1_1_1_0_1_0_0_1_0_1_1.137	2	2	2	2	2	2	2	1	2	2	1	1	1
3359	18	44,449,063	44,464,802	3	2	0_0_0_0_0_0_0_0_0_0_0_0_1.67	2	2	2	2	2	2	2	1	2	3	1	1	1
3360	18	44,464,803	44,785,643	3	3	0_0_0_0_0_0_0_0_0_0_0_0_1.67	2	2	2	2	2	2	2	1	2	3	1	1	1
3361	18	44,785,644	44,785,644	3	2	0_0_0_0_0_0_0_0_0_0_0_0_1.67	2	2	2	2	2	2	2	1	2	3	1	1	1
3362	18	44,785,645	46,252,178	2	1	1_1_1_1_1_0_1_0_0_1_0_1_0.255	2	2	2	2	2	2	2	1	2	1	1	1	1
3363	18	46,252,179	46,313,126	3	2	0_0_0_0_0_0_0_0_0_0_0_0_1.182	2	2	2	2	2	2	2	1	2	3	1	1	1
3364	18	46,313,127	47,352,135	2	1	0_0_0_0_0_0_0_0_0_0_0_0_1.182	1	1	1	1	1	1	1	1	1	2	1	1	1
3365	18	47,352,136	47,445,340	1	0														
3366	18	47,445,341	47,818,223	2	1	1_1_1_1_1_0_1_0_0_1_0_1_1.366	2	2	2	2	2	2	2	1	2	2	1	1	1
3367	18	47,818,224	47,819,923	1	0														
3368	18	47,819,924	48,404,777	2	1	1_1_1_1_1_0_1_0_0_1_0_1_0.704	2	2	2	2	2	2	2	1	2	1	1	1	1
3369	18	48,404,778	50,184,206	1	0														
3370	18	50,184,207	52,567,506	2	1	1_1_1_1_1_0_1_0_0_1_0_1_1.253	2	2	2	2	2	2	2	1	2	2	1	1	1
3371	18	52,567,507	52,610,006	1	0														
3372	18	52,610,007	53,269,103	2	1	1_1_1_1_1_0_1_0_0_1_1_1_1.146	2	2	2	2	2	2	2	2	2	2	1	1	1
3373	18	53,269,104	53,270,387	3	2	0_0_0_0_0_0_0_0_0_0_0_0_1.204	2	2	2	2	2	2	2	2	2	3	1	1	1
3374	18	53,270,388	53,462,875	3	3	0_0_0_0_0_0_0_0_0_0_0_0_1.204	2	2	2	2	2	2	2	2	2	3	1	1	1
3375	18	53,462,876	53,463,419	3	2	1_1_1_1_1_0_1_0_0_1_1_1_0.81	3	3	3	3	3	3	3	3	3	2	1	1	1
3376	18	53,463,420	53,835,804	2	1	1_1_1_1_1_0_1_0_0_1_1_1_1.146	2	2	2	2	2	2	2	2	2	2	1	1	1
3377	18	53,835,805	53,837,004	3	2	0_0_0_0_0_0_0_0_0_0_0_0_1.151	2	2	2	2	2	2	2	2	2	3	1	1	1
3378	18	53,837,005	54,021,605	3	3	0_0_0_0_0_0_0_0_0_0_0_0_1.151	2	2	2	2	2	2	2	2	2	3	1	1	1
3379	18	54,021,606	54,042,382	3	2	0_0_0_0_0_0_0_0_0_0_0_0_1.151	2	2	2	2	2	2	2	2	2	3	1	1	1
3380	18	54,042,383	54,149,109	3	3	0_0_0_0_0_0_0_0_0_0_0_0_1.151	2	2	2	2	2	2	2	2	2	3	1	1	1
3381	18	54,149,110	54,162,536	3	2	0_0_0_0_0_0_0_0_0_0_0_0_1.151	2	2	2	2	2	2	2	2	2	3	1	1	1
3382	18	54,162,537	54,163,096	2	1	0_0_0_0_0_0_0_0_0_0_0_0_1.151	1	1	1	1	1	1	1	1	1	2	1	1	1
3383	18	54,163,097	54,171,686	3	2	0_0_0_0_0_0_0_0_0_0_0_0_1.151	2	2	2	2	2	2	2	2	1	3	1	1	1
3384	18	54,171,687	54,742,630	3	3	0_0_0_0_0_0_0_0_0_0_0_0_1.151	2	2	2	2	2	2	2	2	1	3	1	1	1
3385	18	54,742,631	54,865,656	3	2	0_0_0_0_0_0_0_0_0_0_0_0_1.151	2	2	2	2	2	2	2	2	1	3	1	1	1
3386	18	54,865,657	55,200,838	3	3	0_0_0_0_0_0_0_0_0_0_0_0_1.151	2	2	2	2	2	2	2	2	1	3	1	1	1
3387	18	55,200,839	55,214,546	3	2	0_0_0_0_0_0_0_0_0_0_0_0_1.151	2	2	2	2	2	2	2	2	1	3	1	1	1
3388	18	55,214,547	55,357,124	2	1	0_0_0_0_0_0_0_0_0_0_0_0_1.151	1	1	1	1	1	1	1	1	1	2	1	1	1
3389	18	55,357,125	55,379,618	3	2	0_0_0_0_0_0_0_0_0_0_0_0_1.151	2	2	2	2	2	2	2	2	1	3	1	1	1
3390	18	55,379,619	55,496,962	2	1	1_1_1_1_1_0_0_0_0_1_1_1_0.115	2	2	2	2	2	2	2	2	1	1	1	1	1
3391	18	55,496,963	55,593,173	1	0														
3392	18	55,593,174	56,999,692	2	1	1_1_1_1_1_1_1_0_1_1_1_1_1.256	2	2	2	2	2	2	2	2	2	2	2	1	2
3393	18	56,999,693	57,003,317	3	2	0_0_0_0_0_0_0_1_0_0_1_0_0.165	1	1	1	1	1	1	1	3	1	1	1	2	1

3394	18	57,003,318	57,235,705	3	3	0_0_0_0_0_0_0_0_0_1_0_0.287	1	1	1	1	1	1	1	3	1	1	1	2	1
3395	18	57,235,706	57,237,641	3	2	0_0_0_0_0_0_1_0_0_1_0_0.165	1	1	1	1	1	1	1	3	1	1	1	2	1
3396	18	57,237,642	57,250,558	2	1	1_1_1_1_1_1_1_0_1_1_1_1_1.256	2	2	2	2	2	2	2	2	2	2	2	1	2
3397	18	57,250,559	57,250,914	3	2	0_0_0_0_0_0_0_0_0_0_1_0_1.54	2	2	2	2	2	2	2	3	2	3	2	1	2
3398	18	57,250,915	57,326,322	3	3	0_0_0_0_0_0_0_0_0_0_1_0_1.54	2	2	2	2	2	2	2	3	2	3	2	1	2
3399	18	57,326,323	57,329,453	3	2	0_0_0_0_0_0_0_0_0_0_1_0_1.54	2	2	2	2	2	2	2	3	2	3	2	1	2
3400	18	57,329,454	57,461,701	2	1	1_1_1_1_1_1_1_0_1_1_1_1_1.256	2	2	2	2	2	2	2	2	2	2	2	1	2
3401	18	57,461,702	57,480,205	3	2	0_0_0_0_0_0_0_0_0_0_1_0_1.197	2	2	2	2	2	2	2	3	2	3	2	1	2
3402	18	57,480,206	57,779,042	3	3	0_0_0_0_0_0_0_0_0_0_1_0_1.197	2	2	2	2	2	2	2	3	2	3	2	1	2
3403	18	57,779,043	57,783,699	3	2	1_1_1_1_1_1_1_0_1_1_0_1_0.89	3	3	3	3	3	3	3	2	3	2	3	1	3
3404	18	57,783,700	57,862,406	2	1	1_1_1_1_1_1_1_0_1_1_1_1_1.256	2	2	2	2	2	2	2	2	2	2	2	1	2
3405	18	57,862,407	57,865,290	3	2	1_1_1_1_1_0_0_0_0_1_0_1_0.347	3	3	3	3	3	3	3	2	2	2	2	1	2
3406	18	57,865,291	58,232,839	3	3	1_1_1_1_1_0_0_0_0_1_0_1_0.347	3	3	3	3	3	3	3	1	1	1	1	2	1
3407	18	58,232,840	58,246,044	3	2	1_1_1_1_1_0_0_0_0_1_0_1_0.347	3	3	3	3	3	3	3	1	1	1	1	2	1
3408	18	58,246,045	58,250,711	2	1	1_1_1_1_1_0_0_0_0_1_0_1_0.347	2	2	2	2	2	2	2	1	1	1	1	1	1
3409	18	58,250,712	58,252,130	3	2	1_1_1_1_1_0_0_0_0_1_0_1_0.347	3	3	3	3	3	3	3	2	1	2	1	1	1
3410	18	58,252,131	58,729,605	3	3	0_0_0_0_0_0_0_0_0_0_1_0_1.426	2	2	2	2	2	2	2	3	1	3	1	1	1
3411	18	58,729,606	58,731,205	3	2	1_1_1_1_1_0_0_0_0_1_0_1_0.347	3	3	3	3	3	3	3	2	1	2	1	1	1
3412	18	58,731,206	58,738,457	2	1	1_1_1_1_1_0_0_0_0_1_1_1_1.333	2	2	2	2	2	2	2	2	1	2	1	1	1
3413	18	58,738,458	58,739,756	1	0														
3414	18	58,739,757	58,782,556	2	1	0_0_0_0_0_0_0_0_0_0_0_0_1.118	1	1	1	1	1	1	1	1	1	2	1	1	1
3415	18	58,782,557	58,792,513	1	0														
3416	18	58,792,514	58,795,910	2	1	1_1_1_1_1_0_0_0_0_1_1_1_1.149	2	2	2	2	2	2	2	2	1	2	1	1	1
3417	18	58,795,911	58,817,393	3	2	0_0_0_0_0_0_0_0_0_0_1_0_1.129	2	2	2	2	2	2	2	3	1	3	1	1	1
3418	18	58,817,394	58,912,475	3	3	0_0_0_0_0_0_0_0_0_0_1_0_1.129	2	2	2	2	2	2	2	3	1	3	1	1	1
3419	18	58,912,476	58,973,840	3	2	1_1_1_1_1_0_0_0_0_1_0_1_0.158	3	3	3	3	3	3	3	2	1	2	1	1	1
3420	18	58,973,841	59,026,910	2	1	1_1_1_1_1_0_0_0_0_1_1_1_1.149	2	2	2	2	2	2	2	2	1	2	1	1	1
3421	18	59,026,911	59,051,263	3	2	0_0_0_0_0_0_0_0_0_0_1_0_1.37	2	2	2	2	2	2	2	3	1	3	1	1	1
3422	18	59,051,264	59,263,770	3	3	0_0_0_0_0_0_0_0_0_0_1_0_1.37	2	2	2	2	2	2	2	3	1	3	1	1	1
3423	18	59,263,771	59,265,206	3	2	0_0_0_0_0_0_0_0_0_0_1_0_1.37	2	2	2	2	2	2	2	3	1	3	1	1	1
3424	18	59,265,207	59,758,935	2	1	1_1_1_1_1_0_0_0_0_1_1_1_1.149	2	2	2	2	2	2	2	2	1	2	1	1	1
3425	18	59,758,936	59,760,571	3	2	1_1_1_1_1_0_0_0_0_1_0_1_0.122	3	3	3	3	3	3	3	2	1	2	1	1	1
3426	18	59,760,572	60,118,222	3	3	0_0_0_0_0_0_0_0_0_0_1_0_1.537	2	2	2	2	2	2	2	3	1	3	1	1	1
3427	18	60,118,223	60,320,657	3	2	0_0_0_0_0_0_0_0_0_0_1_0_1.537	2	2	2	2	2	2	2	3	1	3	1	1	1
3428	18	60,320,658	60,349,016	2	1	0_0_0_0_0_0_0_0_0_0_1_0_1.537	1	1	1	1	1	1	1	2	1	2	1	1	1
3429	18	60,349,017	60,551,517	3	2	0_0_0_0_0_0_0_0_0_0_1_0_1.537	2	2	2	2	2	2	2	3	1	3	1	1	1
3430	18	60,551,518	60,737,669	3	3	0_0_0_0_0_0_0_0_0_0_1_0_1.537	2	2	2	2	2	2	2	3	1	3	1	1	1
3431	18	60,737,670	61,046,481	3	2	0_0_0_0_0_0_0_0_0_0_1_0_1.537	2	2	2	2	2	2	2	3	1	3	1	1	1
3432	18	61,046,482	61,047,937	2	1	1_1_1_1_1_0_0_0_0_1_0_1_0.433	2	2	2	2	2	2	2	1	1	1	1	1	1
3433	18	61,047,938	61,080,195	3	2	1_1_1_1_1_0_0_0_0_1_0_1_0.433	3	3	3	3	3	3	3	2	1	1	1	1	1
3434	18	61,080,196	61,177,765	2	1	1_1_1_1_1_0_0_0_0_1_1_1_0.135	2	2	2	2	2	2	2	2	1	1	1	1	1
3435	18	61,177,766	61,239,342	3	2	1_1_1_1_1_0_0_0_0_1_0_1_0.130	3	3	3	3	3	3	3	2	1	1	1	1	1
3436	18	61,239,343	61,309,578	2	1	1_1_1_1_1_0_0_0_0_1_0_1_0.130	2	2	2	2	2	2	2	1	1	1	1	1	1
3437	18	61,309,579	61,380,546	3	2	1_1_1_1_1_0_0_0_0_1_0_1_0.130	3	3	3	3	3	3	3	2	1	1	1	1	1
3438	18	61,380,547	61,872,175	3	3	0_0_0_0_0_0_0_0_0_0_1_0_0.407	2	2	2	2	2	2	2	3	1	1	1	1	1
3439	18	61,872,176	61,895,817	3	2	1_1_1_1_1_0_0_0_0_1_0_1_0.130	3	3	3	3	3	3	3	2	1	1	1	1	1
3440	18	61,895,818	61,908,307	2	1	1_1_1_1_1_0_0_0_0_1_0_1_0.130	2	2	2	2	2	2	2	1	1	1	1	1	1
3441	18	61,908,308	61,910,310	3	2	1_1_1_1_1_0_0_0_0_1_0_1_0.130	3	3	3	3	3	3	3	2	1	1	1	1	1
3442	18	61,910,311	62,292,636	3	3	0_0_0_0_0_0_0_0_0_0_1_0_0.282	2	2	2	2	2	2	2	3	1	1	1	1	1
3443	18	62,292,637	62,347,106	3	2	1_1_1_1_1_0_0_0_0_1_0_1_0.130	3	3	3	3	3	3	3	2	1	1	1	1	1
3444	18	62,347,107	62,355,516	2	1	1_1_1_1_1_0_0_0_0_1_1_1_0.776	2	2	2	2	2	2	2	2	1	1	1	1	1
3445	18	62,355,517	62,380,002	3	2	0_0_0_0_0_0_0_0_0_0_1_0_0.244	2	2	2	2	2	2	2	3	1	1	1	1	1
3446	18	62,380,003	62,907,034	3	3	0_0_0_0_0_0_0_0_0_0_1_0_0.244	2	2	2	2	2	2	2	3	1	1	1	1	1
3447	18	62,907,035	62,909,178	3	2	0_0_0_0_0_0_0_0_0_0_1_0_0.244	2	2	2	2	2	2	2	3	1	1	1	1	1
3448	18	62,909,179	62,909,660	2	1	0_0_0_0_0_0_0_0_0_0_1_0_0.244	1	1	1	1	1	1	1	2	1	1	1	1	1
3449	18	62,909,661	62,915,960	3	2	0_0_0_0_0_0_0_0_0_0_1_0_0.244	2	2	2	2	2	2	2	3	2	1	1	1	1
3450	18	62,915,961	63,104,388	2	1	1_1_1_1_1_0_1_0_0_1_1_1_0.366	2	2	2	2	2	2	2	2	2	1	1	1	1
3451	18	63,104,389	63,105,035	3	2	1_1_1_1_1_0_1_0_0_1_0_1_0.422	3	3	3	3	3	3	3	2	3	1	1	1	1
3452	18	63,105,036	63,240,465	3	3	0_0_0_0_0_0_0_0_0_0_1_0_0.120	2	2	2	2	2	2	2	3	2	1	1	1	1
3453	18	63,240,466	63,360,814	3	2	1_1_1_1_1_0_1_0_0_1_0_1_0.422	3	3	3	3	3	3	3	2	3	1	1	1	1
3454	18	63,360,815	63,570,720	2	1	1_1_1_1_1_0_1_0_0_1_0_1_0.422	2	2	2	2	2	2	2	1	2	1	1	1	1
3455	18	63,570,721	63,573,762	3	2	0_0_0_0_0_0_0_0_0_0_1_0_0.64	2	2	2	2	2	2	2	3	2	1	1	1	1
3456	18	63,573,763	63,686,358	3	3	0_0_0_0_0_0_0_0_0_0_1_0_0.64	2	2	2	2	2	2	2	3	2	1	1	1	1
3457	18	63,686,359	63,721,516	3	2	1_1_1_1_1_0_1_0_0_1_0_1_0.422	3	3	3	3	3	3	3	2	3	1	1	1	1
3458	18	63,721,517	63,724,896	2	1	1_1_1_1_1_0_1_0_0_1_1_1_0.102	2	2	2	2	2	2	2	2	2	1	1	1	1
3459	18	63,724,897	63,733,684	3	2	0_1_0_0_0_0_0_0_0_1_0_1_0.799	3	3	3	2	2	2	2	2	2	1	1	1	1
3460	18	63,733,685	65,057,225	3	3	0_1_0_0_0_0_0_0_0_1_0_1_0.799	3	3	3	2	2	2	2	2	2	1	1	1	1
3461	18	65,057,226	65,095,059	3	2	0_1_0_0_0_0_0_0_0_1_0_1_0.799	3	3	3	2	2	2	2	2	2	1	1	1	1
3462	18	65,095,060	65,139,400	2	1	1_1_1_1_1_0_1_0_0_1_1_1_0.102	2	2	2	2	2	2	2	2	2	1	1	1	1
3463	18	65,139,401	65,142,431	3	2	1_0_1_1_1_0_1_0_0_0_1_0_0.609	2	2	2	3	3	3	3	3	3	1	1	1	1
3464	18	65,142,432	65,154,816	3	3	0_1_0_0_0_0_0_0_0_1_0_1_0.24	3	3	3	2	2	2	2	2	2	1	1	1	1
3465	18	65,154,817	65,156,605	3	2	1_0_1_1_1_0_1_0_0_0_1_0_0.609	2	2	2	3	3	3	3	3	3	1	1	1	1
3466	18	65,156,606	65,589,050	2	1	1_0_1_1_1_0_1_0_0_0_1_0_0.609	1	1	1	2	2	2	2	2	2	1	1	1	1
3467	18	65,589,051	65,589,562	3	2	0_1_0_0_0_0_0_0_0_1_0_1_0.103	3	3	3	2	2	2	2	2	2	1	1	1	1
3468	18	65,589,563	65,680,272	3	3	0_1_0_0_0_0_0_0_0_1_0_1_0.103	3	3	3	2	2	2	2	2	2	1	1	1	1
3469	18	65,680,273	65,703,074	3	2	0_1_0_0_0_0_0_0_0_1_0_1_0.103	3	3	3	2	2	2	2	2	2	1	1	1	1
3470	18	65,703,075	66,133,781	2	1	1_1_1_1_1_0_1_0_0_1_1_1_0.232	2	2	2	2	2	2	2	2	2	1	1	1	1
3471	18	66,133,782	66,188,954	1	0														
3472	18	66,188,955	66,250,089	2	1	1_1_1_1_1_0_1_0_0_1_1_1_0.55	2	2	2	2	2	2	2	2	2	1	1	1	1
3473	18	66,250,090	66,256,259	1	0														
3474	18	66,256,260	66,564,830	2	1	0_1_0_0_0_0_0_0_0_1_0_1_0.764	2	2	2	1	1	1	1	1	1	1	1	1	1
3475	18	66,564,831	66,567,488	3	2	0_1_0_0_0_0_0_0_0_1_0_1_0.764	3	3	3	2	2	2	2	2	2	1	2	2	2
3476	18	66,567,489	66,927,304	3	3	0_0_0_0_0_0_0_0_0_0_0_0_1.820	2	2	2	1	1	1	1	1	1	3	1	1	1
3477	18	66,927,305	66,928,621	3	2	0_0_0_0_0_0_0_0_0_0_0_0_1.820	2	2	2	1	1	1	1	1	1	3	1	1	1
3478	18	66,928,622	67,383,001	2	1	0_0_0_0_0_0_0_0_0_0_0_0_1.820	1	1	1	1	1	1	1	1	1	2	1	1	1

3479	18	67,383,002	67,624,970	1	0														
3480	18	67,624,971	68,328,932	2	1	0_1_0_0_0_1_1_1_1_1_0_1_0.103	2	2	2	1	1	1	1	1	2	1	2	2	2
3481	18	68,328,933	68,337,278	1	0														
3482	18	68,337,279	68,338,064	2	1	1_1_1_1_1_0_1_0_0_1_1_1_0.363	2	2	2	2	2	2	2	2	2	1	1	1	1
3483	18	68,338,065	68,340,655	3	2	1_0_1_1_1_0_1_0_0_0_1_0_0.288	2	2	2	3	3	3	3	3	3	1	1	1	1
3484	18	68,340,656	68,792,593	3	3	0_1_0_0_0_0_0_0_0_1_0_1_0.581	3	3	3	2	2	2	2	2	2	1	1	1	1
3485	18	68,792,594	68,792,947	3	2	0_1_0_0_0_0_0_0_0_1_0_1_0.581	3	3	3	2	2	2	2	2	2	1	1	1	1
3486	18	68,792,948	68,827,290	2	1	0_1_0_0_0_0_0_0_0_1_0_1_0.581	2	2	2	1	1	1	1	1	1	1	1	1	1
3487	18	68,827,291	68,827,764	1	0														
3488	18	68,827,765	69,009,994	2	1	1_1_1_1_1_0_1_0_0_1_1_1_0.287	2	2	2	2	2	2	2	2	2	1	1	1	1
3489	18	69,009,995	69,011,932	1	0														
3490	18	69,011,933	69,048,468	2	1	0_1_0_0_0_1_1_1_1_1_0_1_0.27	2	2	2	1	1	1	1	1	2	1	2	2	2
3491	18	69,048,469	69,053,016	1	0														
3492	18	69,053,017	69,153,287	2	1	1_0_1_1_1_0_0_0_0_0_0_0_1.162	1	1	1	2	2	2	2	1	1	2	1	1	1
3493	18	69,153,288	69,155,317	1	0														
3494	18	69,155,318	69,258,974	2	1	0_1_0_0_0_1_1_1_1_1_1_1_1.220	2	2	2	1	1	1	1	2	2	2	2	2	2
3495	18	69,258,975	69,261,996	3	2	0_1_0_0_0_0_0_0_0_1_0_1_0.609	3	3	3	1	1	1	1	2	2	2	2	2	2
3496	18	69,261,997	69,749,701	3	3	0_1_0_0_0_0_0_0_0_1_0_1_0.609	3	3	3	1	1	1	1	2	2	2	2	2	2
3497	18	69,749,702	69,776,083	3	2	0_1_0_0_0_0_0_0_0_1_0_1_0.609	3	3	3	1	1	1	1	2	2	2	2	2	2
3498	18	69,776,084	69,889,675	2	1	0_1_0_0_0_0_0_0_0_1_0_1_0.609	2	2	2	1	1	1	1	1	1	1	1	1	1
3499	18	69,889,676	69,894,571	3	2	0_1_0_0_0_0_0_0_0_1_0_1_0.609	3	3	3	2	2	2	2	1	1	1	1	1	1
3500	18	69,894,572	69,994,983	3	3	0_1_0_0_0_0_0_0_0_1_0_1_0.609	3	3	3	1	1	1	1	2	2	2	2	2	2
3501	18	69,994,984	70,013,974	3	2	0_1_0_0_0_1_1_1_1_1_1_1_1.46	3	3	3	1	1	1	1	2	2	2	2	2	2
3502	18	70,013,975	70,037,781	2	1	1_1_1_1_1_0_0_0_0_1_0_1_0.315	2	2	2	2	2	2	2	1	1	1	1	1	1
3503	18	70,037,782	70,038,241	1	0														
3504	18	70,038,242	70,038,761	2	1	1_1_1_1_1_0_0_0_0_1_1_1_0.359	2	2	2	2	2	2	2	2	1	1	1	1	1
3505	18	70,038,762	70,040,948	3	2	1_0_1_1_1_0_0_0_0_0_1_0_0.523	2	2	2	3	3	3	3	3	1	1	1	1	1
3506	18	70,040,949	70,721,668	3	3	0_1_0_0_0_0_0_0_0_1_0_1_0.147	3	3	3	2	2	2	2	2	1	1	1	1	1
3507	18	70,721,669	70,725,302	3	2	0_1_0_0_0_0_0_0_0_1_0_1_0.147	3	3	3	2	2	2	2	2	1	1	1	1	1
3508	18	70,725,303	71,528,096	2	1	0_1_0_0_0_0_0_0_0_1_0_1_0.147	2	2	2	1	1	1	1	1	1	1	1	1	1
3509	18	71,528,097	71,702,974	3	2	0_1_0_0_0_0_0_0_0_1_0_1_0.147	3	3	3	2	2	2	2	2	1	1	1	1	1
3510	18	71,702,975	71,802,782	3	3	0_1_0_0_0_0_0_0_0_1_0_1_0.147	3	3	3	2	2	2	2	2	1	1	1	1	1
3511	18	71,802,783	71,805,553	3	2	0_1_0_0_0_0_0_0_0_1_0_1_0.147	3	3	3	2	2	2	2	2	1	1	1	1	1
3512	18	71,805,554	71,806,532	2	1	0_1_0_0_0_0_0_0_0_1_0_1_0.147	2	2	2	1	1	1	1	1	1	1	1	1	1
3513	18	71,806,533	71,816,020	3	2	0_1_0_0_0_0_0_0_0_1_0_1_0.147	3	3	3	1	1	1	1	1	2	1	2	2	2
3514	18	71,816,021	71,916,579	2	1	0_1_0_0_0_1_1_1_1_1_0_1_0.192	2	2	2	1	1	1	1	1	2	1	2	2	2
3515	18	71,916,580	71,949,243	3	2	0_1_0_0_0_0_0_0_0_1_0_1_0.203	3	3	3	1	1	1	1	1	2	1	2	2	2
3516	18	71,949,244	72,205,181	2	1	0_1_0_0_0_0_0_0_0_1_0_1_0.203	2	2	2	1	1	1	1	1	1	1	1	1	1
3517	18	72,205,182	72,277,881	1	0														
3518	18	72,277,882	72,630,747	2	1	0_0_0_0_0_0_0_0_0_0_0_0_1.205	1	1	1	1	1	1	1	1	1	2	1	1	1
3519	18	72,630,748	74,517,708	1	0														
3520	18	74,517,709	75,403,707	2	1	1_1_1_1_1_0_0_0_0_1_1_1_0.145	2	2	2	2	2	2	2	2	1	1	1	1	1
3521	18	75,403,708	75,406,722	1	0														
3522	18	75,406,723	77,270,422	2	1	1_1_1_1_1_0_1_0_0_1_1_1_0.172	2	2	2	2	2	2	2	2	2	1	1	1	1
3523	18	77,270,423	77,274,346	1	0														
3524	18	77,274,347	80,479,075	2	1	1_1_1_1_1_0_0_0_0_1_1_1_0.328	2	2	2	2	2	2	2	2	1	1	1	1	1
3525	18	80,479,076	84,638,461	1	0														
3526	18	84,638,462	87,649,603	2	1	1_1_1_1_1_0_0_0_0_1_1_1_0.311	2	2	2	2	2	2	2	2	1	1	1	1	1
3527	19	1,447,690	8,643,837	2	1	0_0_0_0_0_0_0_0_0_0_0_0_1.662	1	1	1	1	1	1	1	1	1	2	1	1	1
3528	19	8,643,838	9,130,031	1	0														
3529	19	9,130,032	9,527,092	2	1	1_1_1_1_1_0_1_0_0_1_1_1_0.485	2	2	2	2	2	2	2	2	2	1	1	1	1
3530	19	9,527,093	10,221,432	1	0														
3531	19	10,221,433	10,518,229	2	1	1_1_1_1_1_0_1_0_0_1_1_1_0.323	2	2	2	2	2	2	2	2	2	1	1	1	1
3532	19	10,518,230	13,669,182	1	0														
3533	19	13,669,183	16,438,516	2	1	1_1_1_1_1_0_1_0_0_1_1_1_0.228	2	2	2	2	2	2	2	2	2	1	1	1	1
3534	19	16,438,517	16,507,094	1	0														
3535	19	16,507,095	16,665,403	2	1	1_1_1_1_1_0_1_0_0_1_1_1_0.237	2	2	2	2	2	2	2	2	2	1	1	1	1
3536	19	16,665,404	18,895,046	1	0														
3537	19	18,895,047	21,518,732	2	1	1_1_1_1_1_0_0_0_0_1_1_1_1.654	2	2	2	2	2	2	2	2	1	2	1	1	1
3538	19	21,518,733	23,442,335	1	0														
3539	19	23,442,336	23,445,655	2	1	0_0_0_0_0_0_0_0_0_0_0_0_1.356	1	1	1	1	1	1	1	1	1	2	1	1	1
3540	19	23,445,656	23,939,118	3	2	0_0_0_0_0_0_0_0_0_0_0_0_1.356	2	2	2	2	2	2	2	2	1	3	1	1	1
3541	19	23,939,119	23,993,020	2	1	0_0_0_0_0_0_0_0_0_0_0_0_1.356	1	1	1	1	1	1	1	1	1	2	1	1	1
3542	19	23,993,021	24,123,657	1	0														
3543	19	24,123,658	25,386,280	2	1	1_1_1_1_1_0_0_0_0_1_1_1_1.470	2	2	2	2	2	2	2	2	1	2	1	1	1
3544	19	25,386,281	25,437,806	1	0														
3545	19	25,437,807	25,675,313	2	1	0_0_0_0_0_0_0_0_0_0_0_0_1.322	1	1	1	1	1	1	1	1	1	2	1	1	1
3546	19	25,675,314	25,695,426	3	2	0_0_0_0_0_0_0_0_0_0_0_0_1.322	2	2	2	2	2	2	2	2	1	3	1	1	1
3547	19	25,695,427	25,699,616	2	1	1_1_1_1_1_0_0_0_0_1_1_1_0.36	2	2	2	2	2	2	2	2	1	1	1	1	1
3548	19	25,699,617	29,487,517	1	0														
3549	19	29,487,518	29,487,695	2	1	1_1_1_1_1_0_0_0_0_1_1_1_0.191	2	2	2	2	2	2	2	2	1	1	1	1	1
3550	19	29,487,696	31,050,726	3	2	0_0_0_0_0_0_0_0_0_0_0_0_1.121	2	2	2	2	2	2	2	2	1	3	1	1	1
3551	19	31,050,727	31,290,823	3	3	0_0_0_0_0_0_0_0_0_0_0_0_1.121	2	2	2	2	2	2	2	2	1	3	1	1	1
3552	19	31,290,824	31,446,650	3	2	0_0_0_0_0_0_0_0_0_0_0_0_1.121	2	2	2	2	2	2	2	2	1	3	1	1	1
3553	19	31,446,651	31,998,370	2	1	1_1_1_1_1_0_0_0_0_1_1_1_0.191	2	2	2	2	2	2	2	2	1	1	1	1	1
3554	19	31,998,371	32,254,023	1	0														
3555	19	32,254,024	32,695,961	2	1	1_1_1_1_1_0_0_0_0_1_1_1_1.695	2	2	2	2	2	2	2	2	1	2	1	1	1
3556	19	32,695,962	34,212,550	1	0														
3557	19	34,212,551	34,251,554	2	1	1_1_1_1_1_0_0_0_0_1_1_1_1.22	2	2	2	2	2	2	2	2	1	2	1	1	1
3558	19	34,251,555	34,252,639	1	0														
3559	19	34,252,640	34,752,478	2	1	0_1_0_0_0_0_0_0_0_1_1_1_0.532	2	2	2	1	1	1	1	2	1	1	1	1	1
3560	19	34,752,479	34,755,500	1	0														
3561	19	34,755,501	35,126,888	2	1	0_1_0_0_0_0_0_0_0_1_1_1_1.246	2	2	2	1	1	1	1	2	1	2	1	1	1
3562	19	35,126,889	35,192,135	1	0														
3563	19	35,192,136	35,261,936	2	1	0_1_0_0_0_0_0_0_0_1_1_1_1.51	2	2	2	1	1	1	1	2	1	2	1	1	1

Disease Models & Mechanisms • Supplementary information

3564	19	35,261,937	35,426,949	1	0														
3565	19	35,426,950	35,717,064	2	1	0_1_0_0_0_0_0_0_0_1_1_1_1.134	2	2	2	1	1	1	1	2	1	2	1	1	1
3566	19	35,717,065	38,002,079	1	0														
3567	19	38,002,080	38,337,335	2	1	0_0_0_0_0_0_0_0_0_0_0_0_1.197	1	1	1	1	1	1	1	1	1	2	1	1	1
3568	19	38,337,336	38,339,093	1	0														
3569	19	38,339,094	38,493,662	2	1	0_1_0_0_0_0_0_0_0_1_1_1_0.144	2	2	2	1	1	1	1	2	1	1	1	1	1
3570	19	38,493,663	38,539,712	1	0														
3571	19	38,539,713	38,539,803	2	1	0_0_0_0_0_0_0_0_0_0_0_0_1.175	1	1	1	1	1	1	1	1	1	2	1	1	1
3572	19	38,539,804	38,730,474	3	2	0_0_0_0_0_0_0_0_0_0_0_0_1.175	1	1	1	2	2	2	2	1	2	3	1	1	1
3573	19	38,730,475	38,731,721	2	1	1_0_1_1_1_0_1_0_0_0_0_0_1.129	1	1	1	2	2	2	2	1	2	2	1	1	1
3574	19	38,731,722	39,151,509	1	0														
3575	19	39,151,510	39,151,734	2	1	1_1_1_1_1_0_1_0_0_1_1_1_0.475	2	2	2	2	2	2	2	2	2	1	1	1	1
3576	19	39,151,735	40,145,207	3	2	0_1_0_0_0_0_0_0_0_1_1_1_0.355	3	3	3	2	2	2	2	3	2	1	1	1	1
3577	19	40,145,208	40,284,153	2	1	1_1_1_1_1_0_1_0_0_1_1_1_0.475	2	2	2	2	2	2	2	2	2	1	1	1	1
3578	19	40,284,154	40,334,883	1	0														
3579	19	40,334,884	40,862,547	2	1	1_0_1_1_1_0_1_0_0_0_1_0_0.250	1	1	1	2	2	2	2	2	2	1	1	1	1
3580	19	40,862,548	40,871,731	3	2	0_1_0_0_0_0_0_0_0_1_0_1_0.119	3	3	3	2	2	2	2	2	2	1	1	1	1
3581	19	40,871,732	41,067,249	3	3	0_1_0_0_0_0_0_0_0_1_0_1_0.119	3	3	3	2	2	2	2	2	2	1	1	1	1
3582	19	41,067,250	41,108,578	3	2	1_0_1_1_1_0_1_0_0_0_1_0_0.250	2	2	2	3	3	3	3	3	3	1	1	1	1
3583	19	41,108,579	42,049,969	2	1	1_0_1_1_1_0_1_0_0_0_1_0_0.250	1	1	1	2	2	2	2	2	2	1	1	1	1
3584	19	42,049,970	42,094,669	3	2	1_0_1_1_1_0_1_0_0_0_1_0_0.250	2	2	2	3	3	3	3	3	3	1	1	1	1
3585	19	42,094,670	42,944,239	2	1	1_0_1_1_1_0_1_0_0_0_1_0_0.250	1	1	1	2	2	2	2	2	2	1	1	1	1
3586	19	42,944,240	42,965,994	1	0														
3587	19	42,965,995	43,255,292	2	1	1_0_1_1_1_0_0_0_0_0_1_0_0.97	1	1	1	2	2	2	2	2	1	1	1	1	1
3588	19	43,255,293	43,255,875	3	2	1_0_1_1_1_0_0_0_0_0_1_0_0.97	2	2	2	3	3	3	3	3	1	1	1	1	1
3589	19	43,255,876	43,315,295	3	3	0_1_0_0_0_0_0_0_0_1_0_1_0.53	3	3	3	2	2	2	2	2	1	1	1	1	1
3590	19	43,315,296	43,316,797	3	2	0_1_0_0_0_0_0_0_0_1_0_1_0.53	3	3	3	2	2	2	2	2	1	1	1	1	1
3591	19	43,316,798	43,320,769	2	1	0_1_0_0_0_0_0_0_0_1_0_1_0.53	2	2	2	1	1	1	1	1	1	1	1	1	1
3592	19	43,320,770	43,330,244	3	2	0_1_0_0_0_0_0_0_0_1_0_1_0.53	3	3	3	2	2	2	2	2	2	1	1	1	1
3593	19	43,330,245	43,461,777	2	1	1_0_1_1_1_0_1_0_0_0_1_0_0.178	1	1	1	2	2	2	2	2	2	1	1	1	1
3594	19	43,461,778	43,477,408	1	0														
3595	19	43,477,409	43,599,926	2	1	1_0_1_1_1_0_0_0_0_0_1_0_0.101	1	1	1	2	2	2	2	2	1	1	1	1	1
3596	19	43,599,927	43,658,660	3	2	1_0_1_1_1_0_0_0_0_0_1_0_0.101	2	2	2	3	3	3	3	3	1	1	1	1	1
3597	19	43,658,661	43,950,563	3	3	0_1_0_0_0_0_0_0_0_1_0_1_0.59	3	3	3	2	2	2	2	2	1	1	1	1	1
3598	19	43,950,564	44,067,125	3	2	0_1_0_0_0_0_0_0_0_1_0_1_0.59	3	3	3	2	2	2	2	2	1	1	1	1	1
3599	19	44,067,126	44,144,818	2	1	1_0_1_1_1_0_0_0_0_0_1_0_0.101	1	1	1	2	2	2	2	2	1	1	1	1	1
3600	19	44,144,819	44,269,743	3	2	0_1_0_0_0_0_0_0_0_1_0_1_0.26	3	3	3	2	2	2	2	2	1	1	1	1	1
3601	19	44,269,744	44,328,384	3	3	0_1_0_0_0_0_0_0_0_1_0_1_0.26	3	3	3	2	2	2	2	2	1	1	1	1	1
3602	19	44,328,385	44,328,663	3	2	1_0_1_1_1_0_0_0_0_0_1_0_0.101	2	2	2	3	3	3	3	3	1	1	1	1	1
3603	19	44,328,664	44,415,492	2	1	1_0_1_1_1_0_0_0_0_0_1_0_0.101	1	1	1	2	2	2	2	2	1	1	1	1	1
3604	19	44,415,493	44,457,060	3	2	0_1_0_0_0_0_0_0_0_1_0_1_0.63	3	3	3	2	2	2	2	2	1	1	1	1	1
3605	19	44,457,061	44,479,800	2	1	1_0_1_1_1_0_0_0_0_0_1_0_0.101	1	1	1	2	2	2	2	2	1	1	1	1	1
3606	19	44,479,801	44,550,877	1	0														
3607	19	44,550,878	44,841,664	2	1	1_0_1_1_1_0_0_0_0_0_1_0_0.255	1	1	1	2	2	2	2	2	1	1	1	1	1
3608	19	44,841,665	44,913,993	1	0														
3609	19	44,913,994	45,105,447	2	1	1_1_1_1_1_0_0_0_0_1_1_1_1.164	2	2	2	2	2	2	2	2	1	2	1	1	1
3610	19	45,105,448	45,254,926	1	0														
3611	19	45,254,927	48,262,399	2	1	0_1_0_0_0_1_1_1_1_1_0_1_0.451	2	2	2	1	1	1	1	1	2	1	2	2	2
3612	19	48,262,400	48,264,756	1	0														
3613	19	48,264,757	48,290,288	2	1	1_0_1_1_1_1_1_1_1_0_1_0_0.65	1	1	1	2	2	2	2	2	2	1	2	2	2
3614	19	48,290,289	48,319,478	3	2	0_0_0_0_0_0_0_0_0_0_0_0_1.111	1	1	1	2	2	2	2	2	2	3	2	2	2
3615	19	48,319,479	48,368,066	2	1	0_0_0_0_0_0_0_0_0_0_0_0_1.111	1	1	1	1	1	1	1	1	1	2	1	1	1
3616	19	48,368,067	48,383,565	3	2	0_0_0_0_0_0_0_0_0_0_0_0_1.111	2	2	2	2	2	2	2	2	1	3	1	1	1
3617	19	48,383,566	49,013,980	2	1	0_0_0_0_0_0_0_0_0_0_0_0_1.111	1	1	1	1	1	1	1	1	1	2	1	1	1
3618	19	49,013,981	49,032,781	3	2	0_0_0_0_0_0_0_0_0_0_0_0_1.111	2	2	2	2	2	2	2	2	1	3	1	2	1
3619	19	49,032,782	49,112,806	2	1	1_1_1_1_1_0_0_1_0_1_1_1_0.44	2	2	2	2	2	2	2	2	1	1	1	2	1
3620	19	49,112,807	49,387,974	1	0														
3621	19	49,387,975	50,023,752	2	1	1_1_1_1_1_0_0_1_0_1_1_1_0.923	2	2	2	2	2	2	2	2	1	1	1	2	1
3622	19	50,023,753	50,030,605	1	0														
3623	19	50,030,606	50,814,733	2	1	1_1_1_1_1_0_0_1_0_1_1_1_1.974	2	2	2	2	2	2	2	2	1	2	1	2	1
3624	19	50,814,734	50,815,028	3	2	0_1_0_0_0_0_0_0_0_1_0_1_0.218	3	3	3	2	2	2	2	2	1	2	1	2	1
3625	19	50,815,029	51,119,007	3	3	0_1_0_0_0_0_0_0_0_1_0_1_0.218	3	3	3	2	2	2	2	2	1	2	1	2	1
3626	19	51,119,008	51,207,715	3	2	1_0_1_1_1_0_0_1_0_0_1_0_1.424	2	2	2	3	3	3	3	3	1	3	1	3	1
3627	19	51,207,716	51,293,559	2	1	1_0_1_1_1_0_0_1_0_0_1_0_1.424	1	1	1	2	2	2	2	2	1	2	1	2	1
3628	19	51,293,560	51,332,222	3	2	0_1_0_0_0_0_0_0_0_1_0_1_0.335	3	3	3	2	2	2	2	2	1	2	1	2	1
3629	19	51,332,223	51,674,982	2	1	0_1_0_0_0_0_0_0_0_1_0_1_0.335	2	2	2	1	1	1	1	1	1	1	1	1	1
3630	19	51,674,983	51,675,394	1	0														
3631	19	51,675,395	51,859,480	2	1	1_1_1_1_1_0_0_0_0_1_1_1_0.126	2	2	2	2	2	2	2	2	1	1	1	1	1
3632	19	51,859,481	51,866,286	3	2	1_0_1_1_1_0_0_0_0_0_1_0_0.304	2	2	2	3	3	3	3	3	1	1	1	1	1
3633	19	51,866,287	52,325,718	3	3	0_1_0_0_0_0_0_0_0_1_0_1_0.370	3	3	3	2	2	2	2	2	1	1	1	1	1
3634	19	52,325,719	52,326,039	3	2	1_0_1_1_1_0_0_0_0_0_1_0_0.304	2	2	2	3	3	3	3	3	1	1	1	1	1
3635	19	52,326,040	52,771,502	2	1	1_1_1_1_1_0_0_0_0_1_1_1_0.126	2	2	2	2	2	2	2	2	1	1	1	1	1
3636	19	52,771,503	52,773,934	3	2	0_1_0_0_0_0_0_0_0_1_0_1_0.220	3	3	3	2	2	2	2	2	1	1	1	1	1
3637	19	52,773,935	53,687,784	3	3	0_1_0_0_0_0_0_0_0_1_0_1_0.220	3	3	3	2	2	2	2	2	1	1	1	1	1
3638	19	53,687,785	53,724,335	3	2	0_1_0_0_0_0_0_0_0_1_0_1_0.220	3	3	3	2	2	2	2	2	1	1	1	1	1
3639	19	53,724,336	53,863,317	2	1	1_1_1_1_1_0_0_0_0_1_1_1_0.126	2	2	2	2	2	2	2	2	1	1	1	1	1
3640	19	53,863,318	53,868,423	1	0														
3641	19	53,868,424	54,372,251	2	1	0_1_0_0_0_0_0_0_0_1_0_1_0.106	2	2	2	1	1	1	1	1	1	1	1	1	1
3642	19	54,372,252	54,387,338	3	2	0_1_0_0_0_0_0_0_0_1_0_1_0.106	3	3	3	2	2	2	2	2	1	1	1	1	1
3643	19	54,387,339	55,921,166	3	3	0_1_0_0_0_0_0_0_0_1_0_1_0.106	3	3	3	2	2	2	2	2	1	1	1	1	1
3644	19	55,921,167	56,003,904	3	2	1_0_1_1_1_0_0_0_0_0_1_0_0.883	2	2	2	3	3	3	3	3	1	1	1	1	1
3645	19	56,003,905	56,087,556	3	3	0_1_0_0_0_0_0_0_0_1_0_1_0.338	3	3	3	2	2	2	2	2	1	1	1	1	1
3646	19	56,087,557	56,200,593	3	2	0_1_0_0_0_0_0_0_0_1_0_1_0.338	3	3	3	2	2	2	2	2	1	1	1	1	1
3647	19	56,200,594	56,672,802	3	3	0_1_0_0_0_0_0_0_0_1_0_1_0.338	3	3	3	2	2	2	2	2	1	1	1	1	1
3648	19	56,672,803	56,754,089	3	2	0_1_0_0_0_0_0_0_0_1_0_1_0.338	3	3	3	2	2	2	2	2	1	1	1	1	1

Disease Models & Mechanisms • Supplementary information

3649	19	56,754,090	56,853,064	2	1	1_0_1_1_1_0_0_0_0_0_1_0_0.105	1	1	1	2	2	2	2	2	1	1	1	1	1
3650	19	56,853,065	56,858,030	3	2	0_1_0_0_0_0_0_0_0_1_0_1_0.602	3	3	3	2	2	2	2	2	1	1	1	1	1
3651	19	56,858,031	57,486,307	3	3	0_1_0_0_0_0_0_0_0_1_0_1_0.602	3	3	3	2	2	2	2	2	1	1	1	1	1
3652	19	57,486,308	57,575,734	3	2	1_0_1_1_1_0_0_0_0_0_1_0_0.105	2	2	2	3	3	3	3	3	1	1	1	1	1
3653	19	57,575,735	57,941,678	2	1	1_1_1_1_1_0_0_0_0_1_1_1_0.145	2	2	2	2	2	2	2	2	1	1	1	1	1
3654	19	57,941,679	57,954,810	3	2	0_1_0_0_0_0_0_0_0_1_0_1_0.245	3	3	3	2	2	2	2	2	1	1	1	1	1
3655	19	57,954,811	58,253,410	3	3	0_1_0_0_0_0_0_0_0_1_0_1_0.245	3	3	3	2	2	2	2	2	1	1	1	1	1
3656	19	58,253,411	58,321,496	3	2	1_0_1_1_1_0_0_0_0_0_1_0_0.280	2	2	2	3	3	3	3	3	1	1	1	1	1
3657	19	58,321,497	58,455,325	3	3	0_1_0_0_0_0_0_0_0_1_0_1_0.85	3	3	3	2	2	2	2	2	1	1	1	1	1
3658	19	58,455,326	58,467,479	3	2	0_1_0_0_0_0_0_0_0_1_0_1_0.85	3	3	3	2	2	2	2	2	1	1	1	1	1
3659	19	58,467,480	58,504,490	2	1	1_1_1_1_1_0_0_0_0_1_1_1_0.145	2	2	2	2	2	2	2	2	1	1	1	1	1
3660	19	58,504,491	58,509,903	3	2	1_0_1_1_1_0_0_0_0_0_1_0_0.183	2	2	2	3	3	3	3	3	1	1	1	1	1
3661	19	58,509,904	58,650,915	3	3	0_1_0_0_0_0_0_0_0_1_0_1_0.516	3	3	3	2	2	2	2	2	1	1	1	1	1
3662	19	58,650,916	58,697,767	3	2	0_1_0_0_0_0_0_0_0_1_0_1_0.516	3	3	3	2	2	2	2	2	1	1	1	1	1
3663	19	58,697,768	58,719,886	2	1	0_1_0_0_0_0_0_0_0_1_0_1_0.516	2	2	2	1	1	1	1	1	1	1	1	1	1
3664	19	58,719,887	58,859,801	3	2	0_1_0_0_0_0_0_0_0_1_0_1_0.516	3	3	3	2	2	2	2	2	1	1	1	1	1
3665	19	58,859,802	59,066,046	2	1	0_1_0_0_0_0_0_0_0_1_0_1_0.516	2	2	2	1	1	1	1	1	1	1	1	1	1
3666	19	59,066,047	59,318,429	1	0														
3667	19	59,318,430	60,305,823	2	1	1_1_1_1_1_0_0_0_0_1_1_1_0.725	2	2	2	2	2	2	2	2	1	1	1	1	1
3668	20	54,204	618,344	2	1	1_1_1_1_1_0_0_0_0_1_1_1_1.707	2	2	2	2	2	2	2	2	1	2	1	1	1
3669	20	618,345	829,789	1	0														
3670	20	829,790	3,139,603	2	1	1_1_1_1_1_0_0_0_0_1_1_1_1.759	2	2	2	2	2	2	2	2	1	2	1	1	1
3671	20	3,139,604	3,305,962	1	0														
3672	20	3,305,963	3,655,209	2	1	1_1_1_1_1_0_0_0_0_1_1_1_0.325	2	2	2	2	2	2	2	2	1	1	1	1	1
3673	20	3,655,210	3,660,400	1	0														
3674	20	3,660,401	5,757,380	2	1	1_1_1_1_1_0_0_0_0_1_1_1_1.199	2	2	2	2	2	2	2	2	1	2	1	1	1
3675	20	5,757,381	5,791,793	1	0														
3676	20	5,791,794	6,524,642	2	1	1_1_1_1_1_0_1_0_0_1_1_1_0.870	2	2	2	2	2	2	2	2	2	1	1	1	1
3677	20	6,524,643	6,526,982	1	0														
3678	20	6,526,983	6,805,895	2	1	1_1_1_1_1_0_0_0_0_1_1_1_1.113	2	2	2	2	2	2	2	2	1	2	1	1	1
3679	20	6,805,896	6,860,683	1	0														
3680	20	6,860,684	7,053,414	2	1	1_1_1_1_1_0_1_0_0_1_1_1_0.173	2	2	2	2	2	2	2	2	2	1	1	1	1
3681	20	7,053,415	7,130,330	1	0														
3682	20	7,130,331	7,204,129	2	1	1_1_1_1_1_0_0_0_0_1_1_1_1.41	2	2	2	2	2	2	2	2	1	2	1	1	1
3683	20	7,204,130	7,211,053	1	0														
3684	20	7,211,054	8,074,297	2	1	1_1_1_1_1_0_1_0_0_1_1_1_1.302	2	2	2	2	2	2	2	2	2	2	1	1	1
3685	20	8,074,298	8,093,083	1	0														
3686	20	8,093,084	8,127,114	2	1	1_1_1_1_1_0_1_0_0_1_1_1_0.43	2	2	2	2	2	2	2	2	2	1	1	1	1
3687	20	8,127,115	8,284,802	1	0														
3688	20	8,284,803	9,904,601	2	1	1_1_1_1_1_0_1_0_0_1_1_1_0.175	2	2	2	2	2	2	2	2	2	1	1	1	1
3689	20	9,904,602	10,193,474	1	0														
3690	20	10,193,475	10,427,656	2	1	1_1_1_1_1_0_1_0_0_0_1_0_0.296	2	1	1	2	2	2	2	2	2	1	1	1	1
3691	20	10,427,657	10,446,943	1	0														
3692	20	10,446,944	10,915,104	2	1	0_0_0_0_0_1_1_1_1_1_0_1_1.479	1	2	2	1	1	1	1	1	2	2	2	2	2
3693	20	10,915,105	10,916,182	1	0														
3694	20	10,916,183	10,917,976	2	1	1_1_1_1_1_0_0_0_0_1_1_1_1.71	2	2	2	2	2	2	2	2	1	2	1	1	1
3695	20	10,917,977	10,919,499	3	2	0_0_0_0_0_0_0_0_0_0_0_0_1.569	2	2	2	2	2	2	2	2	1	3	1	1	1
3696	20	10,919,500	11,148,257	3	3	0_0_0_0_0_0_0_0_0_0_0_0_1.569	2	2	2	2	2	2	2	2	1	3	1	1	1
3697	20	11,148,258	11,165,996	3	2	0_0_0_0_0_0_0_0_0_0_0_0_1.569	2	2	2	2	2	2	2	2	1	3	1	1	1
3698	20	11,165,997	11,242,078	2	1	0_0_0_0_0_0_0_0_0_0_0_0_1.569	1	1	1	1	1	1	1	1	1	2	1	1	1
3699	20	11,242,079	11,410,802	3	2	0_0_0_0_0_0_0_0_0_0_0_0_1.569	2	2	2	2	2	2	2	2	1	3	1	1	1
3700	20	11,410,803	11,411,597	2	1	1_1_1_1_1_0_0_0_0_1_1_1_0.176	2	2	2	2	2	2	2	2	1	1	1	1	1
3701	20	11,411,598	11,857,659	1	0														
3702	20	11,857,660	13,453,763	2	1	1_1_1_1_1_0_0_0_0_1_1_1_1.844	2	2	2	2	2	2	2	2	1	2	1	1	1
3703	20	13,453,764	13,781,435	1	0														
3704	20	13,781,436	13,986,195	2	1	1_1_1_1_1_0_0_0_0_1_1_1_0.138	2	2	2	2	2	2	2	2	1	1	1	1	1
3705	20	13,986,196	19,930,566	1	0														
3706	20	19,930,567	20,062,788	2	1	1_0_1_1_1_1_1_1_1_0_1_0_0.168	1	1	1	2	2	2	2	2	2	1	2	2	2
3707	20	20,062,789	20,065,167	1	0														
3708	20	20,065,168	20,065,640	2	1	0_1_0_0_0_0_0_0_0_1_0_1_0.50	2	2	2	1	1	1	1	1	1	1	1	1	1
3709	20	20,065,641	20,067,837	3	2	0_1_0_0_0_0_0_0_0_1_0_1_0.50	3	3	3	2	2	2	2	2	1	1	1	1	1
3710	20	20,067,838	20,128,234	3	3	0_1_0_0_0_0_0_0_0_1_0_1_0.50	3	3	3	2	2	2	2	2	1	1	1	1	1
3711	20	20,128,235	20,212,972	3	2	1_0_1_1_1_0_0_0_0_0_1_0_0.45	2	2	2	3	3	3	3	3	1	1	1	1	1
3712	20	20,212,973	20,451,629	2	1	1_1_1_1_1_0_0_0_0_1_1_1_0.480	2	2	2	2	2	2	2	2	1	1	1	1	1
3713	20	20,451,630	22,224,564	1	0														
3714	20	22,224,565	25,782,159	2	1	1_1_1_1_1_0_1_0_0_1_1_1_0.283	2	2	2	2	2	2	2	2	2	1	1	1	1
3715	20	25,782,160	26,406,286	1	0														
3716	20	26,406,287	28,364,853	2	1	1_1_1_1_1_0_1_0_0_1_1_1_0.120	2	2	2	2	2	2	2	2	2	1	1	1	1
3717	20	28,364,854	29,136,370	1	0														
3718	20	29,136,371	29,792,124	2	1	1_1_1_1_1_0_1_0_0_1_1_1_0.597	2	2	2	2	2	2	2	2	2	1	1	1	1
3719	20	29,792,125	30,934,828	1	0														
3720	20	30,934,829	34,728,613	2	1	1_1_1_1_1_0_1_0_0_1_1_1_0.297	2	2	2	2	2	2	2	2	2	1	1	1	1
3721	20	34,728,614	39,081,858	1	0														
3722	20	39,081,859	40,212,386	2	1	1_1_1_1_1_0_1_0_0_1_1_1_0.164	2	2	2	2	2	2	2	2	2	1	1	1	1
3723	20	40,212,387	40,217,168	1	0														
3724	20	40,217,169	40,541,747	2	1	1_1_1_1_1_0_1_0_0_1_1_1_1.185	2	2	2	2	2	2	2	2	2	2	1	1	1
3725	20	40,541,748	40,765,215	1	0														
3726	20	40,765,216	41,971,529	2	1	1_1_1_1_1_0_1_0_0_1_1_1_0.134	2	2	2	2	2	2	2	2	2	1	1	1	1
3727	20	41,971,530	42,013,012	1	0														
3728	20	42,013,013	42,337,732	2	1	1_1_1_1_1_0_1_0_0_1_1_1_1.194	2	2	2	2	2	2	2	2	2	2	1	1	1
3729	20	42,337,733	42,338,905	1	0														
3730	20	42,338,906	42,396,916	2	1	1_1_1_1_1_0_1_0_0_1_1_1_0.138	2	2	2	2	2	2	2	2	2	1	1	1	1
3731	20	42,396,917	43,259,505	1	0														
3732	20	43,259,506	45,700,840	2	1	1_1_1_1_1_0_1_0_0_1_1_1_1.153	2	2	2	2	2	2	2	2	2	2	1	1	1
3733	20	45,700,841	46,311,511	1	0														

3734	20	46,311,512	48,453,371	2	1	1_1_1_1_1_0_1_0_0_1_1_1_0.134	2	2	2	2	2	2	2	2	2	1	1	1	1
3735	20	48,453,372	48,492,850	1	0														
3736	20	48,492,851	48,714,346	2	1	1_1_1_1_1_0_1_0_0_1_1_1_1.51	2	2	2	2	2	2	2	2	2	2	1	1	1
3737	20	48,714,347	48,787,726	1	0														
3738	20	48,787,727	49,169,902	2	1	1_1_1_1_1_0_1_0_0_1_1_1_0.783	2	2	2	2	2	2	2	2	2	1	1	1	1
3739	20	49,169,903	49,398,326	1	0														
3740	20	49,398,327	49,855,149	2	1	1_1_1_1_1_0_1_0_0_1_1_1_0.416	2	2	2	2	2	2	2	2	2	1	1	1	1
3741	20	49,855,150	49,925,817	1	0														
3742	20	49,925,818	50,450,817	2	1	1_1_1_1_1_0_1_0_0_1_1_1_0.821	2	2	2	2	2	2	2	2	2	1	1	1	1
3743	20	50,450,818	50,452,406	1	0														
3744	20	50,452,407	50,786,829	2	1	0_0_0_0_0_0_0_0_0_0_0_0_1.268	1	1	1	1	1	1	1	1	1	2	1	1	1
3745	20	50,786,830	51,874,492	1	0														
3746	20	51,874,493	51,891,022	2	1	0_0_0_0_0_0_0_0_0_0_0_0_1.165	1	1	1	1	1	1	1	1	1	2	1	1	1
3747	20	51,891,023	52,011,571	3	2	0_0_0_0_0_0_0_0_0_0_0_0_1.165	2	2	2	2	2	2	2	2	2	3	1	1	1
3748	20	52,011,572	52,098,265	3	3	0_0_0_0_0_0_0_0_0_0_0_0_1.165	2	2	2	2	2	2	2	2	2	3	1	1	1
3749	20	52,098,266	52,112,288	3	2	0_0_0_0_0_0_0_0_0_0_0_0_1.165	2	2	2	2	2	2	2	2	2	3	1	1	1
3750	20	52,112,289	52,157,180	3	3	0_0_0_0_0_0_0_0_0_0_0_0_1.165	2	2	2	2	2	2	2	2	2	3	1	1	1
3751	20	52,157,181	52,168,927	3	2	0_0_0_0_0_0_0_0_0_0_0_0_1.165	2	2	2	2	2	2	2	2	2	3	1	1	1
3752	20	52,168,928	52,213,612	2	1	0_0_0_0_0_0_0_0_0_0_0_0_1.165	1	1	1	1	1	1	1	1	1	2	1	1	1
3753	20	52,213,613	52,368,796	3	2	0_0_0_0_0_0_0_0_0_0_0_0_1.165	2	2	2	2	2	2	2	2	2	3	1	1	1
3754	20	52,368,797	52,401,397	2	1	0_0_0_0_0_0_0_0_0_0_0_0_1.165	1	1	1	1	1	1	1	1	1	2	1	1	1
3755	20	52,401,398	52,572,630	3	2	0_0_0_0_0_0_0_0_0_0_0_0_1.165	2	2	2	2	2	2	2	2	2	3	1	1	1
3756	20	52,572,631	52,655,371	2	1	0_0_0_0_0_0_0_0_0_0_0_0_1.165	1	1	1	1	1	1	1	1	1	2	1	1	1
3757	20	52,655,372	52,931,373	3	2	0_0_0_0_0_0_0_0_0_0_0_0_1.165	2	2	2	2	2	2	2	2	2	3	1	1	1
3758	20	52,931,374	52,979,025	2	1	1_1_1_1_1_0_1_0_0_1_1_1_0.657	2	2	2	2	2	2	2	2	2	1	1	1	1
3759	20	52,979,026	53,203,961	3	2	0_0_0_0_0_0_0_0_0_0_0_0_1.938	2	2	2	2	2	2	2	2	2	3	1	1	1
3760	20	53,203,962	53,441,265	3	3	0_0_0_0_0_0_0_0_0_0_0_0_1.938	2	2	2	2	2	2	2	2	2	3	1	1	1
3761	20	53,441,266	53,570,743	3	2	0_0_0_0_0_0_0_0_0_0_0_0_1.938	2	2	2	2	2	2	2	2	2	3	1	1	1
3762	20	53,570,744	53,766,767	2	1	0_0_0_0_0_0_0_0_0_0_0_0_1.938	1	1	1	1	1	1	1	1	1	2	1	1	1
3763	20	53,766,768	53,997,632	3	2	0_0_0_0_0_0_0_0_0_0_0_0_1.938	2	2	2	2	2	2	2	2	2	3	1	1	1
3764	20	53,997,633	54,175,335	3	3	0_0_0_0_0_0_0_0_0_0_0_0_1.938	2	2	2	2	2	2	2	2	2	3	1	1	1
3765	20	54,175,336	54,281,567	3	2	0_0_0_0_0_0_0_0_0_0_0_0_1.938	2	2	2	2	2	2	2	2	2	3	1	1	1
3766	20	54,281,568	54,639,130	3	3	0_0_0_0_0_0_0_0_0_0_0_0_1.938	2	2	2	2	2	2	2	2	2	3	1	1	1
3767	20	54,639,131	54,925,251	3	2	0_0_0_0_0_0_0_0_0_0_0_0_1.938	2	2	2	2	2	2	2	2	2	3	1	1	1
3768	20	54,925,252	55,126,986	3	3	0_0_0_0_0_0_0_0_0_0_0_0_1.938	2	2	2	2	2	2	2	2	2	3	1	1	1
3769	20	55,126,987	55,214,160	3	2	1_1_1_1_1_0_1_0_0_1_1_1_0.873	3	3	3	3	3	3	3	3	3	2	1	1	1
3770	20	55,214,161	55,613,581	3	3	0_0_0_0_0_0_0_0_0_0_0_0_1.206	2	2	2	2	2	2	2	2	2	3	1	1	1
3771	20	55,613,582	55,614,590	3	2	1_1_1_1_1_0_1_0_0_1_1_1_0.873	3	3	3	3	3	3	3	3	3	2	1	1	1
3772	20	55,614,591	56,200,536	2	1	1_1_1_1_1_0_1_0_0_1_1_1_1.422	2	2	2	2	2	2	2	2	2	2	1	1	1
3773	21	730,814	1,768,805	2	1	0_1_0_0_0_0_0_0_0_1_1_1_1.190	2	2	2	1	1	1	1	2	1	2	1	1	1
3774	21	1,768,806	6,120,277	3	2	0_1_0_0_0_0_0_0_0_1_1_1_1.190	3	3	3	1	1	1	1	3	2	3	2	2	2
3775	21	6,120,278	6,332,680	2	1	0_1_0_0_0_0_0_0_0_1_1_1_1.190	2	2	2	1	1	1	1	2	1	2	1	1	1
3776	21	6,332,681	6,342,308	3	2	0_1_0_0_0_0_0_0_0_1_1_1_1.190	3	3	3	2	2	2	2	3	1	3	1	1	1
3777	21	6,342,309	6,365,697	5	3	0_1_0_0_0_0_0_0_0_1_1_1_1.190	5	5	5	3	3	3	3	5	2	4	1	1	1
3778	21	6,365,698	8,592,662	3	2	0_1_0_0_0_0_0_0_0_1_1_1_1.190	3	3	3	2	2	2	2	3	1	3	1	1	1
3779	21	8,592,663	9,977,455	2	1	1_1_1_1_1_0_0_0_0_1_1_1_1.300	2	2	2	2	2	2	2	2	1	2	1	1	1
3780	21	9,977,456	10,858,060	3	2	0_1_0_0_0_0_0_0_0_1_1_1_1.141	3	3	3	2	2	2	2	3	1	3	1	1	1
3781	21	10,858,061	11,268,842	2	1	0_1_0_0_0_0_0_0_0_1_1_1_1.141	2	2	2	1	1	1	1	2	1	2	1	1	1
3782	21	11,268,843	11,506,783	3	2	0_1_0_0_0_0_0_0_0_1_1_1_1.141	3	3	3	2	2	2	2	3	1	3	1	1	1
3783	21	11,506,784	11,685,599	2	1	1_1_1_1_1_0_0_0_0_1_1_1_1.38	2	2	2	2	2	2	2	2	1	2	1	1	1
3784	21	11,685,600	12,124,452	1	0														
3785	21	12,124,453	15,778,761	2	1	0_1_0_0_0_1_1_1_1_1_1_1_1.291	2	2	2	1	1	1	1	2	2	2	2	2	2
3786	21	15,778,762	27,002,060	1	0														
3787	21	27,002,061	27,492,881	2	1	0_1_0_0_0_1_1_1_1_1_1_1_1.69	2	2	2	1	1	1	1	2	2	2	2	2	2
3788	21	27,492,882	27,493,147	1	0														
3789	21	27,493,148	27,896,659	2	1	1_1_1_1_1_0_0_0_0_1_0_1_0.157	2	2	2	2	2	2	2	1	1	1	1	1	1
3790	21	27,896,660	27,919,945	3	2	0_0_0_0_0_0_0_0_0_0_0_0_1.37	2	2	2	2	2	2	2	1	1	3	1	1	1
3791	21	27,919,946	28,198,044	3	3	0_0_0_0_0_0_0_0_0_0_0_0_1.37	1	1	1	1	1	1	1	2	2	3	2	2	2
3792	21	28,198,045	28,413,232	3	2	0_0_0_0_0_1_1_1_1_0_1_0_0.40	2	2	2	2	2	2	2	3	3	1	3	3	3
3793	21	28,413,233	28,533,294	2	1	1_1_1_1_1_0_0_0_0_1_0_1_0.157	2	2	2	2	2	2	2	1	1	1	1	1	1
3794	21	28,533,295	28,557,706	1	0														
3795	21	28,557,707	29,256,982	2	1	0_0_0_0_0_0_0_0_0_0_1_0_0.185	1	1	1	1	1	1	1	2	1	1	1	1	1
3796	21	29,256,983	29,288,799	1	0														
3797	21	29,288,800	32,054,538	2	1	1_1_1_1_1_0_0_0_0_1_0_1_0.102	2	2	2	2	2	2	2	1	1	1	1	1	1
3798	21	32,054,539	32,078,822	3	2	0_0_0_0_0_0_0_0_0_0_1_0_0.27	2	2	2	2	2	2	2	3	1	1	1	1	1
3799	21	32,078,823	36,757,052	2	1	1_1_1_1_1_0_0_0_0_1_0_1_0.102	2	2	2	2	2	2	2	1	1	1	1	1	1
3800	21	36,757,053	48,966,273	1	0														
3801	21	48,966,274	48,973,022	2	1	1_0_1_1_1_0_0_0_0_0_1_0_0.51	1	1	1	2	2	2	2	2	1	1	1	1	1
3802	21	48,973,023	48,989,925	3	2	1_0_1_1_1_0_0_0_0_0_1_0_0.51	2	2	2	3	3	3	3	3	1	1	1	1	1
3803	21	48,989,926	49,398,552	3	3	0_1_0_0_0_0_0_0_0_1_0_1_0.87	3	3	3	2	2	2	2	2	1	1	1	1	1
3804	21	49,398,553	49,403,505	3	2	0_1_0_0_0_0_0_0_0_1_0_1_0.87	3	3	3	2	2	2	2	2	1	1	1	1	1
3805	21	49,403,506	49,404,844	2	1	1_1_1_1_1_0_0_0_0_1_1_1_0.40	2	2	2	2	2	2	2	2	1	1	1	1	1
3806	21	49,404,845	52,967,856	1	0														
3807	21	52,967,857	56,156,175	2	1	1_1_1_1_1_0_0_0_0_1_0_1_0.228	2	2	2	2	2	2	2	1	1	1	1	1	1
3808	21	56,156,176	57,084,722	1	0														
3809	21	57,084,723	64,388,375	2	1	1_1_1_1_1_0_0_0_0_1_1_1_0.617	2	2	2	2	2	2	2	2	1	1	1	1	1
3810	21	64,388,376	65,473,360	3	2	0_0_0_0_0_0_0_0_0_0_0_0_1.283	2	2	2	2	2	2	2	2	1	3	1	1	1
3811	21	65,473,361	67,287,190	2	1	0_0_0_0_0_0_0_0_0_0_0_0_1.283	1	1	1	1	1	1	1	1	1	2	1	1	1
3812	21	67,287,191	67,320,546	3	2	0_0_0_0_0_0_0_0_0_0_0_0_1.283	2	2	2	2	2	2	2	2	1	3	1	1	1
3813	21	67,320,547	68,126,209	2	1	1_1_1_1_1_0_0_0_0_1_1_1_0.32	2	2	2	2	2	2	2	2	1	1	1	1	1
3814	21	68,126,210	75,897,565	1	0														
3815	21	75,897,566	76,995,119	2	1	1_1_1_1_1_0_0_0_0_1_0_1_0.164	2	2	2	2	2	2	2	1	1	1	1	1	1
3816	21	76,995,120	80,002,781	3	2	0_1_0_0_0_0_0_0_0_1_0_1_0.159	3	3	3	2	2	2	2	1	1	1	1	1	1
3817	21	80,002,782	80,035,602	3	3	0_1_0_0_0_0_0_0_0_1_0_1_0.159	3	3	3	1	1	1	1	2	2	2	2	2	2
3818	21	80,035,603	80,145,789	3	2	0_1_0_0_0_0_0_0_0_1_0_1_0.159	3	3	3	1	1	1	1	2	2	2	2	2	2

Disease Models & Mechanisms • Supplementary information

3819	21	80,145,790	80,148,279	2	1	0_1_0_0_0_0_0_0_0_1_0_1_0.159	2	2	2	1	1	1	1	1	1	1	1	1	1
3820	21	80,148,280	80,886,206	3	2	0_1_0_0_0_0_0_0_0_1_0_1_0.159	3	3	3	2	2	2	2	2	1	1	1	1	1
3821	21	80,886,207	81,875,334	2	1	0_1_0_0_0_0_0_0_0_1_0_1_0.159	2	2	2	1	1	1	1	1	1	1	1	1	1
3822	21	81,875,335	82,190,617	3	2	0_1_0_0_0_0_0_0_0_1_0_1_0.159	3	3	3	2	2	2	2	2	1	1	1	1	1
3823	21	82,190,618	99,873,453	3	3	0_1_0_0_0_0_0_0_0_1_0_1_0.159	3	3	3	2	2	2	2	2	1	1	1	1	1
3824	21	99,873,454	99,882,483	3	2	0_1_0_0_0_0_0_0_0_1_0_1_0.159	3	3	3	2	2	2	2	2	1	1	1	1	1
3825	21	99,882,484	99,889,494	2	1	0_1_0_0_0_0_0_0_0_1_0_1_0.159	2	2	2	1	1	1	1	1	1	1	1	1	1
3826	21	99,889,495	99,890,537	3	2	0_1_0_0_0_0_0_0_0_1_0_1_0.159	3	3	3	1	1	1	1	2	1	1	1	1	1
3827	21	99,890,538	100,331,734	3	3	0_0_0_0_0_0_0_0_0_0_1_0_0.272	2	2	2	1	1	1	1	3	1	1	1	1	1
3828	21	100,331,735	100,361,947	3	2	0_0_0_0_0_0_0_0_0_0_1_0_0.272	2	2	2	1	1	1	1	3	1	1	1	1	1
3829	21	100,361,948	101,660,381	2	1	0_0_0_0_0_0_0_0_0_0_1_0_0.272	1	1	1	1	1	1	1	2	1	1	1	1	1
3830	21	101,660,382	105,151,536	1	0														
3831	21	105,151,537	105,739,048	2	1	0_1_0_0_0_0_0_0_0_1_1_1_0.236	2	2	2	1	1	1	1	2	1	1	1	1	1
3832	21	105,739,049	105,743,748	1	0														
3833	21	105,743,749	106,653,372	2	1	1_1_1_1_1_0_0_0_0_1_1_1_0.229	2	2	2	2	2	2	2	2	1	1	1	1	1
3834	21	106,653,373	106,664,867	1	0														
3835	21	106,664,868	110,662,271	2	1	0_0_0_0_0_0_0_0_0_0_1_0_0.332	1	1	1	1	1	1	1	2	1	1	1	1	1
3836	21	110,662,272	110,700,060	1	0														
3837	21	110,700,061	116,208,966	2	1	1_1_1_1_1_0_0_0_0_1_0_1_0.111	2	2	2	2	2	2	2	1	1	1	1	1	1
3838	21	116,208,967	116,215,635	1	0														
3839	21	116,215,636	116,596,329	2	1	0_0_0_0_0_0_0_0_0_0_1_0_0.79	1	1	1	1	1	1	1	2	1	1	1	1	1
3840	21	116,596,330	118,096,282	1	0														
3841	21	118,096,283	118,856,091	2	1	1_1_1_1_1_0_0_0_0_1_0_1_0.166	2	2	2	2	2	2	2	1	1	1	1	1	1
3842	21	118,856,092	119,297,417	1	0														
3843	21	119,297,418	119,517,235	2	1	1_1_1_1_1_0_0_0_0_1_1_1_0.42	2	2	2	2	2	2	2	2	1	1	1	1	1
3844	21	119,517,236	119,618,059	1	0														
3845	21	119,618,060	120,547,024	2	1	0_0_0_0_0_0_0_0_0_0_1_0_0.667	1	1	1	1	1	1	1	2	1	1	1	1	1
3846	21	120,547,025	120,698,171	3	2	0_0_0_0_0_0_0_0_0_0_1_0_0.667	2	2	2	2	2	2	2	3	1	1	1	1	1
3847	21	120,698,172	123,370,159	2	1	0_0_0_0_0_0_0_0_0_0_1_0_0.667	1	1	1	1	1	1	1	2	1	1	1	1	1
3848	21	123,370,160	123,555,755	1	0														
3849	21	123,555,756	123,749,281	2	1	1_1_1_1_1_0_0_0_0_1_0_1_0.54	2	2	2	2	2	2	2	1	1	1	1	1	1
3850	21	123,749,282	123,900,993	1	0														
3851	21	123,900,994	129,066,425	2	1	1_1_1_1_1_0_0_0_0_1_1_1_0.532	2	2	2	2	2	2	2	2	1	1	1	1	1
3852	21	129,066,426	129,115,315	1	0														
3853	21	129,115,316	129,401,977	2	1	0_0_0_0_0_0_0_0_0_0_0_0_1.44	1	1	1	1	1	1	1	1	1	2	1	1	1
3854	21	129,401,978	131,385,589	1	0														
3855	21	131,385,590	133,487,932	2	1	1_1_1_1_1_0_0_0_0_1_1_1_0.787	2	2	2	2	2	2	2	2	1	1	1	1	1
3856	21	133,487,933	133,865,381	3	2	0_0_0_0_0_0_0_0_0_0_0_0_1.51	2	2	2	2	2	2	2	2	1	3	1	1	1
3857	21	133,865,382	138,654,445	2	1	1_1_1_1_1_0_0_0_0_1_1_1_0.787	2	2	2	2	2	2	2	2	1	1	1	1	1
3858	21	138,654,446	138,898,783	1	0														
3859	21	138,898,784	139,537,737	2	1	1_1_1_1_1_0_0_0_0_1_1_1_1.124	2	2	2	2	2	2	2	2	1	2	1	1	1
3860	21	139,537,738	140,225,502	1	0														
3861	21	140,225,503	140,950,116	2	1	1_0_1_1_1_0_0_0_0_0_1_0_0.110	1	1	1	2	2	2	2	2	1	1	1	1	1
3862	21	140,950,117	159,553,583	1	0														
3863	21	159,553,584	159,949,745	2	1	1_0_1_1_1_0_0_0_0_0_1_0_0.41	1	1	1	2	2	2	2	2	1	1	1	1	1

Table S2. Progenitor strain samples used for microarray analysis of target tissues

	Heart	Whole kidney	Renal cortex	Aorta	Liver
SHR/Izm	4	8	4	4	4
SHRSP/Izm	16	4	5	5	4
WKY/Izm	20<br>(4 vs SHR,<br>16 vs SHRSP)	12<br>(8 vs SHR,<br>4 vs SHRSP)	5	9<br>(4 vs SHR,<br>5 vs SHRSP)	6

Eight (4 SHR/Izm and 4 WKY/Izm) samples are overlapped between whole kidney and renal cortex.

Two pairs of inter-strain comparisons (WKY/Izm vs SHR/Izm and WKY/Izm vs SHRSP/Izm) are made separately for the heart, whole kidney and aorta, and all the data are combined by regression.

Table S3A. A list of significant transcripts for shared differential expression in the heart.

no	ProbeName	SHR/lzm (vs WKY/lzm)			SHRSP/lzm (vs WKY/lzm)			GeneName	Ensembl_rat	Chr	Transcription_start_site (Rnor_6.0)
		Fold change (log2-scale)	SE (log2-scale)	P-value	Fold change (log2-scale)	SE (log2-scale)	P-value				
13786	A_44_P101700	0.602	0.106	1.9E-06	0.374	0.053	2.7E-08	Stx11	ENSRNOG00000014902	1	7,064,870
814	A_44_P445359	0.649	0.088	1.2E-08	0.471	0.044	1.1E-12	Pex7	ENSRNOG00000012322	1	15,374,850
8464	A_64_P129293	1.418	0.171	1.1E-09	1.474	0.070	3.6E-21	RGD1306565	ENSRNOG00000031700	1	15,412,603
8390	A_43_P23129	-0.327	0.065	1.3E-05	-0.453	0.032	4.0E-16	Ptprk	ENSRNOG00000047605	1	18,058,055
1771	A_43_P12841	0.502	0.110	5.3E-05	0.254	0.055	4.6E-05	Enpp1	ENSRNOG00000013994	1	21,748,261
3394	A_42_P484738	1.000	0.123	1.1E-09	1.278	0.062	1.1E-21	Ctgf	ENSRNOG00000015036	1	21,854,773
6563	A_64_P119169	-0.329	0.054	4.8E-07	-0.342	0.027	6.5E-15	LOC684270	ENSRNOG00000050348	1	72,509,431
6169	A_64_P058871	0.525	0.119	8.6E-05	0.420	0.059	2.6E-08	Tnnt1	ENSRNOG00000028041	1	72,889,278
18278	A_44_P156211	0.505	0.113	7.8E-05	0.331	0.057	1.1E-06	Itpkc	ENSRNOG00000013945	1	84,040,433
28121	A_64_P027503	0.879	0.137	1.9E-07	0.469	0.068	5.1E-08	Fbxo17	ENSRNOG00000019942	1	86,914,137
603	A_44_P1018090	0.445	0.076	1.1E-06	0.423	0.038	3.2E-13	Lgi4	ENSRNOG00000021087	1	89,491,654
10829	A_44_P449994	0.983	0.098	6.1E-12	0.800	0.049	3.4E-18	LOC365238	ENSRNOG00000015168	1	94,784,451
11981	A_64_P148550	0.600	0.108	2.9E-06	0.731	0.054	1.1E-15	Lim2	ENSRNOG00000017681	1	98,501,249
13915	A_64_P080941	-1.547	0.164	3.9E-11	-1.656	0.084	1.2E-20	LOC102556967	ENSRNOG00000033729	1	99,505,677
19170	A_64_P049828	-1.609	0.169	2.1E-11	-0.868	0.084	2.8E-12	Dbp	ENSRNOG00000021027	1	101,687,855
3773	A_64_P049827	-1.515	0.176	2.9E-10	-0.865	0.088	9.8E-12	Dbp	ENSRNOG00000021027	1	101,687,855
24946	A_64_P017188	-1.481	0.155	2.0E-11	-0.955	0.078	1.7E-14	Dbp	ENSRNOG00000021027	1	101,687,855
13613	A_44_P466271	0.423	0.079	4.9E-06	0.213	0.039	4.3E-06	Sphk2	ENSRNOG00000021032	1	101,697,277
1350	A_42_P506076	1.275	0.263	2.4E-05	0.670	0.132	1.1E-05	Ptpn5	ENSRNOG00000013981	1	103,256,823
16073	A_44_P997843	0.611	0.081	6.6E-09	0.624	0.041	2.0E-17	Csrp3	ENSRNOG00000014327	1	104,157,855
259	A_44_P536125	0.836	0.121	4.3E-08	0.406	0.061	7.6E-08	Prmt3	ENSRNOG00000014829	1	105,113,595
7527	A_64_P151448	-0.712	0.095	7.7E-09	-0.589	0.048	1.5E-14	Fancf	ENSRNOG00000023968	1	107,232,305
225	A_42_P602570	2.254	0.188	3.5E-14	0.884	0.094	2.8E-11	Bex1	ENSRNOG00000033844	1	116,567,189
19183	A_64_P133177	-0.680	0.126	4.3E-06	-1.146	0.063	8.4E-20	Sema4b	ENSRNOG00000025167	1	141,986,145
26335	A_64_P157285	2.049	0.150	7.8E-16	2.170	0.075	1.4E-26		ENSRNOG00000025003	1	142,027,250
16081	A_43_P10751	0.467	0.089	7.5E-06	0.287	0.045	1.8E-07	Ngrn	ENSRNOG00000013553	1	142,050,458
5536	A_42_P528580	-0.338	0.055	4.7E-07	-0.301	0.028	5.6E-13	Hddc3	ENSRNOG00000012236	1	142,136,452
3363	A_64_P016059	-1.154	0.076	2.5E-17	-0.576	0.038	2.6E-17	Homer2	ENSRNOG00000061450	1	143,535,583
16902	A_64_P045536	-0.369	0.084	8.6E-05	-0.338	0.042	1.2E-09	Eftud1	ENSRNOG00000032723	1	144,601,410

70	A_42_P628981	0.543	0.105	8.4E-06	0.810	0.052	1.6E-17	Prss23	ENSRNOG00000017307	1	153,752,541
29997	A_42_P733209	-0.656	0.129	1.1E-05	-0.382	0.064	8.4E-07	Dgat2	ENSRNOG00000016573	1	164,143,818
10025	A_44_P301608	0.717	0.139	9.1E-06	1.128	0.069	3.4E-18	Ucp2	ENSRNOG00000017854	1	165,506,361
24566	A_43_P14847	-0.544	0.065	5.4E-10	-0.352	0.032	6.3E-13	Mrpl48	ENSRNOG00000018042	1	165,606,375
28025	A_44_P520159	-0.512	0.116	8.5E-05	-0.385	0.058	9.2E-08	P2ry2	ENSRNOG00000019283	1	166,037,424
29337	A_43_P22401	0.495	0.089	2.8E-06	0.451	0.045	4.5E-12	Lrrc51	ENSRNOG00000020124	1	167,005,839
599	A_64_P162476	-0.926	0.196	3.4E-05	-1.276	0.098	3.2E-15	Art1	ENSRNOG00000020251	1	167,197,549
18258	A_64_P042586	-0.950	0.136	3.6E-08	-1.005	0.068	7.4E-17	Art5	ENSRNOG00000020242	1	167,202,767
23520	A_64_P072633	-0.545	0.123	8.1E-05	-1.245	0.061	2.3E-21	Olr59	ENSRNOG00000018606	1	167,911,961
22874	A_64_P112431	1.082	0.205	6.2E-06	0.682	0.102	8.8E-08	Olr200	ENSRNOG00000048852	1	170,147,300
4890	A_42_P797965	1.687	0.107	7.9E-18	1.355	0.053	1.2E-24	RGD1306959	ENSRNOG00000013744	1	174,330,695
29915	A_64_P086565	0.619	0.064	1.5E-11	0.637	0.032	4.8E-21	Rras2	ENSRNOG00000012258	1	179,010,257
7347	A_42_P793598	0.472	0.094	1.3E-05	0.247	0.047	6.6E-06	LOC378467	ENSRNOG00000048250	1	189,870,622
9145	A_43_P12996	1.131	0.117	1.3E-11	0.650	0.058	2.9E-13	Crym	ENSRNOG00000061215	1	189,960,073
9654	A_64_P019021	2.218	0.267	3.5E-09	1.193	0.161	3.7E-08	LOC691551	ENSRNOG00000028473	1	190,914,946
3234	A_64_P084966	0.377	0.084	7.1E-05	-0.246	0.042	1.1E-06	Tnrc6a	ENSRNOG00000024737	1	193,076,430
8445	A_42_P791460	-0.342	0.066	8.0E-06	-0.379	0.033	1.1E-13	Jmjd5	ENSRNOG00000015180	1	196,839,321
16693	A_64_P089531	0.673	0.120	2.3E-06	0.612	0.060	3.5E-12	Il4ra	ENSRNOG00000015441	1	196,942,364
10614	A_44_P395538	-0.829	0.077	7.5E-13	-0.523	0.038	8.6E-16	Rabep2	ENSRNOG00000018462	1	197,839,430
12122	A_44_P321110	-0.320	0.067	3.2E-05	-0.289	0.034	2.9E-10	Eif3c	ENSRNOG00000018761	1	197,982,601
15860	A_44_P562972	0.521	0.103	1.3E-05	0.837	0.052	3.5E-18	Apob48r	ENSRNOG00000017403	1	197,999,037
5221	A_42_P812008	-0.963	0.097	7.0E-12	-0.338	0.048	3.4E-08	Nupr1	ENSRNOG00000050922	1	198,045,154
18482	A_44_P1002996	-0.788	0.105	7.4E-09	-0.866	0.053	2.2E-18	Ino80e	ENSRNOG00000019960	1	198,298,076
19479	A_44_P275029	-0.375	0.079	3.5E-05	-0.218	0.040	3.4E-06	Mvp	ENSRNOG00000020182	1	198,450,047
29205	A_44_P1029472	-0.906	0.069	2.3E-15	-0.430	0.034	1.1E-14	RGD1305592	ENSRNOG00000020217	1	198,450,688
6295	A_44_P473234	0.768	0.107	1.8E-08	0.886	0.053	1.7E-18	Znf688	ENSRNOG00000018379	1	198,869,009
14224	A_64_P045578	1.669	0.149	2.5E-13	1.304	0.074	3.0E-19	Rnf40	ENSRNOG00000018840	1	199,037,544
23664	A_64_P019325	0.930	0.066	3.1E-16	0.785	0.033	1.2E-23	Orai3	ENSRNOG00000039730	1	199,217,016
24684	A_64_P080856	0.334	0.075	8.3E-05	0.469	0.038	1.2E-14	RGD1310127	ENSRNOG00000020144	1	199,716,205
29778	A_44_P252921	-0.340	0.076	7.6E-05	-0.376	0.038	8.4E-12	Sec23ip	ENSRNOG00000020411	1	200,167,169
2815	A_42_P740370	0.688	0.106	1.4E-07	0.431	0.053	1.0E-09	Cpxm2	ENSRNOG00000015902	1	204,087,001
13842	A_64_P116485	0.571	0.088	1.6E-07	-0.320	0.044	1.5E-08	Pnpla2	ENSRNOG00000018736	1	214,434,626
5484	A_64_P153870	0.754	0.083	7.1E-11	0.465	0.041	2.5E-13	Chid1	ENSRNOG00000050181	1	214,511,529

22350	A_64_P097784	0.793	0.162	2.2E-05	0.559	0.084	1.3E-07	Fadd	ENSRNOG00000047035	1	217,748,628
5014	A_42_P614175	4.251	0.359	2.9E-13	1.344	0.153	4.8E-10	Gal	ENSRNOG00000015156	1	218,657,925
21228	A_64_P091940	4.413	0.351	8.0E-12	1.115	0.222	4.4E-05	Gal	ENSRNOG00000015156	1	218,657,925
4555	A_44_P367971	-0.441	0.094	3.8E-05	-0.289	0.047	4.2E-07	Ccdc85b	ENSRNOG00000046560	1	220,836,504
21831	A_64_P029622	-0.424	0.081	7.0E-06	-0.396	0.040	1.0E-11		ENSRNOG00000017884	1	223,549,904
21375	A_64_P024861	-0.420	0.090	4.4E-05	-0.278	0.045	4.3E-07		ENSRNOG00000017884	1	223,549,904
4232	A_64_P082401	0.545	0.099	3.0E-06	-0.245	0.049	1.6E-05	Ahnak	ENSRNOG00000057569	1	225,184,939
14221	A_64_P155741	-0.564	0.075	6.2E-09	-0.193	0.037	8.7E-06	Sdhaf2	ENSRNOG00000020646	1	226,572,349
30101	A_44_P896040	-0.916	0.081	2.0E-13	0.178	0.041	9.3E-05	Cybasc3	ENSRNOG00000020702	1	226,634,009
7124	A_64_P062545	-0.454	0.088	8.8E-06	0.626	0.044	2.0E-16	Tmem132a	ENSRNOG00000021338	1	226,924,244
11594	A_64_P146165	1.253	0.222	2.9E-06	1.543	0.125	9.7E-14	RGD1560242	ENSRNOG00000036615	1	241,460,868
5424	A_44_P315626	0.449	0.067	7.5E-08	0.257	0.033	3.9E-09	Kank1	ENSRNOG00000016023	1	243,276,403
16557	A_44_P310596	-0.713	0.133	4.8E-06	-0.515	0.066	3.2E-09	Il33	ENSRNOG00000016456	1	248,132,090
11396	A_44_P284753	1.043	0.073	2.1E-16	0.611	0.037	1.5E-18	Ankrd1	ENSRNOG00000018598	1	254,735,548
27529	A_43_P12437	-0.597	0.112	5.4E-06	-0.309	0.056	3.1E-06	Hhex	ENSRNOG00000016595	1	256,101,903
393	A_64_P082037	0.475	0.096	1.7E-05	0.278	0.048	1.2E-06	LOC679127	ENSRNOG00000048495	1	261,140,389
9329	A_44_P262593	2.500	0.306	9.9E-10	1.906	0.153	1.2E-14	Ankrd2	ENSRNOG00000013840	1	261,281,543
7394	A_42_P644278	-0.883	0.094	2.9E-11	-0.804	0.047	5.9E-19	Bloc1s2	ENSRNOG00000012684	1	263,920,962
25205	A_44_P325439	0.486	0.086	2.0E-06	0.369	0.043	3.1E-10	Fbxw4	ENSRNOG00000046211	1	265,420,503
1478	A_42_P759043	1.033	0.071	1.1E-16	1.050	0.035	6.1E-27	RGD1311783	ENSRNOG00000020076	1	266,451,021
27106	A_44_P995874	0.365	0.070	7.7E-06	0.488	0.035	4.2E-16	LOC100364597	ENSRNOG00000020179	1	266,782,910
2102	A_44_P990043	0.585	0.095	4.4E-07	0.396	0.048	6.4E-10	Pdcd11	ENSRNOG00000020304	1	266,866,931
2336	A_42_P462015	-0.579	0.093	3.3E-07	-0.237	0.046	1.0E-05	Obfc1	ENSRNOG00000020376	1	267,315,729
2768	A_42_P739860	0.662	0.141	3.9E-05	1.420	0.071	3.3E-21	Dusp5	ENSRNOG00000014061	1	274,245,184
22292	A_64_P026191	0.581	0.114	1.0E-05	0.289	0.057	1.1E-05	Nrap	ENSRNOG00000016714	1	277,181,414
20097	A_44_P183488	-0.483	0.067	1.5E-08	-0.164	0.033	1.9E-05	Casp7	ENSRNOG00000056216	1	277,190,964
15734	A_44_P403005	-0.769	0.100	3.8E-09	-0.599	0.050	3.6E-14	Trub1	ENSRNOG00000017321	1	278,311,244
17131	A_64_P088836	0.464	0.101	4.8E-05	-0.957	0.050	2.0E-20	Gpr98	ENSRNOG00000016306	2	8,939,686
528	A_42_P523612	-0.480	0.102	3.9E-05	-0.331	0.051	1.6E-07	Mef2c	ENSRNOG00000033134	2	11,658,568
14818	A_44_P337311	1.452	0.198	1.2E-08	2.251	0.099	5.5E-23	Thbs4	ENSRNOG00000012471	2	22,385,855
19077	A_44_P131470	0.471	0.065	1.7E-08	0.526	0.033	4.2E-18	Hexb	ENSRNOG00000025274	2	28,003,186
15807	A_64_P068038	0.508	0.101	1.4E-05	0.632	0.051	1.1E-14	Hexb	ENSRNOG00000025274	2	28,003,186
9172	A_43_P10887	0.969	0.151	2.0E-07	1.014	0.076	1.4E-15	Map1b	ENSRNOG00000017428	2	29,768,750

Disease Models & Mechanisms • Supplementary information

28802	A_42_P705481	0.584	0.097	6.3E-07	0.467	0.048	1.6E-11	Il6st	ENSRNOG00000013963	2	44,289,393
2831	A_42_P663871	-1.598	0.177	8.0E-11	-0.396	0.088	7.0E-05	Gzma	ENSRNOG00000010603	2	44,981,458
18451	A_43_P12257	1.200	0.223	4.7E-06	1.350	0.112	2.8E-14	Esm1	ENSRNOG00000010797	2	45,104,305
24749	A_43_P16449	0.568	0.070	9.7E-10	0.479	0.035	6.0E-16	Pelo	ENSRNOG00000061128	2	47,268,625
5239	A_42_P463754	0.704	0.087	1.2E-09	0.414	0.044	2.1E-11	Itga1	ENSRNOG00000053550	2	47,281,421
16351	A_43_P14915	-0.731	0.108	6.7E-08	-0.445	0.054	8.4E-10	C6	ENSRNOG00000024115	2	54,466,280
11752	A_64_P087930	-0.537	0.108	1.6E-05	-0.486	0.054	9.2E-11	C7	ENSRNOG00000061379	2	54,777,729
8100	A_44_P168265	0.721	0.137	7.0E-06	0.633	0.069	5.1E-11	Prkaa1	ENSRNOG00000012799	2	54,857,688
10271	A_44_P421887	0.606	0.134	6.4E-05	0.523	0.067	3.0E-09	Ankrd33b	ENSRNOG00000010888	2	84,436,912
2038	A_43_P10785	-0.431	0.096	7.4E-05	-0.650	0.048	1.1E-15	Cmbl	ENSRNOG00000011260	2	84,645,084
8628	A_64_P048755	-1.291	0.127	3.5E-12	-0.723	0.063	1.5E-13	LOC679974	ENSRNOG00000046390	2	102,370,757
10154	A_64_P092201	0.429	0.097	8.5E-05	0.416	0.048	3.1E-10	Cp	ENSRNOG00000011913	2	104,744,461
14034	A_44_P468304	-1.255	0.276	6.4E-05	-0.801	0.143	2.8E-06	Terc	ENSRNOG00000051777	2	116,432,691
17597	A_43_P23319	0.657	0.123	4.9E-06	0.286	0.061	4.1E-05	Usp13	ENSRNOG00000030639	2	119,197,239
10605	A_42_P462239	0.405	0.060	5.9E-08	0.347	0.030	8.6E-14	Acad9	ENSRNOG00000014178	2	122,782,060
29381	A_44_P472994	0.585	0.075	3.1E-09	0.600	0.038	5.8E-18	Anxa5	ENSRNOG00000014453	2	123,193,130
27496	A_44_P424218	-0.841	0.087	1.3E-11	-0.523	0.043	3.0E-14	Mlf1	ENSRNOG00000012827	2	164,549,455
26163	A_43_P12345	-0.489	0.101	2.6E-05	-0.608	0.051	3.7E-14	Bche	ENSRNOG00000009826	2	171,196,395
25283	A_64_P050994	1.004	0.172	1.2E-06	1.161	0.087	2.9E-15	Sema4a	ENSRNOG00000019737	2	187,817,578
29294	A_44_P191285	0.932	0.160	1.2E-06	0.708	0.080	1.5E-10	RGD1564171	ENSRNOG00000037406	2	189,413,966
24202	A_64_P053307	1.255	0.160	1.5E-08	0.990	0.103	2.3E-10	Nup210l	ENSRNOG00000055790	2	189,454,340
299	A_42_P524308	-0.416	0.065	1.8E-07	-0.198	0.032	4.6E-07	Jtb	ENSRNOG00000016379	2	189,591,780
6842	A_42_P516658	-0.715	0.100	2.2E-08	-0.284	0.050	1.9E-06	Gatad2b	ENSRNOG00000015553	2	189,655,702
14525	A_64_P038452	0.358	0.068	6.4E-06	0.453	0.034	1.6E-15	Cdc42se1	ENSRNOG00000060538	2	196,402,837
10680	A_64_P140977	-0.978	0.100	9.9E-12	-0.962	0.050	1.3E-20	Prune	ENSRNOG00000021120	2	196,456,481
27712	A_64_P041307	-0.589	0.084	3.0E-08	-0.791	0.042	2.7E-20	Tars2	ENSRNOG00000057194	2	197,878,142
28265	A_44_P1033661	0.303	0.065	4.2E-05	0.249	0.032	4.3E-09	Prpf3	ENSRNOG00000025629	2	197,971,456
28118	A_64_P152753	-2.711	0.267	1.0E-11	-1.279	0.145	3.1E-10	RGD1566380	ENSRNOG00000042717	2	197,991,198
4465	A_44_P520475	0.735	0.152	2.4E-05	0.983	0.076	3.7E-15	Anp32e	ENSRNOG00000021168	2	198,040,536
8030	A_44_P393822	0.695	0.093	8.4E-09	0.804	0.047	5.1E-19	Plekho1	ENSRNOG00000021170	2	198,120,120
4450	A_42_P649672	0.929	0.195	3.1E-05	0.525	0.098	4.7E-06	Sv2a	ENSRNOG00000021182	2	198,321,142
25399	A_64_P102889	0.757	0.086	1.6E-10	0.213	0.043	1.7E-05	Polr3gl	ENSRNOG00000021209	2	198,706,428
20625	A_44_P548426	0.776	0.160	2.6E-05	0.493	0.065	8.6E-09	Ankrd35	ENSRNOG00000025108	2	198,797,159

Disease Models & Mechanisms • Supplementary information

20853	A_64_P076891	-1.724	0.133	3.6E-15	-1.778	0.066	2.0E-25	RGD1565469	ENSRNOG00000037217	2	198,834,925
21010	A_44_P513428	0.626	0.139	6.7E-05	0.627	0.069	8.6E-11	Polr3c	ENSRNOG00000033560	2	198,852,161
7498	A_44_P386936	0.395	0.063	2.7E-07	0.463	0.031	7.2E-17	Gpr89	ENSRNOG00000000095	2	199,038,702
20777	A_44_P544023	-0.367	0.081	6.5E-05	0.203	0.041	1.6E-05	Tuba1c	ENSRNOG00000015544	2	199,199,845
12118	A_64_P040804	0.329	0.054	4.7E-07	0.357	0.027	1.8E-15	Rpl38	ENSRNOG00000030747	2	199,479,309
18650	A_44_P1059229	-0.667	0.096	3.4E-08	-0.525	0.048	4.6E-13	Slc22a15	ENSRNOG00000033051	2	204,341,676
8432	A_64_P074102	-1.121	0.175	2.3E-07	-0.472	0.081	1.3E-06	Wnt2b	ENSRNOG00000014385	2	207,455,258
23813	A_43_P15024	0.734	0.162	6.3E-05	1.695	0.081	8.8E-22	Sypl2	ENSRNOG00000019780	2	211,017,778
19556	A_44_P822283	-1.341	0.081	1.9E-18	-0.299	0.041	1.1E-08	Sort1	ENSRNOG00000031814	2	211,078,334
20758	A_43_P11683	0.864	0.120	1.8E-08	0.429	0.060	2.1E-08	F3	ENSRNOG00000011800	2	225,310,624
2551	A_43_P11570	0.901	0.116	3.2E-09	0.570	0.058	9.5E-12	Abcd3	ENSRNOG00000011929	2	225,389,120
2707	A_64_P164926	0.410	0.069	7.2E-07	0.313	0.034	6.2E-11	LOC691931	ENSRNOG00000010875	2	232,245,319
8355	A_64_P151756	1.201	0.220	4.2E-06	1.295	0.090	4.1E-16	LOC679958	ENSRNOG00000023404	2	240,495,371
12459	A_64_P161185	0.615	0.090	5.6E-08	0.369	0.045	9.7E-10	Odf2l	ENSRNOG00000014059	2	250,995,517
8693	A_44_P1020608	-0.556	0.102	3.9E-06	-0.486	0.051	2.3E-11	Ifi44	ENSRNOG00000022218	2	256,915,569
6564	A_43_P11426	0.354	0.078	5.8E-05	0.538	0.039	5.0E-16	Pigk	ENSRNOG00000042359	2	257,911,126
20957	A_43_P23049	-1.359	0.101	1.3E-15	-1.223	0.051	6.3E-24	Cryz	ENSRNOG00000028319	2	260,884,337
1123	A_42_P558445	-0.288	0.066	9.6E-05	-0.193	0.033	1.0E-06	Surf1	ENSRNOG00000060005	3	5,483,239
26230	A_44_P213133	-2.126	0.078	1.3E-25	-2.025	0.039	1.8E-35	Endog	ENSRNOG00000016033	3	8,741,766
17007	A_64_P129901	-1.051	0.110	2.1E-11	-1.058	0.055	1.5E-20	Ccbl1	ENSRNOG00000016097	3	8,766,433
12417	A_64_P050722	0.304	0.068	7.4E-05	0.310	0.034	6.2E-11		ENSRNOG00000031434	3	8,955,538
22393	A_43_P18366	1.926	0.108	1.6E-19	2.056	0.054	8.3E-31	Tor1b	ENSRNOG00000006435	3	9,792,899
16409	A_44_P447373	1.253	0.280	7.9E-05	1.054	0.143	1.2E-08	Ass1	ENSRNOG00000008837	3	10,375,826
3868	A_64_P101908	0.719	0.129	2.6E-06	0.564	0.065	2.0E-10	LOC688582	ENSRNOG00000062252	3	10,408,115
16058	A_44_P1012226	-0.666	0.086	3.7E-09	-0.328	0.043	5.1E-09	Pomt1	ENSRNOG00000010477	3	11,254,026
2282	A_42_P553960	-0.404	0.060	7.4E-08	-0.366	0.030	2.3E-14	Uck1	ENSRNOG00000011467	3	11,277,757
29474	A_44_P532754	-0.506	0.094	4.8E-06	-0.333	0.047	2.7E-08	Pbx3	ENSRNOG00000022162	3	13,435,979
28010	A_64_P038074	0.888	0.154	1.4E-06	0.427	0.077	2.7E-06	Lypd6b	ENSRNOG00000004614	3	35,271,786
23123	A_64_P045400	-1.027	0.120	3.1E-10	-0.824	0.060	6.1E-16	Gca	ENSRNOG00000007359	3	48,626,038
16975	A_44_P960805	-0.584	0.094	3.7E-07	-0.848	0.047	1.2E-19	Gca	ENSRNOG00000007359	3	48,626,038
658	A_44_P231223	0.651	0.112	1.1E-06	0.748	0.056	1.3E-15	Xirp2	ENSRNOG00000034258	3	53,563,194
14170	A_64_P141349	1.294	0.207	2.7E-06	1.248	0.134	4.0E-09	B3galt1	ENSRNOG00000007625	3	54,253,949
19663	A_64_P084088	0.463	0.083	2.5E-06	0.318	0.041	4.5E-09	Cybrd1	ENSRNOG00000009620	3	57,718,364

Disease Models & Mechanisms • Supplementary information

14032	A_64_P134564	0.300	0.052	1.6E-06	0.326	0.026	1.2E-14	Dync1i2	ENSRNOG00000009781	3	57,817,693
1340	A_44_P653272	0.645	0.092	2.9E-08	0.587	0.046	5.2E-15	Gpr155	ENSRNOG00000018485	3	60,105,212
15603	A_44_P464145	0.782	0.140	2.6E-06	0.855	0.070	2.4E-14	Gpr155	ENSRNOG00000018485	3	60,105,212
24139	A_44_P992697	-1.507	0.098	1.8E-17	-1.179	0.049	7.0E-24	Dusp19	ENSRNOG00000008868	3	67,849,966
30134	A_64_P048824	0.364	0.079	4.9E-05	0.207	0.040	7.3E-06	Zc3h15	ENSRNOG00000005256	3	71,020,534
4545	A_44_P1011687	1.427	0.115	1.3E-14	1.194	0.057	1.1E-21	Tfpi	ENSRNOG00000005039	3	71,893,600
25328	A_64_P080025	0.504	0.091	3.1E-06	0.374	0.046	9.4E-10	Serping1	ENSRNOG00000007457	3	72,171,078
1516	A_42_P561644	-0.494	0.071	4.0E-08	-0.425	0.036	4.5E-14	Timm10	ENSRNOG00000007883	3	72,226,613
8277	A_64_P022076	0.910	0.163	2.5E-06	0.405	0.082	1.7E-05	Slc43a3	ENSRNOG00000061768	3	72,329,967
17375	A_43_P15275	-1.626	0.089	8.4E-20	-1.083	0.045	5.8E-24	Ptprj	ENSRNOG00000034025	3	79,390,956
18771	A_44_P107766	-0.740	0.074	6.2E-12	-0.445	0.037	3.6E-14	RGD1309540	ENSRNOG00000014798	3	80,349,145
10251	A_64_P045671	1.113	0.164	5.8E-08	0.520	0.082	2.3E-07	Mdk	ENSRNOG00000017560	3	80,842,916
18605	A_64_P126818	-0.534	0.111	2.7E-05	-0.652	0.056	7.0E-14	Tspan18	ENSRNOG00000008758	3	82,236,664
25223	A_64_P115142	-0.673	0.095	2.4E-08	-0.776	0.047	2.7E-18	Accs	ENSRNOG00000009199	3	82,756,953
15888	A_64_P164232	-0.589	0.098	7.3E-07	-0.366	0.049	8.6E-09	RGD1306717	ENSRNOG00000008663	3	93,698,121
2959	A_64_P135773	1.850	0.177	1.8E-12	2.045	0.089	3.0E-23	Prrg4	ENSRNOG00000022710	3	94,808,861
29107	A_64_P033322	0.483	0.087	2.8E-06	0.386	0.044	1.4E-10	Lin7c	ENSRNOG00000045698	3	101,010,899
18122	A_44_P852315	0.596	0.095	2.9E-07	0.658	0.047	4.8E-16	Fibin	ENSRNOG00000004699	3	101,547,478
10565	A_64_P078914	0.831	0.176	3.4E-05	0.975	0.088	3.5E-13	Phgr1	ENSRNOG00000060434	3	110,508,403
16898	A_42_P527070	-0.356	0.080	8.0E-05	-0.185	0.040	4.8E-05	Gchfr	ENSRNOG00000012290	3	110,975,923
3204	A_42_P618145	0.829	0.081	3.6E-12	0.724	0.041	1.8E-19	Hisppd2a	ENSRNOG00000014436	3	113,312,127
20811	A_64_P156094	0.812	0.153	6.0E-06	0.943	0.077	1.7E-14	Hisppd2a	ENSRNOG00000014436	3	113,312,127
25901	A_44_P714860	0.325	0.070	4.4E-05	0.447	0.035	5.8E-15	Serf2	ENSRNOG00000015277	3	113,415,774
15624	A_43_P19265	0.346	0.076	6.2E-05	0.200	0.038	7.3E-06	Shf	ENSRNOG00000028685	3	114,307,250
17734	A_44_P122372	0.762	0.103	8.6E-09	0.717	0.051	3.6E-16	Pldn	ENSRNOG00000037160	3	114,869,459
5981	A_64_P111786	-0.678	0.084	1.2E-09	-0.673	0.042	4.6E-18	Sqrdl	ENSRNOG00000000172	3	114,900,343
26669	A_64_P100963	-0.941	0.155	5.5E-07	-0.392	0.077	1.3E-05	Ncaph	ENSRNOG00000012051	3	119,611,136
834	A_42_P561329	-0.668	0.096	3.4E-08	-0.494	0.048	2.5E-12	Anapc1	ENSRNOG00000016965	3	121,226,125
19676	A_64_P098527	-0.415	0.077	4.9E-06	-0.275	0.039	2.3E-08	Chchd5	ENSRNOG00000018491	3	121,660,110
29825	A_64_P000678	0.453	0.103	9.5E-05	0.247	0.052	2.9E-05	Sirpa	ENSRNOG00000004763	3	122,114,754
18410	A_64_P143459	-0.740	0.112	1.0E-07	-0.843	0.056	3.5E-17	Snrpb	ENSRNOG00000006961	3	122,703,705
5717	A_64_P024798	-0.904	0.084	7.8E-13	-0.258	0.042	4.2E-07	Ubox5	ENSRNOG00000021230	3	123,171,875
1564	A_42_P501233	-1.515	0.210	1.6E-08	-2.744	0.105	4.2E-25	Adra1d	ENSRNOG00000021256	3	124,145,566

Disease Models & Mechanisms • Supplementary information

27875	A_64_P015227	0.536	0.113	3.4E-05	0.428	0.057	6.0E-09	Trmt6	ENSRNOG00000021270	3	125,470,413
25424	A_44_P378742	-1.021	0.153	8.2E-08	-0.677	0.076	1.3E-10	Flrt3	ENSRNOG00000004874	3	134,696,654
13519	A_44_P370537	0.692	0.135	1.0E-05	0.653	0.067	1.4E-11	Polr3f	ENSRNOG00000007548	3	138,684,685
28840	A_44_P243599	-0.787	0.126	3.3E-07	-0.569	0.063	8.6E-11	RGD1566320	ENSRNOG00000036971	3	138,765,027
1909	A_43_P12825	-0.477	0.096	1.7E-05	-0.608	0.048	7.8E-15	Entpd6	ENSRNOG00000007427	3	146,546,387
8320	A_64_P144278	0.489	0.107	5.5E-05	0.499	0.053	3.6E-11	Snph	ENSRNOG00000009588	3	147,143,576
12668	A_44_P363204	-0.643	0.109	9.9E-07	-0.609	0.055	3.1E-13	Csnk2a1	ENSRNOG00000005276	3	147,713,821
24270	A_64_P117901	1.828	0.237	4.8E-09	1.823	0.110	3.4E-18	Trib3	ENSRNOG00000007319	3	147,819,571
18709	A_44_P305135	0.295	0.059	1.6E-05	0.464	0.030	1.2E-17	Epb41l1	ENSRNOG00000057817	3	152,552,822
2734	A_42_P777935	0.454	0.097	3.8E-05	0.506	0.048	1.9E-12	RGD1311066	ENSRNOG00000020086	3	152,626,757
19643	A_44_P761309	0.720	0.083	2.5E-10	0.479	0.042	1.2E-13	Lpin3	ENSRNOG00000016636	3	156,886,454
5599	A_43_P18817	-0.921	0.123	8.0E-09	-1.006	0.062	3.0E-18	Ift52	ENSRNOG00000007692	3	159,392,193
608	A_42_P629321	0.853	0.191	7.5E-05	0.721	0.095	6.0E-09	Wisp2	ENSRNOG00000010666	3	160,207,913
18760	A_64_P037510	0.324	0.058	2.7E-06	0.299	0.029	3.0E-12	Rpl38	ENSRNOG00000048701	3	164,002,482
20958	A_64_P002904	0.566	0.094	6.5E-07	0.689	0.047	8.9E-17	Pmepa1	ENSRNOG00000050404	3	171,342,646
16812	A_64_P058749	0.909	0.080	1.6E-13	0.580	0.040	1.1E-16		ENSRNOG00000043150	3	176,479,335
15543	A_44_P307964	-0.653	0.079	6.8E-10	-0.585	0.039	5.6E-17	Polr3k	ENSRNOG00000017843	3	177,374,812
24680	A_43_P12222	-0.349	0.068	9.2E-06	-0.287	0.034	4.1E-10	Pmpcb	ENSRNOG00000012693	4	9,921,610
4023	A_64_P148535	0.580	0.113	1.0E-05	0.386	0.057	5.7E-08	Napepld	ENSRNOG00000011363	4	9,966,891
18208	A_64_P166489	0.471	0.108	9.9E-05	0.383	0.054	2.2E-08	Napepld	ENSRNOG00000011363	4	9,981,958
21357	A_64_P016751	-0.826	0.090	5.2E-11	-1.041	0.045	2.7E-23	Tmem243 (RGD1	ENSRNOG00000042758	4	21,920,651
9693	A_64_P039888	2.025	0.157	6.3E-15	1.703	0.080	1.0E-21	Abcb1b	ENSRNOG00000008012	4	22,307,453
17357	A_64_P095855	1.345	0.255	6.6E-06	0.715	0.128	2.3E-06	Rundc3b	ENSRNOG00000008463	4	22,445,414
5089	A_64_P113443	0.642	0.102	2.6E-07	0.295	0.051	1.3E-06	Cdk6	ENSRNOG00000009258	4	27,966,398
5850	A_44_P139763	0.441	0.068	1.3E-07	0.275	0.034	1.1E-09	Bet1	ENSRNOG00000011008	4	29,092,753
10442	A_44_P899239	-0.434	0.090	2.6E-05	-0.229	0.045	1.2E-05	Samd9l	ENSRNOG00000051922	4	31,229,913
10121	A_44_P338381	-0.904	0.156	1.7E-06	-1.084	0.083	1.3E-14	Cadps2	ENSRNOG00000007636	4	50,860,756
12560	A_44_P494632	0.608	0.118	9.9E-06	0.641	0.059	6.8E-13	Flnc	ENSRNOG00000007281	4	56,711,049
30022	A_44_P260072	0.642	0.133	2.5E-05	0.763	0.066	1.3E-13	Tbxas1	ENSRNOG00000007918	4	66,670,618
29522	A_44_P489995	-0.574	0.063	7.8E-11	-0.287	0.032	7.7E-11	Mrps33	ENSRNOG00000026528	4	67,610,710
2447	A_64_P002506	1.093	0.176	5.3E-07	1.590	0.098	2.3E-17		ENSRNOG00000005506	4	72,718,458
448	A_44_P930917	2.148	0.175	4.4E-14	1.997	0.071	3.9E-25	Arhgef5	ENSRNOG00000005506	4	72,718,458
13843	A_44_P990376	1.171	0.193	5.4E-07	0.740	0.096	4.2E-09	Gpnmb	ENSRNOG00000008816	4	78,694,447

4571	A_64_P044087	1.240	0.201	4.3E-07	0.922	0.101	6.1E-11	Nap1l5	ENSRNOG00000007808	4	89,151,184
21325	A_64_P152919	-0.565	0.096	8.9E-07	-0.295	0.048	4.0E-07		ENSRNOG00000023657	4	89,695,928
17936	A_64_P159902	-0.584	0.106	2.9E-06	-0.385	0.053	1.4E-08	Mmrn1	ENSRNOG00000024986	4	90,990,088
24747	A_64_P157405	-0.598	0.086	3.6E-08	-0.722	0.043	1.1E-18	Reep1	ENSRNOG00000008481	4	99,618,622
19570	A_64_P027404	-0.522	0.118	8.5E-05	-0.625	0.059	1.2E-12	Mat2a	ENSRNOG00000013520	4	100,303,080
20592	A_44_P402507	-1.909	0.082	2.4E-23	-2.219	0.041	3.4E-36	Retsat	ENSRNOG00000014090	4	100,465,170
8298	A_42_P693964	1.689	0.189	1.1E-10	0.598	0.095	2.6E-07	Cyp26b1	ENSRNOG00000015076	4	116,278,615
27845	A_44_P200846	-0.454	0.103	9.0E-05	-0.421	0.052	9.6E-10	Sfxn5	ENSRNOG00000037871	4	117,111,653
25583	A_64_P128971	0.684	0.132	8.2E-06	0.381	0.066	1.3E-06	Chst13	ENSRNOG00000025930	4	122,237,754
18655	A_64_P011674	2.176	0.225	1.5E-11	2.275	0.113	2.8E-21	Shq1	ENSRNOG00000005433	4	133,131,953
1140	A_64_P156263	0.491	0.087	2.2E-06	0.714	0.044	2.8E-18	Lmcd1	ENSRNOG00000005690	4	144,192,989
13196	A_44_P622992	-0.794	0.093	3.0E-10	-0.635	0.046	6.5E-16	Tuba8	ENSRNOG00000048169	4	153,774,486
14600	A_64_P157059	-0.796	0.159	1.4E-05	-0.403	0.079	1.1E-05	Usp18	ENSRNOG00000037198	4	153,805,993
16017	A_64_P038107	0.637	0.129	1.8E-05	0.450	0.064	3.4E-08	Apobec1	ENSRNOG00000015411	4	155,401,480
19984	A_64_P166460	1.400	0.210	1.1E-07	0.810	0.109	1.2E-08	C3ar1	ENSRNOG00000009211	4	155,690,869
6653	A_64_P101301	0.684	0.142	2.5E-05	0.574	0.071	1.2E-09	Clec4a3	ENSRNOG00000010018	4	155,923,079
12718	A_43_P17428	-0.443	0.074	6.3E-07	-0.260	0.037	2.7E-08	Lpcat3	ENSRNOG00000012269	4	157,181,795
5930	A_64_P026222	1.538	0.250	6.2E-07	1.751	0.104	8.3E-18	LOC689757	ENSRNOG00000037076	4	162,230,859
10286	A_64_P012119	1.184	0.207	2.4E-06	1.309	0.104	5.7E-14	Gprc5d	ENSRNOG00000008439	4	168,884,886
19079	A_64_P023431	0.784	0.120	1.2E-07	0.842	0.060	3.0E-16	Hebp1	ENSRNOG00000000024	4	168,933,079
19656	A_64_P113645	1.679	0.229	3.4E-08	2.197	0.134	1.6E-16	RGD1561551	ENSRNOG00000036959	4	177,039,677
26918	A_44_P372998	1.994	0.145	6.4E-16	0.980	0.073	1.1E-15	Cpa6	ENSRNOG00000005655	5	8,459,660
826	A_43_P20387	0.617	0.136	5.9E-05	0.305	0.068	7.0E-05	Arfgef1	ENSRNOG00000005703	5	8,666,200
15292	A_43_P14372	-1.136	0.075	3.0E-17	-1.266	0.037	5.7E-29		ENSRNOG00000031769	5	16,845,631
27282	A_64_P000859	0.413	0.094	8.9E-05	0.391	0.047	6.0E-10	Plekhf2	ENSRNOG00000026662	5	24,255,606
10504	A_44_P532297	-0.597	0.067	1.1E-10	-0.378	0.033	1.9E-13	RGD1309085 (NI	ENSRNOG00000040040	5	24,320,786
4766	A_44_P775787	-0.808	0.106	5.3E-09	-0.316	0.053	8.2E-07	LOC500420	ENSRNOG00000028185	5	35,902,262
17136	A_64_P067968	-0.703	0.116	6.1E-07	-1.053	0.058	1.0E-19	RGD1562865	ENSRNOG00000006170	5	47,546,014
15347	A_64_P104850	-0.334	0.068	2.0E-05	-0.429	0.034	8.8E-15	Lyrm2	ENSRNOG00000043105	5	48,047,913
9389	A_44_P1041460	-1.359	0.149	6.4E-11	-1.549	0.074	1.0E-21	Rragd	ENSRNOG00000007331	5	48,224,994
9581	A_44_P715035	0.348	0.071	1.9E-05	0.260	0.035	1.1E-08	RGD1359108	ENSRNOG00000009478	5	50,684,409
23043	A_43_P18183	0.559	0.125	7.3E-05	0.429	0.063	7.5E-08	RGD1359108	ENSRNOG00000009478	5	50,684,409
20705	A_44_P576954	-0.757	0.112	6.7E-08	-0.662	0.056	5.5E-14	Ddx58	ENSRNOG00000006384	5	56,536,772

Disease Models & Mechanisms • Supplementary information

10900	A_42_P672071	0.734	0.121	5.7E-07	0.553	0.061	6.3E-11	Ccl19	ENSRNOG00000015668	5	58,183,017
29008	A_64_P144482	1.761	0.144	2.3E-14	1.380	0.072	1.8E-20		ENSRNOG00000016731	5	59,025,631
321	A_42_P698240	-0.854	0.153	2.6E-06	-1.305	0.077	7.7E-19	Ptgr1	ENSRNOG00000015072	5	76,129,441
13215	A_43_P15212	-0.373	0.079	3.5E-05	-0.178	0.040	6.5E-05	Pole3	ENSRNOG00000055277	5	78,384,047
9363	A_44_P146845	1.255	0.257	2.1E-05	1.818	0.128	2.5E-16	Orm1	ENSRNOG00000007886	5	79,179,417
13242	A_44_P1040796	1.265	0.116	6.1E-13	1.286	0.058	1.4E-22	Atp6v1g1	ENSRNOG00000008163	5	79,367,663
2972	A_42_P832422	0.677	0.130	8.4E-06	0.643	0.065	8.7E-12	Mllt3	ENSRNOG00000011280	5	106,207,126
3813	A_44_P1008463	0.508	0.067	5.3E-09	0.544	0.033	3.1E-18	RGD1311849	ENSRNOG00000024402	5	106,409,456
946	A_44_P777439	-0.377	0.077	2.0E-05	-0.460	0.038	4.1E-14	Hook1	ENSRNOG00000026226	5	114,940,053
18791	A_64_P000171	0.526	0.069	4.3E-09	0.671	0.034	7.8E-21		ENSRNOG00000006787	5	126,164,674
28037	A_43_P10410	0.376	0.079	3.1E-05	0.400	0.040	4.3E-12	Txndc12	ENSRNOG00000008090	5	128,450,680
15449	A_64_P071918	-0.652	0.090	1.5E-08	-0.497	0.045	3.7E-13	Nsun4	ENSRNOG00000012020	5	134,382,794
12837	A_44_P558028	0.763	0.142	4.8E-06	0.606	0.071	3.5E-10	Ccdc17	ENSRNOG00000023216	5	135,442,031
9670	A_44_P117119	-0.720	0.108	8.1E-08	-0.422	0.054	2.5E-09	Hpdl	ENSRNOG00000018143	5	135,677,432
20248	A_64_P138415	2.267	0.139	3.3E-16	2.602	0.087	2.3E-23	Cldn19	ENSRNOG00000007922	5	138,300,107
955	A_64_P032604	-0.881	0.082	9.7E-13	-0.746	0.041	1.0E-19	Ak2	ENSRNOG00000000122	5	147,185,474
3334	A_64_P097842	-0.582	0.088	1.2E-07	-0.445	0.044	5.2E-12	Marcksl1	ENSRNOG00000009113	5	147,714,163
10388	A_64_P035962	-0.906	0.126	1.8E-08	-1.000	0.063	7.2E-18	LOC682360	ENSRNOG00000049425	5	147,784,311
22059	A_43_P17437	0.327	0.065	1.4E-05	0.302	0.033	4.5E-11	Taf12	ENSRNOG00000048288	5	150,459,713
23745	A_44_P175875	0.799	0.086	4.2E-11	0.548	0.043	6.2E-15	Pigv	ENSRNOG00000000121	5	151,895,016
5655	A_43_P11466	0.518	0.064	1.3E-09	0.439	0.032	6.9E-16	Fuca1	ENSRNOG00000009325	5	154,269,118
982	A_42_P636963	-0.481	0.083	1.4E-06	-0.375	0.042	9.5E-11	Zfp46	ENSRNOG00000049975	5	154,650,632
3505	A_42_P703647	-0.515	0.112	4.9E-05	-0.625	0.056	2.7E-13	Cda	ENSRNOG00000015677	5	156,734,541
15453	A_64_P048668	-0.745	0.108	3.9E-07	-1.037	0.091	3.6E-11	Pla2g2c	ENSRNOG00000016647	5	157,165,341
15824	A_44_P549075	-0.505	0.059	3.1E-10	-0.365	0.029	1.4E-14	Pla2g5	ENSRNOG00000016838	5	157,268,903
3322	A_42_P569711	0.641	0.100	2.1E-07	1.015	0.050	2.7E-21	Epha2	ENSRNOG00000009222	5	159,845,774
1445	A_64_P029878	-0.856	0.073	6.5E-14	-0.686	0.036	2.7E-20	Clcnkb	ENSRNOG00000009897	5	159,962,676
1979	A_42_P638494	1.146	0.131	1.9E-10	1.343	0.066	1.7E-21	Nppb	ENSRNOG00000008141	5	164,796,185
1363	A_44_P317600	2.413	0.233	2.3E-12	1.277	0.116	4.7E-13	Nppa	ENSRNOG00000008176	5	164,808,323
23292	A_64_P075910	1.060	0.142	7.9E-09	0.324	0.071	5.7E-05	Ajap1	ENSRNOG00000050137	5	170,679,315
21962	A_44_P100870	-0.379	0.077	2.0E-05	-0.186	0.039	2.5E-05	RGD1308251	ENSRNOG00000013468	5	172,307,431
28765	A_64_P038692	-0.323	0.067	2.4E-05	-0.371	0.033	3.1E-13	Cox7a2l	ENSRNOG00000004526	6	6,709,783
14904	A_44_P328717	-0.912	0.123	8.6E-09	-0.810	0.061	2.1E-15	Mtm1	ENSRNOG00000002516	6	18,821,815

26647	A_43_P11724	1.432	0.120	4.4E-14	0.658	0.060	4.9E-13	Gckr	ENSRNOG00000048874	6	26,385,761
18372	A_64_P017363	0.596	0.103	1.3E-06	0.635	0.051	1.6E-14	Slc30a3	ENSRNOG00000006204	6	26,642,783
24275	A_44_P413979	0.650	0.075	2.8E-10	0.797	0.038	6.1E-22	Rab10	ENSRNOG00000047088	6	27,721,120
4161	A_64_P076450	0.483	0.088	3.6E-06	0.475	0.044	8.3E-13	Pomc	ENSRNOG00000012686	6	28,382,962
28085	A_64_P108486	0.731	0.137	5.0E-06	0.729	0.068	1.0E-12	Dnajc27	ENSRNOG00000003988	6	28,515,025
7900	A_64_P155756	1.212	0.221	3.3E-06	0.673	0.110	5.2E-07	Tpo	ENSRNOG00000004646	6	49,089,855
16459	A_44_P222786	-0.442	0.054	9.6E-10	-0.513	0.027	2.2E-20	RGD1563296	ENSRNOG00000056610	6	50,954,631
12252	A_64_P145433	0.747	0.108	3.9E-08	0.404	0.054	7.0E-09		ENSRNOG00000004342	6	55,001,464
19783	A_64_P127402	-0.414	0.075	3.2E-06	-0.566	0.038	3.8E-17	Bzw2	ENSRNOG00000005096	6	55,647,665
1431	A_42_P600642	-0.606	0.106	1.7E-06	-0.534	0.053	5.3E-12	Ispd	ENSRNOG00000006199	6	55,881,484
3423	A_44_P545553	-0.981	0.120	9.2E-10	-1.400	0.060	2.0E-23	Dgkb	ENSRNOG00000030771	6	57,516,713
4107	A_44_P211061	1.163	0.135	2.9E-10	1.179	0.068	3.6E-19	Etv1	ENSRNOG00000006867	6	58,467,254
6710	A_64_P008628	-0.386	0.082	3.5E-05	-0.336	0.041	8.3E-10	Ndufa10l1	ENSRNOG00000062245	6	62,798,384
1845	A_64_P013182	0.696	0.111	3.2E-07	0.640	0.056	1.3E-13	Stxbp6	ENSRNOG00000004198	6	65,319,527
17692	A_64_P124599	-0.685	0.092	8.9E-09	-0.508	0.046	4.1E-13		ENSRNOG00000004165	6	71,199,110
23405	A_64_P045378	-0.609	0.120	1.2E-05	-0.959	0.060	5.7E-18		ENSRNOG00000056721	6	86,852,323
1503	A_42_P789482	0.765	0.104	1.2E-08	0.451	0.052	2.5E-10	Daam1	ENSRNOG00000004345	6	94,636,222
30010	A_43_P19862	-1.264	0.202	3.1E-07	-1.115	0.101	3.9E-13	Lrrc9	ENSRNOG00000005409	6	95,205,153
28307	A_44_P285390	0.422	0.082	8.9E-06	0.282	0.041	4.3E-08	Atp6v1d	ENSRNOG00000009080	6	102,047,758
7901	A_64_P136751	-0.645	0.099	1.5E-07	-0.578	0.050	8.3E-14		ENSRNOG00000008082	6	106,496,992
8745	A_44_P260589	-1.755	0.187	7.4E-11	-1.871	0.100	3.1E-19		ENSRNOG00000009448	6	107,245,820
5759	A_64_P065368	1.384	0.204	6.2E-08	1.120	0.102	4.7E-13	Acot4	ENSRNOG00000046864	6	107,517,668
16745	A_64_P067447	2.065	0.312	1.8E-07	2.020	0.100	8.6E-20	Acot4	ENSRNOG00000046864	6	107,517,668
18088	A_44_P337991	0.724	0.107	6.6E-08	0.529	0.054	8.1E-12	Acot3	ENSRNOG00000053460	6	107,531,528
20123	A_64_P074370	-1.168	0.109	9.7E-13	-1.231	0.055	7.3E-23	Dnal1	ENSRNOG00000042333	6	107,596,785
29970	A_64_P064381	-0.387	0.072	4.8E-06	-0.609	0.036	9.3E-19	Ptgr2	ENSRNOG00000038166	6	107,999,846
28805	A_64_P028095	-0.315	0.071	8.0E-05	-0.673	0.035	2.0E-20	Abcd4	ENSRNOG00000011964	6	108,329,464
14620	A_64_P124473	1.208	0.187	1.7E-07	1.388	0.094	6.1E-17	Ltbp2	ENSRNOG00000012094	6	108,596,569
28988	A_64_P068847	1.017	0.226	7.3E-05	1.475	0.105	5.2E-16	Ltbp2	ENSRNOG00000012094	6	108,596,569
25368	A_64_P031035	-0.833	0.108	3.9E-09	-0.919	0.054	7.9E-19	Gstz1	ENSRNOG00000047708	6	111,176,798
4908	A_42_P720155	0.669	0.085	2.2E-09	0.245	0.042	1.3E-06	LOC500700	ENSRNOG00000003908	6	114,966,751
804	A_42_P701779	-0.456	0.077	8.3E-07	-0.287	0.038	7.6E-09	Rps6ka5	ENSRNOG00000004362	6	124,735,741
5142	A_43_P12172	0.279	0.059	3.8E-05	0.295	0.030	7.2E-12	Lgmn	ENSRNOG00000007089	6	126,320,726

29887	A_64_P148433	-0.688	0.109	2.7E-07	-0.439	0.055	1.4E-09	Ifi27	ENSRNOG00000009263	6	127,327,959
22152	A_44_P382464	0.488	0.079	4.3E-07	0.544	0.040	6.9E-16	RGD1308470	ENSRNOG00000042427	6	129,519,709
20830	A_44_P185355	-1.231	0.173	2.2E-08	-1.579	0.086	7.7E-20	Bcl11b	ENSRNOG00000005776	6	131,926,272
8534	A_64_P006368	-0.518	0.104	1.5E-05	-0.451	0.052	2.2E-10	RGD1565168	ENSRNOG00000033736	6	135,939,534
5921	A_64_P004007	0.689	0.130	5.8E-06	0.420	0.065	1.6E-07	RGD1305627	ENSRNOG00000043031	6	136,550,371
13859	A_64_P068378	-0.517	0.079	1.3E-07	-0.542	0.039	6.4E-16		ENSRNOG00000028650	6	137,164,535
16943	A_64_P079523	0.696	0.159	9.4E-05	0.860	0.079	6.4E-13	Ahnak2	ENSRNOG00000028545	6	137,353,249
1529	A_42_P742797	-0.581	0.059	7.6E-12	-0.521	0.029	1.9E-19	Brf1	ENSRNOG00000014595	6	137,808,303
15597	A_64_P103580	-1.015	0.151	8.2E-08	-0.695	0.077	1.0E-10	LOC690422	ENSRNOG00000005153	6	137,967,401
11175	A_64_P002455	-0.909	0.110	7.6E-10	-0.868	0.055	8.5E-18	LOC690422	ENSRNOG00000005153	6	137,967,401
13502	A_44_P852545	-0.523	0.116	6.8E-05	-0.451	0.058	3.2E-09	Tmem196	ENSRNOG00000037435	6	147,876,237
12833	A_44_P1015388	-0.396	0.080	1.7E-05	-0.415	0.040	2.1E-12	Ankrd24	ENSRNOG00000006759	7	10,936,415
974	A_42_P842823	0.701	0.097	1.6E-08	0.619	0.049	6.0E-15	Reep6	ENSRNOG00000033262	7	12,246,729
10600	A_64_P099017	-2.230	0.186	3.9E-14	-1.769	0.093	2.1E-20	Cyp4f17	ENSRNOG00000062306	7	14,529,483
2475	A_64_P079666	0.497	0.070	2.5E-08	0.311	0.035	1.4E-10	Cyp4f5	ENSRNOG00000042496	7	14,559,878
15805	A_64_P255450	-0.876	0.095	5.1E-11	-0.915	0.048	1.4E-20	Zfp799	ENSRNOG00000032552	7	15,072,703
22219	A_64_P108737	0.328	0.073	6.6E-05	0.285	0.036	2.8E-09		ENSRNOG00000046020	7	15,386,329
12870	A_64_P146682	-0.796	0.147	4.1E-06	-0.520	0.073	2.5E-08	Pctk2	ENSRNOG00000004148	7	34,001,506
13077	A_44_P1008751	0.510	0.115	8.6E-05	0.663	0.058	1.3E-13	Fgd6	ENSRNOG00000054515	7	34,952,011
27527	A_64_P108742	0.508	0.063	1.5E-09	0.312	0.032	8.4E-12	Ndufa12	ENSRNOG00000007407	7	35,125,424
1345	A_42_P543004	-0.721	0.094	4.3E-09	-0.585	0.047	1.2E-14	Socs2	ENSRNOG00000008965	7	36,499,784
17690	A_44_P216654	0.509	0.092	3.0E-06	0.480	0.046	2.1E-12	Nap1l1	ENSRNOG00000003890	7	54,213,319
29539	A_43_P11911	0.391	0.068	1.4E-06	0.435	0.034	4.9E-15	Rab3ip	ENSRNOG00000005362	7	59,957,232
9888	A_64_P025286	0.742	0.168	8.8E-05	0.832	0.084	7.8E-12	Lyz2	ENSRNOG00000005825	7	60,341,264
1448	A_42_P669907	0.879	0.153	1.5E-06	0.344	0.077	6.9E-05	Slc16a7	ENSRNOG00000007839	7	68,512,397
9948	A_44_P194803	-0.649	0.121	4.9E-06	-0.804	0.060	1.7E-15	Baalc	ENSRNOG00000004697	7	77,763,512
5860	A_64_P047206	0.864	0.187	4.6E-05	0.524	0.093	2.3E-06	Depdc6	ENSRNOG00000004328	7	94,795,214
4282	A_44_P686854	-0.547	0.105	8.4E-06	-0.456	0.053	2.5E-10	RGD1563224	ENSRNOG00000008934	7	98,709,344
18442	A_64_P044345	0.546	0.108	1.3E-05	0.686	0.054	6.9E-15	Plec	ENSRNOG00000023781	7	117,267,803
30277	A_64_P142942	-0.836	0.116	1.7E-08	-0.495	0.058	3.4E-10	Scx	ENSRNOG00000021812	7	117,519,075
12545	A_64_P039737	-0.893	0.081	4.3E-13	-0.800	0.041	6.2E-21	Scx	ENSRNOG00000021812	7	117,519,075
12381	A_64_P110838	0.429	0.093	4.9E-05	0.260	0.047	2.5E-06	Apol3	ENSRNOG00000042771	7	118,685,022
24480	A_44_P286788	0.911	0.187	2.1E-05	0.852	0.093	6.4E-11	Ncf4	ENSRNOG00000006940	7	119,482,272

Disease Models & Mechanisms • Supplementary information

22505	A_42_P809869	0.614	0.127	2.6E-05	0.852	0.064	1.5E-15	Tst	ENSRNOG00000000186	7	119,623,072
28193	A_44_P265709	1.441	0.108	1.8E-15	1.607	0.054	5.8E-27	Il2rb	ENSRNOG00000048636	7	119,712,888
19289	A_44_P975458	-0.745	0.119	3.1E-07	-0.449	0.060	6.2E-09	C1qtnf6	ENSRNOG00000007300	7	119,753,290
25974	A_43_P12557	0.834	0.066	7.9E-15	0.906	0.033	8.3E-26	Polr2f	ENSRNOG00000011214	7	120,380,544
13289	A_42_P620762	0.411	0.085	2.5E-05	0.219	0.043	9.4E-06	Atf4	ENSRNOG00000017801	7	121,480,723
3854	A_42_P633840	-0.815	0.160	1.1E-05	0.406	0.080	1.1E-05	Ttll1	ENSRNOG00000010141	7	124,367,630
8829	A_64_P267897	-2.395	0.133	1.1E-19	0.386	0.066	1.2E-06		ENSRNOG00000011094	7	124,929,025
23584	A_64_P039342	0.470	0.091	9.0E-06	0.333	0.046	1.2E-08	Arhgap8	ENSRNOG00000033570	7	125,795,980
26976	A_64_P065313	0.527	0.089	9.5E-07	0.279	0.045	3.3E-07	RGD1304694	ENSRNOG00000031269	7	125,893,168
16279	A_44_P1034541	-0.571	0.103	2.9E-06	-0.640	0.052	1.3E-14	Pim3	ENSRNOG00000029698	7	129,860,114
2045	A_44_P179787	-1.366	0.113	2.9E-14	-0.857	0.057	3.0E-17	Panx2	ENSRNOG00000055530	7	130,042,947
27247	A_64_P005309	-0.658	0.094	3.3E-08	-0.410	0.047	2.0E-10	Mapk12	ENSRNOG00000031233	7	130,120,579
3165	A_42_P524610	0.539	0.063	2.9E-10	0.573	0.031	7.4E-20	Pphln1	ENSRNOG00000022778	7	134,603,121
25471	A_44_P653647	0.534	0.099	4.6E-06	0.527	0.050	1.2E-12	Tmem117	ENSRNOG00000006068	7	136,182,224
14589	A_44_P152395	1.805	0.142	7.0E-15	1.278	0.071	1.3E-19	Rnd1	ENSRNOG00000059857	7	140,356,209
3857	A_64_P073129	-2.105	0.161	3.1E-15	-1.961	0.081	5.5E-24	Map3k12	ENSRNOG00000015134	7	144,109,116
3769	A_43_P12287	-0.508	0.060	3.4E-10	-0.413	0.030	4.6E-16	Ppp1r1a	ENSRNOG00000036827	7	145,154,131
11171	A_44_P436859	0.667	0.132	1.3E-05	0.751	0.066	1.8E-13	Mre11a	ENSRNOG00000009506	8	13,305,152
17711	A_44_P119527	-1.511	0.156	1.4E-11	-1.991	0.078	1.0E-24	Med17	ENSRNOG00000051989	8	13,526,025
8626	A_44_P100886	0.929	0.178	3.0E-05	1.426	0.115	1.8E-11		ENSRNOG00000010999	8	13,909,188
30089	A_42_P809733	-0.338	0.059	1.7E-06	-0.208	0.030	3.1E-08	RGD1309410	ENSRNOG00000011162	8	14,060,394
11775	A_44_P518434	-0.812	0.094	2.7E-10	-0.834	0.047	2.1E-19	Mrpl4	ENSRNOG00000020659	8	22,021,213
1575	A_44_P238246	0.718	0.134	4.9E-06	0.621	0.067	4.2E-11	Ilf3	ENSRNOG00000022741	8	22,402,890
25445	A_43_P11901	0.572	0.096	7.5E-07	-0.250	0.048	7.7E-06	Kcnj5	ENSRNOG00000033796	8	33,463,467
12442	A_42_P779282	1.385	0.110	9.0E-15	1.637	0.055	5.2E-27	Scn3b	ENSRNOG00000057221	8	44,136,496
12147	A_64_P118392	-0.326	0.070	4.0E-05	-0.286	0.035	9.0E-10	Nlrx1	ENSRNOG00000052386	8	48,597,867
1405	A_42_P598602	-1.028	0.217	3.4E-05	-0.501	0.109	4.8E-05	Cd3g	ENSRNOG00000015945	8	49,280,901
12744	A_43_P11865	0.415	0.094	9.0E-05	0.301	0.047	2.2E-07	Pts	ENSRNOG00000009250	8	54,961,265
13478	A_64_P093452	-0.437	0.086	1.1E-05	-0.202	0.043	3.5E-05	RGD1305464	ENSRNOG00000018689	8	61,917,125
3353	A_44_P1030943	-0.341	0.061	2.4E-06	0.179	0.030	9.9E-07	Mpi	ENSRNOG00000018898	8	62,332,115
7313	A_64_P032579	-0.472	0.085	2.6E-06	-0.285	0.042	7.8E-08	Rab27a	ENSRNOG00000052499	8	79,722,334
26700	A_64_P151869	0.747	0.157	3.0E-05	0.830	0.078	1.2E-12	Myo5a	ENSRNOG00000058866	8	82,037,977
13551	A_64_P081939	2.769	0.310	8.3E-09	3.152	0.200	1.6E-13		ENSRNOG00000025957	8	85,720,790

26870	A_64_P001219	0.362	0.072	1.2E-05	0.167	0.036	4.3E-05	Mthfs	ENSRNOG00000013229	8	96,564,877
26117	A_44_P289125	0.557	0.105	5.8E-06	0.360	0.052	4.8E-08	Spsb4	ENSRNOG00000012862	8	104,912,959
18938	A_64_P048004	0.819	0.119	4.5E-08	0.406	0.059	5.4E-08		ENSRNOG00000010899	8	111,965,889
3885	A_43_P16086	-0.669	0.143	3.9E-05	-0.368	0.071	9.1E-06	Cish	ENSRNOG00000029543	8	116,054,465
2461	A_64_P025903	-0.496	0.089	2.4E-06	-0.361	0.044	1.1E-09	Slc26a6	ENSRNOG00000020450	8	117,648,745
27455	A_42_P834104	0.330	0.058	2.1E-06	0.278	0.029	2.3E-11	Cdc25a	ENSRNOG00000020737	8	117,953,444
21735	A_64_P149924	1.180	0.143	7.5E-10	0.498	0.071	3.4E-08	Fbxl2	ENSRNOG00000027099	8	122,311,431
13157	A_64_P020581	0.292	0.056	8.0E-06	0.195	0.028	3.7E-08	Crtap	ENSRNOG00000048343	8	122,421,384
5815	A_43_P11432	-0.608	0.085	2.1E-08	-0.837	0.043	6.7E-21	Acaa1a	ENSRNOG00000032908	8	128,027,958
360	A_64_P120679	-2.033	0.160	1.1E-14	-2.150	0.081	9.2E-25	Cck	ENSRNOG00000019321	8	130,127,392
588	A_44_P388755	0.843	0.167	6.9E-05	0.781	0.147	3.9E-05	Kif15	ENSRNOG00000060356	8	132,032,944
11481	A_44_P113974	1.170	0.169	3.9E-08	0.965	0.084	1.4E-13	Ccr5	ENSRNOG00000049115	8	133,210,473
11479	A_64_P153159	0.540	0.116	4.3E-05	1.240	0.058	4.3E-22	Ebi3	ENSRNOG00000050509	9	11,114,843
28637	A_64_P006077	-0.837	0.183	5.3E-05	-0.490	0.091	4.9E-06	LOC682829	ENSRNOG00000050521	9	14,513,037
6284	A_42_P512838	0.469	0.105	7.6E-05	0.856	0.053	3.1E-18	Trem2	ENSRNOG00000013578	9	14,618,013
22777	A_64_P146497	1.959	0.173	1.8E-13	1.148	0.086	1.7E-15	Cnpy3	ENSRNOG00000016315	9	16,543,688
26431	A_43_P15195	0.382	0.066	1.4E-06	0.169	0.033	1.1E-05	Polr1c	ENSRNOG00000019079	9	17,120,759
5468	A_43_P22042	0.698	0.115	5.6E-07	0.478	0.058	6.7E-10	Pla2g7	ENSRNOG00000025691	9	19,978,013
14908	A_64_P078852	0.919	0.178	8.9E-06	0.960	0.089	7.3E-13	RGD1560151	ENSRNOG00000046426	9	20,004,280
1988	A_42_P459212	0.687	0.120	1.6E-06	0.799	0.060	1.7E-15	Tnfrsf21	ENSRNOG00000011517	9	20,621,051
8885	A_44_P217919	1.747	0.169	2.4E-12	1.313	0.085	1.4E-17	Myot	ENSRNOG00000050004	9	37,728,008
8527	A_64_P069739	1.353	0.124	5.7E-13	1.196	0.062	1.4E-20	Ankrd23	ENSRNOG00000016151	9	43,116,521
20812	A_44_P166524	-0.721	0.136	5.7E-06	-0.467	0.068	4.5E-08	Lonrf2	ENSRNOG00000023312	9	45,505,767
3969	A_42_P825912	0.421	0.083	1.2E-05	-0.270	0.042	1.5E-07		ENSRNOG00000011677	9	60,039,297
5552	A_42_P667782	-0.501	0.073	5.6E-08	-0.342	0.037	4.0E-11	Fastkd2	ENSRNOG00000023923	9	70,450,444
29481	A_64_P024826	-0.718	0.148	8.5E-05	-0.642	0.125	4.4E-05	Crygd	ENSRNOG00000032219	9	71,778,323
4543	A_43_P14184	0.304	0.059	9.4E-06	0.139	0.029	3.5E-05	Acadl	ENSRNOG00000012966	9	73,871,888
13713	A_44_P531653	1.801	0.155	8.9E-14	1.849	0.077	9.8E-24	Myl1	ENSRNOG00000013262	9	73,958,480
25119	A_64_P102930	0.505	0.063	1.6E-09	0.446	0.032	2.6E-16	Atic	ENSRNOG00000015511	9	78,862,013
11375	A_64_P055930	0.605	0.130	4.0E-05	0.501	0.065	3.5E-09	Tmbim1	ENSRNOG00000014797	9	81,586,469
14961	A_64_P059491	-0.988	0.186	5.6E-06	-0.673	0.093	1.5E-08	Plcd4	ENSRNOG00000016361	9	81,816,872
8548	A_64_P139485	-1.192	0.169	2.6E-08	-0.566	0.084	7.5E-08	Plcd4	ENSRNOG00000016361	9	81,816,872
20938	A_42_P674200	0.367	0.076	2.8E-05	0.206	0.039	6.6E-06	Rnf25	ENSRNOG00000016886	9	81,879,211

16469	A_43_P11476	-1.206	0.202	7.4E-07	-0.589	0.101	1.1E-06	Inha	ENSRNOG00000020097	9	82,700,468
9798	A_44_P438863	0.501	0.091	3.0E-06	1.071	0.045	1.5E-23	Serpine2	ENSRNOG00000015461	9	85,626,094
29140	A_43_P18275	0.445	0.091	2.2E-05	0.482	0.046	1.4E-12	Agfg1	ENSRNOG00000015619	9	88,607,508
8283	A_43_P11985	1.607	0.296	4.3E-06	1.065	0.151	3.0E-08	Ccl20	ENSRNOG00000015992	9	88,918,433
25683	A_64_P157743	1.635	0.344	3.1E-05	1.070	0.172	3.4E-07	Ccl20	ENSRNOG00000015992	9	88,918,433
29722	A_64_P045191	2.635	0.379	7.0E-07	1.837	0.214	2.4E-08	Dner	ENSRNOG00000016640	9	92,291,220
18250	A_64_P091464	0.674	0.074	6.2E-11	0.659	0.037	1.5E-19	Sp100	ENSRNOG00000022769	9	92,681,078
18795	A_43_P10901	1.513	0.084	1.2E-19	1.406	0.042	8.1E-29	Itm2c	ENSRNOG00000017359	9	92,916,469
17490	A_64_P064234	-0.695	0.133	7.3E-06	-0.371	0.066	2.5E-06	B3gnt7	ENSRNOG00000018267	9	93,326,283
5606	A_44_P467591	-2.049	0.193	1.2E-12	-1.702	0.097	2.5E-19	Neu2	ENSRNOG00000016962	9	94,702,129
30046	A_64_P087940	1.313	0.171	4.1E-09	1.182	0.085	5.0E-16	Rbm44	ENSRNOG00000024878	9	98,279,022
16420	A_64_P110873	-0.458	0.084	4.0E-06	-0.466	0.042	3.8E-13	Ndufa10	ENSRNOG00000016470	9	99,651,813
1205	A_42_P739929	1.550	0.112	4.9E-16	1.218	0.056	2.2E-22		ENSRNOG00000051528	9	114,111,580
357	A_42_P744999	-0.368	0.061	5.6E-07	-0.350	0.030	1.1E-13	Rab12	ENSRNOG00000019295	9	114,709,546
20373	A_64_P034114	-0.802	0.139	1.5E-06	-1.019	0.070	9.5E-17	Epb41l3	ENSRNOG00000016724	9	117,538,009
11494	A_44_P123492	-0.842	0.119	2.5E-08	-1.414	0.059	1.1E-23	Epb41l3	ENSRNOG00000016724	9	117,538,009
300	A_42_P826853	0.785	0.166	3.3E-05	1.086	0.083	2.6E-15	Tnfrsf17	ENSRNOG00000021987	10	4,257,868
983	A_44_P1038028	1.108	0.160	4.2E-08	0.959	0.080	4.0E-14	Tnfrsf12a	ENSRNOG00000003546	10	12,997,930
16980	A_64_P159959	1.433	0.121	4.8E-14	1.524	0.060	1.4E-24	Tpsab1	ENSRNOG00000024181	10	14,703,668
7078	A_64_P060003	-0.807	0.144	2.4E-06	-0.454	0.072	2.8E-07	Wwc1	ENSRNOG00000008065	10	20,818,128
9066	A_64_P027288	1.047	0.183	1.6E-06	0.822	0.091	9.4E-11	RGD1564516	ENSRNOG00000038894	10	31,880,918
17349	A_44_P367550	0.768	0.123	3.4E-07	0.299	0.062	2.4E-05	Sqstm1	ENSRNOG00000003147	10	35,716,294
7761	A_44_P273989	0.355	0.066	4.8E-06	0.377	0.033	1.6E-13	Ppp2ca	ENSRNOG00000005389	10	37,554,664
9604	A_64_P067654	1.169	0.126	4.0E-11	0.619	0.063	8.9E-12	Aldh3a1	ENSRNOG00000002331	10	47,490,153
27222	A_64_P048486	0.876	0.166	8.4E-06	0.723	0.096	1.2E-08	Hs3st3a1	ENSRNOG00000024591	10	50,928,309
24608	A_64_P025618	-0.443	0.070	2.7E-07	-0.500	0.035	2.2E-16	Slc25a35	ENSRNOG00000004668	10	55,555,089
18624	A_64_P101792	-0.616	0.125	1.9E-05	-0.277	0.063	8.5E-05	Efnb3	ENSRNOG00000010320	10	56,167,426
1240	A_44_P477406	-0.575	0.115	1.6E-05	-0.401	0.058	3.5E-08	Slc2a4	ENSRNOG00000017226	10	56,558,487
14716	A_64_P104481	-0.395	0.088	7.5E-05	-0.213	0.044	2.7E-05	Spag7	ENSRNOG00000004246	10	57,291,146
11003	A_44_P853916	0.829	0.096	2.3E-10	0.436	0.048	6.7E-11	Slc43a2	ENSRNOG00000003835	10	63,677,396
23809	A_44_P1018622	0.889	0.139	2.2E-07	0.699	0.070	5.6E-12	Fam101b	ENSRNOG00000006674	10	64,202,380
17068	A_43_P17012	0.336	0.072	4.2E-05	0.264	0.036	1.3E-08	Glod4	ENSRNOG00000007788	10	64,398,294
12673	A_64_P066699	-0.540	0.108	1.5E-05	-0.383	0.054	2.5E-08	Timm22	ENSRNOG00000007988	10	64,556,064

6452	A_44_P839975	-0.654	0.127	9.8E-06	-0.378	0.064	8.1E-07	Tmem132e	ENSRNOG00000007455	10	69,737,422
7469	A_64_P039447	-0.408	0.090	6.3E-05	-0.500	0.045	3.5E-13	Heatr6	ENSRNOG00000002542	10	71,054,344
21499	A_44_P960156	-0.465	0.086	3.9E-06	-0.522	0.043	2.3E-14	Usp32	ENSRNOG00000027711	10	72,417,070
20323	A_64_P091857	1.227	0.189	1.5E-07	1.150	0.095	2.4E-14	RGD1311564	ENSRNOG00000005447	10	74,298,599
4760	A_44_P762828	0.484	0.087	2.7E-06	-0.210	0.044	2.7E-05	LOC497978	ENSRNOG00000002338	10	76,407,989
1958	A_64_P075938	-0.580	0.108	4.6E-06	-0.554	0.054	2.8E-12	Cox11	ENSRNOG00000052096	10	78,111,050
7956	A_64_P004584	0.744	0.148	1.4E-05	0.590	0.074	1.8E-09	Cacna1g	ENSRNOG00000060528	10	82,197,848
5476	A_64_P150256	0.784	0.141	2.7E-06	-0.431	0.071	4.9E-07	Epn3	ENSRNOG00000003284	10	82,229,140
14953	A_64_P021606	-0.894	0.080	3.2E-13	-1.387	0.040	2.8E-29	Mycbpap	ENSRNOG00000042912	10	82,252,963
9226	A_42_P458494	-1.327	0.164	1.3E-09	-1.296	0.082	8.7E-18	Acsf2	ENSRNOG00000003330	10	82,326,771
3031	A_43_P16949	-0.894	0.170	6.6E-06	-2.230	0.085	3.8E-25	Sgca	ENSRNOG00000003998	10	82,785,142
143	A_64_P070448	-0.487	0.074	1.2E-07	-0.760	0.037	1.7E-21	Nxph3	ENSRNOG00000005185	10	83,332,851
19934	A_64_P142229	1.421	0.090	8.7E-18	1.392	0.045	1.3E-27	Gngt2	ENSRNOG00000006108	10	83,655,460
112	A_42_P778587	1.383	0.076	1.1E-19	1.357	0.038	1.0E-29	Gngt2	ENSRNOG00000006108	10	83,655,460
27361	A_64_P049761	0.670	0.073	5.7E-11	0.319	0.037	2.0E-10	Nfe2l1	ENSRNOG00000008830	10	84,698,886
29794	A_43_P12209	-0.676	0.088	3.9E-09	-0.495	0.044	2.1E-13	Stat5b	ENSRNOG00000019075	10	88,754,829
14026	A_42_P829031	-0.300	0.056	4.4E-06	-0.216	0.028	3.3E-09	Coasy	ENSRNOG00000019918	10	88,992,487
17829	A_64_P147164	-0.376	0.070	5.1E-06	-0.200	0.035	1.8E-06	Tubg1	ENSRNOG00000020213	10	89,030,865
6804	A_64_P150509	-0.824	0.169	2.3E-05	-1.007	0.086	1.1E-13	Ccr10	ENSRNOG00000020275	10	89,088,993
24388	A_44_P377156	-1.018	0.209	2.2E-05	-0.561	0.104	4.6E-06	Cntnap1	ENSRNOG00000020277	10	89,089,646
4593	A_42_P517554	-0.518	0.078	9.3E-08	-0.550	0.039	2.8E-16	Polg2	ENSRNOG00000013728	10	94,979,259
8186	A_44_P398864	0.494	0.088	2.0E-06	0.436	0.044	6.5E-12	Cog1	ENSRNOG00000002795	10	102,167,771
15465	A_64_P149794	0.588	0.077	5.2E-09	0.330	0.039	3.5E-10	Fam104a	ENSRNOG00000002851	10	102,200,400
2179	A_64_P100240	0.468	0.102	5.4E-05	0.249	0.051	2.2E-05	Llgl2	ENSRNOG00000004834	10	104,368,247
22560	A_64_P157691	-1.324	0.201	1.1E-07	-1.714	0.101	7.3E-19	RGD1559482	ENSRNOG00000048771	10	104,952,237
29929	A_44_P497302	-0.638	0.109	1.1E-06	-0.566	0.055	2.3E-12	St6galnac2	ENSRNOG00000012083	10	105,668,593
10737	A_64_P001424	1.212	0.071	6.5E-19	1.304	0.035	2.9E-30	Mxra7	ENSRNOG00000042915	10	105,771,972
12301	A_44_P520929	-0.930	0.106	1.9E-10	-0.786	0.053	6.7E-17	C1qtnf1	ENSRNOG00000003259	10	107,455,845
24209	A_44_P227616	-0.405	0.082	1.6E-05	-0.427	0.041	1.7E-12	Engase	ENSRNOG00000027498	10	107,502,695
22363	A_64_P117233	-0.707	0.120	1.0E-06	-0.391	0.060	1.5E-07		ENSRNOG00000003699	10	108,630,823
27031	A_44_P506741	1.364	0.137	6.9E-12	0.970	0.069	2.6E-16	Azi1	ENSRNOG00000047814	10	109,244,701
12448	A_64_P153134	-0.391	0.079	1.8E-05	-0.178	0.040	7.1E-05	LOC688310	ENSRNOG00000047517	10	109,630,005
22449	A_42_P539095	-0.483	0.073	9.6E-08	-0.204	0.036	2.4E-06	LOC688311	ENSRNOG00000049235	10	109,639,054

Disease Models & Mechanisms • Supplementary information

8870	A_42_P621872	-1.278	0.137	3.4E-11	-0.679	0.068	6.9E-12	Gcgr	ENSRNOG00000036692	10	109,707,962
9588	A_64_P032052	0.758	0.155	2.2E-05	0.542	0.078	3.4E-08	LOC688319	ENSRNOG00000048172	10	109,904,259
6976	A_44_P352538	1.781	0.198	4.8E-10	1.626	0.105	6.2E-16	Sectm1a	ENSRNOG00000036672	10	110,274,768
23085	A_64_P048951	2.739	0.231	5.0E-14	3.421	0.115	5.8E-27	Tex19	ENSRNOG00000036671	10	110,308,514
14465	A_64_P138565	-0.557	0.085	1.3E-07	-0.469	0.043	4.2E-13	Narf	ENSRNOG00000036664	10	110,445,797
22819	A_44_P159569	0.954	0.118	1.3E-09	1.102	0.059	3.9E-20	Wdr45l	ENSRNOG00000036662	10	110,555,629
1712	A_42_P727022	-0.712	0.088	1.4E-09	-0.574	0.044	3.5E-15	Rab40b	ENSRNOG00000036661	10	110,585,376
13697	A_64_P013526	-0.763	0.173	8.6E-05	-0.803	0.086	3.9E-11	Cxadr	ENSRNOG00000001557	11	16,826,399
20993	A_64_P071362	0.676	0.096	3.0E-08	0.670	0.048	4.2E-16	Krtap16-5	ENSRNOG00000040064	11	28,900,376
5982	A_64_P071367	0.617	0.092	8.4E-08	0.659	0.046	1.9E-16	Krtap16-5	ENSRNOG00000040064	11	28,900,376
17844	A_64_P067708	-0.532	0.076	2.8E-08	-0.657	0.038	3.8E-19	Tmem50b	ENSRNOG00000002028	11	31,752,243
1270	A_42_P581567	0.492	0.079	3.6E-07	0.562	0.040	2.4E-16	Gart	ENSRNOG00000028292	11	31,805,728
15351	A_64_P072973	1.097	0.139	3.4E-09	1.473	0.073	1.3E-20	Kcne2	ENSRNOG00000029811	11	32,440,237
12330	A_44_P121280	4.027	0.279	1.4E-16	3.838	0.140	7.7E-26	Kcne1	ENSRNOG00000001984	11	32,508,420
23688	A_64_P116600	1.543	0.131	5.7E-14	1.004	0.065	1.9E-17	Rcan1	ENSRNOG00000001979	11	32,550,539
26800	A_64_P116606	1.458	0.177	8.6E-10	0.915	0.089	2.6E-12	Rcan1	ENSRNOG00000001979	11	32,550,539
2645	A_44_P1039678	-0.398	0.081	2.0E-05	-0.302	0.041	8.5E-09	Setd4	ENSRNOG00000001699	11	33,801,999
16374	A_64_P036352	-0.774	0.094	8.1E-10	-0.779	0.047	1.8E-18	Cbr1	ENSRNOG00000049911	11	33,863,500
3179	A_42_P554722	-0.746	0.078	1.7E-11	-0.682	0.039	2.7E-19	Pigp	ENSRNOG00000039850	11	34,598,275
8239	A_64_P103808	0.365	0.079	5.0E-05	0.220	0.040	2.8E-06	Dscr3	ENSRNOG00000001681	11	34,791,993
8216	A_64_P322881	0.568	0.092	4.0E-07	0.613	0.046	1.7E-15	Erg	ENSRNOG00000001652	11	35,749,594
1366	A_64_P057975	0.346	0.067	9.2E-06	0.170	0.034	1.2E-05	Hmgn1	ENSRNOG00000050978	11	36,479,868
6884	A_42_P732251	-0.943	0.100	3.1E-11	-1.160	0.050	3.1E-23	Phldb2	ENSRNOG00000002171	11	57,404,196
6750	A_64_P013875	-0.950	0.135	3.0E-08	-0.825	0.068	2.3E-14		ENSRNOG00000002171	11	57,430,166
24679	A_64_P058744	-0.426	0.074	1.3E-06	-0.418	0.037	1.8E-13	Abhd10	ENSRNOG00000043107	11	57,486,365
1239	A_43_P12572	0.558	0.101	2.7E-06	0.553	0.050	4.4E-13	Cd200	ENSRNOG00000002141	11	60,371,729
19518	A_64_P127803	-3.334	0.243	6.6E-16	-3.795	0.122	9.3E-28	Btla	ENSRNOG00000030246	11	60,547,201
7489	A_64_P083933	0.742	0.108	5.0E-08	0.750	0.054	4.9E-16	Adcy5	ENSRNOG00000002229	11	68,842,320
27241	A_43_P11558	1.277	0.275	4.5E-05	0.935	0.138	6.1E-08	Apod	ENSRNOG00000048273	11	72,705,129
13604	A_44_P436604	0.792	0.138	1.5E-06	1.030	0.069	4.6E-17	Leprel1	ENSRNOG00000055751	11	78,029,038
25187	A_44_P179145	1.134	0.167	1.6E-07	1.030	0.098	1.3E-11	Dgkg	ENSRNOG00000001796	11	81,972,219
18133	A_64_P009857	-1.017	0.137	9.1E-09	-0.599	0.069	1.9E-10	Tbx1	ENSRNOG00000001892	11	86,552,022
349	A_64_P043259	-1.072	0.180	1.1E-06	-0.655	0.087	1.1E-08	P2rx6	ENSRNOG00000001873	11	87,435,185

Disease Models & Mechanisms • Supplementary information

7350	A_64_P048136	-0.673	0.141	2.9E-05	-0.337	0.070	2.9E-05	Brca2	ENSRNOG00000001111	12	504,007
26071	A_64_P030834	7.370	0.256	1.7E-26	6.700	0.128	1.2E-35		ENSRNOG00000000952	12	9,607,168
16025	A_64_P148325	1.383	0.218	2.7E-07	1.314	0.111	8.0E-14		ENSRNOG00000062055	12	11,083,897
3604	A_42_P746109	-0.766	0.085	9.1E-11	-0.618	0.043	1.1E-16	Asmtl	ENSRNOG00000028166	12	18,531,990
19145	A_64_P081501	1.142	0.097	5.5E-14	1.785	0.048	2.6E-30	Mcm7	ENSRNOG00000001349	12	19,314,016
1521	A_44_P534154	0.634	0.110	1.3E-06	0.820	0.055	4.6E-17	Cnpy4	ENSRNOG00000027175	12	19,328,957
23117	A_42_P540609	-1.064	0.122	1.9E-10	-1.150	0.061	2.5E-20	LOC680711	ENSRNOG00000031343	12	19,440,501
24237	A_64_P194847	-0.750	0.159	3.8E-05	-1.023	0.074	8.1E-16		ENSRNOG00000027061	12	19,512,591
25622	A_64_P119299	-1.267	0.190	8.9E-08	-1.190	0.095	1.0E-14		ENSRNOG00000027061	12	19,512,591
8198	A_64_P027108	-1.371	0.167	3.6E-09	-1.282	0.088	3.1E-15	RGD1562319	ENSRNOG00000039216	12	19,561,347
18384	A_64_P079080	0.729	0.165	8.6E-05	1.129	0.082	7.0E-16		ENSRNOG00000059625	12	20,667,601
1742	A_64_P144034	1.856	0.256	3.7E-07	2.770	0.181	6.4E-13	LOC363894	ENSRNOG00000045875	12	20,814,122
15839	A_64_P032265	1.212	0.193	2.8E-07	1.429	0.096	6.1E-17		ENSRNOG00000033017	12	21,678,580
29237	A_43_P17858	0.663	0.126	6.5E-06	0.600	0.063	2.1E-11	LOC685020	ENSRNOG00000033017	12	21,678,580
21128	A_43_P12143	0.459	0.089	9.1E-06	0.406	0.044	6.4E-11	Clip2	ENSRNOG00000021611	12	25,173,005
17101	A_64_P051322	-0.679	0.129	7.1E-06	-0.500	0.065	3.7E-09	Ncf1	ENSRNOG00000001480	12	25,497,104
4649	A_43_P12593	-0.696	0.157	8.0E-05	-0.911	0.078	8.6E-14	Phkg1	ENSRNOG00000000920	12	30,450,316
1906	A_64_P142962	1.811	0.276	3.4E-07	1.886	0.097	2.9E-18	Ccdc92	ENSRNOG00000021691	12	37,211,316
3175	A_44_P1030714	2.361	0.133	2.1E-19	2.524	0.067	1.1E-30	Ccdc92	ENSRNOG00000021691	12	37,211,316
16497	A_64_P032524	0.307	0.070	8.9E-05	0.367	0.035	1.4E-12	Atp6v0a2	ENSRNOG00000052704	12	37,398,329
9969	A_42_P747877	0.348	0.076	5.5E-05	0.250	0.038	1.2E-07	Eif2b1	ENSRNOG00000001039	12	37,444,103
13814	A_43_P10909	-0.430	0.092	4.0E-05	-0.344	0.046	7.6E-09	Gtf2h3	ENSRNOG00000001035	12	37,444,282
13761	A_64_P135202	-0.886	0.117	5.3E-09	-1.171	0.058	3.3E-21	Snrnp35	ENSRNOG00000001060	12	37,538,403
11508	A_64_P043731	0.607	0.090	7.1E-08	0.216	0.045	2.7E-05	Vps37b	ENSRNOG00000001086	12	37,984,790
28906	A_64_P112312	0.696	0.126	3.1E-06	0.547	0.063	2.4E-10	Clip1	ENSRNOG00000001247	12	38,345,456
6196	A_44_P686406	0.422	0.095	7.8E-05	0.213	0.047	6.7E-05	RGD1562310	ENSRNOG00000001123	12	43,940,798
12127	A_44_P1056161	-0.357	0.073	1.9E-05	-0.518	0.036	2.0E-16	Prkab1	ENSRNOG00000001142	12	46,316,236
741	A_42_P548796	-1.021	0.083	1.5E-14	-0.387	0.041	3.4E-11	Tsn	ENSRNOG00000002319	13	34,251,650
4104	A_44_P346848	-0.759	0.103	1.1E-08	-0.338	0.052	1.3E-07	Tmem177	ENSRNOG00000025484	13	35,933,243
28389	A_44_P574134	0.524	0.112	4.1E-05	0.652	0.056	9.3E-14	Slc35f5	ENSRNOG00000003403	13	41,883,137
1167	A_64_P119588	1.248	0.255	2.4E-05	0.705	0.104	8.6E-08	Tmem163	ENSRNOG00000003769	13	44,345,735
5228	A_64_P081287	-2.041	0.200	3.7E-12	-1.322	0.100	2.2E-15	Klhdc8a	ENSRNOG00000000036	13	49,074,644
23346	A_64_P013147	0.626	0.124	1.3E-05	0.729	0.062	6.5E-14	Prelp	ENSRNOG00000003120	13	50,761,306

19228	A_44_P452176	1.940	0.253	4.2E-09	2.356	0.126	3.8E-20	Chi3l1	ENSRNOG00000053272	13	51,022,681
10145	A_64_P103707	0.403	0.063	2.1E-07	-0.179	0.032	1.8E-06	Ndufv3	ENSRNOG00000027593	13	51,297,621
28935	A_64_P141541	0.342	0.055	3.5E-07	-0.221	0.028	1.6E-09	Ndufv3	ENSRNOG00000027593	13	51,297,621
3383	A_42_P653257	0.534	0.117	5.7E-05	0.367	0.059	3.0E-07	Ptpn7	ENSRNOG00000005807	13	51,958,834
22664	A_42_P577677	0.850	0.135	2.8E-07	0.349	0.068	8.9E-06	Tnni1	ENSRNOG00000009073	13	52,624,878
16675	A_64_P055196	0.552	0.125	8.9E-05	0.514	0.063	8.9E-10	Camsap1l1	ENSRNOG00000008741	13	53,225,349
19291	A_44_P475444	1.543	0.106	1.2E-16	1.264	0.053	1.1E-23	RGD1564614	ENSRNOG00000042369	13	56,958,549
2005	A_42_P819656	1.928	0.097	5.3E-21	1.469	0.049	3.1E-27	Cfh	ENSRNOG00000030715	13	57,080,549
17693	A_64_P034344	1.398	0.088	6.7E-18	0.961	0.044	2.0E-22	Cfh	ENSRNOG00000030715	13	57,080,549
6797	A_64_P035709	0.685	0.123	2.5E-06	0.296	0.061	2.6E-05	B3galt2	ENSRNOG00000003251	13	60,435,946
5214	A_44_P109862	2.093	0.166	8.9E-15	1.275	0.083	2.1E-17	Ivns1abp	ENSRNOG00000002618	13	68,707,776
24164	A_44_P971500	1.898	0.112	1.0E-18	1.111	0.056	5.8E-21		ENSRNOG00000002618	13	68,707,776
17011	A_64_P097847	-0.390	0.089	9.5E-05	-0.454	0.044	3.4E-12	RGD1309104	ENSRNOG00000028236	13	69,385,074
9844	A_64_P113615	1.142	0.209	4.1E-06	0.973	0.109	1.9E-10		ENSRNOG00000057851	13	75,059,927
17702	A_64_P033270	1.080	0.073	6.5E-17	1.061	0.037	1.1E-26	LOC680254	ENSRNOG00000057851	13	75,059,927
4122	A_44_P358194	2.488	0.238	1.8E-12	2.534	0.119	4.9E-22	Fmo3	ENSRNOG00000003620	13	80,862,963
14514	A_44_P180259	0.804	0.092	2.2E-10	1.585	0.046	3.5E-29	Dpt	ENSRNOG00000002947	13	83,073,550
26528	A_64_P016901	1.309	0.118	3.7E-13	1.261	0.059	4.5E-22	Creg1	ENSRNOG00000003291	13	83,972,212
22348	A_44_P1018997	-0.373	0.075	1.7E-05	-0.411	0.038	5.1E-13	Tmco1	ENSRNOG00000003928	13	85,465,792
3268	A_64_P005029	0.823	0.101	1.1E-09	0.715	0.051	2.9E-16	Pcp4l1	ENSRNOG00000003209	13	89,565,813
314	A_42_P487031	-0.319	0.053	6.8E-07	-0.225	0.027	4.3E-10	B4galt3	ENSRNOG00000003551	13	89,643,881
13087	A_44_P282716	0.515	0.067	4.1E-09	0.538	0.034	5.1E-18	Nit1	ENSRNOG00000003881	13	89,725,806
12930	A_64_P014213	0.467	0.081	1.4E-06	0.226	0.041	2.5E-06	Pex19	ENSRNOG00000057116	13	90,514,336
6787	A_64_P067035	-0.660	0.099	8.4E-08	-0.266	0.049	4.5E-06	Casq1	ENSRNOG00000006930	13	90,602,365
6685	A_64_P126623	0.617	0.140	8.7E-05	0.658	0.070	2.8E-11	Fcgr2a	ENSRNOG00000046663	13	91,228,901
20982	A_64_P126851	0.656	0.131	1.4E-05	0.796	0.065	2.4E-14	LOC498276	ENSRNOG00000046663	13	91,228,935
1235	A_64_P069641	0.383	0.087	8.6E-05	0.285	0.043	1.1E-07	Psen2	ENSRNOG00000002879	13	98,529,040
23959	A_44_P177914	0.504	0.115	9.1E-05	0.323	0.057	2.1E-06	Nvl	ENSRNOG00000003629	13	99,468,778
4088	A_44_P217715	0.953	0.132	1.7E-08	0.932	0.066	2.9E-16	Enah	ENSRNOG00000031934	13	100,405,339
19118	A_43_P12532	1.235	0.170	1.5E-08	1.300	0.085	2.4E-17	Tgfb2	ENSRNOG00000002418	13	105,141,030
3795	A_43_P13418	-0.886	0.145	4.6E-07	-0.604	0.072	5.9E-10	Kcnk2	ENSRNOG00000002653	13	107,831,014
10054	A_64_P054568	1.261	0.233	6.0E-06	0.729	0.100	2.5E-08	Vash2	ENSRNOG00000003832	13	109,552,589
9059	A_42_P803590	-1.434	0.080	1.6E-19	-1.374	0.040	3.8E-29	LOC289378	ENSRNOG00000046460	13	109,663,364

Disease Models & Mechanisms • Supplementary information

10206	A_64_P052464	1.769	0.181	2.9E-11	1.270	0.087	5.6E-16	Ints7	ENSRNOG00000004263	13	110,257,571
3954	A_42_P661065	1.382	0.150	5.4E-11	1.345	0.075	1.5E-19	Ints7	ENSRNOG00000004263	13	110,257,571
4659	A_64_P155629	0.554	0.098	2.1E-06	0.697	0.049	2.3E-16	Csf2ra	ENSRNOG00000049782	14	1,462,358
16112	A_64_P072873	1.459	0.176	6.8E-10	1.390	0.088	8.0E-18	Cplx1	ENSRNOG00000000060	14	2,194,933
3732	A_42_P522488	-0.498	0.098	1.2E-05	-0.476	0.049	1.4E-11	Znf644	ENSRNOG00000002112	14	4,125,380
12879	A_44_P552655	-0.881	0.118	7.8E-09	-0.450	0.059	4.8E-09	Abcg3l1	ENSRNOG00000030216	14	6,541,171
21816	A_64_P085715	0.702	0.086	9.8E-10	0.491	0.043	1.5E-13	Pkd2	ENSRNOG00000002146	14	6,645,257
3605	A_44_P491796	3.152	0.357	1.5E-10	1.735	0.178	1.2E-11	Spp1	ENSRNOG00000043451	14	6,679,901
10617	A_44_P1043814	-0.397	0.079	1.4E-05	-0.335	0.040	4.2E-10	Hsd17b11	ENSRNOG00000002210	14	7,073,445
19510	A_64_P021440	0.781	0.157	1.6E-05	0.481	0.072	1.1E-07	Slc10a6	ENSRNOG00000002057	14	7,618,022
16277	A_64_P138543	0.435	0.080	4.2E-06	0.267	0.040	9.3E-08	Bmp2k	ENSRNOG00000002040	14	14,192,048
18694	A_44_P325508	0.473	0.069	5.7E-08	0.274	0.035	2.2E-09	Anxa3	ENSRNOG00000002045	14	14,426,437
23631	A_64_P013437	0.991	0.144	4.4E-08	0.399	0.072	2.7E-06	Cxcl13	ENSRNOG00000024899	14	15,258,207
13827	A_44_P404591	0.933	0.127	1.2E-08	0.703	0.064	3.9E-13	Hopx	ENSRNOG00000024689	14	33,354,807
1112	A_64_P041476	-0.524	0.094	2.5E-06	-0.481	0.047	3.1E-12	LOC498368	ENSRNOG00000002191	14	46,054,022
21733	A_44_P386824	0.435	0.092	3.5E-05	0.484	0.046	1.6E-12	Yipf7	ENSRNOG00000002224	14	62,595,854
22	A_44_P992306	-0.549	0.085	1.5E-07	-0.209	0.042	1.8E-05	Gnpda2	ENSRNOG00000002177	14	62,646,110
18510	A_64_P143400	1.275	0.156	9.9E-10	0.897	0.078	1.2E-13	Gba3	ENSRNOG00000024634	14	64,535,170
21557	A_44_P177950	0.331	0.076	9.8E-05	0.260	0.038	4.7E-08	Med28	ENSRNOG00000003592	14	70,098,021
25255	A_64_P150071	-0.251	0.057	9.4E-05	-0.249	0.029	2.2E-10	Rab28	ENSRNOG00000017074	14	73,889,213
3647	A_42_P571433	-0.527	0.117	6.9E-05	-0.649	0.059	3.6E-13	Msx1	ENSRNOG00000006876	14	77,712,240
8159	A_44_P898195	0.802	0.155	8.8E-06	0.380	0.078	2.0E-05	Cytl1	ENSRNOG00000028108	14	77,810,147
9903	A_64_P155691	-0.342	0.072	3.2E-05	-0.630	0.036	2.9E-19	Zfp278	ENSRNOG00000018709	14	83,510,640
18032	A_44_P402445	1.076	0.161	4.3E-07	1.851	0.113	2.6E-15	Nefh	ENSRNOG00000008716	14	85,191,557
19355	A_64_P244845	0.726	0.084	2.9E-10	0.510	0.042	2.9E-14	Tns3	ENSRNOG00000025695	14	88,886,198
21874	A_64_P089280	0.609	0.106	1.6E-06	0.584	0.053	4.7E-13	Tns3	ENSRNOG00000025695	14	88,886,198
5272	A_64_P038050	1.268	0.083	2.6E-17	-0.209	0.042	1.4E-05	Acyp2	ENSRNOG00000042419	14	115,052,450
10722	A_64_P108876	0.873	0.078	2.7E-13	0.256	0.039	1.2E-07	Dusp13	ENSRNOG00000057795	15	2,526,368
30065	A_42_P829349	0.498	0.067	9.2E-09	0.176	0.034	7.0E-06	Comtd1	ENSRNOG00000013968	15	2,631,529
736	A_42_P653332	-0.342	0.063	3.7E-06	-0.163	0.031	8.4E-06	Mrps16	ENSRNOG00000006898	15	4,351,292
15076	A_44_P289862	0.437	0.093	3.8E-05	0.412	0.047	1.4E-10	Abhd6	ENSRNOG00000008167	15	18,675,431
18294	A_44_P234129	-0.817	0.108	5.9E-09	-0.678	0.054	9.7E-15	Dlgap5	ENSRNOG00000010721	15	24,199,341
12606	A_64_P098006	-0.457	0.070	1.4E-07	-0.356	0.035	3.9E-12	Fitm1	ENSRNOG00000019019	15	34,251,606

Disease Models & Mechanisms • Supplementary information

27632	A_64_P151264	-0.607	0.100	5.0E-07	-0.699	0.050	3.3E-16	Fam158a	ENSRNOG00000019162	15	34,263,224
12799	A_64_P153273	-1.241	0.172	2.0E-08	-0.430	0.080	4.8E-06	Mcpt10	ENSRNOG00000049991	15	34,694,180
510	A_64_P068192	-1.435	0.122	6.3E-14	-0.680	0.061	2.9E-13	Mcpt8	ENSRNOG00000049991	15	34,694,244
10995	A_64_P124294	-1.413	0.130	6.0E-13	-0.813	0.065	1.1E-14	Mcpt9	ENSRNOG00000031792	15	35,002,406
10858	A_64_P028371	-0.713	0.124	1.5E-06	-1.747	0.062	3.4E-26	Phf11	ENSRNOG00000053891	15	38,709,984
23836	A_64_P151851	-0.668	0.150	7.8E-05	-1.125	0.075	4.2E-17	Phf11	ENSRNOG00000053891	15	38,709,984
7039	A_64_P022452	0.670	0.144	4.6E-05	1.509	0.073	3.5E-21	Pebp4	ENSRNOG00000026247	15	51,535,864
16197	A_44_P654933	-0.653	0.144	6.2E-05	-0.380	0.072	6.5E-06	Piwil2	ENSRNOG00000009871	15	52,116,001
17860	A_64_P142046	-0.358	0.069	7.3E-06	0.253	0.034	1.0E-08	Nudt15	ENSRNOG00000025239	15	55,424,024
12137	A_43_P14977	0.617	0.085	1.6E-08	1.364	0.043	4.2E-28	Tsc22d1	ENSRNOG00000001030	15	58,554,374
6846	A_64_P005043	0.847	0.088	1.7E-11	0.773	0.044	2.9E-19	Nt5dc2	ENSRNOG00000018358	16	7,212,488
30347	A_64_P066923	0.810	0.115	3.0E-08	0.716	0.058	1.3E-14	Ncoa4	ENSRNOG00000019768	16	8,302,950
25712	A_64_P153759	1.297	0.132	9.0E-12	0.890	0.066	1.1E-15		ENSRNOG00000029662	16	9,430,743
23686	A_64_P131151	0.556	0.088	2.3E-07	0.688	0.044	9.4E-18	Opn4	ENSRNOG00000053893	16	10,952,549
5974	A_42_P596050	1.857	0.215	2.5E-10	0.941	0.107	1.8E-10	Pcdh21	ENSRNOG00000013330	16	14,348,046
1186	A_42_P467381	1.103	0.062	2.0E-19	0.870	0.031	4.3E-26	Fam32a	ENSRNOG00000039528	16	19,308,842
9044	A_64_P137784	-0.580	0.102	1.8E-06	-0.698	0.051	7.4E-16	Plvap	ENSRNOG00000017676	16	19,918,689
11317	A_44_P177414	-0.349	0.067	7.0E-06	-0.157	0.033	3.6E-05	Fam125a	ENSRNOG00000017949	16	19,973,636
10403	A_64_P011489	2.738	0.225	2.3E-14	2.453	0.112	2.1E-22	Gdf15	ENSRNOG00000019661	16	20,555,395
19569	A_44_P1024788	0.465	0.079	1.1E-06	0.547	0.040	5.9E-16	LOC498606	ENSRNOG00000019971	16	20,657,099
6546	A_64_P124701	0.940	0.139	6.3E-08	1.325	0.069	1.8E-20	Crlf1	ENSRNOG00000020030	16	20,686,317
9891	A_64_P056648	0.948	0.099	1.7E-11	1.544	0.049	8.8E-28	Crlf1	ENSRNOG00000020030	16	20,686,317
15836	A_44_P295789	1.122	0.235	2.9E-05	0.601	0.117	1.0E-05	Comp	ENSRNOG00000048472	16	20,807,070
23904	A_64_P100904	-1.239	0.163	5.4E-09	-1.289	0.082	8.2E-18	LOC688966	ENSRNOG00000020427	16	21,029,134
25971	A_64_P004531	-1.051	0.084	9.0E-15	-0.926	0.042	1.2E-22	LOC688966	ENSRNOG00000020427	16	21,029,134
4835	A_44_P1016480	0.723	0.091	1.8E-09	0.405	0.045	1.2E-10	Lpl	ENSRNOG00000012181	16	22,561,496
8763	A_64_P144978	0.704	0.099	2.4E-08	0.602	0.050	2.5E-14	Csgalnact1	ENSRNOG00000013024	16	22,979,444
8543	A_44_P265003	-1.024	0.113	8.5E-11	-1.128	0.057	4.7E-21	Anxa10	ENSRNOG00000014339	16	29,674,793
24363	A_64_P010393	0.461	0.093	1.8E-05	0.377	0.047	1.3E-09	Clcn3	ENSRNOG00000010682	16	32,449,116
15146	A_44_P1036068	-0.479	0.072	9.0E-08	-0.472	0.036	2.6E-15	RGD1311747	ENSRNOG00000011293	16	32,540,217
26255	A_64_P130050	1.081	0.197	3.3E-06	0.976	0.098	7.5E-12	Hand2	ENSRNOG00000060448	16	36,373,546
3982	A_64_P007462	0.653	0.111	1.0E-06	1.047	0.056	2.9E-20	Spcs3	ENSRNOG00000038933	16	40,050,734
26075	A_64_P041016	-1.311	0.125	1.7E-12	-1.202	0.063	1.5E-20	Wwc2	ENSRNOG00000013248	16	47,368,768

8059	A_42_P664913	-1.245	0.191	1.4E-07	-0.703	0.095	1.1E-08	Ankrd37	ENSRNOG00000031335	16	49,462,889
27848	A_44_P1021361	0.977	0.105	4.2E-11	0.744	0.053	2.6E-16	Tlr3	ENSRNOG00000021726	16	50,016,857
171	A_64_P040806	1.184	0.086	9.0E-16	1.126	0.044	1.9E-24	Tlr3	ENSRNOG00000021726	16	50,016,857
25609	A_64_P097598	0.408	0.079	8.3E-06	0.461	0.039	7.3E-14	Mtus1	ENSRNOG00000010748	16	54,332,660
9554	A_64_P048315	1.067	0.119	1.0E-10	1.308	0.059	1.6E-22		ENSRNOG00000055088	16	59,517,000
27771	A_44_P1000842	1.166	0.145	2.6E-08	0.723	0.091	3.7E-08	Tex15	ENSRNOG00000007748	16	62,373,253
28774	A_44_P438492	0.493	0.078	2.6E-07	0.986	0.039	1.4E-24	Mak16	ENSRNOG00000010783	16	64,729,221
11412	A_44_P1042163	0.906	0.089	4.0E-12	1.279	0.045	1.8E-26	RGD1310414	ENSRNOG00000023494	16	64,745,207
1931	A_43_P12961	0.470	0.089	6.4E-06	0.297	0.045	8.9E-08	Eif4ebp1	ENSRNOG00000012582	16	68,968,248
1443	A_42_P798776	-0.453	0.087	8.4E-06	-0.555	0.044	6.7E-15	LOC100364957	ENSRNOG00000059911	16	70,905,993
17467	A_64_P121981	-1.234	0.206	7.9E-07	-0.688	0.105	1.4E-07	Htra4	ENSRNOG00000061160	16	71,787,966
28259	A_64_P157693	-0.489	0.095	8.7E-06	-0.677	0.047	1.8E-16	Ank1	ENSRNOG00000018241	16	73,827,488
4278	A_43_P12782	0.536	0.071	5.5E-09	0.291	0.035	7.6E-10	Ikbkb	ENSRNOG00000019073	16	74,177,215
1616	A_43_P12689	2.315	0.426	5.1E-06	2.577	0.205	3.6E-14	Defb1	ENSRNOG00000013768	16	75,309,176
6310	A_44_P368065	0.713	0.118	6.2E-07	0.284	0.059	2.7E-05	Adprhl1	ENSRNOG00000062013	16	81,616,604
28399	A_44_P189837	-0.470	0.098	2.6E-05	-0.522	0.049	9.6E-13	Carkd	ENSRNOG00000015021	16	83,342,667
18894	A_64_P022751	1.110	0.104	9.6E-13	0.884	0.052	7.8E-19	Isca1	ENSRNOG00000018343	17	5,281,727
19764	A_44_P429781	0.361	0.065	2.8E-06	0.177	0.033	4.1E-06	Mak10	ENSRNOG00000018417	17	5,463,898
30268	A_44_P215479	0.725	0.081	1.2E-10	0.495	0.041	2.6E-14	Rmi1	ENSRNOG00000019108	17	6,660,536
20495	A_64_P300082	0.610	0.111	3.1E-06	0.581	0.055	1.6E-12	Spin1	ENSRNOG00000046068	17	14,091,766
8900	A_64_P059262	1.105	0.061	1.1E-19	1.070	0.031	1.7E-29	Riok1	ENSRNOG00000014049	17	27,451,832
21031	A_64_P067678	-0.671	0.088	4.7E-09	-0.565	0.044	4.6E-15	Aldh5a1	ENSRNOG00000023538	17	42,133,076
23925	A_64_P030919	-0.723	0.075	1.3E-11	-0.714	0.037	1.6E-20		ENSRNOG00000023538	17	42,133,076
24052	A_64_P017273	0.645	0.105	4.5E-07	0.388	0.053	1.0E-08	Lrrc16a	ENSRNOG00000016576	17	43,050,789
14569	A_64_P057801	0.478	0.087	3.2E-06	0.658	0.043	3.1E-17	Hist1h2bl	ENSRNOG00000047776	17	43,808,673
20825	A_64_P004639	0.508	0.093	3.4E-06	0.675	0.046	1.1E-16	Hist1h2bc	ENSRNOG00000059382	17	44,738,330
13074	A_64_P130572	0.464	0.096	2.5E-05	0.679	0.048	2.6E-16	Hist1h2bc	ENSRNOG00000059382	17	44,738,330
5615	A_44_P506017	0.483	0.109	8.6E-05	0.681	0.055	1.2E-14		ENSRNOG00000054296	17	44,793,927
25440	A_43_P12803	0.490	0.104	3.7E-05	0.363	0.052	3.5E-08	Arid4b	ENSRNOG00000049751	17	53,915,076
17147	A_44_P238897	0.832	0.141	9.1E-07	0.811	0.070	1.2E-13		ENSRNOG00000018110	17	55,346,279
17288	A_64_P165005	-0.506	0.090	2.3E-06	-0.545	0.045	2.9E-14	Fzd8	ENSRNOG00000061031	17	62,262,129
22265	A_64_P014867	0.579	0.113	9.8E-06	0.643	0.056	1.6E-13	Pfkp	ENSRNOG00000017163	17	68,559,471
25805	A_44_P220256	-0.633	0.092	4.8E-08	-0.669	0.046	1.2E-16	Prtfdc1	ENSRNOG00000024874	17	88,037,040

Disease Models & Mechanisms • Supplementary information

20226	A_64_P104454	-0.992	0.101	1.0E-11	-0.634	0.051	9.9E-15	Impad1	ENSRNOG00000046647	17	90,218,013
20714	A_64_P056712	0.963	0.144	9.9E-08	0.479	0.067	2.3E-08	Gpr137b	ENSRNOG00000002480	17	90,696,019
30354	A_64_P089742	-0.338	0.073	4.4E-05	-0.427	0.036	6.6E-14	Cdig2	ENSRNOG00000012016	18	3,662,683
14497	A_64_P174430	-0.324	0.072	6.5E-05	-0.351	0.036	1.0E-11	Cdig2	ENSRNOG00000012016	18	3,662,683
16785	A_44_P1028952	-0.393	0.079	1.7E-05	-0.297	0.040	7.0E-09		ENSRNOG00000016467	18	6,474,990
3764	A_44_P469584	0.902	0.204	8.6E-05	0.685	0.102	7.6E-08	Cd14	ENSRNOG00000017819	18	29,562,153
24116	A_64_P026818	0.662	0.101	1.2E-07	0.465	0.050	5.1E-11	Gramd3	ENSRNOG00000015225	18	51,492,196
2027	A_44_P1006931	-1.192	0.087	7.4E-16	-0.311	0.044	2.1E-08	LOC100174910	ENSRNOG00000013738	18	52,047,816
1555	A_64_P079379	-0.997	0.193	8.6E-06	-0.433	0.096	6.8E-05	Fbn2	ENSRNOG00000043219	18	53,181,503
25074	A_64_P045764	0.669	0.116	1.4E-06	0.563	0.058	1.3E-11	Adamts19	ENSRNOG00000019577	18	53,915,807
2039	A_64_P135224	2.710	0.116	2.3E-23	2.459	0.058	2.2E-32	RGD1309362	ENSRNOG00000038960	18	55,505,993
3030	A_44_P538334	-0.615	0.136	6.6E-05	-0.571	0.068	5.6E-10	Impa2	ENSRNOG00000018516	18	63,016,577
10937	A_43_P22556	-0.902	0.094	1.6E-11	-1.264	0.047	1.5E-25	Cidea	ENSRNOG00000018505	18	63,098,144
9291	A_64_P156333	-0.699	0.085	8.4E-10	-1.314	0.042	1.3E-27	Cidea	ENSRNOG00000018505	18	63,098,144
12179	A_64_P078837	-1.349	0.131	2.9E-12	-1.059	0.066	4.3E-18		ENSRNOG00000055190	18	63,839,026
18168	A_44_P213175	-1.357	0.129	1.5E-12	-0.534	0.065	7.3E-10	Ccdc68	ENSRNOG00000021381	18	68,408,890
17166	A_44_P419615	-1.078	0.138	2.9E-09	-0.842	0.069	2.3E-14	Stard6	ENSRNOG00000026324	18	68,983,545
24668	A_64_P128569	0.281	0.058	2.2E-05	0.322	0.029	3.0E-13	Rpl38	ENSRNOG00000033808	18	69,818,720
7335	A_42_P595630	-0.607	0.059	2.9E-12	-0.537	0.030	9.3E-20	Pqlc1	ENSRNOG00000059215	18	76,770,012
22519	A_64_P021320	-0.659	0.056	6.3E-14	-0.671	0.028	8.6E-24	Pqlc1	ENSRNOG00000059215	18	76,770,012
24850	A_64_P102870	0.350	0.076	4.6E-05	0.337	0.038	1.2E-10	Fam96b	ENSRNOG00000011865	19	551,708
18626	A_64_P097078	-0.735	0.115	1.9E-07	-0.632	0.057	4.0E-13	Pdp2	ENSRNOG00000012343	19	601,469
17810	A_64_P148413	-0.474	0.100	3.5E-05	-0.886	0.050	2.4E-19	Ces1d	ENSRNOG00000015519	19	15,033,108
6593	A_42_P721692	-0.771	0.105	1.0E-08	-0.751	0.052	1.6E-16	Inpp4b	ENSRNOG00000018382	19	29,951,493
7992	A_44_P1046713	0.367	0.082	6.8E-05	0.197	0.041	2.6E-05	Cog4	ENSRNOG00000017745	19	43,391,828
28178	A_64_P103254	-0.410	0.091	6.5E-05	-0.202	0.045	8.1E-05	Klhl36	ENSRNOG00000016422	19	52,515,022
13312	A_64_P117413	0.819	0.147	2.6E-06	1.020	0.073	4.7E-16	RGD1304884	ENSRNOG00000017431	19	53,723,822
18126	A_44_P192288	0.527	0.121	9.9E-05	0.304	0.060	1.3E-05	RGD1561507	ENSRNOG00000038239	19	56,010,085
12200	A_64_P035694	1.361	0.158	2.6E-10	0.752	0.079	2.0E-11	LOC678766	ENSRNOG00000057601	19	56,443,632
1276	A_43_P18610	0.684	0.129	5.7E-06	0.506	0.064	2.4E-09	RGD1563235	ENSRNOG00000017690	19	56,633,633
26328	A_44_P255236	0.839	0.109	3.8E-09	1.479	0.054	1.1E-25	Acta1	ENSRNOG00000017786	19	56,677,084
8933	A_64_P121016	0.696	0.114	4.6E-07	0.675	0.057	4.8E-14	Exoc8	ENSRNOG00000019766	19	57,649,827
12642	A_64_P121262	-1.059	0.065	3.5E-18	-0.860	0.033	3.3E-25	Sipa1l2	ENSRNOG00000019791	19	58,399,816

Disease Models & Mechanisms • Supplementary information

28321	A_44_P299349	-0.737	0.126	1.0E-06	-1.198	0.063	1.9E-20	Prr3	ENSRNOG00000025806	20	3,300,247
4436	A_42_P627572	-0.979	0.116	4.7E-10	-0.812	0.058	3.8E-16	Mdc1	ENSRNOG00000032813	20	3,405,285
16747	A_64_P113970	-0.298	0.064	4.5E-05	-0.292	0.032	6.9E-11	Gtf2h4	ENSRNOG00000000831	20	3,582,742
18363	A_64_P134927	0.477	0.109	9.6E-05	0.433	0.054	1.8E-09	RT1-Db1	ENSRNOG00000033215	20	4,087,618
27475	A_44_P991532	0.492	0.109	7.0E-05	0.506	0.055	4.7E-11	RT1-Da	ENSRNOG00000032844	20	4,132,616
8347	A_44_P367438	0.449	0.081	2.8E-06	0.505	0.041	1.2E-14	Stk38	ENSRNOG00000000519	20	6,257,604
14913	A_44_P914438	1.506	0.134	2.3E-13	1.000	0.067	4.6E-17	Cdkn1a	ENSRNOG00000000521	20	6,356,423
20527	A_43_P15406	1.904	0.156	2.1E-13	0.606	0.085	5.3E-08	Grm4	ENSRNOG00000000487	20	6,772,903
10330	A_44_P1000115	0.511	0.091	2.2E-06	0.595	0.045	2.6E-15	RGD735065	ENSRNOG00000000524	20	6,869,767
12621	A_44_P532249	0.706	0.150	3.7E-05	0.445	0.075	8.6E-07	Fkbp5	ENSRNOG00000022523	20	8,019,020
21148	A_64_P145286	-1.591	0.183	2.2E-10	-1.198	0.092	2.8E-15	Dnah8	ENSRNOG00000000542	20	9,313,271
7522	A_64_P121031	-1.732	0.196	1.4E-10	-1.529	0.098	1.1E-17		ENSRNOG00000000542	20	9,313,271
17120	A_44_P305200	-0.284	0.063	6.9E-05	-0.176	0.032	2.4E-06	Cstb	ENSRNOG00000001201	20	10,968,432
3691	A_42_P781999	-0.689	0.096	1.9E-08	-0.558	0.048	9.0E-14	Agpat3	ENSRNOG00000001205	20	11,114,164
8306	A_64_P008361	0.635	0.108	1.1E-06	0.660	0.054	2.4E-14	Pwp2	ENSRNOG00000001210	20	11,228,844
25935	A_64_P146698	0.664	0.100	9.3E-08	0.691	0.050	5.1E-16	Pwp2	ENSRNOG00000001210	20	11,228,844
24999	A_44_P840891	-1.392	0.161	1.6E-09	-1.013	0.091	4.7E-12	Icoslg	ENSRNOG00000023109	20	11,340,296
9892	A_44_P992662	1.504	0.159	3.2E-11	1.100	0.081	1.4E-15	Ftcd	ENSRNOG00000001261	20	12,820,466
19033	A_44_P1015203	1.111	0.127	1.9E-10	0.753	0.064	5.2E-14	Gstt3	ENSRNOG00000001242	20	13,817,795
25463	A_43_P13290	3.615	0.189	1.6E-20	2.924	0.094	1.2E-27	Pbld	ENSRNOG00000000386	20	27,117,663
8781	A_44_P152177	4.190	0.339	5.7E-14	3.800	0.107	6.0E-28	Spock2	ENSRNOG00000061544	20	29,655,226
405	A_42_P559737	0.579	0.082	2.8E-08	0.566	0.041	5.7E-16	Srgn	ENSRNOG00000000394	20	32,133,431
987	A_42_P461003	-0.421	0.082	1.0E-05	-0.203	0.041	1.7E-05	Snx3	ENSRNOG00000046705	20	47,253,664
4080	A_64_P076865	-0.518	0.091	1.9E-06	-0.729	0.046	6.0E-18	LOC641520	ENSRNOG00000047102	20	50,394,650
110	A_64_P099457	-0.838	0.169	2.8E-05	-0.580	0.106	7.0E-06	Hoxa9	ENSRNOG00000060292	L567939	87,504
20987	A_64_P141038	0.837	0.120	3.4E-08	0.297	0.060	1.7E-05	Bex4	ENSRNOG00000060103	L568122	30,542
10171	A_64_P022135	0.571	0.129	8.3E-05	0.617	0.064	1.9E-11		ENSRNOG00000031780	MT	1
4493	A_64_P147403	2.008	0.103	1.1E-20	2.105	0.052	8.5E-32		ENSRNOG00000031780	MT	1
7214	A_44_P759995	1.477	0.135	5.5E-13	0.855	0.068	8.1E-15		ENSRNOG00000031780	MT	1
16708	A_44_P311620	-0.648	0.093	3.4E-08	-0.305	0.046	1.2E-07		ENSRNOG00000031780	MT	1
889	A_44_P403017	0.805	0.108	8.5E-09	0.553	0.054	3.3E-12		ENSRNOG00000031780	MT	1
5065	A_64_P146350	-0.516	0.101	1.1E-05	-0.327	0.051	1.7E-07		ENSRNOG00000031780	MT	1
23156	A_44_P914760	1.038	0.102	3.4E-12	0.289	0.051	1.8E-06		ENSRNOG00000031780	MT	1

26156	A_64_P068425	0.685	0.143	2.8E-05	0.770	0.071	7.7E-13	ENSRNOG00000031780	MT	1
24098	A_64_P042811	-1.527	0.218	3.2E-08	-2.384	0.109	2.0E-22	ENSRNOG00000031780	MT	1
24070	A_64_P085331	0.332	0.075	8.7E-05	0.328	0.038	2.0E-10	ENSRNOG00000031780	MT	1
22332	A_64_P040803	0.373	0.066	1.8E-06	0.360	0.033	4.9E-13	ENSRNOG00000031780	MT	1
29125	A_64_P084699	0.323	0.062	8.6E-06	0.365	0.031	7.6E-14	ENSRNOG00000031780	MT	1
9394	A_64_P119214	0.492	0.073	6.8E-08	0.715	0.036	7.1E-21	ENSRNOG00000031780	MT	1
24899	A_64_P063443	-0.720	0.141	3.0E-05	-0.489	0.084	5.5E-06	ENSRNOG00000031780	MT	1
9336	A_64_P063588	-0.745	0.085	1.9E-10	-0.819	0.043	1.4E-20	ENSRNOG00000031780	MT	1
24429	A_64_P086550	-1.074	0.159	6.6E-08	-0.792	0.079	6.5E-12	ENSRNOG00000031780	MT	1
15576	A_64_P073174	0.312	0.068	4.9E-05	0.175	0.034	8.7E-06	ENSRNOG00000031780	MT	1
21135	A_64_P118308	-1.194	0.206	1.2E-06	-1.017	0.103	8.1E-12	ENSRNOG00000031780	MT	1
27679	A_64_P143154	0.712	0.111	2.0E-07	0.744	0.056	1.5E-15	ENSRNOG00000031780	MT	1
211	A_42_P619996	-0.326	0.071	5.2E-05	-0.356	0.036	5.8E-12	ENSRNOG00000031780	MT	1
19166	A_64_P052972	-0.855	0.117	1.2E-08	-0.689	0.058	5.7E-14	ENSRNOG00000031780	MT	1
5482	A_64_P326359	1.678	0.101	1.6E-18	1.243	0.050	3.4E-24	ENSRNOG00000031780	MT	1
16475	A_64_P071381	0.657	0.096	4.7E-08	0.687	0.048	1.6E-16	ENSRNOG00000031780	MT	1
17573	A_64_P042099	0.562	0.094	7.3E-07	0.601	0.047	5.5E-15	ENSRNOG00000031780	MT	1
6958	A_64_P042096	0.604	0.111	3.6E-06	0.619	0.055	2.6E-13	ENSRNOG00000031780	MT	1
12287	A_64_P047720	-1.136	0.228	3.1E-05	-2.481	0.149	9.5E-16	ENSRNOG00000031780	MT	1
186	A_64_P148326	0.880	0.177	1.7E-05	0.778	0.089	1.8E-10	ENSRNOG00000031780	MT	1
28481	A_44_P392004	2.168	0.145	1.0E-15	2.790	0.081	2.1E-26	ENSRNOG00000031780	MT	1
29293	A_64_P029190	0.744	0.142	7.5E-06	1.109	0.071	1.3E-17	ENSRNOG00000031780	MT	1
23113	A_64_P145766	0.543	0.124	9.7E-05	0.440	0.062	2.3E-08	ENSRNOG00000031780	MT	1
2399	A_44_P793124	-0.376	0.061	3.7E-07	-0.281	0.030	4.3E-11	ENSRNOG00000031780	MT	1
10120	A_64_P135893	1.059	0.102	1.4E-11	1.440	0.059	7.8E-22	ENSRNOG00000031780	MT	1
22436	A_64_P045775	1.252	0.149	1.3E-09	1.902	0.074	4.3E-23	ENSRNOG00000031780	MT	1
25519	A_64_P129231	0.547	0.090	5.0E-07	0.440	0.045	9.3E-12	ENSRNOG00000031780	MT	1
11784	A_64_P085918	0.395	0.078	1.2E-05	0.415	0.039	1.1E-12	ENSRNOG00000031780	MT	1
7558	A_64_P119802	-0.551	0.086	1.9E-07	-0.200	0.043	4.0E-05	ENSRNOG00000031780	MT	1
1339	A_64_P042093	1.090	0.150	1.4E-08	1.563	0.075	9.0E-22	ENSRNOG00000031780	MT	1
26291	A_64_P142460	-0.628	0.127	1.8E-05	-0.807	0.064	7.2E-15	ENSRNOG00000031780	MT	1
13083	A_64_P077603	-0.673	0.103	1.2E-07	-0.844	0.051	2.3E-18	ENSRNOG00000031780	MT	1
4008	A_64_P098848	0.595	0.120	1.9E-05	0.485	0.062	4.9E-09	ENSRNOG00000031780	MT	1

Disease Models & Mechanisms • Supplementary information

18772	A_64_P090798	0.601	0.135	7.9E-05	0.388	0.068	1.5E-06		ENSRNOG00000031780	MT	1
4590	A_64_P033230	-0.820	0.169	2.4E-05	-0.611	0.085	1.7E-08		ENSRNOG00000031780	MT	1
28308	A_64_P028365	0.335	0.042	1.9E-09	0.315	0.021	5.0E-17		ENSRNOG00000031780	MT	1
21103	A_64_P039347	0.627	0.116	4.1E-06	0.262	0.058	6.2E-05		ENSRNOG00000031780	MT	1
6792	A_64_P157318	0.720	0.117	4.3E-07	0.334	0.059	1.7E-06		ENSRNOG00000031780	MT	1
2193	A_44_P535899	0.814	0.110	9.3E-09	0.331	0.055	6.4E-07		ENSRNOG00000029707	MT	10,160
28451	A_64_P123361	-2.154	0.296	1.4E-08	-1.032	0.148	3.4E-08		ENSRNOG00000029042	MT	14,061
1960	A_42_P508471	-0.659	0.123	7.0E-06	-0.335	0.068	2.5E-05	Rgn	ENSRNOG00000007949	X	1,848,904
8917	A_64_P145716	1.748	0.146	4.0E-14	1.053	0.073	1.5E-16	Maoa	ENSRNOG00000002848	X	6,620,722
8786	A_64_P062462	0.723	0.104	3.8E-08	0.300	0.052	1.4E-06	Pir	ENSRNOG00000003674	X	31,968,152
15094	A_64_P102468	1.020	0.193	6.0E-06	0.566	0.096	1.0E-06		ENSRNOG00000024479	X	39,956,841
15483	A_64_P111300	-0.553	0.095	1.2E-06	-0.357	0.048	7.3E-09	LOC678880	ENSRNOG00000045535	X	73,616,348
8565	A_64_P087409	0.527	0.110	2.9E-05	0.528	0.055	1.9E-11	RGD1561318	ENSRNOG00000050970	X	99,389,351
8471	A_64_P288221	3.241	0.235	2.2E-11	2.837	0.182	2.5E-12	LOC679989	ENSRNOG00000011034	X	106,116,886
17278	A_64_P140144	2.735	0.163	1.1E-18	1.871	0.081	3.4E-23	LOC679989	ENSRNOG00000011034	X	106,116,886
3600	A_64_P102049	1.728	0.186	3.8E-11	1.125	0.093	2.7E-14	LOC680319	ENSRNOG00000037645	X	106,774,980
27661	A_64_P102043	1.572	0.154	3.3E-12	1.054	0.077	7.0E-16	LOC680319	ENSRNOG00000037645	X	106,774,980
1547	A_42_P803274	1.223	0.117	1.7E-12	0.583	0.058	6.4E-12	Wbp5	ENSRNOG00000034198	X	106,791,333
1615	A_42_P741034	-0.381	0.084	5.8E-05	-0.653	0.042	1.3E-17		ENSRNOG00000055721	X	110,818,716
265	A_64_P121643	-0.674	0.154	9.4E-05	-0.524	0.077	5.5E-08	Capn6	ENSRNOG00000004882	X	115,073,890
15663	A_64_P048852	-1.576	0.087	1.1E-19	-1.417	0.044	2.3E-28		ENSRNOG00000006459	X	120,313,696
187	A_64_P130668	-0.672	0.108	3.5E-07	-0.501	0.054	4.2E-11	Klhl13	ENSRNOG00000014029	X	121,731,543
18535	A_64_P070155	2.245	0.251	1.1E-10	2.102	0.126	1.3E-18	Rhox5	ENSRNOG00000046548	X	123,912,361
948	A_42_P637189	-1.043	0.176	8.5E-07	-1.044	0.088	4.9E-14	Apln	ENSRNOG00000003984	X	134,866,210
18231	A_64_P045426	1.170	0.153	4.7E-09	1.449	0.077	2.5E-20		ENSRNOG00000048494	X	145,822,398
11811	A_43_P11812	0.669	0.102	1.1E-07	0.463	0.051	6.6E-11	Bgn	ENSRNOG00000055962	X	157,331,204
3177	A_42_P476474	0.693	0.132	7.3E-06	0.845	0.066	5.8E-15	Fhl1	ENSRNOG00000000875	X	159,112,880
27064	A_64_P004348	0.344	0.071	2.4E-05	0.280	0.036	2.3E-09	Ftl			
12619	A_64_P060319	-0.743	0.118	2.9E-07	-0.857	0.059	1.2E-16	no transcript			
6725	A_64_P063194	-0.399	0.070	1.8E-06	-0.187	0.035	5.5E-06	no transcript			
17114	A_44_P877632	0.483	0.095	1.1E-05	0.348	0.047	1.2E-08	Otulin (LOC1003	ENSRNOG00000012017	2	80,267,724
5960	A_44_P114491	0.535	0.092	1.2E-06	0.600	0.046	3.1E-15	no transcript			
25822	A_44_P314087	1.143	0.221	1.0E-05	0.770	0.115	1.1E-07	no transcript			

21575	A_64_P110744	-0.460	0.086	4.7E-06	-0.330	0.043	3.7E-09	Shld1 (LOC681292)_partial			
28819	A_64_P004279	-0.722	0.140	9.6E-06	-0.924	0.070	2.3E-15	RGD1561472			
17474	A_44_P201084	-0.856	0.162	6.3E-06	-0.704	0.081	2.2E-10	Fgfr1l			
7034	A_44_P534028	1.057	0.119	1.2E-10	0.971	0.059	2.7E-18	no transcript			
28213	A_44_P476598	1.208	0.155	3.2E-09	0.923	0.078	4.9E-14	Phf24 (RGD1559864)_no homolog			
21356	A_42_P588504	1.400	0.104	1.1E-15	0.858	0.052	1.8E-18	Phf24 (RGD1559864)_no homolog			
30162	A_44_P745269	-0.573	0.077	9.5E-09	-0.292	0.039	6.4E-09	no transcript			
8819	A_64_P132726	-1.060	0.125	4.4E-10	-0.711	0.063	1.8E-13	no transcript			
27091	A_64_P025432	5.071	0.215	1.4E-23	4.681	0.107	7.3E-33	no transcript			
9344	A_64_P029333	-0.610	0.094	1.6E-07	-0.814	0.047	4.9E-19	Acaa1b			
12460	A_64_P029335	-0.703	0.092	4.2E-09	-0.769	0.046	1.1E-18	Acaa1b			
19485	A_64_P161768	0.404	0.087	4.2E-05	0.408	0.043	2.9E-11	Synrg			
10999	A_64_P070586	0.639	0.092	3.6E-08	0.680	0.046	6.3E-17	Krtap21-2			
20392	A_64_P081063	2.195	0.157	8.9E-13	2.735	0.094	9.3E-20	no transcript			
15659	A_43_P18931	0.797	0.102	2.9E-09	0.537	0.051	1.5E-12	Tp53i3 (RGD130	ENSRNOG00000005177	13	75,101,759
15442	A_64_P155603	-1.444	0.143	4.3E-12	-0.725	0.071	3.8E-12	RGD1565970			
26191	A_44_P154298	-1.538	0.177	2.3E-10	-1.091	0.089	1.7E-14	RGD1565970			
17854	A_64_P116919	1.279	0.176	1.4E-08	0.579	0.088	1.1E-07	LOC681303			
8329	A_64_P124041	-1.598	0.202	2.1E-09	-0.788	0.101	2.9E-09	Ddx60			
6183	A_42_P546857	-0.353	0.062	1.7E-06	-0.201	0.031	1.4E-07	Coprs (RGD1565675)_ no homolog			
16435	A_64_P078050	1.236	0.134	4.6E-11	0.933	0.067	3.9E-16	RGD1559908			
7429	A_64_P157171	1.307	0.169	3.7E-09	0.848	0.085	5.7E-12	no transcript			
25709	A_64_P011719	-0.909	0.077	5.9E-14	-0.885	0.039	3.8E-23	Chmp1b	N/A	18	62,923,620
24897	A_64_P150597	-1.990	0.214	6.9E-11	-0.714	0.101	3.4E-08	no transcript			
11195	A_64_P084698	0.317	0.070	6.2E-05	0.395	0.035	2.0E-13	LOC680353			

Genes are regarded as significant for “shared” differential expression when they show $P<1\times10^{-4}$ reproducibly in two types of comparison, ie, SHR/Izm versus WKY/Izm and SHRSP/Izm versus WKY/Izm, with a concordant direction of differential expression.

Table S3B. A list of significant transcripts for shared differential expression in the aorta.

no	ProbeName	SHR/lzm (vs WKY/lzm)			SHRSP/lzm (vs WKY/lzm)			GeneName	Ensembl_rat	Chr	Transcription_ start_site (Rnor_6.0)
		Fold change (log2-scale)	SE (log2-scale)	P-value	Fold change (log2-scale)	SE (log2-scale)	P-value				
14405	A_44_P452186	1.467	0.196	1.3E-06	1.402	0.175	5.6E-07	Epm2a	ENSRNOG00000040242	1	5,448,958
8464	A_64_P129293	2.185	0.164	1.1E-09	1.706	0.156	1.5E-08	RGD1306565	ENSRNOG00000031700	1	15,412,603
8390	A_43_P23129	-0.685	0.125	5.0E-05	-0.834	0.112	1.4E-06	Ptprk	ENSRNOG00000047605	1	18,058,055
3394	A_42_P484738	1.439	0.261	4.8E-05	2.613	0.234	5.9E-09	Ctgf	ENSRNOG00000015036	1	21,854,773
11848	A_64_P121575	-1.029	0.111	7.8E-08	-0.574	0.099	2.8E-05	Slc12a7	ENSRNOG00000016372	1	32,140,137
22666	A_44_P866091	-0.964	0.122	6.6E-07	-0.631	0.109	2.8E-05	Slc12a7	ENSRNOG00000016372	1	32,140,137
24761	A_64_P139942	-1.439	0.237	3.0E-05	-1.328	0.220	3.1E-05	Slc6a19	ENSRNOG00000026501	1	32,199,810
5937	A_64_P110103	-0.414	0.076	5.3E-05	-0.578	0.068	2.5E-07	Sod2	ENSRNOG00000019048	1	47,921,587
16064	A_64_P107642	-0.406	0.073	4.4E-05	-0.621	0.065	5.7E-08	Tmem160	ENSRNOG00000015304	1	78,417,719
16383	A_64_P042455	-0.567	0.074	1.1E-06	-0.704	0.067	1.3E-08	Bloc1s3	ENSRNOG00000017463	1	80,416,273
1747	A_42_P641234	-0.628	0.077	4.3E-07	-0.540	0.069	7.2E-07	Snrpa	ENSRNOG00000001501	1	84,008,293
27603	A_64_P125676	-1.111	0.168	6.2E-06	-1.480	0.151	3.6E-08	Sptbn4	ENSRNOG00000055371	1	84,254,645
24147	A_64_P131903	-0.673	0.104	8.1E-06	-0.563	0.093	1.7E-05	Dyrk1b	ENSRNOG00000019254	1	85,112,247
6144	A_42_P554610	-0.736	0.127	2.8E-05	-1.116	0.114	3.7E-08	Eid2	ENSRNOG00000019310	1	85,517,360
4928	A_64_P079807	-0.435	0.078	4.5E-05	-0.499	0.070	2.5E-06	Yif1b	ENSRNOG00000020616	1	87,180,624
17505	A_64_P063428	-1.035	0.170	1.6E-05	-1.318	0.152	1.9E-07	Tmem149	ENSRNOG00000020957	1	89,020,341
17701	A_64_P078982	-0.656	0.123	6.6E-05	-1.191	0.110	9.0E-09	LOC688869	ENSRNOG00000024309	1	89,084,859
9877	A_64_P060305	-0.875	0.149	2.4E-05	-1.077	0.133	5.0E-07	Lsr	ENSRNOG00000021053	1	89,399,041
19858	A_64_P060303	-0.746	0.124	1.8E-05	-1.105	0.111	3.0E-08	Lsr	ENSRNOG00000021053	1	89,399,041
11122	A_44_P515321	-0.521	0.083	1.2E-05	-0.560	0.075	1.3E-06	LOC499136	ENSRNOG00000014455	1	97,785,490
11981	A_64_P148550	-1.309	0.134	3.8E-08	-2.256	0.120	2.6E-12	Lim2	ENSRNOG00000017681	1	98,501,249
25711	A_44_P215157	-1.314	0.174	1.7E-06	-2.120	0.174	3.6E-09	Lin7b	ENSRNOG00000020746	1	101,360,971
15212	A_64_P166076	1.472	0.198	1.4E-06	0.933	0.177	7.7E-05	Bcat2	ENSRNOG00000020956	1	101,554,642
456	A_64_P016403	-0.724	0.123	2.4E-05	-1.070	0.110	4.3E-08	Ldha	ENSRNOG00000013009	1	102,900,286
22429	A_44_P1046705	-0.587	0.087	4.7E-06	-0.841	0.078	9.4E-09	Mrps11	ENSRNOG00000018531	1	140,477,868
26335	A_64_P157285	1.157	0.209	4.6E-05	1.349	0.187	2.1E-06	AC114460.1 (par	ENSRNOG00000025003	1	142,027,250
3363	A_64_P016059	-1.620	0.120	3.8E-10	-1.541	0.107	1.5E-10	Homer2	ENSRNOG00000061450	1	143,535,583
6438	A_64_P084767	-0.564	0.088	9.5E-06	-0.446	0.079	3.8E-05	Aamdc (RGD156	ENSRNOG00000012584	1	162,533,893
4323	A_64_P028140	0.865	0.102	2.7E-07	0.597	0.091	7.0E-06	Plekhb1	ENSRNOG00000018627	1	165,680,206

Disease Models & Mechanisms • Supplementary information

8799	A_64_P095313	-0.778	0.124	1.2E-05	-1.152	0.111	1.8E-08		ENSRNOG00000051149	1	166,727,805
28650	A_44_P177931	-0.673	0.071	6.5E-08	-0.616	0.064	4.7E-08	Anapc15 (RGD13	ENSRNOG00000019936	1	166,983,175
10242	A_64_P019125	-0.998	0.101	3.4E-08	-0.465	0.090	9.8E-05	AC098008.3	ENSRNOG00000051318	1	167,654,236
28504	A_64_P092754	1.270	0.204	2.3E-05	1.227	0.221	7.2E-05	Mrvi1	ENSRNOG00000017767	1	175,796,040
887	A_64_P152697	-1.237	0.144	2.2E-07	-0.728	0.128	3.6E-05	AABR07005563.	ENSRNOG00000062034	1	187,634,359
29269	A_64_P151353	1.131	0.116	4.2E-08	0.779	0.104	1.3E-06	Acsm5	ENSRNOG00000031211	1	189,241,593
9654	A_64_P019021	0.949	0.150	5.7E-05	1.108	0.102	3.5E-07	LOC691551	ENSRNOG00000028473	1	190,914,946
26011	A_64_P158793	-0.755	0.096	7.6E-07	-0.526	0.086	1.6E-05	AABR07005667.	ENSRNOG00000024217	1	193,537,137
21952	A_64_P024138	-6.085	0.184	4.2E-16	-6.806	0.165	1.2E-17	Sbk1	ENSRNOG00000057696	1	197,659,187
19479	A_44_P275029	-1.291	0.117	7.4E-09	-0.997	0.105	5.7E-08	Mvp	ENSRNOG00000020182	1	198,450,047
29205	A_44_P1029472	-1.901	0.115	1.9E-11	-1.366	0.103	4.9E-10	Pagr1 (RGD1305	ENSRNOG00000020217	1	198,450,688
6234	A_42_P505241	2.110	0.193	8.2E-09	1.298	0.173	1.3E-06	Qprt	ENSRNOG00000016980	1	198,559,568
13092	A_44_P285803	-0.659	0.091	2.0E-06	-0.667	0.082	4.3E-07	Dctpp1	ENSRNOG00000017850	1	198,706,852
14224	A_64_P045578	1.869	0.106	7.6E-12	2.035	0.095	3.7E-13	Rnf40	ENSRNOG00000018840	1	199,037,544
9878	A_42_P834903	-1.007	0.108	7.4E-08	-0.529	0.097	5.1E-05	Bckdk	ENSRNOG00000019485	1	199,351,628
3819	A_44_P409232	3.458	0.260	4.9E-10	1.563	0.233	5.0E-06	Cyp2e1	ENSRNOG00000012458	1	213,511,874
28576	A_64_P036285	-1.759	0.208	2.7E-07	-1.422	0.186	1.0E-06	Scgb1c1	ENSRNOG00000012847	1	213,595,240
1482	A_42_P495579	0.862	0.148	2.6E-05	1.407	0.132	1.2E-08	Ano1	ENSRNOG00000020865	1	217,844,957
5014	A_42_P614175	1.138	0.199	4.1E-05	1.962	0.189	3.2E-08	Gal	ENSRNOG00000015156	1	218,657,925
19340	A_44_P132522	-0.740	0.129	3.0E-05	-0.691	0.115	1.9E-05	Ndufs8	ENSRNOG00000017446	1	219,144,610
21218	A_64_P089356	-2.090	0.202	1.8E-08	-1.356	0.181	1.3E-06	Syt12	ENSRNOG00000019306	1	219,719,272
5718	A_64_P119314	-1.694	0.320	7.4E-05	-2.442	0.286	2.5E-07	LOC689065	ENSRNOG00000036641	1	219,745,654
9900	A_44_P635869	1.109	0.152	1.9E-06	0.808	0.136	2.1E-05	Bbs1	ENSRNOG00000019832	1	220,165,678
20841	A_64_P051802	-0.593	0.085	3.1E-06	-0.407	0.076	6.4E-05	Rab1b	ENSRNOG00000050510	1	220,491,469
27062	A_64_P150876	-0.924	0.146	1.4E-05	-1.979	0.146	8.5E-10	Cst6	ENSRNOG00000020455	1	220,729,000
18532	A_64_P053322	0.442	0.077	3.2E-05	0.465	0.069	5.0E-06	Sf1	ENSRNOG00000021085	1	221,735,517
200	A_42_P780882	-0.468	0.080	2.4E-05	-0.450	0.071	1.0E-05	Esrra	ENSRNOG00000021139	1	222,178,725
22791	A_64_P193845	-0.568	0.086	5.9E-06	-0.913	0.077	2.4E-09	Ppp1r14b	ENSRNOG00000021151	1	222,229,835
10859	A_64_P128124	-0.700	0.099	2.6E-06	-0.911	0.088	1.8E-08	Bscl2	ENSRNOG00000052393	1	225,037,737
9	A_44_P944998	-0.746	0.090	3.7E-07	-1.001	0.081	1.3E-09	LOC690344	ENSRNOG00000045561	1	225,077,079
20761	A_44_P532027	-0.749	0.127	2.4E-05	-0.681	0.114	2.0E-05	B3gat3	ENSRNOG00000019804	1	225,120,061
3243	A_42_P651632	0.675	0.097	3.3E-06	1.098	0.087	1.0E-09	Tut1	ENSRNOG00000020047	1	225,151,404
7775	A_64_P033447	-0.871	0.135	7.9E-06	-0.881	0.120	1.8E-06	Dagla	ENSRNOG00000027264	1	226,353,611

26689	A_44_P335974	1.537	0.129	2.4E-09	1.389	0.115	2.0E-09	Aldh1a7	ENSRNOG00000017878	1	240,601,744
9329	A_44_P262593	-1.610	0.180	1.3E-07	-1.746	0.161	9.3E-09	Ankrd2	ENSRNOG00000013840	1	261,281,543
7394	A_42_P644278	-1.146	0.086	4.8E-10	-0.995	0.077	7.4E-10	Bloc1s2	ENSRNOG00000012684	1	263,920,962
14196	A_64_P062643	-1.167	0.158	2.3E-06	-0.854	0.158	7.3E-05	Pkd2l1	ENSRNOG00000012591	1	263,959,318
22924	A_64_P062648	-0.992	0.175	3.6E-05	-2.154	0.157	2.9E-10	Pkd2l1	ENSRNOG00000012591	1	263,959,318
27137	A_64_P017268	-1.270	0.168	1.2E-06	-1.330	0.150	1.5E-07	Trim8	ENSRNOG00000019968	1	266,255,797
2102	A_44_P990043	0.712	0.131	5.5E-05	1.014	0.117	2.0E-07	Pdcd11	ENSRNOG00000020304	1	266,866,931
2768	A_42_P739860	2.756	0.371	1.5E-06	1.909	0.332	3.0E-05	Dusp5	ENSRNOG00000014061	1	274,245,184
28938	A_44_P240330	-0.721	0.126	3.3E-05	-1.131	0.113	2.8E-08	AABR07007032.	ENSRNOG00000046333	1	277,867,630
9184	A_43_P21414	-0.923	0.103	1.2E-07	-1.010	0.092	7.4E-09	Atg10	ENSRNOG00000016342	2	20,152,884
26408	A_44_P306344	1.133	0.181	1.6E-05	1.381	0.181	1.6E-06	F2rl2	ENSRNOG00000018054	2	26,240,385
1004	A_42_P640277	0.985	0.176	4.1E-05	0.963	0.157	1.5E-05	Enc1	ENSRNOG00000016541	2	28,049,217
15719	A_44_P202659	-0.699	0.122	3.2E-05	-0.696	0.109	9.6E-06	Ndufs4	ENSRNOG00000011383	2	46,476,203
19499	A_44_P244851	1.383	0.203	4.2E-06	1.969	0.181	8.9E-09	Car3	ENSRNOG00000010079	2	88,135,410
21613	A_43_P10081	-1.230	0.199	1.8E-05	-1.350	0.199	6.5E-06	Aadacl1	ENSRNOG00000013313	2	112,868,707
3745	A_64_P093467	3.369	0.362	3.2E-06	3.499	0.362	2.3E-06	Trpc4	ENSRNOG00000011133	2	143,475,323
4422	A_44_P761041	-1.008	0.154	6.7E-06	-0.860	0.137	1.2E-05	Trim2	ENSRNOG00000010124	2	183,210,799
29014	A_64_P154297	-0.881	0.157	3.9E-05	0.767	0.140	5.2E-05	Iqgap3	ENSRNOG00000027894	2	187,447,501
10652	A_44_P508711	-0.871	0.105	3.3E-07	-0.526	0.093	3.8E-05	Flad1	ENSRNOG00000020642	2	188,736,462
21227	A_64_P159614	-0.592	0.077	9.6E-07	-0.629	0.069	1.0E-07	Lenep	ENSRNOG00000020642	2	188,736,462
6842	A_42_P516658	-0.704	0.093	1.2E-06	-0.870	0.083	1.5E-08	Gatad2b	ENSRNOG00000015553	2	189,655,702
998	A_43_P15245	0.701	0.099	2.7E-06	0.521	0.089	2.4E-05	Npr1	ENSRNOG00000014684	2	189,856,090
20304	A_44_P132960	-1.194	0.199	2.5E-05	-1.252	0.199	1.5E-05	S100a3	ENSRNOG00000012008	2	189,955,836
3660	A_44_P184817	1.513	0.185	4.3E-07	1.723	0.166	1.6E-08	Selenbp1	ENSRNOG00000047158	2	195,423,787
27712	A_64_P041307	-1.380	0.090	6.3E-11	-1.453	0.081	5.3E-12	Tars2	ENSRNOG00000057194	2	197,878,142
16742	A_64_P155201	-1.389	0.193	4.9E-06	-1.410	0.209	9.6E-06	LOC690126	ENSRNOG00000028975	2	198,380,836
9919	A_64_P004344	-0.862	0.089	4.6E-08	-0.958	0.080	2.2E-09		ENSRNOG00000052555	2	198,388,809
19415	A_64_P098081	-2.171	0.226	8.8E-08	-2.231	0.226	6.2E-08	RGD1563516	ENSRNOG00000052555	2	198,388,809
8080	A_64_P013900	-0.648	0.112	2.7E-05	-0.600	0.100	1.8E-05	Hist2h4	ENSRNOG00000032224	2	198,412,350
18785	A_44_P269697	-1.315	0.162	4.8E-07	-1.107	0.145	1.1E-06		ENSRNOG00000060366	2	198,417,619
28930	A_64_P043871	0.519	0.081	8.6E-06	0.483	0.072	5.4E-06	Polr3gl	ENSRNOG00000021209	2	198,706,428
13090	A_64_P061012	-1.255	0.178	4.1E-06	-1.334	0.178	2.0E-06	Itga10	ENSRNOG00000021217	2	198,772,937
20853	A_64_P076891	-1.809	0.140	1.6E-09	-1.903	0.140	7.8E-10	RGD1565469	ENSRNOG00000037217	2	198,834,925

Disease Models & Mechanisms • Supplementary information

14417	A_44_P220193	-0.538	0.083	8.1E-06	-0.396	0.075	7.1E-05		ENSRNOG00000000098	2	198,852,500
12118	A_64_P040804	0.386	0.064	1.9E-05	0.404	0.058	3.0E-06	Rpl38	ENSRNOG00000030747	2	199,479,309
1601	A_64_P078624	2.678	0.297	1.9E-07	2.814	0.282	5.3E-08	Vtcn1	ENSRNOG00000015279	2	203,200,427
20012	A_42_P783572	-0.584	0.100	2.6E-05	-1.116	0.090	1.3E-09	Atp1a1	ENSRNOG00000030019	2	204,032,023
8411	A_42_P716352	1.713	0.219	7.5E-07	1.064	0.196	5.6E-05	Gstm7	ENSRNOG00000018937	2	210,782,856
2086	A_42_P488758	-0.769	0.066	3.4E-09	-0.528	0.059	1.3E-07	Alg14	ENSRNOG00000011528	2	224,851,383
1835	A_44_P1029956	0.930	0.162	3.2E-05	0.794	0.145	5.3E-05	Cnn3	ENSRNOG00000011559	2	225,005,019
16874	A_44_P323053	1.135	0.158	7.5E-06	1.393	0.194	7.4E-06	Usp53	ENSRNOG00000014660	2	227,160,379
745	A_42_P775635	-0.627	0.118	7.3E-05	-1.047	0.106	3.3E-08	Hs2st1	ENSRNOG00000012549	2	250,600,517
1173	A_64_P091053	-1.059	0.111	5.4E-08	-1.740	0.099	7.7E-12	Hs2st1	ENSRNOG00000012549	2	250,600,517
26425	A_43_P12207	1.565	0.201	8.0E-07	2.108	0.180	3.0E-09	Ddah1	ENSRNOG00000014613	2	251,634,431
20558	A_44_P114500	-1.557	0.248	1.5E-05	-2.209	0.248	2.3E-07	Gipc2	ENSRNOG00000042152	2	257,376,756
20957	A_43_P23049	-0.933	0.119	7.2E-07	-0.888	0.106	3.2E-07	Cryz	ENSRNOG00000028319	2	260,884,337
29729	A_44_P745585	-0.699	0.120	2.7E-05	-0.579	0.107	6.1E-05	LOC499749	ENSRNOG00000010896	3	2,480,232
12397	A_64_P007965	-1.936	0.169	4.1E-09	-1.424	0.151	6.3E-08	Gpsm1	ENSRNOG00000018666	3	3,767,394
24094	A_64_P064944	-0.420	0.080	8.0E-05	-0.695	0.071	4.2E-08	Trub2	ENSRNOG00000043189	3	8,348,746
29855	A_44_P945862	-0.637	0.099	8.1E-06	-0.960	0.088	8.7E-09	Trub2	ENSRNOG00000043189	3	8,348,746
19528	A_44_P1007333	0.610	0.117	8.6E-05	0.919	0.105	1.7E-07	Gle1	ENSRNOG00000015237	3	8,498,122
26230	A_44_P213133	-2.546	0.124	6.6E-13	-3.034	0.111	7.7E-15	Endog	ENSRNOG00000016033	3	8,741,766
12417	A_64_P050722	0.513	0.083	1.4E-05	0.476	0.074	9.0E-06		ENSRNOG00000031434	3	8,955,538
22393	A_43_P18366	1.570	0.142	7.1E-09	2.278	0.127	5.5E-12	Tor1b	ENSRNOG00000006435	3	9,792,899
25202	A_42_P472520	-0.774	0.074	1.6E-08	-0.638	0.066	4.8E-08	RGD1561113	ENSRNOG00000022681	3	11,410,732
1661	A_42_P705502	-1.467	0.171	2.2E-07	-1.205	0.153	6.8E-07		ENSRNOG00000050834	3	11,554,457
4674	A_42_P512417	-0.488	0.071	4.1E-06	-0.488	0.064	1.0E-06	Dpm2	ENSRNOG00000049110	3	11,587,941
13641	A_44_P466189	1.130	0.196	2.9E-05	1.017	0.175	2.7E-05	Angptl2	ENSRNOG00000016678	3	12,262,822
16648	A_64_P052670	0.478	0.087	5.1E-05	0.852	0.078	8.2E-09	Gsn	ENSRNOG00000018991	3	14,467,330
28766	A_44_P536749	-0.799	0.098	4.3E-07	-0.810	0.087	8.1E-08	Map1d	ENSRNOG00000061587	3	58,084,606
24139	A_44_P992697	-1.726	0.108	3.3E-11	-1.845	0.097	2.2E-12	Dusp19	ENSRNOG00000008868	3	67,849,966
7192	A_42_P811170	-0.576	0.095	1.6E-05	-0.801	0.085	6.1E-08	Med19	ENSRNOG00000006050	3	72,080,630
1516	A_42_P561644	-0.707	0.124	3.3E-05	-1.106	0.111	3.0E-08	Timm10	ENSRNOG00000007883	3	72,226,613
17375	A_43_P15275	-1.600	0.160	2.8E-08	-1.286	0.143	1.2E-07	Ptprj	ENSRNOG00000034025	3	79,390,956
11832	A_64_P130763	-0.639	0.083	9.6E-07	-0.474	0.074	9.4E-06	Ptpmt1	ENSRNOG00000009723	3	79,743,737
13379	A_44_P271769	-0.529	0.081	7.5E-06	-0.420	0.073	2.9E-05	Acp2	ENSRNOG00000013594	3	80,021,440

25223	A_64_P115142	-1.277	0.154	3.6E-07	-1.936	0.138	2.1E-10	Accs	ENSRNOG00000009199	3	82,756,953
9425	A_64_P110971	-0.447	0.077	2.7E-05	-0.602	0.069	1.8E-07	Rfk	ENSRNOG00000004381	3	89,745,705
14812	A_64_P143307	-0.521	0.085	1.4E-05	-1.142	0.076	7.5E-11	Rfk	ENSRNOG00000004381	3	89,745,705
4693	A_44_P340143	-0.602	0.078	9.0E-07	-0.630	0.070	1.1E-07	Commd9	ENSRNOG00000004755	3	91,463,660
6359	A_64_P097527	-0.938	0.087	1.0E-08	-0.548	0.078	2.9E-06		ENSRNOG00000006947	3	92,969,050
25469	A_64_P122489	-0.805	0.118	4.0E-06	-1.073	0.105	2.2E-08	Hdc	ENSRNOG00000010262	3	119,075,619
12846	A_44_P530547	-0.970	0.156	1.2E-05	-1.469	0.139	1.3E-08	Prom2	ENSRNOG00000014710	3	120,106,697
19676	A_64_P098527	-1.078	0.066	2.4E-11	-1.248	0.059	4.6E-13	Chchd5	ENSRNOG00000018491	3	121,660,110
29198	A_44_P1013851	1.454	0.209	3.4E-06	1.115	0.187	2.1E-05	Bmp2	ENSRNOG00000021276	3	126,335,863
18447	A_44_P336489	0.962	0.186	9.6E-05	0.991	0.167	2.1E-05	Tmx4	ENSRNOG00000024852	3	127,569,093
8484	A_64_P052705	1.975	0.136	1.3E-10	2.140	0.121	7.2E-12	Ankrd5	ENSRNOG00000005792	3	129,501,105
28951	A_64_P086580	-0.660	0.095	3.4E-06	-0.471	0.085	4.5E-05		ENSRNOG00000027574	3	132,052,612
6630	A_42_P471608	-0.544	0.102	6.6E-05	-0.670	0.091	1.6E-06	Kif16b	ENSRNOG00000004951	3	136,936,674
13519	A_44_P370537	0.912	0.145	1.4E-05	1.489	0.145	3.5E-08	Polr3f	ENSRNOG00000007548	3	138,684,685
9033	A_64_P122790	-1.195	0.149	5.6E-07	-1.074	0.133	5.3E-07	Acss1	ENSRNOG00000007102	3	146,470,293
18341	A_42_P842833	-0.725	0.123	2.2E-05	-1.384	0.110	1.0E-09	Srxn1	ENSRNOG00000031167	3	147,609,095
12668	A_44_P363204	-0.594	0.101	2.5E-05	-0.775	0.091	2.4E-07	Csnk2a1	ENSRNOG00000005276	3	147,713,821
3654	A_44_P529403	-0.755	0.131	2.9E-05	-0.866	0.117	1.5E-06	Tbc1d20	ENSRNOG00000005766	3	147,772,165
8852	A_64_P152189	-0.410	0.064	9.1E-06	-0.599	0.057	1.6E-08	Map1lc3a	ENSRNOG00000025443	3	150,801,289
15681	A_44_P389034	-1.168	0.212	4.8E-05	-1.176	0.190	1.3E-05	Tp53inp2	ENSRNOG00000018225	3	150,910,398
6036	A_44_P262407	-1.976	0.237	5.4E-07	-1.676	0.237	3.9E-06	Ggt7	ENSRNOG00000018441	3	151,032,302
2025	A_64_P073974	-0.843	0.113	1.3E-06	-0.980	0.101	4.1E-08	Myh7b	ENSRNOG00000018997	3	151,126,591
6299	A_64_P011194	-0.345	0.064	6.4E-05	-0.565	0.057	3.6E-08	Romo1	ENSRNOG00000019823	3	151,688,454
11686	A_64_P103956	-0.997	0.100	2.9E-08	-1.771	0.089	1.2E-12	Acot8	ENSRNOG00000015187	3	161,272,460
2951	A_42_P666951	-1.162	0.199	2.6E-05	-1.125	0.178	1.1E-05	Cd40	ENSRNOG00000018488	3	161,519,743
29765	A_64_P139173	-1.395	0.196	2.5E-06	-0.935	0.175	6.8E-05	Cd40	ENSRNOG00000018488	3	161,519,743
28578	A_64_P008473	0.373	0.070	7.0E-05	0.357	0.063	3.4E-05	Rpl38	ENSRNOG00000048701	3	164,002,482
13887	A_44_P317539	-0.508	0.076	5.3E-06	-0.422	0.068	1.2E-05	Psma7	ENSRNOG00000056853	3	175,426,395
16812	A_64_P058749	0.718	0.085	3.0E-07	0.691	0.076	1.1E-07		ENSRNOG00000043150	3	176,479,335
14217	A_64_P136394	1.446	0.161	2.3E-06	1.861	0.236	7.9E-06	Col20a1	ENSRNOG00000010326	3	176,494,768
29541	A_64_P059775	-2.182	0.171	1.9E-09	-3.425	0.171	3.3E-12	Stmn3	ENSRNOG00000013657	3	176,816,114
15543	A_44_P307964	-0.969	0.096	2.5E-08	-0.673	0.086	7.3E-07	Polr3k	ENSRNOG00000017843	3	177,374,812
7521	A_64_P158058	-0.511	0.086	2.0E-05	-0.680	0.077	1.5E-07	Slc4a2	ENSRNOG00000014347	4	7,281,223

Disease Models & Mechanisms • Supplementary information

24680	A_43_P12222	-0.426	0.074	3.0E-05	-0.494	0.066	1.4E-06	Pmpcb	ENSRNOG00000012693	4	9,921,610
21357	A_64_P016751	-0.982	0.104	6.1E-08	-1.208	0.093	6.6E-10	Tmem243 (RGD1	ENSRNOG00000042758	4	21,920,651
9693	A_64_P039888	0.923	0.171	9.3E-05	1.151	0.184	2.2E-05	Abcb1b	ENSRNOG00000008012	4	22,307,453
5850	A_44_P139763	0.636	0.097	6.3E-06	0.900	0.086	1.6E-08	Bet1	ENSRNOG00000011008	4	29,092,753
24172	A_64_P025464	-0.467	0.063	1.4E-06	-0.461	0.056	4.0E-07	Shfm1	ENSRNOG00000010420	4	32,087,600
29522	A_44_P489995	-0.795	0.072	6.8E-09	-1.019	0.064	3.5E-11	Mrps33	ENSRNOG00000026528	4	67,610,710
26495	A_64_P143875	0.950	0.165	3.0E-05	1.335	0.148	1.1E-07	Zyx	ENSRNOG00000017354	4	71,740,532
13479	A_64_P092534	1.255	0.168	1.4E-06	2.129	0.150	1.9E-10	Sspo	ENSRNOG00000025848	4	78,080,310
24482	A_64_P118256	-0.469	0.082	3.3E-05	-0.404	0.074	5.0E-05	Ccdc72	ENSRNOG00000043400	4	79,015,660
29493	A_44_P278387	-0.572	0.096	2.0E-05	-0.532	0.086	1.3E-05	Wipf3	ENSRNOG00000009571	4	84,597,323
19084	A_64_P078538	-0.412	0.066	1.2E-05	-0.523	0.059	1.5E-07		ENSRNOG00000011052	4	85,235,172
7404	A_43_P23115	1.650	0.122	4.0E-10	1.251	0.110	4.4E-09	Inmt	ENSRNOG00000011250	4	85,386,231
11506	A_44_P671449	-0.492	0.095	9.3E-05	-0.677	0.085	6.0E-07	Mrpl35	ENSRNOG00000008546	4	99,746,560
1827	A_64_P025633	0.707	0.110	8.4E-06	0.606	0.098	1.4E-05	Tmem150a	ENSRNOG00000061100	4	100,218,661
20592	A_44_P402507	-1.273	0.112	4.7E-09	-1.086	0.100	9.3E-09	Retsat	ENSRNOG00000014090	4	100,465,170
23887	A_44_P248947	-0.928	0.086	1.0E-08	-0.852	0.077	6.8E-09	Pole4	ENSRNOG00000006102	4	113,497,455
17379	A_44_P358440	2.744	0.385	5.3E-06	2.117	0.281	2.8E-06	Cml3	ENSRNOG00000015763	4	117,490,721
8677	A_64_P090548	-1.256	0.133	6.4E-08	-0.983	0.119	3.9E-07	Rassf4	ENSRNOG00000013526	4	148,803,988
13196	A_44_P622992	-1.353	0.245	4.7E-05	-2.276	0.219	1.7E-08	Tuba8	ENSRNOG00000048169	4	153,774,486
7183	A_64_P013456	-0.436	0.082	6.9E-05	-0.456	0.073	1.3E-05	Atn1	ENSRNOG00000060594	4	157,274,755
3106	A_64_P003046	-0.835	0.086	4.4E-08	-0.491	0.077	9.5E-06	Gapdh	ENSRNOG00000018630	4	157,679,962
19879	A_64_P052510	-0.978	0.105	7.8E-08	-0.519	0.094	4.8E-05	Gapdh	ENSRNOG00000018630	4	157,679,962
3747	A_64_P022586	-1.484	0.182	4.4E-07	-1.019	0.163	1.2E-05	Vamp1	ENSRNOG00000019219	4	157,726,941
7314	A_42_P560781	-1.162	0.189	1.4E-05	-1.002	0.169	2.1E-05	Vamp1	ENSRNOG00000019219	4	157,726,941
14671	A_64_P060628	-0.969	0.173	6.7E-05	-1.552	0.160	1.5E-07		ENSRNOG00000053804	4	160,334,910
13001	A_44_P409860	-0.839	0.147	3.2E-05	-0.773	0.131	2.3E-05		ENSRNOG00000048735	4	170,092,848
2691	A_42_P754654	-1.247	0.131	5.4E-08	-0.826	0.117	2.7E-06	RGD1562378	ENSRNOG00000045840	4	170,763,916
13669	A_64_P037993	0.913	0.177	9.6E-05	-0.846	0.158	6.7E-05	Rps4y2	ENSRNOG00000031041	4	182,745,448
11610	A_44_P271062	-0.522	0.099	7.8E-05	-0.665	0.089	1.3E-06	Tmem70	ENSRNOG00000006608	5	2,037,038
25630	A_64_P078937	0.435	0.081	7.6E-05	0.481	0.081	2.7E-05	Rpl7	ENSRNOG00000006992	5	2,632,712
17621	A_44_P412710	1.355	0.239	3.5E-05	1.705	0.214	5.8E-07	Adhfe1	ENSRNOG00000007069	5	9,429,859
2369	A_44_P415901	-0.470	0.079	2.0E-05	-0.380	0.070	6.0E-05	Mrpl15	ENSRNOG00000008566	5	14,609,674
15292	A_43_P14372	-0.590	0.083	2.6E-06	-0.853	0.074	4.2E-09		ENSRNOG00000031769	5	16,845,631

Disease Models & Mechanisms • Supplementary information

4766	A_44_P775787	-1.410	0.199	2.6E-06	-1.893	0.178	1.2E-08	LOC500420	ENSRNOG00000028185	5	35,902,262
9389	A_44_P1041460	-2.122	0.193	7.3E-09	-2.444	0.172	1.9E-10	Rragd	ENSRNOG00000007331	5	48,224,994
16552	A_44_P1060444	-0.573	0.096	2.1E-05	-0.640	0.086	1.5E-06	RGD1305158	ENSRNOG00000023035	5	50,239,458
28980	A_64_P101386	-0.640	0.079	4.9E-07	-0.565	0.071	5.9E-07	RGD1305158	ENSRNOG00000023035	5	50,239,458
21588	A_42_P680570	-0.942	0.148	9.2E-06	-0.687	0.132	8.7E-05	Nfx1	ENSRNOG00000009015	5	57,291,156
321	A_42_P698240	-1.571	0.113	2.6E-10	-1.652	0.101	2.3E-11	Ptgr1	ENSRNOG00000015072	5	76,129,441
13424	A_44_P175822	-0.907	0.097	6.8E-08	-0.618	0.086	2.3E-06	Hdhd3	ENSRNOG00000015195	5	78,361,647
29448	A_64_P090543	-0.647	0.066	3.5E-08	-0.657	0.059	5.9E-09	Hdhd3	ENSRNOG00000015195	5	78,361,647
13242	A_44_P1040796	1.850	0.073	2.6E-14	1.898	0.065	3.1E-15	Atp6v1g1	ENSRNOG00000008163	5	79,367,663
12871	A_44_P438675	0.865	0.135	8.5E-06	1.442	0.120	2.2E-09	Pappa	ENSRNOG00000033527	5	80,920,568
1289	A_42_P528537	1.364	0.263	9.0E-05	1.212	0.235	9.7E-05	Cdkn2b	ENSRNOG00000006735	5	107,857,320
19065	A_64_P138420	0.916	0.149	1.4E-05	1.664	0.133	1.2E-09	Cldn19	ENSRNOG00000007922	5	138,300,107
20248	A_64_P138415	2.383	0.271	2.8E-07	3.523	0.271	1.5E-09	Cldn19	ENSRNOG00000007922	5	138,300,107
7584	A_64_P055067	-0.849	0.113	1.3E-06	-1.106	0.101	8.6E-09	Ppcs	ENSRNOG00000008572	5	138,470,096
8503	A_44_P361357	0.699	0.115	1.7E-05	0.549	0.103	7.0E-05		ENSRNOG00000058461	5	145,079,803
29067	A_64_P092971	-0.808	0.097	3.3E-07	-1.364	0.087	3.9E-11	Fndc5	ENSRNOG00000030238	5	147,323,240
17989	A_44_P943514	0.484	0.080	1.7E-05	0.455	0.071	9.3E-06	Rbbp4	ENSRNOG00000021492	5	147,535,240
22186	A_64_P001401	0.820	0.126	7.7E-06	0.661	0.113	2.5E-05	Tinagl1	ENSRNOG00000013179	5	148,392,697
29934	A_64_P110798	-1.599	0.160	2.9E-08	-1.877	0.143	5.9E-10	Serinc2	ENSRNOG00000012989	5	148,492,232
12678	A_44_P262091	-2.094	0.324	8.0E-06	-2.120	0.290	1.8E-06	Fabp3	ENSRNOG00000012879	5	148,528,725
23071	A_64_P063696	-1.131	0.114	3.3E-08	-1.126	0.102	7.4E-09	Ptpru	ENSRNOG00000013515	5	149,987,910
4986	A_43_P13041	-2.783	0.220	1.0E-09	-4.205	0.197	3.8E-13	Nr0b2	ENSRNOG00000007229	5	151,776,004
23953	A_44_P279208	-0.709	0.133	6.7E-05	-0.699	0.119	2.3E-05	Eif4g3	ENSRNOG00000014368	5	156,396,695
28038	A_64_P052422	-1.513	0.192	6.9E-07	-2.480	0.172	1.4E-10	Otud3	ENSRNOG00000017037	5	157,368,450
22754	A_64_P051064	-0.444	0.079	3.8E-05	-0.599	0.071	2.6E-07	RGD1560286	ENSRNOG00000009183	5	159,662,754
27403	A_64_P052453	-0.678	0.116	2.5E-05	-0.823	0.104	6.4E-07	RGD1560286	ENSRNOG00000009183	5	159,662,754
3322	A_42_P569711	1.272	0.174	1.8E-06	1.236	0.156	6.4E-07	Epha2	ENSRNOG00000009222	5	159,845,774
11232	A_44_P731487	-0.636	0.099	8.7E-06	-0.708	0.089	5.7E-07	Fbxo44	ENSRNOG00000009298	5	164,971,903
28901	A_44_P429298	-2.217	0.259	2.4E-07	-2.894	0.232	1.2E-09	Tnfrsf9	ENSRNOG00000036942	5	168,009,393
11565	A_64_P127337	1.358	0.222	1.5E-05	1.069	0.199	6.3E-05	Per3	ENSRNOG00000018413	5	168,123,030
15177	A_64_P119405	1.274	0.208	1.5E-05	0.973	0.186	8.4E-05	Per3	ENSRNOG00000018413	5	168,123,030
8236	A_42_P586154	0.678	0.122	4.5E-05	0.698	0.109	9.3E-06	Vwa1	ENSRNOG00000018338	5	173,222,440
10772	A_64_P013112	-1.612	0.231	4.4E-06	-2.073	0.231	2.0E-07	Sult6b1	ENSRNOG00000049335	6	1,493,670

Disease Models & Mechanisms • Supplementary information

14831	A_44_P340316	-0.833	0.106	6.9E-07	-0.633	0.094	5.2E-06	RGD1311578	ENSRNOG00000005279	6	1,534,594
26699	A_64_P028551	-0.734	0.131	4.1E-05	-0.817	0.117	3.3E-06	RGD1311578	ENSRNOG00000005279	6	1,534,594
3693	A_44_P840715	1.000	0.129	8.9E-07	0.645	0.116	4.3E-05	Galm	ENSRNOG00000007023	6	2,808,841
17323	A_44_P119486	1.719	0.298	6.6E-05	2.161	0.365	5.1E-05	Plekhh2	ENSRNOG00000005124	6	7,793,735
18372	A_64_P017363	-0.766	0.088	1.8E-07	-0.759	0.078	4.5E-08	Slc30a3	ENSRNOG00000006204	6	26,642,783
6402	A_43_P17213	-1.644	0.205	5.7E-07	-2.291	0.184	1.2E-09	Abhd1	ENSRNOG00000025689	6	26,792,808
2913	A_64_P036900	-0.739	0.081	1.1E-07	-0.482	0.073	6.0E-06	Kcnk3	ENSRNOG00000009790	6	27,190,132
24275	A_44_P413979	0.482	0.080	1.8E-05	0.828	0.071	3.6E-09	Rab10	ENSRNOG00000047088	6	27,721,120
4161	A_64_P076450	-0.564	0.106	6.9E-05	-0.955	0.095	2.6E-08	Pomc	ENSRNOG00000012686	6	28,382,962
3618	A_44_P377278	-1.883	0.225	3.1E-07	-1.491	0.201	1.5E-06	Adcy3	ENSRNOG00000003999	6	28,571,351
1796	A_42_P525962	-1.107	0.078	1.7E-10	-1.583	0.069	1.4E-13	RGD1561792	ENSRNOG00000004059	6	28,663,602
4792	A_42_P570241	-0.574	0.111	9.3E-05	-0.551	0.099	4.4E-05	Nampt	ENSRNOG00000009754	6	52,122,085
27573	A_44_P945846	1.077	0.164	1.3E-05	1.556	0.178	4.9E-07	Twistnb	ENSRNOG00000010750	6	52,751,106
12252	A_64_P145433	0.547	0.098	4.2E-05	0.535	0.088	1.6E-05		ENSRNOG00000004342	6	55,001,464
18239	A_44_P504133	1.328	0.222	2.0E-05	2.585	0.199	6.7E-10	Tmem195	ENSRNOG00000023116	6	56,846,789
4189	A_64_P098703	-0.521	0.063	3.8E-07	-0.605	0.057	1.1E-08	LOC288913	ENSRNOG00000053727	6	69,619,295
6785	A_42_P483463	-0.420	0.074	3.4E-05	-0.575	0.066	1.8E-07	Ap4s1	ENSRNOG00000005765	6	72,461,977
24462	A_64_P071223	-0.386	0.075	1.0E-04	-0.502	0.067	1.4E-06	Psma3l	ENSRNOG00000007851	6	93,423,002
28435	A_64_P057261	-0.733	0.088	3.6E-07	-0.585	0.079	1.6E-06	Synj2bp	ENSRNOG00000006399	6	105,261,549
8745	A_44_P260589	-1.354	0.180	1.2E-06	-0.925	0.161	3.0E-05		ENSRNOG00000009448	6	107,245,820
12379	A_44_P417429	-0.450	0.074	1.6E-05	-0.652	0.066	3.6E-08	Coq6	ENSRNOG00000011164	6	108,076,306
25368	A_64_P031035	1.510	0.139	9.1E-09	1.545	0.124	1.3E-09	Gstz1	ENSRNOG00000047708	6	111,176,798
474	A_42_P799159	-0.481	0.078	1.4E-05	-0.884	0.070	9.7E-10	LOC688717	ENSRNOG00000012314	6	111,476,768
3674	A_64_P164400	-0.501	0.088	3.6E-05	-0.822	0.079	1.7E-08	LOC688717	ENSRNOG00000012314	6	111,476,768
13736	A_44_P455065	-0.891	0.079	5.1E-09	-0.983	0.071	2.4E-10	Ppp2r5c	ENSRNOG00000004973	6	134,804,141
22919	A_64_P056406	-0.888	0.087	2.3E-08	-0.945	0.078	1.9E-09	Ppp2r5c	ENSRNOG00000004973	6	134,804,141
13877	A_64_P124863	-0.569	0.089	8.8E-06	-0.669	0.079	2.9E-07	RGD1304719	ENSRNOG00000011542	6	136,185,487
28369	A_64_P148906	-4.601	0.599	9.6E-07	-3.860	0.536	2.2E-06	Aspg	ENSRNOG00000012843	6	136,682,126
1529	A_42_P742797	-0.599	0.089	5.1E-06	-0.435	0.080	5.4E-05	Brf1	ENSRNOG00000014595	6	137,808,303
1701	A_64_P145857	-0.786	0.089	1.6E-07	-0.788	0.080	3.3E-08	LOC100359980	ENSRNOG00000028837	7	2,912,491
9961	A_64_P030554	-0.589	0.076	8.7E-07	-0.383	0.068	3.8E-05	Mbd3	ENSRNOG00000028956	7	12,179,203
7110	A_44_P236761	-1.497	0.124	2.0E-09	-1.749	0.111	3.9E-11	Rasal3	ENSRNOG00000006167	7	14,418,950
10600	A_64_P099017	-1.956	0.173	5.1E-09	-1.939	0.155	1.2E-09	Cyp4f17	ENSRNOG00000062306	7	14,529,483

9077	A_64_P091460	0.652	0.124	8.0E-05	1.238	0.111	6.2E-09		ENSRNOG00000048170	7	20,075,974
24192	A_64_P043738	-0.745	0.086	1.9E-07	-0.589	0.077	9.7E-07	Mrpl42	ENSRNOG00000042740	7	36,597,462
24727	A_64_P158133	-0.623	0.088	2.6E-06	-0.523	0.079	5.7E-06	Rap1b	ENSRNOG00000007048	7	60,860,990
1448	A_42_P669907	0.739	0.136	5.8E-05	0.884	0.122	2.0E-06	Slc16a7	ENSRNOG00000007839	7	68,512,397
21486	A_64_P018497	-0.490	0.071	3.9E-06	-0.496	0.064	8.4E-07	Mterfd1	ENSRNOG00000004492	7	71,293,823
5860	A_64_P047206	-1.093	0.162	5.0E-06	-1.682	0.145	3.6E-09	Depdc6	ENSRNOG00000004328	7	94,795,214
12545	A_64_P039737	1.506	0.260	2.8E-05	2.089	0.233	1.3E-07	Scx	ENSRNOG00000021812	7	117,519,075
30277	A_64_P142942	0.913	0.145	1.1E-05	0.666	0.129	9.8E-05	Scx	ENSRNOG00000021812	7	117,519,075
12493	A_44_P537024	-0.476	0.083	3.2E-05	-0.697	0.074	7.0E-08	Gpr172a	ENSRNOG00000032561	7	117,605,050
13898	A_44_P445070	-0.484	0.077	1.2E-05	-0.545	0.069	7.1E-07	Lgals1	ENSRNOG00000009884	7	120,153,184
7934	A_44_P309650	-0.901	0.135	5.4E-06	-0.998	0.121	3.7E-07	Nol12	ENSRNOG00000010209	7	120,161,091
6095	A_64_P039165	0.761	0.096	6.3E-07	0.647	0.086	1.2E-06	Gcat	ENSRNOG00000055408	7	120,263,054
21107	A_44_P758203	-0.422	0.080	8.2E-05	-0.414	0.072	3.0E-05	Srek1ip1	ENSRNOG00000047506	7	122,160,171
10558	A_43_P12905	-0.798	0.141	4.5E-05	-0.894	0.141	1.3E-05	Bik	ENSRNOG00000010359	7	124,391,432
3165	A_42_P524610	0.451	0.085	7.0E-05	0.484	0.076	9.3E-06	Pphln1	ENSRNOG00000022778	7	134,603,121
22334	A_64_P113640	-0.810	0.111	1.8E-06	-0.775	0.099	7.7E-07	Nckap5l	ENSRNOG00000056678	7	141,093,924
1937	A_42_P751562	0.837	0.162	9.6E-05	0.915	0.145	1.1E-05	Mettl7a	ENSRNOG00000001376	7	141,973,553
28027	A_64_P110526	-1.146	0.211	5.8E-05	-1.550	0.189	4.2E-07	LOC683720	ENSRNOG00000058340	7	143,538,579
19949	A_64_P070736	0.782	0.136	3.0E-05	0.930	0.122	1.0E-06	Eif4b	ENSRNOG00000010103	7	143,679,617
6078	A_42_P589680	-0.682	0.079	2.1E-07	-0.591	0.071	3.2E-07	Tarbp2	ENSRNOG00000042355	7	144,121,744
3769	A_43_P12287	2.135	0.122	8.2E-12	2.029	0.109	3.3E-12	Ppp1r1a	ENSRNOG00000036827	7	145,154,131
25629	A_64_P145261	2.073	0.113	3.8E-12	2.070	0.101	7.0E-13	Ppp1r1a	ENSRNOG00000036827	7	145,154,131
2497	A_64_P064621	0.933	0.130	3.3E-06	0.740	0.124	2.5E-05	Trpc6	ENSRNOG00000006324	8	6,811,543
24267	A_64_P120759	-1.103	0.174	1.0E-05	-1.311	0.156	2.9E-07		ENSRNOG00000008990	8	13,109,487
17711	A_44_P119527	-1.588	0.176	1.2E-07	-1.471	0.158	7.5E-08	Med17	ENSRNOG00000051989	8	13,526,025
8626	A_44_P100886	1.676	0.142	1.4E-07	1.662	0.208	6.7E-06		ENSRNOG00000010999	8	13,909,188
11775	A_44_P518434	-1.274	0.140	1.0E-07	-1.217	0.125	4.2E-08	Mrpl4	ENSRNOG00000020659	8	22,021,213
19561	A_64_P051647	-1.079	0.098	7.5E-09	-0.838	0.088	5.4E-08	Pde4a	ENSRNOG00000020828	8	22,189,600
22156	A_64_P121123	-1.328	0.145	9.1E-08	-0.935	0.129	2.1E-06	Pde4a	ENSRNOG00000020828	8	22,189,600
20324	A_64_P114595	-0.813	0.138	2.3E-05	-1.007	0.123	4.3E-07	Atg4d	ENSRNOG00000047625	8	22,291,445
1575	A_44_P238246	0.650	0.095	4.1E-06	1.152	0.085	3.6E-10	Ilf3	ENSRNOG00000022741	8	22,402,890
28744	A_43_P11181	-0.391	0.075	8.6E-05	-0.548	0.067	4.4E-07	Elof1	ENSRNOG00000014284	8	23,130,877
1785	A_42_P553201	-1.233	0.117	1.3E-08	-0.704	0.105	4.9E-06	Acp5	ENSRNOG00000046261	8	23,148,396

Disease Models & Mechanisms • Supplementary information

13689	A_42_P820829	-0.715	0.111	7.8E-06	-0.527	0.099	6.9E-05	Oaf	ENSRNOG00000009243	8	47,529,689
3609	A_44_P122206	-0.890	0.144	1.3E-05	-1.510	0.129	2.9E-09	Timm8b	ENSRNOG00000009888	8	55,037,750
3560	A_42_P624403	-0.407	0.072	3.7E-05	-0.466	0.064	2.0E-06	Fdx1	ENSRNOG00000012123	8	56,393,233
21973	A_44_P272353	-0.757	0.092	3.8E-07	-0.716	0.082	1.8E-07	LOC367117	ENSRNOG00000031132	8	86,286,991
5042	A_64_P093234	-0.587	0.106	4.8E-05	-1.248	0.095	5.9E-10	Col12a1	ENSRNOG00000058470	8	87,158,368
3358	A_43_P10191	-0.493	0.089	4.6E-05	-0.716	0.080	1.2E-07	Rnf7	ENSRNOG00000011663	8	104,335,729
4841	A_44_P373483	-1.805	0.149	4.1E-09	-1.374	0.149	1.5E-07	Slco2a1	ENSRNOG00000009005	8	111,495,331
21381	A_43_P16567	-1.832	0.199	8.9E-08	-2.056	0.178	3.7E-09	Glyctk	ENSRNOG00000046307	8	114,853,103
23112	A_42_P834090	-0.826	0.106	7.7E-07	-0.712	0.095	1.2E-06	LOC686298	ENSRNOG00000046535	8	114,892,910
18260	A_43_P16646	-0.525	0.085	1.3E-05	-0.921	0.076	1.7E-09	Tex264	ENSRNOG00000013201	8	115,388,266
29684	A_44_P1024703	-0.769	0.130	2.3E-05	-1.026	0.117	1.6E-07	Cyb561d2	ENSRNOG00000021887	8	116,297,850
20909	A_44_P509845	-0.643	0.087	1.5E-06	-0.620	0.077	5.6E-07	Dag1	ENSRNOG00000019400	8	116,993,193
23340	A_44_P550228	0.815	0.144	3.6E-05	1.030	0.129	5.7E-07	Lamb2	ENSRNOG00000047768	8	117,268,337
3435	A_42_P573412	0.871	0.142	1.5E-05	0.664	0.127	8.7E-05	Spink8	ENSRNOG00000037199	8	117,906,014
23743	A_44_P126027	-2.101	0.181	7.0E-09	-1.388	0.181	1.5E-06	Pfkfb4	ENSRNOG00000048130	8	118,392,773
18691	A_64_P057548	-0.697	0.081	2.3E-07	-0.841	0.073	3.6E-09	Cmc1	ENSRNOG00000010149	8	126,495,347
8158	A_42_P671285	0.940	0.131	2.2E-06	1.491	0.117	8.8E-10	Ctdspl	ENSRNOG00000046257	8	127,702,534
14049	A_44_P625333	0.816	0.125	6.8E-06	1.242	0.111	6.2E-09	Ctdspl	ENSRNOG00000046257	8	127,702,534
26317	A_44_P431661	1.438	0.218	6.4E-06	1.509	0.195	9.0E-07	Vill	ENSRNOG00000011446	8	127,735,258
1637	A_42_P586141	-1.117	0.125	1.3E-07	-1.131	0.112	2.5E-08	Plcd1	ENSRNOG00000032238	8	127,782,070
20183	A_44_P306507	-0.970	0.122	6.3E-07	-1.112	0.109	2.2E-08	Higd1a	ENSRNOG00000019428	8	130,491,998
24536	A_64_P118187	0.843	0.127	5.9E-06	1.253	0.114	7.2E-09	Hdgf2	ENSRNOG00000049142	9	10,991,820
13374	A_44_P405628	-0.665	0.100	5.4E-06	-0.715	0.089	5.4E-07	Ubxn6	ENSRNOG00000048509	9	10,995,198
8822	A_64_P160484	-0.989	0.137	2.0E-06	-1.253	0.122	2.0E-08	Trem3	ENSRNOG00000013748	9	14,724,093
16969	A_64_P045957	-0.597	0.068	1.6E-07	-0.928	0.061	6.1E-11	Mrpl14	ENSRNOG00000019734	9	17,698,569
4274	A_44_P428507	-1.261	0.119	1.3E-08	-1.599	0.106	7.8E-11	Nfkbie	ENSRNOG00000019907	9	17,835,240
2347	A_44_P111507	-1.756	0.177	5.6E-08	-2.377	0.177	9.3E-10	Tcte1	ENSRNOG00000019961	9	17,866,927
17848	A_64_P004231	1.356	0.171	6.2E-07	2.127	0.153	2.4E-10	Gsta5	ENSRNOG00000056847	9	27,402,381
3838	A_42_P624195	1.590	0.219	1.9E-06	1.895	0.195	4.4E-08	Gsta3	ENSRNOG00000013484	9	27,452,902
11587	A_64_P139113	-0.456	0.086	7.1E-05	-0.441	0.077	3.1E-05	Arpc5l	ENSRNOG00000016444	9	49,795,936
25117	A_64_P060967	0.995	0.087	4.4E-09	0.921	0.078	2.7E-09	Lancl1	ENSRNOG00000013557	9	74,048,244
8097	A_44_P1031737	0.761	0.134	3.5E-05	0.753	0.120	1.1E-05	Pecr	ENSRNOG00000055295	9	79,630,536
17524	A_44_P384090	0.890	0.170	8.1E-05	1.148	0.152	1.2E-06	Des	ENSRNOG00000019810	9	82,556,573

Disease Models & Mechanisms • Supplementary information

18795	A_43_P10901	1.889	0.189	2.9E-08	2.520	0.169	8.9E-11	Itm2c	ENSRNOG00000017359	9	92,916,469
3751	A_42_P471768	-0.641	0.074	2.1E-07	-0.657	0.066	3.3E-08	Eif4e2	ENSRNOG00000019634	9	94,310,921
5606	A_44_P467591	-1.808	0.243	1.4E-06	-3.265	0.217	7.8E-11	Neu2	ENSRNOG00000016962	9	94,702,129
7344	A_64_P010174	0.920	0.133	3.6E-06	0.821	0.119	3.7E-06	Man2a1	ENSRNOG00000015439	9	112,293,388
8020	A_64_P054942	0.450	0.073	1.4E-05	0.491	0.065	1.2E-06	Wash2	ENSRNOG00000053306	9	113,918,858
9804	A_64_P148368	1.173	0.149	6.9E-07	0.954	0.133	2.3E-06	RGD1308319	ENSRNOG00000025527	9	114,619,711
11494	A_44_P123492	-1.026	0.176	2.5E-05	-0.913	0.157	2.7E-05	Epb41l3	ENSRNOG00000016724	9	117,538,009
6655	A_64_P105228	0.718	0.129	4.5E-05	0.903	0.116	7.8E-07	RGD1309537	ENSRNOG00000015278	9	119,332,967
9876	A_64_P105231	0.678	0.122	4.2E-05	0.769	0.109	2.7E-06	RGD1309537	ENSRNOG00000015278	9	119,332,967
970	A_44_P163018	-0.551	0.070	7.2E-07	-0.484	0.063	9.1E-07	Nubp1	ENSRNOG00000002574	10	5,344,121
22702	A_64_P088038	-0.519	0.100	9.5E-05	-0.739	0.090	3.9E-07	Tmem186	ENSRNOG00000027087	10	7,077,488
22463	A_44_P142850	-0.826	0.113	1.7E-06	0.984	0.101	4.0E-08	Fam86a	ENSRNOG00000002876	10	10,530,365
29102	A_42_P475151	0.673	0.123	5.1E-05	1.031	0.110	6.9E-08	Rogdi	ENSRNOG00000003125	10	10,761,477
12289	A_43_P12438	-0.359	0.069	9.0E-05	-0.804	0.062	6.8E-10	Hmox2	ENSRNOG00000003773	10	11,035,484
24015	A_44_P331119	-0.466	0.085	4.8E-05	-0.622	0.076	4.0E-07	Spsb3	ENSRNOG00000015300	10	14,248,399
10341	A_64_P006373	-0.478	0.071	4.6E-06	-0.689	0.063	8.4E-09	Mrps34	ENSRNOG00000015479	10	14,257,001
21059	A_64_P066501	0.963	0.131	1.7E-06	1.243	0.117	1.3E-08	Gng13	ENSRNOG00000039350	10	15,088,935
23671	A_64_P062663	0.944	0.132	2.4E-06	1.301	0.118	7.5E-09	Gng13	ENSRNOG00000039350	10	15,088,935
4846	A_44_P114851	-0.766	0.106	2.1E-06	-1.220	0.095	7.8E-10	Tmem8a	ENSRNOG00000020374	10	15,485,905
7281	A_64_P062288	-1.072	0.166	7.9E-06	-0.812	0.148	5.2E-05	Ccdc99	ENSRNOG00000007292	10	19,652,898
14617	A_64_P028561	1.747	0.277	1.1E-05	1.769	0.248	2.4E-06		ENSRNOG00000058024	10	29,806,895
4451	A_44_P1001820	-1.079	0.136	6.1E-07	-1.053	0.121	1.9E-07	Slc22a5	ENSRNOG00000008432	10	39,323,816
11928	A_64_P043746	-0.427	0.065	6.7E-06	-0.604	0.058	1.7E-08	Mrpl22	ENSRNOG00000027039	10	43,601,689
5028	A_64_P015236	-0.683	0.108	1.1E-05	-1.102	0.097	4.7E-09	RGD1306000	ENSRNOG00000022725	10	45,514,878
22884	A_64_P058312	-0.534	0.103	9.4E-05	-0.654	0.092	2.6E-06	RGD1306000	ENSRNOG00000022725	10	45,514,878
27887	A_44_P258277	-0.682	0.106	8.4E-06	-0.608	0.095	8.8E-06	Dhrs7b	ENSRNOG00000005360	10	47,065,850
13618	A_64_P017053	-2.728	0.250	6.7E-08	-1.720	0.204	1.3E-06		ENSRNOG00000004171	10	52,710,862
4290	A_64_P011819	-0.586	0.088	5.4E-06	-0.421	0.078	6.4E-05	Sco1	ENSRNOG00000028699	10	53,595,854
22062	A_64_P112579	1.396	0.185	1.2E-06	1.031	0.165	1.2E-05	Slc16a11	ENSRNOG00000018748	10	56,822,756
2308	A_44_P993464	-0.318	0.061	9.0E-05	-0.441	0.055	5.3E-07	Med11	ENSRNOG00000019384	10	57,057,608
23056	A_64_P032833	-0.565	0.103	4.9E-05	-0.704	0.092	9.9E-07	Aspa	ENSRNOG00000019659	10	59,888,200
7399	A_44_P332734	-0.545	0.105	8.7E-05	-0.601	0.094	8.8E-06	LOC100365943	ENSRNOG00000022652	10	65,835,966
23384	A_64_P016978	1.185	0.163	2.0E-06	0.936	0.146	8.9E-06	Ccl11	ENSRNOG00000007335	10	69,434,941

3550	A_42_P785926	-0.549	0.069	5.7E-07	-0.538	0.061	1.7E-07	Mrps23	ENSRNOG00000010363	10	75,529,009
26348	A_64_P009540	-1.088	0.202	6.3E-05	-1.261	0.181	3.3E-06	Akap1	ENSRNOG00000002373	10	76,166,276
28956	A_43_P12890	-0.966	0.183	7.4E-05	-1.004	0.163	1.4E-05	Akap1	ENSRNOG00000002373	10	76,166,276
8482	A_44_P292487	-1.113	0.086	7.2E-10	-1.009	0.077	5.8E-10	Scpep1	ENSRNOG00000002358	10	76,263,866
1958	A_64_P075938	-0.828	0.157	7.4E-05	-1.387	0.140	3.3E-08	Cox11	ENSRNOG00000052096	10	78,111,050
23141	A_64_P068570	-0.877	0.144	1.6E-05	-1.119	0.128	1.9E-07	Cox11	ENSRNOG00000052096	10	78,168,715
15574	A_64_P057453	-1.108	0.101	7.6E-09	-0.467	0.090	9.2E-05	LOC303448	ENSRNOG00000054719	10	78,690,857
6815	A_44_P407138	-0.716	0.082	1.9E-07	-0.902	0.074	1.6E-09	Nme1	ENSRNOG00000002693	10	81,666,523
5476	A_64_P150256	-2.105	0.172	1.6E-09	-2.302	0.154	8.3E-11	Epn3	ENSRNOG00000003284	10	82,229,140
14953	A_64_P021606	-2.380	0.192	1.4E-09	-3.235	0.172	2.6E-12	Mycbpap	ENSRNOG00000042912	10	82,252,963
3474	A_64_P063871	-1.444	0.219	6.3E-06	-1.293	0.196	6.2E-06	Phospho1	ENSRNOG00000005569	10	83,636,518
27361	A_64_P049761	-0.923	0.109	2.8E-07	-0.739	0.098	1.2E-06	Nfe2l1	ENSRNOG00000008830	10	84,698,886
3940	A_42_P453894	-0.519	0.080	7.8E-06	-0.662	0.072	8.4E-08	RGD1306682	ENSRNOG00000050874	10	84,847,857
15673	A_64_P097994	-0.612	0.074	3.6E-07	-0.504	0.066	1.0E-06	Npepps	ENSRNOG00000023095	10	85,222,861
11808	A_64_P024482	-0.678	0.080	2.6E-07	-0.783	0.071	8.0E-09	Cisd3	ENSRNOG00000036894	10	85,628,491
28614	A_44_P1038655	-2.330	0.307	1.1E-06	-2.029	0.274	1.6E-06	Ttc25	ENSRNOG00000017473	10	88,459,490
29794	A_43_P12209	-0.862	0.159	5.6E-05	-0.879	0.142	1.3E-05	Stat5b	ENSRNOG00000019075	10	88,754,829
10835	A_42_P652586	-0.982	0.127	9.1E-07	-1.446	0.114	9.5E-10	Stat5a	ENSRNOG00000019496	10	88,764,732
6804	A_64_P150509	-1.360	0.215	1.1E-05	-2.176	0.193	5.2E-09	Ccr10	ENSRNOG00000020275	10	89,088,993
24388	A_44_P377156	-1.065	0.153	3.3E-06	-1.219	0.137	1.4E-07	Cntnap1	ENSRNOG00000020277	10	89,089,646
24058	A_64_P141424	-0.700	0.086	4.6E-07	-0.934	0.077	1.8E-09	Ccdc56	ENSRNOG00000020487	10	89,199,736
14741	A_64_P100516	0.668	0.101	5.9E-06	0.468	0.090	9.0E-05	Aoc3	ENSRNOG00000051307	10	89,251,370
12992	A_64_P051393	-1.926	0.166	3.6E-09	-1.547	0.149	1.7E-08	Pyy	ENSRNOG00000020877	10	90,049,112
25120	A_64_P051392	-1.342	0.157	2.4E-07	-1.158	0.140	3.9E-07	Pyy	ENSRNOG00000020877	10	90,049,112
21785	A_44_P130785	-0.468	0.077	1.7E-05	-0.490	0.069	2.6E-06	Slc25a39	ENSRNOG00000020994	10	90,356,242
12629	A_64_P073629	-0.578	0.094	1.5E-05	-0.644	0.084	1.1E-06	Magmas	ENSRNOG00000028427	10	91,752,934
7748	A_42_P612977	-1.157	0.142	4.6E-07	-0.782	0.127	1.4E-05	Cyb561	ENSRNOG00000007433	10	94,147,556
15636	A_64_P143770	-0.984	0.127	8.3E-07	-0.640	0.113	3.7E-05		ENSRNOG00000030224	10	102,289,837
8362	A_64_P055738	0.515	0.078	6.2E-06	0.923	0.070	5.3E-10	Itgb4	ENSRNOG00000005580	10	104,523,996
3531	A_64_P059385	0.634	0.105	1.7E-05	0.592	0.094	1.0E-05	Acox1	ENSRNOG00000008755	10	104,748,050
22560	A_64_P157691	-2.001	0.197	2.3E-08	-1.721	0.176	3.9E-08	RGD1559482	ENSRNOG00000048771	10	104,952,237
25707	A_44_P436597	-0.551	0.094	2.6E-05	-0.890	0.084	1.4E-08	RGD1306284	ENSRNOG00000000249	10	105,787,935
4081	A_64_P085405	-1.608	0.140	4.1E-09	-1.012	0.125	5.1E-07	9-Sep	ENSRNOG00000002807	10	106,264,434

Disease Models & Mechanisms • Supplementary information

22449	A_42_P539095	-0.895	0.066	3.8E-10	-0.876	0.059	1.0E-10	LOC688311	ENSRNOG00000049235	10	109,639,054
18404	A_43_P13256	1.550	0.183	2.8E-07	1.342	0.164	4.3E-07	Dcxr	ENSRNOG00000050315	10	109,909,646
17254	A_64_P138560	-1.015	0.148	4.1E-06	-0.992	0.133	1.4E-06	Narf	ENSRNOG00000061691	10	110,125,109
15351	A_64_P072973	1.950	0.156	1.2E-09	2.174	0.140	4.6E-11	Kcne2	ENSRNOG00000029811	11	32,440,237
23285	A_44_P884080	-0.478	0.089	6.1E-05	-0.435	0.079	5.0E-05	LOC100174909	ENSRNOG00000039877	11	32,450,587
26800	A_64_P116606	1.291	0.202	1.7E-05	1.588	0.218	4.2E-06	Rcan1	ENSRNOG00000001979	11	32,550,539
3179	A_42_P554722	-0.701	0.119	2.3E-05	-0.808	0.107	1.1E-06	Pigp	ENSRNOG00000039850	11	34,598,275
1366	A_64_P057975	0.876	0.109	5.4E-07	1.202	0.098	1.5E-09	Hmgn1	ENSRNOG00000050978	11	36,479,868
6802	A_42_P625419	0.940	0.172	5.2E-05	1.154	0.154	1.3E-06	RGD1565927	ENSRNOG00000001645	11	45,124,423
6884	A_42_P732251	-0.935	0.163	3.0E-05	-0.925	0.145	9.7E-06	Phldb2	ENSRNOG00000002171	11	57,404,196
22903	A_43_P12844	-0.355	0.068	8.7E-05	-1.050	0.061	1.0E-11	Cox17	ENSRNOG00000038951	11	64,968,437
29503	A_64_P124200	1.293	0.240	6.0E-05	1.126	0.214	7.9E-05	Gp1bb	ENSRNOG00000046981	11	86,520,992
2679	A_64_P160658	-0.714	0.093	9.3E-07	-0.438	0.083	7.6E-05	Txnrd2	ENSRNOG00000001890	11	86,716,063
16895	A_64_P038926	1.281	0.194	6.1E-06	1.345	0.173	8.3E-07	Prodh	ENSRNOG00000000281	11	87,058,616
24093	A_44_P244257	0.987	0.151	6.9E-06	0.780	0.135	2.9E-05	Fgd4	ENSRNOG00000059491	11	88,699,222
9868	A_64_P013239	-0.481	0.078	1.3E-05	-0.584	0.069	2.9E-07	Higd2a	ENSRNOG00000001071	12	4,546,287
12967	A_64_P047002	0.895	0.137	6.9E-06	0.813	0.122	5.8E-06	Fry	ENSRNOG00000000894	12	5,682,608
16115	A_44_P267068	-0.465	0.078	1.9E-05	-0.427	0.069	1.4E-05	Nudt1	ENSRNOG00000001260	12	16,395,029
3604	A_42_P746109	-0.846	0.071	2.4E-09	-0.638	0.063	2.6E-08	Asmtl	ENSRNOG00000028166	12	18,531,990
1594	A_64_P024429	0.583	0.107	5.6E-05	1.081	0.096	5.4E-09	Il3ra	ENSRNOG00000001325	12	18,540,166
19145	A_64_P081501	1.255	0.163	9.3E-07	1.561	0.146	1.1E-08	Mcm7	ENSRNOG00000001349	12	19,314,016
23117	A_42_P540609	-1.633	0.155	2.6E-08	-1.668	0.155	1.9E-08	LOC680711	ENSRNOG00000031343	12	19,440,501
24237	A_64_P194847	-1.462	0.187	1.2E-06	-1.797	0.187	9.0E-08		ENSRNOG00000027061	12	19,512,591
25622	A_64_P119299	-1.703	0.217	1.1E-06	-1.840	0.217	4.2E-07		ENSRNOG00000027061	12	19,512,591
21845	A_44_P1012866	-0.550	0.072	9.6E-07	-0.611	0.064	5.5E-08	RGD1309735	ENSRNOG00000027028	12	19,573,909
1742	A_64_P144034	1.243	0.167	4.0E-05	1.915	0.167	1.2E-06	LOC363894	ENSRNOG00000045875	12	20,814,122
19679	A_64_P045833	-1.142	0.176	7.4E-06	-1.517	0.157	4.6E-08		ENSRNOG00000050128	12	21,450,204
15839	A_64_P032265	0.707	0.120	2.3E-05	1.079	0.107	2.7E-08		ENSRNOG00000033017	12	21,678,580
21526	A_44_P579382	-1.327	0.188	2.7E-06	-2.279	0.168	3.6E-10	Zcwpw1	ENSRNOG00000024425	12	21,720,094
25316	A_64_P053855	-0.498	0.082	1.7E-05	-0.680	0.073	8.1E-08	Znhit1	ENSRNOG00000001418	12	22,726,643
25740	A_44_P1041034	-0.600	0.080	1.2E-06	-0.650	0.071	1.0E-07	Polr2j	ENSRNOG00000001430	12	23,592,774
18878	A_44_P992908	1.628	0.263	1.3E-05	1.294	0.235	4.9E-05	Hspb1	ENSRNOG00000023546	12	23,841,049
17894	A_64_P069394	0.947	0.144	6.7E-06	1.283	0.129	3.1E-08	Styxl1	ENSRNOG00000023366	12	23,954,574

Disease Models & Mechanisms • Supplementary information

6654	A_44_P1009552	0.974	0.182	6.5E-05	0.914	0.163	3.9E-05	Gtf2ird1	ENSRNOG00000001478	12	25,264,192
4941	A_42_P589190	1.553	0.266	2.5E-05	2.949	0.238	1.3E-09	Aacs	ENSRNOG00000000967	12	36,555,694
3175	A_44_P1030714	1.490	0.147	2.4E-08	2.102	0.132	3.2E-11	Ccdc92	ENSRNOG00000021691	12	37,211,316
13761	A_64_P135202	-0.879	0.086	2.2E-08	-0.806	0.077	1.5E-08	Snrnp35	ENSRNOG00000001060	12	37,538,403
24243	A_44_P184650	-0.773	0.083	8.2E-08	-0.730	0.075	3.9E-08	Rilpl2	ENSRNOG00000001061	12	37,544,918
2306	A_42_P630463	-1.026	0.197	8.7E-05	-2.218	0.176	1.1E-09	Abcb9	ENSRNOG00000001082	12	37,923,528
6667	A_44_P187005	-1.246	0.195	9.1E-06	-1.851	0.174	1.2E-08	Abcb9	ENSRNOG00000001082	12	37,923,528
2579	A_64_P024060	-0.437	0.073	1.9E-05	-0.489	0.065	1.3E-06		ENSRNOG00000001106	12	38,116,919
29139	A_44_P838992	-2.382	0.189	1.1E-09	-1.494	0.169	1.5E-07	LOC687609	ENSRNOG00000042607	12	38,880,377
2210	A_64_P023670	0.527	0.087	1.6E-05	0.897	0.077	3.6E-09	Aldh2	ENSRNOG00000001344	12	40,466,495
19071	A_43_P17480	-0.897	0.169	7.1E-05	-0.971	0.151	8.6E-06	RGD1306772	ENSRNOG00000037720	12	41,486,076
17976	A_64_P106176	0.479	0.082	2.6E-05	0.454	0.073	1.3E-05		ENSRNOG00000001120	12	43,503,456
6196	A_44_P686406	1.149	0.165	3.4E-06	1.064	0.148	2.2E-06	RGD1562310	ENSRNOG00000001123	12	43,940,798
741	A_42_P548796	-0.875	0.130	5.1E-06	-0.623	0.117	6.7E-05	Tsn	ENSRNOG00000002319	13	34,251,650
20428	A_44_P272253	-0.496	0.088	3.6E-05	-0.952	0.078	1.8E-09	LOC288978	ENSRNOG00000045570	13	36,156,182
23346	A_64_P013147	0.865	0.161	6.4E-05	1.032	0.144	2.3E-06	Prelp	ENSRNOG00000003120	13	50,761,306
10145	A_64_P103707	-0.708	0.098	2.2E-06	-0.830	0.088	6.4E-08	Ndufv3	ENSRNOG00000027593	13	51,297,621
28935	A_64_P141541	-0.616	0.078	6.9E-07	-0.795	0.070	4.7E-09	Ndufv3	ENSRNOG00000027593	13	51,297,621
16455	A_64_P145820	0.721	0.078	8.7E-08	0.940	0.070	4.1E-10	Gpr37l1	ENSRNOG00000006237	13	51,992,693
19291	A_44_P475444	1.000	0.184	5.6E-05	1.036	0.165	1.1E-05	RGD1564614	ENSRNOG00000042369	13	56,958,549
2005	A_42_P819656	1.339	0.223	1.8E-05	1.385	0.199	3.3E-06	Cfh	ENSRNOG00000030715	13	57,080,549
4627	A_42_P553319	-0.734	0.137	6.6E-05	-0.885	0.123	2.1E-06		ENSRNOG00000004736	13	74,520,634
4122	A_44_P358194	2.817	0.289	4.2E-08	3.061	0.259	2.7E-09	Fmo3	ENSRNOG00000003620	13	80,862,963
19873	A_64_P055216	3.019	0.145	5.3E-13	1.876	0.129	1.3E-10	RGD1309708	ENSRNOG00000002860	13	82,574,966
27423	A_64_P083364	-0.634	0.104	1.6E-05	-0.755	0.093	5.0E-07	Brp44	ENSRNOG00000003150	13	83,681,322
26528	A_64_P016901	0.777	0.102	1.1E-06	1.198	0.091	6.0E-10	Creg1	ENSRNOG00000003291	13	83,972,212
22348	A_44_P1018997	-0.964	0.077	1.3E-09	-0.478	0.069	3.6E-06	Tmco1	ENSRNOG00000003928	13	85,465,792
20771	A_64_P001255	-0.863	0.123	3.0E-06	-0.957	0.110	1.9E-07	Ppox	ENSRNOG00000003567	13	89,654,244
502	A_64_P051346	-1.316	0.135	4.2E-08	-1.195	0.121	3.4E-08	Pex19	ENSRNOG00000057116	13	90,514,362
1369	A_42_P647505	0.555	0.092	1.9E-05	0.870	0.083	1.4E-08		ENSRNOG00000023541	13	98,078,555
43	A_44_P174374	1.120	0.120	7.2E-08	1.310	0.107	1.6E-09	Ephx1	ENSRNOG00000003515	13	99,300,579
2623	A_64_P057526	1.433	0.137	1.5E-08	1.095	0.123	1.3E-07		ENSRNOG00000037928	13	99,335,020
23959	A_44_P177914	0.577	0.089	7.3E-06	0.786	0.079	3.2E-08	Nvl	ENSRNOG00000003629	13	99,468,778

11923	A_44_P313786	-0.538	0.078	4.0E-06	-0.793	0.070	5.1E-09	Tmem14c	ENSRNOG00000022144	13	107,580,629
1758	A_64_P027298	-0.806	0.124	7.8E-06	-0.867	0.111	8.0E-07	Ptpn14	ENSRNOG00000003407	13	108,841,482
9059	A_42_P803590	-1.556	0.105	9.6E-11	-1.424	0.094	6.8E-11	LOC289378	ENSRNOG00000046460	13	109,663,364
3954	A_42_P661065	1.100	0.140	7.0E-07	1.219	0.125	4.1E-08	Ints7	ENSRNOG00000004263	13	110,257,571
10206	A_64_P052464	1.059	0.190	4.3E-05	1.320	0.170	8.5E-07	Ints7	ENSRNOG00000004263	13	110,257,571
18334	A_64_P124502	-0.567	0.108	8.3E-05	-0.597	0.097	1.4E-05	Rcor3	ENSRNOG00000046445	13	110,864,469
3766	A_42_P601802	0.679	0.085	5.7E-07	0.727	0.076	5.2E-08	Crlf2	ENSRNOG00000049828	14	1,461,543
3061	A_44_P454646	-0.612	0.093	6.0E-06	-0.665	0.083	5.4E-07	Fam69a	ENSRNOG00000023533	14	2,789,650
2950	A_42_P683634	1.158	0.157	1.6E-06	1.398	0.140	2.9E-08	Sparcl1	ENSRNOG00000015093	14	6,994,190
10617	A_44_P1043814	-0.670	0.104	8.1E-06	-0.701	0.093	1.2E-06	Hsd17b11	ENSRNOG00000002210	14	7,073,445
4776	A_43_P14648	0.837	0.154	5.4E-05	0.709	0.137	9.6E-05	Art3	ENSRNOG00000002256	14	17,225,389
8045	A_64_P151226	0.855	0.150	3.3E-05	0.952	0.134	2.6E-06	Art3	ENSRNOG00000002256	14	17,225,389
6146	A_64_P144670	-0.841	0.163	9.8E-05	-1.273	0.146	1.9E-07	Usp46	ENSRNOG00000002106	14	36,687,134
11340	A_64_P150662	2.078	0.170	1.7E-09	2.024	0.152	4.9E-10	Corin	ENSRNOG00000002302	14	38,247,791
14100	A_44_P376940	-0.926	0.150	1.3E-05	-0.699	0.134	8.4E-05	Otop1	ENSRNOG00000028423	14	77,263,729
6252	A_64_P074260	-0.908	0.102	1.3E-07	-1.145	0.091	1.1E-09	Letm1	ENSRNOG00000016427	14	82,227,708
2976	A_43_P19289	-0.765	0.101	1.1E-06	-0.750	0.090	3.5E-07	Mtp18	ENSRNOG00000004640	14	84,334,066
10255	A_64_P115903	-0.603	0.092	6.4E-06	-0.481	0.082	2.4E-05	Zmat5	ENSRNOG00000007849	14	84,953,786
16167	A_64_P046754	0.781	0.079	3.6E-08	0.730	0.071	1.9E-08	Igfbp3	ENSRNOG00000061910	14	87,465,374
13521	A_64_P007498	-2.087	0.228	9.7E-08	-3.682	0.204	5.0E-12	Fam161a	ENSRNOG00000009881	14	107,785,029
5167	A_64_P049628	-1.080	0.108	2.7E-08	-1.102	0.096	4.2E-09	Acyp2	ENSRNOG00000042419	14	115,052,450
8650	A_42_P607939	-0.742	0.069	1.0E-08	-0.561	0.062	1.0E-07	Chchd1	ENSRNOG00000009297	15	4,026,637
30150	A_64_P031606	-0.520	0.078	5.5E-06	-0.807	0.070	3.6E-09	Mrps16	ENSRNOG00000006898	15	4,351,292
14168	A_43_P12698	-1.089	0.167	7.1E-06	0.779	0.149	8.5E-05	Lgals3	ENSRNOG00000010645	15	24,141,651
22906	A_44_P465868	-0.830	0.098	2.8E-07	-0.810	0.088	8.7E-08	Dhrs4	ENSRNOG00000018239	15	34,155,504
25572	A_64_P157611	0.899	0.170	7.5E-05	1.445	0.152	5.8E-08	Cpne6	ENSRNOG00000018399	15	34,187,223
4177	A_42_P597308	-0.515	0.083	1.3E-05	-0.680	0.074	9.5E-08	N6amt2	ENSRNOG00000009849	15	37,830,131
21303	A_44_P206091	-0.374	0.072	9.4E-05	-0.447	0.065	3.5E-06	Mrp63	ENSRNOG00000010855	15	38,095,803
9768	A_64_P102498	-1.773	0.266	5.5E-06	-3.282	0.238	2.8E-10	LOC683415	ENSRNOG00000047258	15	47,442,664
24711	A_44_P321176	-1.647	0.209	7.0E-07	-1.080	0.187	2.9E-05	Hr	ENSRNOG00000011427	15	52,241,801
5602	A_64_P153447	-1.551	0.285	6.8E-05	-2.380	0.285	5.1E-07	Dct	ENSRNOG00000008671	15	103,244,494
25871	A_44_P308369	1.223	0.232	7.7E-05	2.139	0.207	1.9E-08		ENSRNOG00000058057	15	104,349,745
15927	A_44_P250594	1.232	0.132	2.4E-07	0.951	0.143	1.1E-05		ENSRNOG00000058057	15	104,393,682

Disease Models & Mechanisms • Supplementary information

12187	A_43_P16710	0.532	0.087	1.5E-05	0.743	0.078	5.4E-08	Ipo5	ENSRNOG00000010989	15	106,257,650
18365	A_43_P14210	-0.730	0.128	3.3E-05	-0.731	0.114	9.2E-06	Ppif	ENSRNOG00000010558	16	1,979,191
20187	A_44_P481160	-0.786	0.137	3.2E-05	-1.855	0.123	7.4E-11	Wnt5a	ENSRNOG00000015618	16	4,469,468
6846	A_64_P005043	1.370	0.123	6.0E-09	1.068	0.110	4.0E-08	Nt5dc2	ENSRNOG00000018358	16	7,212,488
11411	A_64_P158738	-1.310	0.153	6.3E-07	-1.748	0.165	4.8E-08	Dnah1	ENSRNOG00000026914	16	7,406,951
30347	A_64_P066923	0.728	0.132	4.8E-05	1.000	0.118	2.8E-07	Ncoa4	ENSRNOG00000019768	16	8,302,950
16759	A_43_P14157	0.484	0.091	7.4E-05	0.456	0.082	4.3E-05	Glud1	ENSRNOG00000057367	16	10,662,021
18286	A_44_P384017	1.193	0.112	1.1E-08	0.964	0.100	4.6E-08	Sncg	ENSRNOG00000058006	16	10,726,707
5974	A_42_P596050	-1.734	0.281	2.5E-05	-2.034	0.260	1.9E-06	Pcdh21	ENSRNOG00000013330	16	14,348,046
1186	A_42_P467381	0.497	0.073	4.5E-06	0.796	0.065	1.8E-09	Fam32a	ENSRNOG00000039528	16	19,308,842
9955	A_64_P063014	-0.538	0.093	2.9E-05	-0.437	0.083	8.2E-05	Ocel1	ENSRNOG00000032575	16	19,767,264
14171	A_64_P077627	-0.518	0.064	5.2E-07	-0.466	0.058	4.8E-07	MGC94542	ENSRNOG00000017071	16	19,792,838
22813	A_64_P028088	-0.896	0.092	4.2E-08	-0.838	0.082	2.3E-08	Lsm4	ENSRNOG00000019572	16	20,511,818
15836	A_44_P295789	1.832	0.296	1.3E-05	2.057	0.265	8.4E-07	Comp	ENSRNOG00000048472	16	20,807,070
23904	A_64_P100904	-1.012	0.147	3.7E-06	-1.478	0.131	5.4E-09	LOC688966	ENSRNOG00000020427	16	21,029,134
25971	A_64_P004531	-1.129	0.069	2.1E-11	-1.338	0.061	2.8E-13	LOC688966	ENSRNOG00000020427	16	21,029,134
231	A_64_P154034	-1.260	0.125	2.6E-08	-1.129	0.112	2.5E-08	Hapln4	ENSRNOG00000049949	16	21,089,508
16603	A_44_P508691	-1.713	0.248	3.6E-06	-2.224	0.222	2.7E-08	Tm6sf2	ENSRNOG00000042237	16	21,099,878
21524	A_64_P148892	-1.410	0.157	1.3E-07	-1.586	0.141	5.2E-09	Tm6sf2	ENSRNOG00000042237	16	21,099,878
30226	A_64_P158345	-0.687	0.122	3.9E-05	-0.991	0.109	1.1E-07	Ddx60	ENSRNOG00000056947	16	31,301,880
15146	A_44_P1036068	-0.719	0.086	3.1E-07	-0.636	0.077	3.5E-07	RGD1311747	ENSRNOG00000011293	16	32,540,217
26075	A_64_P041016	-0.738	0.124	2.2E-05	-1.432	0.111	7.8E-10	Wwc2	ENSRNOG00000013248	16	47,368,768
9194	A_44_P297252	-1.033	0.145	2.4E-06	-0.989	0.129	1.0E-06	Cyp4v3	ENSRNOG00000042426	16	50,111,306
9554	A_64_P048315	-1.056	0.139	1.1E-06	-0.696	0.124	4.1E-05		ENSRNOG00000055088	16	59,517,000
1443	A_42_P798776	-0.582	0.084	3.7E-06	-0.779	0.076	1.9E-08	LOC100364957	ENSRNOG00000059911	16	70,905,993
7832	A_44_P289173	-0.897	0.127	2.7E-06	-1.377	0.114	1.8E-09	Grtp1	ENSRNOG00000019504	16	81,616,642
3375	A_44_P309325	1.169	0.135	2.1E-07	1.018	0.121	2.9E-07	Lig4	ENSRNOG00000014605	16	85,331,866
5624	A_64_P096459	-1.239	0.202	1.4E-05	-1.330	0.180	1.6E-06	Fbp2	ENSRNOG00000017637	17	389,967
18894	A_64_P022751	0.440	0.077	3.1E-05	0.418	0.069	1.6E-05	Isca1	ENSRNOG00000018343	17	5,281,727
23510	A_44_P1039249	-0.990	0.133	1.4E-06	0.847	0.119	2.4E-06	Dok3	ENSRNOG00000013564	17	9,639,330
20732	A_42_P712949	-0.751	0.137	5.0E-05	-1.430	0.122	3.2E-09		ENSRNOG00000018122	17	10,364,503
3376	A_64_P025459	0.662	0.104	9.5E-06	0.582	0.093	1.2E-05	Bicd2	ENSRNOG00000016031	17	15,718,035
18820	A_64_P109392	0.660	0.117	3.9E-05	0.580	0.105	4.7E-05	Bicd2	ENSRNOG00000016031	17	15,718,035

Disease Models & Mechanisms • Supplementary information

22979	A_64_P133632	-0.455	0.073	1.2E-05	-0.497	0.065	1.0E-06	RGD1306058	ENSRNOG00000016560	17	15,845,931
9723	A_64_P062368	-0.483	0.091	7.2E-05	-0.616	0.081	1.2E-06	Tpmt	ENSRNOG00000016468	17	18,031,228
9873	A_44_P768945	-0.512	0.092	4.2E-05	-0.496	0.082	1.7E-05	Dtnbp1	ENSRNOG00000048719	17	20,090,246
8900	A_64_P059262	1.018	0.126	5.3E-07	1.510	0.113	4.6E-10	Riok1	ENSRNOG00000014049	17	27,451,832
21031	A_64_P067678	-1.054	0.176	1.9E-05	-1.429	0.158	1.1E-07	Aldh5a1	ENSRNOG00000023538	17	42,133,076
23925	A_64_P030919	-0.953	0.146	7.3E-06	-1.252	0.131	5.3E-08		ENSRNOG00000023538	17	42,133,076
3516	A_64_P091282	-1.807	0.203	2.3E-07	-1.261	0.203	1.7E-05	Hist1h2bb	ENSRNOG00000059949	17	43,627,930
18292	A_64_P057562	-0.680	0.111	1.5E-05	-0.525	0.099	7.5E-05		ENSRNOG00000046636	17	43,673,294
9685	A_64_P020269	-1.395	0.224	1.2E-05	-1.138	0.200	3.4E-05	Hist1h2bh	ENSRNOG00000021198	17	43,689,311
10957	A_64_P162197	-1.831	0.204	1.2E-07	-1.562	0.182	2.3E-07	Hist1h2bh	ENSRNOG00000021198	17	43,689,311
16404	A_64_P068622	-0.649	0.110	2.2E-05	-0.885	0.098	1.2E-07	Hist1h2ail	ENSRNOG00000061185	17	43,776,460
4753	A_64_P150321	-0.909	0.163	4.3E-05	-1.438	0.146	3.5E-08		ENSRNOG00000049198	17	43,791,657
14569	A_64_P057801	-1.769	0.172	1.9E-08	-1.350	0.154	1.7E-07	Hist1h2bl	ENSRNOG00000047776	17	43,808,673
12452	A_64_P063035	-1.392	0.148	1.2E-07	-1.077	0.148	2.8E-06	LOC680097	ENSRNOG00000050933	17	43,821,536
24323	A_64_P063038	-0.833	0.125	5.7E-06	-0.637	0.112	3.4E-05		ENSRNOG00000050933	17	43,821,536
13074	A_64_P130572	-1.779	0.135	5.4E-10	-1.315	0.121	8.4E-09	Hist1h2bc	ENSRNOG00000059382	17	44,738,330
20825	A_64_P004639	-1.794	0.133	3.9E-10	-1.300	0.119	8.3E-09	Hist1h2bc	ENSRNOG00000059382	17	44,738,330
5615	A_44_P506017	-1.724	0.139	1.3E-09	-1.226	0.124	3.5E-08		ENSRNOG00000054296	17	44,793,927
13053	A_64_P112008	-0.600	0.108	4.3E-05	-0.577	0.096	2.0E-05	Hist1h4b	ENSRNOG00000057690	17	44,815,995
8233	A_64_P014508	-1.598	0.157	2.3E-08	-1.358	0.141	4.6E-08	Hist1h2bm	ENSRNOG00000054966	17	44,841,000
11231	A_44_P665075	1.290	0.196	6.5E-06	1.432	0.176	4.4E-07	Dhtkd1	ENSRNOG00000023587	17	76,306,585
22322	A_64_P066267	-1.347	0.186	2.0E-06	-1.724	0.166	1.7E-08	Ccdc3	ENSRNOG00000017933	17	77,093,308
1172	A_42_P509365	0.884	0.145	1.6E-05	1.438	0.130	6.9E-09	Vim	ENSRNOG00000018087	17	80,882,666
515	A_42_P499282	0.692	0.093	1.4E-06	0.642	0.083	9.0E-07	Msrb2	ENSRNOG00000016873	17	86,066,833
13071	A_43_P17392	0.571	0.095	1.8E-05	0.819	0.085	4.6E-08	Arhgap21	ENSRNOG00000008659	17	87,826,421
16586	A_64_P071639	-0.864	0.121	2.4E-06	-1.094	0.108	2.5E-08	Thnsl1	ENSRNOG00000033076	17	88,095,830
20226	A_64_P104454	-0.967	0.165	2.5E-05	-1.015	0.148	3.8E-06	Impad1	ENSRNOG00000046647	17	90,218,013
4237	A_64_P071695	-0.522	0.101	9.4E-05	-0.753	0.090	3.3E-07	Mtcp1	ENSRNOG00000056435	18	410,098
24027	A_64_P056485	-0.558	0.081	3.8E-06	-0.672	0.073	8.1E-08	Mtcp1	ENSRNOG00000056435	18	410,098
7739	A_43_P16725	-0.673	0.093	2.0E-06	-0.573	0.083	3.7E-06	RGD1311805	ENSRNOG00000012597	18	3,597,240
17665	A_64_P113692	-2.118	0.107	4.8E-11	-4.714	0.131	2.4E-14	Myo7b	ENSRNOG00000015035	18	24,823,858
12527	A_44_P165870	0.685	0.133	9.8E-05	0.733	0.119	1.4E-05	Cdo1	ENSRNOG00000000158	18	40,716,686
2039	A_64_P135224	3.385	0.164	6.6E-13	3.763	0.147	2.3E-14	RGD1309362	ENSRNOG00000038960	18	55,505,993

6991	A_64_P050921	-0.978	0.112	1.9E-07	-1.167	0.100	3.4E-09	Ablim3	ENSRNOG00000019365	18	57,201,740
16799	A_64_P022357	2.303	0.184	2.6E-09	2.953	0.175	4.0E-11	Mc2r	ENSRNOG00000016681	18	64,177,729
17166	A_44_P419615	-1.480	0.147	2.7E-08	-1.193	0.132	1.1E-07	Stard6	ENSRNOG00000026324	18	68,983,545
24668	A_64_P128569	0.369	0.069	6.2E-05	0.355	0.061	2.8E-05	Rpl38	ENSRNOG00000033808	18	69,818,720
8500	A_64_P027255	-1.366	0.134	1.5E-07	-1.764	0.134	7.2E-09	Katnal2	ENSRNOG00000017788	18	73,360,373
4027	A_64_P158234	-0.585	0.080	1.8E-06	-0.387	0.072	6.1E-05		ENSRNOG00000023805	19	8,943,956
301	A_42_P491486	-1.920	0.242	6.5E-07	-1.489	0.217	3.8E-06	Pllp	ENSRNOG00000016558	19	10,731,855
1045	A_42_P540711	1.899	0.264	2.2E-06	1.416	0.236	1.9E-05	Rasd2	ENSRNOG00000014761	19	14,653,198
2495	A_44_P555689	1.615	0.273	2.8E-05	2.611	0.259	4.7E-08	LOC291863	ENSRNOG00000015519	19	15,033,108
17810	A_64_P148413	1.586	0.184	2.2E-07	1.962	0.165	2.4E-09	Ces1d	ENSRNOG00000015519	19	15,033,108
6652	A_64_P141469	1.255	0.150	1.5E-06	1.678	0.184	5.5E-07	Ces1a	ENSRNOG00000039725	19	15,339,152
17218	A_64_P068930	1.888	0.139	2.0E-09	3.134	0.150	6.7E-12	Ces1a	ENSRNOG00000039725	19	15,339,152
12326	A_64_P016398	-0.748	0.132	3.6E-05	-1.102	0.118	7.3E-08		ENSRNOG00000030864	19	17,967,827
27160	A_44_P482605	-0.554	0.071	7.2E-07	-0.393	0.063	1.2E-05	Gipc1	ENSRNOG00000003864	19	24,786,628
22335	A_44_P464196	-0.726	0.089	4.8E-07	-0.639	0.080	5.8E-07	Cd97	ENSRNOG00000004489	19	24,875,137
24628	A_44_P539047	-0.628	0.096	6.6E-06	-0.885	0.086	1.8E-08	Tmem208	ENSRNOG00000015974	19	37,282,018
4796	A_64_P094515	0.641	0.103	1.3E-05	-0.999	0.092	9.4E-09		ENSRNOG00000053623	19	38,643,108
7237	A_64_P052690	-0.527	0.078	4.4E-06	-0.456	0.069	6.5E-06	Pdf	ENSRNOG00000022303	19	39,246,545
21761	A_64_P127678	-0.581	0.107	5.5E-05	-1.250	0.095	6.0E-10	Dhodh	ENSRNOG00000015063	19	42,066,351
26931	A_64_P335246	0.523	0.090	2.6E-05	0.412	0.080	9.8E-05	Fuk	ENSRNOG00000059453	19	43,338,171
18704	A_43_P18171	0.572	0.106	6.1E-05	1.171	0.095	1.5E-09	Ldhd	ENSRNOG00000019036	19	43,848,937
364	A_64_P111604	-0.636	0.099	8.7E-06	-0.756	0.089	2.5E-07	Gabarapl2	ENSRNOG00000019425	19	44,164,935
12878	A_64_P138281	-1.310	0.134	3.9E-08	-1.042	0.120	1.9E-07	Cenpn	ENSRNOG00000011296	19	49,340,184
21514	A_43_P10505	1.691	0.249	4.3E-06	1.498	0.222	4.9E-06	Jph3	ENSRNOG00000018784	19	54,553,419
12200	A_64_P035694	1.262	0.155	4.5E-07	1.099	0.138	6.3E-07	LOC678766	ENSRNOG00000057601	19	56,443,632
13304	A_64_P067878	-1.194	0.227	8.0E-05	-1.452	0.203	2.4E-06	Trim67	ENSRNOG00000019024	19	57,556,694
21048	A_64_P084234	-0.741	0.135	5.2E-05	-0.950	0.121	7.4E-07	Egln1	ENSRNOG00000019773	19	57,699,113
4436	A_42_P627572	-1.452	0.134	9.3E-09	-1.104	0.120	8.8E-08	Mdc1	ENSRNOG00000032813	20	3,405,285
13335	A_43_P14382	0.727	0.113	8.2E-06	0.647	0.101	8.7E-06		ENSRNOG00000054031	20	4,873,982
10076	A_64_P056247	-1.369	0.231	2.1E-05	-1.970	0.206	5.4E-08	Msh5	ENSRNOG00000000857	20	5,037,088
21960	A_64_P139202	0.609	0.099	1.4E-05	0.903	0.089	2.1E-08	RT1-A1	ENSRNOG00000038999	20	5,414,448
20712	A_64_P094812	-0.516	0.097	7.1E-05	-0.659	0.087	1.1E-06	RGD1306917	ENSRNOG00000025909	20	5,723,737
10233	A_44_P258008	-1.828	0.163	5.8E-09	-1.028	0.146	2.8E-06	Fgd2	ENSRNOG00000000528	20	6,973,398

Disease Models & Mechanisms • Supplementary information

12621	A_44_P532249	1.435	0.254	3.7E-05	2.158	0.227	5.9E-08	Fkbp5	ENSRNOG00000022523	20	8,019,020
3691	A_42_P781999	-1.571	0.224	3.0E-06	-1.234	0.201	1.4E-05	Agpat3	ENSRNOG00000001205	20	11,114,164
8306	A_64_P008361	0.903	0.120	1.2E-06	1.209	0.107	5.1E-09	Pwp2	ENSRNOG00000001210	20	11,228,844
25935	A_64_P146698	1.112	0.127	1.8E-07	1.249	0.114	7.8E-09	Pwp2	ENSRNOG00000001210	20	11,228,844
9503	A_44_P447650	0.815	0.154	7.3E-05	0.789	0.137	3.1E-05	Adarb1	ENSRNOG00000001227	20	11,972,381
26210	A_42_P701582	1.238	0.095	6.4E-10	1.935	0.085	1.4E-13	Gstt1	ENSRNOG00000049771	20	13,799,102
14331	A_64_P152447	2.057	0.159	2.2E-08	2.960	0.180	1.5E-09	Gstt3	ENSRNOG00000001242	20	13,817,795
19033	A_44_P1015203	2.262	0.134	1.4E-11	2.584	0.120	3.4E-13	Gstt3	ENSRNOG00000001242	20	13,817,795
4293	A_64_P159078	1.959	0.269	1.9E-06	1.802	0.241	1.3E-06	RGD1306739	ENSRNOG00000000634	20	21,316,826
16577	A_64_P055843	-0.618	0.081	1.1E-06	-0.475	0.073	7.2E-06	Ascc1	ENSRNOG00000056647	20	29,558,689
8781	A_44_P152177	3.507	0.171	2.5E-12	4.400	0.163	4.3E-14	Spock2	ENSRNOG00000061544	20	29,655,226
20647	A_64_P039414	-0.687	0.105	7.1E-06	-0.858	0.094	1.0E-07	Pcbd1	ENSRNOG00000000566	20	30,690,810
14899	A_44_P1042436	-0.539	0.094	3.3E-05	-0.656	0.084	8.3E-07	Lace1	ENSRNOG00000043033	20	47,044,786
6389	A_64_P117488	-0.705	0.109	7.9E-06	-0.513	0.097	7.8E-05	Sobpl	ENSRNOG00000000316	20	47,910,375
20987	A_64_P141038	1.441	0.134	2.0E-08	0.947	0.134	3.9E-06	Bex4	ENSRNOG00000060103	L568122	30,542
141	A_64_P114320	-0.916	0.117	7.3E-07	-0.547	0.104	8.3E-05		ENSRNOG00000031780	MT	1
518	A_64_P151924	-0.968	0.110	1.6E-07	-0.517	0.098	7.8E-05		ENSRNOG00000031780	MT	1
1164	A_44_P173370	-0.911	0.096	5.5E-08	-0.445	0.085	8.8E-05		ENSRNOG00000031780	MT	1
1339	A_64_P042093	1.029	0.180	3.2E-05	1.425	0.161	1.5E-07		ENSRNOG00000031780	MT	1
1784	A_64_P108963	-0.720	0.087	3.5E-07	-0.433	0.078	4.2E-05		ENSRNOG00000031780	MT	1
1962	A_64_P011664	-1.163	0.111	1.4E-08	-0.681	0.099	3.8E-06		ENSRNOG00000031780	MT	1
3749	A_64_P046398	-1.288	0.124	1.6E-08	-0.716	0.111	7.9E-06		ENSRNOG00000031780	MT	1
3901	A_64_P067650	-0.713	0.105	4.6E-06	-0.708	0.094	1.3E-06		ENSRNOG00000031780	MT	1
3942	A_64_P023336	-0.638	0.107	2.0E-05	-0.997	0.095	1.6E-08		ENSRNOG00000031780	MT	1
4044	A_64_P072545	-1.242	0.184	4.7E-06	-0.930	0.164	3.6E-05		ENSRNOG00000031780	MT	1
4493	A_64_P147403	1.797	0.188	5.4E-08	2.134	0.168	9.7E-10		ENSRNOG00000031780	MT	1
4577	A_64_P016393	-0.692	0.094	1.6E-06	-1.154	0.084	2.9E-10		ENSRNOG00000031780	MT	1
4711	A_44_P414984	-0.931	0.162	3.0E-05	-1.247	0.145	2.2E-07		ENSRNOG00000031780	MT	1
5065	A_64_P146350	-0.861	0.161	6.5E-05	-1.453	0.144	2.4E-08		ENSRNOG00000031780	MT	1
5091	A_44_P342334	-0.551	0.070	7.4E-07	-0.391	0.063	1.3E-05		ENSRNOG00000031780	MT	1
5100	A_64_P128335	-0.668	0.106	1.0E-05	-0.495	0.094	8.2E-05		ENSRNOG00000031780	MT	1
5223	A_64_P049606	-1.160	0.155	1.3E-06	-0.719	0.138	8.9E-05		ENSRNOG00000031780	MT	1
5273	A_64_P005669	-1.081	0.099	8.7E-09	-0.662	0.089	1.4E-06		ENSRNOG00000031780	MT	1

Disease Models & Mechanisms • Supplementary information

5295	A_64_P159095	-0.971	0.107	1.1E-07	-0.528	0.096	4.9E-05	ENSRNOG00000031780	MT	1
5482	A_64_P326359	0.840	0.088	5.5E-08	1.272	0.079	2.7E-11	ENSRNOG00000031780	MT	1
5765	A_64_P069541	-1.266	0.112	4.9E-09	-0.695	0.100	3.3E-06	ENSRNOG00000031780	MT	1
5941	A_64_P136655	-0.573	0.106	6.0E-05	-0.679	0.095	2.4E-06	ENSRNOG00000031780	MT	1
5979	A_64_P127664	-0.625	0.091	3.7E-06	-0.690	0.081	2.6E-07	ENSRNOG00000031780	MT	1
6008	A_64_P013827	-1.043	0.104	2.8E-08	-0.593	0.093	9.5E-06	ENSRNOG00000031780	MT	1
6077	A_44_P854356	1.401	0.170	6.1E-07	1.049	0.162	1.0E-05	ENSRNOG00000031780	MT	1
6153	A_64_P068832	-0.877	0.103	2.6E-07	-0.522	0.092	3.6E-05	ENSRNOG00000031780	MT	1
6998	A_64_P115122	0.652	0.094	3.3E-06	0.447	0.084	6.8E-05	ENSRNOG00000031780	MT	1
7558	A_64_P119802	-1.208	0.156	8.6E-07	-0.749	0.140	6.3E-05	ENSRNOG00000031780	MT	1
7605	A_64_P048712	-1.558	0.204	3.8E-06	-1.759	0.166	9.9E-08	ENSRNOG00000031780	MT	1
7864	A_64_P060396	-1.342	0.114	2.8E-09	-0.819	0.102	5.3E-07	ENSRNOG00000031780	MT	1
8064	A_64_P004327	-1.159	0.165	3.0E-06	-0.814	0.148	4.8E-05	ENSRNOG00000031780	MT	1
8089	A_64_P030000	-1.010	0.104	4.3E-08	-0.614	0.093	6.2E-06	ENSRNOG00000031780	MT	1
8258	A_64_P028824	-0.997	0.156	9.2E-06	-0.809	0.140	2.8E-05	ENSRNOG00000031780	MT	1
8379	A_64_P005704	-1.029	0.108	5.6E-08	-0.648	0.097	5.2E-06	ENSRNOG00000031780	MT	1
8814	A_64_P134032	-1.611	0.174	8.1E-08	-0.909	0.155	2.5E-05	ENSRNOG00000031780	MT	1
9679	A_64_P061971	-1.082	0.106	2.2E-08	-0.614	0.095	7.9E-06	ENSRNOG00000031780	MT	1
9737	A_64_P152543	-0.928	0.110	2.8E-07	-0.506	0.098	9.7E-05	ENSRNOG00000031780	MT	1
9965	A_64_P022180	-1.046	0.106	3.2E-08	-0.701	0.094	1.5E-06	ENSRNOG00000031780	MT	1
10100	A_64_P138684	-0.842	0.098	2.4E-07	-0.628	0.088	2.4E-06	ENSRNOG00000031780	MT	1
10707	A_44_P295548	-0.968	0.096	2.6E-08	-0.594	0.086	3.6E-06	ENSRNOG00000031780	MT	1
10749	A_44_P410318	-0.773	0.113	4.0E-06	0.616	0.101	1.6E-05	ENSRNOG00000031780	MT	1
10987	A_64_P147669	-1.182	0.150	7.1E-07	-0.694	0.134	9.7E-05	ENSRNOG00000031780	MT	1
11073	A_64_P053888	-1.372	0.141	4.1E-08	-0.759	0.126	1.8E-05	ENSRNOG00000031780	MT	1
11217	A_64_P161448	-1.476	0.120	1.6E-09	-0.850	0.108	6.8E-07	ENSRNOG00000031780	MT	1
11571	A_64_P070896	-1.044	0.092	4.7E-09	-0.570	0.082	3.4E-06	ENSRNOG00000031780	MT	1
11646	A_64_P049816	-0.952	0.091	1.5E-08	-0.458	0.081	3.7E-05	ENSRNOG00000031780	MT	1
11648	A_64_P003045	-1.011	0.097	1.5E-08	-0.561	0.086	7.6E-06	ENSRNOG00000031780	MT	1
12489	A_64_P121623	-0.720	0.104	3.6E-06	-0.564	0.093	1.7E-05	ENSRNOG00000031780	MT	1
13083	A_64_P077603	-0.700	0.128	5.2E-05	-1.061	0.115	8.2E-08	ENSRNOG00000031780	MT	1
13321	A_44_P335415	-1.235	0.106	3.3E-09	-0.731	0.095	9.1E-07	ENSRNOG00000031780	MT	1
13469	A_64_P232363	-0.877	0.108	4.8E-07	-0.497	0.097	1.0E-04	ENSRNOG00000031780	MT	1

13844	A_64_P054907	-0.386	0.067	2.8E-05	-0.570	0.060	5.3E-08	ENSRNOG00000031780	MT	1
13857	A_64_P155584	-1.049	0.114	9.2E-08	-0.540	0.102	7.5E-05	ENSRNOG00000031780	MT	1
14000	A_64_P069824	-1.155	0.102	4.6E-09	-0.693	0.091	1.0E-06	ENSRNOG00000031780	MT	1
14216	A_64_P079438	-1.298	0.147	1.5E-07	-0.789	0.131	1.8E-05	ENSRNOG00000031780	MT	1
14499	A_64_P074731	-0.620	0.103	1.8E-05	-0.653	0.092	2.5E-06	ENSRNOG00000031780	MT	1
15006	A_64_P083725	-0.635	0.090	2.8E-06	-0.485	0.081	1.8E-05	ENSRNOG00000031780	MT	1
15317	A_64_P086459	-0.819	0.125	6.9E-06	-0.631	0.112	3.8E-05	ENSRNOG00000031780	MT	1
15462	A_64_P057452	-0.852	0.100	2.5E-07	-0.492	0.089	4.9E-05	ENSRNOG00000031780	MT	1
15472	A_64_P103681	-0.920	0.092	3.0E-08	-0.474	0.083	3.1E-05	ENSRNOG00000031780	MT	1
15612	A_64_P010052	-1.732	0.218	2.4E-06	-1.523	0.267	7.3E-05	ENSRNOG00000031780	MT	1
15639	A_44_P468694	-0.979	0.114	2.2E-07	-0.561	0.102	4.7E-05	ENSRNOG00000031780	MT	1
15646	A_64_P258712	-1.070	0.108	3.1E-08	-0.504	0.096	8.3E-05	ENSRNOG00000031780	MT	1
15692	A_44_P147356	-0.987	0.107	8.1E-08	-0.554	0.095	2.7E-05	ENSRNOG00000031780	MT	1
16690	A_64_P005724	-1.057	0.104	2.4E-08	-0.625	0.093	5.2E-06	ENSRNOG00000031780	MT	1
16734	A_64_P046785	-0.777	0.098	6.8E-07	-0.574	0.088	7.2E-06	ENSRNOG00000031780	MT	1
16966	A_44_P138098	-0.640	0.081	7.2E-07	-0.769	0.073	1.3E-08	ENSRNOG00000031780	MT	1
17820	A_64_P106351	-0.940	0.098	4.9E-08	-0.632	0.087	2.1E-06	ENSRNOG00000031780	MT	1
18412	A_64_P059846	-0.618	0.096	8.6E-06	-0.520	0.086	1.8E-05	ENSRNOG00000031780	MT	1
18542	A_64_P134283	-1.021	0.133	9.5E-07	-0.679	0.119	3.2E-05	ENSRNOG00000031780	MT	1
18662	A_64_P105019	-0.915	0.084	8.2E-09	-0.537	0.075	2.3E-06	ENSRNOG00000031780	MT	1
19007	A_64_P115916	-0.668	0.093	2.4E-06	-0.735	0.084	1.7E-07	ENSRNOG00000031780	MT	1
19023	A_64_P066639	-1.189	0.124	5.3E-08	-0.742	0.111	5.5E-06	ENSRNOG00000031780	MT	1
19449	A_64_P098784	-0.805	0.133	2.3E-05	-0.870	0.133	9.9E-06	ENSRNOG00000031780	MT	1
19614	A_64_P005744	-0.976	0.099	3.4E-08	-0.566	0.088	8.9E-06	ENSRNOG00000031780	MT	1
19768	A_64_P064259	-1.041	0.097	9.9E-09	-0.646	0.086	1.4E-06	ENSRNOG00000031780	MT	1
20224	A_64_P066848	-1.022	0.097	1.4E-08	-0.640	0.087	1.6E-06	ENSRNOG00000031780	MT	1
20460	A_64_P029376	-1.219	0.161	1.1E-06	-0.767	0.144	6.8E-05	ENSRNOG00000031780	MT	1
20800	A_44_P545895	-1.063	0.113	6.8E-08	1.043	0.101	1.9E-08	ENSRNOG00000031780	MT	1
21135	A_64_P118308	-1.654	0.131	1.0E-09	-0.631	0.117	6.1E-05	ENSRNOG00000031780	MT	1
21251	A_64_P154655	-1.374	0.106	7.4E-10	-0.732	0.095	9.4E-07	ENSRNOG00000031780	MT	1
21285	A_64_P083179	-1.539	0.183	2.9E-07	-0.899	0.163	4.9E-05	ENSRNOG00000031780	MT	1
21806	A_64_P094555	-0.983	0.135	1.8E-06	-1.179	0.120	3.8E-08	ENSRNOG00000031780	MT	1
21864	A_44_P114892	-0.956	0.104	8.6E-08	-0.549	0.093	2.2E-05	ENSRNOG00000031780	MT	1

Disease Models & Mechanisms • Supplementary information

21883	A_64_P123207	-0.898	0.089	2.5E-08	-0.454	0.079	3.3E-05	ENSRNOG00000031780	MT	1
22460	A_44_P105441	-0.943	0.098	5.1E-08	-0.496	0.088	3.8E-05	ENSRNOG00000031780	MT	1
22492	A_64_P086469	-1.184	0.114	1.7E-08	-0.681	0.102	5.3E-06	ENSRNOG00000031780	MT	1
23014	A_64_P152542	-1.027	0.096	1.1E-08	-0.508	0.086	2.1E-05	ENSRNOG00000031780	MT	1
23075	A_64_P076495	-0.844	0.104	4.9E-07	-0.502	0.093	6.1E-05	ENSRNOG00000031780	MT	1
23570	A_64_P112534	-0.792	0.138	3.2E-05	-1.270	0.124	2.0E-08	ENSRNOG00000031780	MT	1
23622	A_64_P132427	-1.014	0.096	1.3E-08	-0.640	0.086	1.4E-06	ENSRNOG00000031780	MT	1
23877	A_64_P086353	-1.232	0.112	7.5E-09	-0.689	0.100	3.8E-06	ENSRNOG00000031780	MT	1
24034	A_64_P044613	-0.722	0.096	1.2E-06	-0.506	0.086	2.2E-05	ENSRNOG00000031780	MT	1
24098	A_64_P042811	-2.145	0.201	1.2E-08	-2.160	0.180	2.1E-09	ENSRNOG00000031780	MT	1
24790	A_64_P104314	-1.090	0.123	1.5E-07	-0.615	0.110	4.1E-05	ENSRNOG00000031780	MT	1
25275	A_64_P055502	-1.452	0.207	2.9E-06	-1.001	0.185	5.8E-05	ENSRNOG00000031780	MT	1
25367	A_64_P052280	0.459	0.089	9.9E-05	0.551	0.080	3.6E-06	ENSRNOG00000031780	MT	1
25459	A_64_P146637	-1.103	0.163	4.6E-06	-2.882	0.146	1.2E-12	ENSRNOG00000031780	MT	1
25465	A_64_P154826	0.702	0.110	8.9E-06	0.843	0.098	2.2E-07	ENSRNOG00000031780	MT	1
26291	A_64_P142460	-0.691	0.112	1.3E-05	-0.993	0.100	3.1E-08	ENSRNOG00000031780	MT	1
26385	A_64_P081630	-0.682	0.089	1.0E-06	-0.734	0.080	9.0E-08	ENSRNOG00000031780	MT	1
26390	A_64_P034947	-0.817	0.096	2.4E-07	-0.453	0.085	7.3E-05	ENSRNOG00000031780	MT	1
26402	A_64_P162272	-1.046	0.130	5.1E-07	-1.867	0.116	2.8E-11	ENSRNOG00000031780	MT	1
26659	A_64_P051019	-1.086	0.099	7.8E-09	-0.711	0.089	5.5E-07	ENSRNOG00000031780	MT	1
26716	A_64_P070288	-1.071	0.107	2.8E-08	-0.685	0.096	2.3E-06	ENSRNOG00000031780	MT	1
26724	A_64_P128336	-0.939	0.092	2.1E-08	-0.557	0.082	4.5E-06	ENSRNOG00000031780	MT	1
26751	A_64_P119395	-0.972	0.102	5.8E-08	-0.482	0.092	7.7E-05	ENSRNOG00000031780	MT	1
27129	A_64_P162408	-0.717	0.095	1.2E-06	-0.891	0.085	1.5E-08	ENSRNOG00000031780	MT	1
27262	A_64_P143003	-0.960	0.185	9.2E-05	-1.292	0.166	7.9E-07	ENSRNOG00000031780	MT	1
27283	A_64_P014787	-0.895	0.113	6.7E-07	-0.528	0.101	8.6E-05	ENSRNOG00000031780	MT	1
27679	A_64_P143154	0.526	0.099	7.1E-05	0.981	0.089	6.8E-09	ENSRNOG00000031780	MT	1
27740	A_64_P024476	-0.813	0.121	5.0E-06	-0.679	0.108	1.1E-05	ENSRNOG00000031780	MT	1
28018	A_64_P101600	-5.911	0.243	3.5E-10	-6.418	0.188	1.3E-11	ENSRNOG00000031780	MT	1
28168	A_64_P001399	-0.912	0.084	9.4E-09	-0.439	0.075	2.7E-05	ENSRNOG00000031780	MT	1
28308	A_64_P028365	0.359	0.064	4.3E-05	0.408	0.058	2.7E-06	ENSRNOG00000031780	MT	1
28481	A_44_P392004	2.345	0.212	1.3E-08	2.771	0.201	6.8E-10	ENSRNOG00000031780	MT	1
29033	A_64_P150064	-0.813	0.090	1.1E-07	-0.579	0.081	2.2E-06	ENSRNOG00000031780	MT	1

Disease Models & Mechanisms • Supplementary information

29125	A_64_P084699	0.366	0.068	5.9E-05	0.344	0.061	3.5E-05		ENSRNOG00000031780	MT	1
29480	A_64_P134485	-1.022	0.170	1.9E-05	-1.269	0.152	3.3E-07		ENSRNOG00000031780	MT	1
29528	A_64_P096684	-0.871	0.090	4.5E-08	-0.436	0.080	5.8E-05		ENSRNOG00000031780	MT	1
29633	A_64_P005745	-0.915	0.094	4.0E-08	-0.469	0.084	4.1E-05		ENSRNOG00000031780	MT	1
29738	A_44_P898566	-1.095	0.167	6.9E-06	-1.393	0.150	7.7E-08		ENSRNOG00000031780	MT	1
29810	A_64_P034311	-0.852	0.094	1.1E-07	-0.445	0.084	7.4E-05		ENSRNOG00000031780	MT	1
21771	A_44_P808769	-1.209	0.142	2.5E-07	-1.303	0.127	2.0E-08	Clcn4-2	ENSRNOG00000003533	X	25,016,401
5870	A_64_P018964	-1.516	0.220	5.4E-06	-1.298	0.220	3.0E-05	Hmga1	ENSRNOG00000026969	X	33,349,481
3164	A_44_P389448	-0.896	0.113	6.1E-07	-0.893	0.101	1.5E-07		ENSRNOG00000003860	X	43,831,892
4242	A_44_P356605	0.503	0.088	3.1E-05	0.800	0.078	2.1E-08	Las1l	ENSRNOG00000021748	X	65,083,911
29031	A_64_P067148	-1.082	0.098	6.7E-09	-0.647	0.087	1.5E-06		ENSRNOG00000051290	X	70,234,622
29973	A_64_P098404	-0.493	0.094	8.3E-05	-0.581	0.084	3.7E-06		ENSRNOG00000018301	X	74,372,283
1020	A_42_P764985	1.026	0.156	6.4E-06	1.138	0.139	4.4E-07	Nap1l3	ENSRNOG00000029087	X	95,062,132
1864	A_44_P534557	1.385	0.232	2.0E-05	1.245	0.207	1.9E-05	Gprasp2	ENSRNOG00000037658	X	106,360,393
17032	A_42_P822638	1.124	0.125	1.2E-07	0.637	0.111	3.2E-05	Tsc22d3	ENSRNOG00000056135	X	111,887,906
15663	A_64_P048852	-1.668	0.118	1.9E-10	-1.512	0.105	1.5E-10		ENSRNOG00000006459	X	120,313,696
16887	A_64_P065957	-0.523	0.088	2.2E-05	-0.583	0.079	1.6E-06	Ube2a	ENSRNOG00000039985	X	123,486,989
25743	A_64_P076705	0.497	0.088	3.6E-05	0.462	0.079	2.4E-05	Gdi1	ENSRNOG00000056870	X	156,407,404
2375	A_43_P16542	-0.478	0.091	7.8E-05	-0.667	0.081	4.0E-07	no transcript			
3613	A_64_P154736	-0.566	0.087	7.4E-06	-0.662	0.078	2.5E-07	LOC690885			
6725	A_64_P063194	-0.779	0.089	1.8E-07	-1.105	0.080	2.7E-10	RGD1311186			
7034	A_44_P534028	1.150	0.176	7.1E-06	1.473	0.158	7.2E-08	Stau2			
7737	A_64_P123798	-0.523	0.078	5.0E-06	-0.389	0.070	4.0E-05	LOC681193			
7826	A_64_P024117	-2.112	0.208	1.5E-06	-2.002	0.269	2.3E-05	LOC497952			
8329	A_64_P124041	-0.847	0.120	4.0E-06	-1.056	0.120	2.7E-07	Ddx60			
9001	A_42_P483302	0.875	0.119	1.6E-06	1.044	0.106	3.7E-08	Znf23			
10788	A_43_P18376	-2.525	0.213	2.6E-09	-3.772	0.190	1.2E-12	Tex49 (RGD1305928)_partial			
11195	A_64_P084698	0.458	0.072	9.1E-06	0.394	0.064	1.4E-05	LOC680353			
15156	A_64_P133051	1.389	0.195	2.5E-06	0.950	0.174	5.5E-05	RGD1566102			
15289	A_44_P393282	-1.498	0.162	8.4E-08	-1.287	0.145	1.4E-07	44083			
15659	A_43_P18931	0.813	0.102	6.0E-07	0.852	0.091	7.3E-08	Tp53i3_RGD130	ENSRNOG00000005177	13	75,101,759
17474	A_44_P201084	-0.949	0.154	1.3E-05	-0.932	0.137	4.5E-06	Fgfr1l			
19713	A_44_P160189	-0.601	0.100	1.8E-05	-0.459	0.089	9.8E-05	Smim20_RGD15	N/A	14	60,057,493

Disease Models & Mechanisms • Supplementary information

20392	A_64_P081063	2.293	0.216	1.9E-07	2.765	0.341	3.4E-06	LOC363746			
20627	A_64_P091328	-1.011	0.128	6.9E-07	-1.124	0.115	3.8E-08	Rrm1-ps1			
20926	A_64_P106426	0.452	0.082	5.0E-05	0.400	0.074	5.5E-05	LOC688632			
21953	A_64_P005659	-1.217	0.212	3.1E-05	-1.070	0.190	3.8E-05	Gapdh-ps1			
23957	A_64_P139208	-0.551	0.105	8.4E-05	-0.500	0.094	7.2E-05	RT1-N1			
24017	A_64_P060938	-1.580	0.196	5.1E-07	-1.945	0.175	6.5E-09	LOC686120			
24789	A_44_P324482	0.719	0.133	5.7E-05	0.887	0.119	1.3E-06	Smyd3			
25709	A_64_P011719	-0.951	0.106	1.3E-07	-0.768	0.095	5.0E-07	Chmp1b	N/A	18	62,923,620
25822	A_44_P314087	-1.292	0.194	5.5E-06	-1.453	0.173	3.1E-07	LOC681186			

Genes are regarded as significant for “shared” differential expression when they show $P<1\times10^{-4}$ reproducibly in two types of comparison, ie, SHR/Izm versus WKY/Izm and SHRSP/Izm versus WKY/Izm, with a concordant direction of differential expression.

Disease Models & Mechanisms • Supplementary information

Table S3C. A list of significant transcripts for shared differential expression in the whole kidney.

no	ProbeName	SHR/lzm (vs WKY/lzm)			SHRSP/lzm (vs WKY/lzm)			GeneName	Ensembl_rat	Chr	Transcription_start_site (Rnor_6.0)
		Fold change (log2-scale)	SE (log2-scale)	P-value	Fold change (log2-scale)	SE (log2-scale)	P-value				
2382	A_64_P063358	1.040	0.166	2.3E-06	1.586	0.235	7.5E-07	Samd5	ENSRNOG00000023549	1	3,763,392
8464	A_64_P129293	1.425	0.178	4.4E-08	1.848	0.251	1.9E-07	RGD1306565	ENSRNOG00000031700	1	15,412,603
11055	A_44_P128306	0.448	0.065	4.7E-07	0.578	0.091	1.9E-06	Aldh8a1	ENSRNOG00000014907	1	16,910,069
21505	A_64_P117799	0.753	0.145	2.9E-05	-1.092	0.205	2.2E-05	Slc9a3	ENSRNOG00000015159	1	31,777,070
7941	A_42_P689905	1.273	0.130	1.3E-09	1.626	0.184	8.2E-09	Apoc2	ENSRNOG00000018402	1	80,594,136
15577	A_44_P370267	-0.377	0.069	1.4E-05	0.560	0.097	7.4E-06	Ethe1	ENSRNOG00000019982	1	81,456,984
13915	A_64_P080941	-1.273	0.095	5.7E-12	-1.456	0.130	1.6E-10	LOC102556967	ENSRNOG00000033729	1	99,505,677
22453	A_44_P163243	-1.604	0.153	3.6E-10	-2.088	0.217	1.7E-09	Klks3	ENSRNOG00000032857	1	100,059,967
19183	A_64_P133177	-0.914	0.092	9.1E-10	-1.135	0.130	9.8E-09	Sema4b	ENSRNOG00000025167	1	141,986,145
26335	A_64_P157285	0.978	0.134	2.0E-07	1.228	0.189	1.3E-06	RGD1560277 (in	ENSRNOG00000025003	1	142,027,250
25376	A_64_P109232	-2.130	0.082	2.3E-17	-2.703	0.120	4.5E-16	Nmb	ENSRNOG00000011011	1	142,724,511
14093	A_42_P788740	0.537	0.105	3.7E-05	0.986	0.149	9.7E-07	Folr2	ENSRNOG00000019890	1	166,919,302
20060	A_44_P384389	0.540	0.081	9.1E-07	-1.289	0.115	9.0E-11		ENSRNOG00000033924	1	178,367,828
21647	A_64_P150981	0.510	0.102	4.5E-05	-0.918	0.144	1.7E-06		ENSRNOG00000025122	1	183,630,695
29269	A_64_P151353	1.764	0.086	3.5E-16	2.146	0.122	8.7E-15	Acsm5	ENSRNOG00000031211	1	189,241,593
5216	A_43_P15937	0.425	0.077	1.3E-05	0.700	0.109	1.5E-06	Acsm3	ENSRNOG00000032246	1	189,514,553
15247	A_44_P1005462	1.210	0.144	1.9E-08	1.329	0.203	1.2E-06	Mylpf	ENSRNOG00000017645	1	198,655,742
4048	A_44_P452222	2.154	0.133	2.3E-10	1.491	0.211	6.0E-06	Itgal	ENSRNOG00000017980	1	198,744,050
14224	A_64_P045578	1.120	0.153	1.9E-07	1.128	0.216	2.8E-05	Rnf40	ENSRNOG00000018840	1	199,037,544
4254	A_42_P784614	0.545	0.083	1.0E-06	0.780	0.117	8.7E-07	Htra1	ENSRNOG00000020533	1	201,499,028
10007	A_44_P196717	3.476	0.212	1.9E-10	-2.705	0.501	9.9E-05	Stk32c	ENSRNOG00000006962	1	211,520,699
13890	A_64_P031501	-0.915	0.102	5.7E-09	-0.685	0.144	8.5E-05	Slc22a9	ENSRNOG00000056396	1	222,975,509
11870	A_64_P073303	-0.691	0.132	2.8E-05	-1.962	0.187	3.5E-10	Plac1l (OOSP2)	ENSRNOG00000036624	1	228,014,924
13	A_42_P751152	-0.382	0.050	1.1E-07	-0.380	0.071	2.1E-05	Ndufb8	ENSRNOG00000014078	1	264,303,762
26828	A_64_P043941	-1.461	0.150	1.4E-09	-1.512	0.212	3.2E-07	Sorcs1	ENSRNOG00000011313	1	270,472,866
3220	A_64_P066271	-0.754	0.083	5.1E-09	-0.772	0.118	1.1E-06	Mxi1	ENSRNOG00000034078	1	274,030,978
17839	A_64_P115991	-0.895	0.098	4.4E-09	-0.706	0.139	3.8E-05	Mxi1	ENSRNOG00000034078	1	274,030,978
22136	A_64_P066276	-1.000	0.101	1.1E-09	-0.724	0.143	4.3E-05	Mxi1	ENSRNOG00000034078	1	274,030,978
18506	A_64_P087184	0.704	0.113	2.3E-06	0.760	0.159	8.4E-05	Vwa2	ENSRNOG00000025581	1	277,689,729
22904	A_44_P535284	-0.634	0.098	1.3E-06	-1.027	0.138	1.5E-07	Bhmt	ENSRNOG00000011200	2	23,256,158

Disease Models & Mechanisms • Supplementary information

8784	A_44_P899264	-0.722	0.104	4.9E-07	-0.772	0.147	2.6E-05	Lhfpl2	ENSRNOG00000011032	2	23,770,721
8031	A_64_P163219	-2.065	0.119	3.3E-14	-1.535	0.163	3.8E-09		ENSRNOG00000018615	2	30,780,121
18451	A_43_P12257	1.641	0.098	2.9E-14	1.742	0.139	1.1E-11	Esm1	ENSRNOG00000010797	2	45,104,305
912	A_42_P603536	0.420	0.087	7.2E-05	0.661	0.123	1.9E-05	Arl15	ENSRNOG00000011105	2	45,668,969
24926	A_64_P154605	-0.705	0.096	1.7E-07	-0.665	0.135	5.8E-05	Fst	ENSRNOG00000011631	2	46,544,457
5239	A_42_P463754	0.628	0.063	9.2E-10	0.529	0.089	5.1E-06	Itga1	ENSRNOG00000053550	2	47,281,421
1843	A_42_P469751	-0.901	0.068	3.5E-12	-1.026	0.096	2.5E-10	C7	ENSRNOG00000061379	2	54,777,729
11752	A_64_P087930	-1.108	0.106	3.4E-10	-1.046	0.149	4.1E-07	C7	ENSRNOG00000061379	2	54,777,729
5037	A_44_P187181	-0.849	0.088	1.6E-09	-0.841	0.124	7.1E-07	Prlr	ENSRNOG00000057557	2	60,131,776
27013	A_64_P111315	-0.781	0.133	6.0E-06	-1.028	0.189	1.6E-05	Ropn1l	ENSRNOG00000042781	2	84,531,192
15982	A_42_P778330	-1.703	0.153	1.9E-10	-1.890	0.209	7.6E-09	Sox2	ENSRNOG00000012199	2	121,165,137
30009	A_64_P144879	0.502	0.076	9.2E-07	0.779	0.107	2.2E-07	Rarres1	ENSRNOG00000037853	2	164,684,985
16967	A_44_P773166	-0.796	0.122	1.2E-06	-1.013	0.172	5.6E-06	Tmem144	ENSRNOG00000010081	2	178,612,470
11879	A_64_P020616	-0.480	0.081	5.0E-06	-0.778	0.115	6.6E-07	Clk2	ENSRNOG00000020500	2	188,476,820
21728	A_64_P009738	1.012	0.125	2.2E-06	1.704	0.204	1.6E-06	RGD1559714	ENSRNOG00000061251	2	195,279,218
10680	A_64_P140977	-0.900	0.169	2.1E-05	-1.452	0.238	3.4E-06	Prune	ENSRNOG00000021120	2	196,456,481
27712	A_64_P041307	-0.793	0.061	5.7E-12	-0.886	0.087	5.7E-10	Tars2	ENSRNOG00000057194	2	197,878,142
7908	A_64_P126230	-0.527	0.091	6.5E-06	-0.914	0.128	3.0E-07	Car14	ENSRNOG00000023162	2	198,016,898
4450	A_42_P649672	1.501	0.142	2.9E-10	2.169	0.201	1.9E-10	Sv2a	ENSRNOG00000021182	2	198,321,142
4369	A_64_P069023	0.412	0.068	1.4E-05	-0.754	0.109	2.8E-06	Cd160	ENSRNOG00000000097	2	198,942,732
4856	A_64_P139078	3.583	0.163	6.6E-16	3.520	0.282	3.9E-11	Hsd3b6	ENSRNOG00000019441	2	202,350,929
24143	A_44_P506299	1.322	0.185	2.9E-07	1.833	0.261	4.0E-07	RGD1562344	ENSRNOG00000037149	2	206,314,213
8954	A_64_P052833	0.434	0.074	5.5E-06	-0.751	0.104	2.6E-07		ENSRNOG00000053044	2	208,225,888
18048	A_44_P398167	-0.646	0.069	2.9E-09	-0.513	0.098	2.6E-05	Prdx1	ENSRNOG00000017695	2	208,738,132
18309	A_64_P071615	0.474	0.076	2.2E-06	-0.796	0.107	1.5E-07		ENSRNOG00000030491	2	226,825,635
1880	A_42_P770336	1.222	0.131	2.9E-09	1.380	0.185	1.4E-07	Mcoln2	ENSRNOG00000015089	2	252,018,597
6820	A_64_P116854	0.317	0.058	1.4E-05	-0.385	0.081	9.3E-05		ENSRNOG00000036451	3	1,260,569
28745	A_43_P15529	-0.726	0.130	1.1E-05	-1.188	0.183	1.3E-06	Ptgds	ENSRNOG00000015550	3	2,689,084
26230	A_44_P213133	-2.047	0.091	4.7E-17	-2.158	0.129	2.6E-14	Endog	ENSRNOG00000016033	3	8,741,766
6137	A_64_P119821	-0.868	0.132	1.1E-06	-1.059	0.187	9.2E-06	RGD1311084	ENSRNOG00000018486	3	9,636,058
22393	A_43_P18366	1.402	0.094	3.0E-13	1.682	0.133	8.7E-12	Tor1b	ENSRNOG00000006435	3	9,792,899
24771	A_43_P10292	-0.579	0.065	7.4E-09	-0.460	0.092	4.9E-05	siat7D	ENSRNOG00000048870	3	11,607,225
19913	A_44_P102477	-1.659	0.208	4.9E-08	-2.107	0.295	2.9E-07	Morn5	ENSRNOG00000026111	3	15,379,109

Disease Models & Mechanisms • Supplementary information

9295	A_64_P057740	-1.207	0.184	1.1E-06	-1.581	0.260	3.5E-06	Ccdc148	ENSRNOG00000057740	3	45,210,474
12780	A_64_P025310	-1.072	0.115	3.3E-09	-1.441	0.163	8.2E-09	Galnt3	ENSRNOG00000005727	3	52,212,412
8214	A_64_P077658	-0.990	0.120	2.6E-08	-1.737	0.169	5.0E-10		ENSRNOG00000005868	3	52,361,060
30302	A_64_P308666	-1.227	0.130	2.3E-09	-1.853	0.183	6.8E-10	Ttc21b	ENSRNOG00000005868	3	52,361,060
25593	A_44_P166206	0.876	0.105	2.2E-08	0.830	0.148	1.1E-05	Nostrin	ENSRNOG00000006611	3	55,369,214
16738	A_44_P344777	0.425	0.063	8.0E-07	-0.704	0.090	6.2E-08		ENSRNOG00000047948	3	57,770,948
24139	A_44_P992697	-1.777	0.169	3.2E-10	-1.411	0.239	5.2E-06	Dusp19	ENSRNOG00000008868	3	67,849,966
17375	A_43_P15275	-1.700	0.071	1.1E-17	-1.534	0.100	1.7E-13	Ptprj	ENSRNOG00000034025	3	79,390,956
18771	A_44_P107766	-1.048	0.100	3.5E-10	-0.984	0.141	4.6E-07	RGD1309540	ENSRNOG00000014798	3	80,349,145
7851	A_64_P062618	0.609	0.109	1.1E-05	1.024	0.154	9.4E-07	Chst1	ENSRNOG00000007989	3	81,498,022
25223	A_64_P115142	-0.809	0.087	3.3E-09	-0.833	0.123	7.1E-07	Accs	ENSRNOG00000009199	3	82,756,953
30117	A_64_P058202	0.589	0.090	1.2E-06	-1.173	0.127	3.9E-09		ENSRNOG00000045620	3	85,544,827
3427	A_44_P1024721	1.005	0.116	1.1E-08	1.337	0.164	3.2E-08	Slc28a2	ENSRNOG00000028668	3	114,355,798
12403	A_44_P398033	0.443	0.085	2.8E-05	0.818	0.120	6.3E-07	Prnp	ENSRNOG00000021259	3	124,515,978
12542	A_64_P042611	-2.639	0.171	1.5E-13	-3.020	0.242	1.1E-11		ENSRNOG00000036900	3	147,992,754
26332	A_64_P088452	-1.796	0.197	4.4E-09	-2.854	0.278	5.1E-10	Defb29	ENSRNOG00000023195	3	148,010,176
5599	A_43_P18817	-1.356	0.150	5.4E-09	-1.237	0.212	6.3E-06	Ift52	ENSRNOG00000007692	3	159,392,193
27964	A_44_P576289	-1.028	0.170	3.7E-06	-1.323	0.240	1.4E-05	Stk4	ENSRNOG00000013529	3	160,467,552
11686	A_64_P103956	-0.380	0.059	1.6E-06	-0.438	0.084	2.7E-05	Acot8	ENSRNOG00000015187	3	161,272,460
16812	A_64_P058749	0.569	0.074	8.2E-08	0.534	0.104	3.5E-05		ENSRNOG00000043150	3	176,479,335
21357	A_64_P016751	-1.150	0.101	7.5E-11	-1.207	0.143	1.9E-08	Tmem243 (RGD1	ENSRNOG00000042758	4	21,920,651
12659	A_64_P010925	0.846	0.082	4.5E-10	1.004	0.116	1.1E-08	Abcb1b	ENSRNOG00000008012	4	22,307,453
4219	A_64_P103537	0.944	0.143	1.1E-06	1.206	0.203	4.8E-06	Cftr	ENSRNOG00000055103	4	42,692,836
17535	A_64_P072314	0.747	0.056	2.6E-12	0.733	0.079	3.1E-09		ENSRNOG00000052702	4	43,915,333
15750	A_64_P077103	-0.397	0.083	9.0E-05	-0.899	0.113	6.8E-08	Kcp	ENSRNOG00000021855	4	56,786,754
12618	A_64_P009490	0.998	0.171	6.3E-06	1.285	0.242	2.2E-05	LOC679651	ENSRNOG00000047635	4	68,011,932
7316	A_43_P16967	-1.420	0.099	2.9E-12	-1.099	0.146	2.2E-07	Snx10	ENSRNOG00000011944	4	81,311,490
7404	A_43_P23115	1.592	0.144	1.2E-10	2.436	0.203	2.6E-11	Inmt	ENSRNOG00000011250	4	85,386,231
4571	A_64_P044087	0.505	0.097	3.3E-05	0.846	0.132	2.0E-06	Nap1l5	ENSRNOG00000007808	4	89,151,184
24747	A_64_P157405	-1.076	0.093	1.6E-10	-1.470	0.137	6.0E-10	Reep1	ENSRNOG00000008481	4	99,618,622
19570	A_64_P027404	-0.682	0.091	1.3E-07	-0.647	0.128	4.2E-05	Mat2a	ENSRNOG00000013520	4	100,303,080
6906	A_64_P130496	-0.596	0.093	2.3E-06	-0.685	0.136	5.9E-05		ENSRNOG00000013605	4	100,380,578
20592	A_44_P402507	-1.036	0.075	1.4E-12	-0.969	0.106	4.2E-09	Retsat	ENSRNOG00000014090	4	100,465,170

Disease Models & Mechanisms • Supplementary information

6125	A_44_P399999	0.424	0.055	9.6E-08	0.391	0.078	4.9E-05	Suclg1	ENSRNOG00000005587	4	101,180,404
4924	A_42_P508921	1.020	0.100	5.6E-10	0.910	0.141	1.5E-06	Aqp3	ENSRNOG00000009797	5	57,429,245
321	A_42_P698240	-1.913	0.081	1.6E-17	-2.051	0.115	6.6E-15	Ptgr1	ENSRNOG00000015072	5	76,129,441
22698	A_43_P13226	0.622	0.124	4.7E-05	-1.241	0.176	3.5E-07	Slc31a1	ENSRNOG00000014475	5	78,222,504
29448	A_64_P090543	-0.752	0.072	3.7E-10	-0.649	0.102	1.7E-06	Hdhd3	ENSRNOG00000015195	5	78,361,647
29767	A_44_P454532	1.140	0.158	2.5E-07	1.139	0.223	3.7E-05	Ambp	ENSRNOG00000006889	5	78,985,990
13242	A_44_P1040796	0.831	0.066	1.0E-11	1.018	0.094	1.7E-10	Atp6v1g1	ENSRNOG00000008163	5	79,367,663
19537	A_44_P996124	0.557	0.091	3.6E-06	0.999	0.124	5.4E-08	Tlr4	ENSRNOG00000010522	5	82,587,420
12084	A_64_P061875	0.805	0.096	2.0E-08	-1.280	0.135	2.4E-09		ENSRNOG00000056007	5	110,978,644
15856	A_64_P065280	0.646	0.076	1.8E-08	0.593	0.108	1.4E-05	Fam151a	ENSRNOG00000007799	5	126,334,803
23037	A_43_P15784	-0.928	0.076	1.7E-11	-1.583	0.107	3.7E-13	Cyp4a3	ENSRNOG00000009741	5	134,484,839
3744	A_43_P10290	-2.605	0.107	8.3E-18	-2.319	0.152	1.8E-13	Tspan1	ENSRNOG00000023320	5	135,024,447
9670	A_44_P117119	1.177	0.079	3.3E-13	2.175	0.112	1.1E-15	Hpdl	ENSRNOG00000018143	5	135,677,432
10831	A_64_P362701	-1.326	0.163	3.3E-08	-1.575	0.230	5.8E-07	RGD1563714	ENSRNOG00000031494	5	136,112,344
16661	A_44_P511294	-0.494	0.070	3.7E-07	-0.686	0.099	4.9E-07	Tmem53	ENSRNOG00000019186	5	136,112,417
9897	A_44_P314295	-1.575	0.099	2.3E-12	-1.507	0.213	1.1E-06	Zmynd12	ENSRNOG00000022199	5	138,470,069
955	A_64_P032604	0.581	0.065	7.3E-09	0.766	0.092	2.5E-08	Ak2	ENSRNOG00000000122	5	147,185,474
1608	A_64_P032611	0.726	0.064	8.5E-11	0.734	0.091	4.1E-08	Ak2	ENSRNOG00000000122	5	147,185,474
22000	A_64_P007550	-1.576	0.119	1.3E-11	-0.895	0.175	4.7E-05	Matn1	ENSRNOG00000010932	5	149,077,412
23745	A_44_P175875	0.818	0.090	5.0E-09	1.174	0.128	3.8E-09	Pigv	ENSRNOG00000000121	5	151,895,016
8203	A_44_P1004790	1.334	0.097	1.5E-12	0.810	0.137	5.0E-06	Slc30a2	ENSRNOG00000054142	5	152,559,577
28901	A_44_P429298	-1.254	0.167	1.8E-07	-1.444	0.228	2.3E-06	Tnfrsf9	ENSRNOG00000036942	5	168,009,393
4895	A_64_P146507	0.607	0.101	3.9E-06	0.838	0.142	5.4E-06		ENSRNOG00000042062	5	173,139,628
23507	A_64_P160689	0.737	0.118	2.4E-06	-1.092	0.167	1.2E-06		ENSRNOG00000062235	6	11,580,965
26647	A_43_P11724	0.945	0.137	5.5E-06	1.163	0.202	4.0E-05	Gckr	ENSRNOG00000048874	6	26,385,761
24275	A_44_P413979	0.790	0.074	2.3E-10	0.562	0.104	1.9E-05	Rab10	ENSRNOG00000047088	6	27,721,120
3618	A_44_P377278	-0.759	0.114	1.2E-06	-0.806	0.156	3.6E-05	Adcy3	ENSRNOG00000003999	6	28,571,351
12750	A_64_P147334	-0.686	0.099	5.1E-07	-0.844	0.140	4.1E-06	Fkbp1b	ENSRNOG00000047143	6	29,977,797
27573	A_44_P945846	0.870	0.172	4.1E-05	1.163	0.243	8.2E-05	Twistnb	ENSRNOG00000010750	6	52,751,106
22866	A_64_P123690	0.582	0.099	6.0E-06	-1.127	0.141	4.4E-08	RGD1563613	ENSRNOG00000029737	6	57,396,220
18839	A_44_P363009	0.962	0.180	2.0E-05	1.422	0.254	1.1E-05	Scin	ENSRNOG00000004498	6	60,054,279
9915	A_64_P045102	0.704	0.114	2.7E-06	-1.259	0.161	6.6E-08		ENSRNOG00000032000	6	96,202,477
5759	A_64_P065368	0.610	0.077	5.8E-08	0.852	0.109	7.1E-08	Acot4	ENSRNOG00000046864	6	107,517,668

Disease Models & Mechanisms • Supplementary information

16745	A_64_P067447	0.718	0.075	2.0E-09	0.747	0.106	4.0E-07	Acot4	ENSRNOG00000046864	6	107,517,668
18088	A_44_P337991	0.592	0.086	5.5E-07	0.962	0.122	5.6E-08	Acot3	ENSRNOG00000053460	6	107,531,528
28805	A_64_P028095	-0.544	0.109	5.0E-05	-0.839	0.155	1.7E-05	Abcd4	ENSRNOG00000011964	6	108,329,464
4675	A_44_P539231	0.481	0.068	3.1E-07	-0.825	0.095	1.2E-08		ENSRNOG00000042542	6	115,769,793
7028	A_43_P13410	0.885	0.122	2.5E-07	1.297	0.173	1.4E-07	Serpina4	ENSRNOG00000009788	6	127,743,971
11175	A_64_P002455	-1.025	0.100	5.3E-10	-0.793	0.141	1.1E-05	LOC690422	ENSRNOG00000005153	6	137,967,401
17240	A_44_P147572	-2.227	0.145	1.7E-13	-1.495	0.205	2.2E-07		ENSRNOG00000034190	6	138,508,753
10291	A_64_P095554	-2.609	0.353	1.7E-07	-3.259	0.499	1.2E-06		ENSRNOG00000034190	6	138,632,203
10600	A_64_P099017	-2.008	0.064	1.2E-17	-1.944	0.139	2.1E-11	Cyp4f17	ENSRNOG00000062306	7	14,529,483
23692	A_64_P044648	0.604	0.098	3.0E-06	-0.735	0.139	2.4E-05		ENSRNOG00000048170	7	20,075,974
29455	A_64_P130573	0.604	0.093	1.3E-06	-0.980	0.132	1.5E-07		ENSRNOG00000032424	7	29,909,120
9888	A_64_P025286	0.921	0.067	1.4E-12	1.177	0.094	1.1E-11	Lyz2	ENSRNOG00000005825	7	60,341,264
18940	A_64_P072442	0.903	0.118	1.0E-07	1.160	0.167	4.9E-07	Tsfm	ENSRNOG00000048843	7	70,319,346
23558	A_64_P144913	0.437	0.091	7.4E-05	0.859	0.128	8.1E-07	Nab2	ENSRNOG00000008415	7	70,969,905
19834	A_64_P036553	1.809	0.112	6.1E-14	2.053	0.159	5.7E-12	Rdh2	ENSRNOG00000029651	7	70,980,422
7840	A_44_P318662	-1.097	0.165	9.2E-07	-1.329	0.233	8.7E-06	Tac2	ENSRNOG00000004229	7	71,023,976
27443	A_64_P036548	1.168	0.145	4.0E-08	1.277	0.205	2.4E-06	Rdh2	ENSRNOG00000029651	7	71,057,911
14630	A_64_P134465	-0.465	0.086	1.9E-05	-0.638	0.122	2.8E-05	Fam91a1	ENSRNOG00000008271	7	98,302,953
16387	A_64_P012232	-1.133	0.100	8.4E-11	-1.352	0.142	2.1E-09	Sqle	ENSRNOG00000009550	7	99,609,191
2500	A_64_P065611	1.029	0.099	3.8E-10	1.310	0.140	2.7E-09	Khdrbs3	ENSRNOG00000009539	7	110,031,696
26593	A_44_P1049252	0.456	0.059	7.5E-08	0.410	0.083	5.5E-05	Naprt1	ENSRNOG00000007939	7	116,926,555
18442	A_64_P044345	0.698	0.091	8.5E-08	0.784	0.128	3.1E-06	Plec	ENSRNOG00000023781	7	117,267,803
7795	A_64_P044137	-0.499	0.102	6.1E-05	-0.738	0.144	3.5E-05	Lrrc24	ENSRNOG00000016204	7	117,784,638
28509	A_44_P267706	0.869	0.101	1.4E-08	1.119	0.143	6.9E-08	Apol11a	ENSRNOG00000023122	7	118,840,634
19289	A_44_P975458	-0.966	0.105	3.7E-09	-0.766	0.148	3.1E-05	C1qtnf6	ENSRNOG00000007300	7	119,753,290
25974	A_43_P12557	0.442	0.090	5.9E-05	0.708	0.127	1.2E-05	Polr2f	ENSRNOG00000011214	7	120,380,544
30340	A_44_P930152	1.029	0.145	3.2E-07	1.815	0.205	7.6E-09	Mpped1	ENSRNOG00000010834	7	124,680,294
13547	A_44_P204758	0.747	0.096	6.9E-08	0.759	0.135	1.1E-05	Sult4a1	ENSRNOG00000046975	7	124,982,566
4907	A_64_P138430	-0.941	0.123	1.4E-07	-0.908	0.168	2.1E-05	Odf3b	ENSRNOG00000037060	7	130,350,570
26026	A_64_P060569	-0.530	0.072	1.7E-07	-0.638	0.102	2.1E-06	Arsa	ENSRNOG00000012953	7	130,451,283
25452	A_64_P012962	-1.813	0.089	3.2E-15	-1.931	0.131	1.8E-12		ENSRNOG00000054046	7	143,239,617
15494	A_44_P138838	-3.657	0.169	1.0E-16	-3.757	0.239	9.9E-14	Krt76	ENSRNOG00000031785	7	143,497,108
17711	A_44_P119527	-1.418	0.128	1.2E-10	-1.651	0.181	4.5E-09	Med17	ENSRNOG00000051989	8	13,526,025

Disease Models & Mechanisms • Supplementary information

8626	A_44_P100886	1.773	0.196	4.6E-08	1.504	0.266	2.4E-05		ENSRNOG00000010999	8	13,909,188
11775	A_44_P518434	-1.043	0.090	5.3E-11	-1.125	0.128	8.5E-09	Mrpl4	ENSRNOG00000020659	8	22,021,213
29723	A_64_P148966	0.394	0.067	5.2E-06	0.443	0.094	9.9E-05		ENSRNOG00000009373	8	33,075,211
15342	A_44_P161233	0.460	0.097	8.5E-05	0.674	0.137	5.6E-05	Il18	ENSRNOG00000009848	8	54,993,859
8832	A_42_P794120	-0.520	0.103	4.3E-05	-1.071	0.146	2.0E-07	Stra6	ENSRNOG00000008312	8	62,925,357
26700	A_64_P151869	0.713	0.097	1.8E-07	0.773	0.137	9.8E-06	Myo5a	ENSRNOG00000058866	8	82,037,977
8558	A_64_P080566	0.281	0.049	7.0E-06	0.360	0.069	2.6E-05	LOC494499	ENSRNOG00000000201	8	85,553,734
21497	A_44_P604824	-1.261	0.237	2.1E-05	-1.617	0.335	7.3E-05	Prss35	ENSRNOG00000025184	8	94,423,808
12498	A_44_P162115	1.860	0.108	1.4E-14	1.283	0.152	1.9E-08	Slc9a9	ENSRNOG00000008554	8	102,304,095
20855	A_64_P026828	-0.398	0.081	5.7E-05	-0.551	0.114	7.2E-05	Abhd14a	ENSRNOG00000011936	8	115,149,527
5815	A_43_P11432	-0.388	0.053	1.9E-07	-0.400	0.075	1.9E-05	Acaa1a	ENSRNOG00000032908	8	128,027,958
25489	A_44_P387780	4.859	0.122	2.2E-20	3.389	0.161	5.2E-15	Cyp8b1	ENSRNOG00000019481	8	130,550,388
588	A_44_P388755	1.634	0.112	5.5E-11	1.417	0.150	3.9E-08	Kif15	ENSRNOG00000060356	8	132,032,944
22889	A_44_P342756	0.532	0.084	2.1E-06	-0.981	0.119	2.9E-08		ENSRNOG00000042034	9	25,203,534
17867	A_44_P1029892	1.788	0.080	5.8E-17	2.189	0.114	1.3E-15	Tmem14a	ENSRNOG00000046593	9	27,343,853
17848	A_64_P004231	0.398	0.083	7.8E-05	0.643	0.117	1.5E-05	Gsta5	ENSRNOG00000056847	9	27,402,381
3838	A_42_P624195	0.471	0.076	2.5E-06	0.643	0.107	4.2E-06	Gsta3	ENSRNOG00000013484	9	27,452,902
2478	A_42_P623733	0.568	0.091	2.3E-06	0.701	0.129	1.6E-05	LOC501110	ENSRNOG00000033402	9	27,511,176
19994	A_64_P097257	-0.994	0.188	3.7E-05	-1.845	0.249	3.9E-07		ENSRNOG00000058900	9	29,356,963
6671	A_64_P159044	0.477	0.091	2.6E-05	-1.160	0.129	5.5E-09	LOC685674	ENSRNOG00000055155	9	56,454,959
10938	A_44_P998060	0.470	0.092	4.4E-05	-0.868	0.126	6.6E-07	Mobkl3	ENSRNOG00000014980	9	61,720,583
9937	A_64_P024841	-2.098	0.131	7.0E-14	-1.666	0.186	6.1E-09	Cryga	ENSRNOG00000014790	9	71,830,730
27465	A_64_P055450	-0.426	0.075	8.5E-06	-0.497	0.106	9.8E-05	Idh1	ENSRNOG00000015020	9	71,900,044
8097	A_44_P1031737	-0.838	0.096	9.6E-09	-1.012	0.135	1.4E-07	Pecr	ENSRNOG00000055295	9	79,630,536
29277	A_64_P008502	-0.941	0.176	2.0E-05	-1.831	0.249	1.9E-07	Tmem169	ENSRNOG00000016091	9	79,630,604
21241	A_64_P123879	-0.474	0.078	3.5E-06	-0.545	0.110	5.6E-05	Xrcc5	ENSRNOG00000016105	9	79,659,251
11810	A_43_P12005	-0.809	0.063	7.3E-12	-0.475	0.090	2.3E-05	Resp18	ENSRNOG00000019704	9	82,477,181
18795	A_43_P10901	1.248	0.089	9.9E-13	1.153	0.125	3.9E-09	Itm2c	ENSRNOG00000017359	9	92,916,469
273	A_44_P143922	0.494	0.063	7.0E-08	0.562	0.090	2.2E-06	Hes6	ENSRNOG00000020194	9	98,551,410
11494	A_44_P123492	-0.585	0.085	5.6E-07	-0.644	0.120	2.0E-05	Epb41l3	ENSRNOG00000016724	9	117,538,009
21591	A_64_P058107	-0.515	0.062	2.5E-08	-0.491	0.088	1.1E-05	Enpp6	ENSRNOG00000053864	10	197,050
19341	A_44_P274762	0.447	0.092	6.3E-05	0.692	0.129	2.0E-05	Cpped1	ENSRNOG00000015118	10	3,655,038
21059	A_64_P066501	-0.509	0.068	1.4E-07	-1.746	0.096	4.8E-15	Gng13	ENSRNOG00000039350	10	15,088,935

Disease Models & Mechanisms • Supplementary information

23671	A_64_P062663	-0.527	0.067	5.4E-08	-1.596	0.094	2.1E-14	Gng13	ENSRNOG00000039350	10	15,088,935
6367	A_64_P133657	-2.225	0.134	3.2E-14	-2.614	0.190	1.5E-12	Timd2	ENSRNOG00000021345	10	31,790,263
9066	A_64_P027288	0.863	0.122	1.4E-05	1.485	0.234	3.9E-05	RGD1564516	ENSRNOG00000038894	10	31,880,918
24608	A_64_P025618	-0.504	0.077	1.0E-06	-0.620	0.108	7.9E-06	Slc25a35	ENSRNOG00000004668	10	55,555,089
92	A_42_P520349	0.810	0.122	9.5E-07	0.840	0.173	6.8E-05	Clec10a	ENSRNOG00000018715	10	56,764,927
25686	A_43_P23363	0.868	0.146	4.7E-06	1.180	0.206	8.2E-06	Spata22	ENSRNOG00000037307	10	59,893,067
23809	A_44_P1018622	0.594	0.089	9.2E-07	1.496	0.126	3.3E-11	Fam101b	ENSRNOG00000006674	10	64,202,380
5923	A_64_P006628	-1.536	0.228	7.4E-07	-1.522	0.322	9.5E-05	Tac4	ENSRNOG00000004404	10	83,081,168
26032	A_44_P384066	0.603	0.098	2.8E-06	0.731	0.138	2.3E-05	G6pc	ENSRNOG00000053448	10	89,236,258
29333	A_64_P066261	-0.731	0.103	4.2E-07	-0.745	0.140	2.6E-05	Cacng5	ENSRNOG00000003288	10	96,131,880
9292	A_44_P555201	-4.289	0.175	1.3E-14	0.842	0.142	1.8E-05	Apoh	ENSRNOG00000003566	10	96,639,924
9605	A_44_P1011244	-0.649	0.100	1.3E-06	-0.674	0.141	8.3E-05	MGC95210	ENSRNOG00000024780	10	102,199,837
14484	A_64_P050477	0.438	0.070	2.3E-06	0.579	0.099	6.0E-06	Hn1	ENSRNOG00000003661	10	104,075,777
3531	A_64_P059385	0.673	0.071	2.2E-09	0.534	0.100	2.2E-05	Acox1	ENSRNOG00000008755	10	104,748,050
28378	A_64_P059390	0.713	0.080	6.7E-09	0.835	0.113	1.7E-07	Acox1	ENSRNOG00000008755	10	104,748,050
19778	A_64_P131873	-1.814	0.091	4.2E-14	-1.383	0.196	1.1E-06	Galr2	ENSRNOG00000061733	10	105,156,632
10355	A_64_P100346	-0.867	0.116	1.4E-07	-1.143	0.164	4.4E-07	Qrich2	ENSRNOG00000025254	10	105,412,627
21260	A_44_P237153	-2.053	0.197	3.7E-10	-1.847	0.278	9.3E-07	Birc5	ENSRNOG00000050819	10	106,856,097
22449	A_42_P539095	-0.858	0.083	4.2E-10	-0.783	0.117	8.4E-07	LOC688311	ENSRNOG00000049235	10	109,639,054
18404	A_43_P13256	0.278	0.054	3.2E-05	0.454	0.076	4.7E-06	Dcxr	ENSRNOG00000050315	10	109,909,646
5982	A_64_P071367	0.687	0.104	1.0E-06	1.014	0.147	5.3E-07	Krtap16-5	ENSRNOG00000040064	11	28,900,376
20993	A_64_P071362	0.684	0.094	2.3E-07	0.919	0.133	5.2E-07	Krtap16-5	ENSRNOG00000040064	11	28,900,376
12330	A_44_P121280	-1.652	0.153	2.0E-10	-1.682	0.217	7.6E-08	Kcne1	ENSRNOG00000001984	11	32,508,420
16374	A_64_P036352	-1.257	0.098	6.3E-12	-1.096	0.138	5.4E-08	Cbr1	ENSRNOG00000049911	11	33,863,500
3179	A_42_P554722	-0.581	0.072	3.7E-08	-0.517	0.101	3.8E-05	Pigp	ENSRNOG00000039850	11	34,598,275
16198	A_43_P18730	0.976	0.087	9.3E-11	1.199	0.123	1.3E-09	LOC304000	ENSRNOG00000028216	11	36,736,586
6884	A_42_P732251	-1.283	0.097	3.5E-12	-1.429	0.137	3.9E-10	Phldb2	ENSRNOG00000002171	11	57,404,196
6750	A_64_P013875	-1.193	0.096	1.1E-11	-1.107	0.135	3.1E-08		ENSRNOG00000002171	11	57,430,166
1239	A_43_P12572	0.704	0.095	1.6E-07	0.788	0.134	5.7E-06	Cd200	ENSRNOG00000002141	11	60,371,729
10972	A_64_P118532	-0.525	0.077	6.5E-07	-0.623	0.109	8.4E-06	Casr	ENSRNOG00000002265	11	67,188,630
8585	A_64_P101978	-1.128	0.120	2.7E-09	-1.425	0.170	2.0E-08	Cldn16	ENSRNOG00000055138	11	77,703,255
22617	A_44_P635089	-0.514	0.089	6.9E-06	-0.815	0.126	1.3E-06	Etv5	ENSRNOG00000001785	11	82,194,657
26833	A_43_P13230	0.421	0.088	8.0E-05	0.668	0.124	1.9E-05	Ehhadh	ENSRNOG00000001770	11	82,945,104

24638	A_64_P005893	-0.809	0.143	1.1E-05	-1.412	0.195	3.1E-07		ENSRNOG00000050898	11	85,532,526
10269	A_64_P126226	-0.606	0.116	2.8E-05	-0.832	0.165	4.2E-05	Car15	ENSRNOG00000037983	11	87,762,301
7350	A_64_P048136	-0.719	0.086	3.0E-08	-0.622	0.117	2.6E-05	Brca2	ENSRNOG00000001111	12	504,007
1534	A_42_P762213	-0.752	0.087	1.1E-08	-0.639	0.123	2.9E-05	Lrrc8e	ENSRNOG00000028460	12	2,568,382
3604	A_42_P746109	-0.715	0.072	9.9E-10	-0.604	0.102	5.1E-06	Asmtl	ENSRNOG00000028166	12	18,531,990
19145	A_64_P081501	1.197	0.095	8.6E-12	1.895	0.134	8.7E-13	Mcm7	ENSRNOG00000001349	12	19,314,016
1742	A_64_P144034	1.964	0.146	4.2E-11	1.266	0.195	3.4E-06	LOC363894	ENSRNOG00000045875	12	20,814,122
17119	A_44_P654973	0.950	0.110	1.3E-08	1.050	0.156	7.7E-07	Rimbp2	ENSRNOG00000022893	12	31,530,699
28008	A_43_P15918	0.845	0.131	1.4E-06	1.004	0.185	1.6E-05	Rimbp2	ENSRNOG00000022893	12	31,530,699
3175	A_44_P1030714	1.054	0.120	8.4E-09	1.807	0.169	2.4E-10	Ccdc92	ENSRNOG00000021691	12	37,211,316
13761	A_64_P135202	-0.873	0.092	2.4E-09	-0.666	0.131	3.8E-05	Snrnp35	ENSRNOG00000001060	12	37,538,403
5287	A_64_P115222	0.758	0.089	1.5E-08	0.727	0.126	6.9E-06	Pitpnm2	ENSRNOG00000029260	12	37,805,332
29139	A_44_P838992	-0.989	0.145	6.1E-07	-1.085	0.205	2.3E-05	LOC687609	ENSRNOG00000042607	12	38,880,377
2789	A_42_P612939	-0.781	0.134	8.8E-06	-1.053	0.197	2.7E-05	Tmem119	ENSRNOG00000000700	12	48,598,647
14258	A_43_P13249	1.131	0.066	1.7E-14	0.962	0.094	4.9E-10	Acmsd	ENSRNOG00000003884	13	44,424,689
4010	A_64_P083623	0.779	0.085	4.4E-09	-1.247	0.121	4.4E-10		ENSRNOG00000022174	13	56,262,190
12995	A_44_P349273	1.791	0.124	6.1E-13	1.467	0.176	2.2E-08	F13b	ENSRNOG00000012613	13	56,598,957
19291	A_44_P475444	0.393	0.080	6.4E-05	0.613	0.114	1.8E-05	RGD1564614	ENSRNOG00000042369	13	56,958,549
2005	A_42_P819656	0.652	0.081	3.8E-08	0.593	0.114	2.9E-05	Cfh	ENSRNOG00000030715	13	57,080,549
11876	A_64_P068222	0.662	0.093	9.2E-07	2.042	0.200	4.1E-09	Fam129a	ENSRNOG00000002403	13	68,949,665
20329	A_44_P538789	0.350	0.071	6.2E-05	-0.579	0.101	8.0E-06		ENSRNOG00000051105	13	79,493,365
1416	A_64_P091088	0.857	0.105	3.3E-08	1.112	0.149	1.4E-07	Fmo2	ENSRNOG00000003510	13	80,775,264
20699	A_44_P486312	1.127	0.100	7.8E-11	1.193	0.141	1.7E-08	Fmo2	ENSRNOG00000003510	13	80,775,264
14514	A_44_P180259	1.452	0.084	1.5E-14	1.835	0.119	1.6E-13	Dpt	ENSRNOG00000002947	13	83,073,550
16004	A_64_P086314	0.397	0.070	9.0E-06	-0.684	0.099	4.9E-07		ENSRNOG00000054035	13	84,272,663
22348	A_44_P1018997	-0.559	0.082	5.7E-07	-0.571	0.115	5.4E-05	Tmco1	ENSRNOG00000003928	13	85,465,792
12064	A_64_P027625	-4.487	0.123	2.5E-20	-0.888	0.159	1.6E-05	Rxrg	ENSRNOG00000004537	13	85,818,427
21779	A_44_P306439	0.806	0.113	3.2E-07	1.284	0.160	4.5E-08	Fcgr3a	ENSRNOG00000024382	13	89,385,859
25592	A_44_P575255	1.966	0.113	1.2E-14	2.153	0.160	2.6E-12	RGD1562658	ENSRNOG00000034221	13	89,480,058
20771	A_64_P001255	-0.533	0.075	3.1E-07	-0.749	0.106	3.4E-07	Ppox	ENSRNOG00000003567	13	89,654,244
22158	A_64_P003491	-1.903	0.193	1.7E-09	-2.045	0.263	1.0E-07	LOC689766	ENSRNOG00000021411	13	95,887,708
9059	A_42_P803590	-1.682	0.159	2.9E-10	-1.715	0.225	1.0E-07	LOC289378	ENSRNOG00000046460	13	109,663,364
3954	A_42_P661065	0.694	0.138	4.4E-05	1.168	0.195	4.3E-06	Ints7	ENSRNOG00000004263	13	110,257,571

10206	A_64_P052464	1.019	0.162	2.2E-06	1.358	0.230	5.2E-06	Ints7	ENSRNOG00000004263	13	110,257,571
29630	A_64_P015335	1.814	0.128	8.7E-13	2.207	0.181	1.8E-11	Mfsd7	ENSRNOG00000023937	14	2,311,122
783	A_64_P071159	-3.773	0.096	1.7E-22	-4.096	0.135	6.3E-20	LOC360919	ENSRNOG00000039504	14	19,072,677
17181	A_64_P071158	-3.726	0.098	4.2E-22	-4.197	0.139	6.9E-20	LOC360919	ENSRNOG00000039504	14	19,072,677
16082	A_44_P353446	-2.403	0.169	8.0E-13	-2.285	0.239	1.9E-09	Afm	ENSRNOG00000002878	14	19,132,208
7897	A_43_P13995	1.707	0.127	2.4E-12	1.974	0.179	1.3E-10	Gc	ENSRNOG00000003119	14	20,266,891
8034	A_43_P12233	0.460	0.077	4.2E-06	0.843	0.108	7.4E-08	Sult1b1	ENSRNOG00000001967	14	22,142,364
27643	A_64_P053257	0.449	0.070	1.6E-06	0.721	0.099	2.2E-07	Sult1b1	ENSRNOG00000001967	14	22,142,364
16339	A_43_P11686	0.508	0.071	2.6E-07	0.478	0.100	8.2E-05	Kdr	ENSRNOG00000046829	14	34,727,623
28582	A_43_P11601	-1.099	0.092	2.5E-11	-1.016	0.130	6.4E-08	Sod3	ENSRNOG00000003869	14	60,964,324
28877	A_64_P043150	-0.425	0.071	1.1E-05	0.974	0.157	7.9E-06	Ccdc157	ENSRNOG00000005147	14	84,393,421
18032	A_44_P402445	1.950	0.085	1.1E-14	1.630	0.177	3.5E-08	Nefh	ENSRNOG00000008716	14	85,191,557
11690	A_44_P330188	2.958	0.094	2.8E-20	2.380	0.133	6.5E-15	Acox2	ENSRNOG00000007378	15	18,451,144
17115	A_64_P031249	0.834	0.080	4.2E-10	1.240	0.114	1.6E-10	Gnpnat1	ENSRNOG00000008641	15	19,733,967
1315	A_64_P134160	-2.289	0.183	2.0E-11	-2.448	0.279	1.3E-08	Defb42	ENSRNOG00000038762	15	46,166,335
22837	A_44_P428988	0.516	0.071	2.5E-07	-1.035	0.101	5.3E-10		ENSRNOG00000038122	15	66,165,529
1825	A_42_P787239	0.644	0.079	3.3E-08	0.729	0.112	1.2E-06	Farp1	ENSRNOG00000011203	15	106,434,177
7588	A_64_P029008	-0.400	0.069	6.2E-06	-0.604	0.097	2.4E-06	Chdh	ENSRNOG00000015859	16	6,078,122
30347	A_64_P066923	0.800	0.113	3.7E-07	1.173	0.160	2.0E-07	Ncoa4	ENSRNOG00000019768	16	8,302,950
1186	A_42_P467381	0.539	0.073	1.9E-07	0.712	0.104	5.7E-07	Fam32a	ENSRNOG00000039528	16	19,308,842
8703	A_64_P085936	-2.522	0.149	2.2E-14	-2.728	0.211	5.8E-12	Mef2b	ENSRNOG00000020400	16	21,017,163
25971	A_64_P004531	-0.970	0.089	1.7E-10	-0.776	0.126	3.0E-06	LOC688966	ENSRNOG00000020427	16	21,029,134
10778	A_44_P204704	0.593	0.114	2.9E-05	1.005	0.161	2.4E-06	Cpe	ENSRNOG00000043387	16	26,906,716
8243	A_44_P652899	-0.545	0.112	6.7E-05	-0.754	0.158	8.7E-05	Gpm6a	ENSRNOG00000010731	16	39,719,187
26075	A_64_P041016	-0.813	0.084	1.4E-09	-0.912	0.118	8.5E-08	Wwc2	ENSRNOG00000013248	16	47,368,768
171	A_64_P040806	0.816	0.092	7.6E-09	0.812	0.130	2.4E-06	Tlr3	ENSRNOG00000021726	16	50,016,857
11412	A_44_P1042163	0.640	0.089	2.9E-07	1.154	0.126	4.4E-09	RGD1310414	ENSRNOG00000023494	16	64,745,207
7494	A_42_P633958	0.552	0.101	1.6E-05	0.927	0.143	1.4E-06	Col4a2	ENSRNOG00000023972	16	83,438,561
11386	A_64_P014278	-1.416	0.074	1.6E-15	-1.255	0.105	2.6E-11	LOC680692	ENSRNOG00000018400	17	5,382,034
4329	A_44_P620106	0.596	0.076	7.1E-08	0.962	0.108	7.1E-09	Tgfbi	ENSRNOG00000012216	17	8,429,338
24746	A_64_P163822	-1.348	0.181	1.5E-07	-1.291	0.256	4.3E-05	Cks2	ENSRNOG00000014130	17	13,593,423
4180	A_64_P165035	5.867	0.143	6.5E-23	6.191	0.202	4.6E-20	RGD1305679	ENSRNOG00000014481	17	22,619,891
8900	A_64_P059262	0.837	0.136	2.8E-06	1.295	0.192	7.3E-07	Riok1	ENSRNOG00000014049	17	27,451,832

Disease Models & Mechanisms • Supplementary information

28972	A_64_P060715	-0.873	0.164	2.2E-05	-1.401	0.232	3.8E-06		ENSRNOG00000022505	17	43,458,553
7060	A_42_P555365	0.641	0.067	1.8E-09	0.564	0.094	4.5E-06	Slc17a3	ENSRNOG00000032745	17	43,537,293
14149	A_64_P080667	-1.245	0.135	3.8E-09	-1.270	0.191	9.2E-07	LOC684841	ENSRNOG00000045644	17	43,807,540
18077	A_64_P127848	-1.336	0.108	2.7E-11	-1.486	0.148	1.2E-09	Aoah	ENSRNOG00000054964	17	46,115,004
17105	A_64_P040038	2.659	0.091	1.4E-19	2.441	0.129	1.8E-15	RGD1563564	ENSRNOG00000016217	17	63,145,577
13903	A_44_P515798	1.895	0.162	4.1E-11	2.855	0.229	1.2E-11	Ucma	ENSRNOG00000017987	17	77,261,731
23649	A_44_P191021	0.389	0.073	2.1E-05	0.771	0.103	1.4E-07	Phyh	ENSRNOG00000018044	17	77,304,530
26649	A_64_P059949	0.759	0.110	6.9E-07	0.987	0.150	1.4E-06	C1ql3	ENSRNOG00000017459	17	80,320,681
20714	A_64_P056712	0.621	0.096	1.3E-06	0.819	0.135	3.7E-06	Gpr137b	ENSRNOG00000002480	17	90,696,019
14941	A_64_P041481	-0.573	0.072	4.9E-08	-0.868	0.102	1.5E-08	Mep1b	ENSRNOG00000049345	18	15,089,988
23099	A_44_P173289	-0.846	0.154	1.4E-05	-1.244	0.218	8.5E-06	Pkd2l2	ENSRNOG00000025489	18	27,190,974
2039	A_64_P135224	2.504	0.113	6.5E-17	2.592	0.160	5.2E-14	RGD1309362	ENSRNOG00000038960	18	55,505,993
4992	A_44_P549494	0.841	0.062	2.3E-12	1.046	0.088	3.0E-11	Cd74	ENSRNOG00000018735	18	56,071,478
17166	A_44_P419615	-1.462	0.122	1.5E-10	-1.038	0.203	5.4E-05	Stard6	ENSRNOG00000026324	18	68,983,545
25215	A_64_P118728	-0.597	0.109	1.7E-05	-1.071	0.148	3.2E-07	LOC100192205	ENSRNOG00000059055	18	76,753,902
7335	A_42_P595630	-0.865	0.084	4.4E-10	-0.997	0.118	1.9E-08	Pqlc1	ENSRNOG00000059215	18	76,770,012
22519	A_64_P021320	-0.914	0.074	1.3E-11	-1.069	0.104	5.0E-10	Pqlc1	ENSRNOG00000059215	18	76,770,012
30080	A_64_P110941	-2.256	0.173	4.4E-12	-1.736	0.244	3.2E-07	Cndp1	ENSRNOG00000027739	18	81,499,051
15276	A_44_P1039606	-0.699	0.116	1.9E-05	-2.190	0.317	3.8E-06	Cenpt	ENSRNOG00000024178	19	37,819,789
5405	A_64_P121061	-1.053	0.111	2.3E-09	-1.256	0.157	4.8E-08	Tmed6	ENSRNOG00000020406	19	39,267,928
19705	A_44_P220325	-1.578	0.181	1.4E-08	-1.333	0.246	2.0E-05	Dynlrb2	ENSRNOG00000012450	19	49,016,891
10809	A_44_P374638	-0.899	0.173	2.9E-05	-1.167	0.245	8.4E-05	Hsd17b2	ENSRNOG00000013982	19	50,246,402
4436	A_42_P627572	-1.466	0.120	1.8E-11	-1.367	0.170	4.2E-08	Mdc1	ENSRNOG00000032813	20	3,405,285
7136	A_64_P062110	0.466	0.063	1.5E-07	0.685	0.088	7.9E-08	RT1-DMb	ENSRNOG00000049491	20	3,945,601
1417	A_64_P043911	0.673	0.092	2.1E-07	0.915	0.131	4.0E-07	RT1-Bb	ENSRNOG00000032708	20	4,039,413
27475	A_44_P991532	0.491	0.079	2.5E-06	0.578	0.112	3.1E-05	RT1-Da	ENSRNOG00000032844	20	4,132,616
12415	A_64_P128478	0.831	0.071	3.6E-11	0.854	0.100	1.4E-08	RT1-DMa	ENSRNOG00000047864	20	5,241,496
15230	A_64_P048033	0.727	0.082	6.9E-09	0.778	0.115	7.5E-07	Agpat3	ENSRNOG00000001205	20	11,114,164
25935	A_64_P146698	0.590	0.095	2.4E-06	0.803	0.134	4.2E-06	Pwp2	ENSRNOG00000001210	20	11,228,844
7074	A_64_P001461	-1.188	0.152	8.8E-08	-1.039	0.207	5.0E-05	Dnmt3l	ENSRNOG00000001212	20	11,359,090
19033	A_44_P1015203	0.690	0.059	4.8E-11	0.515	0.084	3.1E-06	Gstt3	ENSRNOG00000001242	20	13,817,795
1320	A_42_P604331	1.412	0.119	3.2E-11	1.564	0.169	3.4E-09	Spock2	ENSRNOG00000061544	20	29,655,226
405	A_42_P559737	0.763	0.080	1.8E-09	0.962	0.113	1.4E-08	Srgn	ENSRNOG00000000394	20	32,133,431

220	A_42_P796980	-2.546	0.211	2.2E-11	-3.337	0.298	9.6E-11	Ros1	ENSRNOG00000000406	20	33,323,367
24586	A_43_P14875	-0.678	0.069	1.2E-09	-0.462	0.098	9.4E-05	Prep	ENSRNOG00000051061	20	50,172,618
18623	A_64_P042519	-1.027	0.107	4.1E-09	-0.787	0.157	6.0E-05	Grik2	ENSRNOG00000000368	20	54,517,709
11936	A_44_P547801	-0.878	0.092	2.2E-09	-0.777	0.131	4.9E-06	Gstt2	ENSRNOG00000052360	L568423	501
1141	A_64_P097927	0.570	0.115	5.5E-05	-0.779	0.163	8.2E-05		ENSRNOG00000031780	MT	1
1339	A_64_P042093	0.940	0.124	1.2E-07	1.710	0.176	1.4E-09		ENSRNOG00000031780	MT	1
1670	A_64_P110465	0.607	0.113	1.9E-05	-1.150	0.160	2.6E-07		ENSRNOG00000031780	MT	1
2034	A_64_P012268	0.617	0.112	1.4E-05	-0.820	0.159	3.2E-05		ENSRNOG00000031780	MT	1
2130	A_64_P030494	0.820	0.100	2.9E-08	-1.525	0.141	2.0E-10		ENSRNOG00000031780	MT	1
3172	A_64_P122755	0.407	0.056	2.3E-07	-0.831	0.079	3.3E-10		ENSRNOG00000031780	MT	1
3666	A_64_P108841	0.682	0.076	5.6E-09	-0.738	0.107	5.1E-07		ENSRNOG00000031780	MT	1
4385	A_42_P471835	0.566	0.085	8.7E-07	0.734	0.120	3.2E-06		ENSRNOG00000031780	MT	1
5091	A_44_P342334	-0.743	0.077	1.8E-09	-0.541	0.109	5.5E-05		ENSRNOG00000031780	MT	1
5336	A_64_P112262	0.660	0.118	1.2E-05	-1.207	0.167	2.5E-07		ENSRNOG00000031780	MT	1
5482	A_64_P326359	1.084	0.087	1.3E-11	0.987	0.124	4.7E-08		ENSRNOG00000031780	MT	1
5682	A_64_P035685	0.726	0.131	1.3E-05	-1.272	0.185	5.5E-07		ENSRNOG00000031780	MT	1
6703	A_64_P005742	0.413	0.083	4.8E-05	-0.661	0.117	9.5E-06		ENSRNOG00000031780	MT	1
6958	A_64_P042096	0.911	0.131	4.5E-07	0.949	0.185	3.5E-05		ENSRNOG00000031780	MT	1
8758	A_64_P147268	0.385	0.063	3.5E-06	-0.844	0.090	2.5E-09		ENSRNOG00000031780	MT	1
9083	A_64_P130249	0.591	0.091	1.4E-06	-0.821	0.129	1.8E-06		ENSRNOG00000031780	MT	1
9336	A_64_P063588	0.655	0.080	3.2E-08	0.717	0.113	2.0E-06		ENSRNOG00000031780	MT	1
9394	A_64_P119214	0.808	0.062	5.5E-12	1.163	0.088	3.8E-12		ENSRNOG00000031780	MT	1
9586	A_64_P007528	0.460	0.073	2.1E-06	-0.725	0.103	4.0E-07		ENSRNOG00000031780	MT	1
10091	A_64_P150008	-0.951	0.124	8.8E-08	-1.025	0.175	5.7E-06		ENSRNOG00000031780	MT	1
10168	A_64_P014010	2.213	0.078	2.5E-19	2.340	0.110	1.5E-16		ENSRNOG00000031780	MT	1
10544	A_64_P085915	-1.055	0.147	2.8E-07	-1.659	0.208	4.8E-08		ENSRNOG00000031780	MT	1
11098	A_44_P156705	0.410	0.071	6.9E-06	-0.648	0.100	1.4E-06		ENSRNOG00000031780	MT	1
11823	A_64_P143271	0.381	0.079	7.6E-05	-0.558	0.112	5.0E-05		ENSRNOG00000031780	MT	1
12614	A_64_P098021	0.688	0.104	9.8E-07	-1.101	0.147	1.4E-07		ENSRNOG00000031780	MT	1
13135	A_64_P131326	0.538	0.110	6.1E-05	-1.217	0.155	6.5E-08		ENSRNOG00000031780	MT	1
13275	A_64_P016797	0.566	0.082	5.0E-07	-1.053	0.116	4.8E-09		ENSRNOG00000031780	MT	1
13523	A_64_P118493	0.530	0.079	7.8E-07	-0.877	0.112	6.3E-08		ENSRNOG00000031780	MT	1
14338	A_64_P031397	0.814	0.083	1.2E-09	-1.157	0.118	1.1E-09		ENSRNOG00000031780	MT	1

Disease Models & Mechanisms • Supplementary information

14637	A_64_P068112	0.333	0.068	6.1E-05	-0.572	0.096	4.7E-06	ENSRNOG00000031780	MT	1
15278	A_64_P013704	-1.317	0.144	1.0E-08	-1.202	0.212	1.3E-05	ENSRNOG00000031780	MT	1
15949	A_64_P024000	0.514	0.089	6.7E-06	-0.812	0.125	1.4E-06	ENSRNOG00000031780	MT	1
16302	A_64_P079528	-0.764	0.155	5.7E-05	-1.161	0.219	2.3E-05	ENSRNOG00000031780	MT	1
16858	A_64_P146089	-1.602	0.188	1.5E-08	-1.613	0.265	3.5E-06	ENSRNOG00000031780	MT	1
17319	A_64_P109342	0.556	0.115	7.7E-05	-0.752	0.156	8.4E-05	ENSRNOG00000031780	MT	1
17552	A_64_P127173	0.542	0.089	3.7E-06	0.663	0.127	2.6E-05	ENSRNOG00000031780	MT	1
17573	A_64_P042099	0.955	0.117	3.0E-08	0.823	0.165	4.9E-05	ENSRNOG00000031780	MT	1
17634	A_64_P011339	0.578	0.071	3.1E-08	-0.988	0.100	1.0E-09	ENSRNOG00000031780	MT	1
19318	A_44_P393985	0.712	0.095	1.3E-07	-0.857	0.134	1.6E-06	ENSRNOG00000031780	MT	1
19343	A_64_P070068	0.762	0.107	3.3E-07	-1.201	0.152	5.5E-08	ENSRNOG00000031780	MT	1
19358	A_64_P114888	0.750	0.103	2.0E-07	-1.181	0.145	3.4E-08	ENSRNOG00000031780	MT	1
21099	A_64_P143316	0.659	0.102	1.3E-06	-1.123	0.144	6.8E-08	ENSRNOG00000031780	MT	1
21135	A_64_P118308	-1.481	0.108	3.6E-12	-1.374	0.148	4.9E-09	ENSRNOG00000031780	MT	1
21748	A_64_P071088	0.827	0.065	6.7E-12	1.108	0.091	2.0E-11	ENSRNOG00000031780	MT	1
22005	A_64_P114908	0.548	0.097	9.2E-06	-1.103	0.137	3.9E-08	ENSRNOG00000031780	MT	1
22436	A_64_P045775	1.023	0.130	5.7E-08	1.071	0.183	6.1E-06	ENSRNOG00000031780	MT	1
22567	A_64_P073048	0.723	0.097	1.6E-07	-1.097	0.138	4.7E-08	ENSRNOG00000031780	MT	1
22908	A_64_P130311	0.332	0.067	5.2E-05	-0.570	0.095	4.0E-06	ENSRNOG00000031780	MT	1
23212	A_44_P189674	0.660	0.105	2.2E-06	-0.952	0.149	1.7E-06	ENSRNOG00000031780	MT	1
23791	A_64_P067858	0.668	0.129	2.9E-05	-1.209	0.182	9.2E-07	ENSRNOG00000031780	MT	1
23878	A_64_P127998	0.496	0.087	9.1E-06	-1.072	0.124	1.1E-08	ENSRNOG00000031780	MT	1
23968	A_64_P039666	0.632	0.122	3.1E-05	-1.239	0.173	2.8E-07	ENSRNOG00000031780	MT	1
24098	A_64_P042811	-1.839	0.077	1.1E-17	-2.237	0.109	3.0E-16	ENSRNOG00000031780	MT	1
24725	A_64_P134912	0.820	0.110	1.4E-07	-1.534	0.155	1.0E-09	ENSRNOG00000031780	MT	1
25303	A_44_P450278	0.478	0.096	4.9E-05	-0.893	0.135	1.0E-06	ENSRNOG00000031780	MT	1
25670	A_64_P084678	0.384	0.078	5.5E-05	-0.587	0.110	2.0E-05	ENSRNOG00000031780	MT	1
28639	A_64_P068703	0.619	0.105	5.2E-06	-0.873	0.148	5.3E-06	ENSRNOG00000031780	MT	1
28808	A_64_P086859	0.612	0.077	4.9E-08	-0.805	0.109	1.6E-07	ENSRNOG00000031780	MT	1
28920	A_64_P087796	0.369	0.061	3.7E-06	-0.705	0.086	3.1E-08	ENSRNOG00000031780	MT	1
29106	A_64_P178321	-2.202	0.188	4.5E-10	-2.067	0.406	6.6E-05	ENSRNOG00000031780	MT	1
30061	A_64_P086167	2.938	0.101	1.7E-19	2.543	0.144	7.9E-15	ENSRNOG00000031780	MT	1
18904	A_64_P115786	2.713	0.096	3.0E-19	2.305	0.136	2.0E-14	ENSRNOG00000009609	X	1,615,920

28256	A_64_P003219	-1.225	0.143	2.0E-08	-1.004	0.195	3.7E-05	Asb11	ENSRNOG00000003452	X	31,780,425
11061	A_64_P095116	0.552	0.088	2.4E-06	-0.924	0.125	1.7E-07		ENSRNOG00000061492	X	50,184,697
14632	A_64_P136780	0.558	0.075	1.5E-07	-1.111	0.106	3.4E-10		ENSRNOG00000038181	X	63,440,163
15663	A_64_P048852	-1.237	0.079	9.6E-14	-1.005	0.111	5.2E-09		ENSRNOG00000006459	X	120,313,696
245	A_64_P024665	0.688	0.085	3.5E-08	0.874	0.120	2.1E-07	LOC688090			
415	A_42_P803752	-1.184	0.184	1.5E-06	-1.491	0.260	8.1E-06	RGD1562146			
6789	A_64_P046733	3.745	0.185	3.5E-12	2.781	0.235	5.9E-09	Idi2l			
7899	A_64_P006498	-1.466	0.142	4.6E-10	-1.852	0.201	3.8E-09	Ces1e			
10999	A_64_P070586	0.621	0.098	2.0E-06	0.804	0.139	7.1E-06	Krtap21-2			
11437	A_64_P154992	-0.595	0.088	8.3E-07	-0.671	0.119	1.2E-05	Trim30			
12669	A_64_P115157	-0.606	0.087	4.5E-07	-1.682	0.123	1.8E-12	Ces1e			
12941	A_64_P141251	0.656	0.125	2.6E-05	-0.997	0.177	1.0E-05	LOC681152			
15275	A_44_P760998	0.660	0.115	9.1E-06	1.019	0.156	1.5E-06	Fam185a			
18349	A_64_P157474	-0.453	0.080	9.4E-06	-0.623	0.113	1.4E-05	LOC500956			
19748	A_44_P142179	0.737	0.091	3.5E-08	0.697	0.128	1.6E-05	LOC679547			
19910	A_44_P138800	-0.699	0.135	3.2E-05	-1.036	0.192	1.7E-05	Gcap14			
22943	A_64_P037000	2.684	0.168	3.7E-13	3.498	0.291	7.9E-11	Fam155a			
23957	A_64_P139208	-1.455	0.140	4.3E-10	-1.841	0.199	3.4E-09	RT1-N1			
24636	A_42_P835445	-2.452	0.142	1.3E-14	-2.696	0.200	2.4E-12	LOC688893			
25709	A_64_P011719	-1.117	0.110	6.2E-10	-1.053	0.155	6.9E-07	Chmp1b			

Genes are regarded as significant for “shared” differential expression when they show $P<1\times10^{-4}$ reproducibly in two types of comparison, ie, SHR/Izm versus WKY/Izm and SHRSP/Izm versus WKY/Izm, with a concordant direction of differential expression.

Table S3D. A list of significant transcripts for shared differential expression in the renal cortex.

no	ProbeName	SHR/lzm (vs WKY/lzm)			SHRSP/lzm (vs WKY/lzm)			GeneName	Ensembl_rat	Chr	Transcription_start_site (Rnor_6.0)
		Fold change (log2-scale)	SE (log2-scale)	P-value	Fold change (log2-scale)	SE (log2-scale)	P-value				
2382	A_64_P063358	1.033	0.182	5.7E-05	1.519	0.171	4.1E-07	Samd5	ENSRNOG00000023549	1	3,763,392
20438	A_64_P063363	0.972	0.177	7.9E-05	1.412	0.167	7.1E-07	Samd5	ENSRNOG00000023549	1	3,763,392
30230	A_44_P1025861	0.693	0.082	7.4E-07	0.720	0.077	2.3E-07	Shprh	ENSRNOG00000014450	1	5,240,180
7233	A_64_P165046	-0.563	0.101	6.9E-05	-0.517	0.095	9.0E-05	RGD1309903	ENSRNOG00000011460	1	13,915,594
8464	A_64_P129293	1.273	0.221	5.0E-05	1.249	0.208	3.4E-05	RGD1306565	ENSRNOG00000031700	1	15,412,603
11055	A_44_P128306	0.372	0.068	8.1E-05	0.532	0.064	8.8E-07	Aldh8a1	ENSRNOG00000014907	1	16,910,069
914	A_44_P353836	-0.809	0.111	6.0E-06	-0.635	0.096	1.7E-05	RGD1306962	ENSRNOG00000016371	1	22,748,422
23955	A_64_P162940	-1.796	0.089	9.5E-12	-1.377	0.084	1.5E-10		ENSRNOG00000038251	1	77,782,825
7941	A_42_P689905	1.581	0.090	6.7E-11	2.157	0.085	4.5E-13	Apoc2	ENSRNOG00000018402	1	80,594,136
13915	A_64_P080941	-1.202	0.134	3.7E-07	-0.957	0.127	2.7E-06	LOC102556967	ENSRNOG00000033729	1	99,505,677
19968	A_44_P280954	-1.026	0.124	4.7E-06	-1.442	0.124	1.6E-07	Klk1c10	ENSRNOG00000046297	1	99,894,353
22453	A_44_P163243	-1.414	0.122	1.5E-08	-2.715	0.115	1.1E-12	Klks3	ENSRNOG00000032857	1	100,059,967
21264	A_44_P989658	1.262	0.151	1.4E-06	1.117	0.143	3.0E-06	(Klk4)	ENSRNOG00000032170	1	100,205,147
456	A_64_P016403	0.678	0.058	1.2E-08	0.321	0.054	3.8E-05	Ldha	ENSRNOG00000013009	1	102,900,286
9360	A_43_P11305	-1.940	0.104	2.8E-11	-0.941	0.098	1.5E-07	Tmem86a	ENSRNOG00000013663	1	103,172,987
26600	A_64_P131806	-1.831	0.145	5.2E-09	-0.739	0.137	9.6E-05	Tmem86a	ENSRNOG00000013663	1	103,172,987
14268	A_44_P250476	-0.928	0.082	2.0E-08	-0.714	0.077	2.5E-07	Zdhhc13	ENSRNOG00000014277	1	104,106,245
7527	A_64_P151448	-0.972	0.108	3.3E-07	-0.739	0.101	4.1E-06	Fancf	ENSRNOG00000023968	1	107,232,305
27710	A_44_P273783	-0.765	0.135	7.9E-05	-0.879	0.135	2.0E-05	Aldh1a3	ENSRNOG00000052070	1	127,337,882
4552	A_44_P496255	0.582	0.087	1.1E-05	0.507	0.082	2.5E-05	Slco3a1	ENSRNOG00000032798	1	136,073,540
30158	A_64_P035162	1.078	0.166	1.5E-05	1.223	0.157	1.8E-06	Ntrk3	ENSRNOG00000018674	1	140,262,503
19183	A_64_P133177	-0.799	0.069	1.5E-08	-0.677	0.065	5.7E-08	Sema4b	ENSRNOG00000025167	1	141,986,145
26335	A_64_P157285	0.964	0.081	1.1E-08	1.061	0.076	1.4E-09	RGD1560277 (in	ENSRNOG00000025003	1	142,027,250
25376	A_64_P109232	-2.306	0.137	1.1E-10	-2.115	0.129	1.6E-10	Nmb	ENSRNOG00000011011	1	142,724,511
3363	A_64_P016059	-1.183	0.085	1.3E-09	-1.085	0.080	1.9E-09	Homer2	ENSRNOG00000061450	1	143,535,583
4323	A_64_P028140	-0.661	0.082	1.4E-06	-0.733	0.078	2.0E-07	Plekhb1	ENSRNOG00000018627	1	165,680,206
28025	A_44_P520159	-0.684	0.108	1.8E-05	-0.669	0.101	1.2E-05	P2ry2	ENSRNOG00000019283	1	166,037,424
8799	A_64_P095313	0.751	0.068	2.7E-08	0.417	0.064	1.4E-05		ENSRNOG00000051149	1	166,727,805
14093	A_42_P788740	0.989	0.124	1.4E-06	0.754	0.117	1.5E-05	Folr2	ENSRNOG00000019890	1	166,919,302
11154	A_44_P398300	0.499	0.061	1.1E-06	0.421	0.057	3.8E-06	Fam160a2	ENSRNOG00000017408	1	170,238,890

13671	A_44_P212813	0.759	0.088	5.6E-07	0.977	0.083	1.2E-08	Acsm5	ENSRNOG00000031211	1	189,233,141
29269	A_64_P151353	1.895	0.058	1.4E-14	2.031	0.055	2.3E-15	Acsm5	ENSRNOG00000031211	1	189,241,593
10614	A_44_P395538	-0.402	0.053	2.8E-06	-0.294	0.050	4.4E-05	Rabep2	ENSRNOG00000018462	1	197,839,430
24957	A_64_P011779	0.696	0.105	1.2E-05	0.657	0.099	1.1E-05	Sult1a1	ENSRNOG00000019342	1	198,104,109
18482	A_44_P1002996	-0.516	0.067	2.3E-06	-0.535	0.063	7.6E-07	Ino80e	ENSRNOG00000019960	1	198,298,076
29205	A_44_P1029472	-0.533	0.064	9.4E-07	-0.357	0.061	4.1E-05	RGD1305592	ENSRNOG00000020217	1	198,450,688
15247	A_44_P1005462	1.462	0.141	5.9E-08	1.087	0.133	1.0E-06	Mylpf	ENSRNOG00000017645	1	198,655,742
14224	A_64_P045578	1.842	0.295	2.2E-05	1.944	0.278	6.5E-06	Rnf40	ENSRNOG00000018840	1	199,037,544
29778	A_44_P252921	-0.539	0.074	4.3E-06	-0.499	0.070	5.3E-06	Sec23ip	ENSRNOG00000020411	1	200,167,169
4254	A_42_P784614	0.518	0.093	7.1E-05	0.575	0.088	1.3E-05	Htra1	ENSRNOG00000020533	1	201,499,028
26900	A_44_P1039994	-0.915	0.157	4.3E-05	0.854	0.148	4.7E-05	Irf7	ENSRNOG00000017414	1	214,252,456
21515	A_44_P107596	-1.188	0.205	8.6E-05	-1.271	0.188	2.1E-05	Ctsw (interrupted	ENSRNOG00000027096	1	220,848,153
25349	A_44_P478066	-1.719	0.127	2.0E-09	0.643	0.119	9.7E-05	Incenp	ENSRNOG00000032929	1	225,948,626
20969	A_64_P081982	-1.214	0.121	9.5E-08	-0.949	0.114	9.0E-07	Pgm5	ENSRNOG00000015406	1	242,765,807
19312	A_44_P480433	-1.532	0.113	2.0E-09	-0.979	0.106	2.6E-07	Kazald1	ENSRNOG00000016058	1	264,827,739
25963	A_64_P129182	-1.130	0.136	9.0E-07	-0.805	0.128	2.1E-05	Npm3	ENSRNOG00000017622	1	265,506,046
2901	A_64_P157239	-0.575	0.081	5.8E-06	-0.660	0.077	5.9E-07	~~Sfxn2~~	ENSRNOG00000049308	1	266,333,440
26828	A_64_P043941	-1.751	0.131	2.4E-09	-1.335	0.124	3.7E-08	**Sorcs1**	ENSRNOG00000011313	1	270,472,866
3220	A_64_P066271	-0.742	0.090	9.3E-07	-0.561	0.084	1.1E-05	**Mxi1**	ENSRNOG00000034078	1	274,030,978
17839	A_64_P115991	-0.851	0.091	2.1E-07	-0.636	0.086	3.3E-06	**Mxi1**	ENSRNOG00000034078	1	274,030,978
22136	A_64_P066276	-0.974	0.075	3.7E-09	-0.751	0.071	4.9E-08	**Mxi1**	ENSRNOG00000034078	1	274,030,978
20097	A_44_P183488	0.493	0.078	1.9E-05	0.630	0.074	6.2E-07	**Casp7**	ENSRNOG00000056216	1	277,190,964
15734	A_44_P403005	-0.743	0.096	2.0E-06	-0.733	0.090	1.2E-06	**Trub1**	ENSRNOG00000017321	1	278,311,244
9184	A_43_P21414	-0.946	0.102	2.4E-07	-0.629	0.096	1.3E-05	Atg10	ENSRNOG00000016342	2	20,152,884
13620	A_43_P17958	0.535	0.074	4.6E-06	0.598	0.070	6.4E-07	Atg10	ENSRNOG00000016342	2	20,152,884
6596	A_43_P13126	0.671	0.095	5.9E-06	0.999	0.090	2.5E-08	Acot12	ENSRNOG00000061414	2	20,857,202
8784	A_44_P899264	-0.936	0.081	1.6E-08	-1.235	0.076	2.0E-10	Lhfpl2	ENSRNOG00000011032	2	23,770,721
8031	A_64_P163219	-2.408	0.146	1.3E-09	-1.997	0.110	4.6E-10		ENSRNOG00000018615	2	30,780,121
18451	A_43_P12257	2.413	0.076	2.1E-14	1.563	0.072	3.6E-12	Esm1	ENSRNOG00000010797	2	45,104,305
13783	A_44_P252220	0.524	0.080	1.3E-05	0.488	0.075	1.5E-05	Mocs2	ENSRNOG00000056325	2	46,980,976
5239	A_42_P463754	0.445	0.079	6.5E-05	0.496	0.075	1.1E-05	Itga1	ENSRNOG00000053550	2	47,281,421
27193	A_64_P112889	-1.166	0.155	2.8E-06	-0.985	0.146	9.4E-06	Ccdc152	ENSRNOG00000039473	2	53,140,637
14909	A_64_P092509	-0.593	0.072	9.4E-07	-0.466	0.068	7.3E-06	Fbxo4	ENSRNOG00000015622	2	53,811,134

1843	A_42_P469751	-1.304	0.190	7.8E-06	-1.656	0.179	2.4E-07	C7	ENSRNOG00000061379	2	54,777,729
11752	A_64_P087930	-1.070	0.110	1.4E-07	-1.533	0.104	6.8E-10	C7	ENSRNOG00000061379	2	54,777,729
8100	A_44_P168265	0.704	0.109	1.6E-05	0.865	0.103	7.9E-07	Prkaa1	ENSRNOG00000012799	2	54,857,688
13068	A_44_P226928	-0.827	0.129	1.6E-05	-0.688	0.121	5.9E-05	Pdzd2	ENSRNOG00000013140	2	62,634,785
15982	A_42_P778330	-2.434	0.118	2.7E-11	-2.180	0.102	1.7E-11	Sox2	ENSRNOG00000012199	2	121,165,137
16967	A_44_P773166	-0.672	0.122	7.6E-05	-1.119	0.115	1.3E-07	Tmem144	ENSRNOG00000010081	2	178,612,470
22744	A_64_P055364	-0.670	0.103	1.4E-05	-0.891	0.097	2.8E-07	Tmem144	ENSRNOG00000010081	2	178,612,470
17924	A_64_P151602	-1.397	0.145	1.5E-07	-1.093	0.137	1.4E-06	Trim46	ENSRNOG00000055433	2	188,553,289
6842	A_42_P516658	-1.052	0.101	5.7E-08	-0.996	0.095	5.4E-08	Gatad2b	ENSRNOG00000015553	2	189,655,702
14643	A_43_P12519	-0.561	0.087	1.6E-05	-0.482	0.082	4.3E-05	S100a10	ENSRNOG00000023226	2	193,892,589
17352	A_44_P116875	0.988	0.118	8.5E-07	0.836	0.112	3.0E-06		ENSRNOG00000020836	2	195,617,021
10680	A_64_P140977	-1.175	0.103	1.9E-08	-0.959	0.097	1.2E-07	Prune	ENSRNOG00000021120	2	196,456,481
27712	A_64_P041307	-0.856	0.086	1.0E-07	-0.773	0.081	1.8E-07	Tars2	ENSRNOG00000057194	2	197,878,142
8030	A_44_P393822	0.479	0.086	6.8E-05	0.463	0.081	5.2E-05	Plekho1	ENSRNOG00000021170	2	198,120,120
4450	A_42_P649672	1.667	0.107	3.1E-10	1.759	0.101	7.0E-11	Sv2a	ENSRNOG00000021182	2	198,321,142
29644	A_44_P428326	3.907	0.154	4.6E-13	1.806	0.146	6.3E-09	Hmgcs2	ENSRNOG00000019120	2	200,452,624
24143	A_44_P506299	1.677	0.126	2.6E-09	1.671	0.119	1.3E-09	RGD1562344	ENSRNOG00000037149	2	206,314,213
30118	A_64_P039308	-0.570	0.068	7.5E-07	-0.466	0.064	3.9E-06	Phtf1	ENSRNOG00000019785	2	206,454,208
18048	A_44_P398167	-0.826	0.062	2.5E-09	-0.761	0.058	3.4E-09	Prdx1	ENSRNOG00000017695	2	208,738,132
12324	A_43_P11776	0.952	0.072	3.0E-09	0.417	0.068	2.8E-05	Gstm1	ENSRNOG00000029726	2	210,809,306
25176	A_64_P083399	-0.838	0.127	1.2E-05	-0.879	0.119	3.6E-06		ENSRNOG00000023148	2	216,863,428
27544	A_44_P385704	-1.003	0.115	5.0E-07	-0.971	0.108	3.6E-07		ENSRNOG00000023148	2	216,863,428
8271	A_64_P080876	-0.756	0.105	4.7E-06	-0.671	0.099	9.2E-06	Clca4	ENSRNOG00000029889	2	250,862,419
4532	A_44_P264102	-0.673	0.062	3.6E-08	-0.510	0.059	5.3E-07	Bcl10	ENSRNOG00000042389	2	251,805,392
17603	A_64_P061658	-1.244	0.089	3.4E-09	-0.725	0.077	3.7E-07	Entpd2	ENSRNOG00000013102	3	2,617,805
24094	A_64_P064944	-0.444	0.059	3.0E-06	-0.395	0.056	5.8E-06	Trub2	ENSRNOG00000043189	3	8,348,746
24380	A_64_P151046	-0.610	0.064	1.8E-07	-0.486	0.060	1.3E-06	Trub2	ENSRNOG00000043189	3	8,348,746
29855	A_44_P945862	-0.795	0.076	5.8E-08	-0.595	0.072	9.7E-07	Trub2	ENSRNOG00000043189	3	8,348,746
26230	A_44_P213133	-1.738	0.070	6.2E-13	-1.840	0.066	1.3E-13	Endog	ENSRNOG00000016033	3	8,741,766
21696	A_64_P096782	-0.868	0.101	6.3E-07	-0.633	0.096	1.2E-05	Ier5l	ENSRNOG00000024846	3	9,037,942
6137	A_64_P119821	-1.265	0.083	4.6E-10	-0.906	0.079	1.6E-08	RGD1311084	ENSRNOG00000018486	3	9,636,058
22393	A_43_P18366	1.644	0.084	1.5E-11	1.627	0.079	7.8E-12	Tor1b	ENSRNOG00000006435	3	9,792,899
24771	A_43_P10292	-0.459	0.079	4.8E-05	-0.414	0.075	7.3E-05	siat7D (St6galnac	ENSRNOG00000048870	3	11,607,225

29474	A_44_P532754	-0.576	0.092	2.2E-05	-0.590	0.087	8.9E-06	Pbx3	ENSRNOG00000022162	3	13,435,979
19913	A_44_P102477	-1.700	0.152	2.3E-08	-1.118	0.143	1.8E-06	Morn5	ENSRNOG00000026111	3	15,379,109
9295	A_64_P057740	-0.890	0.127	6.2E-06	-0.862	0.120	4.6E-06	Ccdc148	ENSRNOG00000057740	3	45,210,474
19516	A_64_P074918	0.574	0.093	2.5E-05	0.501	0.088	5.5E-05	Grb14	ENSRNOG00000052498	3	51,054,378
12780	A_64_P025310	-1.558	0.094	1.4E-10	-1.284	0.089	8.6E-10	Galnt3	ENSRNOG00000005727	3	52,212,412
8214	A_64_P077658	-1.615	0.180	3.5E-07	-1.055	0.169	2.2E-05		ENSRNOG00000005868	3	52,361,060
30302	A_64_P308666	-1.744	0.200	5.1E-07	-1.180	0.189	2.2E-05	Ttc21b	ENSRNOG00000005868	3	52,361,060
25593	A_44_P166206	1.049	0.104	8.5E-08	0.644	0.098	1.3E-05	Nostrin	ENSRNOG00000006611	3	55,369,214
17479	A_64_P154292	-0.305	0.053	5.2E-05	-0.406	0.050	1.2E-06	Hnrnpa3	ENSRNOG00000052968	3	62,481,323
26189	A_43_P23211	-0.624	0.104	3.4E-05	-0.562	0.099	5.5E-05	Ssfa2	ENSRNOG00000005865	3	66,593,934
24139	A_44_P992697	-1.587	0.088	4.9E-11	-1.272	0.083	4.2E-10	Dusp19	ENSRNOG00000008868	3	67,849,966
8277	A_64_P022076	1.121	0.070	2.3E-10	0.494	0.066	3.1E-06	Slc43a3	ENSRNOG00000061768	3	72,329,967
17375	A_43_P15275	-1.960	0.075	3.1E-13	-2.078	0.071	6.2E-14	Ptprj	ENSRNOG00000034025	3	79,390,956
13379	A_44_P271769	-0.589	0.076	1.9E-06	-0.551	0.071	2.1E-06	Acp2	ENSRNOG00000013594	3	80,021,440
18771	A_44_P107766	-1.021	0.073	1.3E-09	-0.858	0.069	5.9E-09	RGD1309540	ENSRNOG00000014798	3	80,349,145
25223	A_64_P115142	-0.559	0.090	2.4E-05	-0.636	0.085	3.1E-06	Accs	ENSRNOG00000009199	3	82,756,953
21496	A_44_P808666	0.802	0.085	1.9E-07	0.795	0.080	1.0E-07	Wt1	ENSRNOG00000013074	3	95,133,713
11265	A_43_P14888	-0.662	0.083	1.4E-06	-0.465	0.078	3.4E-05	Scg5	ENSRNOG00000007542	3	105,279,462
15624	A_43_P19265	0.642	0.065	1.2E-07	0.621	0.061	8.3E-08	Shf	ENSRNOG00000028685	3	114,307,250
3427	A_44_P1024721	1.039	0.111	2.1E-07	1.168	0.104	2.3E-08	Slc28a2	ENSRNOG00000028668	3	114,355,798
5981	A_64_P111786	-1.068	0.049	3.7E-12	-0.446	0.046	1.5E-07	Sqrdl	ENSRNOG00000000172	3	114,900,343
16711	A_64_P081944	-0.425	0.068	2.0E-05	-0.384	0.064	3.1E-05	Eid1	ENSRNOG00000008452	3	117,938,500
834	A_42_P561329	-0.897	0.095	1.9E-07	-0.812	0.089	3.1E-07	Anapc1	ENSRNOG00000016965	3	121,226,125
19676	A_64_P098527	-0.708	0.066	4.4E-08	-0.562	0.063	3.6E-07	Chchd5	ENSRNOG00000018491	3	121,660,110
29825	A_64_P000678	0.582	0.082	5.3E-06	0.501	0.077	1.4E-05	Sirpa	ENSRNOG00000004763	3	122,114,754
14641	A_64_P000596	-0.412	0.074	6.9E-05	-0.455	0.070	1.4E-05	Nop56	ENSRNOG00000056237	3	122,807,236
12403	A_44_P398033	0.668	0.074	3.5E-07	1.165	0.070	1.4E-10	Prnp	ENSRNOG00000021259	3	124,515,978
18620	A_64_P125275	0.397	0.073	8.8E-05	0.506	0.069	3.7E-06	Snrpb2	ENSRNOG00000004967	3	137,137,980
18341	A_42_P842833	-0.696	0.081	6.5E-07	-0.790	0.077	6.8E-08	Srxn1	ENSRNOG00000031167	3	147,609,095
12668	A_44_P363204	-0.496	0.080	2.4E-05	-0.424	0.076	6.5E-05	Csnk2a1	ENSRNOG00000005276	3	147,713,821
12542	A_64_P042611	-2.250	0.295	2.5E-06	-1.973	0.279	5.6E-06		ENSRNOG00000036900	3	147,992,754
2111	A_64_P009255	1.315	0.128	7.1E-08	1.174	0.121	1.4E-07	Foxs1	ENSRNOG00000008529	3	148,407,778
2734	A_42_P777935	0.567	0.096	3.9E-05	0.742	0.091	1.1E-06	RGD1311066	ENSRNOG00000020086	3	152,626,757

18249	A_64_P011809	-2.363	0.159	6.2E-10	-1.940	0.150	3.8E-09	RGD1563354	ENSRNOG00000014424	3	154,716,239
5599	A_43_P18817	-1.405	0.144	1.3E-07	-1.291	0.136	1.8E-07	Ift52	ENSRNOG00000007692	3	159,392,193
27964	A_44_P576289	-1.363	0.169	1.3E-06	-1.007	0.159	1.9E-05	Stk4	ENSRNOG00000013529	3	160,467,552
3790	A_42_P589025	-0.848	0.140	3.0E-05	-0.713	0.132	9.6E-05	Slc12a5	ENSRNOG00000018111	3	161,425,988
20958	A_64_P002904	-0.594	0.103	4.7E-05	-0.587	0.097	2.9E-05	Pmepa1	ENSRNOG00000050404	3	171,342,646
16812	A_64_P058749	0.530	0.073	4.4E-06	0.465	0.069	9.7E-06		ENSRNOG00000043150	3	176,479,335
13444	A_42_P623913	0.707	0.107	1.2E-05	0.634	0.101	2.1E-05	Sox18	ENSRNOG00000016248	3	177,179,127
15543	A_44_P307964	-0.743	0.067	2.5E-08	-0.645	0.063	7.2E-08	Polr3k	ENSRNOG00000017843	3	177,374,812
9300	A_64_P054808	0.490	0.069	5.3E-06	0.598	0.065	2.6E-07	Cd36	ENSRNOG00000040108	4	14,001,761
15617	A_64_P113793	0.448	0.071	1.8E-05	0.703	0.066	4.8E-08	Cd36	ENSRNOG00000040108	4	14,151,374
11416	A_64_P113795	0.633	0.106	3.3E-05	0.871	0.100	4.9E-07	LOC685953	ENSRNOG00000062188	4	16,825,767
21357	A_64_P016751	-0.875	0.069	4.6E-09	-0.870	0.065	2.3E-09	Tmem243 (RGD1	ENSRNOG00000042758	4	21,920,651
12659	A_64_P010925	1.207	0.099	7.9E-09	1.442	0.093	3.6E-10	Abcb1b	ENSRNOG00000008012	4	22,307,453
1809	A_43_P12783	-0.615	0.075	1.1E-06	-0.627	0.071	4.5E-07	Col1a2	ENSRNOG00000011292	4	31,534,225
15433	A_44_P959463	1.181	0.132	3.8E-07	1.223	0.124	1.2E-07	AABR07059877.	ENSRNOG00000039626	4	39,727,953
17535	A_64_P072314	0.726	0.067	3.5E-08	0.721	0.063	1.8E-08	AABR07059925.	ENSRNOG00000052702	4	43,915,333
7925	A_64_P015312	-0.542	0.077	6.3E-06	-0.422	0.073	4.8E-05	Fam3c	ENSRNOG00000060349	4	49,439,867
15750	A_64_P077103	-0.580	0.097	3.4E-05	-0.587	0.091	1.6E-05	Kcp	ENSRNOG00000021855	4	56,786,754
29522	A_44_P489995	-0.627	0.050	6.3E-09	-0.600	0.048	5.1E-09	Mrps33	ENSRNOG00000026528	4	67,610,710
12618	A_64_P009490	0.766	0.122	2.1E-05	1.019	0.115	4.3E-07	LOC679651	ENSRNOG00000047635	4	68,011,932
29383	A_44_P228928	0.916	0.131	6.2E-06	1.284	0.123	5.7E-08	Trpv5	ENSRNOG00000015394	4	70,974,006
7316	A_43_P16967	-1.396	0.148	6.9E-07	-1.453	0.136	1.8E-07	Snx10	ENSRNOG00000011944	4	81,311,490
7404	A_43_P23115	1.842	0.119	3.5E-10	2.550	0.112	2.0E-12	Inmt	ENSRNOG00000011250	4	85,386,231
7853	A_44_P758470	-1.096	0.072	4.6E-10	-1.100	0.068	2.0E-10	Ecop	ENSRNOG00000006646	4	88,441,067
13336	A_64_P097974	0.490	0.072	9.1E-06	0.372	0.068	8.5E-05	St3gal5	ENSRNOG00000010284	4	99,937,558
6906	A_64_P130496	-1.252	0.158	1.6E-06	-1.377	0.149	2.5E-07		ENSRNOG00000013605	4	100,380,578
20592	A_44_P402507	-0.772	0.072	3.8E-08	-1.029	0.068	4.4E-10	Retsat	ENSRNOG00000014090	4	100,465,170
6125	A_44_P399999	0.407	0.052	2.0E-06	0.429	0.049	5.4E-07	Suclg1	ENSRNOG00000005587	4	101,180,404
9251	A_64_P019687	-1.130	0.129	4.7E-07	-0.658	0.121	9.0E-05	LOC500227	ENSRNOG00000009917	4	114,854,458
14517	A_44_P1010867	-0.358	0.065	7.4E-05	-0.405	0.061	1.1E-05	Mobkl1b	ENSRNOG00000059474	4	115,024,927
21301	A_44_P268864	0.692	0.081	6.3E-07	0.446	0.076	4.2E-05	Mgll	ENSRNOG00000014508	4	120,671,489
25583	A_64_P128971	1.199	0.153	3.0E-06	1.050	0.146	6.9E-06	Chst13	ENSRNOG00000025930	4	122,237,754
11102	A_44_P917327	1.199	0.183	1.3E-05	1.872	0.172	3.4E-08	Lrtm2	ENSRNOG00000007508	4	151,390,263

9034	A_64_P114913	-1.000	0.134	3.1E-06	-1.189	0.126	2.0E-07	Vwf	ENSRNOG00000019689	4	158,088,505
12161	A_64_P136501	-0.804	0.111	4.2E-06	-1.020	0.104	1.3E-07	Vwf	ENSRNOG00000019689	4	158,088,505
9918	A_64_P033872	-0.964	0.091	4.4E-08	-0.460	0.085	9.6E-05	Etv6	ENSRNOG00000005984	4	167,754,525
11993	A_43_P21564	0.667	0.105	2.7E-05	0.817	0.105	3.2E-06	Tp53inp1	ENSRNOG00000007964	5	24,410,863
5840	A_42_P526030	0.798	0.073	3.0E-08	0.382	0.068	6.8E-05	Decr1	ENSRNOG00000008236	5	29,601,748
17136	A_64_P067968	-0.789	0.109	4.4E-06	-0.850	0.103	9.5E-07	RGD1562865	ENSRNOG00000006170	5	47,546,014
21588	A_42_P680570	-0.682	0.079	5.8E-07	-0.548	0.075	3.7E-06	Nfx1	ENSRNOG00000009015	5	57,291,156
4924	A_42_P508921	1.397	0.114	7.6E-09	1.195	0.108	2.6E-08	Aqp3	ENSRNOG00000009797	5	57,429,245
321	A_42_P698240	-1.872	0.102	3.7E-11	-1.899	0.096	1.4E-11	Ptgr1	ENSRNOG00000015072	5	76,129,441
13424	A_44_P175822	-0.813	0.116	6.1E-06	-0.693	0.109	1.8E-05	Hdhd3	ENSRNOG00000015195	5	78,361,647
29767	A_44_P454532	1.034	0.162	1.7E-05	0.943	0.153	2.5E-05	Ambp	ENSRNOG00000006889	5	78,985,990
13242	A_44_P1040796	0.926	0.054	9.8E-11	1.064	0.051	6.9E-12	Atp6v1g1	ENSRNOG00000008163	5	79,367,663
12834	A_44_P189461	1.108	0.135	1.0E-06	0.829	0.127	1.4E-05	C8b	ENSRNOG00000007639	5	124,300,477
23037	A_43_P15784	-1.177	0.114	6.2E-08	-1.423	0.107	2.6E-09	Cyp4a3	ENSRNOG00000009741	5	134,484,839
16873	A_64_P040870	1.716	0.119	9.2E-10	0.658	0.112	4.3E-05	Cyp4a1	ENSRNOG00000009597	5	134,492,756
3744	A_43_P10290	-3.319	0.093	3.9E-15	-3.022	0.087	6.3E-15	Tspan1	ENSRNOG00000023320	5	135,024,447
9670	A_44_P117119	1.443	0.145	1.0E-07	2.621	0.137	2.1E-11	Hpdl	ENSRNOG00000018143	5	135,677,432
20248	A_64_P138415	0.595	0.107	7.2E-05	0.671	0.101	1.1E-05	Cldn19	ENSRNOG00000007922	5	138,300,107
9897	A_44_P314295	-1.334	0.125	9.1E-07	-0.845	0.109	1.6E-05	Zmynd12	ENSRNOG00000022199	5	138,470,069
7584	A_64_P055067	0.352	0.055	1.7E-05	0.603	0.052	1.4E-08	Ppcs	ENSRNOG00000008572	5	138,470,096
24137	A_64_P114308	-0.677	0.098	7.3E-06	-0.592	0.092	1.7E-05	Heyl	ENSRNOG00000015318	5	140,923,914
13799	A_64_P157944	0.615	0.075	1.0E-06	0.415	0.071	4.0E-05		ENSRNOG00000043364	5	146,973,932
955	A_64_P032604	0.789	0.073	3.8E-08	0.669	0.069	1.4E-07	Ak2	ENSRNOG00000000122	5	147,185,474
1608	A_64_P032611	0.641	0.066	1.4E-07	0.720	0.062	1.6E-08	Ak2	ENSRNOG00000000122	5	147,185,474
4477	A_42_P766913	-0.395	0.064	2.4E-05	-0.378	0.060	2.0E-05	Ccdc28b	ENSRNOG00000047578	5	147,828,449
4551	A_43_P16491	-0.497	0.089	6.6E-05	-0.511	0.084	2.7E-05	Col16a1	ENSRNOG00000031475	5	148,257,642
22186	A_64_P001401	0.613	0.089	7.7E-06	0.584	0.084	7.0E-06	Tinagl1	ENSRNOG00000013179	5	148,392,697
29934	A_64_P110798	-0.646	0.108	3.3E-05	-0.708	0.102	6.6E-06	Serinc2	ENSRNOG00000012989	5	148,492,232
2829	A_43_P15141	-0.538	0.067	1.3E-06	-0.471	0.063	3.0E-06	LOC682988	ENSRNOG00000047303	5	150,653,172
23745	A_44_P175875	0.884	0.101	5.1E-07	0.966	0.096	8.5E-08	Pigv	ENSRNOG00000000121	5	151,895,016
8203	A_44_P1004790	1.129	0.136	9.4E-07	0.691	0.129	1.0E-04	Slc30a2	ENSRNOG00000054142	5	152,559,577
28501	A_64_P036381	1.102	0.128	6.0E-07	0.690	0.121	5.4E-05	Paqr7	ENSRNOG00000022054	5	152,708,775
9445	A_44_P398691	-0.598	0.083	4.8E-06	-0.525	0.078	1.0E-05	Atp13a2	ENSRNOG00000008052	5	159,512,285

7805	A_44_P291028	-0.739	0.100	3.4E-06	-0.939	0.094	1.0E-07	Arhgef19	ENSRNOG00000047967	5	159,755,535
24005	A_64_P001225	-0.916	0.144	1.7E-05	-2.365	0.135	7.0E-11	Mthfr	ENSRNOG00000008553	5	164,845,925
24306	A_44_P999125	1.583	0.101	2.9E-10	0.901	0.095	1.9E-07	Masp2	ENSRNOG00000011258	5	165,415,278
28901	A_44_P429298	-1.738	0.129	2.3E-09	-2.512	0.122	7.7E-12	Tnfrsf9	ENSRNOG00000036942	5	168,009,393
14557	A_44_P272301	-0.587	0.091	1.5E-05	-0.657	0.085	2.2E-06	Mxra8	ENSRNOG00000019244	5	173,288,447
2366	A_42_P538617	-0.451	0.067	9.5E-06	-0.349	0.063	7.3E-05	Ift172	ENSRNOG00000057813	6	26,474,071
3481	A_43_P19743	0.491	0.061	1.2E-06	0.488	0.057	6.6E-07	Krtcap3	ENSRNOG00000047941	6	26,486,695
12750	A_64_P147334	-0.832	0.118	5.7E-06	-0.663	0.111	3.4E-05	Fkbp1b	ENSRNOG00000047143	6	29,977,797
27284	A_43_P14872	0.740	0.119	2.3E-05	0.634	0.112	6.1E-05	Sdc1	ENSRNOG00000059947	6	33,885,495
3351	A_64_P061830	-1.168	0.136	5.9E-07	-0.786	0.128	2.6E-05	Fam84a	ENSRNOG00000004084	6	39,363,458
25705	A_64_P061835	-1.255	0.150	8.0E-07	-0.820	0.141	4.6E-05	Fam84a	ENSRNOG00000004084	6	39,363,458
1766	A_42_P622681	1.705	0.187	3.1E-07	1.732	0.177	1.2E-07	Prkar2b	ENSRNOG00000009079	6	51,356,383
27573	A_44_P945846	0.648	0.103	2.0E-05	1.049	0.097	3.6E-08	Twistnb	ENSRNOG00000010750	6	52,751,106
1431	A_42_P600642	-0.706	0.103	7.9E-06	-0.652	0.097	1.0E-05	Ispd	ENSRNOG00000006199	6	55,881,484
6710	A_64_P008628	-0.528	0.058	3.0E-07	-0.323	0.055	3.9E-05	Ndufa10l1	ENSRNOG00000062245	6	62,798,384
6674	A_64_P080871	-0.611	0.103	3.6E-05	-0.552	0.097	5.6E-05	RGD1307621	ENSRNOG00000023230	6	86,651,196
23405	A_64_P045378	-0.603	0.069	5.2E-07	-0.656	0.065	9.1E-08		ENSRNOG00000056721	6	86,852,323
9006	A_64_P003859	-0.928	0.140	1.2E-05	-0.912	0.132	7.3E-06	Rtn1	ENSRNOG00000004794	6	94,980,004
5759	A_64_P065368	0.836	0.081	6.7E-08	0.789	0.076	6.6E-08	Acot4	ENSRNOG00000046864	6	107,517,668
16745	A_64_P067447	0.827	0.066	6.0E-09	0.703	0.063	2.3E-08	Acot4	ENSRNOG00000046864	6	107,517,668
18088	A_44_P337991	0.638	0.075	7.1E-07	0.737	0.071	6.0E-08	Acot3	ENSRNOG00000053460	6	107,531,528
15326	A_64_P103936	-2.982	0.227	1.8E-08	-1.924	0.171	1.1E-07	Acot5	ENSRNOG00000032508	6	107,550,904
14620	A_64_P124473	-1.305	0.128	7.5E-08	-1.615	0.121	2.3E-09	Ltbp2	ENSRNOG00000012094	6	108,596,569
20332	A_64_P067000	-1.738	0.171	7.7E-08	-0.966	0.161	3.3E-05	Prima1	ENSRNOG00000008915	6	127,127,413
7028	A_43_P13410	1.888	0.157	9.6E-09	2.046	0.148	1.6E-09	Serpina4	ENSRNOG00000009788	6	127,743,971
1711	A_44_P530131	-0.702	0.118	3.6E-05	-0.779	0.111	6.3E-06	Asb2	ENSRNOG00000057803	6	128,989,812
3416	A_64_P019200	-0.756	0.124	7.8E-05	-1.015	0.124	5.3E-06	RGD1560608	ENSRNOG00000043137	6	135,856,218
9408	A_64_P051786	-0.541	0.076	5.1E-06	-0.574	0.071	1.3E-06	Klc1	ENSRNOG00000011572	6	136,330,383
17421	A_64_P018891	-0.724	0.122	3.9E-05	-0.643	0.115	7.0E-05		ENSRNOG00000013661	6	136,720,266
11175	A_64_P002455	-1.007	0.066	4.2E-10	-1.005	0.062	2.0E-10	LOC690422	ENSRNOG00000005153	6	137,967,401
15597	A_64_P103580	-1.172	0.083	1.2E-09	-1.081	0.078	1.6E-09	LOC690422	ENSRNOG00000005153	6	137,967,401
17240	A_44_P147572	-2.190	0.272	1.3E-06	-2.776	0.257	3.6E-08		ENSRNOG00000034190	6	138,508,753
21885	A_44_P643807	-0.374	0.063	3.4E-05	-0.382	0.059	1.5E-05	Arl13b	ENSRNOG00000047194	7	1,188,165

Disease Models & Mechanisms • Supplementary information

15222	A_64_P040620	-0.551	0.052	4.4E-08	-0.365	0.049	3.0E-06	Sarnp	ENSRNOG00000030520	7	3,246,220
5393	A_64_P017836	2.047	0.258	1.5E-06	1.684	0.243	7.0E-06	Creb3l3	ENSRNOG00000032202	7	11,490,852
12776	A_64_P142988	-0.755	0.079	1.6E-07	-0.733	0.074	1.1E-07	Tmprss9	ENSRNOG00000032429	7	11,699,436
28290	A_64_P139250	-0.853	0.094	3.3E-07	-0.737	0.089	9.4E-07	Tmprss9	ENSRNOG00000032429	7	11,699,436
13366	A_64_P085595	-0.371	0.059	2.1E-05	-0.372	0.056	1.1E-05	Ilvbl	ENSRNOG00000028512	7	14,054,639
10600	A_64_P099017	-1.919	0.193	1.0E-07	-1.716	0.182	1.9E-07	Cyp4f17	ENSRNOG00000062306	7	14,529,483
15805	A_64_P255450	-0.860	0.081	4.5E-08	-0.575	0.076	2.7E-06	Zfp799	ENSRNOG00000032552	7	15,072,703
5343	A_64_P088382	2.179	0.212	6.8E-08	1.866	0.200	2.2E-07	Angptl4	ENSRNOG00000007545	7	18,634,079
11774	A_42_P827204	-1.013	0.099	7.2E-08	-0.792	0.093	6.9E-07	Ckap4	ENSRNOG00000008016	7	24,939,498
19762	A_64_P076900	-0.586	0.082	5.0E-06	-0.493	0.077	1.7E-05	Nedd1	ENSRNOG00000004011	7	33,584,564
12870	A_64_P146682	-1.052	0.107	1.2E-07	-1.050	0.101	6.0E-08	Pctk2	ENSRNOG00000004148	7	34,001,506
9888	A_64_P025286	0.983	0.088	2.3E-08	1.231	0.083	5.7E-10	Lyz2	ENSRNOG00000005825	7	60,341,264
18940	A_64_P072442	1.288	0.135	1.7E-07	1.466	0.127	1.7E-08	Tsfm	ENSRNOG00000048843	7	70,319,346
19834	A_64_P036553	1.719	0.105	1.6E-10	1.915	0.099	1.7E-11	Rdh2	ENSRNOG00000029651	7	70,980,422
7840	A_44_P318662	-1.140	0.177	1.5E-05	-1.376	0.167	9.6E-07	Tac2	ENSRNOG00000004229	7	71,023,976
27443	A_64_P036548	0.926	0.121	2.4E-06	1.193	0.114	5.8E-08	Rdh2	ENSRNOG00000029651	7	71,057,911
27543	A_44_P366303	-1.447	0.137	5.0E-08	-1.284	0.129	1.1E-07	Matn2	ENSRNOG00000006060	7	72,985,495
6053	A_44_P417489	-0.731	0.061	1.0E-08	-0.615	0.058	4.4E-08	Utp23	ENSRNOG00000004387	7	91,384,187
27530	A_44_P341075	0.648	0.080	1.2E-06	0.719	0.075	1.7E-07	Enpp2	ENSRNOG00000004089	7	94,563,001
13819	A_44_P324066	-0.782	0.129	3.0E-05	-0.739	0.122	2.9E-05	Fam91a1	ENSRNOG00000008271	7	98,302,953
14630	A_64_P134465	-0.713	0.057	5.9E-09	-0.629	0.054	1.4E-08	Fam91a1	ENSRNOG00000008271	7	98,302,953
16387	A_64_P012232	-1.294	0.123	5.0E-08	-1.247	0.116	3.8E-08	Sqle	ENSRNOG00000009550	7	99,609,191
2500	A_64_P065611	1.405	0.085	1.4E-10	1.336	0.080	1.2E-10	Khdrbs3	ENSRNOG00000009539	7	110,031,696
26593	A_44_P1049252	0.446	0.055	1.2E-06	0.321	0.052	2.4E-05	Naprt1	ENSRNOG00000007939	7	116,926,555
18442	A_64_P044345	0.984	0.089	2.6E-08	0.886	0.083	4.5E-08	Plec	ENSRNOG00000023781	7	117,267,803
21070	A_42_P692476	0.946	0.142	1.1E-05	0.761	0.134	5.8E-05	Slc39a4	ENSRNOG00000014314	7	117,680,004
28100	A_44_P450471	-0.527	0.084	2.0E-05	-0.466	0.079	3.9E-05	Ppp1r16a	ENSRNOG00000015450	7	117,734,002
7795	A_64_P044137	-0.579	0.088	1.3E-05	-0.555	0.083	1.0E-05	Lrrc24	ENSRNOG00000016204	7	117,784,638
28509	A_44_P267706	0.933	0.105	4.2E-07	0.897	0.099	3.3E-07	Apol11a	ENSRNOG00000023122	7	118,840,634
24480	A_44_P286788	0.826	0.116	5.0E-06	0.659	0.109	3.0E-05	Ncf4	ENSRNOG00000006940	7	119,482,272
19289	A_44_P975458	-0.747	0.091	1.1E-06	-0.711	0.086	9.5E-07	C1qtnf6	ENSRNOG00000007300	7	119,753,290
13898	A_44_P445070	-0.906	0.093	1.3E-07	-0.928	0.087	4.6E-08	Lgals1	ENSRNOG00000009884	7	120,153,184
25974	A_43_P12557	0.696	0.058	9.6E-09	0.693	0.055	4.8E-09	Polr2f	ENSRNOG00000011214	7	120,380,544

Disease Models & Mechanisms • Supplementary information

30340	A_44_P930152	1.587	0.106	5.5E-10	1.738	0.100	7.5E-11	Mpped1	ENSRNOG00000010834	7	124,680,294
4907	A_64_P138430	-1.169	0.114	1.4E-07	-1.053	0.099	8.6E-08	Odf3b	ENSRNOG00000037060	7	130,350,570
16698	A_64_P000650	-0.664	0.085	1.7E-06	-0.626	0.080	1.8E-06	Arsa	ENSRNOG00000012953	7	130,451,283
26026	A_64_P060569	-0.566	0.061	2.6E-07	-0.482	0.058	8.9E-07	Arsa	ENSRNOG00000012953	7	130,451,283
25452	A_64_P012962	-2.088	0.106	6.6E-10	-1.872	0.085	2.0E-10		ENSRNOG00000054046	7	143,239,617
15494	A_44_P138838	-3.557	0.138	3.7E-13	-3.767	0.130	7.5E-14	Krt76	ENSRNOG00000031785	7	143,497,108
8405	A_42_P660129	-0.995	0.122	1.1E-06	-0.771	0.115	1.0E-05	Casp12	ENSRNOG00000033434	8	2,659,865
17711	A_44_P119527	-1.887	0.105	4.6E-11	-1.778	0.099	4.6E-11	Med17	ENSRNOG00000051989	8	13,526,025
8626	A_44_P100886	1.345	0.128	2.2E-07	1.368	0.123	1.1E-07		ENSRNOG00000010999	8	13,909,188
11775	A_44_P518434	-0.901	0.080	2.2E-08	-0.763	0.076	8.6E-08	Mrpl4	ENSRNOG00000020659	8	22,021,213
16694	A_64_P153442	-0.454	0.061	3.4E-06	-0.318	0.058	8.0E-05		ENSRNOG00000014331	8	23,282,989
28883	A_44_P177678	-0.770	0.120	1.6E-05	-0.660	0.113	4.4E-05	LOC503192	ENSRNOG00000014331	8	23,282,989
18590	A_64_P025538	-1.055	0.143	3.5E-06	-0.905	0.135	1.0E-05	UBASH3B*	ENSRNOG00000008187	8	45,375,435
20866	A_44_P1030225	1.209	0.135	3.7E-07	1.526	0.127	9.7E-09	Apoa4	ENSRNOG00000055909	8	50,537,009
15342	A_44_P161233	0.456	0.067	8.1E-06	0.401	0.063	1.7E-05	Il18	ENSRNOG00000009848	8	54,993,859
12430	A_43_P14436	-0.908	0.124	3.8E-06	-0.723	0.117	2.4E-05	Pou2af1	ENSRNOG00000011500	8	55,603,968
8832	A_42_P794120	-0.930	0.156	3.6E-05	-1.432	0.147	1.4E-07	Stra6	ENSRNOG00000008312	8	62,925,357
10264	A_64_P149824	0.540	0.077	6.2E-06	0.476	0.073	1.3E-05	Ostbeta	ENSRNOG00000028889	8	70,932,986
26700	A_64_P151869	0.679	0.082	9.9E-07	0.749	0.078	1.5E-07	Myo5a	ENSRNOG00000058866	8	82,037,977
14777	A_64_P156682	-0.946	0.083	1.8E-08	-0.569	0.078	4.0E-06	Hmgn3	ENSRNOG00000031032	8	90,664,554
16214	A_64_P006794	-0.884	0.094	2.0E-07	-0.890	0.088	8.8E-08	Plod2	ENSRNOG00000030183	8	99,977,334
12498	A_44_P162115	2.037	0.086	1.1E-12	2.045	0.081	4.7E-13	Slc9a9	ENSRNOG00000008554	8	102,304,095
1145	A_42_P466706	-1.610	0.122	2.8E-09	-0.903	0.115	1.7E-06	PCOLCE2	ENSRNOG00000046848	8	103,459,161
9017	A_44_P189262	-1.007	0.141	1.2E-05	1.019	0.107	6.0E-07	Camkv	ENSRNOG00000058938	8	116,715,755
11142	A_44_P396460	-0.445	0.063	5.6E-06	-0.319	0.059	9.8E-05	Scap	ENSRNOG00000020853	8	118,570,503
5560	A_64_P047233	0.869	0.109	1.5E-06	0.591	0.103	5.1E-05	Dlec1	ENSRNOG00000032085	8	127,789,048
5404	A_44_P335703	-0.896	0.099	3.2E-07	-0.532	0.093	5.4E-05	Toag1	ENSRNOG00000004085	8	131,845,696
5468	A_43_P22042	0.778	0.086	3.3E-07	1.439	0.081	5.8E-11	Pla2g7	ENSRNOG00000025691	9	19,978,013
17867	A_44_P1029892	2.016	0.077	2.9E-13	2.257	0.073	2.8E-14	Tmem14a	ENSRNOG00000046593	9	27,343,853
13592	A_64_P147029	-0.753	0.085	4.2E-07	-0.681	0.080	6.9E-07	Gulp1	ENSRNOG00000003242	9	51,263,622
22746	A_44_P146518	-0.954	0.088	3.7E-08	-0.895	0.083	3.9E-08	Col3a1	ENSRNOG00000003357	9	52,023,295
10412	A_64_P092419	-0.416	0.070	3.4E-05	-0.484	0.066	3.5E-06	Col5a2	ENSRNOG00000003736	9	52,238,564
22021	A_64_P101204	-0.447	0.063	5.7E-06	-0.331	0.060	7.2E-05	Slc40a1	ENSRNOG00000049995	9	52,830,457

Disease Models & Mechanisms • Supplementary information

4692	A_42_P501877	-0.551	0.072	2.5E-06	-0.473	0.068	7.0E-06	Gls	ENSRNOG00000056246	9	54,212,767
3969	A_42_P825912	-0.627	0.095	1.2E-05	-0.503	0.089	6.2E-05		ENSRNOG00000011677	9	60,039,297
9937	A_64_P024841	-2.160	0.105	7.6E-12	-2.094	0.099	5.2E-12	Cryga	ENSRNOG00000014790	9	71,830,730
25119	A_64_P102930	0.439	0.049	4.1E-07	0.439	0.047	2.0E-07	Atic	ENSRNOG00000015511	9	78,862,013
21094	A_64_P105432	1.038	0.179	4.5E-05	1.271	0.168	2.7E-06		ENSRNOG00000059494	9	79,450,320
29277	A_64_P008502	-1.020	0.072	1.1E-09	-0.912	0.068	2.3E-09	Tmem169	ENSRNOG00000016091	9	79,630,604
21241	A_64_P123879	-0.432	0.057	2.8E-06	-0.475	0.054	4.6E-07	Xrcc5	ENSRNOG00000016105	9	79,659,251
18795	A_43_P10901	1.274	0.100	4.7E-09	1.289	0.095	1.9E-09	Itm2c	ENSRNOG00000017359	9	92,916,469
16972	A_64_P022090	-0.755	0.097	1.8E-06	-0.732	0.091	1.3E-06	Ramp1	ENSRNOG00000019926	9	98,313,632
26373	A_64_P085053	-0.476	0.083	5.4E-05	-0.508	0.078	1.5E-05	Scly	ENSRNOG00000020083	9	98,438,439
26397	A_64_P021433	-0.357	0.065	7.8E-05	-0.381	0.061	2.2E-05	Scly	ENSRNOG00000020083	9	98,438,439
7344	A_64_P010174	0.441	0.074	3.4E-05	0.501	0.069	4.6E-06	Man2a1	ENSRNOG00000015439	9	112,293,388
10864	A_64_P048013	0.657	0.112	4.0E-05	0.681	0.105	1.5E-05	Arhgap28	ENSRNOG00000017065	9	116,222,440
11494	A_44_P123492	-0.746	0.105	5.5E-06	-0.913	0.099	2.6E-07	Epb41l3	ENSRNOG00000016724	9	117,538,009
20373	A_64_P034114	-0.789	0.103	2.3E-06	-0.893	0.097	2.7E-07	Epb41l3	ENSRNOG00000016724	9	117,538,009
21591	A_64_P058107	-0.429	0.080	9.4E-05	-0.556	0.075	3.3E-06	Enpp6	ENSRNOG00000053864	10	197,050
29536	A_44_P288794	-0.458	0.084	8.6E-05	0.701	0.079	4.3E-07	Bfar	ENSRNOG00000003151	10	1,464,765
3252	A_64_P080524	0.680	0.062	2.8E-08	0.648	0.058	2.5E-08	Snn	ENSRNOG00000058739	10	4,644,570
21658	A_64_P010569	0.949	0.084	2.2E-08	0.790	0.079	1.0E-07	Ciita	ENSRNOG00000002659	10	5,260,608
10584	A_44_P337294	1.415	0.089	2.6E-10	0.686	0.084	1.1E-06	Eci1	ENSRNOG00000008843	10	13,797,562
19247	A_64_P077648	0.479	0.084	5.9E-05	0.532	0.080	1.1E-05	Hs3st6	ENSRNOG00000014672	10	14,122,878
1430	A_64_P028788	0.494	0.082	3.1E-05	0.502	0.077	1.4E-05	RGD1311273	ENSRNOG00000020029	10	15,235,740
29411	A_64_P054881	0.428	0.069	2.5E-05	0.503	0.065	2.2E-06	RGD1311273	ENSRNOG00000020029	10	15,235,740
7281	A_64_P062288	0.667	0.093	5.0E-06	0.542	0.088	2.5E-05	Ccdc99	ENSRNOG00000007292	10	19,652,898
24792	A_64_P127609	-1.097	0.110	9.5E-08	-1.107	0.103	4.1E-08	Slit3	ENSRNOG00000007377	10	20,320,878
14617	A_64_P028561	1.457	0.240	3.0E-05	1.496	0.226	1.2E-05		ENSRNOG00000058024	10	29,806,895
6367	A_64_P133657	-2.595	0.109	1.1E-12	-2.529	0.103	7.2E-13	Timd2	ENSRNOG00000021345	10	31,790,263
10135	A_43_P10530	-1.259	0.145	5.2E-07	-1.126	0.137	9.8E-07	Mfap4	ENSRNOG00000002382	10	47,765,432
92	A_42_P520349	1.410	0.115	7.0E-09	0.853	0.108	1.6E-06	Clec10a	ENSRNOG00000018715	10	56,764,927
25686	A_43_P23363	1.348	0.148	3.0E-07	1.606	0.139	1.6E-08	Spata22	ENSRNOG00000037307	10	59,893,067
23809	A_44_P1018622	0.481	0.070	8.2E-06	0.597	0.066	3.4E-07	Fam101b	ENSRNOG00000006674	10	64,202,380
7702	A_42_P695401	-1.290	0.227	5.8E-05	-1.997	0.214	2.3E-07	Ccl2	ENSRNOG00000007159	10	69,412,017
6462	A_64_P099573	-0.902	0.140	1.6E-05	-0.850	0.132	1.6E-05	Rad51l3	ENSRNOG00000021780	10	70,241,254

Disease Models & Mechanisms • Supplementary information

28401	A_44_P373236	0.705	0.115	2.6E-05	1.137	0.108	5.2E-08	Slfn8	ENSRNOG00000021412	10	70,339,578
7469	A_64_P039447	-0.641	0.081	1.5E-06	-0.561	0.076	3.5E-06	Heatr6	ENSRNOG00000002542	10	71,054,344
27034	A_64_P006943	0.987	0.110	3.6E-07	0.931	0.104	3.6E-07	Wfikkn2	ENSRNOG00000002831	10	81,942,188
17149	A_64_P040278	-1.750	0.157	2.5E-08	-1.261	0.148	6.6E-07	Col1a1	ENSRNOG00000003897	10	82,745,801
20882	A_44_P350521	-0.996	0.087	1.9E-08	-0.688	0.082	8.6E-07	Col1a1	ENSRNOG00000003897	10	82,745,801
5923	A_64_P006628	-1.530	0.209	3.9E-06	-1.204	0.197	2.7E-05	Tac4	ENSRNOG00000004404	10	83,081,168
8853	A_44_P401161	-0.783	0.063	6.6E-09	-0.391	0.060	1.3E-05		ENSRNOG00000022375	10	83,147,888
16864	A_43_P16767	0.491	0.090	8.5E-05	0.977	0.085	1.6E-08	Igfbp4	ENSRNOG00000010635	10	86,950,557
27721	A_44_P100207	-1.017	0.078	8.5E-09	-0.979	0.068	2.4E-09	Cnp	ENSRNOG00000017496	10	88,490,798
9605	A_44_P1011244	-0.568	0.079	4.7E-06	-0.635	0.075	6.7E-07	MGC95210	ENSRNOG00000024780	10	102,199,837
14484	A_64_P050477	0.570	0.050	1.9E-08	0.559	0.047	1.1E-08	Hn1	ENSRNOG00000003661	10	104,075,777
3531	A_64_P059385	0.727	0.050	8.4E-10	0.393	0.047	8.9E-07	Acox1	ENSRNOG00000008755	10	104,748,050
28378	A_64_P059390	0.834	0.059	1.1E-09	0.462	0.056	8.8E-07	Acox1	ENSRNOG00000008755	10	104,748,050
10355	A_64_P100346	-1.134	0.120	1.8E-07	-1.061	0.113	2.0E-07	Qrich2	ENSRNOG00000025254	10	105,412,627
1106	A_42_P607183	0.654	0.099	1.1E-05	0.546	0.093	4.1E-05	Ube2o	ENSRNOG00000011007	10	105,552,986
29929	A_44_P497302	-0.731	0.098	3.2E-06	-0.645	0.092	6.6E-06	St6galnac2	ENSRNOG00000012083	10	105,668,593
21260	A_44_P237153	-2.045	0.138	6.3E-10	-1.460	0.130	2.3E-08	Birc5	ENSRNOG00000050819	10	106,856,097
22121	A_44_P147726	-0.632	0.066	1.6E-07	-0.511	0.062	9.8E-07	Pgs1	ENSRNOG00000002949	10	106,991,935
19470	A_44_P1017481	-0.506	0.070	4.5E-06	-0.569	0.066	5.9E-07	Timp2	ENSRNOG00000003148	10	107,386,072
26357	A_64_P094249	-1.120	0.143	1.7E-06	-1.028	0.134	2.4E-06		ENSRNOG00000047367	10	108,440,950
22449	A_42_P539095	-0.719	0.063	1.8E-08	-0.733	0.059	6.4E-09	LOC688311	ENSRNOG00000049235	10	109,639,054
3389	A_64_P009335	-0.503	0.067	3.1E-06	-0.473	0.064	3.2E-06	LOC688318	ENSRNOG00000045926	10	109,893,700
22819	A_44_P159569	0.557	0.081	8.0E-06	0.609	0.077	1.5E-06	Wdr45l	ENSRNOG00000036662	10	110,555,629
5982	A_64_P071367	1.111	0.128	5.3E-07	1.123	0.121	2.3E-07	Krtap16-5	ENSRNOG00000040064	11	28,900,376
20993	A_64_P071362	0.985	0.094	5.1E-08	0.966	0.088	3.1E-08	Krtap16-5	ENSRNOG00000040064	11	28,900,376
6903	A_43_P16731	-0.471	0.081	4.3E-05	-0.439	0.076	4.9E-05	Ifngr2	ENSRNOG00000002032	11	31,694,339
9149	A_64_P162743	-0.884	0.119	3.3E-06	-0.856	0.112	2.4E-06	Dnajc28	ENSRNOG00000002026	11	31,772,984
12330	A_44_P121280	-1.648	0.102	1.9E-10	-0.854	0.096	3.9E-07	Kcne1	ENSRNOG00000001984	11	32,508,420
2645	A_44_P1039678	-0.670	0.064	5.9E-08	-0.543	0.061	3.7E-07	Setd4	ENSRNOG00000001699	11	33,801,999
14097	A_64_P064949	-0.612	0.066	2.5E-07	-0.445	0.062	5.1E-06		ENSRNOG00000051106	11	33,840,530
16374	A_64_P036352	-1.102	0.091	8.6E-09	-0.979	0.086	1.8E-08	Cbr1	ENSRNOG00000049911	11	33,863,500
4611	A_64_P043033	-1.259	0.136	2.5E-07	-1.016	0.128	1.6E-06	Cbr3	ENSRNOG00000001701	11	33,909,439
6829	A_43_P17424	-0.904	0.118	2.3E-06	-0.599	0.111	9.8E-05	Chaf1b	ENSRNOG00000001692	11	34,101,197

Disease Models & Mechanisms • Supplementary information

23291	A_44_P380384	-1.120	0.133	7.6E-07	-1.280	0.125	7.5E-08	Dscr6	ENSRNOG00000001684	11	34,557,679
29585	A_64_P104237	-0.360	0.063	5.8E-05	-0.474	0.060	1.6E-06	Ttc3	ENSRNOG00000001682	11	34,598,492
28300	A_44_P100023	0.323	0.054	3.1E-05	0.313	0.050	2.4E-05	Wrb	ENSRNOG00000001629	11	36,497,509
16198	A_43_P18730	1.147	0.092	6.3E-09	1.238	0.087	1.1E-09	LOC304000	ENSRNOG00000028216	11	36,736,586
15995	A_44_P215917	-1.036	0.152	8.4E-06	-0.888	0.143	2.3E-05	Plcxd2	ENSRNOG00000042289	11	57,207,656
6884	A_42_P732251	-1.590	0.105	4.5E-10	-1.661	0.099	1.1E-10	Phldb2	ENSRNOG00000002171	11	57,404,196
6750	A_64_P013875	-1.566	0.083	2.6E-11	-1.530	0.078	1.6E-11		ENSRNOG00000002171	11	57,430,166
3428	A_42_P840460	-0.679	0.086	1.6E-06	-0.668	0.081	9.6E-07	Ccdc52	ENSRNOG00000039024	11	61,287,914
10972	A_64_P118532	-0.802	0.103	1.9E-06	-0.529	0.097	8.5E-05	Casr	ENSRNOG00000002265	11	67,188,630
7015	A_64_P140781	0.788	0.135	4.2E-05	0.861	0.127	8.9E-06	Ostalpha	ENSRNOG00000001765	11	71,533,078
13604	A_44_P436604	0.504	0.070	4.6E-06	0.475	0.066	4.6E-06	Leprel1	ENSRNOG00000055751	11	78,029,038
5488	A_44_P379377	-0.723	0.113	1.7E-05	0.772	0.107	4.5E-06	Fetub	ENSRNOG00000001806	11	81,660,395
26833	A_43_P13230	0.679	0.077	4.2E-07	0.463	0.072	1.7E-05	Ehhadh	ENSRNOG00000001770	11	82,945,104
2087	A_64_P015758	-2.556	0.366	6.6E-06	-2.187	0.345	1.9E-05		ENSRNOG00000050000	11	86,092,468
16311	A_44_P139291	0.898	0.124	4.3E-06	0.990	0.117	7.1E-07	Prodh	ENSRNOG00000000281	11	87,058,616
16895	A_64_P038926	1.071	0.141	2.6E-06	0.943	0.133	5.6E-06	Prodh	ENSRNOG00000000281	11	87,058,616
7350	A_64_P048136	-1.131	0.102	2.6E-08	-0.724	0.096	2.8E-06	Brca2	ENSRNOG00000001111	12	504,007
2456	A_64_P245720	0.665	0.098	8.8E-06	0.531	0.092	5.1E-05	Zinki	ENSRNOG00000030410	12	5,490,935
3604	A_42_P746109	-0.613	0.069	3.8E-07	-0.564	0.065	5.1E-07	Asmtl	ENSRNOG00000028166	12	18,531,990
19145	A_64_P081501	1.197	0.068	6.9E-11	1.220	0.065	2.4E-11	Mcm7	ENSRNOG00000001349	12	19,314,016
7888	A_64_P156448	-0.660	0.092	5.0E-06	-0.800	0.087	2.7E-07	LOC685157	ENSRNOG00000050981	12	21,362,205
25485	A_64_P057626	-1.977	0.156	1.1E-08	-1.392	0.135	1.3E-07		ENSRNOG00000057201	12	22,380,027
28649	A_44_P975437	0.444	0.069	1.7E-05	0.739	0.065	2.1E-08	Cldn4	ENSRNOG00000001476	12	24,761,210
3110	A_64_P129805	0.493	0.069	5.1E-06	0.538	0.065	9.5E-07	Rfc2	ENSRNOG00000001457	12	25,143,480
7243	A_64_P113867	-0.688	0.117	4.0E-05	-0.706	0.110	1.7E-05	Caln1	ENSRNOG00000000886	12	29,743,705
17119	A_44_P654973	0.559	0.098	5.3E-05	0.596	0.092	1.5E-05	Rimbp2	ENSRNOG00000022893	12	31,530,699
9922	A_42_P818240	-0.474	0.063	2.8E-06	-0.328	0.059	7.5E-05	Ncor2	ENSRNOG00000001004	12	36,871,999
1906	A_64_P142962	1.063	0.136	1.8E-06	1.184	0.128	2.6E-07	Ccdc92	ENSRNOG00000021691	12	37,211,316
3175	A_44_P1030714	1.135	0.072	2.7E-10	1.341	0.068	1.3E-11	Ccdc92	ENSRNOG00000021691	12	37,211,316
13761	A_64_P135202	-0.779	0.105	3.2E-06	-0.657	0.099	1.1E-05	Snrnp35	ENSRNOG00000001060	12	37,538,403
5287	A_64_P115222	0.587	0.065	3.2E-07	0.487	0.061	1.4E-06	Pitpnm2	ENSRNOG00000029260	12	37,805,332
29139	A_44_P838992	-0.681	0.105	1.4E-05	-0.840	0.099	6.9E-07	~~LOC687609~~	ENSRNOG00000042607	12	38,880,377
16776	A_44_P226999	-1.002	0.180	9.2E-05	1.000	0.156	2.3E-05	Oas1i	ENSRNOG00000047076	12	41,155,497

22173	A_64_P025839	-1.043	0.155	1.0E-05	0.816	0.146	6.9E-05	Oas1k	ENSRNOG00000033220	12	41,307,352
2789	A_42_P612939	-1.178	0.152	2.1E-06	-1.155	0.144	1.3E-06	Tmem119	ENSRNOG00000000700	12	48,598,647
15771	A_64_P038532	-0.578	0.089	1.4E-05	-0.479	0.084	5.4E-05	Hps4	ENSRNOG00000000661	12	50,314,406
28389	A_44_P574134	-0.584	0.060	1.3E-07	-0.340	0.057	3.2E-05	Slc35f5	ENSRNOG00000003403	13	41,883,137
11088	A_42_P763058	1.498	0.123	8.1E-09	1.119	0.116	1.5E-07	Lypd1	ENSRNOG00000003453	13	42,263,024
14258	A_43_P13249	1.127	0.089	4.8E-09	1.025	0.084	7.7E-09	Acmsd	ENSRNOG00000003884	13	44,424,689
7157	A_64_P007633	0.712	0.109	1.3E-05	0.861	0.102	7.8E-07	C4bpb	ENSRNOG00000004125	13	47,397,890
22069	A_44_P764174	1.377	0.122	2.1E-08	0.881	0.115	2.3E-06	Faim3	ENSRNOG00000004441	13	47,602,692
585	A_42_P707818	-0.675	0.094	4.6E-06	-1.152	0.088	3.3E-09	Tmcc2	ENSRNOG00000000033	13	49,169,918
5286	A_44_P434271	1.155	0.192	3.3E-05	1.271	0.181	6.2E-06	Cntn2	ENSRNOG00000009033	13	49,313,940
19857	A_43_P15590	-0.432	0.071	2.9E-05	-0.396	0.067	3.8E-05	Fmod	ENSRNOG00000003183	13	50,873,605
16675	A_64_P055196	0.795	0.127	2.2E-05	0.731	0.120	2.8E-05	Camsap1l1	ENSRNOG00000008741	13	53,225,349
12995	A_44_P349273	1.732	0.129	2.2E-09	1.959	0.121	2.0E-10	F13b	ENSRNOG00000012613	13	56,598,957
2005	A_42_P819656	0.594	0.094	2.0E-05	0.501	0.089	6.3E-05	Cfh	ENSRNOG00000030715	13	57,080,549
7372	A_64_P001066	1.135	0.211	9.9E-05	1.107	0.199	7.1E-05	RGD1561738	ENSRNOG00000042995	13	73,661,131
1416	A_64_P091088	1.064	0.096	2.7E-08	1.322	0.091	7.5E-10	Fmo2	ENSRNOG00000003510	13	80,775,264
20699	A_44_P486312	0.955	0.102	2.2E-07	1.021	0.096	4.7E-08	Fmo2	ENSRNOG00000003510	13	80,775,264
14514	A_44_P180259	1.853	0.115	2.1E-10	1.680	0.108	3.5E-10	Dpt	ENSRNOG00000002947	13	83,073,550
22348	A_44_P1018997	-0.425	0.064	1.2E-05	-0.527	0.061	5.3E-07	Tmco1	ENSRNOG00000003928	13	85,465,792
12064	A_64_P027625	-4.908	0.087	7.6E-18	-0.575	0.082	6.6E-06	Rxrg	ENSRNOG00000004537	13	85,818,427
21779	A_44_P306439	0.950	0.104	2.9E-07	1.759	0.098	4.9E-11	Fcgr3a	ENSRNOG00000024382	13	89,385,859
3217	A_42_P735417	1.129	0.189	3.5E-05	0.974	0.178	8.4E-05	Fcgr2b	ENSRNOG00000046452	13	89,433,815
25592	A_44_P575255	2.333	0.113	7.0E-12	2.309	0.106	3.6E-12	RGD1562658	ENSRNOG00000034221	13	89,480,058
3268	A_64_P005029	-0.915	0.087	4.9E-08	-0.742	0.082	3.1E-07	Pcp4l1	ENSRNOG00000003209	13	89,565,813
23319	A_64_P072455	-1.892	0.112	1.2E-08	-2.076	0.128	1.8E-08	LOC689766	ENSRNOG00000021411	13	95,887,708
9059	A_42_P803590	-1.853	0.112	1.5E-10	-1.660	0.106	2.9E-10	LOC289378	ENSRNOG00000046460	13	109,663,364
3954	A_42_P661065	0.953	0.094	8.0E-08	1.120	0.089	5.0E-09	Ints7	ENSRNOG00000004263	13	110,257,571
10206	A_64_P052464	1.761	0.117	5.3E-10	1.897	0.111	9.1E-11	Ints7	ENSRNOG00000004263	13	110,257,571
4910	A_43_P12200	0.424	0.068	2.3E-05	0.448	0.064	7.0E-06	Slc26a1	ENSRNOG00000000041	14	2,050,483
23982	A_64_P015340	1.414	0.180	7.9E-06	1.565	0.174	2.2E-06	Mfsd7	ENSRNOG00000023937	14	2,311,122
29630	A_64_P015335	2.104	0.127	1.4E-10	2.094	0.120	6.9E-11	Mfsd7	ENSRNOG00000023937	14	2,311,122
3061	A_44_P454646	0.641	0.108	3.6E-05	0.621	0.102	2.7E-05	Fam69a	ENSRNOG00000023533	14	2,789,650
27220	A_64_P165953	-1.355	0.180	2.9E-06	-1.292	0.170	2.5E-06	Znf644	ENSRNOG00000002112	14	4,125,380

5642	A_42_P715210	1.079	0.092	1.3E-08	0.933	0.087	4.0E-08	Abcg3l3	ENSRNOG00000039890	14	6,058,544
21816	A_64_P085715	0.539	0.071	2.6E-06	0.551	0.067	1.0E-06	Pkd2	ENSRNOG00000002146	14	6,645,257
10617	A_44_P1043814	-0.495	0.061	1.2E-06	-0.463	0.058	1.3E-06	Hsd17b11	ENSRNOG00000002210	14	7,073,445
15964	A_64_P041270	0.906	0.146	2.3E-05	0.980	0.137	5.1E-06	Fras1	ENSRNOG00000002053	14	14,689,554
23631	A_64_P013437	1.886	0.246	2.3E-06	2.144	0.232	2.5E-07	Cxcl13	ENSRNOG00000024899	14	15,258,207
783	A_64_P071159	-4.520	0.278	1.8E-10	-3.917	0.262	5.6E-10	LOC360919	ENSRNOG00000039504	14	19,072,677
17181	A_64_P071158	-4.519	0.278	1.8E-10	-3.829	0.262	7.4E-10	LOC360919	ENSRNOG00000039504	14	19,072,677
16082	A_44_P353446	-3.330	0.357	2.2E-07	-2.572	0.336	2.3E-06	Afm	ENSRNOG00000002878	14	19,132,208
7897	A_43_P13995	1.851	0.236	1.7E-06	1.899	0.222	6.5E-07	Gc	ENSRNOG00000003119	14	20,266,891
8034	A_43_P12233	0.748	0.068	2.7E-08	0.837	0.064	3.1E-09	Sult1b1	ENSRNOG00000001967	14	22,142,364
27643	A_64_P053257	0.811	0.073	2.4E-08	0.926	0.069	2.1E-09	Sult1b1	ENSRNOG00000001967	14	22,142,364
4292	A_64_P119836	-1.059	0.176	3.3E-05	-1.102	0.166	1.2E-05	Shisa3	ENSRNOG00000025670	14	42,221,225
29204	A_44_P123033	-0.428	0.076	6.3E-05	-0.487	0.072	8.8E-06	Klhl5	ENSRNOG00000008421	14	44,845,218
28582	A_43_P11601	-1.251	0.081	3.6E-10	-1.060	0.076	1.5E-09	Sod3	ENSRNOG00000003869	14	60,964,324
22	A_44_P992306	-1.037	0.092	2.0E-08	-1.038	0.086	9.4E-09	Gnpda2	ENSRNOG00000002177	14	62,646,110
5318	A_42_P674050	-1.136	0.132	6.0E-07	-0.940	0.125	2.7E-06	Sorcs2	ENSRNOG00000007033	14	79,538,911
11690	A_44_P330188	2.796	0.173	1.9E-10	2.826	0.163	7.6E-11	Acox2	ENSRNOG00000007378	15	18,451,144
17115	A_64_P031249	0.909	0.081	2.2E-08	0.911	0.076	1.0E-08	Gnpnat1	ENSRNOG00000008641	15	19,733,967
12606	A_64_P098006	1.071	0.135	1.5E-06	0.807	0.127	1.8E-05	Fitm1	ENSRNOG00000019019	15	34,251,606
510	A_64_P068192	-0.834	0.120	6.7E-06	-0.923	0.113	1.1E-06	Mcpt8_rat	ENSRNOG00000049991	15	34,694,244
10995	A_64_P124294	-1.372	0.171	1.3E-06	-1.167	0.161	4.4E-06	Mcpt9_rat	ENSRNOG00000031792	15	35,002,406
23836	A_64_P151851	-0.742	0.122	3.0E-05	-0.642	0.115	7.1E-05	Phf11	ENSRNOG00000053891	15	38,709,984
22768	A_64_P001612	-0.373	0.062	3.2E-05	-0.411	0.059	6.2E-06	Trim35	ENSRNOG00000009449	15	42,960,307
1315	A_64_P134160	-2.430	0.197	6.7E-09	-2.272	0.185	7.5E-09	Defb42	ENSRNOG00000038762	15	46,166,335
3047	A_44_P105225	-0.765	0.138	7.3E-05	-0.792	0.130	2.9E-05	Loxl2	ENSRNOG00000051538	15	51,364,261
29985	A_44_P520441	-2.843	0.121	7.0E-08	-2.499	0.121	1.7E-07	Pcdh9	ENSRNOG00000038068	15	77,736,892
27981	A_64_P150884	-0.773	0.118	1.9E-05	-0.838	0.118	8.3E-06	Scel	ENSRNOG00000024237	15	87,704,340
1825	A_42_P787239	0.673	0.064	5.6E-08	0.619	0.061	7.6E-08	Farp1	ENSRNOG00000011203	15	106,434,177
6846	A_64_P005043	0.449	0.081	6.9E-05	0.693	0.076	2.9E-07	Nt5dc2	ENSRNOG00000018358	16	7,212,488
30347	A_64_P066923	0.975	0.117	8.5E-07	1.230	0.110	2.4E-08	Ncoa4	ENSRNOG00000019768	16	8,302,950
25096	A_64_P142026	1.136	0.138	1.0E-06	1.105	0.130	7.0E-07	Ppyr1	ENSRNOG00000061026	16	10,490,706
27813	A_44_P228059	0.628	0.100	2.0E-05	0.676	0.094	4.8E-06	Mbl1	ENSRNOG00000011706	16	18,736,154
1186	A_42_P467381	0.660	0.076	5.2E-07	0.565	0.072	1.6E-06	Fam32a	ENSRNOG00000039528	16	19,308,842

6443	A_44_P196779	-0.604	0.085	5.1E-06	-0.465	0.080	4.4E-05	Haus8	ENSRNOG00000052038	16	19,669,196
21276	A_64_P051636	-0.958	0.107	3.5E-07	-1.253	0.100	5.9E-09	Pde4c	ENSRNOG00000019518	16	20,460,959
19242	A_64_P119675	-0.572	0.079	4.5E-06	-0.481	0.075	1.6E-05	Jund	ENSRNOG00000019568	16	20,486,707
6546	A_64_P124701	-1.558	0.176	4.2E-07	-1.921	0.166	1.5E-08	Crlf1	ENSRNOG00000020030	16	20,686,317
17539	A_44_P463822	-0.735	0.082	3.7E-07	-0.727	0.078	2.1E-07	Homer3	ENSRNOG00000020229	16	20,888,907
8703	A_64_P085936	-2.563	0.121	5.3E-12	-2.769	0.114	8.2E-13	Mef2b	ENSRNOG00000020400	16	21,017,163
23904	A_64_P100904	-0.920	0.126	4.0E-06	-0.717	0.119	3.1E-05	LOC688966	ENSRNOG00000020427	16	21,029,134
25971	A_64_P004531	-0.950	0.075	4.5E-09	-0.741	0.070	5.1E-08	LOC688966	ENSRNOG00000020427	16	21,029,134
9569	A_64_P087014	1.039	0.128	1.2E-06	0.711	0.121	4.0E-05	Slc18a1	ENSRNOG00000011992	16	22,361,998
4835	A_44_P1016480	2.037	0.163	5.8E-09	1.746	0.154	2.0E-08	Lpl	ENSRNOG00000012181	16	22,561,496
8763	A_64_P144978	0.703	0.082	6.3E-07	0.505	0.077	1.4E-05	Csgalnact1	ENSRNOG00000013024	16	22,979,444
15146	A_44_P1036068	-0.509	0.060	7.5E-07	-0.494	0.057	5.3E-07	RGD1311747	ENSRNOG00000011293	16	32,540,217
3982	A_64_P007462	0.863	0.098	4.5E-07	1.068	0.092	1.5E-08	Spcs3	ENSRNOG00000038933	16	40,050,734
26075	A_64_P041016	-0.997	0.081	6.8E-09	-1.073	0.076	1.2E-09	Wwc2	ENSRNOG00000013248	16	47,368,768
9554	A_64_P048315	0.916	0.085	3.7E-08	0.893	0.080	2.4E-08		ENSRNOG00000055088	16	59,517,000
28774	A_44_P438492	0.426	0.067	1.8E-05	0.540	0.063	6.3E-07	Mak16	ENSRNOG00000010783	16	64,729,221
11412	A_44_P1042163	0.796	0.061	3.1E-09	0.983	0.057	9.0E-11	RGD1310414	ENSRNOG00000023494	16	64,745,207
18101	A_44_P941895	0.442	0.078	5.6E-05	0.403	0.073	7.8E-05	Prosc	ENSRNOG00000013751	16	69,176,036
7165	A_64_P087109	-0.398	0.073	8.8E-05	-0.440	0.069	1.7E-05	Ankrd10	ENSRNOG00000013618	16	83,206,004
6176	A_64_P150047	-0.457	0.060	2.3E-06	-0.369	0.056	1.3E-05	Rab20	ENSRNOG00000023991	16	83,358,116
3375	A_44_P309325	0.793	0.123	1.5E-05	0.650	0.116	6.5E-05	Lig4	ENSRNOG00000014605	16	85,331,866
18894	A_64_P022751	0.441	0.061	4.0E-06	0.484	0.057	7.1E-07	Isca1	ENSRNOG00000018343	17	5,281,727
11386	A_64_P014278	-1.584	0.057	1.4E-13	-1.410	0.054	3.2E-13	LOC680692	ENSRNOG00000018400	17	5,382,034
30268	A_44_P215479	0.523	0.071	3.9E-06	0.382	0.067	5.8E-05	Rmi1	ENSRNOG00000019108	17	6,660,536
24746	A_64_P163822	-1.220	0.083	6.9E-10	-1.123	0.078	9.5E-10	Cks2	ENSRNOG00000014130	17	13,593,423
16526	A_44_P260348	0.851	0.118	4.5E-06	0.674	0.111	2.9E-05	LOC689316	ENSRNOG00000058611	17	15,108,456
4180	A_64_P165035	6.620	0.112	3.9E-18	5.843	0.106	9.7E-18	RGD1305679	ENSRNOG00000014481	17	22,619,891
8900	A_64_P059262	0.995	0.087	1.8E-08	1.029	0.082	5.5E-09	Riok1	ENSRNOG00000014049	17	27,451,832
5827	A_64_P055102	0.450	0.052	5.9E-07	0.340	0.049	7.5E-06	Eci3	ENSRNOG00000029549	17	30,617,382
21031	A_64_P067678	-0.615	0.111	7.4E-05	-0.624	0.105	3.6E-05	Aldh5a1	ENSRNOG00000023538	17	42,133,076
23925	A_64_P030919	-0.805	0.112	4.6E-06	-0.784	0.105	3.2E-06		ENSRNOG00000023538	17	42,133,076
4429	A_64_P127026	-0.506	0.085	3.6E-05	-0.551	0.080	7.9E-06	Cmah	ENSRNOG00000003094	17	42,640,221
7060	A_42_P555365	0.751	0.083	3.1E-07	0.672	0.078	5.8E-07	Slc17a3	ENSRNOG00000032745	17	43,537,293

14149	A_64_P080667	-0.766	0.125	2.6E-05	-0.743	0.117	1.9E-05	LOC684841	ENSRNOG00000045644	17	43,807,540
18077	A_64_P127848	-1.330	0.151	8.1E-07	-1.420	0.131	7.3E-08	Aoah	ENSRNOG00000054964	17	46,115,004
1977	A_64_P100530	-0.420	0.078	9.7E-05	-0.435	0.074	3.8E-05	Arhgap12	ENSRNOG00000017791	17	54,280,851
17105	A_64_P040038	2.256	0.209	3.6E-08	2.471	0.197	5.3E-09	RGD1563564	ENSRNOG00000016217	17	63,145,577
13903	A_44_P515798	2.632	0.099	2.4E-13	2.608	0.093	1.2E-13	Ucma	ENSRNOG00000017987	17	77,261,731
14129	A_44_P491929	-0.477	0.084	5.9E-05	-0.438	0.079	7.7E-05	Prpf18	ENSRNOG00000018396	17	77,601,914
20226	A_64_P104454	-0.723	0.099	4.1E-06	-0.541	0.094	4.9E-05	Impad1	ENSRNOG00000046647	17	90,218,013
25656	A_44_P245616	-1.011	0.098	6.3E-08	-0.531	0.092	5.0E-05	Nid1	ENSRNOG00000002461	17	90,627,101
20714	A_64_P056712	0.852	0.067	4.8E-09	0.767	0.063	8.6E-09	Gpr137b	ENSRNOG00000002480	17	90,696,019
14497	A_64_P174430	-0.477	0.085	6.4E-05	-0.596	0.080	3.2E-06	Cdig2	ENSRNOG00000012016	18	3,662,683
30354	A_64_P089742	-0.486	0.068	4.7E-06	-0.545	0.064	6.3E-07	Cdig2	ENSRNOG00000012016	18	3,662,683
25080	A_64_P029377	1.155	0.157	3.7E-06	1.179	0.148	1.5E-06	Hrh4	ENSRNOG00000016887	18	4,365,429
16785	A_44_P1028952	-0.596	0.079	2.6E-06	-0.488	0.074	1.2E-05		ENSRNOG00000016467	18	6,474,990
13848	A_43_P19054	1.007	0.130	9.4E-06	1.438	0.126	2.0E-07	Dsc2	ENSRNOG00000039969	18	11,858,744
7111	A_44_P241308	-0.755	0.116	1.4E-05	-0.861	0.109	1.7E-06	LOC100360334	ENSRNOG00000027230	18	16,650,806
28984	A_64_P034128	-1.109	0.156	5.3E-06	-0.797	0.147	9.1E-05	Nrep	ENSRNOG00000020467	18	26,211,445
2039	A_64_P135224	2.166	0.146	6.0E-10	3.101	0.137	2.2E-12	RGD1309362	ENSRNOG00000038960	18	55,505,993
4992	A_44_P549494	0.833	0.086	1.4E-07	1.090	0.081	2.1E-09	Cd74	ENSRNOG00000018735	18	56,071,478
14590	A_64_P160393	-0.454	0.080	6.1E-05	-0.498	0.076	1.3E-05	Tcf4	ENSRNOG00000012405	18	65,285,318
17166	A_44_P419615	-1.487	0.139	1.8E-07	-1.251	0.105	5.4E-08	Stard6	ENSRNOG00000026324	18	68,983,545
7335	A_42_P595630	-1.153	0.070	1.6E-10	-1.060	0.066	2.3E-10	Pqlc1	ENSRNOG00000059215	18	76,770,012
22519	A_64_P021320	-1.114	0.065	9.3E-11	-1.077	0.061	6.7E-11	Pqlc1	ENSRNOG00000059215	18	76,770,012
30080	A_64_P110941	-2.155	0.153	1.2E-09	-1.316	0.144	3.0E-07	Cndp1	ENSRNOG00000027739	18	81,499,051
17183	A_44_P116591	-0.433	0.075	4.8E-05	-0.397	0.071	6.4E-05	Cx3cl1	ENSRNOG00000016326	19	10,653,800
5572	A_64_P123603	0.672	0.111	3.1E-05	0.866	0.105	9.6E-07	Mt1a	ENSRNOG00000025764	19	11,302,938
14056	A_64_P162764	0.828	0.148	6.6E-05	1.117	0.139	1.4E-06	Mt1a_partial	ENSRNOG00000025764	19	11,302,938
2726	A_44_P996729	-0.888	0.104	6.9E-07	-0.887	0.099	3.5E-07	Mmp2	ENSRNOG00000016695	19	15,570,611
6691	A_44_P100716	-1.037	0.126	9.9E-07	-0.763	0.119	1.6E-05	Mylk3	ENSRNOG00000017546	19	27,404,712
13133	A_44_P133335	-1.169	0.150	4.9E-06	-0.856	0.113	6.8E-06	NA	ENSRNOG00000049468	19	28,645,488
3248	A_64_P000604	-0.633	0.088	4.6E-06	-0.461	0.083	7.0E-05	Tradd	ENSRNOG00000015179	19	37,216,572
7827	A_64_P017565	-0.787	0.084	2.3E-07	-0.614	0.080	2.1E-06	Cdh3	ENSRNOG00000020129	19	38,669,230
7237	A_64_P052690	-0.401	0.055	4.2E-06	-0.452	0.052	5.4E-07	PDF*	ENSRNOG00000022303	19	39,246,545
18072	A_44_P184497	0.462	0.085	8.5E-05	0.460	0.080	5.0E-05	Cyb5b	ENSRNOG00000011142	19	39,357,814

19705	A_44_P220325	-1.242	0.142	1.5E-06	-1.237	0.107	7.6E-08	Dynlrb2	ENSRNOG00000012450	19	49,016,891
10809	A_44_P374638	-0.905	0.163	7.3E-05	-1.022	0.154	1.1E-05	Hsd17b2	ENSRNOG00000013982	19	50,246,402
18230	A_44_P340193	-0.568	0.085	1.0E-05	-0.469	0.080	4.0E-05	Hsdl1	ENSRNOG00000015576	19	52,217,728
6490	A_42_P620006	-1.374	0.164	7.9E-07	-0.862	0.154	6.8E-05	Wfdc1	ENSRNOG00000015904	19	52,313,795
28178	A_64_P103254	-0.560	0.074	2.8E-06	-0.437	0.070	2.2E-05	Klhl36	ENSRNOG00000016422	19	52,515,022
4436	A_42_P627572	-1.457	0.066	2.9E-12	-1.519	0.062	7.3E-13	Mdc1	ENSRNOG00000032813	20	3,405,285
7136	A_64_P062110	0.673	0.110	2.8E-05	0.664	0.104	1.7E-05	RT1-DMb	ENSRNOG00000049491	20	3,945,601
1417	A_64_P043911	0.741	0.135	8.1E-05	1.099	0.127	5.8E-07	RT1-Bb	ENSRNOG00000032708	20	4,039,413
18363	A_64_P134927	0.523	0.089	3.8E-05	0.873	0.083	5.5E-08	RT1-Db1	ENSRNOG00000033215	20	4,087,618
12415	A_64_P128478	1.163	0.115	8.0E-08	1.031	0.108	1.7E-07	RT1-DMa	ENSRNOG00000047864	20	5,241,496
8347	A_44_P367438	0.462	0.059	1.8E-06	0.415	0.056	3.1E-06	Stk38	ENSRNOG00000000519	20	6,257,604
18720	A_64_P150618	-0.846	0.127	1.1E-05	-0.675	0.120	6.4E-05	LOC689755	ENSRNOG00000000531	20	8,384,088
15230	A_64_P048033	0.861	0.098	4.5E-07	0.784	0.092	6.8E-07	Agpat3	ENSRNOG00000001205	20	11,114,164
25935	A_64_P146698	0.750	0.061	7.5E-09	0.797	0.058	1.6E-09	Pwp2	ENSRNOG00000001210	20	11,228,844
24999	A_44_P840891	-2.028	0.107	2.3E-11	-1.846	0.101	3.6E-11	Icoslg	ENSRNOG00000023109	20	11,340,296
7074	A_64_P001461	-1.312	0.144	5.5E-07	-0.803	0.125	2.3E-05	Dnmt3l	ENSRNOG00000001212	20	11,359,090
19033	A_44_P1015203	0.554	0.086	1.6E-05	0.811	0.081	1.0E-07	Gstt3	ENSRNOG00000001242	20	13,817,795
23216	A_64_P042607	-1.418	0.178	1.5E-06	-1.135	0.168	9.5E-06	Ctnna3	ENSRNOG00000000378	20	26,589,209
405	A_42_P559737	1.012	0.105	1.5E-07	0.944	0.099	1.7E-07	Srgn	ENSRNOG00000000394	20	32,133,431
220	A_42_P796980	-2.674	0.252	4.7E-08	-2.596	0.238	3.2E-08	Ros1	ENSRNOG00000000406	20	33,323,367
27233	A_44_P942367	-1.581	0.165	1.6E-07	-1.596	0.155	6.8E-08	Tspyl4	ENSRNOG00000000547	20	41,100,071
26015	A_64_P147443	-2.080	0.216	1.5E-07	-1.631	0.204	1.4E-06	LOC679824	ENSRNOG00000051272	20	44,606,610
29075	A_64_P020516	-0.342	0.053	1.7E-05	-0.319	0.050	1.9E-05	Rev3l	ENSRNOG00000000593	20	44,803,666
16018	A_44_P1035656	-0.592	0.086	7.4E-06	-0.552	0.081	8.4E-06	Cd24	ENSRNOG00000000321	20	48,335,540
18623	A_64_P042519	-1.154	0.132	4.8E-07	-1.124	0.124	3.2E-07	Grik2	ENSRNOG00000000368	20	54,517,709
11936	A_44_P547801	-0.872	0.058	5.8E-10	-0.626	0.055	2.0E-08	Gstt2	ENSRNOG00000052360	L568423	501
3314	A_64_P149779	0.763	0.113	1.3E-05	0.742	0.107	1.0E-05		ENSRNOG00000031780	MT	1
4051	A_64_P011635	-0.685	0.086	1.5E-06	-0.487	0.081	3.3E-05		ENSRNOG00000031780	MT	1
5091	A_44_P342334	-0.759	0.071	4.3E-08	-0.768	0.067	1.8E-08		ENSRNOG00000031780	MT	1
5482	A_64_P326359	1.069	0.063	1.1E-10	1.286	0.060	4.0E-12		ENSRNOG00000031780	MT	1
6741	A_64_P123502	-0.933	0.111	8.0E-07	-0.586	0.105	6.8E-05		ENSRNOG00000031780	MT	1
7686	A_64_P011389	0.836	0.145	5.1E-05	1.009	0.137	3.6E-06		ENSRNOG00000031780	MT	1
7793	A_64_P052878	-0.647	0.107	2.9E-05	-0.693	0.101	7.6E-06		ENSRNOG00000031780	MT	1

Disease Models & Mechanisms • Supplementary information

8774	A_64_P164751	1.238	0.160	3.3E-06	1.123	0.152	5.3E-06	ENSRNOG00000031780	MT	1
9302	A_64_P010221	1.467	0.173	6.9E-07	1.061	0.163	1.4E-05	ENSRNOG00000031780	MT	1
9336	A_64_P063588	0.760	0.072	4.9E-08	0.765	0.068	2.2E-08	ENSRNOG00000031780	MT	1
9394	A_64_P119214	1.075	0.063	1.0E-10	0.639	0.060	4.0E-08	ENSRNOG00000031780	MT	1
9500	A_64_P159175	-0.847	0.119	5.3E-06	-0.730	0.112	1.4E-05	ENSRNOG00000031780	MT	1
10091	A_64_P150008	-1.022	0.119	5.9E-07	-1.172	0.112	5.4E-08	ENSRNOG00000031780	MT	1
10168	A_64_P014010	2.047	0.181	2.0E-08	2.186	0.170	4.1E-09	ENSRNOG00000031780	MT	1
10192	A_64_P060434	1.086	0.108	8.6E-08	0.643	0.101	1.9E-05	ENSRNOG00000031780	MT	1
10320	A_64_P096929	-1.188	0.092	3.6E-09	-0.956	0.086	2.7E-08	ENSRNOG00000031780	MT	1
10544	A_64_P085915	-1.074	0.129	8.7E-07	-0.926	0.121	2.4E-06	ENSRNOG00000031780	MT	1
11592	A_64_P152094	0.930	0.149	2.3E-05	0.801	0.141	5.7E-05	ENSRNOG00000031780	MT	1
11641	A_44_P899149	0.889	0.117	2.4E-06	0.760	0.110	7.3E-06	ENSRNOG00000031780	MT	1
11809	A_64_P024675	-2.523	0.167	1.3E-09	-2.405	0.145	4.0E-10	ENSRNOG00000031780	MT	1
12260	A_64_P147014	0.544	0.076	4.7E-06	0.406	0.071	5.6E-05	ENSRNOG00000031780	MT	1
12390	A_64_P143186	-1.046	0.167	2.1E-05	-0.917	0.158	4.5E-05	ENSRNOG00000031780	MT	1
14345	A_64_P020880	-0.644	0.089	4.3E-06	-0.752	0.084	3.5E-07	ENSRNOG00000031780	MT	1
14912	A_64_P097468	1.233	0.099	5.6E-09	0.806	0.093	5.4E-07	ENSRNOG00000031780	MT	1
15455	A_64_P039840	0.695	0.125	7.3E-05	0.642	0.118	8.9E-05	ENSRNOG00000031780	MT	1
15612	A_64_P010052	-1.703	0.121	8.4E-09	-1.859	0.111	1.2E-09	ENSRNOG00000031780	MT	1
16475	A_64_P071381	1.018	0.129	1.7E-06	1.002	0.122	1.0E-06	ENSRNOG00000031780	MT	1
16858	A_64_P146089	-1.963	0.099	1.3E-11	-1.388	0.094	6.2E-10	ENSRNOG00000031780	MT	1
19343	A_64_P070068	1.661	0.277	3.3E-05	1.465	0.261	6.5E-05	ENSRNOG00000031780	MT	1
19961	A_64_P117198	-0.698	0.119	4.2E-05	-0.622	0.112	7.5E-05	ENSRNOG00000031780	MT	1
20674	A_64_P078577	0.682	0.108	2.0E-05	0.633	0.102	2.3E-05	ENSRNOG00000031780	MT	1
21135	A_64_P118308	-1.437	0.086	1.3E-10	-1.512	0.081	3.0E-11	ENSRNOG00000031780	MT	1
21748	A_64_P071088	0.834	0.100	8.9E-07	1.043	0.094	2.8E-08	ENSRNOG00000031780	MT	1
21806	A_64_P094555	0.993	0.114	5.0E-07	0.608	0.107	5.9E-05	ENSRNOG00000031780	MT	1
22371	A_64_P039036	-0.557	0.061	2.8E-07	-0.522	0.057	3.0E-07	ENSRNOG00000031780	MT	1
22436	A_64_P045775	1.063	0.083	4.2E-09	1.033	0.078	2.9E-09	ENSRNOG00000031780	MT	1
22975	A_64_P162760	1.002	0.164	2.8E-05	1.266	0.155	1.1E-06	ENSRNOG00000031780	MT	1
24098	A_64_P042811	-2.176	0.100	3.8E-12	-2.155	0.095	1.9E-12	ENSRNOG00000031780	MT	1
24429	A_64_P086550	-1.365	0.143	1.7E-07	-1.330	0.135	1.1E-07	ENSRNOG00000031780	MT	1
27262	A_64_P143003	1.008	0.110	2.9E-07	0.664	0.104	1.7E-05	ENSRNOG00000031780	MT	1

27514	A_64_P037848	0.797	0.145	7.8E-05	0.779	0.136	5.4E-05		ENSRNOG00000031780	MT	1
27679	A_64_P143154	0.541	0.085	1.8E-05	0.767	0.080	1.6E-07		ENSRNOG00000031780	MT	1
29480	A_64_P134485	1.107	0.124	3.7E-07	0.667	0.117	5.3E-05		ENSRNOG00000031780	MT	1
30061	A_64_P086167	2.970	0.298	1.0E-07	3.074	0.281	3.2E-08		ENSRNOG00000031780	MT	1
30345	A_64_P139614	-0.818	0.122	1.1E-05	-0.827	0.115	4.9E-06		ENSRNOG00000031780	MT	1
18904	A_64_P115786	2.417	0.200	9.0E-09	2.588	0.189	1.7E-09		ENSRNOG00000009609	X	1,615,920
28608	A_43_P11444	0.529	0.091	4.4E-05	0.595	0.085	6.8E-06	S100g	ENSRNOG00000004222	X	33,443,186
10435	A_64_P010338	0.718	0.072	1.0E-07	0.494	0.068	4.2E-06		ENSRNOG00000051268	X	62,977,719
28414	A_64_P085732	0.726	0.095	2.3E-06	0.640	0.089	4.8E-06	Arhgef9	ENSRNOG00000007733	X	64,428,444
11028	A_64_P072533	1.103	0.100	2.7E-08	1.047	0.094	2.5E-08		ENSRNOG00000011100	X	115,426,083
15663	A_64_P048852	-1.660	0.080	7.2E-12	-1.766	0.076	1.4E-12		ENSRNOG00000006459	X	120,313,696
20264	A_44_P478319	0.836	0.127	1.2E-05	0.750	0.120	2.1E-05	Lonrf3	ENSRNOG00000013092	X	122,938,009
3602	A_64_P060874	-0.979	0.085	1.8E-08	-0.781	0.081	1.4E-07	Gpc3	ENSRNOG00000060179	X	139,916,883
245	A_64_P024665	0.895	0.162	7.4E-05	1.304	0.152	6.3E-07	LOC688090			
415	A_42_P803752	-1.044	0.176	3.7E-05	-1.157	0.166	6.6E-06	RGD1562146			
4900	A_44_P668185	-0.471	0.068	7.3E-06	-0.421	0.064	1.3E-05	RGD1310444			
5530	A_42_P538334	0.433	0.061	5.5E-06	0.392	0.058	8.7E-06	Snhg8			
7682	A_64_P077935	0.936	0.147	1.7E-05	1.212	0.138	4.7E-07	LOC690776			
7826	A_64_P024117	-1.376	0.119	8.5E-06	-1.034	0.119	5.5E-05	LOC497952			
7899	A_64_P006498	-1.666	0.166	9.4E-08	-1.449	0.157	2.5E-07	Ces1e_rat			
8819	A_64_P132726	-1.552	0.130	1.0E-08	-1.405	0.122	1.7E-08	no transcript			
10176	A_43_P15246	1.156	0.150	2.2E-06	0.891	0.142	2.0E-05	**Pthlh**	N/A	4	181,663,425
10999	A_64_P070586	0.874	0.105	8.8E-07	0.907	0.099	2.8E-07	Krtap21-2			
12619	A_64_P060319	-0.658	0.102	1.5E-05	-0.582	0.096	2.9E-05	Gsg1l			
12669	A_64_P115157	-1.112	0.116	1.7E-07	-1.076	0.110	1.2E-07	Ces1e_rat			
15289	A_44_P393282	-0.406	0.056	4.0E-06	-0.300	0.052	5.3E-05	no transcript			
15442	A_64_P155603	-1.327	0.148	3.7E-07	-1.007	0.140	4.6E-06	Mcpt8_RGD1565970			
16545	A_44_P369612	-0.537	0.095	5.7E-05	-0.617	0.089	7.2E-06	LOC305633			
16795	A_64_P009823	0.924	0.132	6.4E-06	0.674	0.125	9.3E-05	Slc39a4l			
17474	A_44_P201084	-1.297	0.156	9.0E-07	-1.286	0.147	4.9E-07	Fgfr1l			
18051	A_43_P11739	-0.779	0.055	1.3E-09	-0.546	0.052	5.7E-08	**Ctsl1**	ENSRNOG00000018566	17	1,873,105
18349	A_64_P157474	-0.544	0.077	6.0E-06	-0.701	0.073	1.6E-07	LOC500956			
22943	A_64_P037000	3.271	0.152	6.1E-11	3.475	0.145	1.8E-11	no transcript			

23854	A_64_P021149	-1.449	0.114	2.6E-08	-1.684	0.105	1.8E-09	LOC686141			
23957	A_64_P139208	-1.817	0.118	3.8E-10	-1.585	0.111	1.1E-09	RT1-N1 (part)			
24636	A_42_P835445	-2.341	0.228	7.0E-08	-2.094	0.215	1.3E-07	no transcript			
25709	A_64_P011719	-1.134	0.082	1.5E-09	-0.816	0.077	4.7E-08	**Chmp1b**	N/A	18	62,923,620
26309	A_44_P960266	-0.831	0.085	1.2E-07	-0.706	0.080	4.2E-07	RGD1307493			
30047	A_64_P096096	-0.736	0.111	1.1E-05	-0.572	0.104	8.2E-05	RGD1562551			

Genes are regarded as significant for “shared” differential expression when they show $P<1\times10^{-4}$ reproducibly in two types of comparison, ie, SHR/Izm versus WKY/Izm and SHRSP/Izm versus WKY/Izm, with a concordant direction of differential expression.

Disease Models & Mechanisms • Supplementary information

Table S3E. A list of significant transcripts for shared differential expression in the liver.

no	ProbeName	SHR/lzm (vs WKY/lzm)			SHRSP/lzm (vs WKY/lzm)			GeneName	Ensembl_rat	Chr	Transcription_ start_site (Rnor_6.0)
		Fold change (log2-scale)	SE (log2-scale)	P-value	Fold change (log2-scale)	SE (log2-scale)	P-value				
12215	A_44_P973511	-1.097	0.129	1.4E-06	-1.368	0.129	1.2E-07	Lrp11	ENSRNOG00000014303	1	1,702,696
24903	A_64_P062353	-0.677	0.109	3.5E-05	-0.930	0.109	1.3E-06	Lrp11	ENSRNOG00000014303	1	1,702,696
11055	A_44_P128306	0.816	0.144	8.5E-05	0.890	0.144	3.7E-05	Aldh8a1	ENSRNOG00000014907	1	16,910,069
26496	A_44_P182431	-2.251	0.260	1.1E-06	-2.882	0.260	6.9E-08	Srd5a1	ENSRNOG00000017601	1	36,320,461
27411	A_44_P519871	-2.153	0.271	2.9E-06	-2.567	0.271	4.2E-07	Srd5a1	ENSRNOG00000017601	1	36,320,461
14176	A_64_P164736	1.403	0.240	6.4E-05	1.921	0.240	2.7E-06	LOC100359930	ENSRNOG00000028852	1	82,653,633
22179	A_64_P129593	-1.566	0.228	1.3E-05	-1.594	0.228	1.1E-05	Atp4a	ENSRNOG00000020985	1	89,162,639
7527	A_64_P151448	-0.957	0.151	2.9E-05	-0.908	0.151	4.9E-05	Fancf	ENSRNOG00000023968	1	107,232,305
19183	A_64_P133177	-0.724	0.123	5.9E-05	-1.006	0.123	2.1E-06	Sema4b	ENSRNOG00000025167	1	141,986,145
27674	A_64_P000452	-0.741	0.110	1.6E-05	-0.657	0.110	5.4E-05	Cyp2c7	ENSRNOG00000059330	1	147,435,897
6483	A_44_P154040	2.085	0.146	3.4E-09	1.545	0.146	1.1E-07	Nox4	ENSRNOG00000013925	1	150,797,084
21821	A_44_P975945	1.135	0.191	5.4E-05	1.238	0.191	2.3E-05	Fam168a	ENSRNOG00000018873	1	165,724,451
15042	A_64_P043101	-0.832	0.146	8.1E-05	-0.982	0.146	1.6E-05	Dnhd1	ENSRNOG00000051291	1	170,471,272
18186	A_64_P016077	-0.951	0.154	3.8E-05	-0.952	0.154	3.7E-05	Dnhd1	ENSRNOG00000051291	1	170,471,272
4890	A_42_P797965	0.539	0.079	1.4E-05	0.497	0.079	3.2E-05	RGD1306959	ENSRNOG00000013744	1	174,330,695
13671	A_44_P212813	2.113	0.169	1.7E-08	2.699	0.169	9.0E-10	Acsm5	ENSRNOG00000031211	1	189,233,141
29269	A_64_P151353	3.026	0.130	9.1E-12	3.700	0.130	7.4E-13	Acsm5	ENSRNOG00000031211	1	189,241,593
21952	A_64_P024138	-1.464	0.163	7.3E-07	-0.923	0.163	8.4E-05	Sbk1	ENSRNOG00000057696	1	197,659,187
14224	A_64_P045578	1.176	0.119	2.7E-07	1.251	0.119	1.3E-07	Rnf40	ENSRNOG00000018840	1	199,037,544
22149	A_64_P056569	-0.771	0.114	1.5E-05	-0.778	0.114	1.4E-05	Fus	ENSRNOG00000023360	1	199,412,834
7351	A_42_P718022	1.317	0.233	8.8E-05	1.319	0.233	8.6E-05	Tnni2	ENSRNOG00000020276	1	215,609,036
5718	A_64_P119314	-3.232	0.499	2.4E-05	-3.573	0.499	8.5E-06	LOC689065	ENSRNOG00000036641	1	219,745,654
13480	A_44_P228859	0.932	0.095	2.7E-07	0.841	0.095	8.4E-07	Ust5r	ENSRNOG00000061890	1	224,533,219
2047	A_44_P1012567	2.491	0.159	1.2E-09	1.820	0.159	4.8E-08	Fads3	ENSRNOG00000020385	1	226,091,774
18981	A_64_P013243	-1.712	0.257	1.8E-05	-1.846	0.257	8.3E-06		ENSRNOG00000021811	1	239,319,737
26560	A_64_P071983	1.437	0.218	2.0E-05	1.532	0.218	1.0E-05	Ppp1r3c	ENSRNOG00000018494	1	255,376,833
15747	A_44_P139673	-1.538	0.177	1.1E-06	-2.912	0.177	6.1E-10	Cyp2c12	ENSRNOG00000047945	1	258,766,881
26738	A_64_P143725	0.547	0.097	8.7E-05	0.695	0.097	8.1E-06	Poll	ENSRNOG00000016748	1	265,298,797
25627	A_44_P107308	2.135	0.147	2.8E-09	2.094	0.147	3.6E-09	Gfra1	ENSRNOG00000017438	1	279,277,339
19125	A_64_P001710	1.391	0.187	5.7E-06	1.437	0.187	4.0E-06	Mblac2	ENSRNOG00000016252	2	9,578,345

528	A_42_P523612	-0.753	0.094	2.7E-06	-0.840	0.094	8.1E-07	Mef2c	ENSRNOG00000033134	2	11,658,568
6596	A_43_P13126	1.201	0.157	4.4E-06	1.054	0.157	1.7E-05	Acot12	ENSRNOG00000061414	2	20,857,202
655	A_42_P744291	-1.316	0.138	3.8E-07	-1.234	0.138	7.8E-07	Zbed3	ENSRNOG00000028941	2	25,041,185
15807	A_64_P068038	0.590	0.088	1.7E-05	0.551	0.088	3.3E-05	Hexb	ENSRNOG00000025274	2	28,003,186
19077	A_44_P131470	0.536	0.084	2.7E-05	0.483	0.084	7.3E-05	Hexb	ENSRNOG00000025274	2	28,003,186
28124	A_44_P468239	-1.011	0.123	1.9E-06	-1.116	0.123	6.5E-07	Sgtb	ENSRNOG00000011937	2	34,186,091
2831	A_42_P663871	0.646	0.114	8.7E-05	0.744	0.114	2.3E-05	Gzma	ENSRNOG00000010603	2	44,981,458
27193	A_64_P112889	-1.092	0.154	9.2E-06	-1.436	0.154	4.8E-07	Ccdc152	ENSRNOG00000039473	2	53,140,637
9724	A_43_P11817	1.126	0.180	3.2E-05	1.524	0.180	1.4E-06	Ghr	ENSRNOG00000015654	2	53,313,884
13369	A_44_P320858	0.787	0.086	5.9E-07	0.685	0.086	2.7E-06	Ghr	ENSRNOG00000015654	2	53,313,884
29351	A_64_P041812	1.050	0.128	2.0E-06	1.451	0.128	5.2E-08	Ghr	ENSRNOG00000015654	2	53,313,884
5037	A_44_P187181	-4.170	0.358	2.1E-07	-3.879	0.270	2.6E-08	Prlr	ENSRNOG00000057557	2	60,131,776
465	A_42_P524633	3.946	0.216	1.8E-10	3.908	0.216	2.0E-10	Fam134b	ENSRNOG00000057522	2	78,391,921
19499	A_44_P244851	1.990	0.247	2.4E-06	1.839	0.247	5.6E-06	Car3	ENSRNOG00000010079	2	88,135,410
22736	A_44_P1029241	1.756	0.291	4.7E-05	2.098	0.291	7.9E-06	Car1	ENSRNOG00000010698	2	88,217,188
7069	A_44_P575424	-1.469	0.178	1.9E-06	-1.026	0.178	7.2E-05	Chmp4c	ENSRNOG00000010238	2	93,641,497
5175	A_44_P362981	-1.673	0.233	8.4E-06	-1.667	0.233	8.7E-06	Fabp5	ENSRNOG00000049075	2	93,985,378
29777	A_44_P487504	1.219	0.124	2.8E-07	1.349	0.124	8.9E-08		ENSRNOG00000032715	2	148,262,110
16967	A_44_P773166	-1.044	0.120	1.1E-06	-0.966	0.120	2.4E-06	Tmem144	ENSRNOG00000010081	2	178,612,470
27712	A_64_P041307	-0.986	0.095	1.4E-07	-0.895	0.095	4.3E-07	Tars2	ENSRNOG00000057194	2	197,878,142
29595	A_64_P060667	-4.329	0.146	5.2E-10	-4.105	0.146	8.2E-10	Reg4	ENSRNOG00000019046	2	200,397,967
13762	A_64_P055472	0.879	0.121	7.4E-06	0.727	0.121	4.9E-05	Vangl1	ENSRNOG00000016477	2	204,625,743
10760	A_44_P109455	0.932	0.102	6.1E-07	0.875	0.102	1.2E-06	Gstm2	ENSRNOG00000018937	2	210,782,746
6775	A_64_P027971	3.463	0.193	2.2E-10	3.010	0.193	1.2E-09	Dnase2b	ENSRNOG00000016262	2	252,451,999
3199	A_44_P281352	1.261	0.106	3.2E-08	0.935	0.106	9.4E-07	Dnajb4	ENSRNOG00000013011	2	257,418,425
26230	A_44_P213133	-2.350	0.104	1.3E-11	-2.466	0.104	6.9E-12	Endog	ENSRNOG00000016033	3	8,741,766
22393	A_43_P18366	1.368	0.118	4.0E-08	1.218	0.118	1.5E-07	Tor1b	ENSRNOG00000006435	3	9,792,899
17373	A_42_P638620	1.086	0.110	2.7E-07	1.007	0.110	6.3E-07	Lcn2	ENSRNOG00000013973	3	11,417,546
24771	A_43_P10292	1.628	0.110	2.3E-09	1.237	0.110	5.8E-08	siat7D	ENSRNOG00000048870	3	11,607,225
13463	A_42_P835462	-1.062	0.132	2.5E-06	-1.421	0.132	9.6E-08	Ak1	ENSRNOG00000049056	3	11,653,529
14170	A_64_P141349	2.530	0.225	5.7E-08	2.565	0.225	4.9E-08	B3galt1	ENSRNOG00000007625	3	54,253,949
12228	A_64_P152790	1.510	0.141	1.0E-07	1.102	0.141	3.4E-06	Frzb	ENSRNOG00000007765	3	67,668,772
24139	A_44_P992697	-1.126	0.122	5.5E-07	-1.415	0.122	4.1E-08	Dusp19	ENSRNOG00000008868	3	67,849,966

Disease Models & Mechanisms • Supplementary information

1902	A_64_P018681	0.712	0.119	5.2E-05	0.728	0.119	4.3E-05	P2rx3	ENSRNOG00000008552	3	72,445,775
17375	A_43_P15275	-2.102	0.099	2.9E-11	-1.961	0.099	6.7E-11	Ptprj	ENSRNOG00000034025	3	79,390,956
18771	A_44_P107766	-1.335	0.138	3.2E-07	-1.319	0.138	3.7E-07	RGD1309540	ENSRNOG00000014798	3	80,349,145
3735	A_42_P665509	0.896	0.094	4.1E-07	1.000	0.094	1.2E-07	Mapk8ip1	ENSRNOG00000058478	3	81,304,181
23303	A_44_P362954	1.152	0.185	3.5E-05	1.075	0.185	6.8E-05	Cat	ENSRNOG00000008364	3	93,412,058
3427	A_44_P1024721	0.764	0.120	2.7E-05	0.956	0.120	2.7E-06	Slc28a2	ENSRNOG00000028668	3	114,355,798
27910	A_64_P158384	-1.350	0.159	1.4E-06	-1.106	0.159	1.1E-05	RGD1359452	ENSRNOG00000037124	3	118,427,851
16566	A_44_P1011953	-1.283	0.133	3.3E-07	-0.762	0.133	7.4E-05	Gpcpd1	ENSRNOG00000053201	3	125,213,607
28840	A_44_P243599	-0.830	0.126	4.6E-05	-0.770	0.095	7.3E-06	RGD1566320	ENSRNOG00000036971	3	138,765,027
5599	A_43_P18817	-0.955	0.156	6.0E-05	-1.225	0.170	1.3E-05	Ift52	ENSRNOG00000007692	3	159,392,193
20958	A_64_P002904	0.849	0.126	1.5E-05	0.726	0.126	7.1E-05	Pmepa1	ENSRNOG00000050404	3	171,342,646
14169	A_64_P124090	-0.749	0.111	1.6E-05	-0.656	0.111	5.9E-05	Ntsr1	ENSRNOG00000028708	3	175,885,894
15543	A_44_P307964	-0.725	0.089	2.1E-06	-0.639	0.089	8.2E-06	Polr3k	ENSRNOG00000017843	3	177,374,812
9300	A_64_P054808	-2.114	0.188	5.8E-08	-1.655	0.188	9.2E-07	Cd36	ENSRNOG00000040108	4	14,001,761
15617	A_64_P113793	-1.749	0.160	8.0E-08	-1.101	0.160	1.3E-05	Cd36	ENSRNOG00000040108	4	14,151,374
16630	A_44_P1003728	2.170	0.141	4.0E-09	1.736	0.141	4.7E-08	Lmod2	ENSRNOG00000049639	4	51,661,654
30022	A_44_P260072	0.698	0.094	5.8E-06	0.754	0.094	2.6E-06	Tbxas1	ENSRNOG00000007918	4	66,670,618
7276	A_64_P135813	0.716	0.123	6.5E-05	0.821	0.123	1.7E-05	Mkrn1	ENSRNOG00000009280	4	67,206,426
7404	A_43_P23115	4.828	0.192	3.4E-12	3.929	0.192	4.4E-11	Inmt	ENSRNOG00000011250	4	85,386,231
841	A_44_P299247	-2.158	0.100	2.3E-11	-1.772	0.100	2.6E-10	Aqp1	ENSRNOG00000011648	4	85,551,503
13336	A_64_P097974	1.289	0.091	4.0E-09	1.248	0.091	5.9E-09	St3gal5	ENSRNOG00000010284	4	99,937,558
859	A_64_P145166	0.574	0.102	9.5E-05	0.692	0.102	1.6E-05	Ggcx	ENSRNOG00000012975	4	100,277,391
930	A_44_P512828	-0.607	0.107	8.7E-05	-0.641	0.107	5.2E-05	Nup210	ENSRNOG00000005390	4	122,741,110
10944	A_44_P337351	-0.767	0.118	2.2E-05	-0.661	0.118	9.2E-05	Cxcl12	ENSRNOG00000013589	4	149,261,044
11102	A_44_P917327	1.053	0.164	2.6E-05	0.938	0.164	7.9E-05	Lrtm2	ENSRNOG00000007508	4	151,390,263
17411	A_64_P095378	1.407	0.188	2.6E-05	1.279	0.188	5.7E-05	Lrtm2	ENSRNOG00000007508	4	151,390,263
22190	A_64_P038872	1.530	0.238	2.6E-05	1.395	0.238	6.2E-05	A2m	ENSRNOG00000028896	4	154,309,426
2217	A_64_P091148	2.651	0.255	1.4E-07	2.270	0.255	8.1E-07	Tuba3a	ENSRNOG00000031707	4	157,798,868
25815	A_43_P13004	-4.095	0.331	1.9E-08	-4.757	0.331	3.2E-09	Cdh17	ENSRNOG00000015562	5	25,391,115
1894	A_64_P118499	0.947	0.164	7.0E-05	0.914	0.164	9.7E-05	RGD1561916	ENSRNOG00000013320	5	57,896,475
21610	A_64_P013398	-2.611	0.154	4.4E-10	-2.275	0.154	2.3E-09	Gne	ENSRNOG00000014365	5	59,543,101
696	A_64_P091926	1.306	0.122	1.1E-07	1.354	0.122	7.0E-08	Acnat2	ENSRNOG00000051912	5	64,674,250
19017	A_64_P078573	1.459	0.113	1.1E-08	1.446	0.113	1.3E-08		ENSRNOG00000056688	5	64,718,131

Disease Models & Mechanisms • Supplementary information

6106	A_44_P283971	1.122	0.159	1.0E-05	0.911	0.159	7.9E-05	Zfp189	ENSRNOG00000006972	5	64,789,456
321	A_42_P698240	-0.809	0.113	8.4E-06	-0.784	0.113	1.2E-05	Ptgr1	ENSRNOG00000015072	5	76,129,441
20157	A_64_P137461	-1.506	0.204	6.2E-06	-1.296	0.204	2.8E-05	Bspry	ENSRNOG00000015105	5	78,334,284
13424	A_44_P175822	-1.006	0.129	3.5E-06	-0.772	0.129	5.1E-05	Hdhd3	ENSRNOG00000015195	5	78,361,647
29448	A_64_P090543	-1.135	0.122	5.0E-07	-0.868	0.122	8.9E-06	Hdhd3	ENSRNOG00000015195	5	78,361,647
29932	A_64_P082212	-1.717	0.110	1.3E-09	-1.426	0.110	1.2E-08	Kif12	ENSRNOG00000007080	5	79,008,363
13242	A_44_P1040796	1.527	0.091	4.7E-10	1.553	0.091	3.8E-10	Atp6v1g1	ENSRNOG00000008163	5	79,367,663
22473	A_64_P040989	2.030	0.199	1.8E-07	2.868	0.199	3.1E-09	Cyp4a8	ENSRNOG00000008842	5	134,008,255
23745	A_44_P175875	0.930	0.125	5.7E-06	0.896	0.125	8.5E-06	Pigv	ENSRNOG00000000121	5	151,895,016
4895	A_64_P146507	1.747	0.211	1.8E-06	1.447	0.211	1.3E-05		ENSRNOG00000042062	5	173,139,628
6351	A_44_P163242	-0.979	0.153	2.6E-05	-1.120	0.153	6.7E-06	Epcam	ENSRNOG00000015667	6	11,298,216
24965	A_64_P122457	0.510	0.082	3.7E-05	0.543	0.082	2.0E-05	Rbks	ENSRNOG00000004710	6	26,051,396
3481	A_43_P19743	-2.642	0.142	1.5E-10	-2.664	0.142	1.3E-10	Krtcap3	ENSRNOG00000047941	6	26,486,695
18372	A_64_P017363	3.713	0.140	1.7E-12	2.448	0.140	2.9E-10	Slc30a3	ENSRNOG00000006204	6	26,642,783
6427	A_64_P037020	0.657	0.093	1.0E-05	0.550	0.093	5.9E-05	Seli	ENSRNOG00000059295	6	27,487,284
24275	A_44_P413979	0.691	0.095	7.2E-06	0.970	0.095	1.8E-07	Rab10	ENSRNOG00000047088	6	27,721,120
2367	A_42_P816740	0.956	0.105	6.7E-07	0.844	0.105	2.6E-06	Sh3yl1	ENSRNOG00000005522	6	49,852,130
12825	A_44_P267628	1.597	0.127	1.5E-08	1.286	0.127	1.9E-07	LOC690226	ENSRNOG00000025648	6	95,502,775
4312	A_44_P684199	2.574	0.182	4.0E-09	2.059	0.182	5.5E-08	Dhrs7	ENSRNOG00000025648	6	95,502,845
3166	A_44_P353157	-2.621	0.205	8.4E-08	-3.148	0.155	7.3E-10	Tmem30b	ENSRNOG00000008046	6	96,442,682
8745	A_44_P260589	-1.001	0.125	7.6E-06	-1.084	0.125	3.6E-06		ENSRNOG00000009448	6	107,245,820
15326	A_64_P103936	-4.755	0.217	3.2E-10	-4.713	0.217	3.5E-10	Acot5	ENSRNOG00000032508	6	107,550,904
1038	A_44_P470661	-2.094	0.121	3.4E-10	-2.767	0.121	1.1E-11	Serpina3m	ENSRNOG00000009921	6	127,816,055
29681	A_44_P412140	-4.335	0.123	4.8E-14	-4.868	0.123	1.1E-14	LOC299282	ENSRNOG00000010478	6	128,046,780
2823	A_42_P581060	0.864	0.097	8.7E-07	1.062	0.097	8.5E-08	Glrx5	ENSRNOG00000004206	6	128,750,795
12961	A_44_P974862	1.161	0.157	6.0E-06	1.191	0.157	4.6E-06	Pld4	ENSRNOG00000028566	6	137,323,713
17240	A_44_P147572	-0.836	0.134	3.4E-05	-0.900	0.134	1.7E-05		ENSRNOG00000034190	6	138,508,753
29256	A_44_P321532	1.125	0.083	6.5E-09	0.819	0.083	2.6E-07	Apon	ENSRNOG00000033466	7	2,677,199
1509	A_42_P744495	1.074	0.133	2.4E-06	0.789	0.133	5.5E-05	Cd63	ENSRNOG00000007650	7	3,320,103
23314	A_44_P462040	1.116	0.116	3.6E-07	1.293	0.116	6.6E-08	Mettl7b	ENSRNOG00000007927	7	3,386,522
14192	A_64_P161250	0.810	0.088	5.8E-07	1.039	0.088	3.4E-08	Mier2	ENSRNOG00000000175	7	13,039,781
10600	A_64_P099017	-2.291	0.213	1.0E-07	-2.073	0.213	3.1E-07	Cyp4f17	ENSRNOG00000062306	7	14,529,483
2475	A_64_P079666	1.775	0.135	9.7E-09	1.648	0.135	2.3E-08	Cyp4f5	ENSRNOG00000042496	7	14,559,878

Disease Models & Mechanisms • Supplementary information

15805	A_64_P255450	-1.071	0.174	3.8E-05	-0.979	0.174	8.9E-05	Zfp799	ENSRNOG00000032552	7	15,072,703
17688	A_64_P012739	-1.126	0.155	7.4E-06	-1.162	0.155	5.3E-06	LOC314600	ENSRNOG00000048577	7	15,785,410
21207	A_44_P607542	1.163	0.146	2.8E-06	1.454	0.146	2.4E-07		ENSRNOG00000005758	7	24,313,885
24430	A_44_P1038341	0.733	0.101	7.6E-06	0.921	0.101	6.5E-07	Hal	ENSRNOG00000004502	7	34,326,087
1344	A_42_P647599	0.962	0.100	3.6E-07	1.095	0.100	8.2E-08	Dcn	ENSRNOG00000004554	7	38,742,051
17690	A_44_P216654	0.693	0.111	3.3E-05	0.703	0.111	2.9E-05	Nap1l1	ENSRNOG00000003890	7	54,213,319
23558	A_64_P144913	2.073	0.151	5.7E-09	1.607	0.151	1.1E-07	Nab2	ENSRNOG00000008415	7	70,969,905
19834	A_64_P036553	4.049	0.135	3.7E-13	3.295	0.135	4.8E-12	Rdh2	ENSRNOG00000029651	7	70,980,422
27443	A_64_P036548	3.547	0.194	1.7E-10	3.225	0.194	5.4E-10	Rdh2	ENSRNOG00000029651	7	71,057,911
4653	A_44_P516093	1.009	0.106	4.0E-07	1.321	0.106	1.8E-08	Colec10	ENSRNOG00000008591	7	93,975,451
14838	A_64_P006590	2.181	0.287	4.6E-06	3.816	0.287	8.2E-09	Cyp2b2	ENSRNOG00000033680	7	99,142,450
1609	A_64_P143947	-0.729	0.102	8.6E-06	-0.691	0.102	1.5E-05		ENSRNOG00000055178	7	101,138,860
2438	A_42_P797218	1.591	0.117	6.5E-09	1.484	0.117	1.5E-08	Gpt	ENSRNOG00000033915	7	117,759,083
24492	A_43_P16804	0.636	0.112	8.5E-05	0.812	0.112	7.7E-06	Pole3	ENSRNOG00000004843	7	118,816,655
28509	A_44_P267706	-1.472	0.167	9.2E-07	-1.091	0.167	2.1E-05	Apol11a	ENSRNOG00000023122	7	118,840,634
15205	A_64_P111893	-2.002	0.182	7.5E-08	-1.372	0.182	4.9E-06	Apol9a	ENSRNOG00000023410	7	118,933,812
25974	A_43_P12557	0.820	0.097	1.5E-06	0.707	0.097	7.4E-06	Polr2f	ENSRNOG00000011214	7	120,380,544
3278	A_64_P001211	0.460	0.078	6.1E-05	0.483	0.078	3.8E-05	Cyp2d5	ENSRNOG00000029128	7	123,621,102
13515	A_64_P019790	0.448	0.078	7.3E-05	0.464	0.078	5.2E-05	Cyp2d1	ENSRNOG00000029179	7	123,638,765
21368	A_44_P914022	1.607	0.160	2.0E-06	1.094	0.160	5.3E-05	LOC689933	ENSRNOG00000042056	7	130,368,819
1918	A_64_P019051	-0.804	0.104	3.9E-06	-0.850	0.104	2.2E-06	Smug1	ENSRNOG00000036842	7	144,778,339
8405	A_42_P660129	1.782	0.164	8.9E-08	2.808	0.164	4.0E-10	Casp12	ENSRNOG00000033434	8	2,659,865
17711	A_44_P119527	-1.698	0.183	5.1E-07	-1.704	0.183	4.9E-07	Med17	ENSRNOG00000051989	8	13,526,025
11775	A_44_P518434	-1.027	0.122	1.6E-06	-0.988	0.122	2.4E-06	Mrpl4	ENSRNOG00000020659	8	22,021,213
582	A_42_P577421	1.567	0.261	5.0E-05	1.900	0.261	7.2E-06	Nnmt	ENSRNOG00000005930	8	52,937,972
9382	A_44_P531758	1.556	0.178	1.0E-06	1.254	0.178	1.0E-05	Cryab	ENSRNOG00000010524	8	55,178,289
27101	A_64_P118047	1.003	0.151	1.9E-05	1.127	0.151	5.7E-06	Prtg	ENSRNOG00000055251	8	79,489,790
11738	A_43_P13967	0.645	0.116	9.9E-05	0.688	0.116	5.3E-05	Gsta2	ENSRNOG00000000201	8	85,553,734
29727	A_44_P119575	0.912	0.128	1.4E-05	1.228	0.128	7.2E-07		ENSRNOG00000009613	8	91,099,491
22148	A_44_P283339	1.939	0.133	2.8E-09	1.383	0.133	1.5E-07	Tmed3	ENSRNOG00000013889	8	97,083,120
5815	A_43_P11432	-0.674	0.081	1.6E-06	-0.629	0.081	3.4E-06	Acaa1a	ENSRNOG00000032908	8	128,027,958
11481	A_44_P113974	0.899	0.121	6.0E-06	0.831	0.121	1.4E-05	Ccr5	ENSRNOG00000049115	8	133,210,473
1203	A_44_P974940	2.290	0.145	1.1E-09	2.514	0.145	3.4E-10	RGD1562392	ENSRNOG00000047369	9	4,072,901

7083	A_64_P001334	2.745	0.147	1.3E-10	3.038	0.147	3.8E-11	Sult1c2	ENSRNOG00000047369	9	4,094,995
257	A_42_P501679	-0.795	0.119	1.7E-05	-0.803	0.119	1.5E-05	M6prbp1	ENSRNOG00000048834	9	10,773,901
5143	A_43_P18744	-1.127	0.126	5.6E-06	-1.285	0.112	6.2E-07	Enpp5	ENSRNOG00000010232	9	19,476,646
17867	A_44_P1029892	4.273	0.156	1.2E-12	4.225	0.156	1.4E-12	Tmem14a	ENSRNOG00000046593	9	27,343,853
23432	A_64_P039432	-0.655	0.097	1.6E-05	-0.673	0.097	1.2E-05	Clk1	ENSRNOG00000025768	9	65,307,995
25117	A_64_P060967	0.777	0.102	4.4E-06	0.900	0.102	9.1E-07	Lancl1	ENSRNOG00000013557	9	74,048,244
20772	A_43_P15154	-1.760	0.257	1.4E-05	-1.509	0.257	6.1E-05	Ihh	ENSRNOG00000018059	9	82,214,440
18795	A_43_P10901	1.372	0.094	2.6E-09	1.021	0.094	8.5E-08	Itm2c	ENSRNOG00000017359	9	92,916,469
11494	A_44_P123492	-1.195	0.095	1.5E-08	-1.054	0.095	6.7E-08	Epb41l3	ENSRNOG00000016724	9	117,538,009
9066	A_64_P027288	2.191	0.160	5.6E-09	2.559	0.160	8.7E-10	RGD1564516	ENSRNOG00000038894	10	31,880,918
19797	A_64_P017631	-0.714	0.117	4.2E-05	-0.763	0.117	2.2E-05	RGD1563669	ENSRNOG00000053961	10	46,314,375
21936	A_44_P493911	3.290	0.262	1.6E-08	2.812	0.262	1.0E-07	Slc13a5	ENSRNOG00000014870	10	58,834,538
8183	A_64_P033710	1.137	0.172	2.9E-05	1.114	0.172	3.5E-05	Hlf	ENSRNOG00000002456	10	77,918,191
22560	A_64_P157691	-1.460	0.114	1.3E-08	-1.785	0.114	1.2E-09	RGD1559482	ENSRNOG00000048771	10	104,952,237
25284	A_44_P356866	-1.493	0.175	1.4E-06	-1.753	0.175	2.3E-07	RGD1311078	ENSRNOG00000060307	10	105,073,077
22449	A_42_P539095	-0.646	0.096	1.5E-05	-0.781	0.096	2.1E-06	LOC688311	ENSRNOG00000049235	10	109,639,054
21718	A_44_P239300	1.331	0.121	7.9E-08	1.111	0.121	6.1E-07	RGD1307119	ENSRNOG00000036680	10	109,840,047
18404	A_43_P13256	0.669	0.102	2.1E-05	0.764	0.102	5.3E-06	Dcxr	ENSRNOG00000050315	10	109,909,646
6976	A_44_P352538	0.721	0.095	4.6E-06	0.550	0.095	6.9E-05	Sectm1a	ENSRNOG00000036672	10	110,274,768
22819	A_44_P159569	1.289	0.103	1.7E-08	1.140	0.103	7.2E-08	Wdr45l	ENSRNOG00000036662	10	110,555,629
28640	A_44_P228942	-0.762	0.137	9.8E-05	-0.809	0.137	5.6E-05	Runx1	ENSRNOG00000001704	11	32,858,830
6823	A_44_P761087	-0.861	0.122	1.0E-05	-1.099	0.122	7.5E-07	Ets2	ENSRNOG00000001647	11	36,075,709
6166	A_64_P024394	0.526	0.092	7.6E-05	0.906	0.092	2.6E-07	Hrg	ENSRNOG00000001809	11	81,639,952
2087	A_64_P015758	-1.105	0.195	8.3E-05	-1.223	0.195	3.2E-05		ENSRNOG00000050000	11	86,092,468
9870	A_64_P012902	0.761	0.096	3.1E-06	0.606	0.096	3.2E-05	Sdf2l1	ENSRNOG00000001859	11	88,122,271
14260	A_64_P019401	0.787	0.129	4.1E-05	0.742	0.129	7.2E-05	Sdf2l1	ENSRNOG00000001859	11	88,122,271
1534	A_42_P762213	-2.406	0.185	2.7E-08	-2.319	0.170	1.6E-08	Lrrc8e	ENSRNOG00000028460	12	2,568,382
29020	A_44_P116660	-0.671	0.105	2.6E-05	-0.955	0.105	6.3E-07	Cd209b	ENSRNOG00000029881	12	2,826,378
3552	A_42_P679814	1.385	0.156	8.5E-07	1.690	0.156	9.0E-08	Cyp3a18	ENSRNOG00000000969	12	10,636,275
578	A_44_P1009594	1.804	0.147	2.0E-08	1.754	0.147	2.8E-08	Cyp3a62	ENSRNOG00000001379	12	18,678,594
17338	A_44_P1037342	-2.575	0.141	1.8E-10	-2.375	0.141	5.0E-10	Cyp3a9	ENSRNOG00000046643	12	19,114,399
19145	A_64_P081501	0.750	0.119	3.2E-05	1.291	0.119	9.2E-08	Mcm7	ENSRNOG00000001349	12	19,314,016
23117	A_42_P540609	-0.646	0.112	7.0E-05	-0.641	0.112	7.4E-05	LOC680711	ENSRNOG00000031343	12	19,440,501

25622	A_64_P119299	-0.773	0.094	2.0E-06	-0.775	0.094	1.9E-06		ENSRNOG00000027061	12	19,512,591
8198	A_64_P027108	-0.802	0.116	1.2E-05	-0.811	0.116	1.1E-05	RGD1562319	ENSRNOG00000039216	12	19,561,347
551	A_64_P164498	1.342	0.095	4.2E-09	1.408	0.095	2.3E-09	LOC685438	ENSRNOG00000047079	12	20,276,121
18384	A_64_P079080	1.158	0.096	2.5E-08	1.219	0.096	1.4E-08		ENSRNOG00000059625	12	20,667,601
7888	A_64_P156448	1.063	0.101	1.2E-07	1.103	0.101	8.0E-08	LOC685157	ENSRNOG00000050981	12	21,362,205
15839	A_64_P032265	1.708	0.099	3.8E-10	1.817	0.099	1.8E-10		ENSRNOG00000033017	12	21,678,580
28441	A_64_P022248	-0.719	0.127	8.6E-05	-0.979	0.127	4.0E-06	Ccdc62	ENSRNOG00000038002	12	38,093,050
29139	A_44_P838992	-2.255	0.197	1.1E-07	-2.984	0.214	1.3E-08	LOC687609	ENSRNOG00000042607	12	38,880,377
6196	A_44_P686406	1.339	0.131	1.7E-07	0.806	0.131	3.8E-05	RGD1562310	ENSRNOG00000001123	12	43,940,798
15981	A_44_P224631	0.837	0.120	1.1E-05	0.912	0.120	4.5E-06	Dao	ENSRNOG00000054962	12	48,365,784
14258	A_43_P13249	2.207	0.209	1.2E-07	2.917	0.209	4.7E-09	Acmsd	ENSRNOG00000003884	13	44,424,689
13363	A_42_P667960	0.747	0.115	2.2E-05	0.939	0.115	2.1E-06	Ikbke	ENSRNOG00000025100	13	48,056,394
824	A_44_P137633	-2.198	0.291	4.9E-06	-1.828	0.291	3.2E-05	Adora1	ENSRNOG00000003442	13	51,076,165
16686	A_44_P283508	1.575	0.137	4.5E-08	1.458	0.137	1.1E-07	Fmo1	ENSRNOG00000034191	13	80,745,347
4122	A_44_P358194	0.969	0.114	1.4E-06	1.095	0.114	3.6E-07	Fmo3	ENSRNOG00000003620	13	80,862,963
14514	A_44_P180259	0.663	0.109	4.3E-05	0.816	0.109	5.2E-06	Dpt	ENSRNOG00000002947	13	83,073,550
12064	A_64_P027625	-1.761	0.184	3.7E-07	-1.025	0.184	9.9E-05	Rxrg	ENSRNOG00000004537	13	85,818,427
339	A_42_P743580	1.704	0.146	5.3E-07	-2.229	0.243	4.4E-06	Rgs4	ENSRNOG00000002773	13	88,061,108
21779	A_44_P306439	0.687	0.115	5.3E-05	0.809	0.115	1.1E-05	Fcgr3a	ENSRNOG00000024382	13	89,385,859
25592	A_44_P575255	3.161	0.133	6.7E-12	2.966	0.133	1.5E-11	RGD1562658	ENSRNOG00000034221	13	89,480,058
3268	A_64_P005029	3.908	0.264	2.3E-09	3.025	0.264	4.8E-08	Pcp4l1	ENSRNOG00000003209	13	89,565,813
24112	A_64_P109496	3.085	0.215	3.4E-09	2.726	0.215	1.5E-08	Nr1i3	ENSRNOG00000003260	13	89,586,283
29177	A_64_P109497	2.684	0.168	9.2E-10	2.315	0.168	5.5E-09	Nr1i3	ENSRNOG00000003260	13	89,586,283
5407	A_42_P565621	0.561	0.084	1.7E-05	0.818	0.084	3.1E-07	Slamf9	ENSRNOG00000008045	13	90,839,411
1758	A_64_P027298	1.003	0.135	5.8E-06	0.968	0.135	8.3E-06	Ptpn14	ENSRNOG00000003407	13	108,841,482
9059	A_42_P803590	-2.145	0.153	4.5E-09	-2.213	0.153	3.1E-09	LOC289378	ENSRNOG00000046460	13	109,663,364
2950	A_42_P683634	0.614	0.086	9.3E-06	0.619	0.086	8.5E-06	Sparcl1	ENSRNOG00000015093	14	6,994,190
783	A_64_P071159	-1.822	0.096	1.1E-10	-1.723	0.096	2.1E-10	LOC360919	ENSRNOG00000039504	14	19,072,677
17181	A_64_P071158	-1.872	0.101	1.5E-10	-1.746	0.101	3.4E-10	LOC360919	ENSRNOG00000039504	14	19,072,677
5419	A_64_P045325	2.210	0.093	6.7E-12	2.616	0.093	8.1E-13		ENSRNOG00000001973	14	22,192,970
11942	A_64_P062143	1.330	0.112	3.1E-08	1.740	0.112	1.3E-09	Ugt2a3	ENSRNOG00000001973	14	22,192,970
11973	A_44_P236068	0.685	0.103	1.8E-05	0.795	0.103	3.8E-06	Ugt2b36	ENSRNOG00000001980	14	22,553,662
27421	A_44_P447623	0.694	0.110	3.1E-05	0.824	0.110	5.4E-06	Ugdh	ENSRNOG00000002643	14	44,479,614

22	A_44_P992306	-1.110	0.192	7.0E-05	-1.297	0.192	1.5E-05	Gnpda2	ENSRNOG00000002177	14	62,646,110
22164	A_64_P132516	-1.651	0.175	4.3E-07	-1.943	0.175	6.8E-08	Rtn4	ENSRNOG00000004621	14	114,126,943
29558	A_44_P477322	-1.417	0.174	2.2E-06	-1.999	0.174	4.7E-08	Rtn4	ENSRNOG00000004621	14	114,126,943
11690	A_44_P330188	1.889	0.117	8.1E-10	1.893	0.117	7.9E-10	Acox2	ENSRNOG00000007378	15	18,451,144
3800	A_64_P100123	-0.910	0.102	8.2E-07	-0.938	0.102	5.9E-07	Pxk	ENSRNOG00000008024	15	18,616,589
8986	A_44_P1057690	0.638	0.090	9.2E-06	0.637	0.090	9.4E-06	Dnase1l3	ENSRNOG00000009291	15	18,710,492
5685	A_42_P775609	-1.005	0.145	1.2E-05	-0.951	0.145	2.0E-05	Jub	ENSRNOG00000012791	15	33,218,456
2966	A_64_P106162	0.522	0.092	8.7E-05	0.569	0.092	3.8E-05	Pck2	ENSRNOG00000018536	15	34,216,833
6846	A_64_P005043	1.219	0.093	9.8E-09	1.045	0.093	6.0E-08	Nt5dc2	ENSRNOG00000018358	16	7,212,488
18552	A_64_P142248	-1.152	0.171	1.5E-05	-1.255	0.171	6.4E-06	Colq	ENSRNOG00000019615	16	7,681,576
19242	A_64_P119675	-0.963	0.165	6.4E-05	-0.920	0.165	9.8E-05	Jund	ENSRNOG00000019568	16	20,486,707
25971	A_64_P004531	-0.623	0.092	1.4E-05	-0.778	0.092	1.4E-06	LOC688966	ENSRNOG00000020427	16	21,029,134
28374	A_64_P315172	0.996	0.135	1.7E-05	1.420	0.135	5.8E-07	Nat2	ENSRNOG00000049498	16	23,991,570
3982	A_64_P007462	1.283	0.127	2.0E-07	1.149	0.127	6.8E-07	Spcs3	ENSRNOG00000038933	16	40,050,734
26075	A_64_P041016	-1.211	0.113	1.0E-07	-0.880	0.113	3.5E-06	Wwc2	ENSRNOG00000013248	16	47,368,768
18238	A_43_P11534	-0.577	0.100	7.3E-05	-0.640	0.100	2.7E-05	Klkb1	ENSRNOG00000014118	16	50,152,008
9554	A_64_P048315	1.548	0.109	3.6E-09	1.378	0.109	1.4E-08		ENSRNOG00000055088	16	59,517,000
11412	A_44_P1042163	0.821	0.096	1.2E-06	0.634	0.096	1.9E-05	RGD1310414	ENSRNOG00000023494	16	64,745,207
26021	A_64_P085286	1.218	0.138	8.9E-07	1.335	0.138	3.2E-07	Rnf170	ENSRNOG00000045943	16	70,705,128
28250	A_64_P017645	-0.617	0.100	3.9E-05	-0.721	0.100	8.1E-06	Atp7b	ENSRNOG00000012878	16	74,865,516
712	A_42_P558354	-0.721	0.090	2.5E-06	-0.814	0.090	6.6E-07	Cln8	ENSRNOG00000012565	16	79,838,212
26198	A_64_P064008	2.060	0.315	2.2E-05	2.015	0.315	2.7E-05		ENSRNOG00000061436	17	445,236
11386	A_64_P014278	-0.961	0.117	1.9E-06	-0.728	0.117	3.4E-05	LOC680692	ENSRNOG00000018400	17	5,382,034
23308	A_64_P136894	1.159	0.160	7.7E-06	0.918	0.160	7.7E-05	Omd	ENSRNOG00000039560	17	15,484,269
4180	A_64_P165035	6.544	0.115	1.2E-16	6.715	0.115	8.6E-17	RGD1305679	ENSRNOG00000014481	17	22,619,891
8900	A_64_P059262	0.742	0.125	5.5E-05	0.851	0.125	1.4E-05	Riok1	ENSRNOG00000014049	17	27,451,832
23925	A_64_P030919	-0.911	0.140	2.2E-05	-0.832	0.140	5.4E-05		ENSRNOG00000023538	17	42,133,076
28972	A_64_P060715	-1.519	0.170	8.0E-07	-1.376	0.170	2.3E-06		ENSRNOG00000022505	17	43,458,553
29478	A_64_P100482	-1.604	0.153	1.3E-07	-1.367	0.153	7.8E-07	LOC684568	ENSRNOG00000022505	17	43,458,553
7060	A_42_P555365	1.104	0.159	1.1E-05	1.542	0.159	3.1E-07	Slc17a3	ENSRNOG00000032745	17	43,537,293
14149	A_64_P080667	-1.461	0.153	3.8E-07	-1.383	0.153	7.0E-07	LOC684841	ENSRNOG00000045644	17	43,807,540
13074	A_64_P130572	0.736	0.100	6.3E-06	0.576	0.100	7.2E-05	Hist1h2bc	ENSRNOG00000059382	17	44,738,330
13903	A_44_P515798	4.879	0.168	3.1E-12	4.529	0.168	7.2E-12	Ucma	ENSRNOG00000017987	17	77,261,731

Disease Models & Mechanisms • Supplementary information

25656	A_44_P245616	1.340	0.193	1.2E-05	1.618	0.193	1.6E-06	Nid1	ENSRNOG00000002461	17	90,627,101
7301	A_64_P024440	-1.203	0.124	3.2E-07	-0.871	0.124	1.0E-05	Rbbp8	ENSRNOG00000012899	18	3,163,214
10422	A_64_P024439	-1.141	0.096	2.9E-08	-0.874	0.096	6.2E-07	Rbbp8	ENSRNOG00000012899	18	3,163,214
26378	A_64_P026928	0.914	0.136	1.7E-05	1.384	0.136	1.9E-07	Prr16	ENSRNOG00000019744	18	46,148,849
2039	A_64_P135224	6.025	0.228	1.9E-12	6.883	0.228	3.6E-13	RGD1309362	ENSRNOG00000038960	18	55,505,993
6991	A_64_P050921	-2.248	0.337	1.8E-05	-2.289	0.337	1.5E-05	Ablim3	ENSRNOG00000019365	18	57,201,740
22519	A_64_P021320	-0.506	0.088	7.3E-05	-0.534	0.088	4.4E-05	Pqlc1	ENSRNOG00000059215	18	76,770,012
18626	A_64_P097078	-1.813	0.185	2.9E-07	-1.166	0.185	3.1E-05	Pdp2	ENSRNOG00000012343	19	601,469
2495	A_44_P555689	2.477	0.276	7.5E-07	2.184	0.276	2.9E-06	LOC291863	ENSRNOG00000015519	19	15,033,108
17810	A_64_P148413	1.773	0.133	7.6E-09	1.774	0.133	7.5E-09	Ces1d	ENSRNOG00000015519	19	15,033,108
9089	A_64_P005208	-2.387	0.204	3.7E-08	-1.679	0.204	1.9E-06	Nqo1	ENSRNOG00000012772	19	38,422,164
1018	A_44_P222122	2.564	0.147	3.1E-10	2.605	0.147	2.6E-10	Bcmo1	ENSRNOG00000012027	19	49,637,016
6248	A_64_P157604	-0.824	0.122	1.6E-05	-0.848	0.122	1.2E-05	Cpne7	ENSRNOG00000015397	19	55,929,609
26400	A_64_P064371	-1.330	0.158	1.5E-06	-1.406	0.158	8.2E-07	Spata2L	ENSRNOG00000016167	19	56,037,077
17609	A_64_P058266	-2.143	0.293	6.8E-06	-2.158	0.293	6.3E-06	Cox6c1	ENSRNOG00000032602	19	56,983,807
4436	A_42_P627572	-1.481	0.122	2.4E-08	-1.078	0.122	9.1E-07	Mdc1	ENSRNOG00000032813	20	3,405,285
14913	A_44_P914438	2.115	0.229	5.4E-07	2.099	0.229	5.9E-07	Cdkn1a	ENSRNOG00000000521	20	6,356,423
1872	A_64_P117171	0.774	0.103	5.0E-06	0.653	0.103	2.8E-05	LOC689755	ENSRNOG00000000531	20	8,384,088
15230	A_64_P048033	1.525	0.177	1.2E-06	1.371	0.177	3.7E-06	Agpat3	ENSRNOG00000001205	20	11,114,164
14331	A_64_P152447	2.310	0.146	1.0E-09	3.070	0.146	3.2E-11	Gstt3	ENSRNOG00000001242	20	13,817,795
19033	A_44_P1015203	2.407	0.141	4.0E-10	3.197	0.141	1.2E-11	Gstt3	ENSRNOG00000001242	20	13,817,795
8444	A_44_P1005649	1.422	0.129	7.5E-08	1.341	0.129	1.5E-07	Dnajc12	ENSRNOG00000051960	20	26,893,016
21326	A_44_P496643	-1.641	0.268	4.1E-05	-1.742	0.268	2.3E-05		ENSRNOG00000048535	20	29,280,386
23912	A_64_P126135	-1.877	0.163	4.5E-08	-1.508	0.163	5.5E-07		ENSRNOG00000055765	L568414	23,534
711	A_64_P157099	5.460	0.179	3.0E-13	5.061	0.179	7.8E-13		ENSRNOG00000031780	MT	1
889	A_44_P403017	0.638	0.106	4.9E-05	0.638	0.106	4.9E-05		ENSRNOG00000031780	MT	1
5482	A_64_P326359	1.780	0.171	1.4E-07	1.579	0.171	5.3E-07		ENSRNOG00000031780	MT	1
7877	A_64_P156662	2.486	0.191	1.1E-08	2.116	0.191	7.0E-08		ENSRNOG00000031780	MT	1
8649	A_64_P164474	0.935	0.145	2.5E-05	1.360	0.145	4.7E-07		ENSRNOG00000031780	MT	1
10091	A_64_P150008	-1.370	0.180	4.4E-06	-1.554	0.180	1.1E-06		ENSRNOG00000031780	MT	1
10171	A_64_P022135	1.523	0.137	6.4E-08	1.313	0.137	3.5E-07		ENSRNOG00000031780	MT	1
10544	A_64_P085915	-1.260	0.137	5.6E-07	-1.076	0.137	3.1E-06		ENSRNOG00000031780	MT	1
10820	A_64_P143542	1.650	0.188	9.7E-07	1.907	0.188	1.9E-07		ENSRNOG00000031780	MT	1

Disease Models & Mechanisms • Supplementary information

11809	A_64_P024675	-1.786	0.143	1.7E-08	-1.953	0.143	5.7E-09		ENSRNOG00000031780	MT	1
12812	A_64_P038099	-0.697	0.117	5.3E-05	-0.828	0.117	9.6E-06		ENSRNOG00000031780	MT	1
12900	A_64_P054953	2.614	0.119	1.8E-11	2.940	0.119	4.2E-12		ENSRNOG00000031780	MT	1
12960	A_64_P158343	0.850	0.146	6.8E-05	0.901	0.146	3.9E-05		ENSRNOG00000031780	MT	1
16302	A_64_P079528	-1.095	0.125	3.3E-06	-0.972	0.102	1.5E-06		ENSRNOG00000031780	MT	1
17252	A_44_P900209	-0.797	0.133	4.9E-05	-0.904	0.133	1.4E-05		ENSRNOG00000031780	MT	1
21135	A_64_P118308	-1.630	0.138	7.8E-08	-1.514	0.127	6.9E-08		ENSRNOG00000031780	MT	1
22371	A_64_P039036	-0.674	0.098	1.3E-05	-0.635	0.098	2.4E-05		ENSRNOG00000031780	MT	1
22836	A_64_P123792	0.877	0.126	1.1E-05	1.654	0.126	9.4E-09		ENSRNOG00000031780	MT	1
24098	A_64_P042811	-2.322	0.134	3.2E-10	-2.463	0.134	1.6E-10		ENSRNOG00000031780	MT	1
24283	A_64_P138744	1.607	0.159	2.0E-07	1.501	0.159	4.3E-07		ENSRNOG00000031780	MT	1
24791	A_64_P142146	1.003	0.124	2.4E-06	1.858	0.124	2.0E-09		ENSRNOG00000031780	MT	1
25880	A_64_P006473	-1.765	0.177	2.3E-07	-1.094	0.177	3.7E-05		ENSRNOG00000031780	MT	1
26581	A_64_P135939	2.288	0.273	1.6E-06	2.001	0.273	6.6E-06		ENSRNOG00000031780	MT	1
28018	A_64_P101600	-1.533	0.182	1.5E-06	-1.022	0.182	9.4E-05		ENSRNOG00000031780	MT	1
29293	A_64_P029190	1.132	0.084	7.4E-09	1.222	0.084	3.0E-09		ENSRNOG00000031780	MT	1
8786	A_64_P062462	0.841	0.105	2.7E-06	1.375	0.105	1.0E-08	Pir	ENSRNOG00000003674	X	31,968,152
19288	A_64_P128433	-1.097	0.104	1.2E-07	-1.107	0.104	1.1E-07	Ctps2	ENSRNOG00000004257	X	33,522,836
20840	A_64_P037631	2.887	0.197	2.5E-09	2.709	0.197	5.4E-09	Ar	ENSRNOG00000005639	X	67,656,253
15663	A_64_P048852	-1.298	0.115	5.4E-08	-1.176	0.115	1.7E-07		ENSRNOG00000006459	X	120,313,696
25430	A_64_P008527	-1.469	0.189	6.8E-05	-1.430	0.189	8.2E-05	Wdr44	ENSRNOG00000029068	X	120,860,178
3086	A_42_P768355	2.866	0.179	2.9E-08	2.715	0.179	4.7E-08	no transcript			
4576	A_64_P011301	1.171	0.081	3.0E-09	1.314	0.081	7.4E-10	Ugt2b17 (Ugt2b5)			
5530	A_42_P538334	0.639	0.102	3.2E-05	0.662	0.102	2.3E-05	Snhg8			
6876	A_64_P102739	-1.997	0.124	8.5E-10	-0.990	0.124	2.8E-06	LOC690340			
9344	A_64_P029333	-0.764	0.097	3.0E-06	-0.669	0.097	1.2E-05	Acaa1b			
12669	A_64_P115157	-0.854	0.089	3.7E-07	-0.853	0.089	3.7E-07	Ces1e			
14531	A_64_P098926	1.664	0.126	9.1E-09	1.052	0.126	1.7E-06	no transcript			
15451	A_64_P004422	-0.864	0.124	1.1E-05	-0.878	0.124	9.2E-06	LOC687708			
23957	A_64_P139208	-1.808	0.168	9.9E-08	-1.525	0.168	6.8E-07	RT1-N1 (part)			
25709	A_64_P011719	-1.519	0.153	2.5E-07	-1.198	0.153	3.3E-06	**Chmp1b**	N/A	18	62,923,620
26531	A_64_P063641	-1.526	0.199	1.2E-05	-1.597	0.263	9.3E-05	LOC501251			
27091	A_64_P025432	1.945	0.274	9.4E-06	3.546	0.274	1.1E-08	LOC498829			

Disease Models & Mechanisms • Supplementary information

28226	A_64_P029191	0.770	0.095	2.4E-06	1.021	0.095	1.0E-07 no transcript

Genes are regarded as significant for “shared” differential expression when they show $P<1\times10^{-4}$ reproducibly in two types of comparison, ie, SHR/Izm versus WKY/Izm and SHRSP/Izm versus WKY/Izm, with a concordant direction of differential expression.

Table S3F. A list of significant transcripts for non-shared differential expression in the heart.

no	ProbeName	SHR/lzm (vs WKY/lzm)			SHRSP/lzm (vs WKY/lzm)			GeneName	Ensembl_rat	Chr	Transcription_ start_site (Rnor_6.0)
		Fold change (log2-scale)	SE (log2-scale)	P-value	Fold change (log2-scale)	SE (log2-scale)	P-value				
23135	A_64_P147549	-0.114	0.095	2.4E-01	-0.389	0.047	8.1E-10	Ust	ENSRNOG00000016381	1	2,627,747
4209	A_42_P547213	0.125	0.080	1.2E-01	0.415	0.040	1.9E-12	Fbxo30	ENSRNOG00000014852	1	5,366,379
14654	A_44_P255294	0.064	0.067	3.5E-01	0.261	0.034	3.6E-09	Hbs1l	ENSRNOG00000014531	1	16,819,170
14291	A_44_P191715	-0.183	0.115	1.2E-01	-0.684	0.057	4.6E-14	Trdn	ENSRNOG00000012609	1	25,839,198
27952	A_64_P086235	0.281	0.105	1.1E-02	-0.669	0.053	6.6E-15	Irx1	ENSRNOG00000033609	1	33,910,912
17452	A_64_P042302	-0.094	0.126	4.6E-01	-0.523	0.063	7.1E-10	Ube2ql1	ENSRNOG00000034075	1	36,185,916
24774	A_44_P794669	0.091	0.086	3.0E-01	-0.334	0.043	3.5E-09	Afdn (Mllt4)	ENSRNOG00000023753	1	53,802,658
26771	A_64_P032991	0.024	0.066	7.2E-01	-0.255	0.033	3.3E-09	none	ENSRNOG00000019623	1	65,597,899
21540	A_64_P001475	-0.067	0.060	2.7E-01	-0.369	0.030	1.8E-14	Irf2bp1	ENSRNOG00000014132	1	79,899,155
9254	A_42_P769597	-0.038	0.085	6.6E-01	-0.330	0.043	3.5E-09	Qpctl	ENSRNOG00000015413	1	80,056,574
1817	A_42_P773606	-0.003	0.185	9.9E-01	0.718	0.093	3.3E-09	Ppm1n (RGD156	ENSRNOG00000059266	1	80,191,618
9837	A_42_P496411	-0.146	0.077	6.4E-02	0.957	0.038	2.0E-24	Trappc6a	ENSRNOG00000017468	1	80,417,310
17625	A_64_P047432	-0.130	0.085	1.3E-01	1.126	0.042	2.7E-25	Trappc6a	ENSRNOG00000017468	1	80,417,310
17009	A_64_P010196	0.085	0.102	4.1E-01	0.697	0.051	6.6E-16	Gemin7	ENSRNOG00000049772	1	80,483,487
13331	A_44_P575055	0.049	0.121	6.9E-01	0.622	0.061	3.0E-12	Zfp61	ENSRNOG00000019416	1	81,144,024
28240	A_44_P897008	0.008	0.073	9.1E-01	-0.379	0.036	2.1E-12	Irgq	ENSRNOG00000046500	1	81,395,841
13176	A_64_P003994	0.126	0.081	1.3E-01	0.321	0.040	1.9E-09	Rps19	ENSRNOG00000037897	1	81,750,928
28172	A_44_P318448	0.171	0.103	1.1E-01	0.458	0.052	1.3E-10	Megf8	ENSRNOG00000052687	1	82,185,034
24505	A_43_P15665	0.038	0.133	7.8E-01	-0.601	0.067	8.2E-11	Bckdha	ENSRNOG00000020607	1	82,452,281
8860	A_44_P520943	-0.096	0.095	3.2E-01	-0.490	0.048	2.6E-12	Exosc5	ENSRNOG00000020635	1	82,452,469
11718	A_64_P034456	0.131	0.131	3.3E-01	0.496	0.066	6.1E-09	Adck4	ENSRNOG00000020848	1	84,044,551
10912	A_64_P086519	-0.069	0.053	2.0E-01	0.274	0.026	2.2E-12	Blvrb	ENSRNOG00000024410	1	84,256,159
24874	A_64_P071840	-0.066	0.110	5.5E-01	0.478	0.055	2.1E-10	Blvrb	ENSRNOG00000024410	1	84,256,159
23715	A_64_P024297	-0.017	0.098	8.6E-01	0.486	0.049	8.2E-12	Gmfg	ENSRNOG00000019838	1	85,317,968
16474	A_64_P068858	0.108	0.067	1.2E-01	2.881	0.034	2.3E-43	Rps16	ENSRNOG00000019578	1	85,408,444
25138	A_64_P133421	0.257	0.147	9.0E-02	-0.777	0.074	1.4E-12	Eid2b	ENSRNOG00000045545	1	85,510,114
17605	A_44_P186860	0.073	0.131	5.8E-01	0.495	0.065	6.1E-09	Nfkbib	ENSRNOG00000020063	1	86,948,845
22916	A_44_P997962	0.170	0.135	2.1E-01	-0.528	0.067	2.6E-09	Fam98c (RGD13	ENSRNOG00000024036	1	88,098,785

6002	A_64_P139457	-0.078	0.090	3.9E-01	-1.039	0.045	2.7E-23	LOC499124	ENSRNOG00000030932	1	88,361,712
8683	A_42_P644209	-0.237	0.142	1.1E-01	1.334	0.066	5.9E-21	LOC499124	ENSRNOG00000030932	1	88,361,712
15811	A_64_P110309	0.154	0.091	1.0E-01	-2.533	0.046	1.3E-36	none_LOC68750	ENSRNOG00000047364	1	88,620,317
22281	A_64_P126065	-0.041	0.093	6.6E-01	-0.713	0.047	2.2E-17	Hcst	ENSRNOG00000020849	1	88,881,460
26746	A_64_P083600	0.182	0.134	1.9E-01	0.535	0.067	1.8E-09	Fxyd5	ENSRNOG00000021062	1	89,474,252
16896	A_64_P025098	0.087	0.076	2.6E-01	0.390	0.038	2.8E-12	Cebpg	ENSRNOG00000021144	1	91,296,656
18861	A_64_P006072	0.137	0.098	1.7E-01	-0.694	0.049	2.7E-16	Znf536	ENSRNOG00000014163	1	93,949,187
3330	A_44_P474037	0.250	0.158	1.2E-01	0.594	0.079	6.8E-09	Igf1r	ENSRNOG00000014187	1	128,924,966
1418	A_42_P497225	0.137	0.111	2.2E-01	0.433	0.055	2.6E-09	Mfge8	ENSRNOG00000017510	1	140,860,882
29968	A_64_P131086	0.224	0.126	8.4E-02	0.614	0.063	1.1E-11	Rab30	ENSRNOG00000010224	1	157,573,324
26276	A_44_P344181	0.177	0.114	1.3E-01	0.765	0.057	1.5E-15	Map6	ENSRNOG00000027204	1	164,225,934
14093	A_42_P788740	0.133	0.107	2.2E-01	0.621	0.054	1.0E-13	Folr2	ENSRNOG00000019890	1	166,919,302
28650	A_44_P177931	0.127	0.080	1.2E-01	0.401	0.040	5.9E-12	Anapc15 (RGD13	ENSRNOG00000019936	1	166,983,175
821	A_64_P117345	0.368	0.190	6.1E-02	1.105	0.090	3.9E-14	Arntl	ENSRNOG00000014448	1	178,039,063
22652	A_64_P001922	0.109	0.074	1.5E-01	0.298	0.037	1.5E-09	Vps35l (LOC361(	ENSRNOG00000016063	1	188,448,478
30295	A_44_P337300	0.033	0.109	7.6E-01	0.431	0.054	1.9E-09	Il4ra	ENSRNOG00000015441	1	196,942,364
7398	A_42_P495691	0.079	0.091	3.9E-01	0.345	0.045	5.0E-09	Bcl7c	ENSRNOG00000018916	1	199,159,125
22149	A_64_P056569	-0.106	0.080	1.9E-01	-0.444	0.040	2.9E-13	Fus	ENSRNOG00000023360	1	199,412,834
10630	A_64_P088990	-0.078	0.072	2.8E-01	-0.321	0.036	1.0E-10	Ctbp2	ENSRNOG00000017326	1	205,030,567
18089	A_44_P271720	0.046	0.083	5.8E-01	0.346	0.042	6.4E-10	Cd151	ENSRNOG00000046094	1	214,446,659
28707	A_64_P066601	0.057	0.099	5.6E-01	-0.391	0.049	2.0E-09	Rhod	ENSRNOG00000019220	1	219,682,287
21794	A_42_P837214	-0.268	0.085	3.4E-03	0.416	0.043	1.2E-11	Mgmt	ENSRNOG00000016038	1	209,237,233
5502	A_64_P087105	0.080	0.097	4.2E-01	0.425	0.048	1.7E-10	Gpr137	ENSRNOG00000021145	1	222,197,252
27738	A_64_P159519	0.294	0.076	4.0E-04	-0.288	0.038	4.8E-09	Pla2g16	ENSRNOG00000021206	1	222,844,144
30101	A_44_P896040	-0.916	0.081	2.0E-13	0.178	0.041	9.3E-05	Cybasc3	ENSRNOG00000020702	1	226,634,009
7124	A_64_P062545	-0.454	0.088	8.8E-06	0.626	0.044	2.0E-16	Tmem132a	ENSRNOG00000021338	1	226,924,244
2519	A_64_P065422	0.012	0.185	9.5E-01	-1.519	0.103	6.5E-16	none	ENSRNOG00000047349	1	227,670,159
1377	A_42_P711568	-0.064	0.060	2.9E-01	-0.222	0.030	8.6E-09	Ranbp6	ENSRNOG00000053859	1	247,985,497
13746	A_44_P536613	0.543	0.288	6.7E-02	1.157	0.144	1.4E-09	Ch25h	ENSRNOG00000019141	1	252,808,380
11472	A_44_P198112	0.096	0.087	2.8E-01	0.340	0.043	2.6E-09	Marchf5	ENSRNOG00000017396	1	255,842,783
20886	A_64_P088575	0.308	0.172	8.2E-02	1.901	0.095	5.1E-20	Dhdpsl	ENSRNOG00000029501	1	261,291,870

Disease Models & Mechanisms • Supplementary information

25963	A_64_P129182	-0.023	0.196	9.1E-01	-0.755	0.098	3.7E-09	Npm3	ENSRNOG00000017622	1	265,506,046
17232	A_43_P12332	-0.150	0.076	5.5E-02	-0.300	0.038	2.2E-09	Nolc1	ENSRNOG00000018704	1	265,829,108
23837	A_42_P658264	0.122	0.069	8.5E-02	0.433	0.035	9.9E-15	Nfkb2	ENSRNOG00000019311	1	266,053,002
23188	A_44_P1002173	-0.057	0.082	4.9E-01	0.480	0.041	8.2E-14	Cuedc2	ENSRNOG00000019574	1	266,086,299
688	A_42_P473812	0.003	0.110	9.8E-01	0.491	0.055	1.0E-10	Rbm20	ENSRNOG00000014705	1	274,391,932
17131	A_64_P088836	0.464	0.101	4.8E-05	-0.957	0.050	2.0E-20	Gpr98	ENSRNOG00000016306	2	8,939,686
19833	A_64_P158035	0.474	0.123	4.5E-04	-0.887	0.061	1.4E-16	RGD1562101	ENSRNOG00000016306	2	8,939,686
22758	A_44_P377432	0.382	0.098	4.0E-04	-0.880	0.049	1.3E-19	Gpr98	ENSRNOG00000016306	2	8,939,686
752	A_44_P158798	0.226	0.175	2.1E-01	0.749	0.088	3.3E-10	Vcan	ENSRNOG00000029212	2	18,565,842
16284	A_44_P236418	0.230	0.120	6.3E-02	0.465	0.060	3.1E-09	Gfm2	ENSRNOG00000025285	2	27,949,208
3372	A_43_P10200	0.202	0.110	7.5E-02	-0.445	0.055	1.3E-09	Slc30a5	ENSRNOG00000018746	2	30,846,149
23871	A_64_P104820	0.351	0.192	7.5E-02	0.719	0.096	6.9E-09	Plpp1	ENSRNOG00000009980	2	44,664,124
24142	A_44_P122386	0.121	0.073	1.1E-01	0.511	0.037	3.9E-16	Hspb3	ENSRNOG00000010992	2	45,518,502
3710	A_44_P175584	0.355	0.183	6.1E-02	0.680	0.092	9.0E-09	RGD1561520	ENSRNOG00000026899	2	52,171,016
8809	A_44_P256677	0.421	0.138	4.3E-03	-0.561	0.069	1.1E-09	RGD1308448	ENSRNOG00000014550	2	54,191,538
26171	A_64_P099923	0.129	0.079	1.1E-01	0.453	0.040	1.5E-13	Dap	ENSRNOG00000010747	2	84,275,884
3010	A_42_P581863	0.064	0.071	3.7E-01	0.371	0.036	2.0E-12	Pfn2	ENSRNOG00000017427	2	147,959,567
23875	A_43_P12315	-0.240	0.097	1.8E-02	0.366	0.048	5.6E-09	P2ry12	ENSRNOG00000013902	2	149,444,548
9313	A_43_P11484	0.480	0.154	3.5E-03	-0.586	0.077	4.9E-09	Mme	ENSRNOG00000009514	2	153,803,349
14592	A_64_P104688	0.004	0.065	9.5E-01	0.254	0.033	2.7E-09	Cks1b	ENSRNOG00000042561	2	188,745,144
62	A_42_P548763	0.143	0.105	1.8E-01	0.461	0.052	1.6E-10	Slc39a1	ENSRNOG00000059463	2	189,609,800
998	A_43_P15245	-0.142	0.088	1.2E-01	-0.437	0.044	8.1E-12	Npr1	ENSRNOG00000014684	2	189,856,090
27575	A_44_P355928	0.117	0.112	3.0E-01	0.435	0.056	3.0E-09	Ctss	ENSRNOG00000021157	2	197,655,786
29124	A_44_P836445	-0.071	0.071	3.2E-01	-0.310	0.036	2.0E-10	Aph1a	ENSRNOG00000023816	2	198,006,322
21603	A_44_P354806	-0.085	0.105	4.2E-01	0.389	0.053	9.6E-09	Vps45	ENSRNOG00000021173	2	198,184,739
28930	A_64_P043871	0.669	0.075	1.3E-10	0.041	0.038	2.8E-01	Polr3gl	ENSRNOG00000021209	2	198,706,428
5164	A_64_P078592	-0.108	0.101	2.9E-01	0.467	0.051	5.1E-11	Man1a2	ENSRNOG00000015226	2	203,043,847
3071	A_64_P005243	-0.047	0.098	6.4E-01	-0.503	0.049	2.5E-12	Tspan2	ENSRNOG00000023338	2	205,160,405
5675	A_44_P838096	-0.147	0.088	1.0E-01	-0.665	0.044	3.8E-17	Rsbn1	ENSRNOG00000019671	2	206,392,200
15672	A_44_P328008	-0.124	0.142	3.9E-01	-0.703	0.071	7.4E-12	Alx3	ENSRNOG00000018290	2	210,381,829
12171	A_44_P771864	0.103	0.102	3.2E-01	-0.406	0.051	1.7E-09	Cdc14a	ENSRNOG00000014515	2	219,458,271

Disease Models & Mechanisms • Supplementary information

28932	A_44_P478176	-0.104	0.086	2.4E-01	-0.324	0.043	6.6E-09	Lrrc39	ENSRNOG00000015117	2	219,598,162
24834	A_64_P086083	0.048	0.089	5.9E-01	-0.330	0.044	9.3E-09	Slc35a3	ENSRNOG00000061832	2	219,741,886
23100	A_44_P1011780	0.205	0.085	2.1E-02	-0.369	0.043	2.3E-10	Pla2g12a	ENSRNOG00000057470	2	235,311,719
3199	A_44_P281352	0.249	0.130	6.4E-02	0.533	0.065	9.1E-10	Dnajb4	ENSRNOG00000013011	2	257,418,425
28745	A_43_P15529	0.054	0.116	6.5E-01	0.559	0.058	1.5E-11	Ptgds	ENSRNOG00000015550	3	2,689,084
22299	A_64_P083610	0.245	0.165	1.5E-01	0.679	0.082	7.9E-10	Dbh	ENSRNOG00000006641	3	5,709,236
25202	A_42_P472520	0.088	0.111	4.3E-01	0.598	0.055	7.4E-13	RGD1561113	ENSRNOG00000022681	3	11,410,732
12233	A_44_P474559	0.214	0.108	5.5E-02	0.569	0.054	1.5E-12	Eng	ENSRNOG00000050190	3	11,679,530
19913	A_44_P102477	-0.368	0.190	6.1E-02	-0.947	0.097	1.5E-11	Morn5	ENSRNOG00000026111	3	15,379,109
11083	A_64_P099011	-0.021	0.091	8.2E-01	-0.686	0.046	3.9E-17	Scn1a	ENSRNOG00000053122	3	52,447,622
9452	A_64_P032018	-0.052	0.348	8.8E-01	1.113	0.102	5.6E-12	none	ENSRNOG00000009382	3	57,270,036
25648	A_64_P101921	0.154	0.092	1.0E-01	0.410	0.046	1.3E-10	Dync1i2	ENSRNOG00000009781	3	57,817,693
23651	A_64_P039081	0.110	0.058	6.5E-02	-1.663	0.029	3.7E-37	Eno1	ENSRNOG00000028543	3	59,408,546
14850	A_64_P101032	0.099	0.076	2.0E-01	0.337	0.038	1.5E-10	none	ENSRNOG00000001510	3	59,587,569
21396	A_64_P071585	0.138	0.080	9.3E-02	0.336	0.040	5.2E-10	Fkbp7	ENSRNOG00000011758	3	63,535,991
20525	A_64_P038827	0.117	0.073	1.2E-01	0.303	0.037	7.5E-10	Jmjd7	ENSRNOG00000050072	3	111,818,150
27910	A_64_P158384	-0.076	0.119	5.3E-01	-0.526	0.059	1.3E-10	Fam227b (RGD1	ENSRNOG00000037124	3	118,427,851
30000	A_64_P144492	-0.195	0.110	8.4E-02	-0.547	0.055	6.1E-12	Mall	ENSRNOG00000015599	3	120,306,551
2596	A_64_P004292	0.115	0.098	2.5E-01	0.369	0.049	6.3E-09	Ttl	ENSRNOG00000018205	3	121,596,791
10669	A_44_P792628	0.033	0.097	7.4E-01	0.368	0.048	5.0E-09	Spef1 (RGD1311	ENSRNOG00000021247	3	123,718,432
12403	A_44_P398033	0.719	0.081	1.4E-10	0.080	0.041	5.7E-02	Prnp	ENSRNOG00000021259	3	124,515,978
6630	A_42_P471608	-0.174	0.087	5.4E-02	-0.341	0.044	2.9E-09	Kif16b	ENSRNOG00000004951	3	136,936,674
8939	A_64_P021980	-0.186	0.182	3.1E-01	-0.694	0.091	5.0E-09	Sstr4	ENSRNOG00000004641	3	142,739,781
12022	A_64_P132971	-0.189	0.221	4.0E-01	1.076	0.123	3.7E-10	Dusp15	ENSRNOG00000008534	3	148,428,494
4481	A_64_P164610	0.051	0.065	4.4E-01	-0.807	0.033	2.7E-24	Tti1 (RGD15625ε	ENSRNOG00000012880	3	154,490,851
17021	A_64_P023008	-0.022	0.212	9.2E-01	1.048	0.106	8.4E-12	none_SNORA71	ENSRNOG00000056385	3	154,846,116
927	A_64_P091016	0.197	0.173	2.6E-01	0.689	0.091	7.3E-09	Ppp1r16b	ENSRNOG00000015614	3	155,160,481
17071	A_64_P134303	-0.141	0.108	2.0E-01	-0.542	0.054	5.6E-12	Zhx3	ENSRNOG00000027988	3	156,777,999
3911	A_42_P597242	-0.017	0.067	8.0E-01	0.254	0.033	5.7E-09	RGD1303142	ENSRNOG00000008297	3	159,802,952
17113	A_64_P139590	-0.208	0.107	5.9E-02	1.393	0.053	4.5E-25	Slc35c2	ENSRNOG00000018649	3	161,812,291
15238	A_64_P033979	-0.173	0.153	2.6E-01	1.136	0.076	5.5E-17	Cdh22	ENSRNOG00000018557	3	161,917,285

Disease Models & Mechanisms • Supplementary information

861	A_42_P485932	0.056	0.088	5.3E-01	0.379	0.044	2.8E-10	Sulf2	ENSRNOG00000006052	3	162,872,831
4982	A_64_P155186	-0.112	0.094	2.4E-01	0.402	0.047	3.0E-10	Aurka	ENSRNOG00000004479	3	170,378,210
20646	A_64_P101847	0.012	0.049	8.1E-01	-0.193	0.025	3.1E-09	Ythdf1	ENSRNOG00000027015	3	176,431,423
6319	A_64_P028431	-0.102	0.136	4.6E-01	-0.519	0.068	4.5E-09	Znf512b	ENSRNOG00000015558	3	177,089,199
24460	A_44_P143886	0.103	0.138	4.6E-01	0.627	0.069	7.4E-11	Insig1	ENSRNOG00000006859	4	342,302
23936	A_44_P294359	-0.083	0.100	4.1E-01	-0.405	0.050	1.2E-09	none	ENSRNOG00000040214	4	8,256,611
3918	A_42_P604399	0.149	0.077	6.1E-02	0.346	0.039	9.8E-11	Cyp51	ENSRNOG00000007234	4	27,175,243
26944	A_43_P15431	-0.898	0.074	2.9E-14	-0.050	0.037	1.9E-01	Mterf	ENSRNOG00000007899	4	27,365,376
24172	A_64_P025464	0.107	0.071	1.4E-01	0.280	0.036	2.5E-09	Shfm1	ENSRNOG00000010420	4	32,087,600
15563	A_64_P106533	0.018	0.129	8.9E-01	-0.673	0.067	8.8E-12	Thsd7a	ENSRNOG00000030151	4	39,102,807
19396	A_64_P058923	-0.931	0.114	1.1E-09	-0.091	0.057	1.2E-01	Tes	ENSRNOG00000051952	4	44,321,883
762	A_64_P151146	0.233	0.169	1.8E-01	-1.483	0.085	3.1E-19	Iqub	ENSRNOG00000021729	4	51,586,881
4359	A_44_P326727	-0.077	0.111	4.9E-01	0.510	0.055	5.1E-11	Snd1	ENSRNOG00000031173	4	55,772,377
7030	A_44_P426107	0.123	0.108	2.6E-01	0.466	0.054	2.8E-10	Akr1b8	ENSRNOG00000009734	4	61,772,064
10688	A_44_P492808	-0.008	0.098	9.3E-01	0.476	0.049	1.4E-11	Agk	ENSRNOG00000011509	4	68,483,320
28386	A_64_P153338	-0.005	0.104	9.6E-01	0.460	0.052	1.3E-10	Agk	ENSRNOG00000011509	4	68,483,320
14746	A_44_P638290	-0.156	0.078	5.3E-02	-0.316	0.039	1.1E-09	Znf467	ENSRNOG00000007707	4	78,074,906
5573	A_42_P640700	0.156	0.101	1.3E-01	0.393	0.051	3.3E-09	Tmem176a	ENSRNOG00000023708	4	78,458,625
1042	A_64_P079478	0.053	0.100	6.0E-01	0.713	0.050	1.8E-16	Jazf1	ENSRNOG00000027026	4	83,137,527
20794	A_64_P160437	0.076	0.091	4.1E-01	0.705	0.045	1.5E-17	Jazf1	ENSRNOG00000027026	4	83,137,527
740	A_44_P242614	0.166	0.124	1.9E-01	0.559	0.062	8.4E-11	Znrf2	ENSRNOG00000049057	4	85,009,350
6838	A_42_P507035	0.048	0.079	5.5E-01	-0.397	0.039	5.1E-12	Nod1	ENSRNOG00000010629	4	85,174,951
27937	A_64_P084188	-0.166	0.116	1.6E-01	-0.906	0.058	1.1E-17	Nod1	ENSRNOG00000010629	4	85,174,951
23985	A_44_P216395	-0.156	0.093	1.0E-01	-0.439	0.047	2.9E-11	Gadd45a	ENSRNOG00000005615	4	97,784,842
859	A_64_P145166	0.114	0.066	9.4E-02	0.489	0.033	6.5E-17	Ggcx	ENSRNOG00000012975	4	100,277,391
6104	A_44_P1032296	0.057	0.104	5.9E-01	0.476	0.052	6.0E-11	Capg	ENSRNOG00000013668	4	100,407,658
4378	A_64_P029142	0.208	0.150	1.7E-01	0.718	0.075	1.8E-11	Loxl3	ENSRNOG00000061373	4	113,866,804
26314	A_44_P465660	0.138	0.093	1.5E-01	0.385	0.047	7.4E-10	Anxa4	ENSRNOG00000018159	4	118,595,580
5621	A_64_P110654	0.087	0.108	4.3E-01	0.567	0.054	1.7E-12	Slc25a26	ENSRNOG00000012831	4	126,522,326
22626	A_43_P12768	-0.871	0.090	1.4E-11	-0.002	0.045	9.7E-01	Bhlhe40	ENSRNOG00000007152	4	140,703,619
20302	A_64_P114369	0.117	0.075	1.3E-01	0.289	0.037	3.4E-09	Thumpd3	ENSRNOG00000006941	4	144,985,880

3107	A_44_P541456	-0.273	0.103	1.2E-02	0.622	0.052	3.3E-14	Arpc4	ENSRNOG00000008994	4	145,330,457
9721	A_64_P133343	0.031	0.089	7.3E-01	0.397	0.045	1.2E-10	LOC679934	ENSRNOG00000010184	4	145,559,206
21876	A_64_P037666	0.025	0.167	8.8E-01	-0.726	0.084	2.3E-10	Iqsec3	ENSRNOG00000014083	4	154,044,493
11748	A_64_P039482	0.007	0.176	9.7E-01	0.668	0.088	5.6E-09	Cd4	ENSRNOG00000016294	4	157,408,176
10235	A_64_P034244	0.631	0.434	1.5E-01	2.017	0.177	3.5E-13	Olr1	ENSRNOG00000056219	4	163,261,958
26036	A_44_P807058	0.148	0.122	2.3E-01	0.478	0.061	2.4E-09	Emp1	ENSRNOG00000008676	4	169,147,243
13001	A_44_P409860	0.169	0.111	1.4E-01	0.963	0.055	3.6E-19	none	ENSRNOG00000048735	4	170,092,848
15950	A_64_P039911	0.168	0.100	1.0E-01	1.064	0.050	5.2E-22	none	ENSRNOG00000045840	4	170,763,916
25290	A_64_P048941	-0.418	0.207	5.2E-02	-1.203	0.104	9.8E-14	Art4	ENSRNOG00000005670	4	170,841,187
29087	A_44_P283941	0.094	0.083	2.7E-01	0.526	0.042	7.0E-15	Arhgdib	ENSRNOG00000005809	4	170,932,618
13310	A_44_P884766	0.214	0.212	3.2E-01	-1.020	0.106	1.7E-11	Vxn (RGD15618	ENSRNOG00000021663	5	9,381,214
17621	A_44_P412710	-0.099	0.108	3.7E-01	-0.487	0.054	8.3E-11	Adhfe1	ENSRNOG00000007069	5	9,429,859
11193	A_64_P136641	0.216	0.173	2.2E-01	-1.496	0.086	4.4E-19	Rp1	ENSRNOG00000008807	5	15,043,955
17422	A_44_P159305	-0.155	0.104	1.5E-01	-0.402	0.052	3.9E-09	Klhl32	ENSRNOG00000007441	5	39,215,102
11969	A_44_P195291	0.077	0.091	4.0E-01	-0.396	0.046	2.1E-10	Ccdc107	ENSRNOG00000017253	5	58,995,249
20055	A_44_P323106	0.118	0.126	3.5E-01	-0.706	0.063	2.4E-13	Abca1	ENSRNOG00000018126	5	69,983,015
4055	A_42_P504549	-0.020	0.083	8.1E-01	0.736	0.041	1.7E-19	Tmem38b	ENSRNOG00000028063	5	70,639,156
19775	A_44_P963812	0.314	0.119	1.2E-02	-0.481	0.059	1.2E-09	Ctnnal1	ENSRNOG00000010593	5	73,621,070
3541	A_64_P130627	0.243	0.152	1.2E-01	0.672	0.076	1.4E-10	LOC298139	ENSRNOG00000045733	5	90,338,795
7988	A_42_P623839	-0.003	0.138	9.8E-01	0.627	0.069	7.0E-11	Ttc39b	ENSRNOG00000042603	5	101,405,656
5062	A_44_P134672	0.078	0.076	3.1E-01	0.330	0.038	2.3E-10	Snapc3	ENSRNOG00000010825	5	101,526,551
7815	A_42_P498273	0.153	0.109	1.7E-01	0.472	0.054	2.2E-10	Pgm1	ENSRNOG00000009889	5	118,743,632
4744	A_44_P1015839	0.107	0.140	4.5E-01	-0.651	0.070	4.3E-11	Mroh7 (RGD156	ENSRNOG00000058870	5	126,323,799
7726	A_64_P112295	-0.066	0.118	5.8E-01	-0.605	0.059	3.1E-12	Slc1a7	ENSRNOG00000011644	5	127,571,114
20004	A_44_P217189	-0.125	0.082	1.4E-01	-0.488	0.041	4.7E-14	Echdc2	ENSRNOG00000029333	5	127,770,570
16154	A_64_P100684	0.015	0.073	8.3E-01	-0.320	0.036	1.7E-10	Trabd2b	ENSRNOG00000029141	5	133,221,139
1850	A_42_P570919	0.033	0.062	6.0E-01	0.276	0.031	1.2E-10	Akr1a1	ENSRNOG00000016727	5	135,498,822
11409	A_64_P044221	-0.108	0.067	1.1E-01	-0.270	0.033	1.4E-09	Hyi (RGD156141	ENSRNOG00000037518	5	137,189,473
30286	A_64_P148183	-0.016	0.094	8.7E-01	0.409	0.047	2.1E-10	LOC679583	ENSRNOG00000062077	5	137,350,525
18178	A_64_P161655	0.136	0.072	6.6E-02	0.303	0.036	4.3E-10	Ppt1	ENSRNOG00000012616	5	140,538,260
25379	A_44_P501752	0.044	0.076	5.7E-01	0.320	0.038	4.2E-10	RGD1559909	ENSRNOG00000042421	5	142,845,116

Disease Models & Mechanisms • Supplementary information

24535	A_64_P137198	-0.077	0.057	1.9E-01	-0.221	0.029	3.9E-09	Trappc3	ENSRNOG00000010550	5	144,281,614
17496	A_43_P11236	0.169	0.088	6.2E-02	0.364	0.044	7.1E-10	Laptm5	ENSRNOG00000011054	5	149,047,681
27859	A_64_P080248	0.038	0.086	6.6E-01	0.387	0.043	9.0E-11	Dhdds	ENSRNOG00000014665	5	152,227,677
24569	A_42_P534172	-0.465	0.223	4.4E-02	0.981	0.111	1.6E-10	Cd52	ENSRNOG00000015403	5	152,324,469
22414	A_44_P466209	0.121	0.080	1.4E-01	0.317	0.040	2.2E-09	Tceb3	ENSRNOG00000010902	5	154,363,329
27126	A_44_P359032	-0.046	0.093	6.3E-01	-0.411	0.046	1.3E-10	Pink1	ENSRNOG00000015385	5	156,689,258
26599	A_64_P157371	0.116	0.192	5.5E-01	0.710	0.096	9.2E-09	Pdpn	ENSRNOG00000014961	5	161,981,441
11847	A_44_P306355	-0.023	0.080	7.7E-01	0.360	0.040	8.2E-11	Plod1	ENSRNOG00000007763	5	164,747,083
272	A_44_P318661	0.085	0.124	5.0E-01	-0.886	0.062	1.7E-16	Mthfr	ENSRNOG00000008553	5	164,845,925
8266	A_64_P114713	-0.142	0.134	3.0E-01	-3.658	0.067	2.2E-36	Agtrap	ENSRNOG00000008619	5	164,898,420
892	A_44_P321510	0.064	0.152	6.8E-01	0.573	0.076	6.3E-09	Slc25a33	ENSRNOG00000016949	5	166,726,794
21282	A_64_P093037	0.029	0.090	7.5E-01	-1.846	0.045	7.5E-32	Eno1	ENSRNOG00000017895	5	167,288,223
25873	A_64_P067525	-0.167	0.088	6.5E-02	-1.533	0.044	1.8E-29	Eno1	ENSRNOG00000017895	5	167,288,223
24228	A_64_P068252	0.183	0.129	1.6E-01	0.840	0.064	2.9E-15	Fam179a	ENSRNOG00000026344	6	23,404,368
6402	A_43_P17213	0.121	0.132	3.7E-01	-0.505	0.066	4.6E-09	Abhd1	ENSRNOG00000025689	6	26,792,808
6427	A_64_P037020	-0.070	0.065	2.9E-01	-0.274	0.033	5.1E-10	Seli	ENSRNOG00000059295	6	27,487,284
1354	A_42_P575104	-0.220	0.138	1.2E-01	-0.515	0.069	7.9E-09	Sostdc1	ENSRNOG00000005770	6	55,812,747
14875	A_44_P147560	0.121	0.080	1.4E-01	0.326	0.040	1.1E-09	Cfl2	ENSRNOG00000045892	6	75,763,185
8871	A_64_P019638	-0.131	0.087	1.4E-01	-0.340	0.043	2.7E-09	Prkch	ENSRNOG00000004873	6	96,479,430
29048	A_44_P139742	0.135	0.073	7.3E-02	0.361	0.036	7.7E-12	Npc2	ENSRNOG00000012062	6	108,488,330
6398	A_64_P120640	-0.385	0.229	1.0E-01	-0.926	0.117	2.3E-09	Pgf	ENSRNOG00000005650	6	109,004,598
5025	A_43_P14012	-0.047	0.052	3.7E-01	-0.197	0.026	6.8E-09	Mlh3	ENSRNOG00000006699	6	109,095,557
7582	A_42_P759524	0.013	0.202	9.5E-01	-0.762	0.101	6.0E-09	Serpina5	ENSRNOG00000009855	6	127,766,470
7786	A_44_P111337	0.192	0.113	9.8E-02	0.494	0.056	1.8E-10	Traf3	ENSRNOG00000008145	6	135,610,743
12539	A_64_P015739	-0.007	0.100	9.5E-01	0.399	0.050	1.8E-09	Mta1	ENSRNOG00000004711	6	137,924,293
5400	A_64_P082217	-0.379	0.239	1.2E-01	-0.902	0.119	6.0E-09	Dnah11	ENSRNOG00000005451	6	146,099,053
2313	A_64_P049027	-0.125	0.073	9.6E-02	-0.295	0.037	1.3E-09	Sppl2b	ENSRNOG00000057881	7	11,724,843
6397	A_64_P052740	-0.146	0.148	3.3E-01	-0.570	0.074	3.6E-09	Igf1	ENSRNOG00000004517	7	28,412,198
14916	A_44_P366723	-0.252	0.128	5.6E-02	-0.608	0.064	2.0E-11	Igf1	ENSRNOG00000004517	7	28,412,198
19762	A_64_P076900	-0.002	0.078	9.8E-01	0.331	0.039	4.1E-10	Nedd1	ENSRNOG00000004011	7	33,584,564
9651	A_44_P993671	-0.058	0.209	7.8E-01	-0.943	0.104	8.4E-11	Epyc	ENSRNOG00000004717	7	38,897,278

Disease Models & Mechanisms • Supplementary information

15370	A_44_P874636	0.020	0.077	8.0E-01	-0.338	0.038	1.7E-10	Cpsf6	ENSRNOG00000005927	7	60,416,925
5063	A_44_P375553	0.243	0.122	5.4E-02	0.460	0.061	6.3E-09	Lrp1	ENSRNOG00000025053	7	70,926,903
22629	A_44_P108003	-0.208	0.103	5.2E-02	-0.429	0.052	6.3E-10	Nipal2	ENSRNOG00000005190	7	73,450,262
21449	A_64_P104488	0.010	0.155	9.5E-01	-0.868	0.078	2.8E-13	Spag1	ENSRNOG00000010078	7	74,994,605
8600	A_44_P144591	0.524	0.354	1.5E-01	1.545	0.154	2.9E-11	Cthrc1	ENSRNOG00000004578	7	77,966,722
19370	A_64_P062869	-0.076	0.170	6.6E-01	-0.586	0.066	2.5E-10	Pkhd1l1	ENSRNOG00000004398	7	83,373,022
24750	A_44_P1016829	0.269	0.233	2.6E-01	0.933	0.116	1.5E-09	Trib1	ENSRNOG00000004100	7	99,954,492
14240	A_64_P123307	0.096	0.139	5.0E-01	0.533	0.070	4.3E-09	Zfat	ENSRNOG00000025140	7	109,205,354
22083	A_64_P107623	0.087	0.078	2.7E-01	0.305	0.039	3.0E-09	Rps19	ENSRNOG00000031474	7	111,722,964
10974	A_44_P1003602	-0.049	0.097	6.2E-01	0.602	0.049	1.5E-14	LOC300024	ENSRNOG00000048850	7	116,376,897
9125	A_64_P162437	-0.278	0.145	6.2E-02	-1.029	0.072	2.1E-16	Ly6h	ENSRNOG00000007334	7	116,607,473
21302	A_44_P291102	-0.254	0.149	9.7E-02	-0.635	0.075	3.8E-10	Smpd5	ENSRNOG00000037299	7	117,351,648
2438	A_42_P797218	0.073	0.111	5.2E-01	-0.436	0.056	2.6E-09	Gpt	ENSRNOG00000033915	7	117,759,083
10586	A_64_P025496	-0.218	0.158	1.8E-01	0.710	0.079	9.7E-11	Maff	ENSRNOG00000012886	7	120,580,743
24866	A_44_P123677	0.086	0.093	3.6E-01	0.442	0.046	2.1E-11	Syngr1	ENSRNOG00000017108	7	121,311,024
8729	A_64_P152878	-0.255	0.194	2.0E-01	-0.879	0.100	3.0E-10	Shisa8 (RGD156	ENSRNOG00000049407	7	123,445,613
12113	A_64_P133261	-0.209	0.191	2.8E-01	-0.729	0.095	4.8E-09	A4galt	ENSRNOG00000009736	7	124,089,385
8829	A_64_P267897	-2.395	0.133	1.1E-19	0.386	0.066	1.2E-06	Efcab6	ENSRNOG00000011094	7	124,929,025
4907	A_64_P138430	-0.094	0.131	4.8E-01	-0.579	0.065	1.3E-10	Odf3b	ENSRNOG00000037060	7	130,350,570
27123	A_64_P133967	-0.203	0.179	2.6E-01	-0.728	0.093	4.0E-09	Abcd2	ENSRNOG00000015538	7	132,343,169
27271	A_64_P061865	0.015	0.070	8.3E-01	-0.261	0.035	8.1E-09	Tcfcp2	ENSRNOG00000032395	7	142,180,794
18693	A_64_P030474	-0.003	0.075	9.7E-01	-0.362	0.038	1.7E-11	Tns2	ENSRNOG00000010588	7	143,707,237
27165	A_44_P378799	0.144	0.128	2.7E-01	0.548	0.064	3.3E-10	Igfbp6	ENSRNOG00000010977	7	143,749,221
27356	A_43_P12384	0.106	0.119	3.8E-01	0.453	0.059	4.6E-09	Birc3	ENSRNOG00000005731	8	6,076,598
16694	A_64_P153442	0.031	0.084	7.1E-01	-0.335	0.042	1.6E-09	Zfp810	ENSRNOG00000014331	8	23,282,989
8883	A_64_P151774	-0.153	0.100	1.3E-01	-0.648	0.050	3.3E-15	Bmper	ENSRNOG00000015357	8	24,369,916
26835	A_64_P145023	0.165	0.084	5.6E-02	-0.340	0.042	1.2E-09	Snx19	ENSRNOG00000014719	8	31,497,639
418	A_42_P558703	-0.277	0.177	1.3E-01	-1.087	0.088	1.8E-14	Adamts15	ENSRNOG00000009892	8	32,000,378
20835	A_44_P607412	0.134	0.083	1.1E-01	-0.338	0.041	1.0E-09	Nfrkb	ENSRNOG00000008278	8	32,530,412
14190	A_44_P107097	0.072	0.085	4.0E-01	0.350	0.042	7.6E-10	Dcps	ENSRNOG00000009993	8	36,374,673
8990	A_42_P506685	0.197	0.173	2.6E-01	0.805	0.086	3.9E-11	Fxyd2	ENSRNOG00000016469	8	49,710,477

Disease Models & Mechanisms • Supplementary information

22171	A_64_P126756	0.173	0.159	2.8E-01	0.702	0.079	1.4E-10	Fxyd2	ENSRNOG00000016469	8	49,713,190
24881	A_43_P19907	-0.012	0.144	9.3E-01	0.786	0.072	4.8E-13	Loxl1	ENSRNOG00000008680	8	63,092,009
12017	A_64_P019130	-0.063	0.076	4.1E-01	-0.322	0.038	3.6E-10	Ccpg1	ENSRNOG00000053428	8	79,660,657
14777	A_64_P156682	-0.105	0.080	2.0E-01	-0.361	0.040	9.3E-11	Hmgn3	ENSRNOG00000031032	8	90,664,554
12686	A_64_P136153	-0.023	0.087	7.9E-01	0.321	0.043	9.7E-09	Me1	ENSRNOG00000009715	8	94,352,246
22148	A_44_P283339	0.175	0.105	1.0E-01	0.577	0.052	4.4E-13	Tmed3	ENSRNOG00000013889	8	97,083,120
1145	A_42_P466706	0.022	0.083	8.0E-01	0.383	0.041	4.2E-11	PCOLCE2	ENSRNOG00000046848	8	103,459,161
3780	A_44_P242924	0.013	0.079	8.7E-01	-0.309	0.040	2.9E-09	Trpc1	ENSRNOG00000054902	8	103,554,399
51	A_64_P049401	0.002	0.097	9.9E-01	-0.383	0.048	2.0E-09	RGD1310507	ENSRNOG00000009434	8	111,777,677
6747	A_44_P1024981	-0.725	0.094	4.1E-09	-0.038	0.047	4.2E-01	Alas1	ENSRNOG00000056596	8	114,940,163
18110	A_64_P042591	0.193	0.111	8.9E-02	-0.601	0.055	6.1E-13	RGD1307461	ENSRNOG00000015718	8	116,094,851
21487	A_64_P099055	0.176	0.098	7.9E-02	0.475	0.049	1.2E-11	Lsmem2 (LOC68	ENSRNOG00000052634	8	116,349,896
9855	A_44_P382831	-0.172	0.108	1.2E-01	-0.411	0.054	5.6E-09	Epm2aip1	ENSRNOG00000043006	8	119,524,039
9908	A_42_P689873	-0.032	0.106	7.6E-01	-0.437	0.053	7.3E-10	Cmtm8	ENSRNOG00000011201	8	122,904,913
25973	A_44_P284129	0.121	0.119	3.2E-01	0.483	0.059	1.1E-09	Csrnp1	ENSRNOG00000033433	8	128,672,284
5839	A_64_P173102	-0.016	0.098	8.7E-01	0.399	0.049	1.1E-09	Rpsa	ENSRNOG00000018645	8	128,806,129
14280	A_44_P699398	0.052	0.083	5.4E-01	-0.365	0.042	1.8E-10	Zfp445	ENSRNOG00000025554	8	131,899,023
3570	A_64_P084445	0.047	0.120	7.0E-01	-0.605	0.060	4.5E-12	Prr22	ENSRNOG00000047295	9	10,290,035
257	A_42_P501679	-0.093	0.067	1.8E-01	-0.248	0.034	9.3E-09	M6prbp1	ENSRNOG00000048834	9	10,773,901
18255	A_64_P126165	-0.005	0.075	9.5E-01	-1.350	0.037	6.0E-30	LOC681367	ENSRNOG00000046871	9	16,519,144
9309	A_64_P011739	0.087	0.062	1.7E-01	-0.271	0.031	1.9E-10	Xpo5	ENSRNOG00000019085	9	17,163,170
26450	A_44_P258417	0.100	0.149	5.1E-01	0.716	0.078	8.7E-11	Mad2l1bp	ENSRNOG00000019463	9	17,216,495
16969	A_64_P045957	-0.088	0.073	2.4E-01	-0.429	0.036	5.9E-14	Mrpl14	ENSRNOG00000019734	9	17,698,569
20361	A_44_P241616	-0.039	0.081	6.4E-01	-0.571	0.040	2.7E-16	Ogfrl1	ENSRNOG00000014142	9	29,647,903
15120	A_64_P115204	-0.106	0.110	3.4E-01	0.533	0.055	1.4E-11	Sdhaf4	ENSRNOG00000028087	9	30,251,388
27130	A_44_P451120	0.200	0.118	9.9E-02	0.492	0.059	6.6E-10	Lmbrd1	ENSRNOG00000012178	9	30,939,555
5786	A_44_P975235	0.112	0.076	1.5E-01	-0.291	0.038	4.7E-09	RGD1309220	ENSRNOG00000015417	9	42,943,198
29640	A_44_P822041	0.118	0.139	4.0E-01	0.554	0.070	1.9E-09	none	ENSRNOG00000055728	9	52,937,765
18668	A_64_P142330	-0.057	0.099	5.7E-01	0.479	0.050	1.6E-11	Tmem258b	ENSRNOG00000032740	9	61,552,374
5919	A_42_P664544	0.188	0.168	2.7E-01	0.619	0.084	9.9E-09	Rftn2	ENSRNOG00000015594	9	61,810,417
9726	A_44_P277068	-0.009	0.064	8.9E-01	0.236	0.032	9.3E-09	RGD1562399	ENSRNOG00000032289	9	66,045,962

6093	A_64_P151012	0.042	0.120	7.3E-01	0.448	0.060	7.9E-09	Pard3b	ENSRNOG00000024345	9	68,414,339
20354	A_64_P077570	0.013	0.176	9.4E-01	0.655	0.083	3.3E-09	Fn1	ENSRNOG00000014288	9	78,969,078
21414	A_43_P11879	0.406	0.255	1.2E-01	0.832	0.081	6.8E-12	Htr2b	ENSRNOG00000017625	9	93,125,014
273	A_44_P143922	0.163	0.107	1.4E-01	0.483	0.053	8.5E-11	Hes6	ENSRNOG00000020194	9	98,551,410
27882	A_44_P381270	0.273	0.172	1.2E-01	0.705	0.088	1.8E-09	Macir (RGD1562	ENSRNOG00000032398	9	111,327,402
9804	A_64_P148368	-0.120	0.085	1.7E-01	-0.388	0.043	7.4E-11	Mtcl1 (RGD1308:	ENSRNOG00000025527	9	114,619,711
20174	A_64_P044435	0.252	0.159	1.2E-01	0.680	0.080	3.3E-10	Tgif1	ENSRNOG00000015906	9	119,190,698
22463	A_44_P142850	-0.399	0.109	8.2E-04	1.351	0.055	3.0E-24	Fam86a	ENSRNOG00000002876	10	10,530,365
7939	A_64_P031975	-0.106	0.092	2.6E-01	0.490	0.046	1.2E-12	Alg1	ENSRNOG00000002883	10	10,550,138
20155	A_44_P298013	-0.143	0.078	7.4E-02	-0.318	0.039	9.6E-10	Nudt16l1	ENSRNOG00000003224	10	10,831,782
12289	A_43_P12438	-0.100	0.062	1.2E-01	-0.712	0.031	4.3E-23	Hmox2	ENSRNOG00000003773	10	11,035,484
12372	A_64_P071193	-0.369	0.119	3.8E-03	0.663	0.060	3.1E-13	Nmral1	ENSRNOG00000003794	10	11,046,024
26992	A_42_P826202	0.217	0.191	2.7E-01	2.546	0.081	2.7E-19	Znf597	ENSRNOG00000007576	10	11,912,543
17837	A_64_P050084	0.327	0.189	9.2E-02	0.954	0.094	4.3E-12	Npw	ENSRNOG00000012390	10	14,022,452
17979	A_43_P12626	-4.067	0.231	5.3E-19	-0.202	0.107	6.8E-02	Msln	ENSRNOG00000019445	10	15,125,408
14401	A_44_P419922	0.166	0.193	3.9E-01	-2.750	0.098	1.2E-25	Haghl	ENSRNOG00000019612	10	15,155,412
24121	A_43_P18716	-0.098	0.136	4.8E-01	-3.029	0.089	2.1E-24	RGD1560244	ENSRNOG00000022331	10	15,156,207
21202	A_64_P055748	0.167	0.098	9.7E-02	-0.706	0.049	1.3E-16	Rhbdf1	ENSRNOG00000020594	10	15,672,382
14109	A_44_P556319	0.039	0.137	7.8E-01	-1.444	0.068	6.1E-22	Fgf18	ENSRNOG00000048389	10	18,063,391
3403	A_64_P045750	0.252	0.351	4.8E-01	0.951	0.097	3.2E-10	Kcnmb1	ENSRNOG00000005465	10	18,906,010
21683	A_64_P070801	-0.156	0.238	5.2E-01	-1.029	0.119	2.5E-10	LOC684480	ENSRNOG00000046377	10	34,439,470
21210	A_64_P025690	0.029	0.063	6.5E-01	-1.469	0.031	6.1E-34	none	ENSRNOG00000001512	10	44,659,707
11237	A_64_P023975	0.113	0.082	1.8E-01	-0.338	0.041	9.1E-10	Zbtb4	ENSRNOG00000014689	10	56,381,813
18295	A_64_P009032	-0.123	0.089	1.7E-01	-0.378	0.044	3.5E-10	Camta2	ENSRNOG00000004283	10	57,309,298
6320	A_64_P119134	-0.009	0.095	9.3E-01	-0.362	0.047	4.8E-09	Smtnl2	ENSRNOG00000015157	10	58,973,020
7671	A_44_P135082	0.284	0.238	2.4E-01	0.659	0.079	3.2E-09	P2rx1	ENSRNOG00000017606	10	59,566,223
864	A_44_P144202	-0.026	0.086	7.7E-01	0.466	0.043	6.9E-13	Serpinf1	ENSRNOG00000003172	10	62,254,287
25258	A_44_P961164	0.004	0.117	9.7E-01	-0.622	0.059	1.2E-12	Rhbdl3	ENSRNOG00000005515	10	67,677,071
5896	A_64_P019759	0.124	0.084	1.5E-01	0.351	0.042	5.6E-10	none	ENSRNOG00000037114	10	70,366,725
20882	A_44_P350521	-0.464	0.187	1.8E-02	0.711	0.094	5.3E-09	Col1a1	ENSRNOG00000003897	10	82,745,801
4178	A_64_P008216	0.033	0.096	7.3E-01	0.539	0.048	2.5E-13	Ppp1r9b	ENSRNOG00000052113	10	82,800,704

Disease Models & Mechanisms • Supplementary information

17635	A_42_P528374	-0.028	0.140	8.4E-01	0.616	0.070	1.7E-10	Pnmt	ENSRNOG00000046057	10	86,340,940
10623	A_44_P491759	-0.185	0.158	2.5E-01	0.660	0.079	6.0E-10	Krt24	ENSRNOG00000010970	10	87,195,075
23747	A_64_P096046	0.051	0.092	5.9E-01	-1.545	0.046	8.5E-29	none	ENSRNOG00000051172	10	87,936,692
6755	A_64_P065032	-0.005	0.088	9.6E-01	0.343	0.044	2.9E-09	Sc65	ENSRNOG00000015787	10	88,325,326
30281	A_42_P834852	0.300	0.072	1.7E-04	-0.265	0.036	9.0E-09	Higd1b	ENSRNOG00000002814	10	90,929,423
23038	A_64_P083384	0.060	0.073	4.2E-01	0.418	0.036	1.2E-13	Nmt1	ENSRNOG00000002989	10	91,126,689
28777	A_64_P028270	0.105	0.111	3.5E-01	2.018	0.055	4.0E-30	Mettl2	ENSRNOG00000006131	10	93,354,003
28992	A_44_P680749	0.088	0.091	3.4E-01	-0.412	0.045	7.4E-11	RGD1565033	ENSRNOG00000043295	10	95,242,986
6640	A_64_P064488	0.103	0.063	1.1E-01	0.300	0.032	2.4E-11	RGD1359290	ENSRNOG00000027271	10	95,642,640
76	A_43_P19450	0.193	0.164	2.5E-01	0.644	0.082	2.3E-09	Prkca	ENSRNOG00000003491	10	96,584,947
25564	A_64_P019608	-0.076	0.108	4.9E-01	0.631	0.054	7.8E-14	Prkca	ENSRNOG00000003491	10	96,584,947
25996	A_64_P121995	-0.142	0.093	1.4E-01	-0.397	0.047	3.6E-10	Sap30bp	ENSRNOG00000005482	10	104,483,042
18881	A_64_P134245	0.154	0.088	8.7E-02	0.369	0.044	4.7E-10	Fbf1	ENSRNOG00000008577	10	104,718,621
11007	A_44_P381917	-0.155	0.168	3.6E-01	0.727	0.095	9.0E-09	Sphk1	ENSRNOG00000010626	10	105,498,728
14738	A_44_P380392	0.003	0.059	9.6E-01	0.241	0.030	1.1E-09	Slc38a10	ENSRNOG00000004604	10	109,333,899
13966	A_64_P094267	0.039	0.156	8.0E-01	-0.607	0.078	3.0E-09	Pde6g (LOC6882	ENSRNOG00000046962	10	109,622,745
23270	A_64_P055509	0.180	0.121	1.5E-01	0.507	0.060	5.3E-10	none	ENSRNOG00000050286	11	2,813,912
28311	A_42_P830311	-0.067	0.102	5.1E-01	-0.508	0.051	6.8E-12	Mrap	ENSRNOG00000021524	11	30,904,733
9149	A_64_P162743	-0.027	0.114	8.1E-01	-0.656	0.057	1.3E-13	Dnajc28	ENSRNOG00000002026	11	31,772,984
29585	A_64_P104237	-0.137	0.072	6.5E-02	-0.312	0.036	2.4E-10	Ttc3	ENSRNOG00000001682	11	34,598,492
8596	A_64_P136279	-0.003	0.067	9.6E-01	-0.255	0.034	6.2E-09	Senp7	ENSRNOG00000001616	11	47,027,667
13054	A_43_P11086	-3.201	0.249	4.8E-15	-0.145	0.125	2.5E-01	Upk1b	ENSRNOG00000027380	11	64,522,130
917	A_42_P576953	0.149	0.089	1.0E-01	0.390	0.044	1.7E-10	Pla1a	ENSRNOG00000057153	11	64,882,288
24062	A_64_P087768	-0.031	0.114	7.9E-01	-0.605	0.057	1.1E-12	Nr1i2	ENSRNOG00000002906	11	65,022,100
24064	A_64_P023222	0.167	0.084	5.5E-02	0.468	0.042	3.5E-13	Gtf2e1	ENSRNOG00000026008	11	66,078,820
1641	A_42_P571235	-0.175	0.092	6.7E-02	-0.457	0.046	7.7E-12	Iqcb1	ENSRNOG00000038868	11	66,878,551
27889	A_43_P14782	0.070	0.079	3.8E-01	-0.524	0.040	1.9E-15	Tnk2	ENSRNOG00000001769	11	71,368,456
13359	A_44_P214811	-1.709	0.181	2.6E-11	0.077	0.090	4.0E-01	Tfrc	ENSRNOG00000001766	11	71,419,223
20227	A_64_P047537	-0.048	0.137	7.3E-01	0.675	0.069	9.0E-12	Tmem44	ENSRNOG00000001726	11	73,738,433
7118	A_64_P054708	-0.157	0.211	4.6E-01	1.606	0.106	2.8E-17	Hes1	ENSRNOG00000001720	11	74,315,248
19826	A_64_P129273	-0.149	0.097	1.3E-01	-0.435	0.048	9.4E-11	Hrasls	ENSRNOG00000001711	11	75,144,903

Disease Models & Mechanisms • Supplementary information

6841	A_42_P554157	-0.016	0.110	8.9E-01	0.491	0.055	1.1E-10	Dnajb11	ENSRNOG00000001803	11	81,757,813
16895	A_64_P038926	-0.279	0.144	6.1E-02	-0.773	0.072	8.6E-13	Prodh	ENSRNOG00000000281	11	87,058,616
24450	A_42_P762829	0.263	0.183	1.6E-01	0.683	0.092	7.8E-09	Cebpd	ENSRNOG00000050869	11	89,008,008
27850	A_44_P406805	-0.114	0.069	1.1E-01	0.294	0.034	3.0E-10	Rfc3	ENSRNOG00000001088	12	1,460,538
22669	A_44_P164824	0.053	0.091	5.7E-01	-0.363	0.046	1.8E-09	Ttyh3	ENSRNOG00000055583	12	16,084,266
2131	A_44_P852017	0.038	0.102	7.1E-01	0.448	0.051	1.5E-10	Gpr146	ENSRNOG00000001288	12	17,358,617
4574	A_64_P038729	0.312	0.167	6.9E-02	0.993	0.083	4.3E-14	none_LOC68134	ENSRNOG00000047077	12	20,882,344
25539	A_64_P094434	-0.015	0.083	8.6E-01	0.445	0.042	1.0E-12	Pcolce	ENSRNOG00000025001	12	22,153,983
29597	A_42_P833212	-0.088	0.056	1.2E-01	-0.235	0.028	4.8E-10	Eif4h	ENSRNOG00000001454	12	25,093,149
3110	A_64_P129805	-0.007	0.105	9.5E-01	0.460	0.052	1.6E-10	Rfc2	ENSRNOG00000001457	12	25,143,480
23298	A_64_P110474	0.034	0.061	5.8E-01	-0.234	0.030	4.0E-09	Gtf2i	ENSRNOG00000001479	12	25,411,292
932	A_64_P073004	0.179	0.120	1.4E-01	0.463	0.060	3.7E-09	Gusb	ENSRNOG00000000913	12	30,198,822
1282	A_44_P249902	0.054	0.066	4.1E-01	0.261	0.033	1.8E-09	Vps29	ENSRNOG00000001274	12	39,699,113
12850	A_64_P143507	-0.129	0.100	2.1E-01	-0.388	0.050	3.6E-09	Alkbh2	ENSRNOG00000028584	12	48,257,609
7231	A_44_P776918	0.117	0.147	4.3E-01	-0.737	0.074	5.8E-12	Rnf152	ENSRNOG00000014859	13	25,262,469
25890	A_64_P084578	0.237	0.135	8.7E-02	-0.500	0.067	9.4E-09	Rnf152	ENSRNOG00000014859	13	25,262,469
2841	A_42_P507072	-0.092	0.084	2.8E-01	-0.357	0.042	3.7E-10	none	ENSRNOG00000053210	13	50,196,042
28935	A_64_P141541	0.342	0.055	3.5E-07	-0.221	0.028	1.6E-09	Ndufv3	ENSRNOG00000027593	13	51,297,621
9518	A_44_P560973	-0.049	0.093	6.0E-01	-0.745	0.046	5.1E-18	Lamc2	ENSRNOG00000002667	13	70,626,252
26569	A_44_P480361	-0.024	0.086	7.8E-01	0.320	0.043	8.2E-09	Cenpl	ENSRNOG00000038789	13	78,886,163
13738	A_64_P050502	-0.308	0.186	1.1E-01	-0.998	0.095	2.0E-12	Ankrd45	ENSRNOG00000051303	13	78,976,154
6395	A_42_P547246	-0.221	0.155	1.6E-01	-0.710	0.078	6.0E-11	Myoc	ENSRNOG00000003221	13	80,517,536
6836	A_43_P10161	0.114	0.085	1.9E-01	-0.335	0.042	2.1E-09	Kifap3	ENSRNOG00000002544	13	82,072,497
29150	A_44_P435604	0.056	0.072	4.5E-01	-0.350	0.036	1.3E-11	Atp1b1	ENSRNOG00000002934	13	82,758,004
5634	A_64_P018081	-0.078	0.085	3.6E-01	-0.454	0.043	1.1E-12	Ddr2	ENSRNOG00000002881	13	88,436,789
21779	A_44_P306439	0.300	0.162	7.3E-02	1.147	0.083	7.9E-16	Fcgr3a	ENSRNOG00000024382	13	89,385,859
5407	A_42_P565621	0.141	0.149	3.5E-01	0.692	0.074	3.9E-11	Slamf9	ENSRNOG00000008045	13	90,839,411
27586	A_64_P236342	-0.527	0.167	3.6E-03	1.185	0.086	8.2E-15	Tgfb2	ENSRNOG00000002418	13	105,141,030
6518	A_43_P11044	0.839	0.197	1.4E-04	-0.789	0.099	1.6E-09	G0s2	ENSRNOG00000006019	13	112,005,052
29630	A_64_P015335	0.143	0.105	1.8E-01	0.415	0.053	2.3E-09	Mfsd7	ENSRNOG00000023937	14	2,311,122
27087	A_64_P103517	0.243	0.227	2.9E-01	-0.910	0.113	1.5E-09	Ephx4	ENSRNOG00000023389	14	3,389,943

Disease Models & Mechanisms • Supplementary information

25065	A_64_P247541	-0.140	0.076	7.5E-02	-0.355	0.038	3.9E-11	Pcdh7	ENSRNOG00000012367	14	55,081,551
3786	A_43_P21699	0.090	0.116	4.4E-01	0.490	0.058	4.7E-10	Pi4k2b	ENSRNOG00000003924	14	60,594,844
4984	A_64_P020244	0.152	0.136	2.7E-01	-0.675	0.068	7.9E-12	Prom1	ENSRNOG00000003098	14	71,533,063
19807	A_64_P073904	0.076	0.117	5.2E-01	-0.692	0.058	5.2E-14	Prom1	ENSRNOG00000003098	14	71,533,063
22474	A_64_P079751	0.081	0.112	4.7E-01	0.437	0.056	2.9E-09	Bst1	ENSRNOG00000003064	14	71,814,523
27240	A_44_P248172	-0.052	0.074	4.8E-01	0.489	0.037	2.1E-15	C1qtnf7	ENSRNOG00000005094	14	72,122,158
3073	A_64_P031329	-0.276	0.232	2.4E-01	-1.050	0.116	8.0E-11	Stk32b	ENSRNOG00000031397	14	77,829,400
2419	A_42_P833264	-0.172	0.133	2.0E-01	-0.494	0.066	8.8E-09	Zfp278	ENSRNOG00000018709	14	83,510,640
15680	A_64_P063896	-0.145	0.082	8.3E-02	0.320	0.041	2.5E-09	Selm	ENSRNOG00000061231	14	83,752,393
18458	A_64_P138889	-0.116	0.060	6.0E-02	-0.292	0.030	1.1E-11	Pold2	ENSRNOG00000014098	14	86,116,363
2451	A_42_P491505	-0.099	0.061	1.2E-01	-0.390	0.031	6.9E-15	Purb	ENSRNOG00000056150	14	86,739,335
7796	A_42_P466997	0.024	0.085	7.8E-01	0.357	0.043	5.5E-10	Ccm2	ENSRNOG00000060825	14	86,813,082
12854	A_64_P129855	-0.050	0.102	6.3E-01	-0.466	0.051	6.4E-11	Meis1	ENSRNOG00000004606	14	103,321,270
5272	A_64_P038050	1.268	0.083	2.6E-17	-0.209	0.042	1.4E-05	Acyp2	ENSRNOG00000042419	14	115,052,450
17821	A_64_P100148	1.767	0.152	9.1E-14	-0.264	0.076	1.4E-03	Lrmda (LOC6813	N/A	15	1,269,869
8650	A_42_P607939	-0.676	0.074	5.6E-11	-0.072	0.037	5.8E-02	Chchd1	ENSRNOG00000009297	15	4,026,637
1991	A_42_P723584	-0.023	0.058	7.0E-01	-0.225	0.029	3.7E-09	Nat12	ENSRNOG00000014192	15	26,033,791
5685	A_42_P775609	0.161	0.091	8.5E-02	-0.408	0.046	9.9E-11	Jub	ENSRNOG00000012791	15	33,218,456
25995	A_42_P641517	0.432	0.217	5.4E-02	0.916	0.109	4.6E-10	Mcpt1l1 (intrupte	ENSRNOG00000053494	15	34,612,432
10880	A_44_P238556	0.380	0.193	5.9E-02	0.941	0.101	2.4E-10	Mcpt2	ENSRNOG00000020625	15	34,862,281
1636	A_42_P684885	-0.244	0.182	1.9E-01	-1.120	0.091	1.6E-14	Atp12a	ENSRNOG00000020685	15	36,565,495
15751	A_44_P531870	-0.072	0.155	6.4E-01	-2.707	0.077	1.8E-29	Ephx2	ENSRNOG00000017286	15	42,794,279
22768	A_64_P001612	-0.101	0.077	2.0E-01	-0.569	0.039	7.1E-17	Trim35	ENSRNOG00000009449	15	42,960,307
10988	A_44_P304057	0.093	0.061	1.4E-01	0.260	0.031	3.4E-10	Ppp2r2a	ENSRNOG00000011158	15	43,733,182
7867	A_44_P498522	0.122	0.076	1.2E-01	0.292	0.038	3.7E-09	Kctd9	ENSRNOG00000012951	15	44,411,865
19433	A_44_P432388	-0.096	0.176	5.9E-01	-1.194	0.131	1.3E-09	Tdh	ENSRNOG00000011342	15	46,681,467
4844	A_64_P010902	0.040	0.128	7.5E-01	0.963	0.064	3.6E-17	Msra	ENSRNOG00000012440	15	47,800,024
21894	A_64_P069071	0.320	0.216	1.5E-01	-1.344	0.108	1.3E-14	Lpar6	ENSRNOG00000015577	15	55,126,953
18762	A_44_P135531	0.185	0.108	1.0E-01	-2.319	0.108	3.3E-17	RGD1304929	ENSRNOG00000022760	15	57,065,428
23905	A_64_P163718	0.168	0.086	5.9E-02	-0.346	0.043	1.5E-09	Slc25a30	ENSRNOG00000001052	15	57,834,173
1297	A_44_P276220	0.094	0.102	3.6E-01	0.446	0.051	1.7E-10	Gtf2f2	ENSRNOG00000029316	15	58,068,892

Disease Models & Mechanisms • Supplementary information

8579	A_64_P057340	-0.025	0.074	7.3E-01	-0.421	0.037	1.7E-13	Abcc4	ENSRNOG00000010064	15	103,927,592
4173	A_64_P117248	-0.076	0.100	4.5E-01	0.427	0.050	3.0E-10	Ppif	ENSRNOG00000010558	16	1,979,191
14428	A_44_P1000888	0.029	0.125	8.2E-01	0.755	0.062	2.7E-14	Plac9	ENSRNOG00000011263	16	3,851,270
20576	A_64_P122190	0.194	0.152	2.1E-01	0.787	0.076	2.2E-12	Plac9	ENSRNOG00000011263	16	3,851,270
23502	A_64_P070756	-0.039	0.065	5.5E-01	-0.266	0.032	8.9E-10	Anxa11	ENSRNOG00000010984	16	3,912,148
11180	A_64_P068548	0.242	0.139	9.0E-02	0.710	0.069	3.2E-12	Arhgap22	ENSRNOG00000024728	16	9,486,832
18286	A_44_P384017	0.096	0.172	5.8E-01	0.739	0.086	3.0E-10	Sncg	ENSRNOG00000058006	16	10,726,707
24518	A_64_P046248	0.512	0.206	1.8E-02	-0.774	0.103	7.1E-09	Mat1a	ENSRNOG00000011351	16	18,690,246
24366	A_44_P290394	-0.324	0.162	5.3E-02	-0.823	0.081	4.1E-12	Slc27a1	ENSRNOG00000018170	16	19,999,112
22352	A_44_P215131	0.025	0.098	8.0E-01	0.418	0.049	3.1E-10	Mk1	ENSRNOG00000019657	16	20,534,209
2924	A_43_P14632	-0.119	0.095	2.2E-01	-0.377	0.048	1.9E-09	Acsl1	ENSRNOG00000010633	16	48,981,980
11713	A_64_P014366	0.018	0.146	9.0E-01	0.633	0.073	2.3E-10	Sorbs2	ENSRNOG00000013391	16	49,820,235
15277	A_64_P054561	0.030	0.125	8.1E-01	0.563	0.062	8.7E-11	LOC498662	ENSRNOG00000027245	16	80,729,400
7494	A_42_P633958	0.262	0.152	9.4E-02	1.051	0.076	5.6E-16	Col4a2	ENSRNOG00000023972	16	83,438,561
17237	A_42_P679295	0.188	0.105	8.3E-02	0.438	0.053	6.4E-10	RGD1311345	ENSRNOG00000019232	17	6,683,152
4329	A_44_P620106	-0.026	0.084	7.5E-01	0.312	0.042	8.2E-09	Tgfbi	ENSRNOG00000012216	17	8,429,338
12435	A_64_P054743	-0.001	0.086	9.9E-01	-0.346	0.043	1.5E-09	Pcbd2	ENSRNOG00000047796	17	8,980,571
23510	A_44_P1039249	0.248	0.153	1.1E-01	0.579	0.077	6.0E-09	Dok3	ENSRNOG00000013564	17	9,639,330
24240	A_64_P156438	0.094	0.094	3.3E-01	0.809	0.047	6.1E-19	Zfp346	ENSRNOG00000016867	17	10,061,772
19380	A_64_P155920	-0.104	0.123	4.0E-01	-0.512	0.061	5.9E-10	Cplx2	ENSRNOG00000000105	17	10,818,835
9101	A_44_P201028	-0.103	0.151	5.0E-01	0.568	0.076	6.8E-09	Nfil3	ENSRNOG00000011668	17	12,261,102
20867	A_64_P059032	-0.230	0.163	1.7E-01	0.666	0.077	4.1E-10	Susd3	ENSRNOG00000016525	17	15,814,132
1813	A_42_P783550	-0.050	0.087	5.7E-01	-0.325	0.043	7.5E-09	Rreb1	ENSRNOG00000015701	17	27,602,934
6034	A_64_P023472	0.229	0.164	1.7E-01	0.633	0.082	3.5E-09	Serpinb1a	ENSRNOG00000016581	17	32,904,119
3910	A_64_P045057	-0.004	0.102	9.7E-01	-0.405	0.051	2.1E-09	Gpld1	ENSRNOG00000017702	17	42,127,678
9068	A_44_P548468	-0.037	0.090	6.8E-01	0.394	0.045	1.8E-10	Kif5b	ENSRNOG00000017466	17	54,181,419
16401	A_64_P149491	-0.153	0.135	2.7E-01	-0.640	0.068	2.5E-11	Mpp7	ENSRNOG00000018760	17	60,059,949
23495	A_64_P036509	1.277	0.153	5.6E-10	-0.014	0.076	8.6E-01	Chrm3	ENSRNOG00000049410	17	63,994,169
23649	A_44_P191021	0.004	0.105	9.7E-01	0.468	0.053	1.2E-10	Phyh	ENSRNOG00000018044	17	77,304,530
1142	A_44_P823025	-0.180	0.093	6.2E-02	-0.438	0.047	3.2E-11	Sephs1	ENSRNOG00000018125	17	77,340,934
6327	A_64_P148776	-0.106	0.067	1.2E-01	-0.375	0.033	2.5E-13	Sephs1	ENSRNOG00000018125	17	77,340,934

Disease Models & Mechanisms • Supplementary information

3451	A_64_P126750	0.020	0.108	8.5E-01	0.611	0.054	2.1E-13	Cdnf	ENSRNOG00000026493	17	78,735,324
1172	A_42_P509365	0.086	0.085	3.2E-01	0.348	0.042	8.3E-10	Vim	ENSRNOG00000018087	17	80,882,666
13848	A_43_P19054	-0.131	0.138	3.5E-01	-0.626	0.069	7.1E-11	Dsc2	ENSRNOG00000039969	18	11,858,744
28750	A_44_P382255	0.164	0.115	1.6E-01	-0.513	0.058	1.2E-10	none_Dtna	ENSRNOG00000016671	18	14,471,213
10876	A_64_P049411	-0.012	0.113	9.2E-01	-0.419	0.057	9.4E-09	LOC679229	ENSRNOG00000048101	18	15,856,801
13454	A_64_P109506	0.209	0.140	1.5E-01	0.612	0.070	2.0E-10	Mocos	ENSRNOG00000015113	18	16,590,408
24597	A_64_P083905	0.107	0.068	1.2E-01	0.465	0.034	5.7E-16	Pik3c3	ENSRNOG00000017840	18	22,964,210
18807	A_64_P158068	0.010	0.090	9.1E-01	0.624	0.045	4.5E-16	Wdr36	ENSRNOG00000027355	18	25,637,588
27518	A_44_P1033758	0.141	0.175	4.2E-01	-1.241	0.088	2.4E-16	Wdr36	ENSRNOG00000027355	18	25,637,588
479	A_42_P776018	-0.109	0.084	2.0E-01	-0.554	0.042	2.3E-15	Stard4	ENSRNOG00000020468	18	25,997,555
2083	A_42_P625263	-0.081	0.099	4.2E-01	-0.662	0.049	1.4E-15	Epb41l4a	ENSRNOG00000026050	18	26,658,892
5557	A_43_P15754	-0.100	0.080	2.2E-01	0.352	0.040	1.7E-10	none	ENSRNOG00000019525	18	27,749,235
950	A_64_P124641	-0.100	0.103	3.4E-01	0.414	0.051	1.3E-09	none	ENSRNOG00000060829	18	29,535,280
20756	A_64_P036233	-0.065	0.103	5.3E-01	0.480	0.051	3.4E-11	none	ENSRNOG00000060829	18	29,535,280
9887	A_64_P038414	0.328	0.182	8.0E-02	0.776	0.091	3.6E-10	Lox	ENSRNOG00000014426	18	47,513,065
26924	A_64_P155509	-0.125	0.067	6.9E-02	-0.342	0.033	3.1E-12	Snx2	ENSRNOG00000017832	18	48,132,414
5554	A_44_P897023	-0.187	0.220	4.0E-01	-0.894	0.112	2.0E-09	Snx24	ENSRNOG00000017488	18	48,201,728
8407	A_64_P058564	-0.719	0.057	8.1E-15	0.054	0.028	6.7E-02	Prrc1	ENSRNOG00000016433	18	52,423,883
7776	A_44_P203859	-0.201	0.202	3.3E-01	-0.819	0.101	1.2E-09	none_Piezo2	ENSRNOG00000038784	18	58,499,836
1853	A_64_P039601	0.097	0.065	1.4E-01	0.485	0.032	4.6E-17	Sec11c	ENSRNOG00000017036	18	61,490,031
8960	A_64_P029646	0.055	0.070	4.4E-01	0.319	0.035	6.3E-11	Rpl17	ENSRNOG00000018680	18	70,970,596
19064	A_64_P052765	0.415	0.212	5.8E-02	0.924	0.106	2.1E-10	Cdh16	ENSRNOG00000012222	19	568,287
14056	A_64_P162764	-0.127	0.175	4.7E-01	0.751	0.087	2.9E-10	none	ENSRNOG00000025764	19	11,302,938
12160	A_43_P11151	-0.053	0.083	5.3E-01	-0.338	0.041	1.0E-09	Nfatc3	ENSRNOG00000054264	19	38,039,729
4796	A_64_P094515	0.305	0.092	2.1E-03	-1.317	0.046	1.8E-26	LOC100360619	ENSRNOG00000053623	19	38,643,108
14458	A_44_P480636	0.143	0.113	2.1E-01	0.446	0.056	2.1E-09	Ddx19a	ENSRNOG00000018033	19	43,217,161
27532	A_44_P252441	0.056	0.110	6.1E-01	0.428	0.055	2.9E-09	St3gal2	ENSRNOG00000017932	19	43,290,363
1581	A_42_P712297	1.754	0.196	1.1E-10	-0.068	0.098	4.9E-01	Fa2h	ENSRNOG00000018950	19	43,596,801
21295	A_43_P11451	0.345	0.170	5.0E-02	1.119	0.085	2.3E-15	Ctrb1	ENSRNOG00000019068	19	43,911,057
25128	A_44_P491393	0.300	0.095	3.1E-03	-0.523	0.047	3.9E-13	Nudt7	ENSRNOG00000011976	19	46,455,761
6940	A_64_P052791	-0.016	0.122	9.0E-01	-0.561	0.061	5.1E-11	Cdh13	ENSRNOG00000014371	19	50,848,736

10658	A_44_P561084	-0.018	0.090	8.4E-01	-0.338	0.045	7.1E-09	Cdh13	ENSRNOG00000014371	19	50,848,736
24895	A_44_P529581	0.348	0.175	5.4E-02	0.710	0.087	1.2E-09	Osgin1	ENSRNOG00000014948	19	52,077,109
3616	A_64_P102518	0.091	0.108	4.1E-01	0.735	0.054	9.3E-16	Zc3h18	ENSRNOG00000028501	19	55,197,704
20497	A_44_P311821	0.139	0.077	7.8E-02	0.782	0.038	2.0E-21	Afg3l1	ENSRNOG00000026994	19	56,272,162
14753	A_64_P023365	-0.590	0.070	5.5E-10	-0.017	0.035	6.2E-01	Abcb10	ENSRNOG00000017993	19	56,772,904
27136	A_64_P108001	0.127	0.067	6.6E-02	-0.333	0.033	6.6E-12	Galnt2	ENSRNOG00000019143	19	57,047,830
25296	A_64_P108692	-0.081	0.070	2.5E-01	-0.613	0.035	2.5E-19	Ttc13	ENSRNOG00000018884	19	57,484,566
2423	A_42_P567268	0.496	0.268	7.3E-02	1.001	0.134	7.8E-09	Mt2A	ENSRNOG00000028841	20	3,677,474
21444	A_64_P158939	0.271	0.211	2.1E-01	0.885	0.105	5.1E-10	Mt2A	ENSRNOG00000028841	20	3,677,474
13348	A_64_P130578	0.198	0.127	1.3E-01	0.535	0.063	4.5E-10	RT1-Db1	ENSRNOG00000033215	20	4,087,618
11988	A_64_P026316	-1.203	0.162	8.7E-09	0.000	0.081	1.0E+00	none_C4b	ENSRNOG00000030729	20	4,316,715
15626	A_44_P332606	-0.089	0.081	2.8E-01	0.306	0.040	6.1E-09	C2	ENSRNOG00000000419	20	4,542,947
2785	A_64_P140658	-0.231	0.117	5.7E-02	-0.444	0.059	5.8E-09	Anks1a	ENSRNOG00000000498	20	7,484,550
1548	A_42_P535085	0.769	0.094	9.6E-10	-0.141	0.047	4.8E-03	Ndufv3	ENSRNOG00000001182	20	10,265,806
6923	A_64_P048765	0.421	0.134	3.4E-03	-0.614	0.067	5.8E-11	Cbs	ENSRNOG00000029528	20	10,386,663
21940	A_64_P228661	-0.137	0.070	5.9E-02	0.291	0.035	7.6E-10	Pcbp3	ENSRNOG00000001245	20	12,429,315
23949	A_64_P164581	-0.084	0.115	4.7E-01	0.543	0.058	2.8E-11	Pcbp3	ENSRNOG00000001245	20	12,429,315
13884	A_44_P1047108	-0.065	0.068	3.4E-01	-0.287	0.034	4.3E-10	Ddt	ENSRNOG00000001239	20	13,827,132
6478	A_44_P303982	0.006	0.113	9.6E-01	0.468	0.057	7.7E-10	Susd2	ENSRNOG00000033389	20	13,965,121
25896	A_64_P037095	-0.079	0.125	5.3E-01	-0.479	0.063	4.4E-09	Pald	ENSRNOG00000000561	20	30,975,472
3760	A_44_P393551	0.085	0.084	3.2E-01	-0.334	0.042	2.0E-09	Gja1	ENSRNOG00000000805	20	37,876,650
26775	A_44_P384677	0.102	0.113	3.8E-01	0.447	0.057	2.3E-09	Rtn4ip1	ENSRNOG00000000279	20	48,881,194
18300	A_44_P124772	0.099	0.085	2.5E-01	0.357	0.042	5.1E-10	Ascc3	ENSRNOG00000037604	20	55,253,686
23473	A_43_P12070	-0.046	0.105	6.6E-01	0.637	0.052	2.2E-14	Ly6c	ENSRNOG00000061813	chr7_KL	23,908
558	A_64_P073899	0.125	0.121	3.1E-01	-0.542	0.060	9.3E-11		ENSRNOG00000031780	MT	1
822	A_64_P044731	0.100	0.081	2.3E-01	0.334	0.041	8.8E-10		ENSRNOG00000031780	MT	1
1765	A_42_P661992	-0.315	0.163	6.1E-02	-0.670	0.081	8.3E-10		ENSRNOG00000031780	MT	1
2055	A_44_P232030	-0.092	0.064	1.6E-01	-1.543	0.032	2.1E-34		ENSRNOG00000031780	MT	1
2356	A_64_P153314	-0.067	0.078	4.0E-01	0.305	0.039	3.0E-09		ENSRNOG00000031780	MT	1
3189	A_64_P058192	-0.275	0.159	9.3E-02	-0.677	0.080	3.9E-10		ENSRNOG00000031780	MT	1
3581	A_64_P148683	-0.081	0.069	2.5E-01	-0.364	0.035	1.6E-12		ENSRNOG00000031780	MT	1

3842	A_44_P405704	0.072	0.148	6.3E-01	0.586	0.074	2.2E-09	ENSRNOG00000031780	MT	1
4783	A_44_P306863	0.014	0.079	8.6E-01	0.310	0.040	2.8E-09	ENSRNOG00000031780	MT	1
4915	A_64_P024630	0.164	0.090	7.5E-02	0.418	0.045	3.7E-11	ENSRNOG00000031780	MT	1
5961	A_44_P960035	0.096	0.078	2.3E-01	-0.307	0.039	2.6E-09	ENSRNOG00000031780	MT	1
6646	A_44_P713970	-0.094	0.077	2.3E-01	-1.609	0.039	3.9E-32	ENSRNOG00000031780	MT	1
7968	A_64_P110003	0.165	0.095	9.3E-02	0.455	0.048	2.0E-11	ENSRNOG00000031780	MT	1
8465	A_64_P147586	-0.142	0.097	1.5E-01	-2.394	0.049	1.0E-34	ENSRNOG00000031780	MT	1
8573	A_43_P11131	-0.044	0.090	6.3E-01	-0.396	0.045	1.5E-10	ENSRNOG00000031780	MT	1
9438	A_44_P394150	-0.056	0.100	5.8E-01	-1.588	0.050	5.1E-28	ENSRNOG00000031780	MT	1
10234	A_64_P058928	0.027	0.076	7.2E-01	0.293	0.038	3.4E-09	ENSRNOG00000031780	MT	1
10366	A_64_P118117	0.081	0.079	3.2E-01	0.412	0.040	2.1E-12	ENSRNOG00000031780	MT	1
11796	A_44_P398462	-0.114	0.100	2.6E-01	-1.715	0.050	3.3E-29	ENSRNOG00000031780	MT	1
12392	A_44_P318891	-0.159	0.111	1.6E-01	-2.378	0.055	1.3E-32	ENSRNOG00000031780	MT	1
12488	A_64_P078924	0.119	0.088	1.9E-01	0.326	0.044	9.8E-09	ENSRNOG00000031780	MT	1
13255	A_64_P077069	0.216	0.170	2.1E-01	-0.688	0.085	1.3E-09	ENSRNOG00000031780	MT	1
13682	A_64_P027242	0.075	0.087	4.0E-01	0.395	0.044	8.1E-11	ENSRNOG00000031780	MT	1
13826	A_64_P054363	-0.123	0.133	3.6E-01	-0.554	0.067	6.2E-10	ENSRNOG00000031780	MT	1
13880	A_44_P713734	0.123	0.069	8.1E-02	0.318	0.034	4.7E-11	ENSRNOG00000031780	MT	1
14301	A_43_P16020	0.203	0.355	5.7E-01	3.101	0.185	4.9E-18	ENSRNOG00000031780	MT	1
14314	A_44_P496853	-0.033	0.053	5.4E-01	-1.454	0.027	2.4E-36	ENSRNOG00000031780	MT	1
14990	A_64_P131111	-0.164	0.122	1.9E-01	-0.468	0.061	4.4E-09	ENSRNOG00000031780	MT	1
15048	A_64_P017103	0.055	0.111	6.2E-01	-1.601	0.056	1.7E-26	ENSRNOG00000031780	MT	1
18226	A_44_P836900	0.091	0.069	2.0E-01	0.388	0.034	2.2E-13	ENSRNOG00000031780	MT	1
18386	A_64_P155290	-0.105	0.079	1.9E-01	-0.329	0.040	6.4E-10	ENSRNOG00000031780	MT	1
18880	A_64_P057622	0.030	0.087	7.3E-01	-1.461	0.043	6.1E-29	ENSRNOG00000031780	MT	1
18983	A_64_P047391	0.106	0.072	1.5E-01	0.366	0.036	3.5E-12	ENSRNOG00000031780	MT	1
19493	A_64_P025507	-0.025	0.097	7.9E-01	0.451	0.048	3.7E-11	ENSRNOG00000031780	MT	1
19765	A_64_P155950	0.566	0.309	7.6E-02	0.815	0.098	1.2E-09	ENSRNOG00000031780	MT	1
20422	A_64_P128815	0.020	0.215	9.2E-01	-1.760	0.108	2.7E-18	ENSRNOG00000031780	MT	1
20790	A_64_P033001	0.200	0.113	8.6E-02	0.673	0.057	5.1E-14	ENSRNOG00000031780	MT	1
20800	A_44_P545895	-0.056	0.075	4.6E-01	-0.406	0.038	8.1E-13	ENSRNOG00000031780	MT	1

20804	A_44_P468573	-0.046	0.084	5.9E-01	-1.376	0.042	1.4E-28		ENSRNOG00000031780	MT	1
21008	A_43_P22501	0.011	0.082	9.0E-01	-0.402	0.041	1.2E-11		ENSRNOG00000031780	MT	1
22321	A_42_P603213	-0.028	0.158	8.6E-01	-0.595	0.079	6.1E-09		ENSRNOG00000031780	MT	1
22975	A_64_P162760	-0.093	0.153	5.5E-01	0.687	0.076	9.4E-11		ENSRNOG00000031780	MT	1
23524	A_64_P031108	-0.041	0.158	8.0E-01	-1.120	0.079	2.6E-16		ENSRNOG00000031780	MT	1
23698	A_64_P028379	0.415	0.224	7.3E-02	0.825	0.071	3.1E-13		ENSRNOG00000031780	MT	1
24076	A_44_P945336	-0.076	0.080	3.5E-01	-0.475	0.040	5.0E-14		ENSRNOG00000031780	MT	1
24540	A_64_P050969	0.236	0.140	1.0E-01	-0.532	0.070	5.4E-09		ENSRNOG00000031780	MT	1
24634	A_64_P069294	0.000	0.063	1.0E+00	-1.484	0.032	4.7E-34		ENSRNOG00000031780	MT	1
26704	A_64_P066586	-0.179	0.091	5.6E-02	0.435	0.045	1.7E-11		ENSRNOG00000031780	MT	1
27068	A_64_P143415	0.023	0.087	7.9E-01	-0.631	0.043	1.1E-16		ENSRNOG00000031780	MT	1
27129	A_64_P162408	0.069	0.076	3.7E-01	-1.910	0.038	4.2E-35		ENSRNOG00000031780	MT	1
27926	A_64_P122775	-0.013	0.127	9.2E-01	-1.564	0.064	3.8E-24		ENSRNOG00000031780	MT	1
28053	A_64_P127136	0.078	0.069	2.7E-01	0.369	0.035	9.8E-13		ENSRNOG00000031780	MT	1
28354	A_64_P025510	-0.081	0.112	4.7E-01	0.476	0.056	4.0E-10		ENSRNOG00000031780	MT	1
29141	A_64_P017493	-0.088	0.096	3.7E-01	-2.018	0.048	2.4E-32		ENSRNOG00000031780	MT	1
29296	A_44_P152335	0.090	0.095	3.5E-01	0.357	0.048	7.8E-09		ENSRNOG00000031780	MT	1
29449	A_64_P132109	-0.111	0.086	2.1E-01	-0.447	0.043	2.4E-12		ENSRNOG00000031780	MT	1
11763	A_64_P031741	0.404	0.099	2.3E-04	-0.412	0.049	6.0E-10		ENSRNOG00000031780	MT	1
2074	A_44_P238204	-0.042	0.116	7.2E-01	-0.664	0.058	1.4E-13	Mid1ip1	ENSRNOG00000003228	X	13,116,743
11421	A_44_P180060	0.226	0.120	6.9E-02	0.583	0.060	1.4E-11	Hdac6	ENSRNOG00000047281	X	15,396,895
17831	A_43_P13307	0.305	0.154	5.6E-02	0.950	0.077	1.6E-14	Gpm6b	ENSRNOG00000004613	X	29,648,403
6662	A_44_P1005051	-0.230	0.211	2.8E-01	-0.883	0.112	4.6E-09	Car5b	ENSRNOG00000029330	X	32,232,142
2763	A_42_P685330	-0.047	0.062	4.5E-01	-0.375	0.031	3.2E-14	Ap1s2	ENSRNOG00000038686	X	32,355,296
25456	A_44_P480696	-0.138	0.081	9.8E-02	-0.379	0.041	3.7E-11	Ap1s2	ENSRNOG00000038686	X	32,355,296
19304	A_64_P123949	-0.079	0.117	5.0E-01	-0.443	0.058	5.5E-09	Gk	ENSRNOG00000034116	X	54,303,825
28798	A_44_P473510	0.177	0.301	5.6E-01	2.265	0.206	3.2E-10	Vsig4	ENSRNOG00000038132	X	65,400,298
709	A_44_P260502	-0.034	0.100	7.3E-01	-2.485	0.050	6.5E-35	none	ENSRNOG00000026617	X	67,980,381
8429	A_44_P488594	0.009	0.086	9.1E-01	0.325	0.043	5.8E-09	Tspan6	ENSRNOG00000003786	X	104,726,816
37	A_64_P019719	-0.104	0.140	4.6E-01	0.670	0.070	1.8E-11	Srpx2	ENSRNOG00000003715	X	104,734,082
24862	A_64_P132324	0.009	0.063	8.9E-01	0.300	0.031	1.9E-11	none	ENSRNOG00000058183	X	106,607,352

Disease Models & Mechanisms • Supplementary information

9321	A_44_P263635	-0.086	0.056	1.3E-01	-0.233	0.028	6.4E-10	Prps1	ENSRNOG00000060262	X	111,798,233
28862	A_64_P012725	-0.047	0.203	8.2E-01	-0.874	0.101	2.6E-10	Atp2b3	ENSRNOG00000061304	X	157,312,028
958	A_42_P552341	0.138	0.100	1.8E-01	-0.529	0.050	1.4E-12	Sorl1	N/A	8	46,228,077
2550	A_44_P671340	-0.009	0.113	9.4E-01	0.431	0.056	4.6E-09	none_LOC498601		16	
2624	A_64_P023587	0.080	0.103	4.4E-01	0.419	0.052	1.1E-09	none_Ly96			
7696	A_64_P147838	0.050	0.077	5.2E-01	0.487	0.039	7.7E-15	LOC679199			
10852	A_44_P313230	-0.091	0.096	3.5E-01	0.421	0.048	1.6E-10	LOC685888			
15274	A_64_P095948	-0.058	0.109	6.0E-01	0.517	0.055	2.5E-11	LOC690138			
16008	A_44_P538165	0.053	0.130	6.9E-01	0.936	0.065	1.4E-16	Tspan17 (Fbxo23	ENSRNOG00000018122	17	10,356,980
16163	A_64_P143161	0.112	0.074	1.4E-01	-0.415	0.037	2.5E-13	RGD1309747			
19575	A_44_P986792	-0.104	0.159	5.2E-01	-0.708	0.083	4.9E-10	Sorl1	N/A	8	46,228,077
20759	A_64_P020968	0.314	0.169	7.3E-02	0.915	0.088	1.5E-11	Raet1l			
18371	A_43_P17271	0.784	0.102	4.3E-09	0.098	0.051	6.3E-02	none_RGD1307569		11	

Genes are regarded as significant for “non-shared” differential expression when they show $P<1\times10^{-8}$ in one comparison but not in the other ($P\geq0.05$ with a concordant direction or all P-values in the opposite direction).

Table S3G. A list of significant transcripts for non-shared differential expression in the aorta.

no	ProbeName	SHR/lzm (vs WKY/lzm) Fold change (log2-scale)	SHR/lzm (vs WKY/lzm) SE (log2-scale)	SHR/lzm (vs WKY/lzm) P-value	SHRSP/lzm (vs WKY/lzm) Fold change (log2-scale)	SHRSP/lzm (vs WKY/lzm) SE (log2-scale)	SHRSP/lzm (vs WKY/lzm) P-value	GeneName	Ensembl_rat	Chr	Transcription_start_site (Rnor_6.0)
30079	A_64_P153441	-0.206	0.109	7.6E-02	1.186	0.097	1.7E-09	no transcript	ENSRNOG00000018935	1	47,588,122
26822	A_64_P150067	-0.057	0.071	4.3E-01	-0.764	0.064	2.2E-09	Ndufa3	ENSRNOG00000060293	1	64,175,099
28760	A_64_P060497	6.097	0.273	3.8E-09	-0.336	0.170	8.0E-02	Apoc4	ENSRNOG00000018405	1	80,599,572
16474	A_64_P068858	0.011	0.099	9.1E-01	2.496	0.089	5.0E-15	Rps16	ENSRNOG00000019578	1	85,408,444
8683	A_42_P644209	-0.028	0.156	8.6E-01	1.722	0.139	1.4E-09	no transcript	ENSRNOG00000030932	1	88,361,712
5564	A_42_P696282	0.002	0.115	9.8E-01	-1.138	0.103	6.6E-09	Fuom (RGD1311	ENSRNOG00000018476	1	212,568,224
24939	A_64_P036440	-0.091	0.093	3.4E-01	-0.989	0.083	2.4E-09	Cox8b	ENSRNOG00000014656	1	213,650,247
10669	A_44_P792628	-1.011	0.093	9.0E-09	0.101	0.083	2.4E-01	Spef1 (RGD1311	ENSRNOG00000021247	3	123,718,432
13942	A_44_P271405	-1.633	0.109	8.2E-11	0.058	0.097	5.6E-01	Pxmp4	ENSRNOG00000016975	3	150,108,898
17113	A_64_P139590	-0.016	0.108	8.9E-01	1.168	0.096	1.9E-09	Slc35c2	ENSRNOG00000018649	3	161,812,291
9281	A_44_P885189	0.042	0.137	7.6E-01	1.950	0.131	2.2E-10	Lrrc4	ENSRNOG00000008098	4	56,114,254
8266	A_64_P114713	-0.095	0.164	5.7E-01	-3.665	0.164	6.6E-13	Agtrap	ENSRNOG00000008619	5	164,898,420
19445	A_44_P250988	-3.804	0.176	3.2E-13	0.117	0.158	4.7E-01	Serhl2	ENSRNOG00000022932	7	123,963,381
13264	A_44_P145970	0.217	0.115	7.8E-02	1.326	0.103	7.6E-10	Hs6st1	ENSRNOG00000014516	9	42,620,006
11810	A_43_P12005	-0.190	0.095	6.3E-02	-1.520	0.085	5.7E-12	Resp18	ENSRNOG00000019704	9	82,477,181
21443	A_44_P748640	0.077	0.102	4.6E-01	1.480	0.091	2.6E-11	Parn	ENSRNOG00000002912	10	1,461,269
17678	A_64_P038142	0.193	0.107	9.1E-02	1.037	0.096	9.7E-09	Tubd1	ENSRNOG00000053309	10	73,868,943
28777	A_64_P028270	0.004	0.179	9.8E-01	1.921	0.160	2.1E-09	Mettl2	ENSRNOG00000006131	10	93,354,003
16173	A_64_P050025	0.028	0.099	7.8E-01	-1.064	0.088	2.0E-09	Cyyr1	ENSRNOG00000001544	11	25,078,740
20991	A_64_P004477	-0.015	0.146	9.2E-01	1.696	0.131	6.9E-10	Adora1	ENSRNOG00000003442	13	51,076,165
5214	A_44_P109862	0.214	0.152	1.8E-01	1.942	0.136	1.6E-10	Ivns1abp	ENSRNOG00000002618	13	68,707,776
10858	A_64_P028371	-0.111	0.120	3.7E-01	-1.487	0.107	2.5E-10	Phf11	ENSRNOG00000053891	15	38,709,984
15751	A_44_P531870	-0.052	0.149	7.3E-01	-3.518	0.134	1.5E-14	Ephx2	ENSRNOG00000017286	15	42,794,279
14880	A_64_P009576	0.128	0.124	3.2E-01	1.290	0.111	3.4E-09	AABR07018792.	ENSRNOG00000038078	15	73,180,671
2879	A_43_P13409	-0.205	0.104	6.6E-02	-1.013	0.093	8.7E-09	Zdhhc2	ENSRNOG00000022686	16	55,002,322
27518	A_44_P1033758	0.146	0.138	3.1E-01	-1.591	0.123	7.7E-10	Wdr36	ENSRNOG00000027355	18	25,637,588
27532	A_44_P252441	0.191	0.161	2.5E-01	1.827	0.144	9.4E-10	St3gal2	ENSRNOG00000017932	19	43,290,363
22427	A_64_P154849	-2.880	0.165	8.0E-12	0.094	0.147	5.3E-01	Agt	ENSRNOG00000018445	19	57,333,433

Disease Models & Mechanisms • Supplementary information

4094	A_64_P132069	0.132	0.104	2.2E-01	1.112	0.093	2.4E-09	RT1-CE15	ENSRNOG00000048951	20	2,707,108
5268	A_44_P852241	0.093	0.082	2.8E-01	1.056	0.074	1.6E-10	none	ENSRNOG00000031780	MT	1
1388	A_43_P11495	0.010	0.137	9.4E-01	2.093	0.123	1.2E-11	Prps2	ENSRNOG00000004160	X	28,435,507
22516	A_64_P072813	0.128	0.155	4.3E-01	3.547	0.155	3.0E-09	no transcript			

Genes are regarded as significant for “non-shared” differential expression when they show $P<1\times10^{-8}$ in one comparison but not in the other (P≥0.05 with a concordant direction or all P-values in the opposite direction).

Table S3H. A list of significant transcripts for non-shared differential expression in the whole kidney.

no	ProbeName	SHR/lzm (vs WKY/lzm) Fold change (log2-scale)	SE (log2-scale)	P-value	SHRSP/lzm (vs WKY/lzm) Fold change (log2-scale)	SE (log2-scale)	P-value	GeneName	Ensembl_rat	Chr	Transcription_start_site (Rnor_6.0)
25	A_42_P520891	-0.873	0.083	9.5E-10	-0.245	0.123	6.0E-02	Prc1	ENSRNOG00000013057	1	142,087,208
5874	A_42_P495598	-1.383	0.114	2.0E-11	-0.277	0.161	9.9E-02	Clrn3	ENSRNOG00000028288	1	207,766,250
5564	A_42_P696282	0.786	0.081	1.4E-09	-0.119	0.114	3.1E-01	Fuom (RGD1311	ENSRNOG00000018476	1	212,568,224
17305	A_64_P002924	-0.954	0.065	4.4E-13	-0.035	0.092	7.1E-01	Asrgl1	ENSRNOG00000020202	1	225,329,770
30101	A_44_P896040	-1.063	0.099	2.2E-10	0.061	0.140	6.7E-01	Cybasc3	ENSRNOG00000020702	1	226,634,009
5085	A_44_P534089	-1.281	0.123	6.0E-10	-0.319	0.167	7.0E-02	Ccnb1	ENSRNOG00000058539	2	30,791,221
20681	A_44_P747071	0.729	0.070	3.8E-10	-0.195	0.099	6.1E-02	Vav3	ENSRNOG00000020485	2	212,247,451
10958	A_44_P480573	-1.006	0.112	5.7E-09	-0.211	0.158	2.0E-01	Cytip	ENSRNOG00000004772	3	44,177,689
23902	A_44_P192224	0.198	0.119	1.1E-01	1.584	0.162	1.9E-09	Upp2	ENSRNOG00000005341	3	44,806,106
16485	A_44_P208138	-1.345	0.100	2.4E-12	0.078	0.141	5.9E-01	F2	ENSRNOG00000016325	3	80,543,031
13942	A_44_P271405	-1.754	0.085	3.0E-16	0.085	0.120	4.9E-01	Pxmp4	ENSRNOG00000016975	3	150,108,898
5160	A_44_P1019604	-1.000	0.100	1.3E-09	-0.205	0.136	1.5E-01	Ube2c	ENSRNOG00000015131	3	161,236,971
17113	A_64_P139590	-0.088	0.065	1.9E-01	1.390	0.092	2.5E-13	Slc35c2	ENSRNOG00000018649	3	161,812,291
26944	A_43_P15431	-1.058	0.083	6.8E-12	-0.001	0.117	9.9E-01	Mterf	ENSRNOG00000007899	4	27,365,376
2691	A_42_P754654	-0.122	0.126	3.4E-01	1.638	0.178	3.8E-09	RGD1562378	ENSRNOG00000045840	4	170,763,916
15950	A_64_P039911	-0.219	0.122	8.5E-02	1.536	0.172	6.8E-09	none	ENSRNOG00000045840	4	170,763,916
8266	A_64_P114713	0.053	0.074	4.8E-01	-3.505	0.104	6.5E-21	Agtrap	ENSRNOG00000008619	5	164,898,420
22457	A_44_P128186	0.207	0.104	5.8E-02	2.236	0.147	1.9E-13	Tas1r1	ENSRNOG00000009708	5	169,212,170
16904	A_64_P134445	-1.544	0.113	1.5E-10	-0.143	0.266	6.0E-01	Tmem179 (RGD1	ENSRNOG00000013128	6	137,084,739
19445	A_44_P250988	-2.969	0.125	1.3E-17	-0.126	0.176	4.8E-01	Serhl2	ENSRNOG00000022932	7	123,963,381
2132	A_64_P136083	-0.132	0.075	9.3E-02	-1.122	0.107	3.2E-10	Itga11	ENSRNOG00000006723	8	67,615,635
22463	A_44_P142850	-0.208	0.109	7.0E-02	1.350	0.155	9.9E-09	Fam86a	ENSRNOG00000002876	10	10,530,365
26992	A_42_P826202	0.065	0.073	3.8E-01	1.734	0.094	1.6E-13	Znf597	ENSRNOG00000007576	10	11,912,543
21202	A_64_P055748	-0.025	0.077	7.5E-01	-1.213	0.109	1.0E-10	Rhbdf1	ENSRNOG00000020594	10	15,672,382
25156	A_64_P064516	-0.014	0.078	8.6E-01	2.798	0.183	2.7E-11	Kcnip1	ENSRNOG00000005365	10	18,579,632
14617	A_64_P028561	1.289	0.100	6.2E-12	0.198	0.142	1.8E-01	none	ENSRNOG00000058024	10	29,806,895
14158	A_44_P1011918	3.148	0.134	1.1E-13	-0.142	0.287	6.3E-01	none	ENSRNOG00000042833	10	91,661,558
28777	A_64_P028270	-0.100	0.145	5.0E-01	2.252	0.205	1.4E-10	Mettl2	ENSRNOG00000006131	10	93,354,003

Disease Models & Mechanisms • Supplementary information

24814	A_64_P010531	0.244	0.134	8.1E-02	-1.955	0.189	4.5E-10	Ace	ENSRNOG00000062101	10	94,170,766
24567	A_43_P17925	0.153	0.085	8.5E-02	-1.136	0.120	2.3E-09	Abca8	ENSRNOG00000004040	10	98,294,522
7468	A_64_P024395	0.205	0.144	1.7E-01	2.789	0.204	1.8E-12	LOC681544	ENSRNOG00000001809	11	81,639,952
8935	A_64_P049423	-1.186	0.100	3.3E-11	0.231	0.142	1.2E-01	Sds	ENSRNOG00000001388	12	41,627,741
28125	A_64_P106543	2.126	0.224	8.1E-09	0.444	0.296	1.5E-01	none	ENSRNOG00000052604	13	56,763,981
20637	A_64_P003621	0.036	0.099	7.2E-01	3.006	0.261	8.6E-09	LOC498308	ENSRNOG00000032812	13	108,460,362
23631	A_64_P013437	1.346	0.152	7.4E-09	0.055	0.214	8.0E-01	Cxcl13	ENSRNOG00000024899	14	15,258,207
5038	A_42_P843692	-2.149	0.094	1.0E-16	-0.177	0.128	1.8E-01	Cadps	ENSRNOG00000060863	15	12,756,403
15751	A_44_P531870	0.137	0.095	1.6E-01	-4.041	0.135	7.9E-20	Ephx2	ENSRNOG00000017286	15	42,794,279
29819	A_64_P101346	-0.124	0.073	1.0E-01	0.991	0.103	1.8E-09	Ppic	ENSRNOG00000017416	18	48,384,645
24729	A_64_P014774	-0.038	0.099	7.0E-01	-1.706	0.158	5.8E-09	Mcm5	ENSRNOG00000014336	19	14,523,554
13133	A_44_P133335	-1.141	0.112	1.0E-09	-0.081	0.153	6.0E-01	none	ENSRNOG00000049468	19	28,645,488
14753	A_64_P023365	-1.511	0.100	2.2E-13	-0.032	0.141	8.2E-01	Abcb10	ENSRNOG00000017993	19	56,772,904
6103	A_64_P174934	-0.704	0.067	3.3E-10	0.131	0.095	1.8E-01	Cog2	ENSRNOG00000018228	19	57,286,985
22427	A_64_P154849	-0.915	0.093	1.2E-09	0.000	0.132	1.0E+00	Agt	ENSRNOG00000018445	19	57,333,433
21684	A_44_P421672	0.071	0.090	4.4E-01	-1.637	0.144	2.5E-09	Ager	ENSRNOG00000000439	20	4,363,152
27477	A_44_P335079	-0.682	0.068	7.6E-10	-0.179	0.096	7.6E-02	Tff3	ENSRNOG00000001159	20	9,855,481
20800	A_44_P545895	-0.068	0.085	4.3E-01	1.192	0.120	9.4E-10		ENSRNOG00000031780	MT	1
24540	A_64_P050969	0.123	0.113	2.9E-01	-1.990	0.160	1.1E-11		ENSRNOG00000031780	MT	1
15913	A_64_P148027	1.081	0.085	5.0E-10	-0.098	0.201	6.3E-01		ENSRNOG00000031780	MT	1
20235	A_44_P472776	0.071	0.101	4.9E-01	-3.018	0.238	5.0E-10	Nr0b1	ENSRNOG00000003765	X	54,734,385
27742	A_64_P085946	-1.149	0.125	6.0E-09	0.353	0.171	5.0E-02	Rtl4	ENSRNOG00000031638	X	116,399,611
22516	A_64_P072813	0.060	0.104	5.7E-01	4.295	0.147	3.4E-15	none_LOC680802			
22795	A_64_P164240	-0.205	0.117	9.2E-02	-1.454	0.165	8.4E-09	LOC301193			
9010	A_64_P012759	-0.913	0.094	1.5E-09	-0.098	0.133	4.7E-01	Fhit	N/A	15	15,697,292

Genes are regarded as significant for “non-shared” differential expression when they show $P<1\times10^{-8}$ in one comparison but not in the other ($P\geq0.05$ with a concordant direction or all P-values in the opposite direction).

Table S3I. A list of significant transcripts for non-shared differential expression in the renal cortex.

no	ProbeName	SHR/lzm (vs WKY/lzm)			SHRSP/lzm (vs WKY/lzm)			GeneName	Ensembl_rat	Chr	Transcription_start_site (Rnor_6.0)
		Fold change (log2-scale)	SE (log2-scale)	P-value	Fold change (log2-scale)	SE (log2-scale)	P-value				
16474	A_64_P068858	-0.067	0.072	3.7E-01	2.501	0.068	2.7E-15	Rps16	ENSRNOG00000019578	1	85,408,444
6002	A_64_P139457	0.009	0.077	9.1E-01	-1.286	0.073	5.9E-11	LOC499124	ENSRNOG00000030932	1	88,361,712
30101	A_44_P896040	-1.204	0.072	1.2E-10	-0.028	0.068	6.9E-01	Cybasc3	ENSRNOG00000020702	1	226,634,009
22220	A_64_P005320	0.062	0.072	4.0E-01	1.066	0.068	3.0E-10	no transcript	ENSRNOG00000005112	1	231,365,651
13942	A_44_P271405	-1.713	0.122	1.3E-09	0.168	0.115	1.7E-01	Pxmp4	ENSRNOG00000016975	3	150,108,898
4481	A_64_P164610	-0.015	0.051	7.8E-01	-0.731	0.048	4.3E-10	Tti1 (RGD15625ε	ENSRNOG00000012880	3	154,490,851
17113	A_64_P139590	-0.075	0.065	2.7E-01	1.151	0.062	2.8E-11	Slc35c2	ENSRNOG00000018649	3	161,812,291
26944	A_43_P15431	-0.959	0.068	1.1E-09	-0.056	0.064	4.0E-01	Mterf	ENSRNOG00000007899	4	27,365,376
2691	A_42_P754654	-0.071	0.117	5.5E-01	1.338	0.110	8.6E-09	no transcript	ENSRNOG00000045840	4	170,763,916
8266	A_64_P114713	-0.072	0.069	3.1E-01	-4.091	0.065	1.4E-18	Agtrap	ENSRNOG00000008619	5	164,898,420
17155	A_64_P051430	1.355	0.105	4.0E-09	-0.034	0.099	7.4E-01	Gpx2	ENSRNOG00000055672	6	99,843,245
19445	A_44_P250988	-2.841	0.080	4.5E-15	-0.010	0.076	8.9E-01	Serhl2	ENSRNOG00000022932	7	123,963,381
18255	A_64_P126165	-0.019	0.072	8.0E-01	-1.077	0.068	2.3E-10	no transcript	ENSRNOG00000046871	9	16,519,144
2629	A_64_P166218	-0.126	0.068	8.6E-02	0.865	0.064	2.2E-09	no transcript	ENSRNOG00000045696	10	1,464,780
26992	A_42_P826202	-0.103	0.138	4.6E-01	2.462	0.130	2.3E-11	Znf597	ENSRNOG00000007576	10	11,912,543
21202	A_64_P055748	-0.067	0.053	2.2E-01	-0.766	0.050	3.8E-10	Rhbdf1	ENSRNOG00000020594	10	15,672,382
14158	A_44_P1011918	2.707	0.084	5.4E-13	0.114	0.091	2.4E-01	no transcript	ENSRNOG00000042833	10	91,661,558
28777	A_64_P028270	0.046	0.081	5.8E-01	1.932	0.076	4.5E-13	Mettl2	ENSRNOG00000006131	10	93,354,003
7468	A_64_P024395	0.179	0.117	1.5E-01	2.841	0.110	3.7E-13	LOC681544	ENSRNOG00000001809	11	81,639,952
5272	A_64_P038050	1.060	0.079	2.4E-09	-0.055	0.075	4.8E-01	Acyp2	ENSRNOG00000042419	14	115,052,450
7829	A_44_P107634	-0.147	0.086	1.1E-01	-1.263	0.081	3.0E-10	Haus4	ENSRNOG00000039284	15	33,193,537
11203	A_64_P139544	-0.273	0.129	5.2E-02	-1.888	0.121	3.2E-10	Scara3	ENSRNOG00000016177	15	42,638,392
15751	A_44_P531870	0.256	0.122	5.5E-02	-3.952	0.115	7.2E-15	Ephx2	ENSRNOG00000017286	15	42,794,279
26808	A_64_P110009	-0.035	0.055	5.4E-01	-0.639	0.052	6.9E-09	Hsd17b4	ENSRNOG00000015840	18	44,810,388
18168	A_44_P213175	0.029	0.088	7.5E-01	-0.998	0.083	9.4E-09	Ccdc68	ENSRNOG00000021381	18	68,408,890
14753	A_64_P023365	-1.415	0.087	1.9E-10	-0.083	0.082	3.3E-01	Abcb10	ENSRNOG00000017993	19	56,772,904
14301	A_43_P16020	0.118	0.231	6.2E-01	3.684	0.212	8.8E-09		ENSRNOG00000031780	MT	1
20800	A_44_P545895	-0.132	0.096	1.9E-01	1.675	0.091	3.4E-11		ENSRNOG00000031780	MT	1
10896	A_44_P140429	-0.085	0.101	4.2E-01	-1.243	0.095	3.3E-09	Asb9	ENSRNOG00000003452	X	31,759,161
9010	A_64_P012759	-0.888	0.066	2.2E-09	-0.062	0.062	3.4E-01	Fhit	N/A	15	15,697,292

Genes are regarded as significant for "non-shared" differential expression when they show $P<1\times10^{-8}$ in one comparison but not in the other (P≥0.05 with a concordant direction or all P-values in the opposite direction).

Table S3J. A list of significant transcripts for non-shared differential expression in the liver.

no	ProbeName	SHR/lzm (vs WKY/lzm)			SHRSP/lzm (vs WKY/lzm)			GeneName	Ensembl_rat	Chr	Transcription_ start_site (Rnor_6.0)
		Fold change (log2-scale)	SE (log2-scale)	P-value	Fold change (log2-scale)	SE (log2-scale)	P-value				
28738	A_64_P053124	-2.258	0.122	1.6E-10	-0.175	0.122	1.8E-01	LOC690948	ENSRNOG00000058538	1	63,964,155
16474	A_64_P068858	0.193	0.101	7.9E-02	2.459	0.101	5.0E-12	Rps16	ENSRNOG00000019578	1	85,408,444
17305	A_64_P002924	-1.299	0.086	1.7E-09	0.173	0.086	6.5E-02	Asrgl1	ENSRNOG00000020202	1	225,329,770
12403	A_44_P398033	0.131	0.083	1.4E-01	2.949	0.083	4.9E-14	Prnp	ENSRNOG00000021259	3	124,515,978
17113	A_64_P139590	0.151	0.085	9.9E-02	4.147	0.085	8.3E-16	Slc35c2	ENSRNOG00000018649	3	161,812,291
21898	A_64_P153502	0.152	0.110	1.9E-01	1.593	0.110	2.9E-09	Armc9	ENSRNOG00000025418	9	93,172,673
26992	A_42_P826202	0.152	0.110	2.0E-01	2.439	0.110	3.0E-10	Znf597	ENSRNOG00000007576	10	11,912,543
28777	A_64_P028270	-0.206	0.156	2.1E-01	2.826	0.156	1.9E-10	Mettl2	ENSRNOG00000006131	10	93,354,003
24567	A_43_P17925	0.017	0.122	8.9E-01	-1.651	0.122	6.4E-09	Abca8	ENSRNOG00000004040	10	98,294,522
20334	A_44_P368461	0.109	0.127	4.1E-01	-2.465	0.127	8.8E-11	Cpn2	ENSRNOG00000024384	11	74,057,361
11763	A_64_P031741	0.116	0.097	2.5E-01	-1.287	0.097	8.7E-09		ENSRNOG00000031780	MT	1
24540	A_64_P050969	-0.036	0.119	7.7E-01	-2.152	0.119	2.0E-10		ENSRNOG00000031780	MT	1

Genes are regarded as significant for “non-shared” differential expression when they show $P<1\times10^{-8}$ in one comparison but not in the other (P≥0.05 with a concordant direction or all P-values in the opposite direction).

Table S4A. A list of significant genes for shared differential expression in any of target tissues on RNO1.

Gene	Agilent probe	Pos. on RNO1 (Rnor_6.0)	Heart			Aorta			Renal cortex			Kidney		Liver		
			D/E	SHR log2(FC)	SHRSP log2(FC)	D/E	SHR log2(FC)	SHRSP log2(FC)	D/E	SHR log2(FC)	SHRSP log2(FC)	SHR log2(FC)	SHRSP log2(FC)	D/E	SHR log2(FC)	SHRSP log2(FC)
Lrp11	A_44_P973511	1,702,696	●	-1.10	-1.37		-0.22	0.03		**-0.26**	-0.18	-0.09	-0.19	●	-1.10	-1.37
Samd5	A_64_P063358	3,763,392		N/A	N/A		N/A	N/A	●	1.03	1.52	1.04	1.59		N/A	N/A
Shprh	A_44_P1025861	5,240,180		0.24	0.03	○	**0.53**	**0.72**	●	0.69	0.72	0.63	**0.41**		0.01	**0.20**
Epm2a	A_44_P452186	5,448,958		0.27	0.12	●	1.47	1.40		**-0.33**	0.21	-0.06	-0.17		0.02	-0.26
Stx11	A_44_P101700	7,064,870	●	0.60	0.37		0.57	**0.99**		N/A	N/A	N/A	N/A		N/A	N/A
Arfgef3 (RGD1309903)	A_64_P165046	13,915,594		N/A	N/A		0.30	0.56	●	-0.56	-0.52	-0.02	-0.07		0.10	0.15
Pex7	A_44_P445359	15,374,850	●	0.65	0.47	●	**0.24**	0.59		0.11	0.15	0.09	**0.34**	○	**0.45**	**0.53**
Map3k5 (RGD1306565)	A_64_P129293	15,412,603	●	1.42	1.47	●	2.18	1.71	●	1.27	1.25	1.42	1.85	○	**1.79**	**1.74**
Aldh8a1	A_44_P128306	16,910,069		N/A	N/A		N/A	N/A	●	0.37	0.53	0.45	0.58	●	0.82	0.89
Ptprk	A_43_P23129	18,058,055	●	-0.33	-0.45	●	-0.68	-0.83	○	**-0.29**	**-0.31**	-0.16	-0.23		-0.23	0.01
Enpp1	A_43_P12841	21,748,261	●	0.50	0.25	●	**0.40**	0.71		-0.09	0.09	0.02	0.17	○	**0.32**	**0.56**
Ctgf (Ccn2)	A_42_P484738	21,854,773	●	1.00	1.28	●	1.44	2.61	●	**0.67**	**0.57**	**0.45**	1.09		**-0.71**	**0.37**
Slc18b1 (RGD1306962)	A_44_P353836	22,748,422		-0.71	-0.95		**-0.67**	-0.03	●	-0.81	-0.64	-0.85	**-0.73**		-0.24	-0.05
Slc12a7	A_64_P121575	32,140,137		-0.01	**0.27**	●	-1.03	-0.57		-0.10	-0.15	-0.06	0.12		-0.06	-0.11
Slc6a19	A_64_P139942	32,199,810		N/A	N/A	●	-1.44	-1.33		0.15	-0.02	0.14	0.01		N/A	N/A
Srd5a1	A_44_P182431	36,320,461		-0.36	0.12	●	**-0.42**	-1.33		0.81	0.01	-0.03	-0.14	●	-2.25	-2.88
Sod2	A_64_P110103	47,921,587		0.05	-0.06	●	-0.41	-0.58		0.04	0.11	0.09	0.16		0.08	**0.27**
LOC684270	A_64_P119169	72,509,431	●	-0.33	-0.34		**0.48**	0.00		0.01	0.03	-0.05	-0.02		0.11	0.12
Tnnt1	A_64_P058871	72,889,278	●	0.52	0.42		**-1.70**	0.34		**-0.53**	0.25	**-0.59**	**0.69**		**-0.46**	**0.17**
AABR07002623.1	A_64_P162940	77,782,825		N/A	N/A		N/A	N/A	●	-1.80	-1.38	-1.83	**-0.77**		0.29	-0.34
Tmem160	A_64_P107642	78,417,719		**0.20**	**0.14**	●	-0.41	-0.62	○	**-0.22**	**-0.15**	**-0.29**	-0.06		0.20	0.22
Bloc1s3	A_64_P042455	80,416,273		0.18	0.09	●	-0.57	-0.70		-0.02	-0.04	**-0.27**	0.12		0.06	0.08
Apoc2	A_42_P689905	80,594,136		-0.03	-0.20		**3.14**	-0.17	●	1.58	2.16	1.27	1.63		-0.11	**-0.33**
LOC100359930	A_64_P164736	82,653,633		N/A	N/A		N/A	N/A		N/A	N/A	N/A	N/A	●	1.40	1.92
Snrpa	A_42_P641234	84,008,293		-0.30	**0.26**	●	-0.63	-0.54		-0.07	**-0.23**	**-0.32**	-0.17		-0.10	-0.08
Itpkc	A_44_P156211	84,040,433	●	0.50	0.33		-0.03	**0.44**		0.01	-0.01	-0.11	0.29	○	**0.49**	**0.38**
Sptbn4	A_64_P125676	84,254,645		0.35	0.01	●	-1.11	-1.48		**0.68**	**-0.53**	0.29	0.14		N/A	N/A
Dyrk1b	A_64_P131903	85,112,247		**-0.31**	0.01	●	-0.67	-0.56		**-0.33**	-0.26	**-0.24**	0.02		-0.24	-0.01
Eid2	A_42_P554610	85,517,360	○	**-0.26**	-0.38	●	-0.74	-1.12		**-0.16**	0.06	-0.20	0.07		-0.23	**-0.31**
Fbxo17	A_64_P027503	86,914,137	●	0.88	0.47		0.09	0.01		0.16	0.25	0.06	-0.25		0.41	0.09
Yif1b	A_64_P079807	87,180,624		**-0.36**	0.01	●	-0.43	-0.50		**-0.22**	0.00	**-0.27**	-0.12		**0.32**	-0.02
Tmem149	A_64_P063428	89,020,341		0.27	**-0.18**	●	-1.03	-1.32		**-0.23**	0.10	-0.27	0.32		-0.04	-0.34
LOC688869	A_64_P078982	89,084,859		0.04	0.03	●	-0.66	-1.19		0.003	0.09	**-0.25**	-0.22		0.16	-0.03

Disease Models & Mechanisms • Supplementary information

Atp4a	A_64_P129593	89,162,639		0.02	0.10		-0.16	0.26		-0.06	-0.10	0.09	-0.09	●	-1.57	-1.59
Lsr	A_64_P060303	89,399,041		-0.59	0.04	●	-0.75	-1.10	○	**-0.15**	**-0.20**	**-0.28**	0.16	○	**-0.36**	**-0.24**
Lgi4	A_44_P1018090	89,491,654	●	0.44	0.42	●	**0.31**	0.83		N/A	N/A	N/A	N/A		N/A	N/A
LOC365238	A_44_P449994	94,784,451	●	0.98	0.80	●	**0.26**	0.74	○	**0.54**	**0.72**	-0.02	0.51		**0.35**	0.13
LOC499136	A_44_P515321	97,785,490		-0.22	0.11	●	-0.52	-0.56		-0.14	-0.09	**-0.35**	0.05		0.25	-0.09
Lim2	A_64_P148550	98,501,249	●	0.60	0.73	●	-1.31	-2.26		N/A	N/A	N/A	N/A		N/A	N/A
LOC102556967	A_64_P080941	99,505,677	●	-1.55	-1.66		**-1.07**	-0.24	●	-1.20	-0.96	-1.27	-1.46	●	**-0.90**	-1.19
Klk1c10	A_44_P280954	99,894,353		N/A	N/A		N/A	N/A	●	-1.03	-1.44	-1.18	N/A		N/A	N/A
Klks3	A_44_P163243	100,059,967		N/A	N/A		N/A	N/A	●	-1.41	-2.71	-1.60	-2.09		N/A	N/A
(Klk4 low expression)	A_44_P989658	100,205,147		**0.42**	-0.04		-0.04	0.26	●	1.26	1.12	0.76	**0.96**	●	2.85	2.00
Lin7b	A_44_P215157	101,360,971		-0.24	-0.01	●	-1.31	-2.12		N/A	N/A	-0.60	N/A		N/A	N/A
Bcat2	A_64_P166076	101,554,642		-0.19	**-0.18**	●	1.47	0.93		0.05	**0.26**	-0.08	**0.31**		0.26	**0.65**
Dbp	A_64_P017188	101,687,855	●	-1.48	-0.95		1.65	0.65		-1.16	0.13	-0.39	-0.45		-0.54	-0.32
Sphk2	A_44_P466271	101,697,277	●	0.42	0.21		**-0.41**	0.04		0.12	-0.14	-0.09	0.04		-0.24	-0.07
Ldha	A_64_P016403	102,900,286		0.005	-0.001	●	-0.72	-1.07	●	0.68	0.32	0.30	**0.28**		**-0.33**	**-0.59**
Tmem86a	A_43_P11305	103,172,987	○	**-0.53**	**-0.26**		0.41	-1.25	●	-1.94	-0.94	-0.88	**-0.62**		0.11	0.15
Ptpn5	A_42_P506076	103,256,823	●	1.28	0.67		N/A	N/A	●	**0.50**	N/A	1.33	**1.42**		N/A	N/A
Zdhhc13	A_44_P250476	104,106,245		0.21	**0.30**		-0.14	0.14	●	-0.93	-0.71	-1.05	**-1.02**		-0.05	0.04
Csrp3	A_44_P997843	104,157,855	●	0.61	0.62		0.71	0.05		N/A	N/A	N/A	N/A		N/A	N/A
Prmt3	A_44_P536125	105,113,595	●	0.84	0.41		0.01	**0.51**		0.09	0.15	-0.06	0.24		N/A	N/A
Fancf	A_64_P151448	107,232,305	●	-0.71	-0.59		-0.98	-0.06	●	-0.97	-0.74	-1.08	**-0.60**	●	-0.96	-0.91
Bex1	A_42_P602570	116,567,189	●	2.25	0.88		0.89	0.99	●	0.61	**0.27**	**0.37**	**0.40**		-0.28	-0.20
Aldh1a3	A_44_P273783	127,337,882		N/A	N/A		0.07	**0.71**	●	-0.77	-0.88	-0.60	**-0.54**		N/A	N/A
Slco3a1	A_44_P496255	136,073,540		0.22	0.31		0.30	1.13	●	0.58	0.51	**0.52**	**0.51**		N/A	N/A
Ntrk3	A_64_P035162	140,262,503		0.48	**0.37**		**0.62**	-0.01	●	1.08	1.22	**0.90**	1.90		N/A	N/A
Mrps11	A_44_P1046705	140,477,868		-0.18	0.14	●	-0.59	-0.84		-0.01	0.09	**-0.30**	0.01		0.03	-0.05
Sema4b	A_64_P133177	141,986,145	●	-0.68	-1.15	●	-0.88	**-0.46**	●	-0.80	-0.68	-0.91	-1.13	●	-0.72	-1.01
Ngrn	A_43_P10751	142,050,458	●	0.47	0.29		-0.17	**0.53**		**-0.19**	-0.04	**-0.27**	0.09	○	**-0.38**	**-0.39**
Hddc3	A_42_P528580	142,136,452	●	-0.34	-0.30	●	**-0.22**	-0.78	○	0.05	-0.02	**-0.15**	**-0.21**		-0.30	**-0.43**
Nmb	A_64_P109232	142,724,511	●	**-0.47**	-0.75		1.25	0.89	●	-2.31	-2.12	-2.13	-2.70		0.41	0.34
Homer2	A_64_P016059	143,535,583	●	-1.15	-0.58	●	-1.62	-1.54	●	-1.18	-1.09	-0.78	**-0.59**		-0.22	-0.13
Eftud1	A_64_P045536	144,601,410	●	-0.37	-0.34		-0.08	-0.06	○	**-0.32**	**-0.18**	**-0.19**	**-0.36**		-0.05	0.15
Cyp2c7	A_64_P000452	147,435,897		N/A	N/A		N/A	N/A		N/A	N/A	N/A	N/A	●	-0.74	-0.66
Nox4	A_44_P154040	150,797,084		N/A	N/A	○	**1.33**	**1.01**		**-0.37**	-0.10	-0.15	-0.20	●	2.09	1.55
Prss23	A_42_P628981	153,752,541	●	0.54	0.81		0.43	1.56		-0.02	-0.01	-0.09	-0.08		0.02	0.16
Aamdc (RGD1561459)	A_64_P084767	162,533,893	○	**-0.26**	**-0.13**	●	-0.56	-0.45		-0.06	0.04	**-0.27**	-0.09		**0.27**	0.10
Dgat2	A_42_P733209	164,143,818	●	-0.66	-0.38		**0.48**	**0.58**		**0.22**	0.14	-0.08	**0.30**		-0.02	0.09
Ucp2	A_44_P301608	165,506,361	●	0.72	1.13	○	**0.77**	**0.77**		0.08	**0.31**	-0.14	0.28		0.24	**0.58**

Disease Models & Mechanisms • Supplementary information

Mrpl48	A_43_P14847	165,606,375	●	-0.54	-0.35		0.01	**-0.30**	○	**-0.12**	**-0.12**	**-0.19**	0.05		-0.03	0.03
Plekhb1	A_64_P028140	165,680,206		0.08	0.22	●	0.86	0.60	●	-0.66	-0.73	-0.68	**-0.57**		0.23	**-0.30**
Fam168a (RGD1308929)	A_44_P975945	165,724,451	●	**0.56**	0.58	○	**0.42**	**0.70**	●	**0.34**	0.56	**0.39**	**0.71**	●	1.13	1.24
P2ry2	A_44_P520159	166,037,424	●	-0.51	-0.38	○	**0.46**	**0.63**	●	-0.68	-0.67	**-0.76**	-0.12	●	**-1.09**	-1.29
Folr2	A_42_P788740	166,919,302		0.13	0.62		-0.29	**0.61**	●	0.99	0.75	0.54	0.99	○	**0.42**	**0.55**
Anapc15 (RGD1311634)	A_44_P177931	166,983,175		0.13	0.40	●	-0.67	-0.62		-0.07	0.10	**-0.26**	0.08		0.05	0.09
Lrrc51	A_43_P22401	167,005,839	●	0.50	0.45		0.18	**0.27**		0.02	**0.26**	-0.19	0.30	○	**0.65**	**0.83**
Art1	A_64_P162476	167,197,549	●	-0.93	-1.28		0.05	-0.21		N/A	N/A	N/A	N/A		N/A	N/A
Art5	A_64_P042586	167,202,767	●	-0.95	-1.01		-0.34	0.62		N/A	N/A	N/A	N/A		N/A	N/A
Olr59	A_64_P072633	167,911,961	●	-0.54	-1.25		**-0.80**	-0.59	○	**-0.42**	**-0.34**	0.25	-0.42		0.03	0.00
Olr200	A_64_P112431	170,147,300	●	1.08	0.68		**1.02**	0.28	○	**0.57**	**0.77**	0.29	0.66		N/A	N/A
Fam160a2	A_44_P398300	170,238,890	○	**0.45**	**0.16**		0.22	**0.26**	●	0.50	0.42	**0.35**	0.16		-0.25	-0.06
Dnhd1	A_64_P043101	170,471,272		-0.17	**-0.39**		0.47	0.96	○	**-0.36**	**-0.49**	-0.06	-0.18	●	-0.83	-0.98
Akip1 (RGD1306959)	A_42_P797965	174,330,695	●	1.69	1.36	●	**0.55**	0.93	○	**0.23**	**0.24**	0.13	**0.35**	●	0.54	0.50
Mrvi1	A_64_P092754	175,796,040		N/A	N/A	●	1.27	1.23		N/A	N/A	N/A	N/A		N/A	N/A
Rras2	A_64_P086565	179,010,257	●	0.62	0.64	●	**0.42**	0.71	●	0.37	**0.30**	**0.18**	0.13		0.10	0.02
Acsm5	A_64_P151353	189,241,593		N/A	N/A	●	1.13	0.78	●	1.89	2.03	1.76	2.15	●	3.03	3.70
Acsm3	A_43_P15937	189,514,553		N/A	N/A		-0.05	0.60	●	**0.31**	0.55	0.43	0.70		0.25	**0.31**
LOC378467	A_42_P793598	189,870,622	●	0.47	0.25		-0.03	0.11		-0.09	**-0.15**	**-0.20**	0.03		-0.24	**-0.52**
Crym	A_43_P12996	189,960,073	●	1.13	0.65		-0.04	-0.11		**0.22**	0.10	**0.14**	-0.10	●	0.82	**0.51**
LOC691551	A_64_P019021	190,914,946	●	2.22	1.19	●	0.95	1.11		-0.30	0.32	-0.10	0.21		N/A	N/A
Jmjd5	A_42_P791460	196,839,321	●	-0.34	-0.38		-0.06	-0.17		0.09	0.08	-0.03	-0.03		-0.25	**-0.09**
Il4ra	A_64_P089531	196,942,364	●	0.67	0.61		0.27	-0.02		**-0.49**	-0.09	**-0.50**	0.25	○	**0.66**	**0.55**
Sbk1	A_64_P024138	197,659,187		-0.04	-0.03	●	-6.08	-6.81	●	-0.58	**-0.30**	**-0.54**	-0.17	●	-1.46	-0.92
Rabep2	A_44_P395538	197,839,430	●	-0.83	-0.52		-0.11	0.02	●	-0.40	-0.29	-0.39	-0.04		-0.20	-0.03
Eif3c	A_44_P321110	197,982,601	●	-0.32	-0.29	○	**-0.24**	**-0.22**		**-0.12**	-0.07	-0.13	-0.14		**-0.27**	0.05
Apob48r	A_44_P562972	197,999,037	●	0.52	0.84	○	**0.38**	**0.42**	○	**0.61**	**0.57**	**0.55**	**0.98**		0.31	**0.49**
Nupr1	A_42_P812008	198,045,154	●	-0.96	-0.34		-0.16	**0.80**	○	**-0.89**	**-0.58**	**-0.38**	0.29		-0.25	-1.15
Sult1a1	A_64_P011779	198,104,109		0.07	-0.45	●	**0.79**	1.10	●	0.70	0.66	**0.41**	**0.59**		0.15	0.13
Ino80e (Ccdc95)	A_44_P1002996	198,298,076	●	-0.79	-0.87	●	-0.77	**-0.55**	●	-0.52	-0.53	-0.53	**-0.46**	○	**-0.30**	**-0.36**
Mvp	A_44_P275029	198,450,047	●	-0.37	-0.22	●	-1.29	-1.00		0.13	-0.06	0.02	-0.24		-0.12	0.09
Pagr1 (RGD1305592)	A_44_P1029472	198,450,688	●	-0.91	-0.43	●	-1.90	-1.37	●	-0.53	-0.36	-0.49	-0.20		-0.05	0.05
Qprt	A_42_P505241	198,559,568	●	**0.72**	0.79	●	2.11	1.30		-0.07	0.05	-0.08	-0.20		0.01	**-0.33**
Mylpf	A_44_P1005462	198,655,742		-0.28	0.15		-1.01	**-2.56**	●	1.46	1.09	1.21	1.33		0.12	**0.37**
Dctpp1 (Xtp3tpa)	A_44_P285803	198,706,852		-0.16	0.02	●	-0.66	-0.67	●	**-0.37**	-0.14	-0.50	**-0.37**		-0.03	-0.23
Itgal	A_44_P452222	198,744,050		-0.02	0.87		0.47	**0.88**	●	0.80	0.53	2.15	1.49		-0.10	N/A

Disease Models & Mechanisms • Supplementary information

Znf688	A_44_P473234	198,869,009	●	0.77	0.89		-0.14	0.14		0.08	**0.26**	-0.12	0.18		0.14	0.26
Rnf40	A_64_P045578	199,037,544	●	1.67	1.30	●	1.87	2.03	●	1.84	1.94	1.12	1.13	●	1.18	1.25
Orai3	A_64_P019325	199,217,016	●	0.93	0.79		0.02	0.05		-0.19	-0.14	**-0.22**	-0.06		-0.08	**-0.23**
Bckdk	A_42_P834903	199,351,628		-0.11	**0.13**	●	-1.01	-0.53		-0.44	-0.05	**-0.32**	0.06		**-0.62**	-0.31
Fus	A_64_P056569	199,412,834		-0.11	-0.44		0.41	**0.28**		-0.07	**-0.20**	-0.02	**-0.46**	●	-0.77	-0.78
RGD1310127	A_64_P080856	199,716,205	●	0.33	0.47	●	**0.30**	0.52		0.07	**0.13**	**0.19**	0.19		0.16	0.05
Sec23ip	A_44_P252921	200,167,169	●	-0.34	-0.38		**-0.31**	-0.06	●	-0.54	-0.50	-0.59	**-0.52**	○	**-0.53**	**-0.39**
Htra1	A_42_P784614	201,499,028		**-0.22**	0.00		-0.07	1.02	●	0.52	0.58	0.54	0.78		0.20	-0.04
Cpxm2	A_42_P740370	204,087,001	●	0.69	0.43	○	**0.83**	**0.85**	●	**-0.91**	**-0.70**	**-0.46**	-0.26		N/A	N/A
Cyp2e1	A_44_P409232	213,511,874		0.60	**-0.65**	●	3.46	1.56	●	1.35	**0.53**	0.98	**0.43**		**0.45**	0.34
Scgb1c1	A_64_P036285	213,595,240		**0.69**	0.04	●	-1.76	-1.42		-0.32	-0.01	**-0.44**	-0.46		N/A	N/A
Chid1	A_64_P153870	214,511,529	●	0.75	0.46		**-0.23**	-0.46		**0.41**	0.09	**0.22**	0.20		0.82	0.16
Tnni2	A_42_P718022	215,609,036		**1.15**	0.43		0.79	-1.13		0.44	-0.03	-0.03	0.36	●	1.32	1.32
Fadd	A_64_P097784	217,748,628	●	0.79	0.56		-0.28	-0.04		0.77	0.22	0.34	0.41		N/A	N/A
Ano1 (Tmem16a)	A_42_P495579	217,844,957		**0.27**	-0.08	●	0.86	1.41		**-0.30**	-0.03	**-0.50**	0.02		-0.09	-0.02
Gal	A_42_P614175	218,657,925	●	4.25	1.34	●	1.14	1.96		-0.05	0.14	0.03	-0.03		N/A	N/A
Ndufs8	A_44_P132522	219,144,610		0.06	-0.07	●	-0.74	-0.69		-0.32	-0.12	**-0.18**	0.01		0.07	-0.15
LOC689065	A_64_P119314	219,745,654		**-0.51**	-0.04	●	-1.69	-2.44		**-0.50**	**0.53**	**-0.51**	**0.98**	●	-3.23	-3.57
Bbs1	A_44_P635869	220,165,678		**0.37**	0.01	●	1.11	0.81		**0.40**	**-0.24**	**0.50**	0.09		0.33	-0.08
Rab1b	A_64_P051802	220,491,469		-0.03	0.06	●	-0.59	-0.41		**-0.19**	-0.16	-0.14	0.09		-0.16	0.22
Cst6	A_64_P150876	220,729,000		-0.89	**0.23**	●	-0.92	-1.98		**-0.38**	0.12	**-0.64**	0.26		0.08	0.15
Ccdc85b	A_44_P367971	220,836,504	●	-0.44	-0.29		**0.31**	0.48		-0.47	-0.04	**-0.38**	0.19		**-0.26**	-0.17
Ctsw	A_44_P107596	220,848,153		N/A	-0.24		-0.60	**-0.88**	●	-1.19	-1.27	-1.35	-0.17		N/A	N/A
Sf1	A_64_P053322	221,735,517		**0.25**	0.01	●	0.44	0.46		-0.04	0.11	0.01	0.03		-0.02	-0.05
Esrra	A_42_P780882	222,178,725	●	**-0.11**	-0.17	●	-0.47	-0.45		0.15	0.03	**0.14**	0.03		-0.11	0.05
Ppp1r14b	A_64_P193845	222,229,835		0.13	0.33	●	-0.57	-0.91		0.09	0.02	-0.05	0.03		0.04	**0.34**
Slc22a9	A_64_P031501	222,975,509		N/A	N/A		N/A	N/A	●	**-1.47**	**-0.65**	-0.91	-0.68		N/A	N/A
AABR07006160.1	A_64_P029622	223,549,904	●	-0.42	-0.40		0.13	0.11	○	-0.14	-0.10	**-0.20**	**-0.26**		**-0.34**	-0.13
Ust5r	A_44_P228859	224,533,219		N/A	N/A		N/A	N/A		0.10	0.07	0.05	0.00	●	0.93	0.84
Bscl2	A_64_P128124	225,037,737	●	-0.32	**-0.10**	●	-0.70	-0.91	○	-0.07	0.02	**-0.15**	**-0.29**		-0.07	0.18
LOC690344	A_44_P944998	225,077,079	●	-0.49	0.04	●	-0.75	-1.00		0.01	0.04	**-0.34**	-0.07		**0.28**	**0.20**
B3gat3	A_44_P532027	225,120,061		-0.08	0.21	●	-0.75	-0.68		0.00	0.05	**-0.18**	0.06		0.06	0.12
Tut1	A_42_P651632	225,151,404		-0.06	**-0.19**	●	0.68	1.10		**-0.15**	-0.04	-0.07	0.02		0.00	0.02
Fads3	A_44_P1012567	226,091,774		0.00	**0.20**	●	**0.73**	0.87		0.17	-0.08	0.07	0.05	●	2.49	1.82
Dagla	A_64_P033447	226,353,611		-0.09	**-0.33**	●	-0.87	-0.88		**0.18**	0.45	0.01	0.11		N/A	N/A
Sdhaf2 (RGD1309216)	A_64_P155741	226,572,349	●	-0.56	-0.19		**-0.40**	-0.25		**-0.12**	0.05	-0.33	-0.11		**-0.27**	-0.01
Plac1l (Oosp2)	A_64_P073303	228,014,924		N/A	N/A		N/A	N/A	●	**-1.02**	-1.68	-0.69	-1.96		N/A	N/A
AABR07006542.1	A_64_P013243	239,319,737	○	**-0.31**	**-0.17**	○	**-0.29**	**-0.49**		-0.10	-0.02	0.00	-0.17	●	-1.71	-1.85

Aldh1a7	A_44_P335974	240,601,744		N/A	N/A	●	1.54	1.39		0.12	1.04	0.21	0.26		-2.26	**-1.25**
RGD1560242	A_64_P146165	241,460,868	●	1.25	1.54		N/A	N/A		N/A	N/A	N/A	N/A		N/A	N/A
Pgm5	A_64_P081982	242,765,807		**-0.29**	0.01		0.53	1.84	●	-1.21	-0.95	-1.02	**-0.64**		**-0.61**	-0.12
Kank1 (Ankrd15)	A_44_P315626	243,276,403	●	0.45	0.26	●	**0.61**	1.21		0.13	-0.07	0.12	0.21		-0.15	-0.11
Il33	A_44_P310596	248,132,090	●	-0.71	-0.52		-0.10	**0.35**	○	**-0.43**	**-0.30**	**-0.37**	**-0.55**	●	-1.31	**-1.10**
Ankrd1	A_44_P284753	254,735,548	●	1.04	0.61		-0.38	2.28		-0.11	-0.10	-0.14	0.33		N/A	N/A
Ppp1r3c	A_64_P071983	255,376,833	●	0.66	**0.21**		-0.19	-1.33	○	**0.29**	**0.27**	-0.25	-0.16	●	1.44	1.53
Hhex	A_43_P12437	256,101,903	●	-0.60	-0.31		0.24	**-0.53**		-0.15	**0.23**	-0.19	0.00		0.19	**0.94**
Cyp2c12	A_44_P139673	258,766,881		N/A	N/A		N/A	N/A		N/A	N/A	N/A	N/A	●	-1.54	-2.91
Rrp12 (LOC679127)	A_64_P082037	261,140,389	●	0.47	0.28		0.13	**0.40**		0.16	0.04	0.03	0.13		-0.10	0.24
Ankrd2	A_44_P262593	261,281,543	●	2.50	1.91	●	-1.61	-1.75		N/A	N/A	0.12	-0.20		N/A	N/A
Bloc1s2	A_42_P644278	263,920,962	●	-0.88	-0.80	●	-1.15	-1.00	●	**-0.32**	**-0.32**	-0.49	**-0.50**	○	**-0.68**	**-0.75**
Pkd2l1	A_64_P062648	263,959,318	●	N/A	N/A	●	-1.17	-0.85		N/A	N/A	**-1.49**	0.38		N/A	N/A
Ndufb8	A_42_P751152	264,303,762		-0.07	-0.09		0.03	-0.13	●	-0.49	**-0.37**	-0.38	-0.38		**-0.20**	-0.13
Kazald1	A_44_P480433	264,827,739	●	-0.61	**-0.20**		**-0.78**	0.22	●	-1.53	-0.98	-1.34	**-0.61**	○	**-0.60**	**-0.76**
Poll	A_64_P143725	265,298,797	●	**0.23**	0.47		0.06	0.03	●	**0.22**	0.37	0.12	0.59	●	0.55	0.70
Fbxw4	A_44_P325439	265,420,503	●	0.49	0.37		**-0.36**	**0.21**		-0.29	-0.34	-0.13	-0.10		-0.17	-0.09
Npm3	A_64_P129182	265,506,046		-0.02	-0.75	○	**-1.17**	**-0.97**	●	-1.13	-0.80	-1.13	**-0.69**		-0.24	0.18
Trim8	A_64_P017268	266,255,797		-0.24	-0.05	●	-1.27	-1.33	○	**-0.66**	**-0.32**	**-0.58**	-0.37		-0.20	-0.24
RGD1311783	A_42_P759043	266,451,021	●	1.03	1.03	○	**-0.23**	**-0.37**	○	**-0.39**	**-0.31**	**-0.49**	**-0.34**		-0.22	**-0.34**
LOC100364597	A_44_P995874	266,782,910	●	0.37	0.37		0.24	0.67		**0.19**	0.07	0.12	**0.30**		-0.15	0.11
Pdcd11	A_44_P990043	266,866,931	●	0.58	0.40	●	0.71	1.01	○	**0.23**	0.13	**0.30**	**0.38**		0.05	**0.41**
Obfc1	A_42_P462015	267,315,729	●	-0.58	-0.24		0.10	0.07	●	**-0.25**	**-0.25**	-0.54	**-0.42**		-0.11	**-0.36**
Sorcs1	A_64_P043941	270,472,866		-0.25	0.07		-0.17	0.07	●	-1.75	-1.34	-1.46	-1.51		N/A	N/A
Mxi1	A_64_P066276	274,030,978		**0.17**	**-0.08**		0.38	0.12	●	-0.97	-0.75	-1.00	-0.72		**0.72**	**0.60**
Dusp5	A_42_P739860	274,245,184	●	0.66	1.42	●	2.76	1.91	●	0.27	**0.71**	0.92	**1.02**		0.07	-0.24
Nrap	A_64_P026191	277,181,414	●	0.58	0.29		0.18	**1.29**		N/A	N/A	N/A	N/A		N/A	N/A
Casp7	A_44_P183488	277,190,964	●	-0.48	-0.16		0.09	**0.28**	●	0.49	0.63	**0.39**	**0.54**		0.08	0.13
Vwa2	A_64_P087184	277,689,729		0.34	0.08		0.11	-0.24	●	**0.55**	0.99	0.70	0.76		**0.97**	0.52
AABR07007032.1	A_44_P240330	277,867,630		-0.04	**-0.13**	●	-0.72	-1.13		0.08	0.08	0.03	-0.02		N/A	N/A
Trub1	A_44_P403005	278,311,244	●	-0.77	-0.60		-0.26	-0.08	●	-0.74	-0.73	-0.88	**-0.88**	○	**-0.97**	**-0.81**
Gfra1	A_44_P107308	279,277,339		-0.24	-0.40		**-0.83**	-0.35		-0.13	0.16	-0.28	-0.30	●	2.14	2.09

Fold changes (hypertensive strain vs WKY; $\log_2$-transformed values) are shown in each cell. Significant values are underlined ($P<1\times10^{-4}$) or in bold letter ($1\times10^{-4}\leq P<0.05$).

Significant genes are initially selected for "shared" differential expression (D/E) when they show $P<10^{-4}$ reproducibly in two types of inter-strain comparison, ie, SHRSP/Izm versus WKY/Izm and SHR/Izm versus WKY/Izm, with a concordant direction of D/E in a primary target tissue. Then, to validate D/E of the corresponding genes in tissues other than the primary one, we set suggestive and significant evidence for shared D/E. We consider it as suggestive (open circle in the column D/E) when both types of comparison show $P<0.05$; we claim it to be significant (closed circle in the column D/E) when $P<10^{-4}$ is further attained for at least one type of comparison (ie, $P<0.05$ for the other type).

In some genes, the direction of differential expression is opposite to that in the primary tissue. Some genes are not included in the list when there is no appropriate transcript for the microarray probes annotated.

Table S4B. A list of significant genes for non-shared differential expression in any of target tissues on RNO1.

Gene	Agilent probe	Pos. on RNO1 (Rnor_6.0)	Heart			Renal cortex			Kidney		Liver			Aorta		
			D/E	SHR log2(FC)	SHRSP log2(FC)	D/E	SHR log2(FC)	SHRSP log2(FC)	SHR log2(FC)	SHRSP log2(FC)	D/E	SHR log2(FC)	SHRSP log2(FC)	D/E	SHR log2(FC)	SHRSP log2(FC)
Ust	A_64_P147549	2,627,747	●	-0.11	-0.39		0.18	0.17	-0.03	-0.01		0.17	-0.08		0.16	**0.37**
Fbxo30	A_42_P547213	5,366,379	●	0.13	0.41		0.10	0.10	0.00	0.04		0.15	0.30		**0.64**	0.30
Hbs1l	A_44_P255294	16,819,170	●	0.06	0.26		**-0.21**	**-0.30**	**-0.28**	**-0.27**		-0.04	-0.14		**-0.19**	**0.34**
Trdn	A_44_P191715	25,839,198	●	-0.18	-0.68		N/A	N/A	N/A	N/A		N/A	N/A		0.02	0.49
Irx1	A_64_P086235	33,910,912	●	0.28	-0.67		-0.20	-0.02	0.09	-0.14		**-0.91**	**-0.73**		-0.29	**-0.46**
Ube2ql1	A_64_P042302	36,185,916	●	-0.09	-0.52		0.12	**0.59**	0.09	-0.24		N/A	N/A		0.42	0.63
Afdn (Mllt4)	A_44_P794669	53,802,658	●	0.09	-0.33		**0.18**	0.00	**0.26**	0.06		-0.08	0.01		0.02	-0.18
LOC690948	A_64_P053124	63,964,155		0.26	0.26		**0.55**	-0.06	0.20	0.08	●	-2.26	-0.18		-0.10	-0.30
Ndufa3	A_64_P150067	64,175,099		0.21	0.11		0.10	-0.09	0.08	0.00		**0.39**	**0.18**	●	-0.06	-0.76
Irf2bp1	A_64_P001475	79,899,155	●	-0.07	-0.37		-0.07	0.13	-0.19	0.18		0.13	**0.22**		0.04	0.07
Qpctl	A_42_P769597	80,056,574	●	-0.04	-0.33		**-0.20**	**-0.22**	-0.24	-0.09		0.11	-0.09		-0.07	0.00
Ppm1n (RGD1562091)	A_42_P773606	80,191,618	●	0.00	0.72		-0.03	0.12	**-0.35**	-0.02		-0.17	0.27		0.05	0.48
Trappc6a	A_64_P047432	80,417,310	●	-0.13	1.13	○	-0.20	**0.50**	-0.29	**0.60**		0.17	**0.34**	○	0.03	**0.60**
Gemin7	A_64_P010196	80,483,487	●	0.09	0.70	○	0.17	**1.68**	-0.02	**1.66**	○	**-0.38**	**1.78**	○	-0.09	**1.23**
Apoc4	A_64_P060497	80,599,572		N/A	0.10		N/A	N/A	**0.24**	N/A		0.05	**-0.30**	●	6.10	-0.34
Zfp61	A_44_P575055	81,144,024	●	0.05	0.62	○	-0.19	**0.54**	-0.20	**0.75**		0.10	**0.49**	○	0.05	**1.31**
Irgq	A_44_P897008	81,395,841	●	0.01	-0.38	○	-0.08	**-0.33**	0.06	**-0.35**	○	-0.14	**-0.50**		0.03	-0.12
Rps19	A_64_P003994	81,750,928	●	0.13	0.32		-0.02	-0.05	**-0.12**	**0.20**		0.05	-0.07		0.04	0.12
Megf8	A_44_P318448	82,185,034	●	0.17	0.46	○	-0.26	**0.67**	0.00	**0.76**		N/A	N/A	○	-0.10	**0.82**
Bckdha	A_43_P15665	82,452,281	●	0.04	-0.60		-0.22	**0.13**	**-0.26**	0.19		-0.18	0.04		**0.22**	0.02
Exosc5	A_44_P520943	82,452,469	●	-0.10	-0.49	○	-0.13	**-0.15**	**-0.23**	-0.16	○	0.08	**-0.42**	○	-0.17	**-0.29**
Coq8b (Adck4)	A_64_P034456	84,044,551	●	0.13	0.50		**-0.25**	-0.12	**-0.20**	0.07		-0.03	0.17		0.14	**0.17**
Blvrb	A_64_P086519	84,256,159	●	-0.07	0.27		-0.04	**0.18**	**-0.20**	-0.07		-0.05	**0.19**		-0.27	**-0.61**
Gmfg	A_64_P024297	85,317,968	●	-0.02	0.49		0.08	0.10	0.03	**0.35**		0.11	0.21		-0.02	**0.29**
Rps16	A_64_P068858	85,408,444	●	0.11	2.88	●	-0.07	2.50	-0.24	**2.68**	●	0.19	2.46	●	0.01	2.50
Eid2b	A_64_P133421	85,510,114	●	0.26	-0.78		**-0.28**	**-0.36**	-0.42	0.05		-0.12	**-0.49**		**-0.44**	-0.30
Nfkbib	A_44_P186860	86,948,845	●	0.07	0.49		-0.09	0.02	**-0.19**	0.23		0.02	**0.28**		**-0.42**	0.29
Fam98c (RGD1303117)	A_44_P997962	88,098,785	●	0.17	-0.53		**-0.26**	**-0.74**	**-0.25**	**-0.43**	○	-0.25	**-0.72**		-0.23	-0.18
Hcst	A_64_P126065	88,881,460	●	-0.04	-0.71		-0.21	**-0.75**	**-0.55**	**-0.54**	○	-0.21	**-0.74**	○	-0.11	**-0.96**

Fxyd5	A_64_P083600	89,474,252	●	0.18	0.53		**-0.18**	-0.07	**-0.27**	**0.36**		-0.17	-0.09		0.19	**-0.26**
Cebpg	A_64_P025098	91,296,656	●	0.09	0.39		-0.02	0.10	-0.12	-0.05		**-0.52**	**-0.36**		-0.05	0.15
Znf536	A_64_P006072	93,949,187	●	0.14	-0.69		0.09	-0.34	0.03	**-0.69**		N/A	N/A		**0.47**	**0.58**
Igf1r	A_44_P474037	128,924,966	●	0.25	0.59		0.02	0.10	0.21	**0.44**		N/A	N/A	○	0.35	**1.06**
Mfge8	A_42_P497225	140,860,882	●	0.14	0.43		**0.31**	**0.37**	0.17	**0.48**		0.15	-0.03	○	**0.45**	**1.80**
Prc1	A_42_P520891	142,087,208		**-0.48**	**0.40**	●	**-1.05**	**-0.37**	-0.87	-0.24		N/A	N/A		**-1.06**	**1.05**
Rab30	A_64_P131086	157,573,324	●	0.22	0.61		0.08	-0.04	-0.07	**0.57**		**-0.70**	**-1.52**		-0.20	0.20
Map6	A_44_P344181	164,225,934	●	0.18	0.76		0.23	0.33	-0.10	0.16		N/A	N/A		-0.04	**0.47**
Arntl	A_64_P117345	178,039,063	●	0.37	1.10		0.49	-0.17	-0.10	0.52		N/A	N/A		**-1.29**	-0.28
Vps35l (LOC361635)	A_64_P001922	188,448,478	●	0.11	0.30		-0.04	0.03	0.04	**0.38**		-0.07	-0.11		**0.30**	0.14
Il4ra	A_44_P337300	196,942,364	●	0.03	0.43		**-0.47**	-0.25	**-0.52**	-0.02		-0.31	-0.35		-0.21	0.18
Bcl7c	A_42_P495691	199,159,125	●	0.08	0.35		-0.16	-0.14	**-0.17**	0.01		0.16	0.15		-0.12	0.08
Ctbp2	A_64_P088990	205,030,567	●	-0.08	-0.32		-0.02	-0.09	0.05	0.04		-0.01	0.13		0.07	0.21
Clrn3	A_42_P495598	207,766,250		N/A	N/A	●	**-1.41**	-0.02	-1.38	**-0.28**		N/A	N/A		N/A	N/A
Mgmt	A_42_P837214	209,237,233	●	-0.27	0.42		0.04	0.70	-0.15	0.14	○	**-0.24**	**0.57**		**-0.23**	-0.12
Fuom (RGD1311186)	A_42_P696282	212,568,224		0.19	**-0.23**	●	**0.76**	0.03	0.79	**-0.12**		0.13	**-0.68**	●	0.00	-1.14
Cox8b	A_64_P036440	213,650,247		-0.06	-0.03		**0.72**	**0.38**	**-1.67**	**-2.06**		0.43	0.17	●	-0.09	-0.99
Cd151	A_44_P271720	214,446,659	●	0.05	0.35		**-0.26**	-0.14	**-0.32**	-0.09		**-0.39**	-0.02	○	0.00	**0.74**
Rhod	A_64_P066601	219,682,287	●	0.06	-0.39		**-0.26**	-0.04	**-0.31**	0.18		**-0.54**	-0.23		0.26	**0.61**
Gpr137	A_64_P087105	222,197,252	●	0.08	0.42		**-0.23**	-0.13	**-0.30**	-0.16		0.08	0.14		0.03	**0.34**
Plaat3 (Pla2g16)	A_64_P159519	222,844,144	●	**0.29**	-0.29	●	**-0.62**	**-0.25**	-0.58	**-0.22**		**-0.77**	**-0.48**		-0.01	-0.02
Asrgl1	A_64_P002924	225,329,770		**-0.24**	-0.01	●	**-1.34**	0.14	-0.95	-0.03	●	-1.30	0.17		**-0.71**	**0.54**
Cybasc3	A_44_P896040	226,634,009	●	-0.92	**0.18**	●	-1.20	-0.03	-1.06	0.06		**-0.74**	0.10	○	**-0.97**	0.12
Tmem132a	A_64_P062545	226,924,244	●	-0.45	0.63		-0.15	-0.03	-0.06	**0.39**		**0.44**	**0.47**	○	**0.31**	**0.59**
Ranbp6	A_42_P711568	247,985,497	●	-0.06	-0.22		0.04	0.11	-0.08	-0.02		**-0.26**	-0.18		0.07	**0.26**
Ch25h	A_44_P536613	252,808,380	●	0.54	1.16		-0.10	-0.21	0.20	**0.81**		-0.25	-0.10		**1.46**	0.04
Marchf5	A_44_P198112	255,842,783	●	0.10	0.34		-0.10	0.00	-0.19	0.18		0.15	**0.25**		**-0.50**	**-0.81**
Hoga1 (Dhdpsl)	A_64_P088575	261,291,870	●	0.31	1.90		**-0.24**	-0.11	**-0.21**	-0.20		-0.25	-0.14		-0.03	**-0.40**
Nolc1	A_43_P12332	265,829,108	●	-0.15	-0.30		-0.05	0.05	-0.01	0.02		**-0.36**	0.02		0.01	**0.18**
Nfkb2	A_42_P658264	266,053,002	●	0.12	0.43		0.01	-0.08	-0.05	0.13		0.31	0.21		0.00	**0.37**
Cuedc2	A_44_P1002173	266,086,299	●	-0.06	0.48		0.11	**0.18**	0.05	**0.39**		**0.36**	**0.42**		0.19	**0.33**
Rbm20	A_42_P473812	274,391,932	●	0.00	0.49		N/A	N/A	N/A	N/A		N/A	N/A		N/A	N/A

Fold changes (hypertensive strain vs WKY; log_2-transformed values) are shown in each cell. Significant values are underlined ($P<1×10^{-8}$) or in bold letter ($1×10^{-8}≤P<1×10^{-4}$).

Significant genes are initially selected for “non-shared” differential expression (D/E) when they show $P < 1\times10^{-8}$ in one type of inter-strain comparison but not in the other ($P \geq 0.05$ with a concordant direction or all P-values in the opposite direction). Then, to validate D/E of the corresponding genes in tissues other than the primary one, we set suggestive and significant evidence for non-shared D/E. We consider it as suggestive (open circle in the column D/E) when D/E show $P < 10^{-4}$, in the direction concordant with the primary tissue; we claim it to be significant (closed circle in the column D/E) when criteria for the primary tissue is satisfied in another tissue.

In some genes, the direction of differential expression is opposite to that in the primary tissue. Some genes are not included in the list when there is no appropriate transcript for the microarray probes annotated.

Table S5. A list of genes that are likely to be under cis-acting influences

Gene	Agilent probe	Chr	Pos. (Rnor_6.0)	Heart		Aorta		Whole kidney		Renal cortex		Liver	
				SHR	SHRSP	SHR	SHRSP	SHR	SHRSP	SHR	SHRSP	SHR	SHRSP
Map3k5	A_64_P129293	1	15,412,603	1E-09	4E-21	1E-09	2E-08	4E-08	2E-07	5E-05	3E-05	9E-04	0.001
Sema4b	A_64_P133177	1	141,986,145	4E-06	8E-20	4E-07	2E-04	9E-10	1E-08	2E-08	6E-08	6E-05	2E-06
Homer2	A_64_P016059	1	143,535,583	2E-17	3E-17	4E-10	2E-10	3E-08	3E-04	1E-09	2E-09	0.202	0.456
Acsm5	A_64_P151353	1	189,241,593	N/A	N/A	4E-08	1E-06	3E-16	9E-15	1E-14	2E-15	9E-12	7E-13
Pagr1	A_44_P1029472	1	198,450,688	2E-15	1E-14	2E-11	5E-10	6E-06	0.103	9E-07	4E-05	0.582	0.62
Rnf40	A_64_P045578	1	199,037,544	3E-13	3E-19	8E-12	4E-13	2E-07	3E-05	2E-05	6E-06	3E-07	1E-07
Gatad2b	A_42_P516658	2	189,655,702	2E-08	2E-06	1E-06	2E-08	7E-06	0.001	6E-08	5E-08	0.040	0.014
Tars2	A_64_P041307	2	197,878,142	3E-08	3E-20	6E-11	5E-12	6E-12	6E-10	1E-07	2E-07	1E-07	4E-07
Endog	A_44_P213133	3	8,741,766	1E-25	2E-35	7E-13	8E-15	5E-17	3E-14	6E-13	1E-13	1E-11	7E-12
Tor1b	A_43_P18366	3	9,792,899	2E-19	8E-31	7E-09	6E-12	3E-13	9E-12	2E-11	8E-12	4E-08	2E-07
Dusp19	A_44_P992697	3	67,849,966	2E-17	7E-24	3E-11	2E-12	3E-10	5E-06	5E-11	4E-10	6E-07	4E-08
Ptprj	A_43_P15275	3	79,390,956	8E-20	6E-24	3E-08	1E-07	1E-17	2E-13	3E-13	6E-14	3E-11	7E-11
RGD1309540	A_44_P107766	3	80,349,145	6E-12	4E-14	2E-04	1E-04	3E-10	5E-07	1E-09	6E-09	3E-07	4E-07
Accs	A_64_P115142	3	82,756,953	2E-08	3E-18	4E-07	2E-10	3E-09	7E-07	2E-05	3E-06	2E-04	0.002
Chchd5	A_64_P098527	3	121,660,110	5E-06	2E-08	2E-11	5E-13	9E-10	2E-04	4E-08	4E-07	0.397	0.113
Ift52	A_43_P18817	3	159,392,193	8E-09	3E-18	5E-08	3E-04	5E-09	6E-06	1E-07	2E-07	6E-05	1E-05
Pmepa1	A_64_P002904	3	171,342,646	6E-07	9E-17	0.392	0.001	2E-05	0.021	5E-05	3E-05	2E-05	7E-05
Polr3k	A_44_P307964	3	177,374,812	7E-10	6E-17	2E-08	7E-07	9E-09	5E-04	3E-08	7E-08	2E-06	8E-06
Tmem243	A_64_P016751	4	21,920,651	5E-11	3E-23	6E-08	7E-10	8E-11	2E-08	5E-09	2E-09	0.003	0.006
Mrps33	A_44_P489995	4	67,610,710	8E-11	8E-11	7E-09	3E-11	4E-06	0.001	6E-09	5E-09	0.001	9E-04
Inmt	A_43_P23115	4	85,386,231	N/A	N/A	4E-10	4E-09	1E-10	3E-11	3E-10	2E-12	3E-12	4E-11
Retsat	A_44_P402507	4	100,465,170	2E-23	3E-36	5E-09	9E-09	1E-12	4E-09	4E-08	4E-10	0.294	0.956
Ptgr1	A_42_P698240	5	76,129,441	3E-06	8E-19	3E-10	2E-11	2E-17	7E-15	4E-11	1E-11	8E-06	1E-05

Hdhd3	A_44_P175822	5	78,361,647	8E-04	0.103	7E-08	2E-06	5E-08	0.002	6E-06	2E-05	3E-06	5E-05
Hdhd3	A_64_P090543	5	78,361,647	0.012	0.024	3E-08	6E-09	4E-10	2E-06	2E-04	5E-04	5E-07	9E-06
Atp6v1g1	A_44_P1040796	5	79,367,663	6E-13	1E-22	3E-14	3E-15	1E-11	2E-10	1E-10	7E-12	5E-10	4E-10
Hpdl	A_44_P117119	5	135,677,432	8E-08	3E-09	0.114	0.479	3E-13	1E-15	1E-07	2E-11	NA	NA
Cldn19	A_64_P138415	5	138,300,107	3E-16	2E-23	3E-07	2E-09	2E-05	0.539	7E-05	1E-05	NA	NA
Pigv	A_44_P175875	5	151,895,016	4E-11	6E-15	0.001	5E-07	5E-09	4E-09	5E-07	9E-08	6E-06	8E-06
Slc30a3	A_64_P017363	6	26,642,783	1E-06	2E-14	2E-07	4E-08	0.954	0.453	0.098	0.839	2E-12	3E-10
Rab10	A_44_P413979	6	27,721,120	3E-10	6E-22	2E-05	4E-09	2E-10	2E-05	0.002	0.014	7E-06	2E-07
Cyp4f17	A_64_P099017	7	14,529,483	4E-14	2E-20	5E-09	1E-09	1E-17	2E-11	1E-07	2E-07	1E-07	3E-07
Zfp799	A_64_P255450	7	15,072,703	5E-11	1E-20	0.809	0.994	6E-07	0.005	4E-08	3E-06	4E-05	9E-05
Polr2f	A_43_P12557	7	120,380,544	8E-15	8E-26	4E-04	1E-06	6E-05	1E-05	1E-08	5E-09	2E-06	7E-06
Med17	A_44_P119527	8	13,526,025	1E-11	1E-24	1E-07	7E-08	1E-10	5E-09	5E-11	5E-11	5E-07	5E-07
Cep295	A_44_P100886	8	13,909,188	3E-05	2E-11	1E-07	7E-06	5E-08	2E-05	2E-07	1E-07	0.371	0.334
Mrpl4	A_44_P518434	8	22,021,213	3E-10	2E-19	1E-07	4E-08	5E-11	9E-09	2E-08	9E-08	2E-06	2E-06
Acaa1a	A_43_P11432	8	128,027,958	2E-08	7E-21	0.577	0.532	2E-07	2E-05	9E-04	3E-04	2E-06	3E-06
Itm2c	A_43_P10901	9	92,916,469	1E-19	8E-29	3E-08	9E-11	1E-12	4E-09	5E-09	2E-09	3E-09	8E-08
Epb41l3	A_44_P123492	9	117,538,009	2E-08	1E-23	3E-05	3E-05	6E-07	2E-05	5E-06	3E-07	2E-08	7E-08
Timd4	A_64_P027288	10	31,880,918	2E-06	9E-11	7E-04	0.001	1E-05	4E-05	N/A	N/A	6E-09	9E-10
RGD1559482	A_64_P157691	10	104,952,237	1E-07	7E-19	2E-08	4E-08	2E-06	0.497	0.232	N/A	1E-08	1E-09
Arl16	A_42_P539095	10	109,639,054	1E-07	2E-06	4E-10	1E-10	4E-10	8E-07	2E-08	6E-09	2E-05	2E-06
Dcxr	A_43_P13256	10	109,909,646	0.025	2E-08	3E-07	4E-07	3E-05	5E-06	0.003	2E-05	2E-05	5E-06
Wdr45l	A_44_P159569	10	110,555,629	1E-09	4E-20	0.006	8E-04	0.005	3E-05	8E-06	2E-06	2E-08	7E-08
Pigp	A_42_P554722	11	34,598,275	2E-11	3E-19	2E-05	1E-06	4E-08	4E-05	2E-05	4E-04	0.004	4E-05
Phldb2	A_42_P732251	11	57,404,196	3E-11	3E-23	3E-05	1E-05	4E-12	4E-10	4E-10	1E-10	6E-06	4E-04
Asmtl	A_42_P746109	12	18,531,990	9E-11	1E-16	2E-09	3E-08	1E-09	5E-06	4E-07	5E-07	0.004	0.009
Mcm7	A_64_P081501	12	19,314,016	5E-14	3E-30	9E-07	1E-08	9E-12	9E-13	7E-11	2E-11	3E-05	9E-08

Nxpe5	A_42_P540609	12	19,440,501	2E-10	3E-20	3E-08	2E-08	0.029	0.014	0.002	0.001	7E-05	7E-05
Ccdc92	A_44_P1030714	12	37,211,316	2E-19	1E-30	2E-08	3E-11	8E-09	2E-10	3E-10	1E-11	0.004	0.002
Snrnp35	A_64_P135202	12	37,538,403	5E-09	3E-21	2E-08	2E-08	2E-09	4E-05	3E-06	1E-05	0.001	2E-04
Rhof	A_44_P838992	12	38,880,377	0.003	6E-13	1E-09	2E-07	6E-07	2E-05	1E-05	7E-07	1E-07	1E-08
RGD1562310	A_44_P686406	12	43,940,798	8E-05	7E-05	3E-06	2E-06	0.039	4E-04	5E-04	1E-04	2E-07	4E-05
RGD1564614	A_44_P475444	13	56,958,549	1E-16	1E-23	6E-05	1E-05	6E-05	2E-05	5E-04	0.013	0.049	0.001
Cfh	A_42_P819656	13	57,080,549	5E-21	3E-27	2E-05	3E-06	4E-08	3E-05	2E-05	6E-05	0.575	0.013
Fmo3	A_44_P358194	13	80,862,963	2E-12	5E-22	4E-08	3E-09	5E-05	8E-04	0.498	0.003	1E-06	4E-07
Dpt	A_44_P180259	13	83,073,550	2E-10	3E-29	5E-04	4E-09	2E-14	2E-13	2E-10	3E-10	4E-05	5E-06
Tmco1	A_44_P1018997	13	85,465,792	2E-05	5E-13	1E-09	4E-06	6E-07	5E-05	1E-05	5E-07	0.033	2E-04
Pcp4l1	A_64_P005029	13	89,565,813	1E-09	3E-16	2E-05	1E-04	2E-05	0.182	5E-08	3E-07	2E-09	5E-08
Tatdn3	A_42_P803590	13	109,663,364	2E-19	4E-29	1E-10	7E-11	3E-10	1E-07	1E-10	3E-10	4E-09	3E-09
Ints7	A_42_P661065	13	110,257,571	5E-11	2E-19	7E-07	4E-08	4E-05	4E-06	8E-08	5E-09	0.004	5E-04
Ints7	A_64_P052464	13	110,257,571	3E-11	6E-16	4E-05	9E-07	2E-06	5E-06	5E-10	9E-11	NA	NA
Hsd17b11	A_44_P1043814	14	7,073,445	1E-05	4E-10	8E-06	1E-06	5E-08	5E-04	1E-06	1E-06	0.392	0.378
Gnpda2	A_44_P992306	14	62,646,110	2E-07	2E-05	0.002	1E-06	9E-04	0.544	2E-08	9E-09	7E-05	2E-05
Nt5dc2	A_64_P005043	16	7,212,488	2E-11	3E-19	6E-09	4E-08	0.045	5E-04	7E-05	3E-07	1E-08	6E-08
Ncoa4	A_64_P066923	16	8,302,950	3E-08	1E-14	5E-05	3E-07	4E-07	2E-07	8E-07	2E-08	2E-05	3E-04
Fam32a	A_42_P467381	16	19,308,842	2E-19	4E-26	4E-06	2E-09	2E-07	6E-07	5E-07	2E-06	5E-04	2E-04
Borcs8	A_64_P100904	16	21,029,134	5E-09	8E-18	4E-06	5E-09	2E-06	2E-04	4E-06	3E-05	1E-04	1E-05
Borcs8	A_64_P004531	16	21,029,134	9E-15	1E-22	2E-11	3E-13	2E-10	3E-06	5E-09	5E-08	1E-05	1E-06
Hpf1	A_44_P1036068	16	32,540,217	9E-08	3E-15	3E-07	4E-07	7E-07	0.007	7E-07	5E-07	0.011	0.102
Spcs3	A_64_P007462	16	40,050,734	1E-06	3E-20	0.012	0.011	8E-04	1E-04	4E-07	2E-08	2E-07	7E-07
Wwc2	A_64_P041016	16	47,368,768	2E-12	2E-20	2E-05	8E-10	1E-09	9E-08	7E-09	1E-09	1E-07	3E-06
Tti2	A_44_P1042163	16	64,745,207	4E-12	2E-26	0.005	1E-06	3E-07	4E-09	3E-09	9E-11	1E-06	2E-05
Isca1	A_64_P022751	17	5,281,727	1E-12	8E-19	3E-05	2E-05	5E-08	4E-04	4E-06	7E-07	9E-04	0.003

Riok1	A_64_P059262	17	27,451,832	1E-19	2E-29	5E-07	5E-10	3E-06	7E-07	2E-08	5E-09	5E-05	1E-05
Aldh5a1	A_64_P030919	17	42,133,076	1E-11	2E-20	7E-06	5E-08	5E-07	4E-04	5E-06	3E-06	2E-05	5E-05
Aldh5a1	A_64_P067678	17	42,133,076	5E-09	5E-15	2E-05	1E-07	3E-06	3E-04	7E-05	4E-05	4E-05	1E-04
Hist1h2bc	A_64_P130572	17	44,738,330	2E-05	3E-16	5E-10	8E-09	0.108	0.002	0.842	0.695	6E-06	7E-05
Impad1	A_64_P104454	17	90,218,013	1E-11	1E-14	2E-05	4E-06	2E-05	0.075	4E-06	5E-05	8E-06	1E-04
Chmp1b	A_64_P011719	18	62,923,620	6E-14	4E-23	1E-07	5E-07	6E-10	7E-07	1E-09	5E-08	2E-07	3E-06
Stard6	A_44_P419615	18	68,983,545	3E-09	2E-14	3E-08	1E-07	1E-10	5E-05	2E-07	5E-08	NA	NA
Pqlc1	A_64_P021320	18	76,770,012	6E-14	9E-24	0.083	0.006	1E-11	5E-10	9E-11	7E-11	7E-05	4E-05
Ces1d	A_64_P148413	19	15,033,108	3E-05	2E-19	2E-07	2E-09	5E-06	0.735	9E-04	7E-06	8E-09	8E-09
Mdc1	A_42_P627572	20	3,405,285	5E-10	4E-16	9E-09	9E-08	2E-11	4E-08	3E-12	7E-13	2E-08	9E-07
Pwp2	A_64_P146698	20	11,228,844	9E-08	5E-16	2E-07	8E-09	2E-06	4E-06	8E-09	2E-09	0.038	0.007
Gstt3	A_44_P1015203	20	13,817,795	2E-10	5E-14	1E-11	3E-13	5E-11	3E-06	2E-05	1E-07	4E-10	1E-11
	A_64_P326359	MT		2E-18	3E-24	5E-08	3E-11	1E-11	5E-08	1E-10	4E-12	1E-07	5E-07
	A_64_P042093	MT		1E-08	9E-22	3E-05	1E-07	1E-07	1E-09	0.324	0.013	0.002	4E-06
	A_64_P042811	MT		3E-08	2E-22	1E-08	2E-09	1E-17	3E-16	4E-12	2E-12	3E-10	2E-10
	A_64_P118308	MT		1E-06	8E-12	1E-09	6E-05	4E-12	5E-09	1E-10	3E-11	8E-08	7E-08
	A_64_P143154	MT		2E-07	1E-15	7E-05	7E-09	0.003	5E-04	2E-05	2E-07	7E-04	0.033
AABR07041140.1	A_64_P048852	X	120,313,696	1E-19	2E-28	2E-10	2E-10	1E-13	5E-09	7E-12	1E-12	5E-08	2E-07

Genes that demonstrate shared differential expression in ≥3 tissues are listed in the table.

For each cell, *P*-values are shown for the analysis of differential expression in each inter-strain comparison.

Fold change (FC; hypertensive strain vs WKY) is largely grouped into 3 classes: |FC|≥4 (dark blue or red), 4>|FC|≥2 (medium blue or red), and 2>|FC| (light blue or red).

	↓	↑
|FC| ≥4		
4> |FC| ≥2		
2> |FC|		

Table S6. A list of SSLP markers originally developed

Marker	Gene Symbol	Chr	Position (Rnor_6.0)		PCR product size (bp)			Forward primer (5'-to-3')	Reverse primer (5'-to-3')
			start	end	SHRSP	SHR	WKY		
D1Tkyo24	Igf1r	1	129,086,255	129,086,495	241	243	227	CTCTTGACTGTGGGTTGCTA	CGGAACCAGAAATCTGATCT
D1Tkyo26	Ntrk3_RFLP	1	139,943,456	139,943,630	135+40	135+40	96+79	AGAAGGTTGGCTCCAACTAG	CAGATGCGATGATCCTTGTG
D1Tkyo27	Cib1	1	142,016,448	142,016,558	84	84	109	ACAGAAGGCATTCTTATGTAGGCC	CAGGAGGTCCAGGAAGTCCT
D1Tkyo32	Ctsc	1	151,919,661	151,919,851	175	180	171	GGAGAGACAGGGTTGGCATT	GCAGCTTCCCAATAAACCTGA
D1Tkyo44	Ctsd	1	215,558,920	215,559,126	192	210	192	CCAGCCTCCCCTATTTCC	TGGTGCTCTGTGGGGTCAAG
D1Tkyo49	Insl6	1	247,468,493	247,468,684	183	176	183	CTGGAGGGGTTGCGTTACCACT	TAGGGCAGGAGACTTTGTGCTCAG
D1Tkyo56	Scd (Scd1)	1	264,157,471	264,157,740	268	266	266	ACTGGTGCAGCTGGAAGGTCTCAT	GCAGACTTGTACCGCAGTT
D1Tkyo58	Add3	1	273,957,142	273,957,325	171	171	163	CATCGTCTTCAAACCGCATGG	CACAAACAAATGAGCGGGTAA
D2Tkyo1	Hmgcr	2	27,480,425	27,480,570	151	151	148	GCGGACGTCTGTGTAGAAGA	GAGGCGCAACTGGAAACT
D2Tkyo2	Hmgcs1	2	52,424,108	52,424,275	172	172	157	TATAGCCAATTTTGAGTGACTGAG	GGCAAGCATTAATTACGTGAGAC
D2Tkyo6	Selenof (Sep15)	2	250,631,666	250,631,848	194	194	188	CCTCCAGGACAGCCAAGACTACC	TTCACCTCAGTCGCCAGA
D2Tkyo7	Bcl10	2	251,827,434	251,827,641	178	178	206	AACTCCAGGGAGGTCAGAACTA	TGGAGTTTCCTGTTGGTAGTAAGAA
D2Tkyo10	Uox	2	252,491,774	252,492,002	215	211	207	TCATACCCAGCACCGAACTC	ACATATCAATACATACACACGGAGA
D2Tkyo12	Adgrl4 (Etl)	2	256,616,152	256,616,295	158	158	154	ACAAATGCTGCTGACCACTTA	ATAAAAGAGAGGGAAGTGAATAT
D2Tkyo13	St6galnac3 (Siat7c)	2	259,430,257	259,430,397	159	159	146	GGGACCTAGGCACAATCGAGTG	CGCTTCCATAGCTTGCTCAGTT
D3Tkyo4	Kynu	3	28,448,867	28,449,010	158	152	144	TATGCTCTTTTTTCTCACAGAAAC	TCCACCCTCATAATATGTTCTC
D3Tkyo9	Bfsp1	3	137,952,204	137,952,438	225	225	227	AAGACTATAGAGAGGCCATTAG	GTCAAGGGCTTCATCTAGG
D3Tkyo11	Tgm2	3	154,604,389	154,604,514	131	135	127	TGATAATGGTGCATTGTACCTGAA	GGCCAAATGTTCTTTCTTAGTAT
D4Tkyo1	Tpra1 (Tpra40)	4	120,858,124	120,858,241	119	119	123	TAAGTACAGGGAGGGCTGCGTTGT	CCGCAGCAGCCTTCAAAGGTGTG
D4Tkyo2	Frmd4b (Grsp1)	4	129,769,006	129,769,143	135	135	131	GTGCTTACATCTGCGAACA	GGCATATGGTTACAATGAGTTATC
D4Tkyo8	Slc6a13	4	153,903,676	153,903,876	200	208	200	TGTAGCCCACTATATATCCAATA	GCCAACCTGGACTACATGAGTT
D8Tkyo1	Cnn1_Ecsit	8	23,095,882	23,096,123	212	212	216	TGCTGCTAGTCTGACAACC	ACTGTGTCTGGCTCAACTAGAG
D8Tkyo5	Gldn (Crgl2)	8	58,902,089	58,902,286	191	191	187	ATCTGTCTCAAATTTTCTCAGTA	TTGATTTTTAGAGCAGATATT
D8Tkyo9	Plod2	8	100,011,322	100,011,577	239	239	243	ACACTGTGGCACGGACCTA	GACCCAGTCAGCTGTCAGTACC
D8Tkyo10	Acaa1a (Acaa1)	8	128,032,912	128,033,050	131	131	137	GGTATGAGATCAATGCTGCC	GGTGCATTTGGACATTTGTAG
D9Tkyo4	Kcnq5	9	27,587,386	27,587,578	208	208	216	AGAAAGATATTCCCCAGTTATCCTC	AACGTTCTTATGTACTAAGCCGTCT
D9Tkyo5	Rims1 (Rim1)	9	28,466,437	28,466,705	273	279	276	GAAACCTTGCCTCGAAA	CAGATGCCTCCTTCTCATACTG
D9Tkyo11	LOC102554599	9	79,270,504	79,270,660	158	158	174	CCTCCAAGAACATCAAAATGA	CGGGCTCATAGGAGTACACAG
D13Tkyo1	Bcl2	13	26,605,475	26,605,732	250	250	254	TCTCTGAAGACGCTGCTC	TTTGACCATTTGCCTGAA
D15Tkyo3	Fhit	15	15,826,116	15,826,369	256	270	256	GGGCAGAAGGAGAGACTAACTA	GATGTCCACAAAACTTAACCCTGAC
D15Tkyo6	Rabggta	15	34,385,010	34,385,188	178	178	176	CCATTTAATTCTCCCAACTACAC	GATATTGGCAAGGAAGTACC
D15Tkyo8	Clu	15	42,655,149	42,655,254	87	107	107	TCTGGGCAGGGTTAGAGATG	GAAGCTGGATTGGTTGCAT
D15Tkyo18	Ednrb	15	88,011,655	88,011,915	254	254	256	GCTTCTGACTCCCAAATGAT	GATGCCCCTACTCTGAACC

Table S7. A list of SSLP markers used for a speed-congenic approach to the development of consomic/congenic lines

Marker	Chr	Position (v6.0)	SHRSP-derived consomic/congenic lines							
			RNO1	RNO3	RNO4	RNO7	RNO8	RNO9	RNO13	RNO15
D1Mgh2	1	23,406,428	W	P	P	P	P	P	P	P
D1Rat13	1	40,881,280	W	P	P	P	P	P	P	P
D1Rat125	1	51,412,813	W	P	P	P	P	P	P	P
D1Rat196	1	58,354,072	W							
D1Mgh5	1	79,689,548	W	P	P	P	P	P	P	P
D1Wox17	1	83,103,580	W							
D1Rat27	1	94,201,400	W							
D1Wox18	1	100,133,276	W	P	P	P	P	P	P	P
D1Wox29	1	130,779,148	W							
D1Rat269	1	132,448,117	W	P	P	P	P	P	P	P
D1Rat43	1	144,634,295	W	P	P	P	P	P	P	P
D1Rat44	1	161,493,758	W	P	P	P	P	P	P	P
D1Rat193	1	193,968,438	W							
D1Wox19	1	197,963,658	W			P				
D1Arb21	1	199,254,518	W	P	P	P	P	P	P	P
D1Rat112	1	219,238,476	W	P	P	P	P	P	P	P
D1Rat295	1	227,107,736	W							
D1Rat71	1	230,420,627	W	P	P	P	P	P	P	P
D1Wox10	1	236,763,415	W	P	P	P	P	P	P	P
D1Tkyo49	1	247,468,493	W	P	P	P	P	P	P	P
D1Tkyo56	1	264,157,471	W	P						
D1Mgh13	1	266,793,821	W	P	P	P	P	P	P	P
D1Rat376	1	270,122,137	W							
D1Rat122	1	278,048,619	W	P	P	P	P	P	P	P
D1Rat156	1	278,806,691	W	P	P	P	P	P	P	P
D1Mit14	1	280,632,517	W	P	P	P	P	P	P	P
D1Rat90	1	281,795,624	W	P	P	P	P	P	P	P
D2Rat3	2	8,579,280	P	P	P	P	P	P	P	P
D2Tkyo1	2	27,480,425	P	P	P	P	P	P	P	P
D2Mgh2	2	40,171,834	P	P	P	P	P	P	P	P
D2Mit5	2	66,828,049	P	P	P	P	P	P	P	P
D2Wox6	2	115,783,552	P	P	P	P	P	P	P	P
D2Mgh24	2	140,565,889	P	P	P	P	P	P	P	P

Marker	Chr	Position (v6.0)	SHR-derived consomic/congenic lines					
			RNO1	RNO2	RNO3	RNO4	RNO15	RNO19
D1Mgh2	1	23,406,428	W	S	S	S	S	S
D1Rat125	1	51,412,813	W	S	S	S	S	S
D1Rat316	1	72,887,924	W	S	S	S	S	S
D1Rat27	1	94,201,400	W		S			
D1Wox18	1	100,133,276	W	S	S	S	S	S
D1Tkyo24	1	129,086,255	W	S	S	S	S	S
D1Rat200	1	133,076,978	W	S	S	S	S	S
D1Rat36	1	135,022,396	W					
D1Rat40	1	135,428,373	W	S	S	S	S	S
D1Rat43	1	144,634,295	W	S	S	S	S	S
D1Rat106	1	145,140,412	W					
D1Tkyo32	1	151,919,661	W	S	S	S	S	S
D1Rat44	1	161,493,758	W					
D1Rat236	1	162,891,429	W					
D1Wox33	1	168,126,089	W	S	S	S	S	S
D1Rat132	1	172,789,146	W	S	S	S	S	S
D1Rat164	1	177,235,071	W					
D1Wox19	1	197,963,658	W	S	S	S	S	S
D1Tkyo44	1	215,558,920	W	S	S	S	S	S
D1Rat112	1	219,238,476	W	S	S	S	S	S
D1Wox10	1	236,763,415	W	S	S	S	S	S
D1Tkyo49	1	247,468,493	W	S	S	S	S	S
D1Mgh13	1	266,793,821	W	S	S	S	S	S
D1Rat376	1	270,122,137	W					
D1Rat122	1	278,048,619	W	S	S	S	S	S
D1Rat156	1	278,806,691	W					
D1Mit14	1	280,632,517	W	S	S	S	S	S
D1Rat90	1	281,795,624	W	S	S	S	S	S
D2Rat3	2	8,579,280	S	W	S	S	S	S
D2Rat189	2	14,564,017		W				
D2Rat191	2	20,321,436		W				
D2Rat124	2	26,186,097		W				
D2Tkyo1	2	27,480,425	S	W	S	S	S	S
D2Mgh2	2	40,171,834	S	W	S	S	S	S

D2Rat97	2	174,210,477	P	P	P	P	P	P	P	P
D2Wox24	2	189,854,122	P	P	P	P	P	P	P	P
D2Mgh12	2	217,498,545	P	P	P	P	P	P	P	P
D2Tkyo6	2	250,631,666	P	P	P	P	P	P	P	P
D2Tkyo7	2	251,827,434	P	P	P	P	P	P	P	P
D2Mgh13	2	252,466,425	P	P	P	P	P	P	P	P
D2Tkyo10	2	252,491,774	P	P	P	P	P	P	P	P
D2Mit16	2	260,543,076	P	P	P	P	P	P	P	P
D3Mgh16	3	6,000,748	P	W	P	P	P	P	P	P
D3Mgh19	3	15,203,122	P	W	P	P	P	P	P	P
D3Mit9	3	34,394,121	P	W	P	P	P	P	P	P
D3Wox3	3	30,846,101	P	W	P	P	P	P	P	P
D3Rat80	3	36,599,933		W						
D3Rat186	3	45,228,240		W						
D3Rat227	3	45,406,058		W						
D3Rat110	3	47,155,167	P	W	P	P	P	P	P	P
D3Mgh6	3	54,630,948	P	W	P	P	P	P	P	P
D3Rat108	3	63,849,481		W						
D3Rat74	3	66,480,395		W						
D3Rat93	3	79,874,819		W						
D3Rat127	3	92,851,616		W						
D3Wox16	3	94,028,502	P	W	P	P	P	P	P	P

D2Tkyo2	2	52,424,108		W				
D2Mit5	2	66,828,049	S	W	S	S	S	S
D2Rat21	2	75,687,495	S	W	S	S	S	S
D2Rat122	2	98,545,920	S	W	S	S	S	S
D2Rat160	2	113,358,300		W				
D2Wox6	2	115,783,552	S	W	S	S	S	S
D2Rat171	2	116,075,644	S	W	S	S	S	S
D2Mgh24	2	140,565,889	S	W	S	S	S	S
D2Rat283	2	174,171,668	S	W	S	S	S	S
D2Rat97	2	174,210,477		W			S	
D2Wox24	2	189,854,122	S	W	S	S	S	S
D2Rat231	2	196,644,924		W				
D2Rat239	2	213,923,459	S	W	S	S	S	S
D2Mgh12	2	217,498,545		W				
D2Rat241	2	224,954,385	S	W	S	S	S	S
D2Rat59	2	228,582,621		W				
D2Rat88	2	228,984,526		W				
D2Tkyo6	2	250,631,666		W				
D2Tkyo7	2	251,827,434	S	W	S	S	S	S
D2Tkyo10	2	252,491,774		W				
D2Tkyo12	2	256,616,152		W				
D2Rat106	2	257,446,379	S	W	S	S	S	S
D2Tkyo13	2	259,430,257		W				
D2Mit16	2	260,543,076	S	W	S	S	S	S
D3Mgh16	3	6,000,748	S	S	W	S	S	S
D3Mgh19	3	15,203,122	S	S	W	S	S	S
D3Tkyo4	3	28,448,867		S	W			
D3Mit9	3	34,394,121			W			
D3Wox3	3	30,846,101	S	S	W	S	S	S
D3Rat80	3	36,599,933	S	S	W	S	S	S
D3Rat110	3	47,155,167			W			
D3Mgh6	3	54,630,948	S	S	W	S	S	S
D3Rat108	3	63,849,481			W			
D3Rat74	3	66,480,395		S	W			
D3Rat93	3	79,874,819	S	S	W	S	S	S
D3Rat127	3	92,851,616	S	S	W	S	S	S
D3Wox16	3	94,028,502			W			
D3Rat166	3	106,900,427			W			

Disease Models & Mechanisms • Supplementary information

D3Rat166	3	106,900,427		W						
D3Mit14	3	120,917,788	P	W	P	P	P	P	P	P
D3Mit13	3	120,917,851	P	W						
D3Mgh15	3	123,599,592		W						
D3Rat157	3	128,716,357		W						
D3Kyo3	3	129,787,213	P	W	P	P	P	P	P	P
D3Tkyo9	3	137,952,204	P	W	P	P	P	P	P	P
D3Rat148	3	146,426,801		W						
D3Mit2	3	148,348,517	P	W	P	P	P	P	P	P
D3Rat61	3	153,381,237		W						
D3Rat5	3	154,416,635		W						
D3Wox7	3	160,632,397	P	W	P	P	P	P	P	P
D3Rat1	3	176,417,943		W						
D4Wox27	4	3,044,017			W					
D4Rat4	4	4,364,715	P	P	W	P	P	P	P	P
D4Rat10	4	26,753,655	P	P	W	P	P	P	P	P
D4Rat11	4	34,379,764	P	P	W	P	P	P	P	P
D4Rat101	4	47,638,322			W					
D4Rat15	4	46,898,276			W					
D4Mit9	4	55,324,857	P	P	W	P	P	P	P	P
D4Wox24	4	70,773,499	P	P	W	P	P	P	P	P
D4Rat34	4	86,438,317	P	P	W	P	P	P	P	P
D4Rat41	4	97,758,884	P	P	W	P	P	P	P	P
D4Mgh17	4	113,100,978	P	P	W	P	P	P	P	P
D4Tkyo1	4	120,858,124			W					
D4Tkyo2	4	129,769,006			W					
D4Mgh7	4	136,351,734	P	P	W	P	P	P	P	P
D4Mgh8	4	145,254,666	P	P	W	P	P	P	P	P
D4Mgh11	4	168,046,938	P	P	W	P	P	P	P	P
D5Rat116	5	1,525,098	P	P	P	P	P	P	P	P
D5Mgh1	5	4,644,684	P	P	P	P	P	P	P	P
D5Mgh17	5	14,408,903	P	P	P	P	P	P	P	P
D5Rat190	5	24,382,492	P	P	P	P	P	P	P	P
D5Rat4	5	48,722,038	P	P	P	P	P	P	P	P
D5Mit10	5	56,902,367	P	P	P	P	P	P	P	P

F

D3Mit14	3	120,917,788	S	S	W	S	S	S
D3Rat157	3	128,716,357	S	S	W	S	S	S
D3Kyo3	3	129,787,213			W			
D3Rat63	3	146,076,361			W			
D3Rat148	3	146,426,801			W			
D3Mit2	3	148,348,517	S	S	W	S	S	S
D3Rat61	3	153,381,237			W			
D3Rat6	3	153,412,455			W			
D3Rat5	3	154,416,635	S	S	W	S	S	S
D3Tkyo11	3	154,604,389		S	W			
D3Wox7	3	160,632,397	S	S	W	S	S	S
D3Rat1	3	176,417,943	S	S	W	S	S	S
D4Wox27	4	3,044,017	S	S	S	W	S	S
D4Rat4	4	4,364,715	S	S	S	W	S	S
D4Rat10	4	26,753,655	S	S	S	W	S	S
D4Rat11	4	34,379,764	S	S	S	W	S	S
D4Rat101	4	47,638,322				W		
D4Rat15	4	46,898,276	S	S	S	W	S	S
D4Rat122	4	64,915,651				W		
D4Wox24	4	70,773,499	S	S	S	W	S	S
D4Rat163	4	74,293,997				W		
D4Rat34	4	86,438,317	S	S	S	W	S	S
D4Rat41	4	97,758,884	S	S	S	W	S	S
D4Rat176	4	112,807,675				W		
D4Mgh17	4	113,100,978	S	S	S	W	S	S
D4Tkyo1	4	120,858,124				W		
D4Tkyo2	4	129,769,006				W		
D4Mgh7	4	136,351,734	S	S	S	W	S	S
D4Tkyo8	4	153,903,676	S	S	S	W	S	S
D4Mgh11	4	168,046,938	S	S	S	W	S	S
D5Rat116	5	1,525,098	S	S	S	S	S	S
D5Rat126	5	24,197,001	S	S	S	S	S	S
D5Rat4	5	48,722,038	S	S	S	S	S	S
D5Mit10	5	56,902,367	S	S	S	S	S	S
D5Mit1	5	57,148,431	S	S	S	S	S	S
D5Rat8	5	59,908,789	S	S	S	S	S	S

D5Mit1	5	57,148,431	P	P	P	P	P	P	P	P
D5Rat179	5	70,452,899	P	P	P	P	P	P	P	P
D5Mit11	5	86,205,719	P	P	P	P	P	P	P	P
D5Wox4	5	102,331,548	P	P	P	P	P	P	P	P
D5Rat24	5	105,106,199	P	P	P	P	P	P	P	P
D5Mit13	5	136,640,731	P	P	P	P	P	P	P	P
D5Mgh8	5	149,029,982	P	P	P	P	P	P	P	P
D5Wox10	5	164,807,242	P	P	P	P	P	P	P	P
D5Rat48	5	166,237,695	P	P	P	P	P	P	P	P
D6Mit5	6	12,856,626	P	P	P	P	P	P	P	P
D6Rat147	6	24,862,818	P	P	P	P	P	P	P	P
D6Mit9	6	36,260,131	P	P	P	P	P	P	P	P
D6Mit4	6	60,606,186	P	P	P	P	P	P	P	P
D6Rat129	6	76,902,074	P	P	P	P	P	P	P	P
D6Mit2	6	84,763,275	P	P	P	P	P	P	P	P
D6Rat124	6	95,059,735	P	P	P	P	P	P	P	P
D6Mgh4	6	108,154,445	P	P	P	P	P	P	P	P
D6Rat75	6	127,363,380	P	P	P	P	P	P	P	P
D6Mit10	6	134,727,413	P	P	P	P	P	P	P	P
D6Mgh1	6	135,956,771	P	P	P	P	P	P	P	P
D7Rat35	7	24,175,337	P	P	P	W	P	P	P	P
D7Rat92	7	36,695,145	P	P	P	W	P	P	P	P
D7Arb16	7	51,251,919	P	P	P	W	P	P	P	P
D7Wox27	7	66,701,808	P	P	P	W	P	P	P	P
D7Rat69	7	67,801,457	P	P	P	W	P	P	P	P
D7Rat141	7	91,832,949				W				
D7Rat20	7	98,200,188	P	P	P	W	P	P	P	P
D7Mgh5	7	119,130,825	P	P	P	W	P	P	P	P
D7Rat9	7	128,085,642	P	P	P	W	P	P	P	P
D7Rat77	7	134,876,951	P	P	P	W	P	P	P	P
D7Rat94	7	145,692,398	P	P	P	W	P	P	P	P
D8Rat77	8	2,108,128	P	P	P	P	W	P	P	P
D8Rat55	8	8,578,850	P	P	P	P	W	P	P	P
D8Rat56	8	9,531,047	P	P	P	P	W	P	P	P
D8Rat53	8	21,813,070	P	P	P	P	W	P	P	P

D5Rat179	5	70,452,899	S	S	S	S	S	S
D5Mgh11	5	76,608,035	S	S	S	S	S	S
D5Mit11	5	86,205,719	S	S	S	S	S	S
D5Rat24	5	105,106,199	S	S	S	S	S	S
D5Mit13	5	136,640,731	S	S	S	S	S	S
D5Mgh8	5	149,029,982	S	S	S	S	S	S
D6Rat62	6	5,000,456	S	S	S	S	S	S
D6Mit5	6	12,856,626	S	S	S	S	S	S
D6Mit9	6	36,260,131	S	S	S	S	S	S
D6Mit4	6	60,606,186	S	S	S	S	S	S
D6Rat129	6	76,902,074	S	S	S	S	S	S
D6Mit2	6	84,763,275	S	S	S	S	S	S
D6Rat121	6	100,120,078	S	S	S	S	S	S
D6Mgh4	6	108,154,445	S	S	S	S	S	S
D6Rat9	6	126,394,515					S	S
D6Rat75	6	127,363,380	S	S	S	S	S	S
D6Mit10	6	134,727,413	S	S	S	S	S	S
D6Mgh1	6	135,956,771	S	S	S	S	S	S
D6Rat2	6	138,031,220	S	S	S	S	S	S
D7Rat35	7	24,175,337	S	S	S	S	S	S
D7Rat92	7	36,695,145	S	S	S	S	S	S
D7Wox27	7	66,701,808	S	S	S	S	S	S
D7Rat69	7	67,801,457	S	S	S	S	S	S
D7Rat141	7	91,832,949	S	S	S	S	S	S
D7Rat20	7	98,200,188	S	S	S	S	S	S
D7Mgh5	7	119,130,825	S	S	S	S	S	S
D7Rat77	7	134,876,951	S	S	S	S	S	S
D7Rat94	7	145,692,398	S	S	S	S	S	S
D8Rat56	8	9,531,047	S	S	S	S	S	S
D8Rat53	8	21,813,070	S	S	S	S	S	S
D8Rat51	8	30,918,112	S	S	S	S	S	S
D8Rat146	8	74,920,991	S	S	S	S	S	S

D8Tkyo1	8	23,095,882	P		P		W			
D8Rat51	8	30,918,112	P	P	P	P	W	P	P	P
D8Tkyo5	8	58,902,089					W			
D8Rat146	8	74,920,991	P	P	P	P	W	P	P	P
D8Mgh4	8	89,058,229	P	P	P	P	W	P	P	P
D8Rat75	8	91,990,411	P	P	P	P	W	P	P	P
D8Tkyo9	8	100,011,322					W		P	
D8Rat16	8	101,304,999	P	P	P	P	W	P	P	P
D8Mgh1*	8	120,496,008	P	P	P	P	P	P	P	P
D8Tkyo10	8	128,032,912	P	P	P	P	W	P	P	P
D9Rat135	9	13,713,787						W		
D9Wox18	9	25,692,373	P	P	P	P	P	W	P	P
D9Tkyo4	9	27,587,386						W		
D9Tkyo5	9	28,466,437						W		
D9Rat70	9	29,075,079	P	P	P	P	P	W	P	P
D9Rat128	9	29,354,313						W		
D9Rat29	9	29,466,970						W		
D9Rat122	9	62,132,598	P	P	P	P	P	W	P	P
D9Wox4	9	71,268,192	P	P	P	P	P	W	P	P
D9Rat113	9	78,984,875						W		
D9Rat9	9	89,606,579						W		
D9Rat5	9	93,442,819	P	P	P	P	P	W	P	P
D9Rat67	9	94,845,971						W		
D9Rat4	9	98,606,676						W		
D9Rat83	9	99,498,209		P				W		
D9Mit1	9	113,343,366	P	P	P	P	P	W	P	P
D10Rat47	10	16,094,246	P	P	P	P	P	P	P	P
D10Rat182	10	17,555,386	P	P	P	P	P	P	P	P
D10Rat42	10	21,707,766	P	P	P	P	P	P	P	P
D10Rat72	10	23,941,190	P	P	P	P	P	P	P	P
D10Mgh10	10	36,185,929	P	P	P	P	P	P	P	P
D10Mgh6	10	64,648,175	P	P	P	P	P	P	P	P
D10Rat21	10	82,675,233	P	P	P	P	P	P	P	P
D10Rat99	10	88,250,522	P	P	P	P	P	P	P	P
D10Mgh4	10	95,638,234	P	P	P	P	P	P	P	P
D10Rat13	10	95,967,019	P	P	P	P	P	P	P	P
D10Rat7	10	104,329,177	P	P	P	P	P	P	P	P

D8Mgh4	8	89,058,229	S	S	S	S	S	S
D8Rat75	8	91,990,411	S	S	S	S	S	S
D8Rat16	8	101,304,999	S	S	S	S	S	S
D8Mgh1	8	120,496,008	S	S	S	S	S	S
D8Tkyo10	8	128,032,912	S	S	S	S	S	S
D9Rat135	9	13,713,787	S	S	S	S	S	S
D9Tkyo4	9	27,587,386	S	S	S	S	S	S
D9Rat122	9	62,132,598	S	S	S	S	S	S
D9Wox4	9	71,268,192	S	S	S	S	S	S
D9Tkyo11	9	79,270,504	S	S	S	S	S	S
D9Rat5	9	93,442,819	S	S	S	S	S	S
D9Rat83	9	99,498,209	S	S	S	S	S	S
D9Mit1	9	113,343,366	S	S	S	S	S	S
D10Rat182	10	17,555,386				S	S	
D10Rat42	10	21,707,766	S	S	S	S	S	S
D10Mgh10	10	36,185,929	S	S	S	S	S	S
D10Mgh6	10	64,648,175	S	S	S	S	S	S
D10Rat21	10	82,675,233	S	S	S	S	S	S
D10Rat99	10	88,250,522	S	S	S	S	S	S
D10Rat13	10	95,967,019	S	S	S	S	S	S
D10Rat16	10	96,406,074	S	S	S	S	S	S
D10Mgh1	10	[107,490,444]*	S	S	S	S	S	S

D10Mgh1	10	[107,490,444]*	P	P	P	P	P	P	P	P
D11Rat31	11	6,673,351	P	P	P	P	P	P	P	P
D11Rat76	11	27,344,036	P	P	P	P	P	P	P	P
D11Mgh5	11	44,444,112	P	P	P	P	P	P	P	P
D11Mgh4	11	62,653,194	P	P	P	P	P	P	P	P
D11Mgh2	11	79,885,154	P	P	P	P	P	P	P	P
D12Rat63	12	11,986,905	P	P	P	P	P	P	P	P
D12Mit2	12	22,650,702	P	P	P	P	P	P	P	P
D12Mgh5	12	31,723,565	P	P	P	P	P	P	P	P
D12Rat36	12	37,521,884	P	P	P	P	P	P	P	P
D12Mit4	12	43,798,958	P	P	P	P	P	P	P	P
D13Tkyo1	13	26,605,475	P	P	P	P	P	P	W	P
D13Mgh4	13	42,310,744	P	P	P	P	P	P	W	P
D13Rat122	13	45,955,609							W	
D13Rat123	13	48,776,655							W	
D13Rat41	13	49,058,507							W	
D13Wox5	13	50,507,819	P	P	P	P	P	P	W	P
D13Wox6	13	51,576,838	P	P	P	P	P	P	W	P
D13Mgh7	13	67,206,802	P	P	P	P	P	P	W	P
D13Rat85	13	74,568,378							W	
D13Rat56	13	87,815,427	P	P	P	P	P	P	W	P
D13Mgh6	13	103,613,626	P	P	P	P	P	P	W	P
D13Rat51	13	108,770,533							W	
D14Rat2	14	2,813,534	P	P	P	P	P	P	P	P
D14Mgh3	14	20,266,223	P	P	P	P	P	P	P	P
D14Rat24	14	44,088,622	P	P	P	P	P	P	P	P
D14Rat45	14	69,672,685	P	P	P	P	P	P	P	P
D14Mgh2	14	78,270,281	P	P	P	P	P	P	P	P
D14Got87	14	88,876,924	P	P	P	P	P	P	P	P
D14Rat110	14	110,419,380	P	P	P	P	P	P	P	P
D15Rat2	15	11,605,245	P	P	P	P	P	P	P	W
D15Mgh7	15	18,513,913	P	P	P	P	P	P	P	W
D15Mit3	15	18,093,208	P	P	P	P	P	P	P	W
D15Rat6	15	63,498,932	P	P	P	P	P	P	P	W
D15Tkyo8	15	42,655,149	P	P	P	P	P	P	P	W
D15Rat61	15	50,700,465	P	P	P	P	P	P	P	W

D11Rat76	11	27,344,036	S	S	S	S	S	S
D11Rat75	11	30,537,317		S			S	
D11Rat11	11	33,739,895	S	S	S	S	S	S
D11Mgh5	11	44,444,112	S	S	S	S	S	S
D11Mgh4	11	62,653,194	S	S	S	S	S	S
D11Mgh2	11	79,885,154	S	S	S	S	S	S
D12Rat63	12	11,986,905	S	S	S	S	S	S
D12Mit2	12	22,650,702	S	S	S	S	S	S
D12Mgh5	12	31,723,565	S	S	S	S	S	S
D12Rat36	12	37,521,884	S	S	S	S	S	S
D12Mit4	12	43,798,958	S	S	S	S	S	S
D13Tkyo1	13	26,605,475	S	S	S	S	S	S
D13Mgh4	13	42,310,744	S	S	S	S	S	S
D13Rat41	13	49,058,507	S	S	S	S	S	S
D13Wox5	13	50,507,819	S	S	S	S	S	S
D13Wox6	13	51,576,838	S	S	S	S	S	S
D13Mgh7	13	67,206,802	S	S	S	S	S	S
D13Mgh9	13	86,272,806	S	S	S	S	S	S
D13Rat49	13	106,676,710	S	S	S	S	S	S
D13Rat51	13	108,770,533	S	S	S	S	S	S
D14Rat2	14	2,813,534	S	S	S	S	S	S
D14Mgh3	14	20,266,223	S	S	S	S	S	S
D14Rat24	14	44,088,622	S	S	S	S	S	S
D14Mgh2	14	78,270,281	S	S	S	S	S	S
D14Got87	14	88,876,924	S	S	S	S	S	S
D14Mco5	14	103,778,284	S	S	S	S	S	S
D15Mgh7	15	18,513,913	S	S	S	S	W	S
D15Mit3	15	18,093,208	S	S	S	S	W	S
D15Tkyo6	15	34,385,010					W	
D15Rat68	15	63,498,932	S	S	S	S	W	S
D15Rat95	15	70,559,420					W	
D15Rat94	15	77,072,146	S	S	S	S	W	S

Disease Models & Mechanisms • Supplementary information

D15Rat92	15	54,656,296								W
D15Rat20	15	59,616,275								W
D15Rat68	15	63,498,932	P	P	P	P	P	P	P	W
D15Arb2	15	68,366,508	P	P	P	P	P	P	P	W
D15Rat94	15	77,072,146	P	P	P	P	P	P	P	W
D15Tkyo18	15	88,011,655	P	P	P	P	P	P	P	W
D15Mgh6	15	104,003,455	P	P	P	P	P	P	P	W
D15Rat106	15	106,550,444								W
D16Rat22	16	19,398,173	P	P	P	P	P	P	P	P
D16Wox10	16	19,398,173	P	P	P	P	P	P	P	P
D16Mit3	16	47,346,470	P	P	P	P	P	P	P	P
D16Rat64	16	59,285,775	P	P	P	P	P	P	P	P
D16Rat58	16	69,058,102	P	P	P	P	P	P	P	P
D16Rat108	16	71,460,724	P	P	P	P	P	P	P	P
D16Rat93	16	85,005,570	P	P	P	P	P	P	P	P
D16Rat15	16	80,316,290	P	P	P	P	P	P	P	P
D17Mgh1	17	396,662	P	P	P	P	P	P	P	P
D17Rat6	17	10,529,709	P	P	P	P	P	P	P	P
D17Rat143	17	15,134,275	P	P	P	P	P	P	P	P
D17Rat12	17	27,690,959	P	P	P	P	P	P	P	P
D17Mgh4	17	46,867,215	P	P	P	P	P	P	P	P
D17Mit4	17	63,994,205	P	P	P	P	P	P	P	P
D17Rat99	17	81,334,009	P	P	P	P	P	P	P	P
D17Rat49	17	85,236,284	P	P	P	P	P	P	P	P
D18Rat48	18	5,950,917	P	P	P	P	P	P	P	P
D18Wox7	18	15,539,551	P	P	P	P	P	P	P	P
D18Rat105	18	17,807,783	P	P	P	P	P	P	P	P
D18Mit4	18	27,728,134	P	P	P	P	P	P	P	P
D18Rat57	18	53,861,252	P	P	P	P	P	P	P	P
D18Rat41	18	58,476,128	P	P	P	P	P	P	P	P
D18Mit8	18	61,985,648	P	P	P	P	P	P	P	P
D18Rat123	18	70,370,954	P	P	P	P	P	P	P	P
D18Mgh4	18	79,948,616	P	P	P	P	P	P	P	P
D19Mit3	19	15,702,328	P	P	P	P	P	P	P	P
D19Rat101	19	15,939,528	P	P	P	P	P	P	P	P
D19Wox3	19	31,194,301	P	P	P	P	P	P	P	P
D19Wox4	19	41,685,949	P	P	P	P	P	P	P	P

D15Tkyo18	15	88,011,655	S	S	S	S	W	S
D15Rat102	15	98,027,558					W	
D15Mgh6	15	104,003,455	S	S	S	S	W	S
D15Rat106	15	106,550,444					W	S
D16Rat22	16	19,398,173	S	S	S	S	S	S
D16Wox10	16	19,398,173	S	S	S	S	S	S
D16Mit3	16	47,346,470	S	S	S	S	S	S
D16Rat58	16	69,058,102	S	S	S	S	S	S
D16Rat93	16	85,005,570	S	S	S	S	S	S
D16Rat15	16	80,316,290	S	S	S	S	S	S
D17Rat6	17	10,529,709	S	S	S	S	S	S
D17Rat61	17	14,009,679	S	S	S	S	S	S
D17Rat12	17	27,690,959	S	S	S	S	S	S
D17Mgh4	17	46,867,215	S	S	S	S	S	S
D17Mit4	17	63,994,205	S	S	S	S	S	S
D17Rat49	17	85,236,284	S	S	S	S	S	S
D18Rat48	18	5,950,917	S	S	S	S	S	S
D18Wox7	18	15,539,551	S	S	S	S	S	S
D18Rat57	18	53,861,252	S	S	S	S	S	S
D18Mit8	18	61,985,648	S	S	S	S	S	S
D18Mgh4	18	79,948,616	S	S	S	S	S	S
D19Mit3	19	15,702,328	S	S	S	S	S	W
D19Rat15	19	15,721,763	S	S	S	S	S	W
D19Rat101	19	15,939,528	S	S	S	S	S	W
D19Rat48	19	30,683,540	S	S	S	S	S	W

Disease Models & Mechanisms • Supplementary information

D19Mgh3	19	48,139,992	P	P	P	P	P	P	P	P
D19Rat57	19	60,220,451	P	P	P	P	P	P	P	P
D20Mgh5	20	9,655,642	P	P	P	P	P	P	P	P
D20Rat4	20	12,482,277	P						P	
D20Rat11	20	40,871,079	P	P	P	P	P	P	P	P
D20Rat29	20	49,108,730	P	P	P	P	P	P	P	P
D20Mgh1	20	51,892,390	P	P	P	P	P	P	P	P
DXMit1	X	12,150,263	P	P	P	P	P	P	P	P
DXMit4	X	61,397,905	P	P	P	P	P	P	P	P
DXRat17	X	95,021,031	P	P	P	P	P	P	P	P
DXRat18	X	106,240,541	P	P	P	P	P	P	P	P
DXRat19	X	114,961,056	P	P	P	P	P	P	P	P
DXMgh7	X	119,394,326	P	P	P	P	P	P	P	P

D19Wox3	19	31,194,301	S	S	S	S	S	W
D19Rat35	19	41,471,460						W
D19Wox4	19	41,685,949	S	S	S	S	S	W
D19Rat7	19	49,873,186	S	S	S	S	S	W
D19Rat58	19	56,723,500						W
D19Rat5	19	57,094,703						W
D19Rat104	19	58,308,034	S	S	S	S	S	W
D19Rat57	19	60,220,451						W
D20Rat31	20	10,800,308	S	S	S	S	S	S
D20Rat4	20	12,482,277				S	S	
D20Rat11	20	40,871,079	S	S	S	S	S	S
D20Rat29	20	49,108,730	S	S	S	S	S	S
D20Mgh1	20	51,892,390	S	S	S	S	S	S
DXMit1	X	12,150,263	S	S	S	S	S	S
DXMit4	X	61,397,905	S	S	S	S	S	S
DXRat17	X	95,021,031	S	S	S	S	S	S
DXRat18	X	106,240,541	S	S	S	S	S	S
DXMgh7	X	119,394,326	S	S	S	S	S	S

Allele designation: P, SHRSP/Izm; S, SHR/Izm; W, WKY/Izm.

The position of D10Mgh1 is arbitrarily mapped to the rn6 reference genome (NCBI) using the information for its neighboring SSLP markers (D10Rat7 and D10Rat13) on the Celera map.

*In the RNO8 consomic strain, a chromosomal segment (<27 Mb) remains to exist, interrupting the congenic fragment.

Table S8A. Phenotype comparison in male rats between progenitor and consomic/congenic strains involving SHRSP or SHR and WKY

Strain	*n*	Hw (g)		Hw/Bw (‰)		12wk BP (mmHg)		16wk BP (mmHg)		16wk Bw (g)		T.adip./BW (‰)		P.adip./BW (‰)		FPG		T.chol		TG		NEFA	
		mean ± SE	P, vs SHRSP*	mean ± SE	P, vs SHRSP*	mean ± SE	P, vs SHRSP*	mean ± SE	P, vs SHRSP*	mean ± SE	P, vs SHRSP*	mean ± SE	P, vs SHRSP*	mean ± SE	P, vs SHRSP*	mean ± SE	P, vs SHRSP*	mean ± SE	P, vs SHRSP	mean ± SE	P, vs SHRSP	mean ± SE	P, vs SHRSP
SHRSP	38-173	1.07 ± 0.01	—	3.82 ± 0.03	—	211.0 ± 1.0	—	236.6 ± 1.5	—	283.9 ± 1.5	—	11.05 ± 0.22	—	8.22 ± 0.23	—	133.9 ± 3.3	—	52.2 ± 1.0	—	15.7 ± 0.8	—	1.12 ± 0.06	—
WKY	10-27	0.98 ± 0.01	—	2.61 ± 0.03	—	130.3 ± 2.0	—	123.4 ± 2.3	—	366.6 ± 4.6	—	13.25 ± 0.31	—	10.72 ± 0.34	—	108.3 ± 4.5	—	83.1 ± 3.1	—	12.5 ± 1.3	—	1.30 ± 0.06	—
1pW	58-132	1.02 ± 0.01	1.1E-04	3.51 ± 0.02	4.4E-12	195.7 ± 1.0	7.3E-24	207.6 ± 1.3	3.3E-34	305.5 ± 2.0	1.1E-15	8.00 ± 0.12	2.8E-17	7.25 ± 0.12	4.0E-04	112.2 ± 2.6	8.5E-07	46.4 ± 0.8	1.9E-05	8.4 ± 0.4	1.9E-11	0.97 ± 0.04	0.048
3pW	40-67	1.01 ± 0.01	4.3E-04	3.50 ± 0.02	6.0E-12	192.7 ± 1.6	1.3E-16	200.7 ± 1.7	1.7E-31	285.1 ± 2.4	0.510	9.45 ± 0.20	1.3E-06	9.42 ± 0.17	5.9E-05	127.1 ± 2.5	0.103	39.8 ± 0.6	6.8E-16	8.8 ± 0.5	2.5E-10	0.98 ± 0.04	0.066
4pW	32-80	0.99 ± 0.01	4.8E-08	3.47 ± 0.02	2.4E-13	191.2 ± 1.2	7.4E-29	202.4 ± 1.8	6.1E-32	287.4 ± 2.2	0.128	9.61 ± 0.21	1.3E-05	8.14 ± 0.18	0.773	138.2 ± 2.6	0.303	47.5 ± 0.6	2.1E-04	10.9 ± 0.5	2.6E-06	1.17 ± 0.05	0.511
7pW	24-49	1.00 ± 0.01	2.5E-05	3.53 ± 0.03	9.3E-09	204.8 ± 1.6	9.5E-04	222.9 ± 2.7	4.8E-05	291.5 ± 2.7	0.011	9.56 ± 0.22	1.3E-05	6.93 ± 0.17	2.9E-05	149.5 ± 3.1	7.2E-04	43.7 ± 0.7	1.2E-09	9.0 ± 0.4	2.3E-10	0.84 ± 0.04	2.4E-04
8pW	27-68	1.08 ± 0.01	0.646	3.78 ± 0.03	0.451	210.2 ± 1.6	0.638	232.4 ± 2.3	0.104	279.9 ± 3.2	0.364	11.22 ± 0.19	0.556	8.77 ± 0.14	0.043	142.7 ± 3.0	0.051	46.3 ± 0.6	5.9E-06	14.6 ± 0.8	0.337	1.02 ± 0.05	0.231
9pW	33-70	1.03 ± 0.01	0.025	3.47 ± 0.02	6.0E-13	202.7 ± 1.4	2.5E-06	215.3 ± 1.7	3.5E-17	302.1 ± 2.5	8.7E-09	9.19 ± 0.31	7.2E-06	8.65 ± 0.25	0.207	134.5 ± 2.6	0.886	51.0 ± 0.5	0.301	12.0 ± 0.5	2.5E-04	0.95 ± 0.05	0.029
13pW	26-47	1.01 ± 0.01	5.0E-06	3.50 ± 0.02	3.4E-12	201.8 ± 2.1	1.7E-04	218.0 ± 2.0	2.8E-10	294.7 ± 2.4	2.1E-04	9.59 ± 0.21	1.5E-05	7.83 ± 0.30	0.300	131.8 ± 3.0	0.642	57.7 ± 0.8	7.5E-05	10.1 ± 0.5	1.6E-07	0.94 ± 0.05	0.028
15pW	24-102	0.96 ± 0.01	1.8E-13	3.51 ± 0.03	4.3E-10	202.1 ± 2.0	7.3E-05	213.1 ± 1.7	2.0E-20	287.5 ± 1.9	0.094	9.65 ± 0.20	1.7E-04	8.36 ± 0.21	0.692	141.0 ± 3.5	0.096	64.7 ± 0.7	9.1E-16	13.1 ± 0.6	0.011	1.01 ± 0.04	0.132
1pWC	27-91	—	—	—	—	182.6 ± 1.3	1.5E-42	187.8 ± 1.1	4.6E-58	288.6 ± 2.6	0.122	—	—	—	—	116.8 ± 3.8	0.001	37.5 ± 0.8	7.6E-18	9.8 ± 0.5	2.4E-08	1.01 ± 0.08	0.262
1pWC×9pW	57-60	0.95 ± 0.06	—	3.19 ± 0.07	—	174.3 ± 1.3	1.4E-05	176.8 ± 1.4	6.5E-09	301.2 ± 4.0	0.003	9.93 ± 0.17	—	8.51 ± 0.22	—	137.4 ± 2.0	1.1E-05	—	—	—	—	—	—
1pWC×13pW	68-81	0.92 ± 0.05	—	3.21 ± 0.01	—	172.0 ± 1.2	6.6E-09	173.8 ± 1.1	2.5E-15	284.1 ± 1.5	0.136	9.93 ± 0.11	—	7.72 ± 0.12	—	137.6 ± 1.7	5.9E-06	—	—	—	—	—	—
SHR	23-74	1.11 ± 0.01	—	3.42 ± 0.04	—	186.1 ± 1.6	—	197.6 ± 2.0	—	322.1 ± 2.1	—	12.00 ± 0.33	—	9.74 ± 0.33	—	123.8 ± 3.5	—	59.9 ± 0.9	—	12.4 ± 0.5	—	0.98 ± 0.04	—
1SW	58-132	1.12 ± 0.01	0.512	3.31 ± 0.02	0.013	182.9 ± 1.3	0.116	191.8 ± 1.3	0.017	342.0 ± 2.3	2.2E-09	8.50 ± 0.16	5.4E-11	7.36 ± 0.18	3.4E-07	112.8 ± 2.3	0.010	60.4 ± 0.9	0.709	9.7 ± 0.4	7.2E-05	1.16 ± 0.06	0.030
2SW	27-91	1.12 ± 0.01	0.547	3.35 ± 0.02	0.106	170.2 ± 1.5	5.6E-11	180.7 ± 2.0	2.7E-08	330.8 ± 2.7	0.014	9.47 ± 0.20	9.7E-08	7.89 ± 0.20	2.8E-05	137.7 ± 2.3	0.001	63.3 ± 1.0	0.013	13.9 ± 0.6	0.063	1.20 ± 0.04	0.001
3SW	53-67	1.03 ± 0.01	3.0E-06	3.27 ± 0.02	0.002	176.3 ± 1.1	2.5E-06	183.5 ± 1.3	4.0E-08	316.7 ± 2.0	0.066	9.81 ± 0.18	1.2E-06	9.89 ± 0.18	0.702	115.1 ± 2.4	0.044	47.3 ± 1.1	6.4E-14	6.8 ± 0.2	2.0E-14	1.05 ± 0.05	0.312
4SW	26-103	0.99 ± 0.01	7.5E-11	3.28 ± 0.02	0.002	182.9 ± 1.7	0.172	190.8 ± 1.4	0.006	303.4 ± 2.3	3.7E-08	10.30 ± 0.24	1.5E-04	8.77 ± 0.24	0.022	118.0 ± 2.9	0.207	54.0 ± 0.8	1.6E-06	13.3 ± 0.8	0.295	1.16 ± 0.04	0.004
15SW	22-75	0.90 ± 0.02	9.3E-13	3.15 ± 0.02	1.7E-07	168.7 ± 2.1	8.9E-11	172.3 ± 1.9	1.8E-15	310.9 ± 3.0	0.003	11.14 ± 0.33	0.090	9.18 ± 0.36	0.289	137.8 ± 3.2	0.004	64.1 ± 1.2	0.006	13.3 ± 0.9	0.395	0.94 ± 0.03	0.380
19SW	28-103	1.09 ± 0.02	0.400	3.43 ± 0.10	0.932	178.6 ± 1.7	0.002	188.2 ± 1.6	4.2E-04	323.0 ± 2.8	0.799	10.68 ± 0.21	0.002	10.88 ± 0.26	0.010	133.2 ± 3.6	0.062	65.9 ± 1.0	1.8E-05	13.6 ± 0.9	0.230	0.87 ± 0.04	0.053
1SWC	50-126	1.12 ± 0.01	0.241	3.43 ± 0.02	0.773	168.8 ± 1.2	4.9E-14	167.8 ± 1.0	7.8E-22	328.9 ± 1.6	0.011	7.91 ± 0.12	2.9E-12	6.78 ± 0.16	3.5E-09	191.0 ± 3.5	3.2E-27	—	—	—	—	—	—
4SWC(1)	33-49	1.06 ± 0.01	0.011	3.33 ± 0.02	0.056	175.8 ± 1.8	4.6E-05	183.9 ± 2.1	8.4E-06	317.6 ± 3.2	0.241	10.71 ± 0.19	0.002	8.50 ± 0.17	0.002	136.5 ± 2.3	0.003	63.3 ± 0.9	0.010	11.3 ± 0.7	0.186	1.30 ± 0.04	9.3E-07

Hw, heart weight; Hw/Bw, heart weight divided by body weight; BP, blood pressure; T.adip, peri-testis fat pad; P.adip, retroperitoneal fat pad; FPG, fasting plasma glucose; T.chol, total cholesterol; TG, triglycerides; NEFA, non-esterified fatty acid.
For double congenic rats (1pWC×9pW and 1pWC×13pW), *t*-test was performed in comparison with 1pWC, into which another chromosomal regions (RNO9 and RNO13) were introgressed.

Table S8B. Phenotype comparison in female rats between progenitor and consomic/congenic strains involving SHRSP or SHR and WKY

Strain	*n*	Hw (g)		Hw/Bw (‰)		12wk BP (mmHg)		16wk BP (mmHg)		16wk Bw (g)		T.adip./BW (‰)		P.adip./BW (‰)		FPG		T.chol		TG		NEFA	
		mean ± SE	P, vs SHRSP	mean ± SE	P, vs SHRSP*	mean ± SE	P, vs SHRSP*	mean ± SE	P, vs SHRSP*	mean ± SE	P, vs SHRSP*			mean ± SE	P, vs SHRSP*	mean ± SE	P, vs SHRSP*	mean ± SE	P, vs SHRSP	mean ± SE	P, vs SHRSP	mean ± SE	P, vs SHRSP
SHRSP	35-195	0.72 ± 0.01	—	3.86 ± 0.02	—	176.3 ± 0.8	—	191.3 ± 1.1	—	189.4 ± 0.9	—			6.92 ± 0.26	—	129.5 ± 1.9	—	69.7 ± 1.0	—	15.7 ± 0.6	—	0.88 ± 0.03	—
WKY	10-15	0.62 ± 0.01	—	2.66 ± 0.03	—	116.0 ± 1.8	—	118.8 ± 2.3	—	227.2 ± 2.2	—			11.43 ± 0.40	—	108.9 ± 4.0	—	89.9 ± 3.1	—	15.3 ± 0.6	—	1.18 ± 0.08	—
1pW	30-123	0.72 ± 0.01	0.491	3.67 ± 0.02	6.9E-13	166.2 ± 1.0	9.6E-14	174.6 ± 1.0	1.0E-22	198.7 ± 1.0	5.0E-12			6.41 ± 0.14	0.084	117.8 ± 2.7	5.7E-04	78.0 ± 1.2	1.3E-06	11.2 ± 0.8	1.5E-05	1.25 ± 0.06	8.0E-07
3pW	31-64	0.73 ± 0.01	0.533	3.80 ± 0.03	0.120	166.3 ± 1.4	4.5E-09	167.5 ± 1.9	2.5E-17	187.3 ± 1.5	0.227			9.64 ± 0.17	2.6E-12	128.9 ± 2.5	0.841	63.3 ± 1.5	8.2E-04	12.3 ± 0.6	8.9E-05	0.91 ± 0.05	0.604
4pW	23-91	0.69 ± 0.01	0.024	3.56 ± 0.02	4.6E-15	162.3 ± 1.3	8.3E-17	170.7 ± 1.2	3.6E-26	194.6 ± 1.4	0.001			7.27 ± 0.22	0.307	142.8 ± 3.0	2.7E-04	61.9 ± 1.4	2.0E-05	10.9 ± 0.5	1.6E-08	0.91 ± 0.04	0.441
7pW	33-58	0.70 ± 0.01	0.026	3.72 ± 0.02	4.9E-08	171.5 ± 1.3	0.002	182.6 ± 1.3	5.9E-06	189.6 ± 1.2	0.850			5.31 ± 0.19	5.0E-06	141.0 ± 2.9	0.001	65.2 ± 1.1	0.002	11.6 ± 0.4	7.9E-08	0.96 ± 0.04	0.089
8pW	25-84	0.74 ± 0.01	0.093	3.90 ± 0.03	0.237	182.7 ± 1.4	0.002	193.6 ± 1.3	0.226	191.5 ± 1.4	0.782			6.69 ± 0.18	0.464	132.0 ± 2.6	0.030	65.1 ± 0.9	0.004	16.0 ± 0.7	0.670	1.05 ± 0.04	0.001
9pW	23-72	0.73 ± 0.01	0.684	3.68 ± 0.03	1.8E-06	168.7 ± 1.3	3.0E-06	178.1 ± 1.7	7.9E-09	197.5 ± 1.7	4.9E-05			8.21 ± 0.30	0.002	133.4 ± 3.0	0.272	63.3 ± 1.6	0.001	13.2 ± 0.5	0.002	0.92 ± 0.05	0.496
13pW	32-47	0.71 ± 0.01	0.178	3.65 ± 0.03	1.2E-08	167.5 ± 1.8	4.0E-05	172.3 ± 1.8	4.9E-12	195.3 ± 1.5	0.001			5.98 ± 0.16	0.003	136.1 ± 3.3	0.083	73.8 ± 1.3	0.015	13.5 ± 0.5	0.006	1.18 ± 0.03	2.6E-11
15pW	30-121	0.67 ± 0.01	1.3E-05	3.60 ± 0.02	6.5E-14	174.8 ± 1.7	0.424	179.6 ± 1.2	1.0E-10	186.3 ± 0.9	0.015			6.31 ± 0.28	0.083	141.0 ± 2.5	4.1E-04	90.1 ± 1.7	1.0E-14	16.7 ± 0.9	0.347	0.94 ± 0.04	0.157
1pWC	24-110	—	—	—	—	154.1 ± 0.9	9.4E-49	158.0 ± 1.2	2.6E-46	188.1 ± 1.3	0.411			—	—	127.2 ± 2.3	0.443	62.4 ± 1.6	3.3E-04	12.6 ± 0.7	0.001	1.09 ± 0.04	6.9E-05
1pWC×9pW	74-85	0.67 ± 0.01	—	3.46 ± 0.03	—	147.5 ± 1.0	1.6E-06	152.6 ± 1.1	0.002	194.7 ± 1.5	0.001			7.59 ± 0.14	—	141.7 ± 1.8	3.6E-06	—	—	—	—	—	—
1pWC×13pW	45-65	0.63 ± 0.01	—	3.51 ± 0.02	—	143.4 ± 1.2	1.3E-10	147.0 ± 1.5	1.1E-07	180.2 ± 1.2	1.6E-05			6.62 ± 0.20	—	147.1 ± 1.5	3.4E-10	—	—	—	—	—	—
SHR	26-85	0.74 ± 0.01	—	3.83 ± 0.05	—	161.9 ± 1.3	—	173.6 ± 1.6	—	190.9 ± 1.2	—			7.29 ± 0.19	—	120.5 ± 3.2	—	69.0 ± 1.1	—	10.8 ± 0.3	—	1.13 ± 0.04	—
1SW	32-125	0.73 ± 0.01	0.221	3.62 ± 0.02	0.001	163.2 ± 1.2	0.482	167.8 ± 1.5	0.010	207.5 ± 1.5	1.7E-14			6.67 ± 0.19	0.025	115.1 ± 2.5	0.180	76.3 ± 1.9	0.002	13.2 ± 0.5	2.0E-04	0.97 ± 0.04	0.007
2SW	24-110	0.76 ± 0.01	0.057	3.74 ± 0.03	0.129	153.0 ± 1.1	1.2E-06	159.0 ± 2.0	2.1E-07	198.4 ± 2.0	0.002			5.99 ± 0.17	5.8E-06	141.2 ± 1.8	2.3E-07	86.2 ± 1.7	2.2E-12	14.5 ± 0.6	8.1E-07	1.16 ± 0.04	0.600
3SW	53-78	0.68 ± 0.005	1.2E-07	3.58 ± 0.02	5.4E-05	154.2 ± 1.1	1.1E-05	155.7 ± 1.4	7.1E-14	189.9 ± 1.3	0.564			8.86 ± 0.18	8.9E-08	120.9 ± 2.9	0.937	65.7 ± 1.1	0.039	9.1 ± 0.2	9.6E-06	0.99 ± 0.04	0.010
4SW	30-122	0.70 ± 0.01	0.001	3.69 ± 0.02	0.015	158.9 ± 1.6	0.157	166.7 ± 1.5	0.002	189.8 ± 2.1	0.634			7.28 ± 0.21	0.969	118.7 ± 3.2	0.692	74.4 ± 1.6	0.008	11.3 ± 0.5	0.417	1.32 ± 0.06	0.009
15SW	28-106	0.64 ± 0.01	3.0E-12	3.58 ± 0.03	1.4E-04	146.2 ± 1.6	3.6E-12	144.0 ± 1.4	7.7E-28	191.7 ± 1.4	0.663			6.95 ± 0.25	0.282	140.3 ± 2.7	7.2E-06	86.0 ± 1.1	2.4E-17	12.4 ± 0.5	0.008	1.15 ± 0.03	0.618
19SW	26-79	0.72 ± 0.01	0.026	3.53 ± 0.04	1.8E-05	154.6 ± 1.5	3.9E-04	161.1 ± 1.7	6.5E-07	202.4 ± 2.4	5.6E-05			8.14 ± 0.25	0.009	136.3 ± 2.6	2.2E-04	70.8 ± 1.4	0.318	9.6 ± 0.4	0.024	1.09 ± 0.04	0.508
1SWC	28-64	0.76 ± 0.01	0.046	3.77 ± 0.02	0.319	148.3 ± 1.4	2.1E-10	152.9 ± 1.3	2.1E-17	203.7 ± 1.2	6.5E-13			5.72 ± 0.12	1.0E-08	153.7 ± 3.4	5.6E-11	—	—	—	—	—	—
4SWC(1)	32-38	0.70 ± 0.01	2.8E-04	3.72 ± 0.03	0.083	156.0 ± 1.8	0.011	163.2 ± 1.7	3.2E-05	188.2 ± 2.0	0.262			6.75 ± 0.18	0.045	131.9 ± 1.8	0.002	77.2 ± 1.4	3.4E-05	8.9 ± 0.4	9.6E-04	1.34 ± 0.04	5.4E-04

Hw, heart weight; Hw/Bw, heart weight divided by body weight; BP, blood pressure; T.adip, peri-testis fat pad; P.adip, retroperitoneal fat pad; FPG, fasting plasma glucose; T.chol, total cholesterol; TG, triglycerides; NEFA, non-esterified fatty acid.
For double congenic rats (1pWC×9pW and 1pWC×13pW), *t*-test was performed in comparison with 1pWC, into which another chromosomal regions (RNO9 and RNO13) were introgressed.

Table S9A. Phenotype comparison in male rats between progenitor and RNO1 congenic strains involving SHRSP and WKY

Strain	*n*	Hw (g)		Hw/Bw (‰)		12wk BP (mmHg)		16wk BP (mmHg)		20wk BP (mmHg)		12wk Bw (g)		16wk Bw (g)		20wk Bw (g)		T.adip./BW (‰)		P.adip./BW (‰)		FPG		T.chol		TG		NEFA	
		mean ± SE	P, vs SHRSP	mean ± SE	P, vs SHRSP	mean ± SE	P, vs SHRSP	mean ± SE	P, vs SHRSP	mean ± SE	P, vs SHRSP	mean ± SE	P, vs SHRSP	mean ± SE	P, vs SHRSP	mean ± SE	P, vs SHRSP	mean ± SE	P, vs SHRSP	mean ± SE	P, vs SHRSP	mean ± SE	P, vs SHRSP	mean ± SE	P, vs SHRSP	mean ± SE	P, vs SHRSP	mean ± SE	P, vs SHRSP
SHRSP	38-173	1.07 ± 0.01	—	3.82 ± 0.03	—	211.0 ± 1.0	—	236.6 ± 1.5	—	239.5 ± 2.4	—	251.4 ± 1.4	—	283.9 ± 1.5	—	305.2 ± 3.0	—	11.05 ± 0.22	—	8.22 ± 0.23	—	133.9 ± 3.3	—	52.2 ± 1.0	—	15.7 ± 0.8	—	1.12 ± 0.06	—
WKY	10-27	0.98 ± 0.01	—	2.61 ± 0.03	—	130.3 ± 2.0	—	123.4 ± 2.3	—	129.1 ± 2.0	—	312.8 ± 2.3	—	366.6 ± 4.6	—	397.4 ± 8.4	—	13.25 ± 0.31	—	10.72 ± 0.34	—	108.3 ± 4.5	—	83.1 ± 3.1	—	12.5 ± 1.3	—	1.30 ± 0.06	—
1pW	58-132	1.02 ± 0.01	1.1E-04	3.51 ± 0.02	4.4E-12	195.7 ± 1.0	7.3E-24	207.6 ± 1.3	3.3E-34	202.8 ± 1.8	4.7E-23	269.4 ± 1.6	2.7E-16	305.5 ± 2.0	1.1E-15	344.4 ± 2.0	6.2E-20	8.00 ± 0.12	2.8E-17	7.25 ± 0.12	4.0E-04	112.2 ± 2.6	8.5E-07	46.4 ± 0.8	1.9E-05	8.4 ± 0.4	1.9E-11	0.97 ± 0.04	0.048
1pWC	27-91	—	—	—	—	182.6 ± 1.3	1.5E-42	187.8 ± 1.1	4.6E-58	192.2 ± 1.4	8.5E-33	247.9 ± 1.8	0.118	288.6 ± 2.6	0.122	311.1 ± 2.4	0.124	—	—	—	—	116.8 ± 3.8	0.001	37.5 ± 0.8	7.6E-18	9.8 ± 0.5	2.4E-08	1.01 ± 0.08	0.262
1pW2	26-103	—	—	—	—	192.1 ± 1.3	3.1E-25	199.4 ± 1.7	1.5E-32	206.0 ± 2.2	1.5E-18	248.2 ± 2.0	0.182	274.1 ± 3.0	0.005	296.8 ± 3.0	0.049	—	—	—	—	114.2 ± 2.6	9.3E-06	43.8 ± 1.0	1.4E-07	10.9 ± 0.5	5.8E-06	0.97 ± 0.08	0.149
1pW5-2	26-70	—	—	—	—	195.8 ± 1.4	2.6E-15	201.6 ± 2.1	1.7E-25	207.2 ± 1.8	2.9E-19	256.9 ± 2.6	0.062	285.9 ± 2.7	0.528	308.6 ± 3.2	0.432	—	—	—	—	147.2 ± 2.7	0.002	44.5 ± 1.4	5.1E-05	11.0 ± 1.0	5.1E-04	1.08 ± 0.05	0.680
1pW4-1	31-82	1.07 ± 0.02	0.989	3.69 ± 0.03	0.004	202.0 ± 1.4	3.6E-07	214.9 ± 1.7	2.2E-16	216.1 ± 2.2	7.6E-11	250.9 ± 2.2	0.823	281.9 ± 2.1	0.449	299.3 ± 3.0	0.161	11.07 ± 0.36	0.954	8.83 ± 0.30	0.112	134.8 ± 3.7	0.858	48.5 ± 1.0	0.012	10.3 ± 0.7	2.0E-06	1.09 ± 0.08	0.780
1pW4-2	27-70	—	—	—	—	201.2 ± 1.8	7.9E-06	209.8 ± 1.4	2.4E-26	219.3 ± 1.5	1.1E-10	253.2 ± 2.1	0.480	285.9 ± 2.4	0.470	308.5 ± 2.3	0.379	—	—	—	—	132.7 ± 2.5	0.778	43.1 ± 1.0	1.9E-08	8.3 ± 0.3	9.5E-12	1.00 ± 0.07	0.219
1pW4-3	62-100	1.09 ± 0.01	0.150	3.93 ± 0.03	0.011	202.9 ± 1.4	2.0E-06	219.9 ± 2.0	1.7E-10	222.1 ± 2.9	3.6E-06	256.4 ± 2.7	0.046	279.0 ± 1.7	0.038	286.1 ± 4.2	1.6E-05	10.11 ± 0.30	0.015	7.44 ± 0.21	0.013	150.7 ± 3.6	1.1E-04	—	—	—	—	—	—
1pW4-4	60-93	1.12 ± 0.02	0.015	4.01 ± 0.05	0.003	197.2 ± 1.6	3.2E-11	208.2 ± 3.0	2.7E-13	218.0 ± 3.0	2.0E-07	253.1 ± 2.2	0.491	278.5 ± 1.9	0.032	292.9 ± 3.8	0.004	9.86 ± 0.35	0.008	7.43 ± 0.23	0.019	141.9 ± 3.2	0.056	—	—	—	—	—	—
1pW6-2	25-62	—	—	—	—	197.0 ± 1.8	1.3E-09	207.7 ± 1.6	3.4E-26	216.0 ± 1.9	1.5E-12	245.1 ± 3.6	0.117	290.3 ± 3.5	0.098	306.8 ± 4.1	0.746	—	—	—	—	130.3 ± 3.1	0.432	42.8 ± 0.7	1.2E-10	11.6 ± 0.7	3.5E-04	1.11 ± 0.06	0.911
1pW6-1	26-60	0.99 ± 0.01	2.2E-05	3.54 ± 0.02	2.6E-09	200.2 ± 1.5	2.8E-08	212.1 ± 1.8	4.2E-18	219.8 ± 1.6	5.8E-10	256.7 ± 2.3	0.049	286.0 ± 2.2	0.441	305.7 ± 2.6	0.902	9.85 ± 0.29	0.092	7.18 ± 0.23	0.039	134.4 ± 3.6	0.918	41.3 ± 0.9	1.5E-11	9.2 ± 0.5	2.4E-09	1.09 ± 0.05	0.744
1pW3-2	28-103	—	—	—	—	189.7 ± 1.1	4.8E-34	194.2 ± 1.4	6.6E-50	204.4 ± 1.6	8.0E-23	252.4 ± 1.7	0.655	287.2 ± 2.0	0.192	295.5 ± 5.1	0.102	—	—	—	—	123.4 ± 4.0	0.046	38.5 ± 0.7	1.4E-16	12.7 ± 0.6	0.005	0.96 ± 0.05	0.049
1pW3-1	25-116	—	—	—	—	198.0 ± 1.2	1.2E-14	212.5 ± 1.6	4.3E-21	216.3 ± 2.4	4.4E-10	253.2 ± 2.3	0.516	284.0 ± 2.6	0.965	305.4 ± 3.4	0.962	—	—	—	—	127.0 ± 3.0	0.127	39.2 ± 0.7	8.3E-16	13.7 ± 0.6	0.059	0.91 ± 0.04	0.005
1pW8	28-64	—	—	—	—	208.8 ± 1.6	0.251	223.9 ± 3.1	4.3E-04	237.1 ± 3.0	0.525	245.9 ± 3.0	0.097	276.1 ± 3.3	0.038	287.5 ± 3.9	4.9E-04	—	—	—	—	124.4 ± 3.5	0.051	45.3 ± 1.0	1.0E-05	11.8 ± 0.8	0.001	1.16 ± 0.08	0.650
1pW10	12-28	—	—	—	—	220.3 ± 2.8	0.011	238.4 ± 4.3	0.830	—	—	227.8 ± 3.1	8.2E-09	235.3 ± 8.7	2.1E-04	—	—	—	—	—	—	—	—	64.3 ± 1.3	6.5E-09	—	—	—	—
1pW9	69-111	—	—	—	—	212.9 ± 1.5	0.781	228.2 ± 2.4	0.001	—	—	266.7 ± 1.7	1.1E-09	277.1 ± 3.6	0.132	—	—	—	—	—	—	—	—	59.9 ± 1.1	5.1E-06	—	—	—	—
1pW11	37-44	—	—	—	—	203.2 ± 2.1	2.0E-04	228.1 ± 3.2	0.012	239.6 ± 3.2	0.801	264.9 ± 1.9	1.1E-06	289.8 ± 3.7	0.127	303.1 ± 4.4	0.679	—	—	—	—	—	—	72.6 ± 1.5	1.7E-17	—	—	—	—

Hw, heart weight; Hw/Bw, heart weight divided by body weight; BP, blood pressure; T.adip, peri-testis fat pad; P.adip, retroperitoneal fat pad; FPG, fasting plasma glucose; T.chol, total cholesterol; TG, triglycerides; NEFA, non-esterified fatty acid.

Table S9B. Phenotype comparison in female rats between progenitor and RNO1 congenic strains involving SHRSP and WKY

Strain	*n*	Hw (g)		Hw/Bw (‰)		12wk BP (mmHg)		16wk BP (mmHg)		20wk BP (mmHg)		12wk Bw (g)		16wk Bw (g)		20wk Bw (g)		T.adip./BW (‰)		P.adip./BW (‰)		FPG		T.chol		TG		NEFA	
		mean ± SE	P, vs SHRSP	mean ± SE	P, vs SHRSP	mean ± SE	P, vs SHRSP	mean ± SE	P, vs SHRSP	mean ± SE	P, vs SHRSP	mean ± SE	P, vs SHRSP	mean ± SE	P, vs SHRSP	mean ± SE	P, vs SHRSP	mean ± SE	P, vs SHRSP	mean ± SE	P, vs SHRSP	mean ± SE	P, vs SHRSP	mean ± SE	P, vs SHRSP	mean ± SE	P, vs SHRSP	mean ± SE	P, vs SHRSP
SHRSP	35-195	0.72 ± 0.01	—	3.86 ± 0.02	—	176.3 ± 0.8	—	191.3 ± 1.1	—	198.7 ± 1.2	—	170.7 ± 0.8	—	189.4 ± 0.9	—	200.3 ± 1.1	—			6.92 ± 0.26	—	129.5 ± 1.9	—	69.7 ± 1.0	—	15.7 ± 0.6	—	0.88 ± 0.03	—
WKY	10-15	0.62 ± 0.01	—	2.66 ± 0.03	—	116.0 ± 1.8	—	118.8 ± 2.3	—	124.9 ± 3.5	—	197.6 ± 1.4	—	227.2 ± 2.2	—	239.8 ± 1.8	—			11.43 ± 0.40	—	108.9 ± 4.0	—	89.9 ± 3.1	—	15.3 ± 0.6	—	1.18 ± 0.08	—
1pW	32-125	0.72 ± 0.01	0.491	3.66 ± 0.02	3.4E-13	167.1 ± 1.1	3.8E-11	174.9 ± 1.0	3.7E-23	173.6 ± 1.3	2.5E-30	179.3 ± 0.8	9.4E-13	198.7 ± 1.0	8.6E-12	211.5 ± 1.3	1.2E-09			6.41 ± 0.14	0.084	117.8 ± 2.7	5.7E-04	78.0 ± 1.2	1.3E-06	11.2 ± 0.8	1.5E-05	1.25 ± 0.06	8.0E-07
1pWC	24-110	—	—	—	—	154.1 ± 0.9	9.4E-49	158.0 ± 1.2	2.6E-46	161.4 ± 1.4	3.7E-40	168.6 ± 1.0	0.103	188.1 ± 1.3	0.411	202.9 ± 1.6	0.170			—	—	127.2 ± 2.3	0.443	62.4 ± 1.6	3.3E-04	12.6 ± 0.7	0.001	1.09 ± 0.04	6.9E-05
1pW2	30-122	—	—	—	—	164.0 ± 1.1	1.7E-16	174.4 ± 1.4	1.2E-16	179.3 ± 1.4	1.3E-19	167.2 ± 1.3	0.023	183.6 ± 1.6	0.002	196.7 ± 1.6	0.071			—	—	125.0 ± 2.5	0.157	60.7 ± 1.1	8.4E-08	12.6 ± 1.1	0.018	0.80 ± 0.05	0.248
1pW5-2	30-88	—	—	—	—	168.0 ± 1.2	1.9E-08	172.8 ± 1.8	5.4E-14	177.7 ± 1.7	3.0E-17	175.2 ± 1.1	8.9E-04	190.6 ± 1.4	0.503	200.0 ± 1.5	0.870			—	—	134.1 ± 2.4	0.134	66.2 ± 1.5	0.058	10.9 ± 0.6	2.8E-07	0.88 ± 0.06	0.9835
1pW4-1	29-98	0.74 ± 0.01	0.225	4.02 ± 0.04	2.9E-04	171.5 ± 1.3	0.002	183.0 ± 1.4	5.7E-06	185.3 ± 1.4	1.8E-11	171.0 ± 1.0	0.811	185.3 ± 1.0	0.002	195.0 ± 0.4	0.002			6.00 ± 0.26	0.015	120.8 ± 3.6	0.037	66.9 ± 2.7	0.342	11.2 ± 0.5	3.6E-08	0.87 ± 0.05	0.967
1pW4-2	27-72	—	—	—	—	172.9 ± 1.3	0.023	179.0 ± 1.3	9.2E-11	190.4 ± 1.5	4.6E-05	174.4 ± 6.5	0.011	191.0 ± 1.4	0.351	200.9 ± 1.4	0.719			—	—	128.0 ± 2.2	0.608	67.2 ± 2.2	0.303	13.3 ± 0.7	0.012	0.85 ± 0.03	0.624
1pW4-3	42-67	0.75 ± 0.01	0.038	4.19 ± 0.05	2.8E-06	172.9 ± 1.4	0.018	178.9 ± 0.5	2.1E-09	194.0 ± 2.5	0.0617	168.1 ± 1.7	0.071	182.1 ± 1.3	7.7E-06	192.6 ± 2.4	1.2E-04			5.18 ± 0.21	2.5E-06	155.8 ± 4.5	6.1E-11	—	—	—	—	—	—
1pW4-4	61-83	0.73 ± 0.01	0.567	4.13 ± 0.04	3.8E-06	171.2 ± 1.4	0.002	180.0 ± 1.7	2.2E-07	187.7 ± 1.8	1.9E-06	171.4 ± 1.2	0.634	184.1 ± 1.3	0.001	196.7 ± 1.4	0.034			5.38 ± 0.22	3.5E-05	147.2 ± 2.4	7.4E-09	—	—	—	—	—	—
1pW6-2	27-65	—	—	—	—	165.9 ± 1.6	1.5E-07	172.0 ± 1.5	2.0E-18	176.8 ± 1.8	6.6E-17	171.4 ± 1.6	0.726	187.4 ± 1.8	0.325	194.9 ± 2.1	0.025			—	—	130.6 ± 3.1	0.776	56.3 ± 1.2	3.5E-12	10.5 ± 0.9	6.3E-06	1.08 ± 0.05	7.0E-04
1pW6-1	27-85	0.68 ± 0.01	9.1E-04	3.88 ± 0.03	0.680	167.5 ± 1.4	3.3E-07	172.4 ± 1.2	5.1E-22	180.5 ± 1.5	1.8E-16	172.7 ± 1.0	0.113	185.1 ± 1.1	0.002	196.9 ± 1.5	0.073			4.87 ± 0.21	6.8E-07	133.7 ± 3.3	0.271	58.8 ± 1.5	1.5E-07	10.7 ± 0.4	1.2E-09	1.05 ± 0.05	0.0024
1pW3-2	26-79	—	—	—	—	157.5 ± 1.3	7.4E-25	157.6 ± 1.5	8.9E-35	165.9 ± 1.5	7.7E-33	168.0 ± 1.1	0.045	183.7 ± 1.3	2.7E-04	196.0 ± 1.4	0.016			—	—	132.4 ± 3.9	0.508	57.8 ± 1.7	2.4E-07	10.7 ± 0.5	1.2E-08	1.08 ± 0.05	7.8E-04
1pW3-1	24-116	—	—	—	—	170.7 ± 1.1	4.2E-05	176.5 ± 1.8	9.8E-09	184.2 ± 2.3	3.9E-07	174.4 ± 1.1	0.009	189.9 ± 1.4	0.804	203.3 ± 2.2	0.225			—	—	124.1 ± 2.3	0.074	55.4 ± 2.6	1.4E-05	14.1 ± 1.0	0.150	0.87 ± 0.05	0.968
1pW8	25-68	—	—	—	—	174.5 ± 1.2	0.199	180.6 ± 1.8	2.4E-06	188.2 ± 1.9	1.7E-05	167.8 ± 1.5	0.082	182.9 ± 1.8	0.002	196.0 ± 1.8	0.051			—	—	123.9 ± 4.2	0.220	60.3 ± 1.7	2.1E-05	12.0 ± 0.4	1.7E-06	0.86 ± 0.03	0.7409
1pW10		—	—	—	—	—	—	—	—	—	—	—	—	—	—	—	—			—	—	—	—	—	—	—	—	—	—
1pW9	61-90	—	—	—	—	179.9 ± 1.3	0.022	189.6 ± 1.4	0.479	—	—	180.1 ± 1.1	1.8E-08	196.7 ± 1.6	4.1E-04	—	—			—	—	—	—	88.1 ± 2.3	1.0E-07	—	—	—	—
1pW11	38-47	—	—	—	—	173.5 ± 1.4	0.093	183.7 ± 2.0	0.003	195.9 ± 2.1	0.363	170.5 ± 2.2	0.474	186.5 ± 3.2	0.323	192.3 ± 3.9	0.036			—	—	—	—	81.5 ± 2.6	3.0E-04	—	—	—	—

Hw, heart weight; Hw/Bw, heart weight divided by body weight; BP, blood pressure; T.adip, peri-testis fat pad; P.adip, retroperitoneal fat pad; FPG, fasting plasma glucose; T.chol, total cholesterol; TG, triglycerides; NEFA, non-esterified fatty acid.
For female rats of 1pW10, phenotype characterization was not done, since the rats could not be maintained in the facility due to their high incidence of spontaneous stroke.

Table S9C. Pairwise phenotype comparison (in male and female rats) between RNO1 congenic strains involving SHRSP and WKY for sequential analysis

A pair of congenic strains	Hw (g)		Hw/Bw (‰)		12wk BP (mmHg)		16wk BP (mmHg)		20wk BP (mmHg)		12wk Bw (g)		16wk Bw (g)		20wk Bw (g)		T.adip./BW (‰)		P.adip./BW (‰)		FPG		T.chol		TG		NEFA	
	Male, P	Female, P	Male, P	Female, P	Male, P	Female, P	Male, P	Female, P	Male, P	Female, P	Male, P	Female, P	Male, P	Female, P	Male, P	Female, P	Male, P	Female, P	Male, P	Female, P	Male, P	Female, P	Male, P	Female, P	Male, P	Female, P	Male, P	Female, P
SHRSP_1pW	1.1E-04	0.491	4.4E-12	3.4E-13	7.3E-24	3.8E-11	3.3E-34	3.7E-23	4.7E-23	2.5E-30	2.7E-16	9.4E-13	1.1E-15	8.6E-12	6.2E-20	1.2E-09	2.8E-17	—	4.0E-04	0.084	8.5E-07	5.7E-04	1.9E-05	1.3E-06	1.9E-11	1.5E-05	0.048	8.0E-07
1pW_1pWC	—	—	—	—	4.5E-14	1.3E-17	4.8E-24	4.8E-20	9.8E-06	1.6E-05	1.2E-16	4.5E-14	6.1E-07	1.5E-09	1.5E-18	4.9E-05	—	—	—	—	0.314	0.010	5.7E-12	4.1E-10	0.025	0.170	0.697	0.033
1pWC_1pW2	—	—	—	—	3.2E-07	1.2E-10	2.3E-07	9.3E-15	6.0E-07	3.6E-11	0.930	0.414	4.1E-04	0.030	3.2E-04	0.007	—	—	—	—	0.568	0.527	7.8E-06	0.392	0.119	0.979	0.759	9.2E-05
1pW2_1pW5-2	—	—	—	—	0.049	0.015	0.418	0.482	0.660	0.458	0.008	3.9E-06	0.005	0.001	0.008	0.143	—	—	—	—	2.2E-12	0.011	0.689	0.005	0.956	0.185	0.246	0.366
1pW5-2_1pW4-1	—	—	—	—	0.002	0.040	3.1E-06	1.8E-05	0.002	7.2E-04	0.253	0.004	0.253	0.003	0.035	0.012	—	—	—	—	0.009	0.003	0.024	0.820	0.567	0.774	0.949	0.961
1pW4-1_1pW4-2	—	—	—	—	0.732	0.462	0.027	0.039	0.238	0.017	0.441	0.031	0.206	0.002	0.016	0.002	—	—	—	—	0.647	0.092	3.1E-04	0.939	0.009	0.021	0.407	0.748
1pW4-1_1pW4-3	0.343	0.268	2.9E-07	0.012	0.649	0.429	0.054	0.046	0.087	0.001	0.068	0.062	0.301	0.052	0.003	0.226	0.043	—	4.1E-04	0.020	8.3E-04	2.0E-10	—	—	—	—	—	—
1pW4-3_1pW4-4	0.170	0.245	0.197	0.353	0.007	0.339	0.001	0.638	0.315	0.027	0.244	0.047	0.856	0.052	0.115	0.043	0.604	—	0.974	0.523	0.018	0.021	—	—	—	—	—	—
1pW6-2_1pW6-1	—	—	—	—	0.183	0.489	0.070	0.831	0.121	0.115	0.010	0.456	0.300	0.273	0.815	0.435	—	—	—	—	0.398	0.486	0.183	0.196	0.008	0.800	0.843	0.598
1pW3-2_1pW3-1	—	—	—	—	1.2E-06	2.0E-13	9.0E-15	0.0000	9.3E-05	3.6E-09	0.789	6.9E-05	0.329	0.003	0.108	0.006	—	—	—	—	0.473	0.074	0.490	0.434	0.262	0.005	0.371	0.006
1pW3-1_1pW8	—	—	—	—	3.0E-07	0.020	0.002	0.133	5.6E-07	0.182	0.057	5.2E-04	0.063	0.005	8.1E-04	0.013	—	—	—	—	0.573	0.956	1.5E-05	0.122	0.063	0.062	0.006	0.847
1pW10_1pW9	—	—	—	—	0.024	—	0.051	—	—	—	1.8E-14	—	5.6E-04	—	—	—	—	—	—	—	—	—	0.015	—	—	—	—	—
1pW9_1pW11	—	—	—	—	3.0E-04	0.001	0.989	0.018	—	—	0.501	1.8E-04	0.015	0.007	—	—	—	—	—	—	—	—	3.1E-09	0.064	—	—	—	—

Table S10A. Phenotype comparison in male rats between progenitor and RNO3 congenic strains involving SHR and WKY

Strain	*n*	Hw (g)		Hw/Bw (‰)		12wk BP (mmHg)		16wk BP (mmHg)		16wk Bw (g)		T.adip./BW (‰)		P.adip./BW (‰)		FPG		T.chol		TG		NEFA	
		mean ± SE	P, vs SHR	mean ± SE	P, vs SHR	mean ± SE	P, vs SHR	mean ± SE	P, vs SHR	mean ± SE	P, vs SHR	mean ± SE	P, vs SHR	mean ± SE	P, vs SHR	mean ± SE	P, vs SHR	mean ± SE	P, vs SHR	mean ± SE	P, vs SHR	mean ± SE	P, vs SHR
SHR	23-74	1.11 ± 0.01	—	3.42 ± 0.04	—	186.1 ± 1.6	—	197.6 ± 2.0	—	322.1 ± 2.1	—	12.00 ± 0.33	—	9.74 ± 0.33	—	123.8 ± 3.5	—	59.9 ± 0.9	—	12.4 ± 0.5	—	0.98 ± 0.04	—
WKY	10-27	0.98 ± 0.01	—	2.61 ± 0.03	—	130.3 ± 2.0	—	123.4 ± 2.3	—	366.6 ± 4.6	—	13.25 ± 0.31	—	10.72 ± 0.34	—	108.3 ± 3.3	—	83.1 ± 3.1	—	12.5 ± 1.3	—	1.30 ± 0.06	—
3SW1	51-54	1.08 ± 0.01	0.039	3.19 ± 0.01	3.4E-06	179.2 ± 1.2	7.4E-04	187.6 ± 1.2	3.5E-05	337.5 ± 2.2	1.7E-06	8.92 ± 0.14	1.2E-09	8.02 ± 0.18	6.4E-05	120.4 ± 1.9	0.392	54.4 ± 0.9	2.1E-05	5.7 ± 0.3	1.5E-17	1.33 ± 0.06	9.3E-06
3SW4	85-120	1.10 ± 0.02	0.934	3.40 ± 0.05	0.744	170.3 ± 1.1	7.4E-13	176.5 ± 1.2	1.8E-14	327.3 ± 1.8	0.067	10.05 ± 0.12	5.9E-06	9.99 ± 0.18	0.526	124.6 ± 2.2	0.841	58.0 ± 1.0	0.149	9.3 ± 0.5	8.2E-05	1.35 ± 0.04	4.0E-08
3SW	53-67	1.03 ± 0.01	3.0E-06	3.27 ± 0.02	0.002	176.3 ± 1.1	2.5E-06	183.5 ± 1.3	4.0E-08	316.7 ± 2.0	0.066	9.81 ± 0.18	1.2E-06	9.89 ± 0.18	0.702	115.1 ± 2.4	0.044	47.3 ± 1.1	6.4E-14	6.8 ± 0.2	2.0E-14	1.05 ± 0.05	0.312
3SW5	36-39	1.01 ± 0.01	6.7E-07	3.42 ± 0.03	0.989	166.9 ± 2.5	9.9E-09	169.5 ± 2.2	3.4E-15	296.6 ± 3.0	9.5E-10	11.95 ± 0.18	0.896	10.54 ± 0.25	0.064	144.2 ± 2.6	8.7E-06	52.0 ± 0.8	2.7E-09	7.5 ± 0.5	8.2E-10	1.24 ± 0.05	1.0E-04
3SW3	30-33	1.08 ± 0.02	0.275	3.39 ± 0.05	0.642	175.2 ± 1.6	4.9E-06	177.4 ± 1.7	3.0E-11	322.0 ± 2.8	0.970	10.10 ± 0.18	1.4E-05	9.03 ± 0.22	0.083	143.5 ± 2.9	3.9E-05	56.4 ± 1.1	0.012	5.8 ± 0.3	1.2E-16	0.97 ± 0.04	0.756
3SW6	35-45	1.14 ± 0.01	0.038	3.54 ± 0.02	0.005	172.7 ± 1.9	6.2E-07	184.2 ± 1.9	4.0E-06	322.6 ± 3.3	0.911	11.17 ± 0.17	0.033	9.28 ± 0.28	0.294	134.6 ± 4.3	0.053	64.2 ± 1.0	0.002	12.5 ± 0.7	0.875	1.43 ± 0.04	2.3E-11
3SW2	29-30	1.07 ± 0.02	0.109	3.39 ± 0.05	0.701	175.9 ± 2.3	5.9E-04	183.2 ± 1.7	3.1E-07	315.5 ± 4.0	0.153	11.43 ± 0.23	0.161	8.97 ± 0.36	0.124	126.4 ± 3.4	0.589	54.7 ± 1.0	1.8E-04	8.7 ± 0.7	1.9E-04	1.33 ± 0.05	5.6E-07

Hw, heart weight; Hw/Bw, heart weight divided by body weight; BP, blood pressure; T.adip, peri-testis fat pad; P.adip, retroperitoneal fat pad; FPG, fasting plasma glucose; T.chol, total cholesterol; TG, triglycerides; NEFA, non-esterified fatty acid.

Table S10B. Phenotype comparison in female rats between progenitor and RNO3 congenic strains involving SHR and WKY

Strain	*n*	Hw (g)		Hw/Bw (‰)		12wk BP (mmHg)		16wk BP (mmHg)		16wk Bw (g)		P.adip./BW (‰)		FPG		T.chol		TG		NEFA	
		mean ± SE	P, vs SHR	mean ± SE	P, vs SHR	mean ± SE	P, vs SHR	mean ± SE	P, vs SHR	mean ± SE	P, vs SHR	mean ± SE	P, vs SHR	mean ± SE	P, vs SHR	mean ± SE	P, vs SHR	mean ± SE	P, vs SHR	mean ± SE	P, vs SHR
SHR	26-85	0.74 ± 0.01	—	3.83 ± 0.05	—	161.9 ± 1.3	—	173.6 ± 1.6	—	190.9 ± 1.2	—	7.29 ± 0.19	—	120.5 ± 3.2	—	69.0 ± 1.1	—	10.8 ± 0.3	—	1.13 ± 0.04	—
WKY	10-15	0.62 ± 0.01	—	2.66 ± 0.03	—	116.0 ± 1.8	—	118.8 ± 2.3	—	227.2 ± 2.2	—	11.43 ± 0.4	—	108.9 ± 4.0	—	89.9 ± 3.1	—	15.3 ± 0.6	—	1.18 ± 0.08	—
3SW1	39-45	0.74 ± 0.01	0.894	3.53 ± 0.02	3.1E-06	156.5 ± 1.0	0.001	161.5 ± 1.4	1.6E-07	210.2 ± 1.4	1.3E-17	7.20 ± 0.16	0.729	129.4 ± 2.8	0.038	84.7 ± 1.2	4.0E-15	10.2 ± 0.4	0.249	1.40 ± 0.04	1.5E-05
3SW4	79-125	0.73 ± 0.01	0.187	3.70 ± 0.03	0.036	153.1 ± 1.0	3.5E-07	160.7 ± 1.3	3.0E-09	202.5 ± 3.8	5.7E-05	8.20 ± 0.12	1.8E-04	124.2 ± 2.1	0.331	77.0 ± 0.9	3.8E-07	11.2 ± 0.6	0.508	1.24 ± 0.04	0.054
3SW	53-78	0.68 ± 0.005	1.2E-07	3.58 ± 0.02	5.4E-05	154.2 ± 1.1	1.1E-05	155.7 ± 1.4	7.1E-14	189.9 ± 1.3	0.564	8.86 ± 0.18	8.9E-08	120.9 ± 2.9	0.937	65.7 ± 1.1	0.039	9.1 ± 0.2	9.6E-06	0.99 ± 0.04	0.010
3SW5	33-37	0.71 ± 0.01	0.002	3.92 ± 0.03	0.119	146.1 ± 1.7	1.7E-10	150.4 ± 1.9	4.9E-14	180.2 ± 1.7	2.4E-06	7.88 ± 0.16	0.019	151.1 ± 2.2	6.2E-12	71.3 ± 1.1	0.156	7.7 ± 0.5	1.3E-06	1.19 ± 0.04	0.309
3SW3	45-51	0.74 ± 0.01	0.779	3.78 ± 0.03	0.484	156.0 ± 1.7	0.007	159.9 ± 1.2	3.7E-10	195.9 ± 1.4	0.008	7.23 ± 0.15	0.810	134.2 ± 2.0	4.2E-04	74.6 ± 0.8	1.1E-04	6.5 ± 0.4	2.0E-13	1.10 ± 0.03	0.566
3SW6	45-59	0.75 ± 0.01	0.759	3.98 ± 0.02	0.009	152.0 ± 1.6	3.7E-06	158.1 ± 1.6	4.4E-10	188.2 ± 1.5	0.148	7.38 ± 0.18	0.739	149.1 ± 2.5	1.6E-10	78.3 ± 1.7	2.0E-05	10.9 ± 0.6	0.876	1.52 ± 0.04	8.0E-10
3SW2	41-44	0.72 ± 0.01	0.025	3.82 ± 0.02	0.964	155.3 ± 1.4	9.6E-04	161.4 ± 1.3	6.0E-08	187.8 ± 1.5	0.106	7.31 ± 0.14	0.931	139.9 ± 2.4	4.5E-06	75.7 ± 1.0	2.7E-05	8.5 ± 0.6	0.001	1.17 ± 0.04	0.468

Hw, heart weight; Hw/Bw, heart weight divided by body weight; BP, blood pressure; T.adip, peri-testis fat pad; P.adip, retroperitoneal fat pad; FPG, fasting plasma glucose; T.chol, total cholesterol; TG, triglycerides; NEFA, non-esterified fatty acid.

Table S10C. Pairwise phenotype comparison (in male and female rats) between RNO3 congenic strains involving SHR and WKY for sequential analysis

A pair of congenic strains	Hw (g)		Hw/Bw (‰)		12wk BP (mmHg)		16wk BP (mmHg)		16wk Bw (g)		T.adip./BW (‰)		P.adip./BW (‰)		FPG		T.chol		TG		NEFA	
	Male, P	Female, P	Male, P	Female, P	Male, P	Female, P	Male, P	Female, P	Male, P	Female, P	Male, P	Female, P	Male, P	Female, P	Male, P	Female, P	Male, P	Female, P	Male, P	Female, P	Male, P	Female, P
3SW1_3SW4	0.125	0.150	9.8E-05	8.2E-06	1.5E-07	0.016	6.8E-10	0.518	5.2E-04	0.009	2.1E-08	N/A	4.5E-12	3.9E-06	0.146	0.137	0.007	1.7E-06	3.8E-07	0.151	0.736	0.007
3SW4_3SW	1.1E-04	5.5E-07	0.022	0.001	1.8E-04	0.472	7.9E-05	0.008	1.6E-04	1.8E-05	0.275	N/A	0.710	0.003	0.005	0.349	2.5E-10	5.6E-12	1.4E-04	9.3E-04	1.1E-05	1.6E-05
3SW_3SW5	0.174	0.009	4.2E-04	5.2E-12	1.0E-03	1.6E-04	4.6E-07	0.029	4.0E-07	2.3E-05	9.1E-13	N/A	0.042	9.0E-05	3.3E-12	1.2E-12	8.6E-04	5.8E-04	0.179	0.013	0.005	5.0E-04
3SW5_3SW3	0.001	7.3E-04	0.637	0.004	0.0056	8.1E-05	0.0056	7.8E-05	4.3E-08	9.8E-10	7.0E-10	N/A	3.0E-05	0.004	0.855	2.4E-07	0.002	0.016	0.004	0.054	1.8E-05	0.085
3SW4_3SW6	0.069	0.077	0.007	2.2E-10	0.272	0.716	0.0009	0.939	0.215	2.6E-06	1.5E-06	N/A	0.038	2.7E-04	0.041	7.3E-12	3.9E-05	0.475	4.7E-04	0.694	0.181	4.1E-06

Hw, heart weight; Hw/Bw, heart weight divided by body weight; BP, blood pressure; T.adip, peri-testis fat pad; P.adip, retroperitoneal fat pad; FPG, fasting plasma glucose; T.chol, total cholesterol; TG, triglycerides; NEFA, non-esterified fatty acid.

Table S10D. Phenotype comparison in male rats between progenitor and RNO3 congenic strains involving SHRSP and WKY

Strain	*n*	Hw (g)		Hw/Bw (‰)		12wk BP (mmHg)		16wk BP (mmHg)		16wk Bw (g)		T.adip./BW (‰)		P.adip./BW (‰)		FPG		T.chol		TG		NEFA	
		mean ± SE	P, vs SHRSP	mean ± SE	P, vs SHRSP	mean ± SE	P, vs SHRSP	mean ± SE	P, vs SHRSP	mean ± SE	P, vs SHRSP	mean ± SE	P, vs SHRSP	mean ± SE	P, vs SHRSP	mean ± SE	P, vs SHRSP	mean ± SE	P, vs SHRSP	mean ± SE	P, vs SHRSP	mean ± SE	P, vs SHRSP
SHRSP	38-173	1.07 ± 0.01	—	3.82 ± 0.03	—	211.1 ± 1.0	—	236.9 ± 1.5	—	283.7 ± 1.7	—	11.05 ± 0.22	—	8.22 ± 0.23	—	133.9 ± 3.3	—	52.2 ± 1.0	—	15.7 ± 0.8	—	1.12 ± 0.06	—
WKY	10-27	0.98 ± 0.01	—	2.61 ± 0.03	—	130.3 ± 2.0	—	123.4 ± 2.3	—	366.6 ± 4.6	—	13.25 ± 0.31	—	10.72 ± 0.34	—	108.3 ± 3.3	—	83.1 ± 3.1	—	12.5 ± 1.3	—	1.30 ± 0.06	—
3pW1	35-36	1.02 ± 0.01	0.001	3.44 ± 0.02	2.5E-15	198.6 ± 2.1	2.1E-06	205.3 ± 2.0	3.1E-20	295.6 ± 4.2	0.009	9.26 ± 0.17	2.5E-08	7.53 ± 0.20	0.027	132.7 ± 2.7	0.787	49.2 ± 0.5	0.010	10.0 ± 0.5	6.0E-08	1.33 ± 0.05	0.011
3pW4	34-37	1.00 ± 0.01	1.2E-08	3.55 ± 0.03	6.5E-09	201.2 ± 1.8	1.0E-05	211.7 ± 2.1	1.2E-14	280.8 ± 1.6	0.296	9.22 ± 0.22	1.4E-07	7.88 ± 0.17	0.229	132.1 ± 2.4	0.656	40.5 ± 0.7	2.0E-13	8.8 ± 0.5	5.6E-10	1.05 ± 0.06	0.453
3pW	40-67	1.01 ± 0.01	4.3E-04	3.50 ± 0.02	6.0E-12	192.7 ± 1.6	1.3E-16	200.7 ± 1.7	1.7E-31	285.1 ± 2.4	0.510	9.45 ± 0.20	1.3E-06	9.42 ± 0.17	5.9E-05	127.1 ± 2.5	0.103	39.8 ± 0.6	6.8E-16	8.6 ± 1.2	1.4E-04	0.98 ± 0.04	0.066
3pW5	70-80	1.07 ± 0.01	0.966	3.72 ± 0.02	0.008	188.0 ± 1.7	3.9E-22	203.5 ± 1.7	5.0E-32	287.5 ± 2.0	0.103	11.08 ± 0.13	0.903	9.75 ± 0.19	1.7E-06	139.7 ± 1.8	0.121	48.6 ± 0.6	2.0E-13	7.6 ± 0.7	3.1E-11	1.10 ± 0.04	0.794
3pW3	33-39	0.97 ± 0.01	8.6E-08	3.45 ± 0.03	4.5E-12	191.0 ± 2.0	1.1E-12	197.9 ± 2.4	1.5E-20	282.3 ± 2.8	0.790	10.25 ± 0.17	0.006	9.27 ± 0.24	0.002	132.7 ± 2.0	0.763	49.0 ± 0.6	0.009	5.8 ± 0.4	2.5E-16	0.86 ± 0.03	3.0E-04
3pW2	83-89	1.06 ± 0.01	0.477	3.72 ± 0.02	0.011	201.0 ± 1.5	4.4E-08	215.5 ± 1.8	7.7E-17	285.9 ± 1.6	0.242	12.02 ± 0.17	8.3E-04	9.08 ± 0.16	0.003	131.7 ± 2.2	0.588	50.7 ± 0.5	0.190	11.1 ± 0.6	1.6E-05	1.27 ± 0.05	0.066

Hw, heart weight; Hw/Bw, heart weight divided by body weight; BP, blood pressure; T.adip, peri-testis fat pad; P.adip, retroperitoneal fat pad; FPG, fasting plasma glucose; T.chol, total cholesterol; TG, triglycerides; NEFA, non-esterified fatty acid.

Table S10E. Phenotype comparison in female rats between progenitor and RNO3 congenic strains involving SHRSP and WKY

Strain	*n*	Hw (g)		Hw/Bw (‰)		12wk BP (mmHg)		16wk BP (mmHg)		16wk Bw (g)		P.adip./BW (‰)		FPG		T.chol		TG		NEFA	
		mean ± SE	P, vs SHRSP	mean ± SE	P, vs SHRSP	mean ± SE	P, vs SHRSP	mean ± SE	P, vs SHRSP	mean ± SE	P, vs SHRSP	mean ± SE	P, vs SHRSP	mean ± SE	P, vs SHRSP	mean ± SE	P, vs SHRSP	mean ± SE	P, vs SHRSP	mean ± SE	P, vs SHRSP
SHRSP	35-195	0.72 ± 0.01	—	3.86 ± 0.02	—	176.3 ± 0.8	—	190.8 ± 1.1	—	189.4 ± 0.8	—	6.92 ± 0.26	—	129.5 ± 1.9	—	69.7 ± 1.0	—	15.7 ± 0.6	—	0.88 ± 0.03	—
WKY	10-15	0.62 ± 0.01	—	2.66 ± 0.03	—	116.0 ± 1.8	—	118.8 ± 2.3	—	227.2 ± 2.2	—	11.43 ± 0.4	—	108.9 ± 4.0	—	89.9 ± 3.1	—	15.3 ± 0.6	—	1.18 ± 0.08	—
3pW1	39-41	0.73 ± 0.01	0.496	3.65 ± 0.01	3.2E-14	176.5 ± 2.1	0.941	178.5 ± 1.9	3.0E-07	199.8 ± 1.9	4.7E-06	6.81 ± 0.15	0.703	135.2 ± 1.7	0.027	70.5 ± 0.9	0.556	11.9 ± 0.5	2.3E-06	0.86 ± 0.04	0.762
3pW4	43-54	0.71 ± 0.01	0.426	3.79 ± 0.03	0.014	169.0 ± 1.9	5.7E-04	181.0 ± 1.8	1.3E-05	189.0 ± 1.6	0.848	7.85 ± 0.15	0.003	139.1 ± 1.8	3.1E-04	51.2 ± 1.0	4.8E-18	10.4 ± 0.4	7.1E-11	0.97 ± 0.04	0.063
3pW	34-64	0.73 ± 0.01	0.533	3.80 ± 0.03	0.120	166.3 ± 1.4	4.5E-09	167.5 ± 1.9	2.5E-17	187.3 ± 1.5	0.227	9.64 ± 0.17	2.6E-12	128.9 ± 2.5	0.841	63.3 ± 1.5	0.001	12.3 ± 0.6	8.9E-05	0.91 ± 0.05	0.604
3pW5	79	0.73 ± 0.00	0.625	3.93 ± 0.02	0.003	160.5 ± 1.6	1.1E-14	170.4 ± 1.1	9.3E-29	184.9 ± 1.0	7.2E-04	9.27 ± 0.16	1.1E-10	150.5 ± 2.2	1.2E-10	65.4 ± 1.3	0.008	9.5 ± 0.7	1.4E-09	0.94 ± 0.03	0.187
3pW3	28-35	0.67 ± 0.01	1.4E-04	3.61 ± 0.03	5.1E-10	166.2 ± 1.6	8.6E-07	169.0 ± 2.0	4.9E-13	185.4 ± 2.1	0.086	8.38 ± 0.21	5.1E-05	145.0 ± 2.5	6.9E-06	64.3 ± 1.5	0.003	10.9 ± 0.9	5.6E-05	0.85 ± 0.04	0.640
3pW2	69-72	0.74 ± 0.01	0.065	4.00 ± 0.02	4.4E-07	170.7 ± 1.7	0.003	179.4 ± 1.7	1.6E-07	185.6 ± 1.0	0.005	7.92 ± 0.13	0.001	142.9 ± 2.9	2.6E-04	65.4 ± 1.2	0.007	9.7 ± 0.4	7.9E-13	0.86 ± 0.03	0.764

Hw, heart weight; Hw/Bw, heart weight divided by body weight; BP, blood pressure; T.adip, peri-testis fat pad; P.adip, retroperitoneal fat pad; FPG, fasting plasma glucose; T.chol, total cholesterol; TG, triglycerides; NEFA, non-esterified fatty acid.

Table S10F. Pairwise phenotype comparison (in male and female rats) between RNO3 congenic strains involving SHRSP and WKY for sequential analysis

A pair of congenic strains	Hw (g)		Hw/Bw (‰)		12wk BP (mmHg)		16wk BP (mmHg)		16wk Bw (g)		T.adip./BW (‰)		P.adip./BW (‰)		FPG		T.chol		TG		NEFA	
	Male, P	Female, P	Male, P	Female, P	Male, P	Female, P	Male, P	Female, P	Male, P	Female, P	Male, P	Female, P	Male, P	Female, P	Male, P	Female, P	Male, P	Female, P	Male, P	Female, P	Male, P	Female, P
3pW1_3pW4	0.2287	0.125	6.1E-04	2.4E-05	0.345	0.010	0.033	0.329	1.9E-03	3.6E-05	0.877	N/A	0.193	3.7E-06	0.854	0.119	5.4E-12	2.0E-17	0.122	0.018	0.001	0.050
3pW4_3pW	0.5016	0.177	0.169	0.683	7.7E-04	0.239	1.5E-04	1.1E-06	0.131	0.431	0.436	N/A	8.1E-09	4.2E-11	0.154	0.001	0.442	2.7E-08	0.877	0.011	0.334	0.301
3pW_3pW5	4.2E-04	0.748	1.7E-11	0.001	0.046	0.006	0.253	0.183	0.450	0.176	3.1E-09	N/A	0.201	0.112	7.5E-05	4.4E-09	1.7E-16	0.295	0.143	0.003	0.058	0.629
3pW5_3pW3	7.1E-08	3.3E-06	6.1E-10	1.4E-13	0.251	0.015	0.060	0.528	0.139	0.811	2.1E-04	N/A	0.125	0.002	0.011	0.105	0.643	0.563	0.018	0.227	3.5E-05	0.124
3pW5_3pW2	0.4930	0.049	0.812	0.014	4.0E-08	1.8E-05	1.9E-06	2.1E-05	0.540	0.587	1.5E-05	N/A	0.009	1.0E-09	0.007	0.043	0.009	0.987	1.9E-04	0.755	0.016	0.103

Hw, heart weight; Hw/Bw, heart weight divided by body weight; BP, blood pressure; T.adip, peri-testis fat pad; P.adip, retroperitoneal fat pad; FPG, fasting plasma glucose; T.chol, total cholesterol; TG, triglycerides; NEFA, non-esterified fatty acid.

Table S10G. Phenotype comparison in male rats between progenitor and RNO4 congenic strains involving SHR and WKY

Strain	*n*	Hw (g)		Hw/Bw (‰)		12wk BP (mmHg)		16wk BP (mmHg)		16wk Bw (g)		T.adip./BW (‰)		P.adip./BW (‰)		FPG		T.chol		TG		NEFA	
		mean ± SE	P, vs SHR	mean ± SE	P, vs SHR	mean ± SE	P, vs SHR	mean ± SE	P, vs SHR	mean ± SE	P, vs SHR	mean ± SE	P, vs SHR	mean ± SE	P, vs SHR	mean ± SE	P, vs SHR	mean ± SE	P, vs SHR	mean ± SE	P, vs SHR	mean ± SE	P, vs SHR
SHR	23-74	1.11 ± 0.01	—	3.42 ± 0.04	—	186.1 ± 1.6	—	197.6 ± 2.0	—	322.1 ± 2.1	—	12.00 ± 0.33	—	9.74 ± 0.33	—	123.8 ± 3.5	—	59.9 ± 0.9	—	12.4 ± 0.5	—	0.98 ± 0.04	—
WKY	10-27	0.98 ± 0.01	—	2.61 ± 0.03	—	130.3 ± 2.0	—	123.4 ± 2.3	—	366.6 ± 4.6	—	13.25 ± 0.31	—	10.72 ± 0.34	—	108.3 ± 3.3	—	83.1 ± 3.1	—	12.5 ± 1.3	—	1.30 ± 0.06	—
4SW2	32-39	1.10 ± 0.01	0.620	3.50 ± 0.03	0.081	173.9 ± 1.7	1.E-06	178.8 ± 1.6	6.E-11	312.6 ± 2.9	0.010	10.60 ± 0.16	0.001	8.70 ± 0.25	0.016	129.8 ± 2.0	0.132	62.7 ± 0.8	0.026	9.6 ± 0.4	3.E-05	1.11 ± 0.03	0.022
4SW3	33-43	1.07 ± 0.01	0.021	3.48 ± 0.02	0.151	175.1 ± 1.6	4.E-06	180.2 ± 1.8	4.E-09	307.4 ± 2.8	6.E-05	10.77 ± 0.22	0.003	8.16 ± 0.22	3.E-04	120.0 ± 2.7	0.068	66.1 ± 0.9	5.E-06	11.1 ± 0.5	0.075	1.27 ± 0.03	1.E-06
4SW4	33-40	1.12 ± 0.01	0.526	3.53 ± 0.03	0.032	187.8 ± 1.8	0.480	203.3 ± 1.9	0.040	315.2 ± 2.8	0.053	10.82 ± 0.22	0.004	8.55 ± 0.22	0.005	115.9 ± 3.0	0.089	66.2 ± 0.8	1.E-06	11.9 ± 0.5	0.523	1.41 ± 0.03	1.E-11
4SW1	33-49	1.06 ± 0.01	0.011	3.33 ± 0.02	0.056	175.8 ± 1.8	5.E-05	183.9 ± 2.1	8.E-06	317.6 ± 3.2	0.241	10.71 ± 0.19	0.002	8.50 ± 0.17	0.002	136.5 ± 2.3	0.003	63.3 ± 0.9	0.010	11.3 ± 0.7	0.186	1.30 ± 0.04	9.E-07
4SW5	38-51	1.10 ± 0.02	0.703	3.47 ± 0.03	0.279	175.9 ± 1.8	6.E-05	188.5 ± 2.4	0.005	316.1 ± 3.5	0.145	11.57 ± 0.32	0.349	9.96 ± 0.38	0.665	134.9 ± 3.2	0.021	68.6 ± 1.0	5.E-09	13.9 ± 1.4	0.288	1.41 ± 0.05	4.E-09
4SW6	37-50	1.14 ± 0.02	0.118	3.65 ± 0.04	1.E-04	173.7 ± 1.8	1.E-06	180.3 ± 2.0	2.E-08	313.2 ± 3.3	0.025	10.31 ± 0.21	1.E-04	7.61 ± 0.18	2.E-06	133.3 ± 2.2	0.022	62.5 ± 1.2	0.089	10.8 ± 0.4	0.016	1.30 ± 0.04	1.E-06
4SW	34-43	0.99 ± 0.01	7.E-11	3.28 ± 0.02	0.002	182.9 ± 1.7	0.172	190.8 ± 1.4	0.006	303.4 ± 2.3	4.E-08	10.30 ± 0.24	2.E-04	8.77 ± 0.24	0.022	118.0 ± 2.9	0.207	54.0 ± 0.8	2.E-06	13.3 ± 0.8	0.295	1.16 ± 0.04	0.004

Hw, heart weight; Hw/Bw, heart weight divided by body weight; BP, blood pressure; T.adip, peri-testis fat pad; P.adip, retroperitoneal fat pad; FPG, fasting plasma glucose; T.chol, total cholesterol; TG, triglycerides; NEFA, non-esterified fatty acid.

Table S10H. Phenotype comparison in female rats between progenitor and RNO4 congenic strains involving SHR and WKY

Strain	*n*	Hw (g)		Hw/Bw (‰)		12wk BP (mmHg)		16wk BP (mmHg)		16wk Bw (g)		P.adip./BW (‰)		FPG		T.chol		TG		NEFA	
		mean ± SE	P, vs SHR	mean ± SE	P, vs SHR	mean ± SE	P, vs SHR	mean ± SE	P, vs SHR	mean ± SE	P, vs SHR	mean ± SE	P, vs SHR	mean ± SE	P, vs SHR	mean ± SE	P, vs SHR	mean ± SE	P, vs SHR	mean ± SE	P, vs SHR
SHR	26-85	0.74 ± 0.01	—	3.83 ± 0.05	—	161.9 ± 1.3	—	173.6 ± 1.6	—	190.9 ± 1.2	—	7.29 ± 0.19	—	120.5 ± 3.2	—	69.0 ± 1.1	—	10.8 ± 0.3	—	1.13 ± 0.04	—
WKY	10-15	0.62 ± 0.01	—	2.66 ± 0.03	—	116.0 ± 1.8	—	118.8 ± 2.3	—	227.2 ± 2.2	—	11.43 ± 0.4	—	108.9 ± 4.0	—	89.9 ± 3.1	—	15.3 ± 0.6	—	1.18 ± 0.08	—
4SW2	32-38	0.76 ± 0.01	0.152	3.91 ± 0.03	0.163	152.8 ± 1.4	1.E-05	157.7 ± 1.2	4.E-12	194.5 ± 1.4	0.054	6.56 ± 0.15	0.004	122.9 ± 3.7	0.626	85.0 ± 1.2	1.E-14	9.2 ± 0.4	0.002	1.43 ± 0.04	3.E-07
4SW3	30-38	0.72 ± 0.01	0.054	3.92 ± 0.02	0.076	155.7 ± 1.6	0.004	152.2 ± 1.5	3.E-15	184.1 ± 1.5	9.E-04	6.50 ± 0.18	0.003	131.1 ± 2.9	0.017	78.8 ± 1.4	2.E-06	9.7 ± 0.5	0.074	1.45 ± 0.04	5.E-07
4SW4	32-38	0.73 ± 0.01	0.419	3.88 ± 0.02	0.341	155.4 ± 1.6	0.002	168.6 ± 1.8	0.041	189.1 ± 1.6	0.348	6.81 ± 0.21	0.090	117.2 ± 4.0	0.519	79.4 ± 1.3	3.E-08	9.8 ± 0.6	0.124	1.53 ± 0.05	5.E-09
4SW1	32-38	0.70 ± 0.01	3.E-04	3.72 ± 0.03	0.083	156.0 ± 1.8	0.011	163.2 ± 1.7	3.E-05	188.2 ± 2.0	0.262	6.75 ± 0.18	0.045	131.9 ± 1.8	0.002	77.2 ± 1.4	3.E-05	8.9 ± 0.4	0.001	1.34 ± 0.04	5.E-04
4SW5	37-57	0.76 ± 0.01	0.187	3.92 ± 0.04	0.141	154.4 ± 1.7	8.E-04	161.1 ± 1.8	1.E-06	192.7 ± 2.1	0.459	8.28 ± 0.45	0.045	135.4 ± 3.2	6.E-04	84.2 ± 1.3	8.E-14	13.0 ± 1.2	0.083	1.60 ± 0.04	3.E-13
4SW6	34-49	0.75 ± 0.01	0.460	4.03 ± 0.03	6.E-04	156.9 ± 1.2	0.005	159.1 ± 1.5	3.E-09	186.4 ± 1.4	0.018	6.69 ± 0.15	0.018	134.2 ± 2.6	0.001	85.4 ± 1.5	8.E-13	10.4 ± 0.5	0.487	1.33 ± 0.05	0.002
4SW	26-41	0.70 ± 0.01	0.001	3.69 ± 0.02	0.015	158.9 ± 1.6	0.157	166.7 ± 1.5	0.002	189.8 ± 2.1	0.634	7.28 ± 0.21	0.969	118.7 ± 3.2	0.692	74.4 ± 1.6	0.008	11.3 ± 0.5	0.417	1.32 ± 0.06	0.009

Hw, heart weight; Hw/Bw, heart weight divided by body weight; BP, blood pressure; T.adip, peri-testis fat pad; P.adip, retroperitoneal fat pad; FPG, fasting plasma glucose; T.chol, total cholesterol; TG, triglycerides; NEFA, non-esterified fatty acid.

Table S10I. Pairwise phenotype comparison (in male and female rats) between RNO4 congenic strains involving SHR and WKY for sequential analysis

A pair of congenic strains	Hw (g)		Hw/Bw (‰)		12wk BP (mmHg)		16wk BP (mmHg)		16wk Bw (g)		T.adip./BW (‰)		P.adip./BW (‰)		FPG		T.chol		TG		NEFA	
	Male, P	Female, P	Male, P	Female, P	Male, P	Female, P	Male, P	Female, P	Male, P	Female, P	Male, P	Female, P	Male, P	Female, P	Male, P	Female, P	Male, P	Female, P	Male, P	Female, P	Male, P	Female, P
4SW2_4SW3	0.091	3.E-04	0.508	0.616	0.617	0.188	0.560	0.005	0.202	6.E-06	0.532		0.111	0.778	0.549	0.088	0.007	0.002	0.011	0.452	0.002	0.636
4SW3_4SW4	0.006	0.267	0.203	0.160	9.E-07	0.879	2.E-13	3.E-09	0.051	0.030	0.886		0.220	0.258	2.E-04	0.007	0.955	0.731	0.266	0.932	0.003	0.228
4SW4_4SW1	0.003	0.002	8.E-06	3.E-04	9.E-06	0.794	1.E-09	0.037	0.573	0.745	0.731		0.866	0.840	5.E-07	0.002	0.019	0.242	0.446	0.256	0.041	0.003
4SW1_4SW5	0.071	3.2E-07	5.E-04	2.E-04	0.966	0.535	0.159	0.393	0.745	0.133	0.025		0.001	0.003	0.681	0.297	2.E-04	6.E-04	0.083	0.003	0.102	3.E-05
4SW5_4SW6	7.E-15	0.620	4.E-16	0.012	0.691	0.256	0.011	0.413	0.549	0.017	2.E-09		9.E-07	0.002	0.685	0.759	8.E-16	0.550	2.E-06	0.051	3.E-12	6.E-05
4SW1_4SW	2.E-04	0.897	0.048	0.430	0.005	0.241	0.009	0.130	5.E-04	0.600	0.191		0.380	0.060	4.E-06	7.E-04	5.E-11	0.197	0.042	0.001	0.015	0.816

Hw, heart weight; Hw/Bw, heart weight divided by body weight; BP, blood pressure; T.adip, peri-testis fat pad; P.adip, retroperitoneal fat pad; FPG, fasting plasma glucose; T.chol, total cholesterol; TG, triglycerides; NEFA, non-esterified fatty acid.

Table S10J. Phenotype comparison in male rats between progenitor and RNO15 congenic strains involving SHR and WKY

Strain	*n*	Hw (g)		Hw/Bw (‰)		12wk BP (mmHg)		16wk BP (mmHg)		16wk Bw (g)		T.adip./BW (‰)		P.adip./BW (‰)		FPG		T.chol		TG		NEFA	
		mean ± SE	P, vs SHR	mean ± SE	P, vs SHR	mean ± SE	P, vs SHR	mean ± SE	P, vs SHR	mean ± SE	P, vs SHR	mean ± SE	P, vs SHR	mean ± SE	P, vs SHR	mean ± SE	P, vs SHR	mean ± SE	P, vs SHR	mean ± SE	P, vs SHR	mean ± SE	P, vs SHR
SHR	23-74	1.11 ± 0.01	—	3.42 ± 0.04	—	186.1 ± 1.6	—	197.6 ± 2.0	—	322.1 ± 2.1	—	12.00 ± 0.33	—	9.74 ± 0.33	—	123.8 ± 3.5	—	59.9 ± 0.9	—	12.4 ± 0.5	—	0.98 ± 0.04	—
WKY	10-27	0.98 ± 0.01	—	2.61 ± 0.03	—	130.3 ± 2.0	—	123.4 ± 2.3	—	366.6 ± 4.6	—	13.25 ± 0.31	—	10.72 ± 0.34	—	108.3 ± 3.3	—	83.1 ± 3.1	—	12.5 ± 1.3	—	1.30 ± 0.06	—
15SW2	34-74	1.06 ± 0.02	0.081	3.46 ± 0.04	0.404	172.0 ± 1.5	4.4E-09	179.3 ± 1.4	2.5E-11	315.5 ± 2.2	0.093	10.24 ± 0.21	3.5E-04	8.32 ± 0.25	0.010	129.1 ± 2.9	0.088	59.1 ± 0.8	0.334	11.6 ± 0.5	0.430	1.03 ± 0.05	0.5630
15SW	22-75	0.90 ± 0.02	9.3E-13	3.15 ± 0.02	1.7E-07	168.7 ± 1.9	8.9E-11	172.3 ± 1.9	1.8E-15	310.9 ± 3.0	0.003	11.14 ± 0.33	0.090	9.18 ± 0.36	0.289	137.8 ± 3.2	0.004	64.1 ± 1.2	0.006	13.3 ± 0.9	0.395	0.94 ± 0.03	0.3799
15SW1	34-67	1.05 ± 0.01	0.001	3.28 ± 0.04	0.009	170.3 ± 1.4	1.5E-11	177.2 ± 1.3	1.5E-13	323.7 ± 2.0	0.587	11.24 ± 0.19	0.053	8.72 ± 0.26	0.020	143.5 ± 3.3	8.8E-05	51.8 ± 0.8	2.2E-09	16.4 ± 0.9	2.8E-04	0.95 ± 0.05	0.6369

Hw, heart weight; Hw/Bw, heart weight divided by body weight; BP, blood pressure; T.adip, peri-testis fat pad; P.adip, retroperitoneal fat pad; FPG, fasting plasma glucose; T.chol, total cholesterol; TG, triglycerides; NEFA, non-esterified fatty acid.

Table S10K. Phenotype comparison in female rats between progenitor and RNO15 congenic strains involving SHR and WKY

Strain	*n*	Hw (g)		Hw/Bw (‰)		12wk BP (mmHg)		16wk BP (mmHg)		16wk Bw (g)				P.adip./BW (‰)		FPG		T.chol		TG		NEFA	
		mean ± SE	P, vs SHR	mean ± SE	P, vs SHR	mean ± SE	P, vs SHR	mean ± SE	P, vs SHR	mean ± SE	P, vs SHR			mean ± SE	P, vs SHR	mean ± SE	P, vs SHR	mean ± SE	P, vs SHR	mean ± SE	P, vs SHR	mean ± SE	P, vs SHR
SHR	26-85	0.74 ± 0.01	—	3.83 ± 0.05	—	161.9 ± 1.3	—	173.6 ± 1.6	—	190.9 ± 1.2	—			7.29 ± 0.19	—	120.5 ± 3.2	—	69.0 ± 1.1	—	10.8 ± 0.3	—	1.13 ± 0.04	—
WKY	10-15	0.62 ± 0.01	—	2.66 ± 0.03	—	116.0 ± 1.8	—	118.8 ± 2.3	—	227.2 ± 2.2	—			11.43 ± 0.4	—	108.9 ± 4.0	—	89.9 ± 3.1	—	15.3 ± 0.6	—	1.18 ± 0.08	—
15SW2	33-58	0.72 ± 0.01	0.054	3.78 ± 0.03	0.405	156.7 ± 1.4	0.008	164.2 ± 1.5	5.9E-05	196.8 ± 1.5	0.003			7.84 ± 0.15	0.108	128.7 ± 3.2	0.071	82.4 ± 1.5	1.5E-09	11.9 ± 0.5	0.047	0.94 ± 0.04	0.001
15SW	28-106	0.64 ± 0.01	3.0E-12	3.58 ± 0.03	1.4E-04	146.2 ± 1.6	3.6E-12	144.0 ± 1.4	7.7E-28	191.7 ± 1.4	0.663			6.95 ± 0.25	0.282	140.3 ± 2.7	7.2E-06	86.0 ± 1.1	2.4E-17	12.4 ± 0.5	0.008	1.15 ± 0.03	0.618
15SW1	33-63	0.70 ± 0.01	0.001	3.71 ± 0.02	0.044	149.6 ± 1.4	3.7.E-09	153.2 ± 1.2	8.1E-18	191.3 ± 1.3	0.845			5.99 ± 0.23	2.2E-06	144.1 ± 2.1	1.6E-08	72.0 ± 1.0	0.046	14.5 ± 0.4	1.2E-11	1.04 ± 0.04	0.107

Hw, heart weight; Hw/Bw, heart weight divided by body weight; BP, blood pressure; T.adip, peri-testis fat pad; P.adip, retroperitoneal fat pad; FPG, fasting plasma glucose; T.chol, total cholesterol; TG, triglycerides; NEFA, non-esterified fatty acid.

Table S10L. Pairwise phenotype comparison (in male and female rats) between RNO15 congenic strains involving SHR and WKY for sequential analysis

A pair of congenic strains	Hw (g)		Hw/Bw (‰)		12wk BP (mmHg)		16wk BP (mmHg)		16wk Bw (g)		T.adip./BW (‰)		P.adip./BW (‰)		FPG		T.chol		TG		NEFA	
	Male, P	Female, P	Male, P	Female, P	Male, P	Female, P	Male, P	Female, P	Male, P	Female, P	Male, P	Female, P	Male, P	Female, P	Male, P	Female, P	Male, P	Female, P	Male, P	Female, P	Male, P	Female, P
15SW2-15SW	1.0E-08	4.9E-10	4.E-08	3.0E-05	0.170	2.2E-06	0.004	9.0E-17	0.221	0.017	0.040	N/A	0.078	0.020	0.047	0.007	9.3E-04	0.064	0.113	0.488	0.105	4.9E-05
15SW-15SW1	1.7E-08	9.9E-07	0.003	0.002	0.484	0.125	0.034	1.6E-06	4.7E-04	0.813	0.823	N/A	0.345	0.002	0.223	0.266	8.E-12	4.8E-14	0.017	0.001	0.803	0.029

Hw, heart weight; Hw/Bw, heart weight divided by body weight; BP, blood pressure; T.adip, peri-testis fat pad; P.adip, retroperitoneal fat pad; FPG, fasting plasma glucose; T.chol, total cholesterol; TG, triglycerides; NEFA, non-esterified fatty acid.

Table S10M. Phenotype comparison in male rats between progenitor and RNO15 congenic strains involving SHRSP and WKY

Strain	*n*	Hw (g)		Hw/Bw (‰)		12wk BP (mmHg)		16wk BP (mmHg)		16wk Bw (g)		T.adip./BW (‰)		P.adip./BW (‰)		FPG		T.chol		TG		NEFA	
		mean ± SE	P, vs SHRSP	mean ± SE	P, vs SHRSP	mean ± SE	P, vs SHRSP	mean ± SE	P, vs SHRSP	mean ± SE	P, vs SHRSP	mean ± SE	P, vs SHRSP	mean ± SE	P, vs SHRSP	mean ± SE	P, vs SHRSP	mean ± SE	P, vs SHRSP	mean ± SE	P, vs SHRSP	mean ± SE	P, vs SHRSP
SHRSP	38-173	1.07 ± 0.01	—	3.82 ± 0.03	—	211.1 ± 1.0	—	236.9 ± 1.5	—	283.7 ± 1.7	—	11.05 ± 0.22	—	8.22 ± 0.23	—	133.9 ± 3.3	—	52.2 ± 1.0	—	15.7 ± 0.8	—	1.12 ± 0.06	—
WKY	10-27	0.98 ± 0.01	—	2.61 ± 0.03	—	130.3 ± 2.0	—	123.4 ± 2.3	—	366.6 ± 4.6	—	13.25 ± 0.31	—	10.72 ± 0.34	—	108.3 ± 3.3	—	83.1 ± 3.1	—	12.5 ± 1.3	—	1.30 ± 0.06	—
15pW2	27-63	1.05 ± 0.01	0.177	3.60 ± 0.02	9.3E-07	197.4 ± 1.2	2.4E-15	208.7 ± 1.6	3.2E-25	290.0 ± 1.7	0.005	10.86 ± 0.20	0.529	7.83 ± 0.26	0.263	145.2 ± 2.4	0.007	44.4 ± 1.2	4.5E-06	14.6 ± 0.8	0.340	0.89 ± 0.05	0.006
15pW	24-102	0.96 ± 0.01	1.8E-13	3.51 ± 0.03	4.3E-10	202.1 ± 2.0	7.3E-05	213.1 ± 1.7	2.0E-20	287.5 ± 1.9	0.094	9.65 ± 0.20	1.7E-04	8.36 ± 0.21	0.692	141.0 ± 3.5	0.096	64.7 ± 0.7	9.1E-16	13.1 ± 0.6	0.011	1.01 ± 0.04	0.132
15pW1	30-72	1.09 ± 0.01	0.225	3.52 ± 0.02	1.7E-11	197.4 ± 1.5	2.5E-12	211.2 ± 1.6	2.6E-23	311.6 ± 1.8	4.2E-24	10.67 ± 0.19	0.198	8.28 ± 0.18	0.825	132.7 ± 2.7	0.775	59.7 ± 2.7	0.013	14.7 ± 0.8	0.428	1.04 ± 0.04	0.319

Hw, heart weight; Hw/Bw, heart weight divided by body weight; BP, blood pressure; T.adip, peri-testis fat pad; P.adip, retroperitoneal fat pad; FPG, fasting plasma glucose; T.chol, total cholesterol; TG, triglycerides; NEFA, non-esterified fatty acid.

Table S10N. Phenotype comparison in female rats between progenitor and RNO15 congenic strains involving SHRSP and WKY

Strain	*n*	Hw (g)		Hw/Bw (‰)		12wk BP (mmHg)		16wk BP (mmHg)		16wk Bw (g)				P.adip./BW (‰)		FPG		T.chol		TG		NEFA	
		mean ± SE	P, vs SHRSP	mean ± SE	P, vs SHRSP	mean ± SE	P, vs SHRSP	mean ± SE	P, vs SHRSP	mean ± SE	P, vs SHRSP			mean ± SE	P, vs SHRSP	mean ± SE	P, vs SHRSP	mean ± SE	P, vs SHRSP	mean ± SE	P, vs SHRSP	mean ± SE	P, vs SHRSP
SHRSP	35-195	0.72 ± 0.01	—	3.86 ± 0.02	—	176.3 ± 0.8	—	190.8 ± 1.1	—	189.4 ± 0.8	—			6.92 ± 0.26	—	129.5 ± 1.9	—	69.7 ± 1.0	—	15.7 ± 0.6	—	0.88 ± 0.03	—
WKY	10-15	0.62 ± 0.01	—	2.66 ± 0.03	—	116.0 ± 1.8	—	118.8 ± 2.3	—	227.2 ± 2.2	—			11.43 ± 0.4	—	108.9 ± 4.0	—	89.9 ± 3.1	—	15.3 ± 0.6	—	1.18 ± 0.08	—
15pW2	31-74	0.71 ± 0.01	0.404	3.75 ± 0.02	0.001	170.5 ± 1.3	2.0E-04	177.8 ± 1.4	2.1E-11	188.5 ± 1.1	0.551			7.13 ± 0.19	0.503	145.4 ± 1.7	7.7E-09	53.2 ± 1.7	3.5E-11	14.1 ± 0.6	0.052	0.70 ± 0.03	1.0E-04
15pW	30-121	0.67 ± 0.01	1.3E-05	3.60 ± 0.02	6.5E-14	174.8 ± 1.7	0.424	179.6 ± 1.2	1.0E-10	186.3 ± 0.9	0.015			6.31 ± 0.28	0.083	141.0 ± 2.5	4.1E-04	90.1 ± 1.7	1.0E-14	16.7 ± 0.9	0.347	0.94 ± 0.04	0.157
15pW1	31-72	0.73 ± 0.01	0.305	3.71 ± 0.02	3.5E-08	171.9 ± 1.8	0.031	181.1 ± 1.7	4.1E-06	195.8 ± 1.4	1.2E-04			6.38 ± 0.21	0.098	137.3 ± 1.8	0.004	82.5 ± 3.2	0.001	14.6 ± 0.7	0.234	0.88 ± 0.04	0.928

Hw, heart weight; Hw/Bw, heart weight divided by body weight; BP, blood pressure; T.adip, peri-testis fat pad; P.adip, retroperitoneal fat pad; FPG, fasting plasma glucose; T.chol, total cholesterol; TG, triglycerides; NEFA, non-esterified fatty acid.

Table S10O. Pairwise phenotype comparison (in male and female rats) between RNO15 congenic strains involving SHRSP and WKY for sequential analysis

A pair of congenic strains	Hw (g)		Hw/Bw (‰)		12wk BP (mmHg)		16wk BP (mmHg)		16wk Bw (g)		T.adip./BW (‰)		P.adip./BW (‰)		FPG		T.chol		TG		NEFA	
	Male, P	Female, P	Male, P	Female, P	Male, P	Female, P	Male, P	Female, P	Male, P	Female, P	Male, P	Female, P	Male, P	Female, P	Male, P	Female, P	Male, P	Female, P	Male, P	Female, P	Male, P	Female, P
15pW2-15pW	5.7E-09	5.6E-06	0.015	1.9E-05	0.042	0.044	0.061	0.332	0.332	0.124	0.001	N/A	0.165	0.007	0.264	0.147	5.9E-19	5.7E-23	0.118	0.014	0.060	7.9E-06
15pW-15pW1	7.0E-15	8.4E-08	0.851	0.001	0.057	0.244	0.413	0.482	8.E-17	6.0E-08	0.003	N/A	0.813	0.825	0.032	0.243	0.080	0.042	0.108	0.065	0.587	0.266

Hw, heart weight; Hw/Bw, heart weight divided by body weight; BP, blood pressure; T.adip, peri-testis fat pad; P.adip, retroperitoneal fat pad; FPG, fasting plasma glucose; T.chol, total cholesterol; TG, triglycerides; NEFA, non-esterified fatty acid.

Table S11A. Chromosomal fragments introgressed into SHRSP/SHR-derived RNO1 congenic rat strains

Marker	Position: Rnor_6.0*	Allele			SHRSP-derived																SHR-derived	
		SHRSP	SHR	WKY	1pW	1pWC	1pW2	1pW5-2	1pW4-1	1pW4-2	1pW4-3	1pW4-4	1pW6-2	1pW6-1	1pW3-2	1pW3-1	1pW8	1pW10	1pW9	1pW11	1SW	1SWC
D1Mgh2	23,406,428	145	145	153	○																○	○
D1Rat196	58,354,072	192	192	190		✻	✻	✻	✻	✻												
D1Mgh5	79,689,548	157	161	161		○	○	○	○	○												
D1Rat178	88,429,368	124	120	120						○	✻											
D1Rat224	93,410,058	166	166	169						✻	✻											
D1Rat27	94,201,400	182	184	187							○											
intergenic	98,018,170	–	–	TATATATACAC					○		○	☆										
rs64143603	98,394,078	G/G	G/G	T/T					○		○	○										
Klk1b3 (Ngfg_2)	100,198,334	T.T	T.T	del.A					○		○	○										
intergenic	102,082,430	C/C	del	del					○		○	○										
rs105531410	102,691,817	C/C	C/C	A/A					✻		✻	✻										
D1Rat348	135,005,392	119	119	129									✻									
D1Rat36	135,022,396	120	120	136									○									
D1Rat37	135,040,605	122	122	126										✻								
D1Rat40	135,428,373	122	122	130										○								
D1Rat270	135,611,773	215	215	238				○														
D1Tkyo26 (*Ntrk3*)	139,865,346	135	135	96				✻							✻							
D1Mit2	140,953,686	148	148	146											✻							
D1Tkyo27 (*Cib1*)	142,014,962	84	84	109											○							
D1Rat106	145,140,412	139	139	133			○						○	○		✻						
D1Tkyo32 (*Ctsc*)	151,918,514	175	180	171			✻						○	✻		✻						
D1Rat44	161,493,758	120	158	130									✻			○						
D1Rat164	177,235,071	221	221	240													✻					
D1Smu11 (*Calca*)	184,185,140	186	186	184													○					
D1Rat109	199,953,026	153	153	157											○	○	○					
D1Rat120	202,111,380	211	211	199											○	○	○					
D1Wox22	215,830,072	144	144	135											✻	✻	✻					
D1Rat71	230,420,627	143	148	148														✻	✻			
D1Rat116	232,297,062	165	175	175														○	○			
D1Rat117	233,490,105	132	125	125		○												○	○			
D1Wox10	236,763,415	120	116	114		✻												○				○
intergenic	237,963,635	C/C	T/T	T/T														○	○			
Tmc1 (intron)	238,523,462	A/A	A/A	G/G															✻			
D1Tkyo49 (*Insl6*)	247,468,493	183	176	183																		✻
intergenic	252,270,835	T/T	T/T	C/C															✻			
intergenic	252,279,320	T/T	T/T	G/G															○			
D1Tkyo56 (*Scd*)	264,157,471	268	266	266														✻				
rs198971711	269,979,231	T/T	T/T	C/C														✻		✻		
D1Rat376 (*Sorcs1*)	270,122,139	219	219	217																✻		
D1Tkyo58 (*Add3*)	273,856,421	171	171	163																○		
D1Rat90	281,795,624	156	156	128	○													✻	○	○	○	

Symbols show variants characterized on the border of congenic fragments: open circle (○) for the WKY allele and asterisk (✻) for the recipient (SHRSP or SHR) allele.

*For SSLP markers, the position of 5'-primer site is shown.

Table S11B. Chromosomal fragments introgressed into SHRSP/SHR-derived RNO3 congenic rat strains

Marker	Position: Rnor_6.0*	Allele			SHRSP-derived						SHR-derived						
		SHRSP	SHR	WKY	3pW1	3pW4	3pW	3pW5	3pW3	3pW2	3SW1	3SW4	3SW	3SW5	3SW3	3SW6	3SW2
D3Mgh16	6,000,748	135	135	121	○	○	○				○	○	○				
D3Tkyo4 (*Kynu*)	28,448,867	158	152	144													✻
D3Mit9	34,394,121	248	248	250													○
D3Rat80	36,599,933	272	242	212				✻		✻							
D3Rat186	45,228,240	140	140	147				○		○							
D3Rat110	47,155,167	133	133	117	○						○			✻			
D3Mgh6	54,630,948	109	109	106	✻						✻			○		✻	
D3Rat108	63,849,481	248	248	228												○	
D7Rat130	94,187,793	155	155	171													○
D3Mit16	97,537,495	293	293	289											✻		✻
D3Mgh5	103,141,814	136	136	128					✻						○		
D3Rat166	106,900,427	186	186	175					○	○		○				○	
D3Mit14	120,917,788	196	196	202						✻		✻				✻	
D3Mgh15	123,599,592	123	123	109		○											
D3Rat157	128,716,357	178	178	200		✻											
D3Rat1	176,417,943	138	138	148			○	○	○				○	○	○		

Symbols show variants characterized on the border of congenic fragments: open circle (○) for the WKY allele and asterisk (✻) for the recipient (SHRSP or SHR) allele.

*For SSLP markers, the position of 5'-primer site is shown.

Table S11C. Chromosomal fragments introgressed into SHR-derived RNO4 congenic rat strain

Marker	Position: Rnor_6.0*	Allele			SHR-derived							
		SHRSP	SHR	WKY	4SW	4SW6	4SW5	4SW1	4SW4	4SW3	4SW2	
D4Wox27	3,044,017	109	109	119	○			○	○	○	○	
D4Rat4	4,364,715	186	186	180			✻					
D4Rat136	9,657,095	153	153	159			○					Cd36(chr4:14,150,309)
intergenic	21,827,813	T	T	C							○	
D4Rat10	26,753,655	175	173	171							✻	
intergenic	31,561,535	T	T	A						○		
intergenic	31,782,622	T	T	C						✻		
D4Rat89	34,418,190	175/180	182	180/184		✻			○			
intergenic	42,486,449	T	T	C		○			✻			
D4Rat15	48,656,252	149	149	165	○			○				
intergenic	49,200,013	C	C	T		○	○	○				
intergenic	49,492,085	A	A	C		✻	✻	✻				
D4Mgh11	168,046,938	158	158	151	○							

Symbols show variants characterized on the border of congenic fragments: open circle (○) for the WKY allele and asterisk (✻) for the recipient (SHRSP or SHR) allele.

*For SSLP markers, the position of 5'-primer site is shown.

Table S11D. Chromosomal fragments introgressed into SHR-derived RNO15 congenic rat strains

Marker	Position: Rnor_6.0*	Allele			SHRSP-derived			SHR-derived		
		SHRSP	SHR	WKY	15pW2	15pW	15pW1	15SW2	15SW	15SW1
D15Rat2	11,605,245	196	172	172		○	○			
D15Tkyo3 (*Fhit*)	14,053,354	256	270	256					○	○
D15Mgh7	18,513,913	142	142	144						
D15Rat20	59,616,275	156	148	148	✻					
D15Rat39	62,315,021	240	244	240				✻		
D15Rat68	63,498,932	132	134	150	○			○		○
D15Arb2	68,366,508	221	229	229						
D15Rat95	70,559,420	226	226	208						✻
D15Rat94	77,072,146	185	185	173			○			
D15Tkyo18 (*Ednrb*)	88,060,793	254	254	256			✻			
D15Rat106	106,550,444	220	220	182	○	○		○	○	

Symbols show variants characterized on the border of congenic fragments: open circle (○) for the WKY allele and asterisk (✻) for the recipient (SHRSP or SHR) allele.

*For SSLP markers, the position of 5'-primer site is shown.

Table S12. Inter-strain comparison of quantitative PCR in the kidney for selected genes located in the 1q21-1q22 region

		Normal rat chow									Enalapril administration							
		1pW/SHRSP		1pW4-1/SHRSP		1pW4-3/SHRSP		1pW4-4/SHRSP			WKY			SHRSP				
Gene [a]	Position on RNO1 (v6.0)	Fold change (congenic / SHRSP)	*P* value	Fold change (congenic / SHRSP)	*P* value	Fold change (congenic / SHRSP)	*P* value	Fold change (congenic / SHRSP)	*P* value	Ave. quantity [b]	Fold change (Drug/ control)	*P* value	Ave. quantity [b]	Fold change (Drug/ control)	*P* value	Ave. quantity [b]	Forward primer seq. (5'-to-3')	Reverse primer seq. (5'-to-3')
Uri1	94,346,513 - 94,404,362 (-)	0.77	2E-06	0.95	0.222	0.84	0.009	1.00	0.937	0.012	–	–	–	–	–	–	AAGAGAACCACCCCAAGAAG	ATGAGCAATGGCTGAGTGTG
Ccne1	94,485,830 - 94,495,112 (-)	1.23	0.074	0.66	0.0002	0.68	0.0009	0.78	0.038	0.0004	–	–	–	–	–	–	TTCCAAGCTCAAGCACTTCC	ACTCGGAGGAGGAGAAATCC
LOC690000	94,572,714 - 94,587,842 (+)	0.82	0.051	0.76	0.010	0.79	0.016	1.20	0.223	0.004	–	–	–	–	–	–	TGCCCATCATGGTAGATGAC	GCCGCCTTCATTTTCTTCTC
Plekhf1	94,609,041 - 94,616,565 (-)	1.25	0.014	1.15	0.104	1.24	0.003	1.02	0.740	0.008	–	–	–	–	–	–	ATGACGATGACTCCGATGAG	TGACCAGGAGACACCAGAAG
*Zfp715**	98,614,943 - 98,631,142 (+)	1.12	0.260	0.92	0.594	1.00	0.954	0.92	0.488	0.003	–	–	–	–	–	–	TATGTGTGTGCCGAATGTGG	GGATGTCACTTGTGAATTCTGC
Klk1c12	99,298,965 - 99,303,048 (-)	0.95	0.383	1.29	0.063	0.86	0.081	1.03	0.592	0.001	–	–	–	–	–	–	TTTAAAGATGAACCCTTTGCAC	AGCATCAGGTCATTGCTGTG
Klk1c8 (Klk1b21)	99,352,114 - 99,355,976 (+)	1.44	0.0007	1.11	0.455	0.73	0.007	1.19	0.135	0.001	–	–	–	–	–	–	AGCATCACACCCCTCAAATG	TATCTTTGCCCCCATCCATC
Klk15	99,393,283 - 99,401,339 (+)	0.88	0.553	0.36	0.003	0.69	0.059	1.90	0.025	6.9E-05	0.68	0.040	4.3E-05	1.03	0.850	3.2E-05	GTGACTCTGGAGGACCCTTG	ACTCCAGGCTTGGTGGTAGTATC
Klk1c10	99,890,294 - 99,894,353 (-)	5.07	6E-06	3.13	3E-05	2.60	3E-05	4.03	2E-06	0.0002	1.67	0.009	3E-05	1.19	0.570	0.0003	CACCAAACCCCTGAATTGG	GAGATCTGTCACCTTCTGTTCGT
Klk1c4 (LOC100364577)	94,412,788 - 94,418,026	10.50	0.0004	5.26	0.001	3.74	0.001	9.92	0.0005	0.0002	1.49	0.049	3.8E-05	1.99	0.006	0.0006	TCATGCTGTGTGCAGGAAAG	TGTAGATGCCTGGGTTATGG
Klk1c6 (Gk11)	99,947,714 - 99,964,824 (-)	1.01	0.804	0.77	0.004	0.72	0.197	0.75	0.002	0.044	–	–	–	–	–	–	TGCTGTGTGCAGGAGATTTG	GGTATAGATGGCTGGCATATTG
Klk1c2 (Ton)	99,981,045 - 99,985,422 (-)	1.18	0.481	0.84	0.338	0.58	0.080	3.07	0.017	1.5E-05	1.25	0.027	4.9E-06	1.11	0.340	1E-05	GCTTCCGACACCCTGACTATATC	GTCATTGCTGTGGTCATGCAC
Klk5l	100,055,956 - 100,058,334 (+)	1.49	0.572	1.62	0.301	0.39	0.156	14.73	0.044	1.4E-06	1.99	0.026	1.2E-05	1.89	0.006	1.1E-05	AGGATCCATAGAGGCTGCAAG	CTGTGAGTGCTCATTGTGGTTG
Klk1c9 (Klks3)	100,086,520 - 100,090,406 (+)	2.50	0.002	1.23	0.122	0.97	0.736	1.90	0.009	0.006	1.63	0.007	0.003	1.68	0.002	0.016	GCAAGTGGCTGTCATTGGTAC	CCAGCAAAACACGATAATTCTTG
Klk1c7	100,131,562 - 100,135,556 (+)	1.01	0.969	0.90	0.611	0.59	0.067	1.05	0.811	0.232	–	–	–	–	–	–	CAGTCAAAGCTTCCCTCACC	GATCATTGCTGTGGTCATCG
Klk1c3	100,141,205 - 100,163,945 (+)	0.70	0.453	0.63	0.403	1.14	0.735	0.91	0.857	3.3E-06	–	–	–	–	–	–	TGCTATAGTGATGCCCTCAGC	CATCAGGTCATTGCTGTAGTCCT
Klk1b3 (Klk1c1, Ngfg)	100,199,057 - 100,203,260 (+)	0.93	0.616	0.75	0.087	0.67	0.032	0.85	0.271	0.337	–	–	–	–	–	–	CCCTGGGACGGAATGATG	GCCGAAGTAGTACACAGCCACTT
Dbp	101,687,896 - 101,692,845 (+)	1.28	0.061	0.48	0.005	0.94	0.624	0.92	0.576	0.053	–	–	–	–	–	–	GGTCCTGTGTGCACTTTTAGC	TTCAGGATTGTGTTGATGGAG

1pW, an RNO1 consomic strain derived from SHRSP/Izm; 1pW4-1, 1pW4-3 and 1pW4-4, RNO1 congenic strains derived from SHRSP/Izm.

qPCR is performed on the kidney of progenitor and consomic/congenic strains (n=5 per strain) for genes listed in the table with the primers originally designed. For part of the *Klk1* papralogs (or *Klk* homologs), the effect of enalapril administration is examined in progenitor strains. Inter-strain comparison is done with unpaired *t*-test.

[a] Genes with asterisks are annotated in Ensembl but not in Entrez database. In some cases, aliases are shown in the parenthesis.

[b] The average quantity is arbitrarily defined for reference (or control) rats as a ratio of mRNA expression level of tested gene to *Ppia*.

Supplementary Table 13. Differential expression of candidate genes in the known BP regulatory pathways

Agilent probe	Gene name	Chr	Transcription _start_site (Rnor_6.0)	SHRSP/Izm (versus WKY/Izm)										SHR/Izm (versus WKY/Izm)									
				Heart		Aorta		Renal cortex		Whole kidney		Liver		Heart		Aorta		Renal cortex		Whole kidney		Liver	
				log_2FC (SHRSP /WKY)	P-value	log_2FC (SHRSP /WKY)	P-value	log_2FC (SHRSP /WKY)	P-value	log_2FC (SHRSP /WKY)	P-value	log_2FC (SHRSP /WKY)	P-value	log_2FC (SHRSP /WKY)	P-value	log_2FC (SHRSP /WKY)	P-value	log_2FC (SHRSP /WKY)	P-value	log_2FC (SHRSP /WKY)	P-value	log_2FC (SHRSP /WKY)	P-value
Kallikrein-kinin & renin-angiotensin systems																							
A_44_P1029253	*Mas1*	1	48,077,033	N/A		0.458	0.148	-0.25	0.207	N/A		N/A		N/A		0.159	0.482	0.002	0.992	0.056	0.772	N/A	
A_64_P337849	*Lnpep*	1	68,436,593	-0.166	0.002	-0.253	0.017	-0.014	0.864	0.155	0.337	0.699	0.01	-0.117	0.247	0.39	0.002	-0.105	0.232	-0.043	0.703	0.389	0.116
A_64_P108993	*Klk11*	1	99,712,108	N/A		N/A		N/A		N/A		N/A		N/A		N/A		N/A		-0.288	6E-04	N/A	
A_44_P280954	*Klk1c10*	1	99,894,353	N/A		N/A		-1.442	2E-07	N/A		N/A		N/A		N/A		-1.175	5E-06	-1.175	1E-08	N/A	
A_44_P163243	*Klk1c9 (Klks3)*	1	100,059,967	N/A		N/A		-2.715	1E-12	-2.088	2E-09	N/A		N/A		N/A		-1.414	1E-08	-1.604	4E-10	N/A	
A_44_P399414	*Klk1c7 (Klk1l)*	1	100,059,967	0.17	0.213	-1.618	9E-04	-0.296	0.001	-0.204	0.031	N/A		0.542	0.046	-2.492	4E-05	-0.141	0.091	-0.237	0.001	N/A	
A_44_P374824	*Klk1c3*	1	100,161,872	0.029	0.847	-0.723	0.174	-0.285	0.001	0.048	0.599	N/A		0.236	0.436	-2.412	0.01	-0.183	0.031	-0.152	0.026	N/A	
A_44_P500013	*Anpep*	1	141,564,994	-0.118	0.051	0.527	0.001	-0.398	0.002	-0.199	0.064	0.188	0.164	0.009	0.939	-0.01	0.948	-0.305	0.018	-0.109	0.145	0.358	0.015
A_64_P113184	*Prcp*	1	157,701,089	-0.101	0.346	-0.069	0.598	-0.113	0.209	-0.271	0.234	N/A		-0.411	0.034	-0.711	1E-04	-0.241	0.031	0.017	0.908	N/A	
A_64_P109894	*Agtr1b*	2	105,224,295	N/A		-0.085	0.75	0.072	0.527	0.142	0.438	N/A		N/A		0.392	0.177	0.029	0.809	-0.071	0.581	N/A	
A_44_P209788	*Enpep*	2	233,743,866	-0.161	0.005	0.655	1E-04	-0.131	0.064	-0.118	0.257	-0.141	0.331	-0.234	0.037	0.094	0.528	-0.064	0.37	-5E-04	0.995	-0.329	0.036
A_64_P114713	*Agtrap*	5	164,898,420	-3.658	2E-36	-3.665	7E-13	-4.091	1E-18	-3.505	7E-21	-4.448	2E-10	-0.142	0.297	-0.095	0.571	-0.072	0.314	0.053	0.478	-0.236	0.043
A_64_P036715	*Bdkrb2*	6	129,399,468	0.139	0.339	-0.046	0.816	N/A		0.645	0.036	N/A		0.446	0.041	0.085	0.643	N/A		0.216	0.282	N/A	
A_43_P10861	*Dnpep*	9	82,514,399	0.32	5E-06	0.14	0.131	-0.19	0.04	-0.1	0.417	-0.115	0.526	0.097	0.422	-0.333	0.004	-0.272	0.009	-0.331	8E-04	-0.307	0.106
A_64_P010531	*Ace*	10	94,170,766	0.188	0.063	0.807	6E-06	-1.53	2E-04	-1.955	5E-10	N/A		0.871	8E-05	0.189	0.186	-0.088	0.785	0.244	0.081	N/A	
A_64_P098230	*Ace*	10	94,170,766	0.091	0.209	0.288	0.058	-1.398	1E-05	-1.372	6E-09	-0.291	0.106	0.873	4E-07	0.222	0.179	-0.139	0.551	0.334	0.005	-0.028	0.871
A_44_P348781	*Kng1*	11	81,444,621	N/A		N/A		-1.82	6E-11	-1.09	2E-04	0.071	0.679	N/A		N/A		-0.469	7E-04	-0.87	4E-05	0.194	0.269
A_64_P059056	*Ren*	13	50,514,151	-0.059	0.468	0.14	0.282	0.056	0.534	-0.09	0.421	-0.295	0.137	0.554	0.001	-0.026	0.857	0.556	4E-05	0.483	3E-06	-0.79	1E-03
A_42_P486203	*Agtr1a*	17	35,958,077	-0.236	0.002	0.405	0.002	-0.281	0.003	0.016	0.893	-0.576	0.001	0.081	0.562	0.18	0.154	-0.193	0.039	-0.153	0.079	-0.478	0.005
A_64_P154849	*Agt*	19	57,333,433	-0.883	2E-05	0.094	0.534	-0.073	0.701	3E-04	0.998	0.138	0.228	-0.13	0.717	-2.88	8E-12	-1.126	6E-05	-0.915	1E-09	-0.051	0.646
A_43_P14875	*Prep*	20	50,172,618	-0.225	5E-10	-0.687	3E-08	-0.386	0.044	-0.462	9E-05	0.128	0.352	-0.22	2E-04	-0.254	0.005	-0.888	3E-04	-0.678	1E-09	-0.159	0.254
A_44_P1011008	*Atp6ap2*	X	11,164,915	0.12	0.004	0.63	4E-04	-0.035	0.577	0.039	0.736	-0.156	0.167	0.129	0.107	0.063	0.694	-0.074	0.272	-0.03	0.713	0.066	0.544
A_64_P132696	*Ace2*	X	32,096,016	-0.058	0.325	-0.583	0.001	-0.182	0.407	0.011	0.943	N/A		0.662	2E-06	-0.887	7E-05	-0.487	0.049	-0.429	4E-04	0.17	0.174
Sympathetic nervous system																							
A_44_P491621	*Adrbk1 (Grk2)*	1	219,544,328	0.105	0.087	-0.175	0.015	-0.011	0.892	0.075	0.587	-7E-04	0.995	-0.145	0.231	-0.189	0.019	-0.08	0.379	-0.187	0.065	-0.136	0.274
A_64_P130105	*Adrb1*	1	277,537,585	-0.443	2E-07	-0.363	0.049	0.012	0.933	-0.11	0.331	0.635	0.039	-0.372	0.011	0.089	0.648	0.084	0.578	-0.234	0.009	0.402	0.17
A_64_P053785	*Adra2a*	1	274,766,283	N/A		N/A		N/A		0.087	0.61	N/A		N/A		N/A		N/A		0.435	0.002	N/A	
A_64_P138008	*Adra2b*	3	119,805,941	0.073	0.391	-0.537	0.009	0.461	4E-04	-0.035	0.807	-0.039	0.746	-0.043	0.802	-0.419	0.054	0.436	0.001	0.216	0.043	0.146	0.242
A_42_P501233	*Adra1d*	3	124,145,566	-2.744	4E-25	-0.549	0.002	0.112	0.502	0.634	0.048	N/A		-1.515	2E-08	-0.325	0.071	-0.161	0.364	-0.77	0.002	N/A	
A_64_P138011	*Adra1b*	10	29,450,644	-0.33	8E-09	0.901	1E-03	0.172	0.087	0.204	0.148	0.325	0.002	-0.276	0.004	0.467	0.079	0.498	2E-04	0.468	7E-05	7E-04	0.994
A_64_P053790	*Adra1a*	15	43,298,794	-0.641	4E-11	-1.073	3E-05	-0.134	0.148	-0.303	0.129	N/A		-0.388	0.008	-0.472	0.04	0.037	0.696	-0.104	0.455	N/A	
A_64_P150549	*Adrb3*	16	69,003,868	-0.985	0.004	0.743	0.002	N/A		-0.384	0.336	0.622	0.015	N/A		0.709	0.006	N/A		-0.867	0.039	0.681	0.029
A_44_P188280	*Adrb2*	18	57,515,834	0.23	4E-04	1.239	1E-05	0.201	0.051	0.374	0.282	0.464	0.001	1.011	3E-04	-0.161	0.179	-0.177	0.099	-0.34	0.169	0.21	0.08
Natriuretic peptide system																							
A_44_P317600	*Nppa*	5	164,808,323	1.277	5E-13	N/A		-0.102	0.409	N/A		N/A		2.413	2E-12	N/A		-0.218	0.115	-0.26	0.01	N/A	
A_42_P638494	*Nppb*	5	164,796,185	1.343	2E-21	N/A		N/A		N/A		N/A		1.146	2E-10	N/A		N/A		N/A		N/A	
A_44_P240696	*Nppc*	9	93,735,636	N/A		N/A		N/A		N/A		N/A		N/A		N/A		N/A		N/A		N/A	
A_43_P15245	*NPR1 (Gca)*	2	189,856,090	-0.437	8E-12	0.521	2E-05	-0.151	0.076	0.043	0.709	-0.064	0.455	-0.142	0.116	0.701	3E-06	-0.035	0.68	0.057	0.487	0.005	0.952
A_42_P726573	*NPR2*	5	59,128,315	-0.167	0.002	0.126	0.277	-0.49	2E-05	-0.185	0.269	0.182	0.262	-0.159	0.12	0.438	0.003	-0.33	0.002	-0.441	9E-04	0.293	0.082
A_64_P036616	*NPR3*	2	61,949,926	0.068	0.456	-2.398	4E-07	0.576	8E-04	0.499	0.03	N/A		0.499	0.009	-0.25	0.451	0.466	0.006	0.273	0.087	N/A	
A_42_P641962	*Pcsk6*	1	126,749,508	-0.256	3E-05	0.12	0.317	-0.331	0.294	-0.521	3E-04	-0.634	6E-04	-0.14	0.198	0.051	0.698	-0.38	0.258	0.06	0.493	-0.626	7E-04
A_64_P021014	*Pcsk6*	1	126,749,508	-0.083	0.328	-0.175	0.34	N/A		0.087	0.708	0.115	0.682	-0.175	0.299	-0.344	0.103	N/A		0.114	0.474	-0.112	0.713
A_64_P160586	*Furin*	1	142,197,182	-8E-04	0.988	0.075	0.288	0.067	0.282	0.186	0.101	0.407	0.009	-0.075	0.464	-0.082	0.297	-0.05	0.446	0.169	0.038	0.349	0.021
A_64_P150662	*Corin*	14	38,247,791	-0.198	0.014	2.024	5E-10	N/A		N/A		N/A		-0.066	0.672	2.078	2E-09	N/A		N/A		N/A	
A_43_P11484	*Mme (Nep)*	2	153,803,349	-0.586	5E-09	-1.281	7E-08	-0.294	0.055	-0.242	0.026	0.157	0.517	0.48	0.004	-0.372	0.027	-0.084	0.582	-0.09	0.222	-0.053	0.825

Gene expression data in the table are produce by microarray analysis. Fold changes (hypertensive strain vs WKY; log2-transformed values) are shown in each cell.

For Ace and Corin, the microarray includes two distinct probes, for each of which the expression data are shown in the table.

N/A, data not available because of low mRNA expression of the gene.

Table S14. A list of primers for quantitative RT-PCR

Gene Symbol	Forward primer (5'-to-3')	Reverse primer (5'-to-3')	Original design or drawn from literature
Nppa	CTCCCAGGCCATATTGGAG	TCCAGGTGGTCTAGCAGGTT	PMID: 27161004
Nppb	GCTCTCAAAGGACCAAGGC	CCTAAAACAACCTCAGCCCG	Original
Ren	CTCTGGGCACTCTTGTTGCTC	GCACTGATCCTGGTCATGTCTAC	Original
Prep	ATTTGCGACCCTTATGCTTG	TTCTGTGCCTCCACGAAAG	Original
Ace	TTGTCTGTCACTGGAGCCTGAT	CACCCAAAGCAATTCTTCGTATT	Original
Kng2	TCACAGGTGGTTGCTGGAGT	CCGAGGTGTTTGGTCTCACAA	Original
Klk1c10	TGGTGTGCTTCAAGGCATCA	GACAGTGTGACACTCAGGGG	Original
Homer2	GGTCCGAGAAAAGGAAATGG	GAGACCGCACTTTGTCTTCC	Original
Endog	CCAATCACCGCTGGAGTCA	AGGCCCTGTGCAGACATAAAC	Original
Sgk1	AGATCACGCCCCCATTTAAC	TGTGACAAGGATGCTGTCAG	Original
Cd36	TCAAGGTGTGCTCAACAGCC	ACTCCATCTACAGTGTTATTG	PMID: 18587397

Disease Models & Mechanisms • Supplementary information
